# A Co-conformationally “Topologically” Chiral Catenane

**DOI:** 10.1021/jacs.2c02029

**Published:** 2022-06-28

**Authors:** Arnau Rodríguez-Rubio, Andrea Savoini, Florian Modicom, Patrick Butler, Stephen M. Goldup

**Affiliations:** Chemistry, University of Southampton, Highfield, Southampton SO17 1BJ, United Kingdom

## Abstract

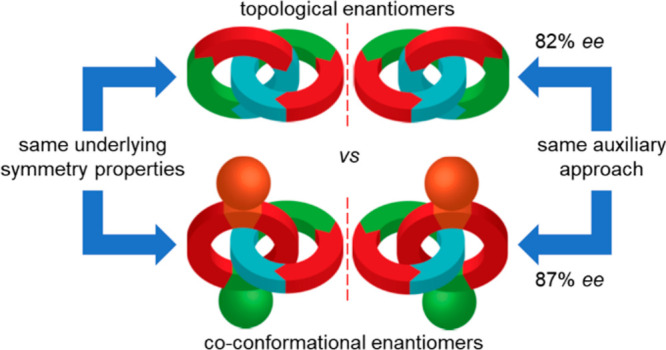

Catenanes composed of two achiral rings that are oriented (C_nh_ symmetry) because of the sequence of atoms they contain are referred to as topologically chiral. Here, we present the synthesis of a highly enantioenriched catenane containing a related but overlooked “co-conformationally ‘topologically’ chiral” stereogenic unit, which arises when a bilaterally symmetric C_nv_ ring is desymmetrized by the position of an oriented macrocycle.

Topology is the study of the properties of objects and networks that are preserved under deformations that do not break connections/surfaces or require surfaces/edges to pass through one another. Chemical topology applies these ideas to molecules.^1^ At the simplest level, constitutional isomers are topologically distinct, as they differ in the network of atoms. More interesting topological isomerism arises when structures contain identical atomic connections,^2^ the most famous examples of which are Möbius ladders (isomers of the untwisted macrocycle),^3,4^ molecular knots (isomers of the unknotted ring),^5^ and [2]catenanes (isomers of two non-interlocked rings).^6^ These structures have nonplanar graphs in that there is no two-dimensional projection of their structures in which bonds do not cross over one another and this property is topologically invariant in three-dimensional space—no matter how the structure is distorted, even drastically altering the geometry around atoms, a planar graph cannot be achieved.^1^

Such topologically nontrivial structures can display chirality in the absence of covalent stereogenic units.^2^ Depending on their topology, Möbius ladders^7^ and molecular knots^8^ can be chiral as a result of the pattern of bond crossing points. Although [2]catenanes do not display unconditional topological stereochemistry,^9^ as recognized by Wasserman and Frisch,^10^ they can be chiral as a result of the constitutional symmetry of the rings; rings that are “oriented” (C_nh_ symmetry) due to the sequence of atoms in the cycle give rise to topologically chiral catenanes (Figure 1a).^11,12^ The absolute stereochemistry of topologically chiral objects is invariant under all topologically allowed deformations in three-dimensional space.^1^

**Figure 1:**
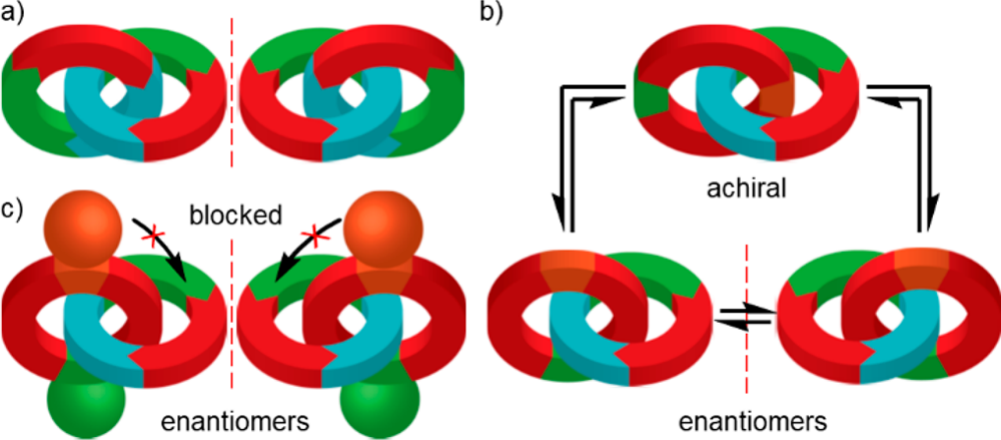
(a) Enantiomeric topologically chiral catenanes (two oriented C_1h_ rings). (b) Achiral and enantiomeric co-conformations of a co-conformationally “topologically” chiral [2]catenane (oriented ring and a C_2v_ ring). (c) Fixed enantiomeric chiral co-conformations of a co-conformationally “topologically” chiral catenane for which co-conformational isomerism is sterically prohibited.

We recently identified^11c^ “missing” stereogenic units that arise in interlocked molecules and give rise to classes of chiral rotaxanes and catenanes that had yet to be discussed or synthesized.^13^ An example that presents particular linguistic problems are [2]catenanes in which one ring is oriented (C_nh_) and the other is bilaterally symmetric (e.g., C_2v_) (Figure 1b). The time averaged structure of such catenanes is achiral, but any co-conformation in which the oriented ring does not lie on the internal mirror plane of the C_2v_ ring is chiral. If the structure is designed such that the oriented ring is permanently prevented from occupying said mirror plane, the molecule will display kinetically fixed molecular chirality (Figure 1c).

As with related co-conformational-covalent^14^ and co-conformational mechanical planar stereochemistry in rotaxanes,^15,16^ this stereogenic unit can be considered to appear due to the oriented ring acting as a substituent of the region of C_2v_ ring that it encircles, effectively reducing its symmetry to C_1h_. Thus, this stereogenic unit arises because one ring is oriented due to its constitution and the other by the molecular co-conformation and so we have previously provisionally termed such molecules “co-conformationally “topologically” chiral” to clearly make the link with the established stereogenic unit of topologically chiral catenanes while also highlighting that the stereochemistry of the system is clearly not topologically invariant.

Semantic arguments aside, we set out to synthesize an enantioenriched co-conformationally “topologically” chiral [2]catenane, in part to highlight the potential for interlocked molecules to display hitherto unnoticed stereochemistry. To achieve this, we developed a stereoselective synthesis of topologically chiral [2]catenanes, which was then extended to a co-conformationally chiral target.

The stereoselective synthesis of a co-conformationally chiral catenane requires (i) the oriented ring to be incorporated at a defined position around the C_2v_ macrocycle and (ii) the oriented ring to be installed stereoselectively. The first requirement can be met by forming the mechanical bond such that the oriented ring is trapped between bulky groups. The second is the same problem as encountered in the synthesis of any topologically chiral [2]catenane.^17^ Although the majority of enantioenriched topologically chiral catenanes in which the mechanical bond is the sole source of stereochemistry^18^ have been accessed by chiral stationary phase HPLC (CSP-HPLC) separation,^12^ we recently developed an auxiliary approach in which a chiral covalent auxiliary directs the stereoselective formation of the mechanical bond.^19^ However, in this proof-of-concept synthesis, the stereoselectivity of the mechanical bond formation was low (*dr* ∼ 2:1), which required the mechanical epimers to be separated prior to removal of the auxiliary, limiting the utility of this methodology for more complicated targets. To overcome this challenge, we set out to extend a phenylalanine-based auxiliary, developed for the synthesis of mechanically planar chiral rotaxanes,^20,21^ to the synthesis of topologically chiral [2]catenanes.

Tyrosine-derived pre-macrocycle (*S*)-**1a** was synthesized (96% *ee*, Figure S40) and reacted under pseudo high-dilution active template^22^ Cu-mediated alkyne–azide cycloaddition^23^ (AT-CuAAC) conditions^24^ with bipyridine macrocycle **2**.^25^ Catenane **3a** was produced with reasonable stereoselectivity (Table 1, entry 1), based on ^1^H NMR analysis of the crude reaction product; proton H_*a*_ of the diastereomers of **3a** resonate at 8.98 (major) and 9.07 (minor) ppm, respectively (Figure S111).^26^^1^H NMR analysis also suggested the presence of several other interlocked species, characterized by higher ppm (9.51–9.61; Figure S286) triazole resonances. LCMS analysis indicated that these signals were due to [3]catenane **4** (Scheme 1), which can be formed as three diastereomers, and the corresponding [2]catenane (not shown, two diastereomers) containing a single bipyridine ring (Supporting Information (SI) section S10). We were unable to obtain pure samples of these compounds.^27^

**Table 1:** Effect of Reaction Conditions and Structure of **1** on the AT-CuAAC Synthesis of Topologically Chiral Catenanes **3**^a^

entry	R	*T* (°C)	*t* (h)	**2**:**3**:oligos^a^	*de*^a^	yield
1	Et	60	4	34:44:22	70%	n.d.
2	Et	60	8	47:37:16	62%	n.d.
3	Et	25	4	15:44:41	74%	39%
4	^*i*^Pr	25	4	14:30:56	82%	26%
5	^*t*^Bu	25	4	77:11:12	68%	n.d.^b^

a Determined by ^1^H NMR analysis of the crude reaction product (SI section S10).

b Not isolated due to low conversion of **2**.

**Scheme 1:**
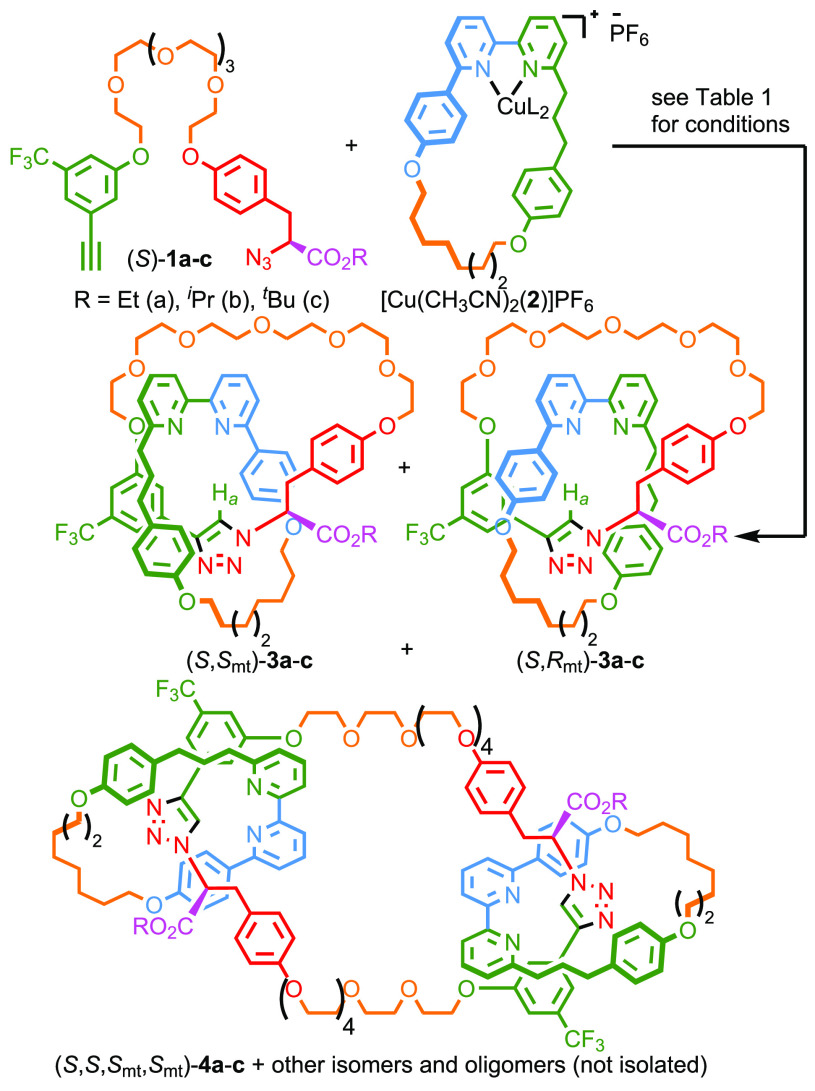
Synthesis of Topologically Chiral Catenanes **3** Reagents and conditions: (*S*)-**1** in CHCl_3_–EtOH (1:1, 10 mM) was added to [Cu(CH_3_CN)_2_(**2**)]PF_6_ (1 equiv, 24 mM), ^*i*^Pr_2_NEt (2 equiv) in CHCl_3_–EtOH (1:1). For full conditions, see Table 1.

Longer addition times (entry 2) resulted in diminished diastereoselectivity, perhaps due to epimerization of the covalent stereogenic center, and lower conversion of macrocycle **2**. Lowering the reaction temperature resulted in enhanced diastereoselectivity (74% *de*) and reduced quantities of oligomeric species, allowing catenane **3b** to be isolated in 39% yield and 74% *de* (entry 3). Although increasing the equivalents of **1a** resulted in higher conversion of **2**, lower yields of **3a** were obtained as the non-interlocked triazole-containing macrocycle was challenging to remove. Varying the solvent did not improve diastereoselectivity or conversion of **2** (SI section S8). Applying the same conditions to (*S*)-**1b**, which features a bulkier ^*i*^Pr ester, gave catenane **3b** in 82% *de*, albeit the conversion of macrocycle **2** was diminished and the formation of oligomeric biproducts was increased, resulting in a low isolated yield (26%, 82% *de*, entry 4). Surprisingly, (*S*)-**1c** gave poor stereoselectivity (68% *de*, entry 5) and low conversion of **2** (∼25%). Pleasingly, single crystal X-ray diffraction (SCXRD) analysis of a racemic sample of catenane **3b** produced using *rac*-**1b** allowed the relative stereochemistry of the major diastereomer to be tentatively assigned as (S*,*S*_mt_*). Thus, the major product of (*S*)-**1b** and macrocycle **2** is assigned as (*S*,*S*_mt_)-**3b** (Figure 2a).^28^

**Figure 2:**
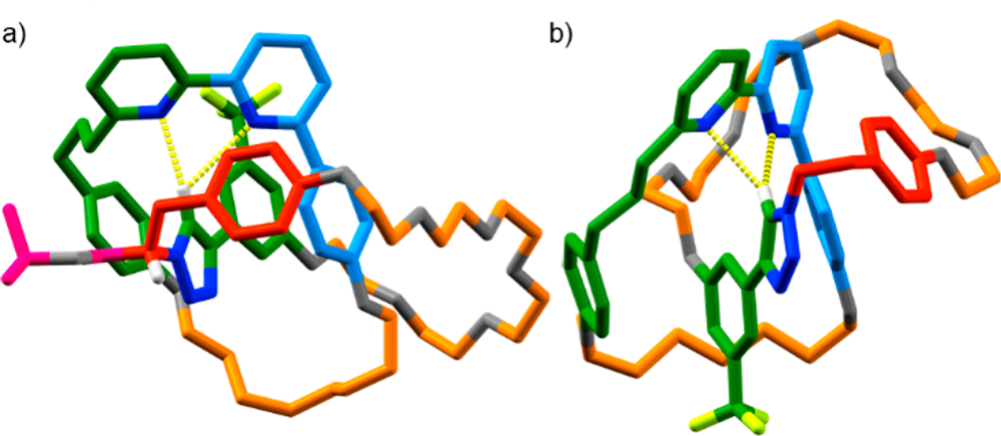
Solid state structures of (a) *rac*-(*S*,*S*_mt_)**-3b** and (b) *rac***-6**. Colors as in Scheme 1 except F (yellow), O (gray), N (dark blue), H (white). Majority of H atoms omitted for clarity. Selected intercomponent interactions highlighted (yellow).

We then turned to methods to remove the covalent stereogenic unit from the mixture of catenane **3b** diastereomers (Scheme 2). Attempts to ablate the covalent stereocenter of a model compound by radical decarboxylation met with failure due to scission of the triazole N^1^–C substituent bond (SI section S9). Ultimately, we found that reduction of ester **3b** to give alcohol catenane **5** followed by tandem Oppenauer-type oxidation/Rh^I^-mediated decarbonylation^29^ gave rise to catenane **6** in reasonable isolated yield (32% over two steps). CSP-HPLC analysis confirmed that the diastereoenriched starting material (82% *de*) was converted with good fidelity to enantioenriched (82% *ee*) catenane **6**. The major stereoisomer of **6** was assigned as (*S*_mt_) based on the assigned stereochemistry of the major diastereomer of **3b**. Crystals of a *rac*-**6** suitable for SCXRD analysis were obtained, allowing the structure of the product to be confirmed (Figure 2b).

**Scheme 2:**
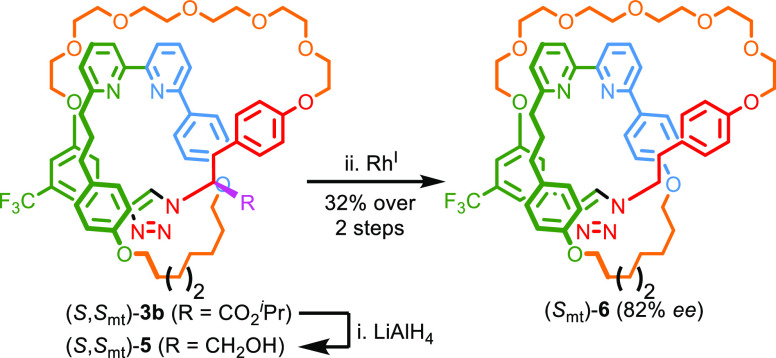
Decarbonylation of Catenane **3b** Reagents and conditions: i. LiAlH_4_, THF, −30 °C, 1 h; ii. [Rh(cod)Cl]_2_, [IrCp*Cl_2_]_2_, benzophenone, *rac*-BINAP, K_2_CO_3_, mesitylene, 170 °C, 5 h.

Finally, we turned to the synthesis of a co-conformationally “topologically” chiral target (Scheme 3). Pre-macrocycle (*S*)-**7** was subjected to the AT-CuAAC reaction with macrocycle **2**. The product, topologically chiral [2]catenane **8**, was isolated as a mixture of diastereomers (88% *de*), as judged by ^1^H NMR (Figure 3ai). By analogy with catenane **3b**, which seems reasonable given the similarities of the functional groups reacting and the similar stereoselectivity obtained, the major isomer is tentatively assigned as (*S*,*S*_mt_)-**8**.

**Scheme 3:**
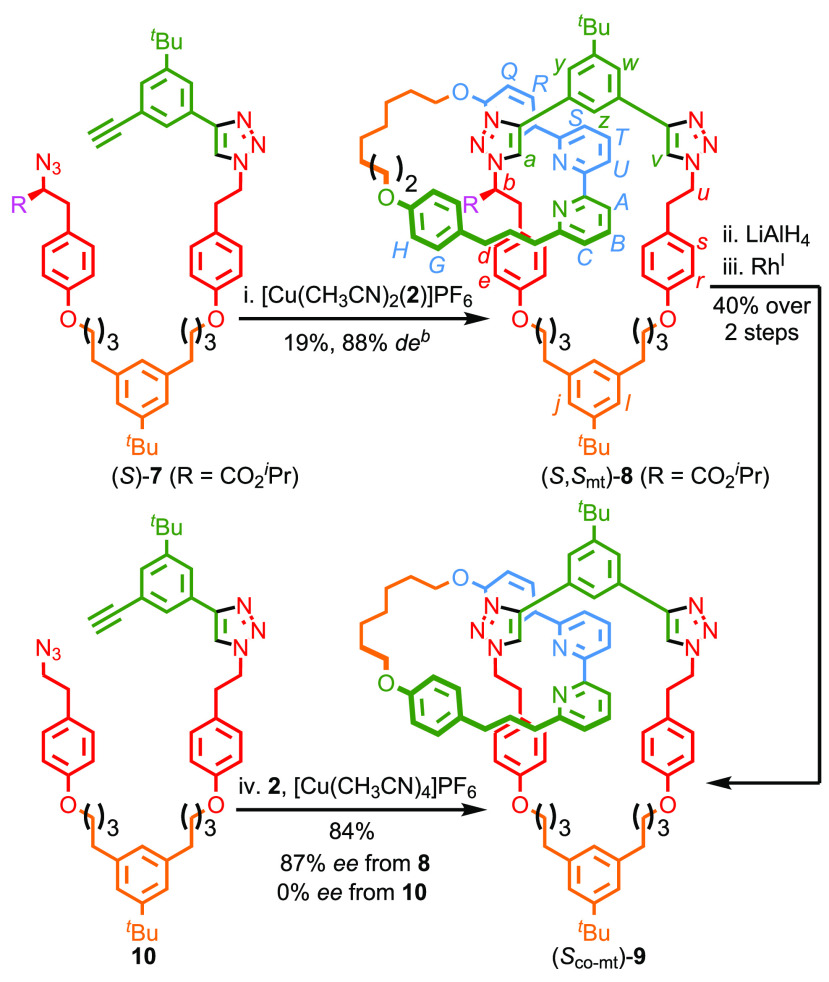
Synthesis of Co-conformationally “Topologically” Chiral Catenane **9** Reagents and conditions: i. (*S*)-**7** in CHCl_3_-EtOH (1:1) added to [Cu(CH_3_CN)_2_(**2**)]PF_6_ (1 equiv), ^*i*^Pr_2_NEt (2 equiv) in CHCl_3_-EtOH (1:1) over 4 h at 60 °C; ii. LiAlH_4_, THF, −30 °C, 1 h; iii. [Rh(cod)Cl]_2_, [IrCp*Cl_2_]_2_, benzophenone, *rac*-BINAP, K_2_CO_3_, mesitylene, 170 °C, 5 h; (iv) **10** in CHCl_3_-EtOH (1:1) added to **2** (1 equiv), [Cu(CH_3_CN)_4_]PF_6_ (0.96 equiv), ^*i*^Pr_2_NEt (2 equiv) in CHCl_3_-EtOH (1:1) over 4 h at 60 °C. Determined by ^1^H NMR.

**Figure 3:**
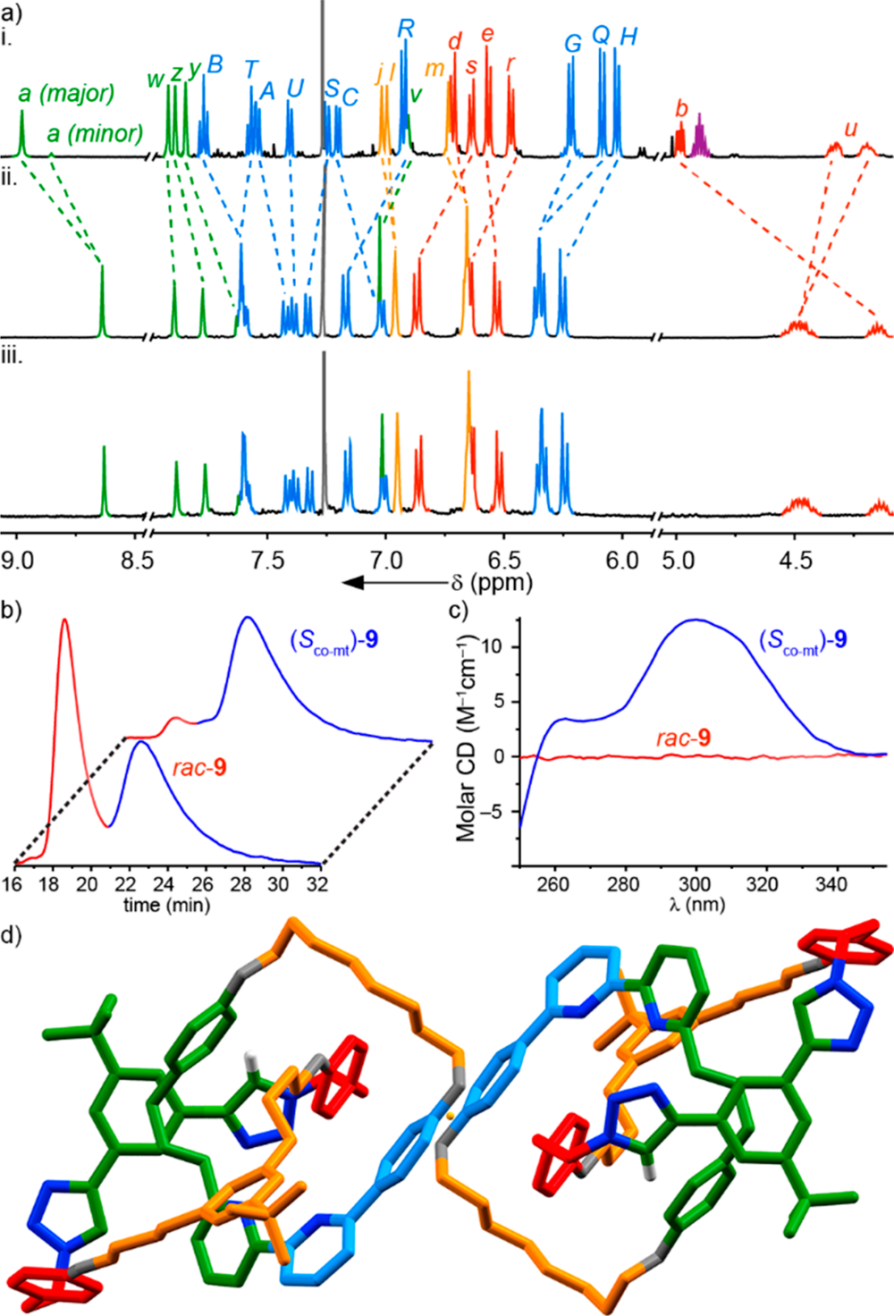
(a) Partial ^1^H NMR (CDCl_3_, 298 K) of i. catenane **8**, ii. catenane *rac*-**9**, and iii. enantioenriched catenane (*S*_co-mt_)-**9**. Atom labels and colors as in Scheme 3, except macrocycle signals (blue). (b) HPLC analysis of catenane *rac*-**9** and (*S*_co-mt_)-**9**. (c) Circular dichroism spectra of catenane *rac*-**9** and (*S*_co-mt_)-**9**. (d) Solid state structure of *rac*-**9** showing a pair of enantiomeric structures related by a point of inversion (orange). Colors as in Scheme 3 except O (gray), N (dark blue), H (white). Majority H atoms omitted for clarity.

Auxiliary removal from (*S*,*S*_mt_)-**8** (88% *de*) yielded [2]catenane **9**, which contains no previously described stereogenic units—it lacks covalent stereogenic units, and the triazole containing ring is not oriented and so the system does not conform to the definition of a topologically chiral catenane. Nevertheless, whereas the compounds produced from **10** and (*S*,*S*_mt_)-**8** produce identical ^1^H NMR spectra (Figure 3aii and 3aiii respectively), the latter is clearly highly enantioenriched, whereas the former is racemic as judged by CSP-HPLC analysis (Figure 3b), which indicates that catenane **9** was formed from (*S*,*S*_mt_)-**8** in 87% *ee*,^30^ and circular dichroism spectroscopy (Figure 3c). SCXRD of a sample of *rac*-**9** confirmed the structure of the product.^31^ As expected, both enantiomeric co-conformations were observed in the unit cell (Figure 3d). We tentatively assign the product of (*S*,*S*_mt_)-**8** to be (*S*_co-mt_)-**9**, as the relative arrangements of the rings cannot change during auxiliary removal.

In conclusion, we have developed an auxiliary for the synthesis of topologically chiral catenanes in high enantiopurity and applied it to the synthesis of catenane (*S*_co-mt_)-**9**, a molecule containing a previously unreported co-conformationally “topologically” chiral stereogenic unit, unambiguously demonstrating the chiral nature of this overlooked form of mechanical stereochemistry. However, it poses a problem of nomenclature—how can the topological stereochemistry of a molecule depend on its co-conformation? In short, it cannot,^1^ but once the fixed co-conformation is considered, the covalent subcomponents of catenane **9** display the same symmetry properties as those that comprise the established stereogenic unit of topologically chiral catenanes, which leads to our linguistic conundrum. One solution to this would be to rename “topologically chiral” catenanes as “mechanically planar chiral”, to bring them in line with the analogous rotaxanes to which they are strongly related, but this would require further discussion in the field. Linguistic issues aside, chiral interlocked molecules are attracting increasing attention for applications in catalysis,^32,33^ sensing,^34^ and as chiroptical^35^ or stereodynamic switches.^15b,16e^ By highlighting their potential to display molecular chirality due to unexplored stereogenic units, we hope to inspire further investigation of their rich stereochemistry.^36^

## Supporting Information Available

Procedures and full characterization data (NMR, MS, CD, SCXRD, HPLC as appropriate) for all novel compounds and supplementary discussion. (PDF)

## Notes

Data (characterization data for reported compounds) is available from the University of Southampton data repository (https://doi.org/10.5258/SOTON/D2279).
