# Supplementary material for: A Co-conformationally “Topologically” Chiral Catenane
Source: J Am Chem Soc. 2022 Jun 28;144(27):11927–32. doi: 10.1021/jacs.2c02029 (PMC9348828; doi:10.1021/jacs.2c02029)
Supplement: Supplementary file 1 — ja2c02029_si_001.pdf [file ja2c02029_si_001.pdf]

Electronic Supporting Information

A Co-conformationally “Topologically” Chiral Catenane

Arnau Rodríguez-Rubio, Andrea Savoini, Florian Modicom, Patrick Butler and Stephen M. Goldup

Department of Chemistry, University of Southampton, Highfield, Southampton, SO17 1BJ

*s.goldup@soton.ac.uk

Table of Contents

S1. General experimental......4

S2. Synthesis of Catenanes 3 (Scheme 1, main text)......6

Synthesis of catenane precursors (S)-1a-c......6

Macrocycle precursor (*S*)-1a......26
Macrocycle precursor (*S*)-1b......45
Macrocycle precursor (*S*)-1c......64
$[Cu(CH_3CN)_2(2)]PF_6$......69

Synthesis of catenanes 3......74

Catenane (*S*,S_{mt})-3a......74
Catenane (*S*,S_{mt})-3b......79
Catenane (*S*,S_{mt})-3c......89

S3. Synthesis of Catenane 6 (Scheme 2, main text)......90

Synthesis of *rac*-6 precursor S13......90

Macrocycle precursor S13......102

Synthesis of catenanes *rac*-6 and (S_{mt})-6......106

Catenane *rac*-6......106
Catenane (S_{mt})-6......112

S4. Synthesis of Catenane 9 (Scheme 3, main text)......115

Synthesis of catenane precursors (*S*)-7 and 10......115

Macrocycle precursor (*S*)-7......142
Macrocycle precursor 10......146

Synthesis of catenane 8......150

Catenane (*S*,S_{mt})-8......150

Synthesis of *rac*-9 and ($S_{co\text{-}mt}$)-9......159

Catenane *rac*-9......159
Catenane ($S_{co\text{-}mt}$)-9......165

S5. Model compounds for auxiliary cleavage study......168

S6. Assignment of Stereochemistry for Catenanes 3, 6, 4, S35, 8 and 9......177

Proposed method for assigning stereochemistry in topologically chiral catenanes......177

Stereochemical assignment of catenanes 3......177

Stereochemical assignment of [2]catenanes S35......177

Stereochemical assignment of [2]catenanes 4......178

Stereochemical assignment of catenane 6......179

Stereochemical assignment of catenane 8......179

Stereochemical assignment of catenane 9......179

S7. Crystallographic data......181

Single Crystal X-ray Diffraction Data for Catenane *rac*-(*S*,S_{mt})-3b......181

Single Crystal X-ray Diffraction Data for Catenane *rac*-6......182

Single Crystal X-ray Diffraction Data for Catenane *rac-9*......183

S8. Optimisation of conditions for the synthesis of catenanes 3......184

S9. Optimisation of Methods to Remove the Chiral Auxiliary 186
Radical Decarboxylation Reactions 186
Rh^I-Mediated Decarbonylation Reactions 186
S10. Identification of By-products S35 and 4.. 188
S11. Co-conformational equilibrium of catenanes 3a and 3b 193
S12. References 198

S1. GENERAL EXPERIMENTAL

Experimental procedures: Unless otherwise stated, all reagents were purchased from commercial sources (Acros Organics, Alfa Aesar, Fisher Scientific, FluoroChem, Sigma Aldrich and VWR) and used without purification. $[Cu(CH_3CN)_4]PF_6$ was prepared as described by Pigorsch and Köckerling.[1] Anhydrous solvents were purchased from Acros Organics. Petrol refers to the fraction of petroleum ether boiling in the range 40-60 °C. DMAP refers to 4-(dimethylamino)pyridine. ADMP refers to 2-Azido-1,3-dimethylimidazolinium hexafluorophosphate. TMSA refers to trimethylsilylacetylene. IPA refers to isopropanol. THF refers to tetrahydrofuran. TFA refers to trifluoroacetic acid. TBAF refers to tetrabutylammonium fluoride. EDTA-NH_3 solution refers to an aqueous solution of NH_3 (17% w/w) saturated with sodium-ethylenediaminetetraacetate. $CDCl_3$ (unstabilized) was distilled over $CaCl_2$ and K_2CO_3 prior to use. Unless otherwise stated, all reactions were performed in oven dried glassware under an inert N_2 atmosphere with purchased anhydrous solvents. Unless otherwise stated, experiments carried out in sealed vessels were performed in CEM microwave vials, with crimped aluminium caps, with PTFE septa. Unless otherwise stated, flash column chromatography was performed using Biotage Isolera-4 or Isolera-1 automated chromatography systems using SiO_2 cartridges purchased from Biotage (Sfär, 60 µm, SNAP or ZIP, 50 µm irregular silica; default flow rates). Deactivated SiO_2 refers to cartridges which were eluted with petrol-NEt_3 (90 : 10, 2 column volumes), followed by petrol (2 column volumes). Analytical TLC was performed on pre-coated almunium-backed silica gel plates (0.25 mm thick, 60F254, Merck, Germany) and observed under UV light (254 nm) or visualised with $KMnO_4$. All melting points were determined using a Griffin apparatus.

NMR analysis: Spectra were recorded on Bruker AV400 or AV500 instrument, at 298 K. Chemical shifts are reported in parts per million from low to high field and referenced to residual solvent (^1H, ^{13}C), 85% H_3PO_4 (^{31}P) or neat $CFCl_3$ (^{19}F). Coupling constants (J) are reported in Hertz (Hz). Standard abbreviations indicating multiplicity were used as follows: m = multiplet, quint = quintet, q = quartet, t = triplet, d = doublet, s = singlet, br = broad, sept = septet. Where the observed signal multiplicity differs from that expected, app. (apparent) precedes the indication of multiplicity. ^1H signal assignment was carried out using 2D NMR methods (COSY, NOESY, ROESY, HSQC, HMBC) as appropriate. In interlocked compounds, proton signals corresponding to triazole-containing macrocycle are in lower case, and those corresponding to the bipyridine macrocycle are in upper case. In cases of complex multiplets with multiple contributing proton signals, exact assignment was not possible. Where ^{13}C signals are coincident, the number of contributing resonances (identified by HMBC/HSQC) is indicated (e.g. 123.1 (×2)). Some ^{13}C signals of compounds **3** and their precursors are split into quartets due to coupling to the three equivalent ^{19}F of the CF_3 group. Although all required ^{13}C environments were identified unambiguously throughout, in some cases, not all lines of these quartets were observed because: i) some signals were obscured by other resonances; ii) the outlying signals were of low intensity (i.e. they appear as an apparent doublet); the split signals are poorly resolved due to line broadening even at high field. Where this occurs, it is clearly indicated in the ^{13}C peak listing. An inset is also included in the corresponding graphical ^{13}C data to highlight the ^{19}F-coupled signals.

MS analysis: Low resolution mass spectrometry was carried out using a Waters TQD mass spectrometer equipped with a triple quadrupole analyser with UHPLC injection (Waters BEH C18 column; CH_3CN-H_2O gradient [0.2% formic acid]). High resolution mass spectrometry was carried out using a Bruker Daltronics MaXis sprectrometer with time-of-flight analyser with UHPLC injection (Waters BEH C18 column; CH_3CN-H_2O gradient [0.2% formic acid]. Isotopic patterns are reported for compounds with *m/z* > 1000 in place of HRMS data, which is not meaningful for compounds of this size.

Chiroptical analysis: Chiral compounds were analysed by circular dichroism (CD) after desiccation under vacuum overnight (Applied Photophysics Chirascan spectropolarimeter, software Ver. 4.2.0; spec. grade $CHCl_3$; 1 cm pathlength, 293 K). CD data is reported for catenanes **3b** and **8** as a mixture of diastereomers so the interested reader can compare their data with those of catenanes **6** and **9** respectively. UV-vis data is given for compounds whose CD response is noisy to indicate where they absorb. In these cases, optical rotation is reported as an alternative chiroptical technique (Optical Activity PolAAr 2001, spec. grade $CHCl_3$; 20 cm pathlength, c in g / 100 mL, 296 K).

Chiral stationary phase HPLC (CSP-HPLC): Analysis was performed using a Waters Acquity Arc Instrument at 303 K, with *n*-hexane-IPA or *n*-hexane-ethanol isocratic eluents and the following columns: Regis Technologies (*S*,*S*)-Whelk-O1 (1-(3,5-dinitrobenzamido)-1,2,3,4-tetrahydrophenanthrene stationary phase), RegisPack (tris-(3,5-dimethylphenyl) carbamoyl amylose stationary phase), RegisPackCLA-1 (tris-(5-chloro-2-methylphenyl) carbamoyl amylose stationary phase); RegisCell (tris-(3,5-dimethylphenyl) carbamoyl cellulose stationary phase) (5 micron, column dimensions 25 cm x 4.6 mm).

Single Crystal Xray Diffraction: Data was collected at 100 K using a Rigaku 007 HF diffractometer equipped with a HYPix6000 enhanced sensitivity detector. Cell determination, data collection, data reduction, cell refinement and absorption correction were performed with CrysAlisPro. Structures were solved using Olex2 with SHELXT dual methods and refined against F2 with SHELXL refinement package using anisotropic thermal displacement parameters for all non-hydrogen atoms. H atoms were placed in calculated position and refined using a riding model.

Compounds synthesised according to literature procedures: **2**,[2] **S2**,[3] **S4a-c**,[4] **S14**,[5] **S15**,[6] **S36**,[7] **S38**,[7] **S39**.[8]

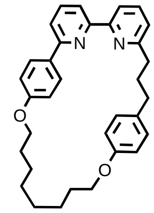

2

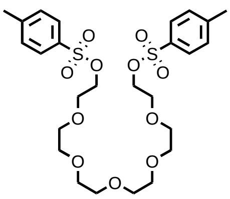

S2

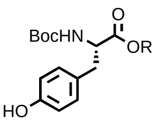

S4a-c

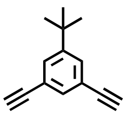

S14

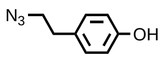

S15

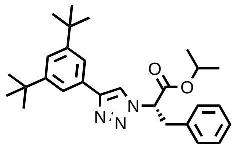

S36

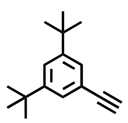

S38

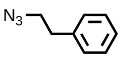

S39

S2. Synthesis of Catenanes 3 (Scheme 1, main text)

Synthesis of catenane precursors (S)-1a-c

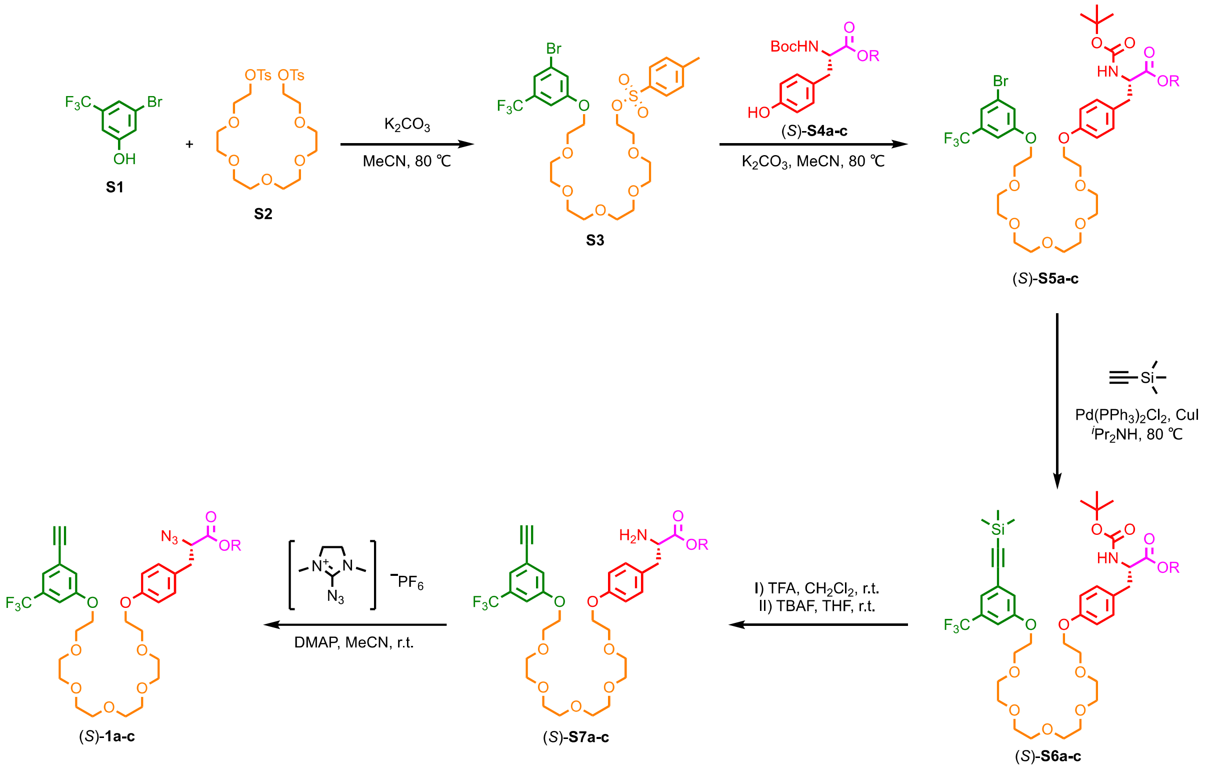

Scheme S1. Synthetic route to macrocycle precursors (*S*)-**1a-c**.

Di-tosylate S2

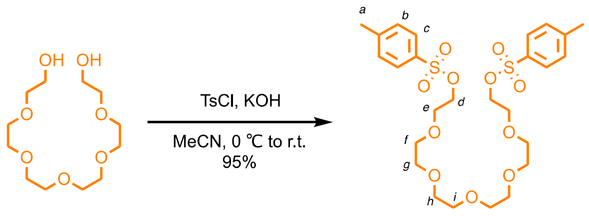

S2

Hexaethylene glycol (6.0 g, 21 mmol, 1.0 equiv.) and *p*-toluenesulfonyl chloride (8.4 g, 47 mmol, 2.2 equiv.) were dissolved in CH_2Cl_2 (20 mL). The solution was cooled at 0°C, then KOH (9.5 g, 17 mmol, 8.0 equiv.) was added portion wise. The suspension was stirred at rt for 16 h. The mixture was poured into H_2O (200 mL), the aqueous phase was extracted with CH_2Cl_2 (2 × 200 mL) and the collected organic phases were washed with brine (3 × 200 mL). The organic phase was dried ($MgSO_4$) and the solvent was removed under reduced pressure to obtain **S2** as a colourless oil (11 g, 95%). No further purification was required. All data are in accordance with previous reports.[3]

^{1}H NMR (400 MHz, $CDCl_3$, 298 K) δ: 7.79 (d, *J* = 7.6, 4H, H_b), 7.34 (d, *J* = 7.6, 4H, H_c), 4.17-4.13 (m, 4H, H_d), 3.70-3.66 (m, 4H, H_e), 3.64-3.53 (m, 16H, H_f, H_g, H_h, H_i), 2.44 (s, 6H, H_a).

^{13}C NMR (101 MHz, $CDCl_3$, 298 K) δ: 144.9, 133.1, 129.9, 128.1, 70.9, 70.7, 70.7, 70.6, 69.4, 68.8, 21.7.

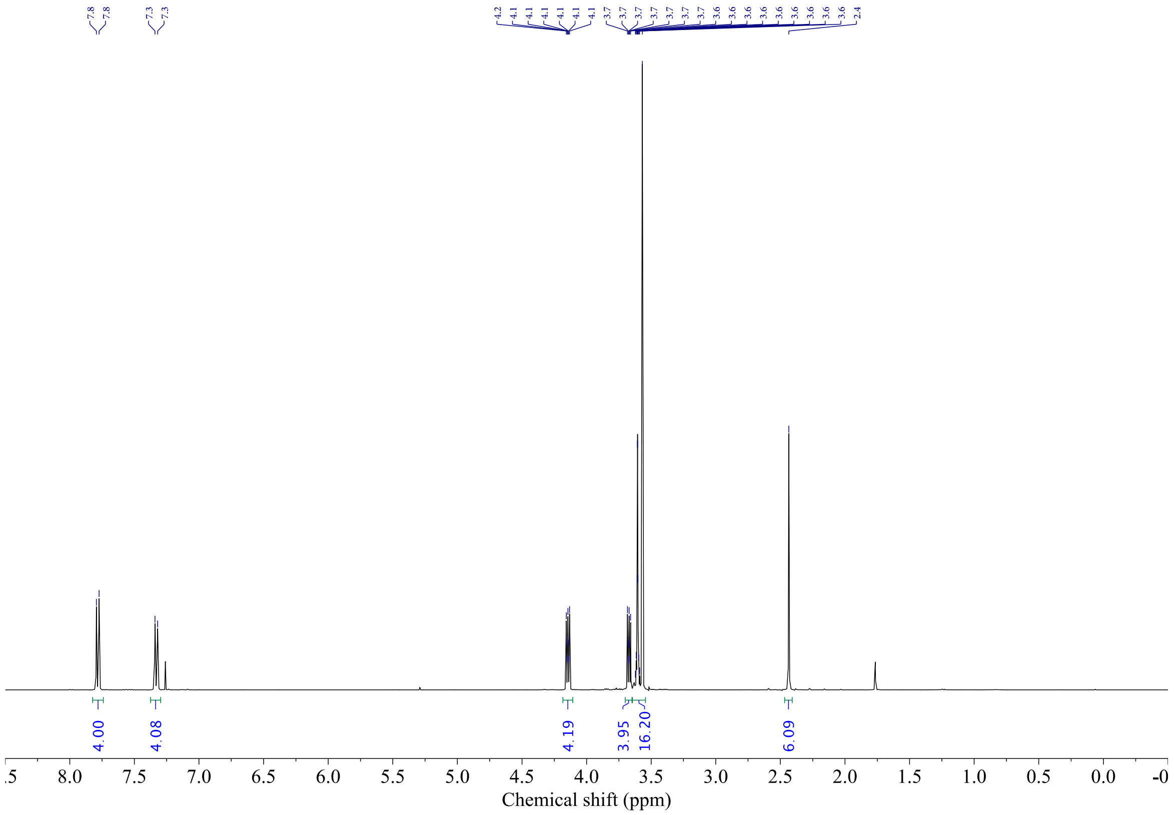

Figure S1. ^{1}H NMR of **S2** ($CDCl_3$, 400 MHz, 298 K)

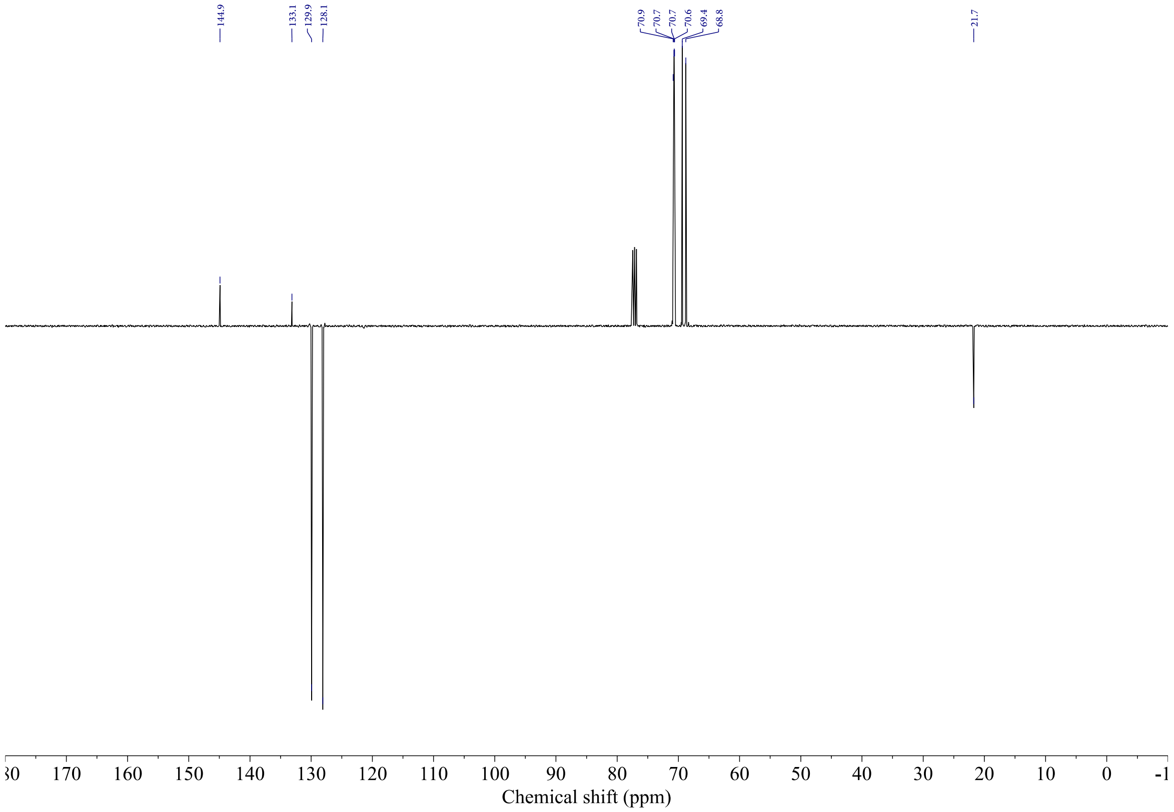

Figure S2. JMOD NMR of **S2** ($CDCl_3$, 101 MHz, 298 K)

Aryl ether S3

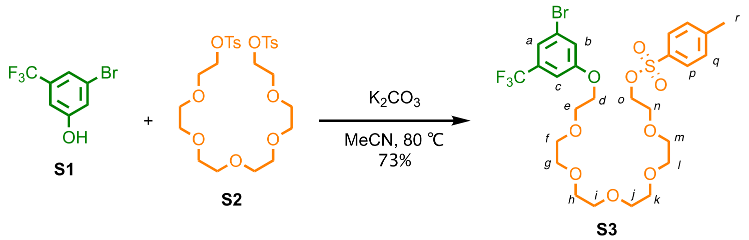

S1 (1.0 g, 4.1 mmol, 1.0 equiv.), **S2** (4.9 g, 8.3 mmol, 2.0 equiv.) and K_2CO_3 (2.3 g, 17 mmol, 4.0 equiv.) were suspended in CH_3CN (40 mL) and the resulting suspension was heated at reflux for 16 h. The reaction mixture was filtered over a Celite® pad, which was washed with CH_2Cl_2 (80 mL). The washings were combined, and the solvent was removed *in vacuo*. The crude was purified by column chromatography (petrol-Et_2O 50 : 50 → 0 : 100) to give **S3** as a clear colorless oil (1.99 g, 73%).

^{1}H NMR (400 MHz, $CDCl_3$, 298 K) δ: 7.79 (d, *J* = 8.3, 2H, H_p), 7.37-7.30 (m, 3H, H_q, H_a), 7.24 (t, *J* = 2.1, 1H, H_b), 7.09 (t, *J* = 1.9, 1H, H_c), 4.31-3.99 (m, 4H, H_d, H_o), 3.91-3.80 (m, 2H, H_e), 3.76-3.44 (m, 18H, H_f, H_g, H_h, H_i, H_j, H_k, H_l, H_m, H_n), 2.44 (s, 3H, H_r).

^{19}F NMR (376 MHz, $CDCl_3$, 298 K) δ: -63.16 (s, 3F, CF_3).

^{13}C NMR (101 MHz, $CDCl_3$, 298 K) δ: 159.8, 144.9, 133.1, 133.0 (q, $J_{C\text{-}F}$ = 33.0), 129.9, 128.0, 123.2, 123.1 (q, $J_{C\text{-}F}$ = 273.0), 121.3, 120.8 (q, $J_{C\text{-}F}$ = 3.9), 111.0 (q, $J_{C\text{-}F}$ = 3.8), 71.0, 70.8, 70.7, 70.7 (×2), 70.6, 70.6, 70.6, 69.5, 69.3, 68.7, 68.3, 21.7.

HR-ESI-MS *m/z* = 659.1142 $[M+H]^+$ calc. 659.1132 for $C_{26}H_{35}BrF_3O_9S$.

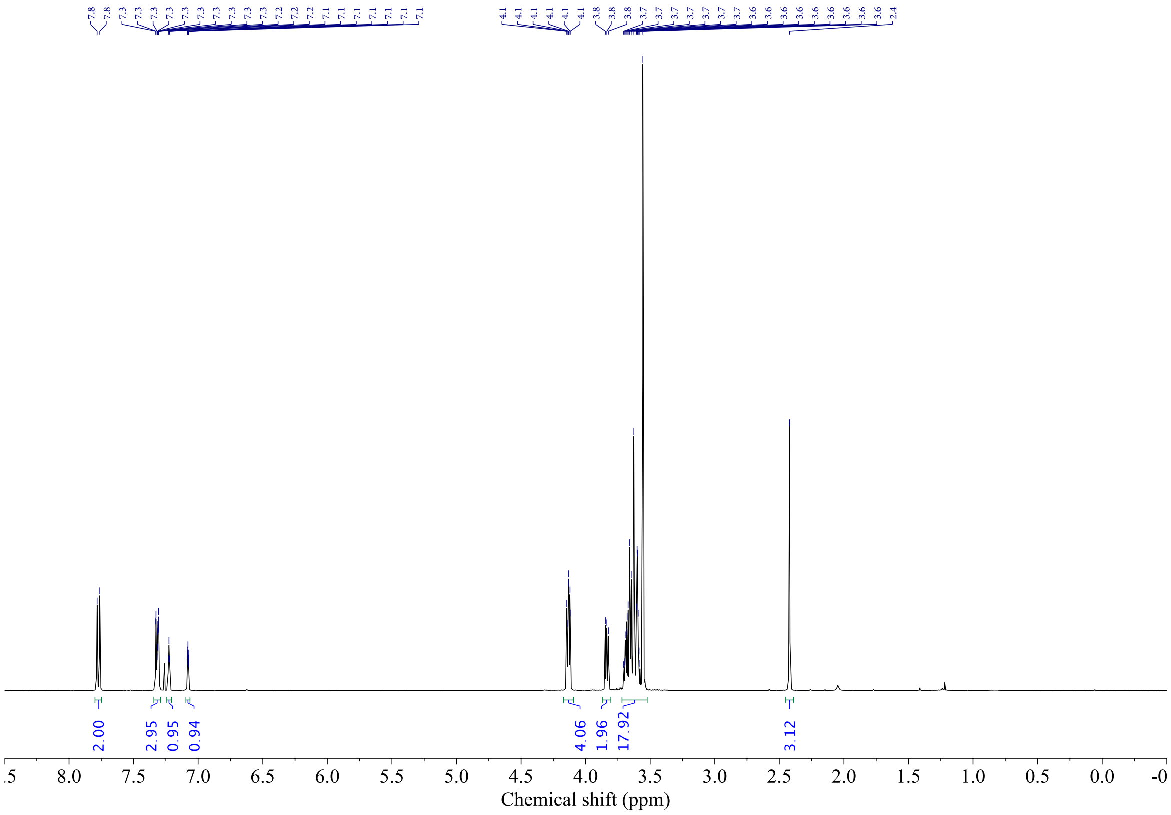

Figure S3. ^{1}H NMR of **S3** ($CDCl_3$, 400 MHz, 298 K)

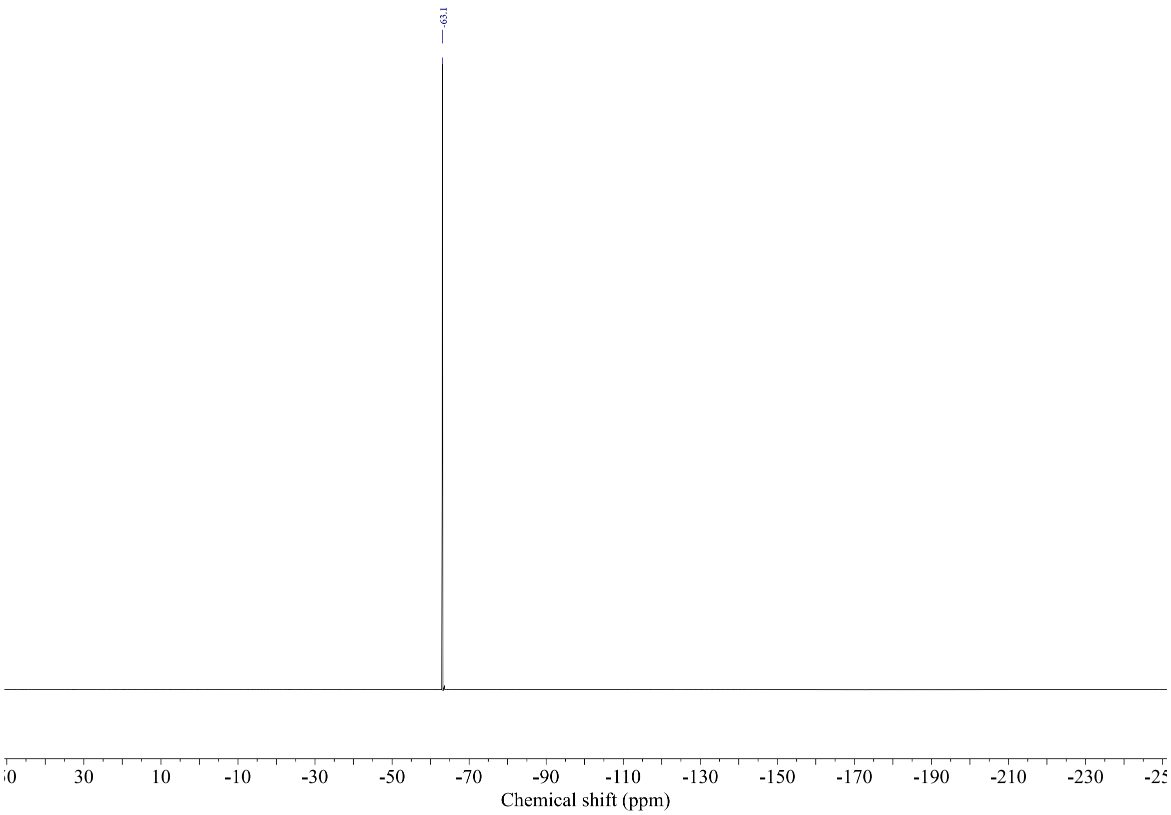

Figure S4. ^{19}F NMR of **S3** ($CDCl_3$, 376 MHz, 298 K)

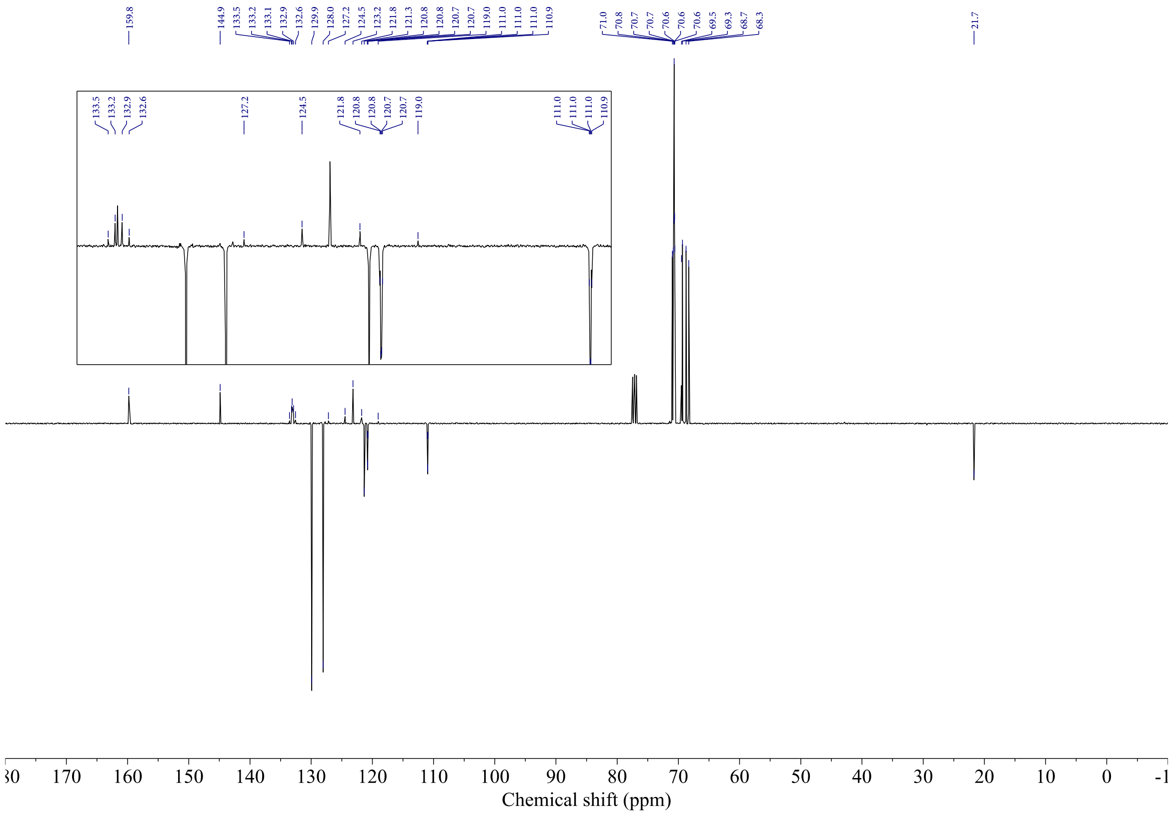

Figure S5. JMOD NMR of **S3** ($CDCl_3$, 101 MHz, 298 K)

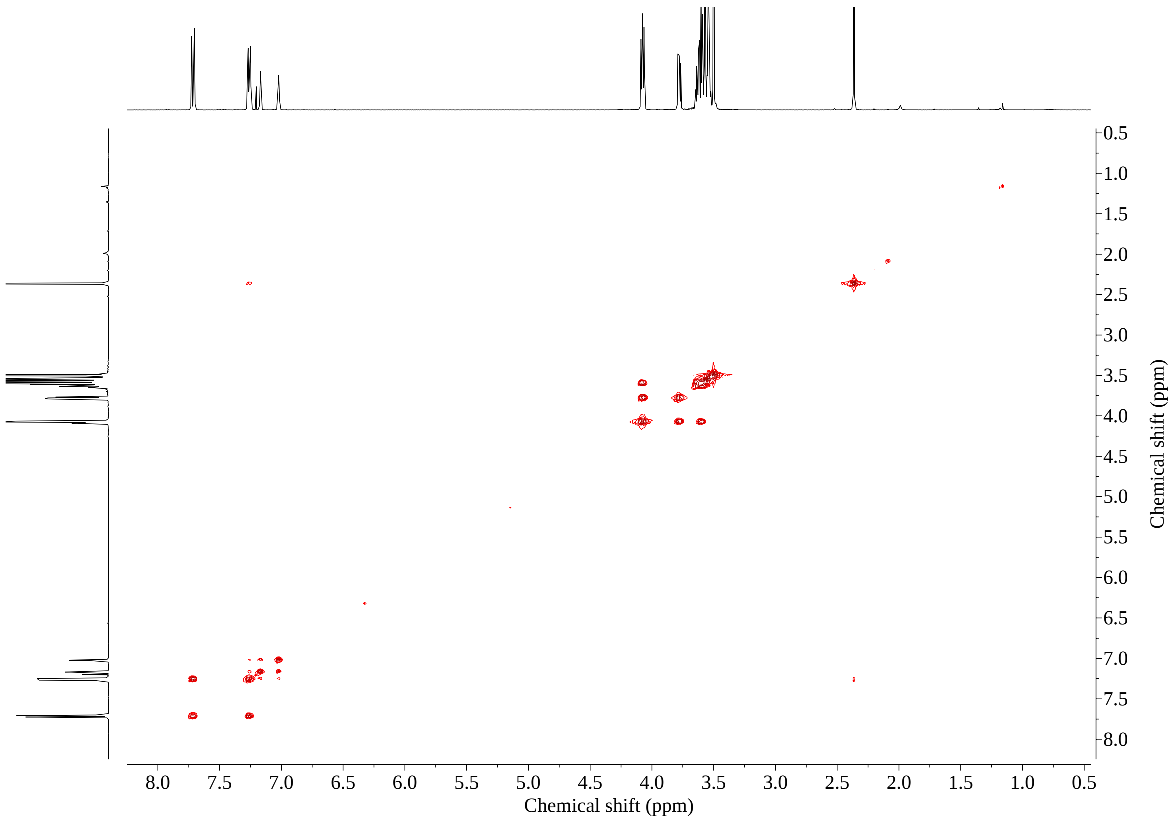

Figure S6. COSY NMR of **S3** ($CDCl_3$, 298 K)

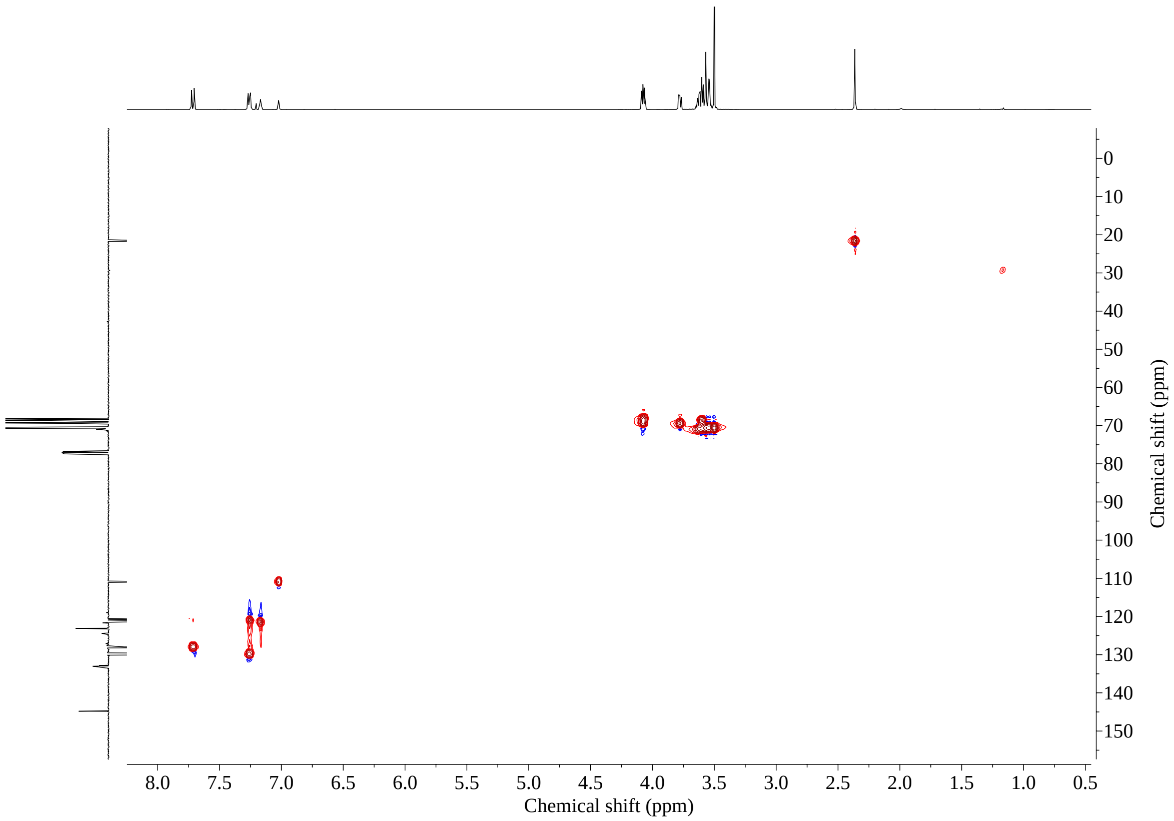

Figure S7. HSQC NMR of **S3** ($CDCl_3$, 298 K)

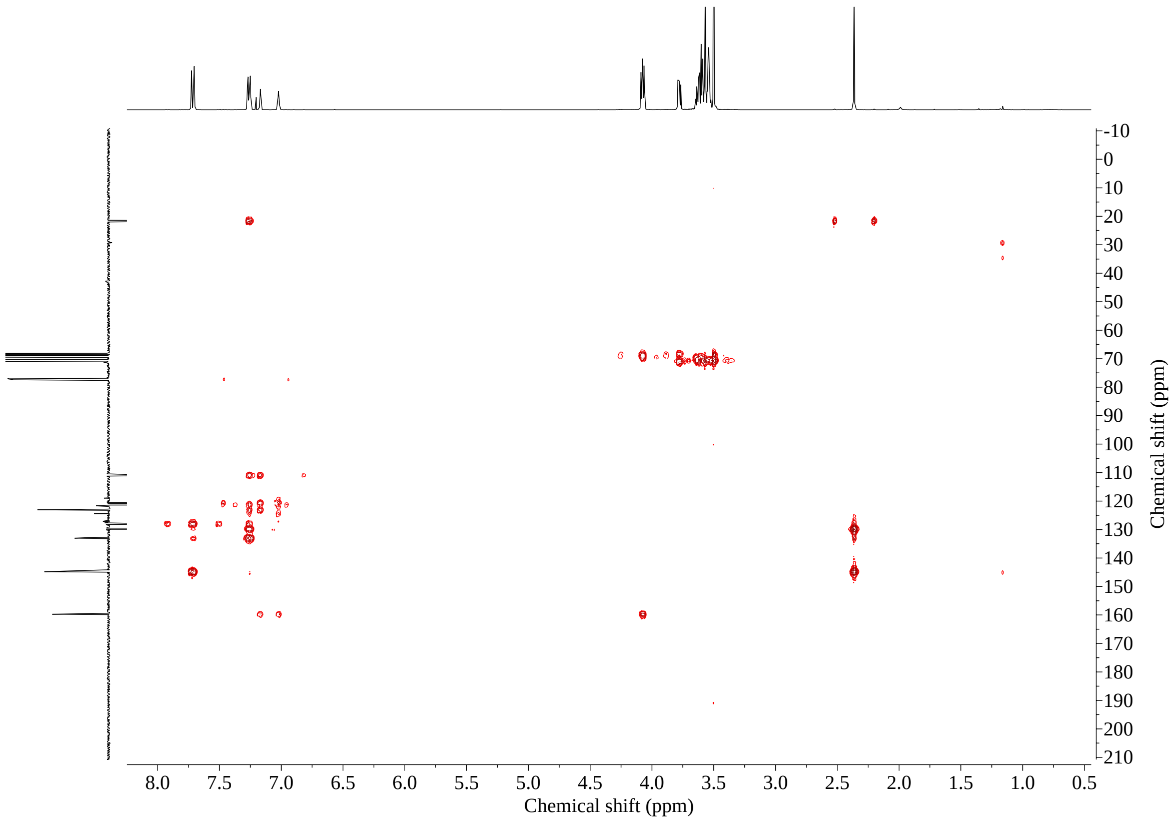

Figure S8. HMBC NMR of **S3** ($CDCl_3$, 298 K)

Boc-amine (*S*)-S5a

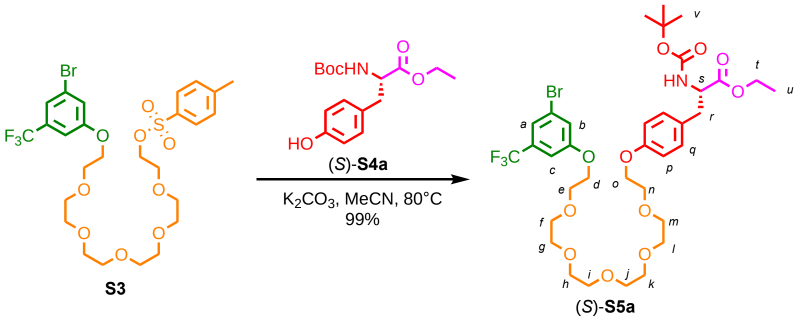

S3 (138 mg, 0.21 mmol, 1.0 equiv.), (*S*)-**S4a** (65 mg, 0.20 mmol, 1.0 equiv.) and K_2CO_3 (154 mg, 0.84 mmol, 4.0 equiv.) were suspended in CH_3CN (2 mL) and the resulting suspension was heated at reflux for 48 h. The reaction mixture was filtered over a Celite® pad, which was washed with CH_2Cl_2 (20 mL). The washings were combined, and the solvent was removed *in vacuo*. The crude was purified by column chromatography (petrol-EtOAc 75 : 25 → 25 : 75) to give (*S*)-**S5a** as a colorless oil (168 mg, 99%).

^{1}H NMR (400 MHz, $CDCl_3$, 298 K) δ 7.35-7.31 (m, 1H, H_a), 7.24 (t, J = 2.1, 1H, H_b), 7.12-7.07 (m, 1H, H_c), 7.03 (d, J = 8.6, 2H, H_q), 6.82 (d, J = 8.6, 2H, H_p), 4.95 (d, J = 8.3, 1H, NH), 4.59-4.44 (m, 1H, H_s), 4.20-4.10 (m, 4H, H_d, H_t), 4.12-4.04 (m, 2H, H_o), 3.88-3.79 (m, 4H, H_e, H_n), 3.74-3.56 (m, 16H, H_f, H_g, H_h, H_i, H_j, H_k, H_l, H_m), 3.09-2.93 (m, 2H, H_r), 1.42 (s, 9H, H_v), 1.23 (t, J = 7.1, 3H, H_u).

^{19}F NMR (376 MHz, $CDCl_3$, 298 K) δ: -63.17 (s, 3F, CF_3).

^{13}C NMR (101 MHz, $CDCl_3$, 298 K) δ: 172.0, 159.8, 158.0, 155.2, 133.1 (q, $J_{C\text{-}F}$ = 33.0), 130.4, 128.4, 123.2, 123.1 (q, $J_{C\text{-}F}$ = 272.9), 121.4, 120.8 (q, $J_{C\text{-}F}$ = 3.9), 114.7, 111.0 (q, $J_{C\text{-}F}$ = 3.8), 79.9, 71.0, 70.9, 70.8, 70.7 (×3), 70.7 (×2), 69.9, 69.5, 68.3, 67.5, 61.4, 54.7, 37.6, 28.4, 14.3.

HR-ESI-MS *m/z* = 813.2789 $[M+NH_4]^+$ calc. 813.2779 for $C_{35}H_{53}BrF_3N_2O_{11}$.

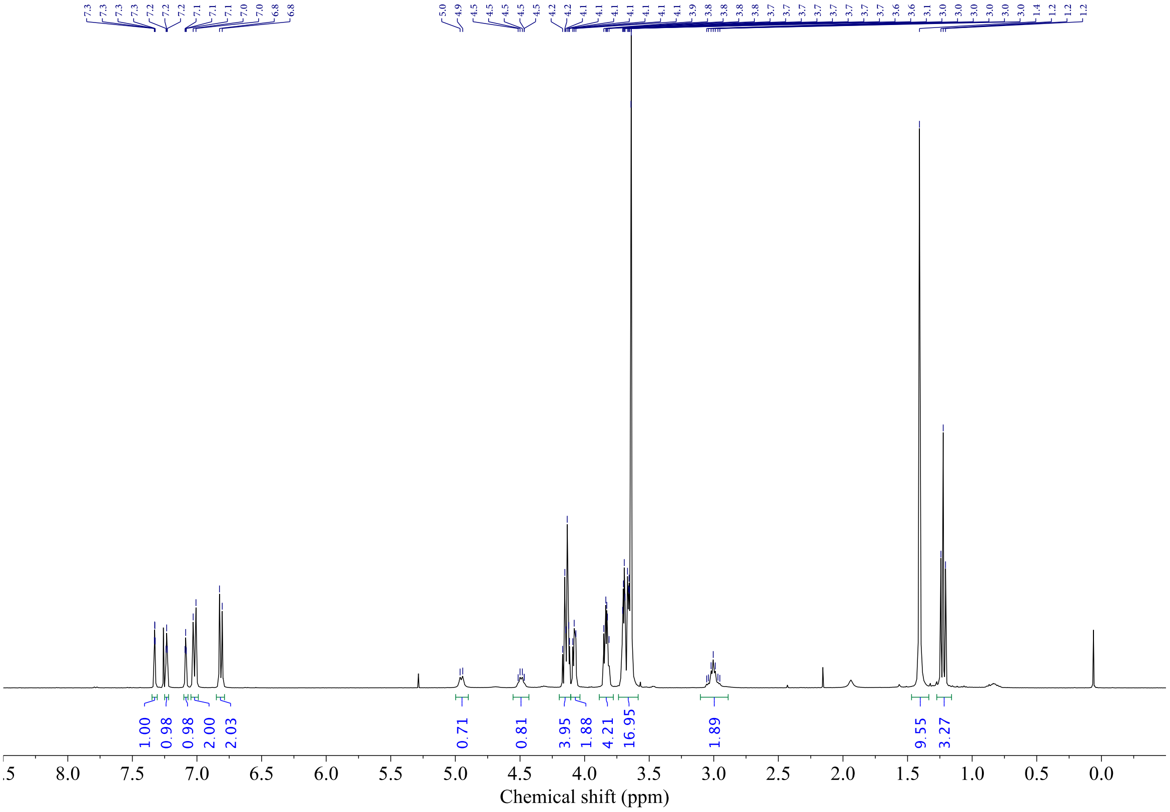

Figure S9. ^{1}H NMR of (*S*)-**S5a** ($CDCl_3$, 400 MHz, 298 K)

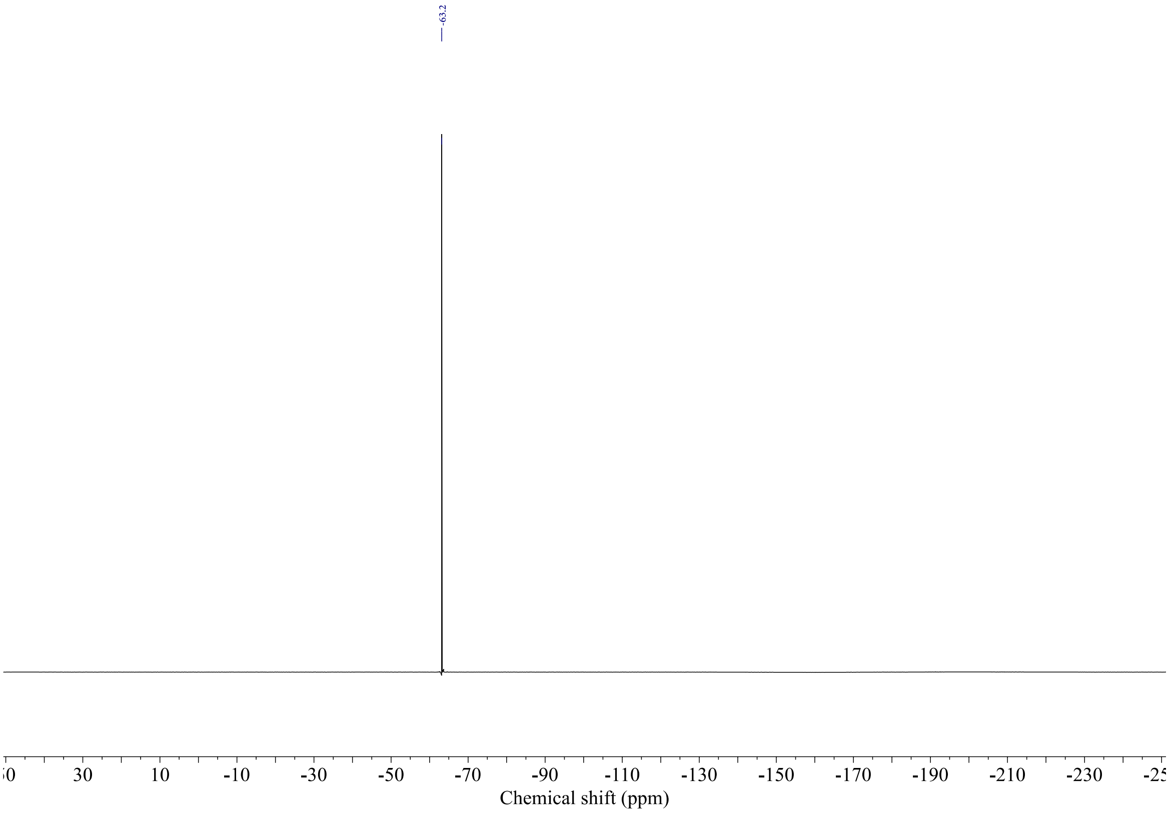

Figure S10. ^{19}F NMR of (*S*)-**S5a** ($CDCl_3$, 376 MHz, 298 K)

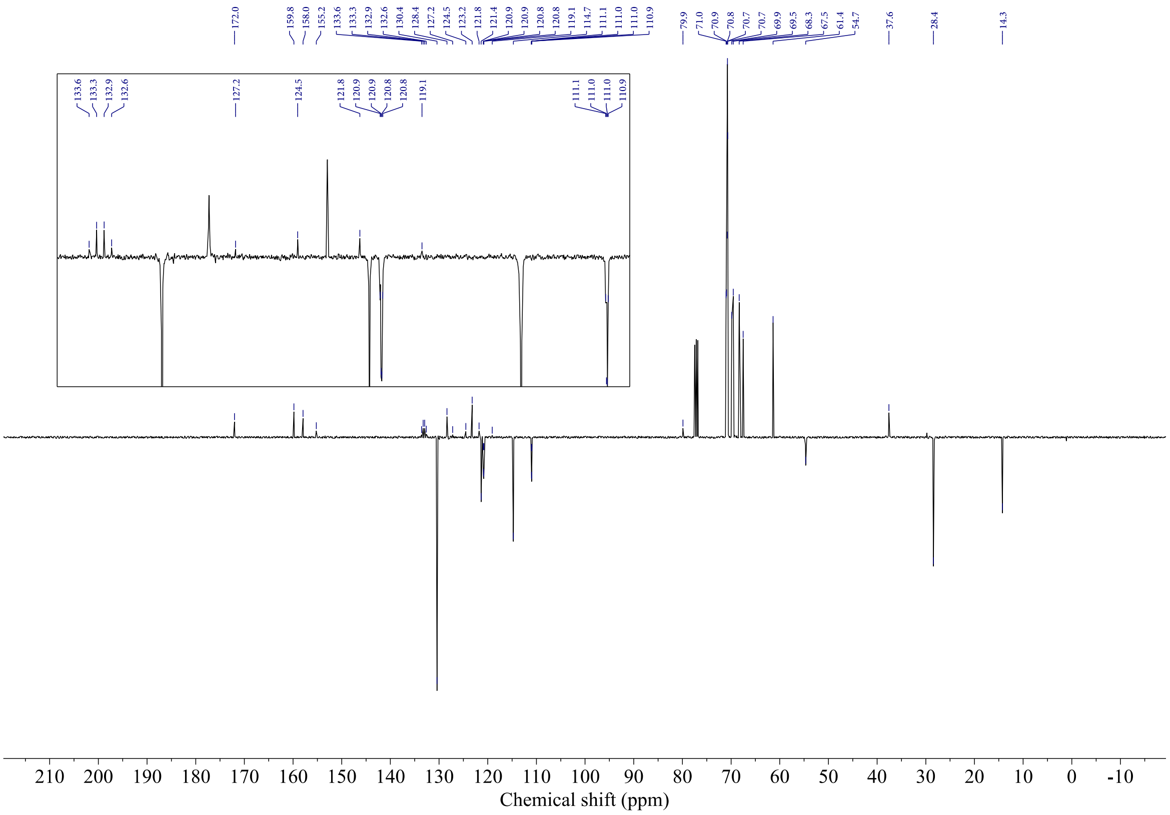

Figure S11. JMOD NMR of (*S*)-**S5a** ($CDCl_3$, 101 MHz, 298 K)

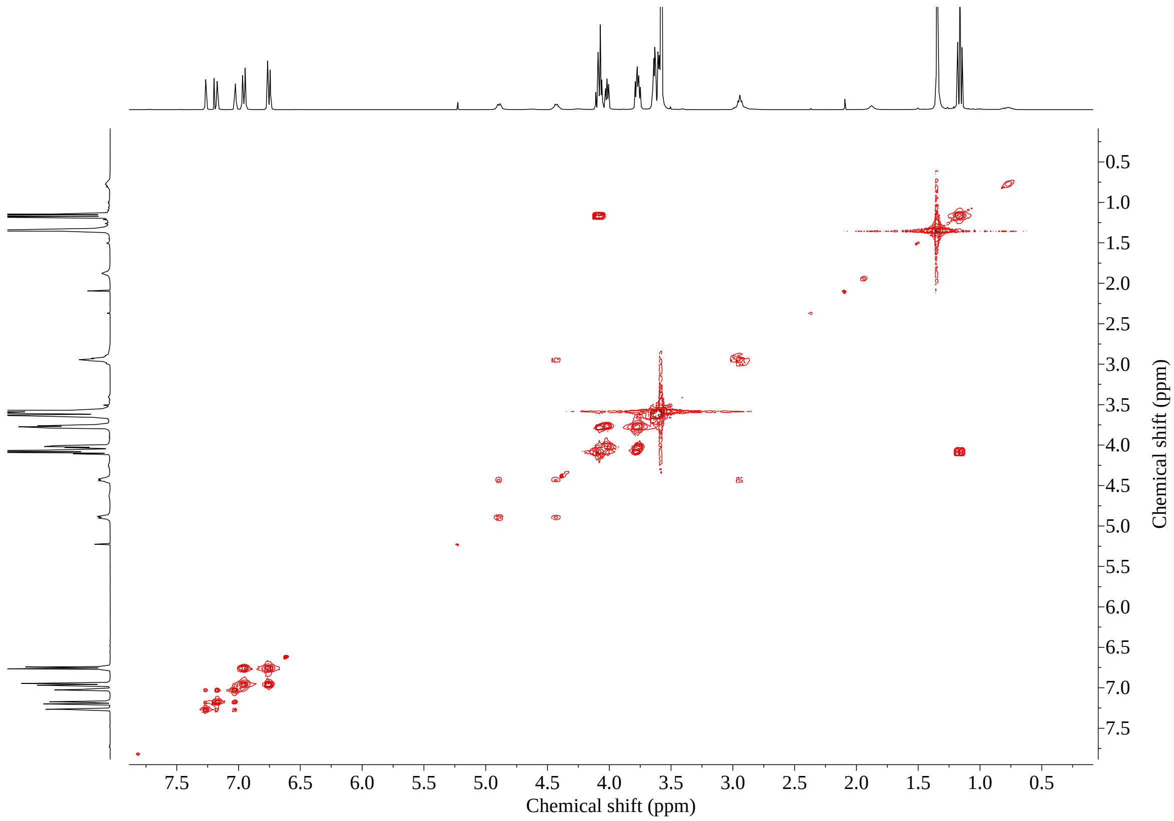

Figure S12. COSY NMR of (*S*)-**S5a** ($CDCl_3$, 298 K)

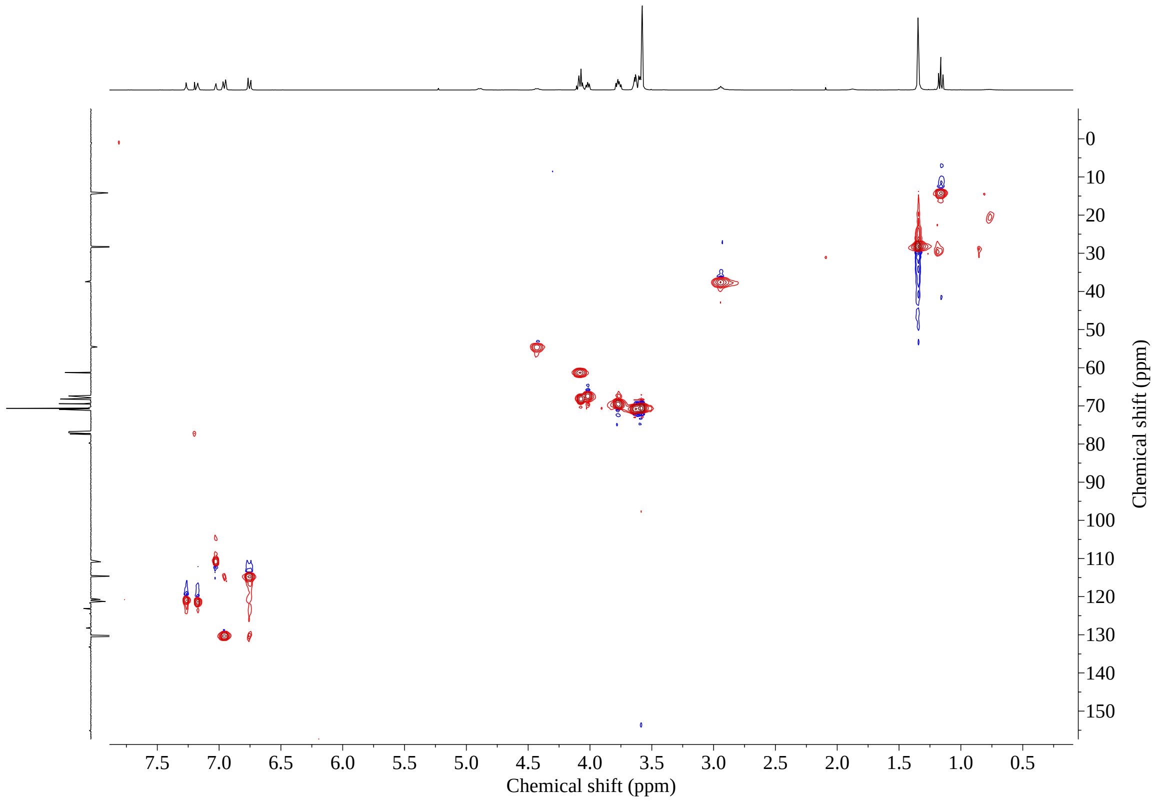

Figure S13. HSQC NMR of (*S*)-**S5a** ($CDCl_3$, 298 K)

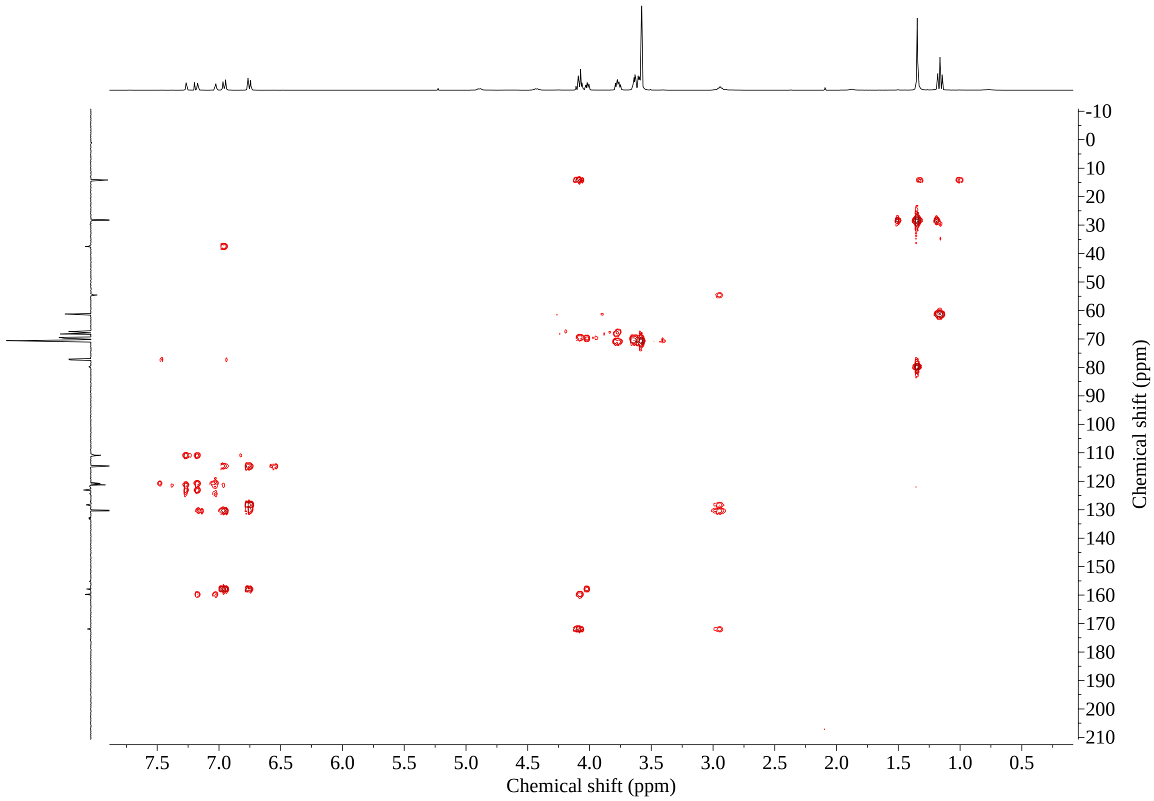

Figure S14. HMBC NMR of (*S*)-**S5a** ($CDCl_3$, 298 K)

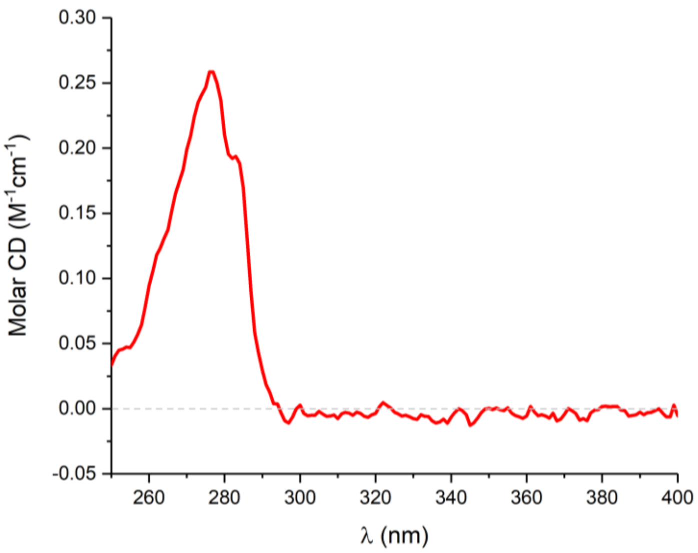

Figure S15. Circular Dichroism Spectra of (*S*)-**S5a** (251 μM) at 293 K in $CHCl_3$

TMS acetylene (*S*)-S6a

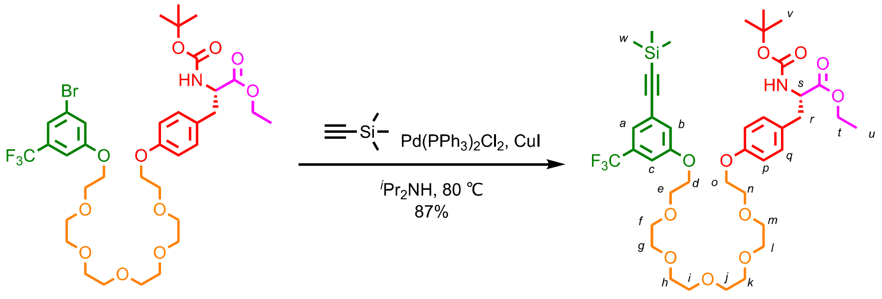

(*S*)-**S5a** (*S*)-**S6a**

(*S*)-**S5a** (147 mg, 0.18 mmol, 1.0 equiv.), $Pd(PPh_3)_2Cl_2$ (3 mg, 0.004 mmol, 0.02 equiv.) and CuI (2 mg, 0.008 mmol, 0.04 equiv.) were suspended in $^iPr_2NH$ (2 mL). The resulting suspension was degassed by bubbling N_2 through the solution over 5 min. Ethynyltrimethylsilane (40 μL, 0.28 mmol, 1.5 equiv.) was added and the mixture was stirred at 80 °C for 16 h. The solvent was removed *in vacuo* and the crude was purified by column chromatography (petrol-EtOAc 80 : 20 → 0 : 100) to give (*S*)-**S6a** as a brown oil (127 mg, 87%).

^{1}H NMR (400 MHz, $CDCl_3$, 298 K) δ: 7.32-7.29 (m, 1H, H_a), 7.16-7.13 (m, 1H, H_b), 7.12-7.09 (m, 1H, H_c), 7.03 (d, *J* = 8.6, 2H, H_q), 6.82 (d, *J* = 8.7, 2H, H_p), 4.95 (d, *J* = 8.1, 1H, NH), 4.58-4.42 (m, 1H, H_s), 4.20-4.12 (m, 4H, H_d, H_t), 4.12-4.05 (m, 2H, H_o), 3.90-3.79 (m, 4H, H_e, H_n), 3.74-3.55 (m, 16H, H_f, H_g, H_h, H_i, H_j, H_k, H_l, H_m), 3.10-2.93 (m, 2H, H_r), 1.42 (s, 9H, H_v), 1.23 (t, *J* = 7.2, 3H, H_u), 0.25 (s, 9H, H_w).

^{19}F NMR (376 MHz, $CDCl_3$, 298 K) δ: -63.27 (s, 3F, CF_3).

^{13}C NMR (101 MHz, $CDCl_3$, 298 K) δ: 171.6, 158.6, 157.6, 154.9, 131.6 (q, J_{C-F} = 32.7), 130.1, 128.1, 124.9, 123.3 (q, J_{C-F} = 272.6), 120.8 (q, J_{C-F} = 3.6), 120.4, 114.4, 112.2 (q, J_{C-F} = 3.6), 103.1, 95.7, 79.3, 70.6, 70.6, 70.4 (×3), 70.4 (×2), 70.3, 69.5, 69.2, 67.8, 67.2, 60.9, 54.4, 37.1, 28.1, 13.9, -0.4.

HR-ESI-MS *m/z* = 831.4070 $[M+NH_4]^+$ calc. 831.4069 for $C_{40}H_{62}F_3N_2O_{11}Si$.

$[\alpha]_D^{23}$ +7.0 (*c* 0.84, $CHCl_3$)

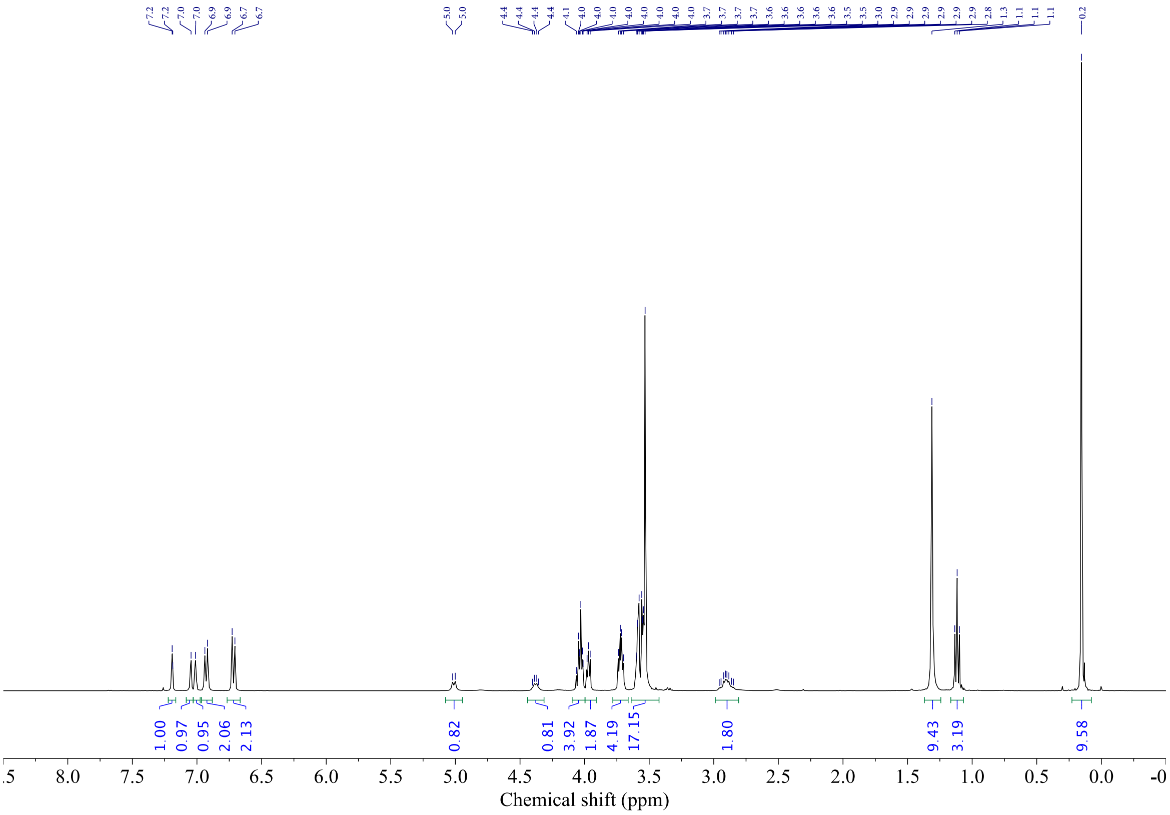

Figure S16. ^{1}H NMR of (*S*)-**S6a** ($CDCl_3$, 400 MHz, 298 K)

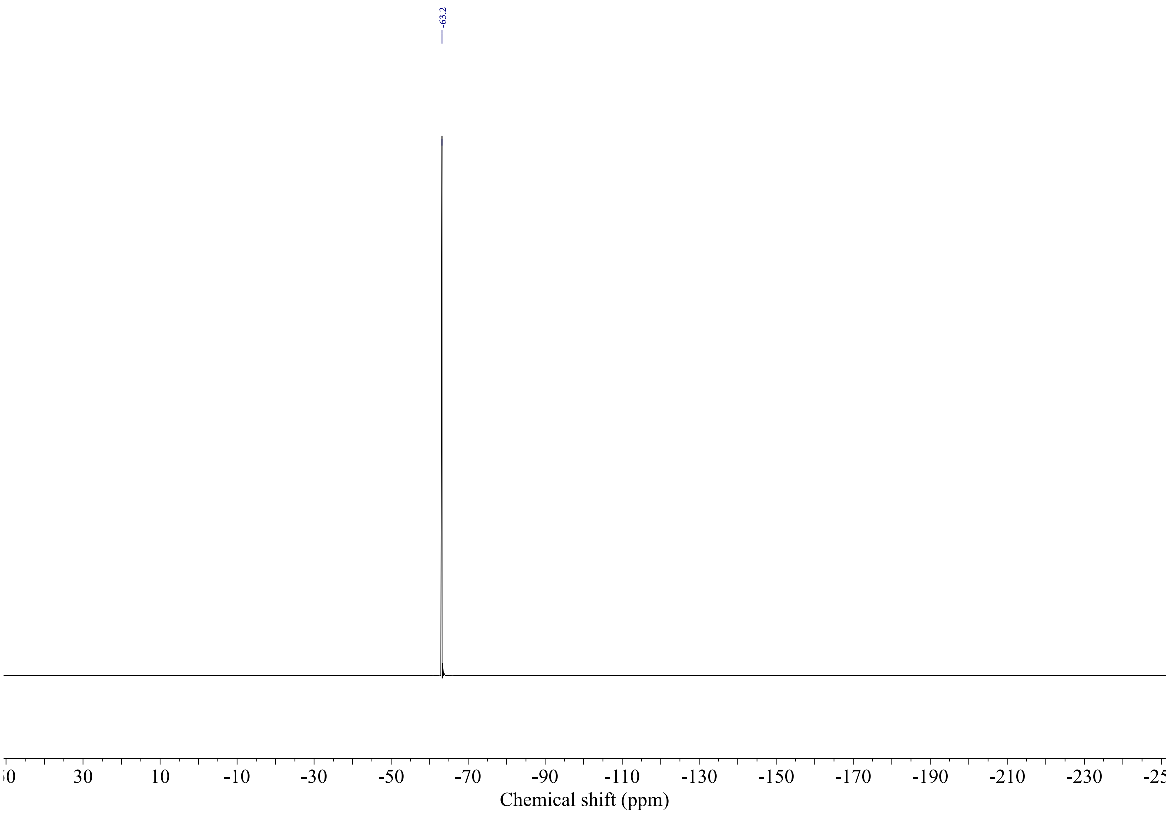

Figure S17. ^{19}F NMR of (*S*)-**S6a** ($CDCl_3$, 376 MHz, 298 K)

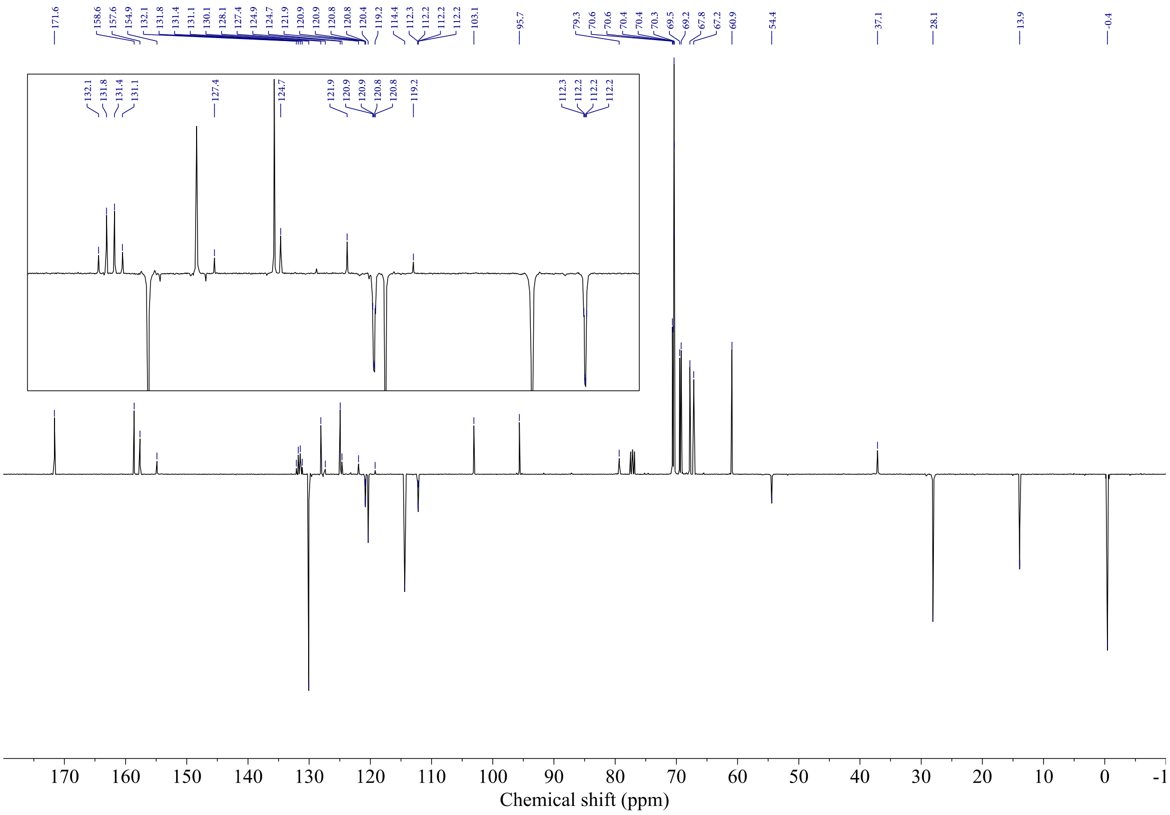

Figure S18. JMOD NMR of (*S*)-**S6a** ($CDCl_3$, 101 MHz, 298 K)

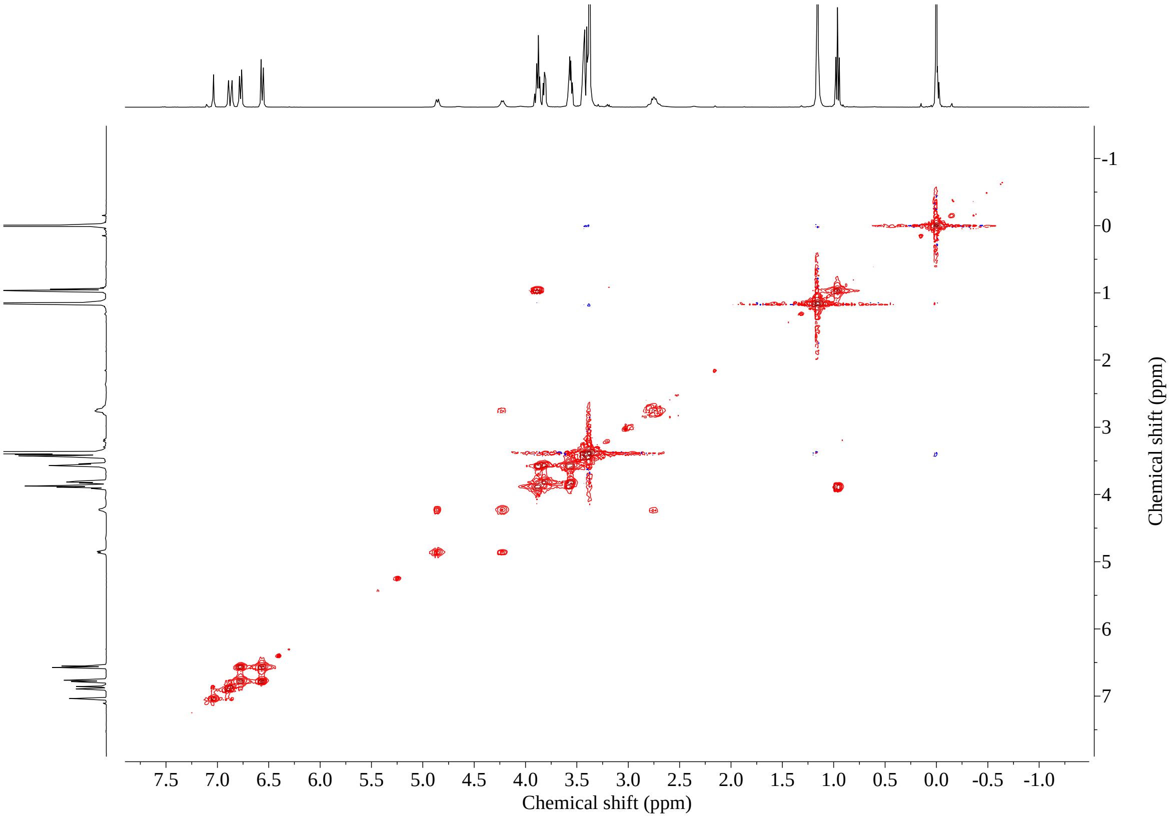

Figure S19. COSY NMR of (*S*)-**S6a** ($CDCl_3$, 298 K)

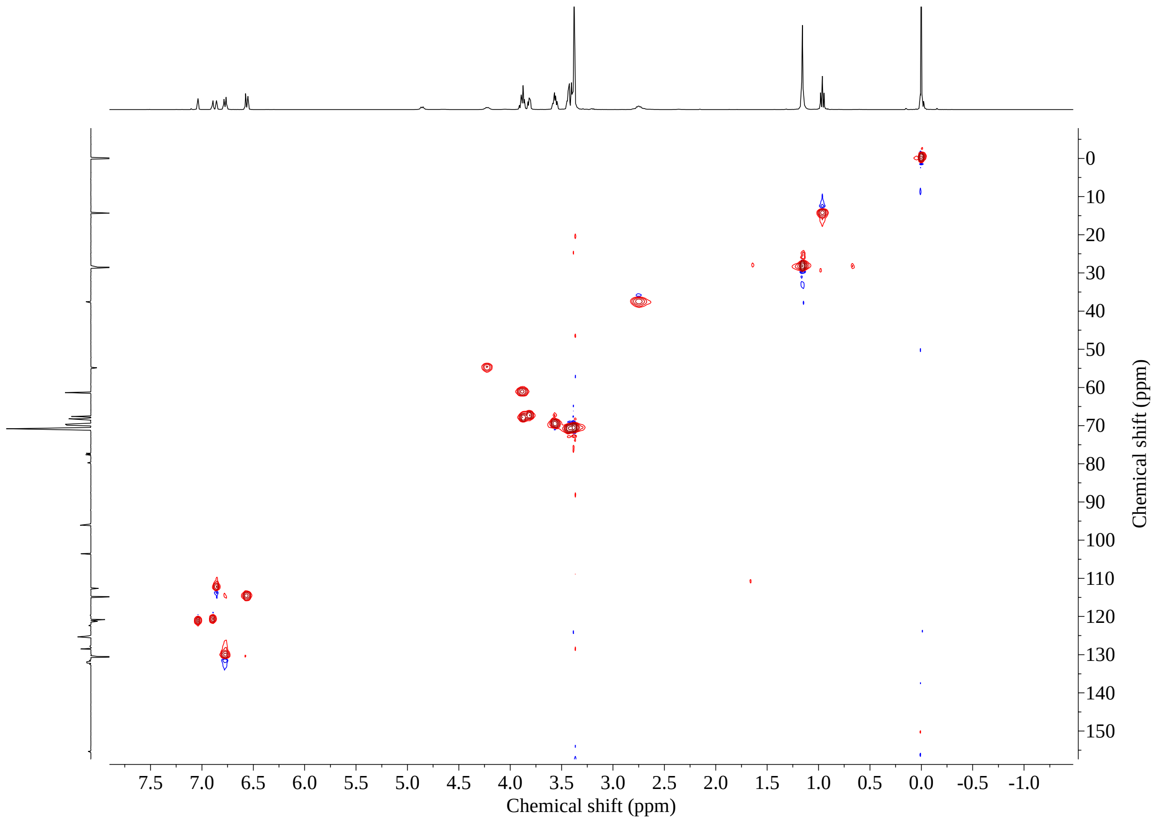

Figure S20. HSQC NMR of (*S*)-**S6a** ($CDCl_3$, 298 K)

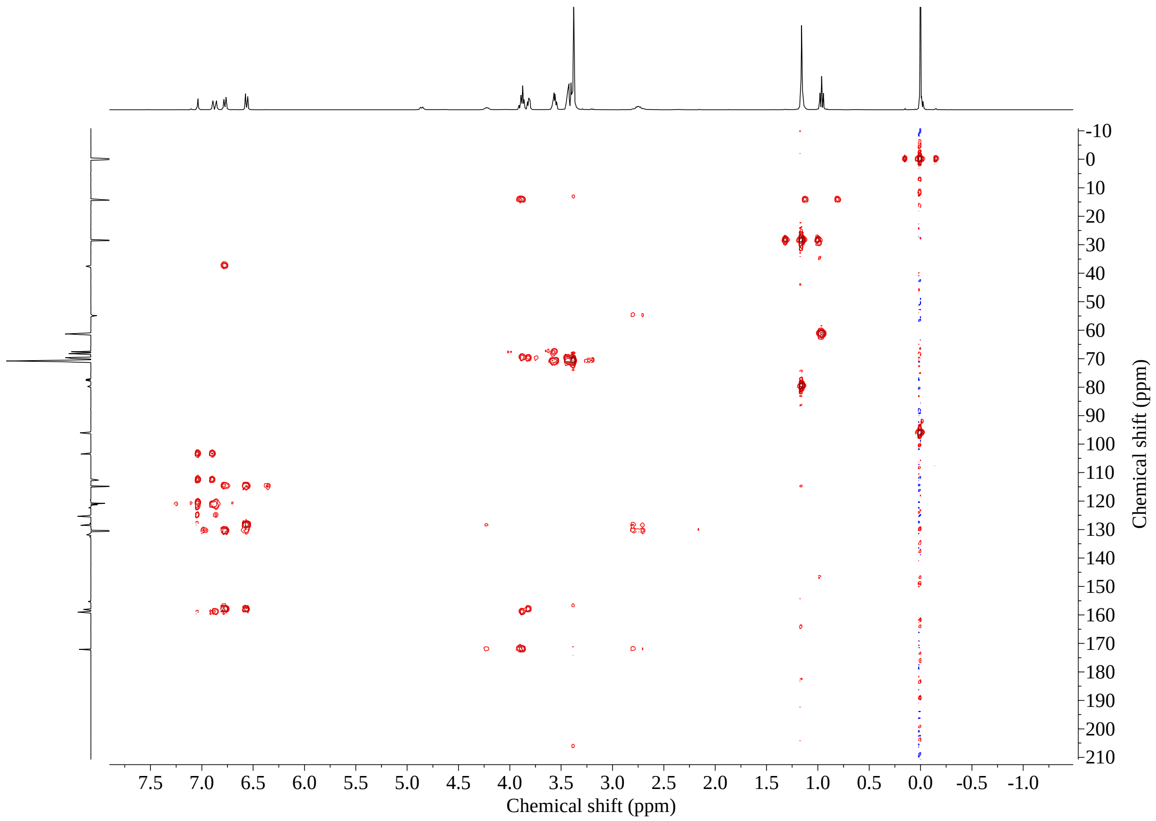

Figure S21. HMBC NMR of (*S*)-**S6a** ($CDCl_3$, 298 K)

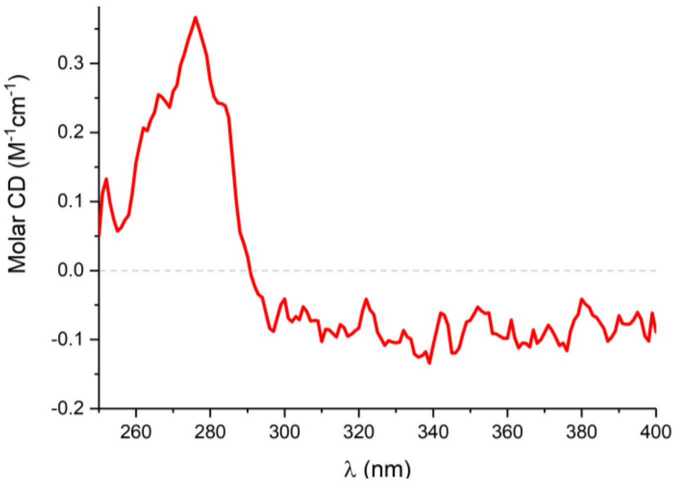

Figure S22. Circular Dichroism Spectra of (*S*)-**S6a** (45.5 μM) at 293 K in $CHCl_3$

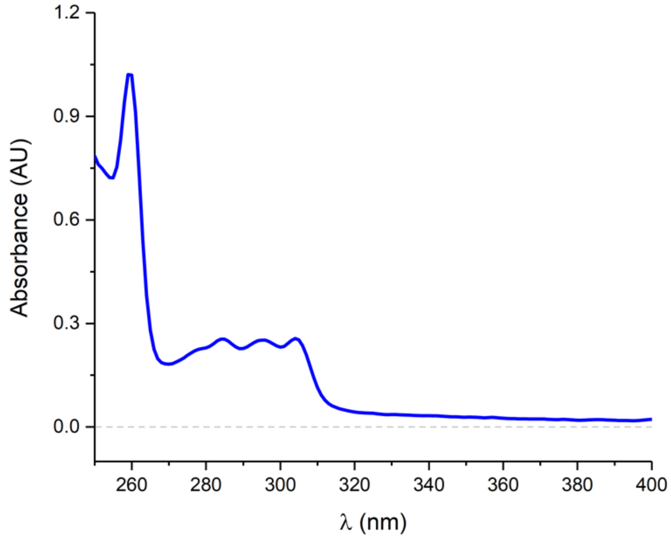

Figure S23. UV-Vis Spectra of (*S*)-**S6a** (45.5 μM) at 293 K in $CHCl_3$

Terminal acetylene (*S*)-S7a

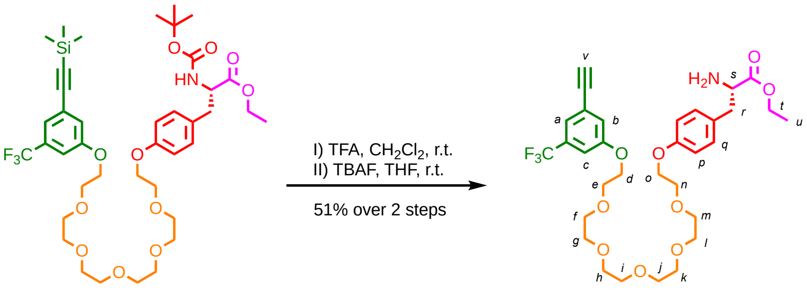

(*S*)-**S6a** (*S*)-**S7a**

(*S*)-**S6a** (152 mg, 0.19 mmol, 1.0 equiv.) was dissolved in CH_2Cl_2 (2 mL). Trifluoroacetic acid (0.15 mL, 1.9 mmol, 10.0 equiv.) was added dropwise to the solution and the mixture was stirred at rt for 16 h. The reaction mixture was quenched with sat. $NaHCO_3$ (10 mL), extracted with CH_2Cl_2 (20 mL) and the organic phase dried ($MgSO_4$). The solvent was removed *in vacuo*. The crude was re-dissolved in THF (5 mL), tetrabutylammonium fluoride (1 M in THF, 0.4 mL, 0.4 mmol, 2 equiv.) was added and the reaction mixture was stirred at rt for 4 h. The reaction mixture was partitioned between H_2O (20 mL) and EtOAc (30 mL), the phases separated and the organic layer dried ($MgSO_4$). The solvent was removed *in vacuo* and the crude was purified by column chromatography (deactivated SiO_2, CH_2Cl_2-acetone 0 → 20%) to give (*S*)-**S7a** as an orange oil (62 mg, 51% over 2 steps).

^{1}H NMR (400 MHz, $CDCl_3$, 298 K) δ 7.34-7.31 (m, 1H, H_a), 7.19-7.16 (m, 1H, H_b), 7.16-7.13 (m, 1H, H_c) 7.10 (d, J = 8.6, 2H, H_q), 6.84 (d, J = 8.7, 2H, H_p), 4.21-4.12 (m, 4H, H_o, H_t,), 4.09 (t, J = 4.9, 2H, H_d), 3.89-3.79 (m, 4H, H_e, H_n), 3.76-3.57, (m, 17H, H_f, H_g, H_h, H_i, H_j, H_k, H_l, H_m, H_s), 3.13 (s, 1H, H_v) 3.03 (dd, J = 13.5, 5.0, 1H, H_r), 2.85 (dd, J = 13.5, 7.4, 1H, H_r), 1.70 (br s, 2H, NH), 1.25 (t, J = 7.6, 3H, H_u).

^{19}F NMR (376 MHz, $CDCl_3$, 298 K) δ: -63.29 (s, 3F, CF_3)

^{13}C NMR (101 MHz, $CDCl_3$, 298 K) δ 174.8, 158.7, 157.5, 131.8 (q, J_{C-F} = 32.7), 130.1, 129.2, 124.0, 123.3 (q, J_{C-F} = 272.7), 121.0 (q, J_{C-F} = 3.8), 120.9, 114.5, 112.4 (q, J_{C-F} = 3.7), 81.8, 78.8, 70.7, 70.6, 70.4, 70.4 (×3), 70.4 (×2), 69.5, 69.3, 67.8, 67.2, 60.6, 55.7, 40.0, 14.0.

HR-ESI-MS *m/z* = 642.2901 $[M+H]^+$ calc. 642.2884 for $C_{32}H_{43}F_3NO_9$.

$[\alpha]_D^{23}$ −0.7 (c 0.61, $CHCl_3$)

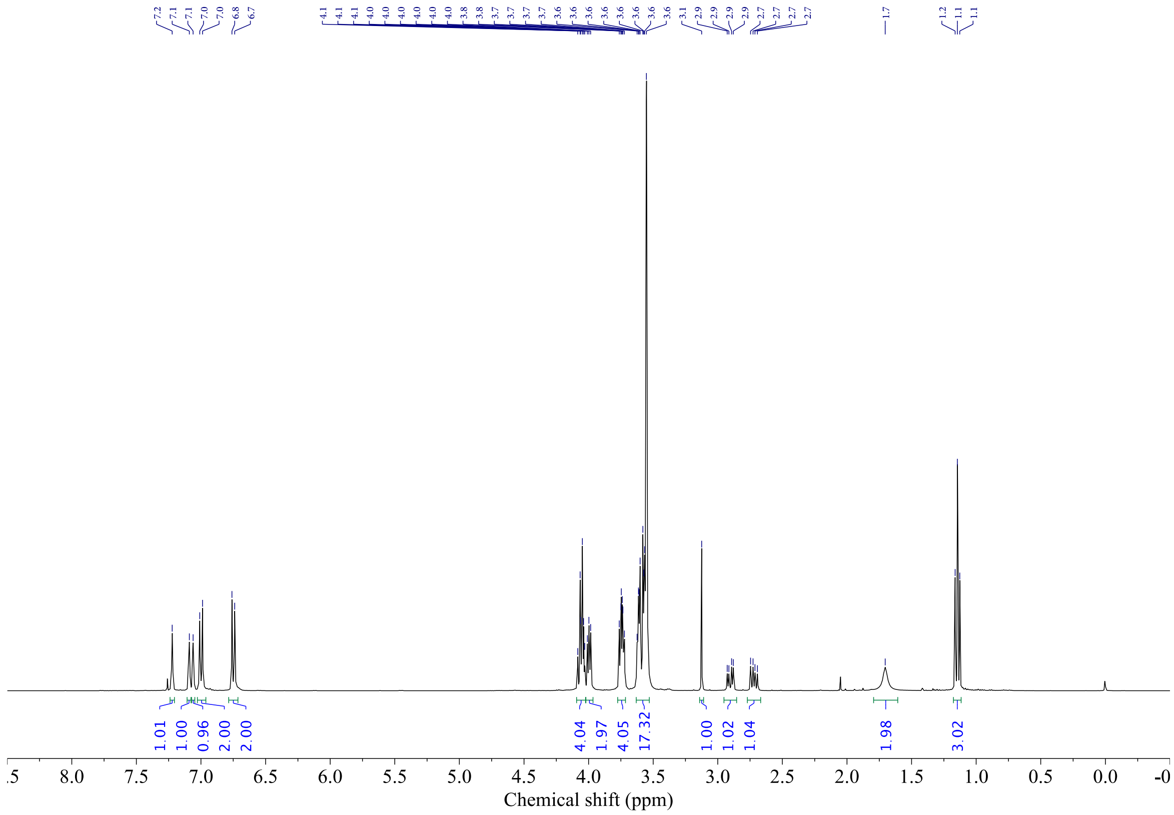

Figure S24. ^{1}H NMR of (S)-**S7a** ($CDCl_3$, 400 MHz, 298 K)

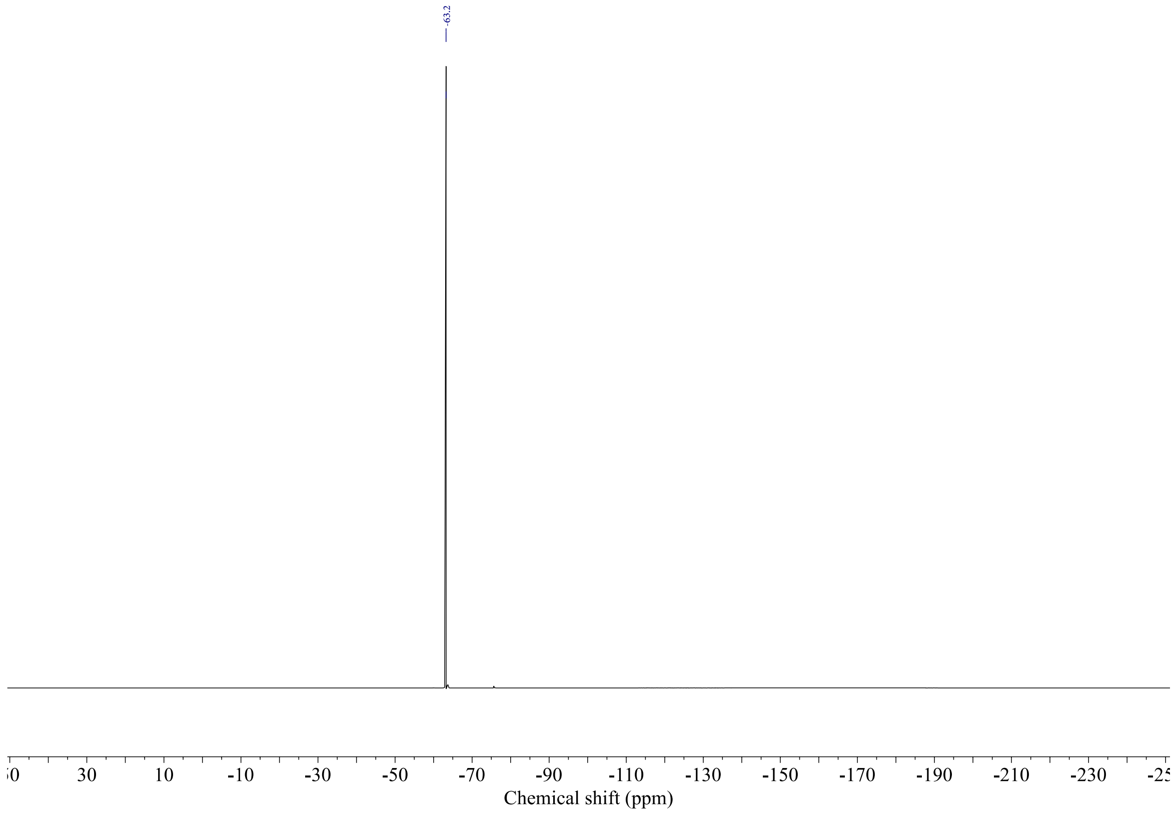

Figure S25. ^{19}F NMR of (*S*)-**S7a** ($CDCl_3$, 376 MHz, 298 K)

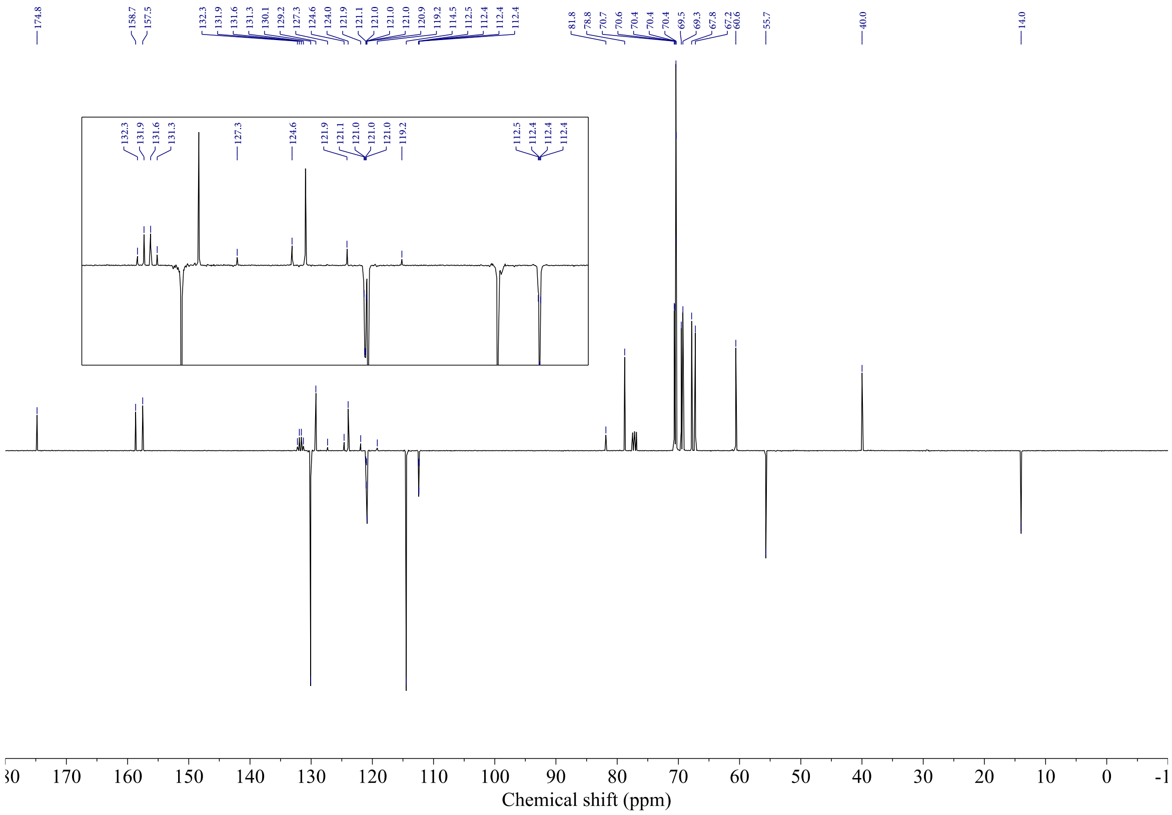

Figure S26. JMOD NMR of (*S*)-**S7a** ($CDCl_3$, 101 MHz, 298 K)

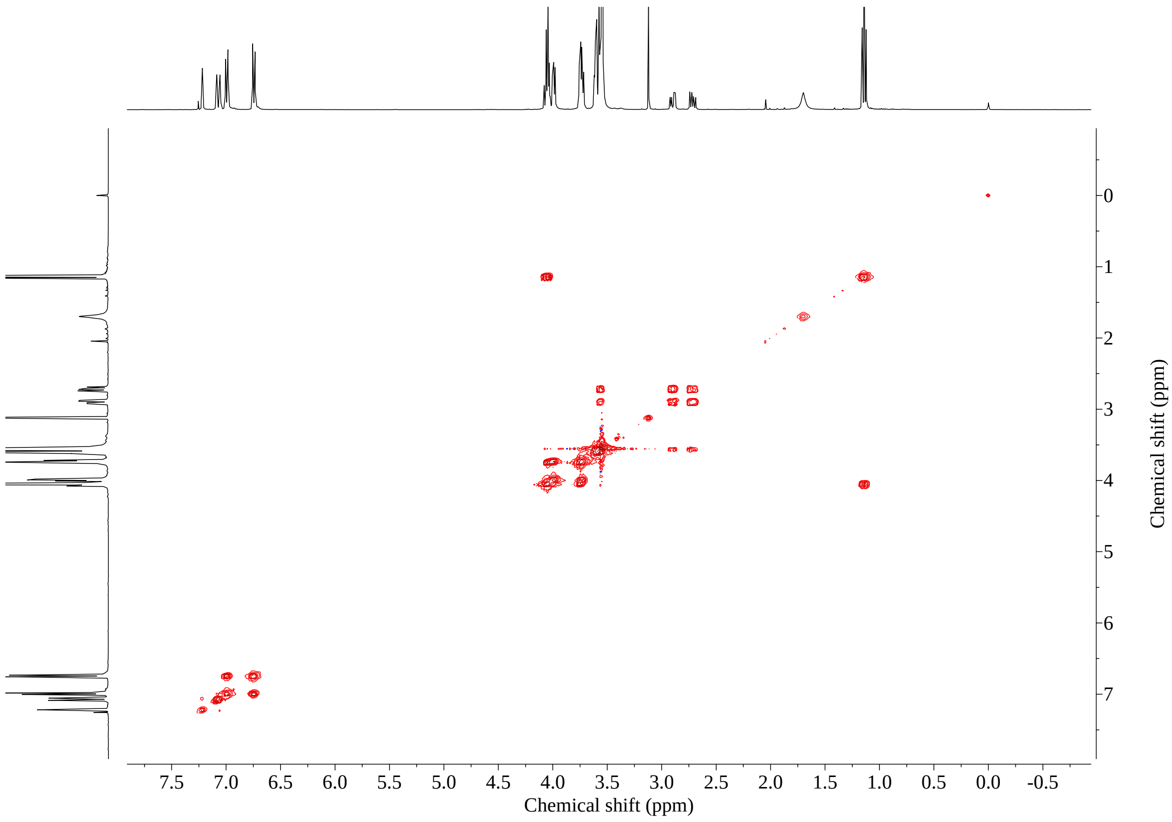

Figure S27. COSY NMR of (*S*)-**S7a** ($CDCl_3$, 298 K)

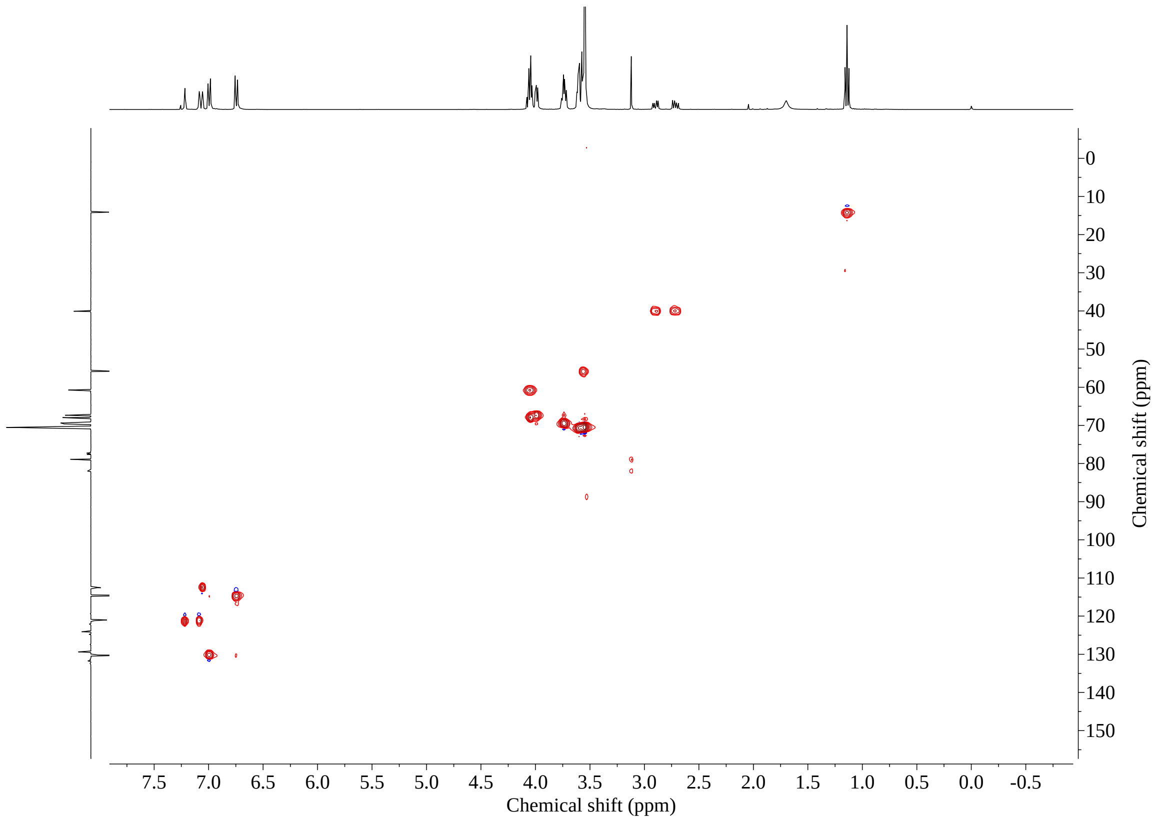

Figure S28. HSQC NMR of (*S*)-**S7a** ($CDCl_3$, 298 K)

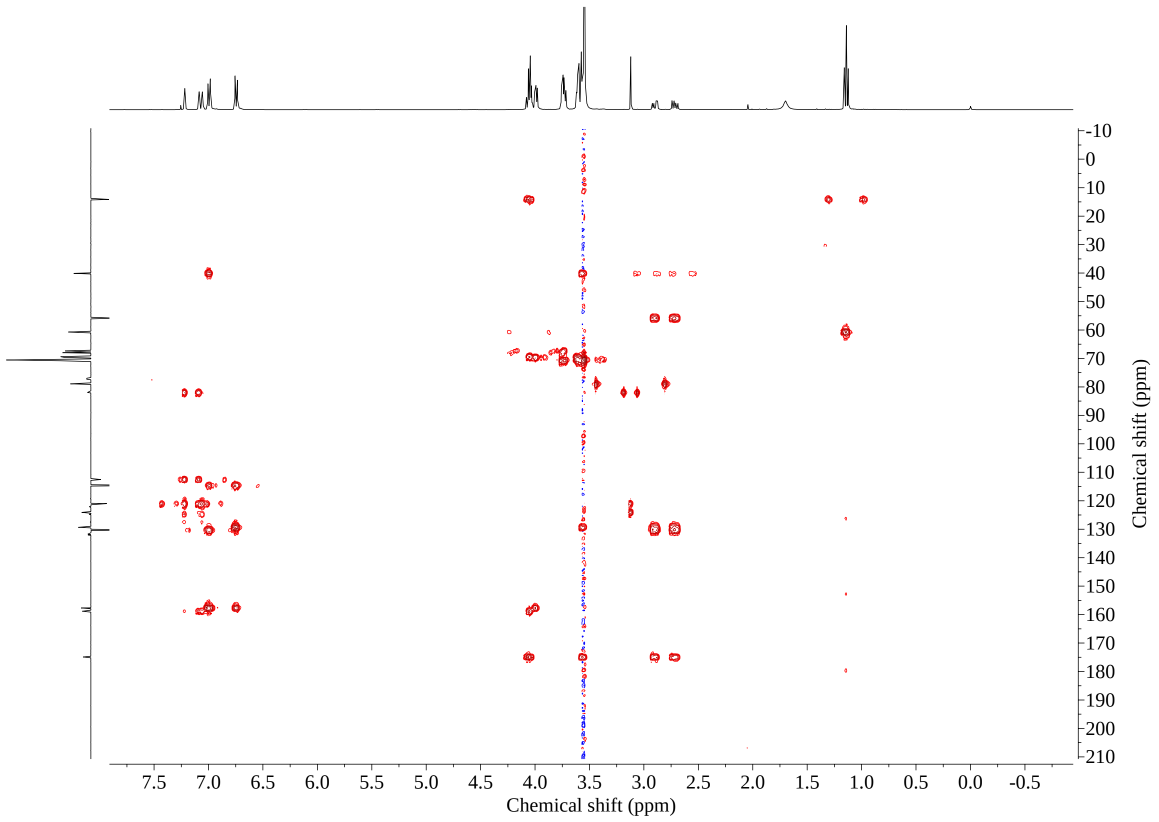

Figure S29. HMBC NMR of (*S*)-**S7a** ($CDCl_3$, 298 K)

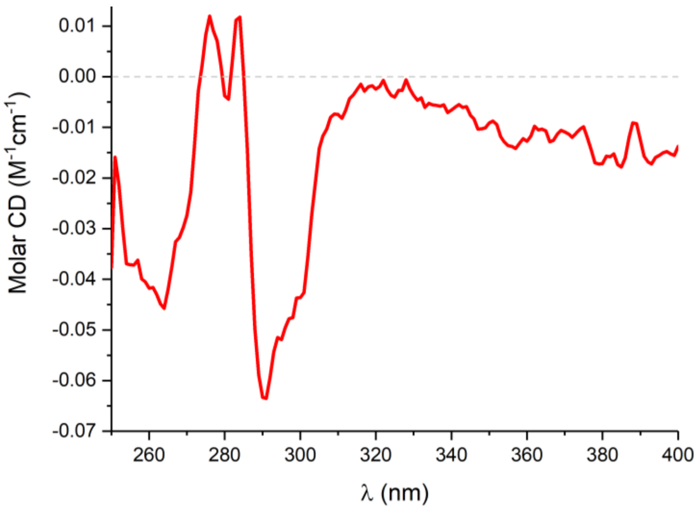

Figure S30. Circular Dichroism Spectra of (*S*)-**S7a** (163 µM) at 293 K in $CHCl_3$

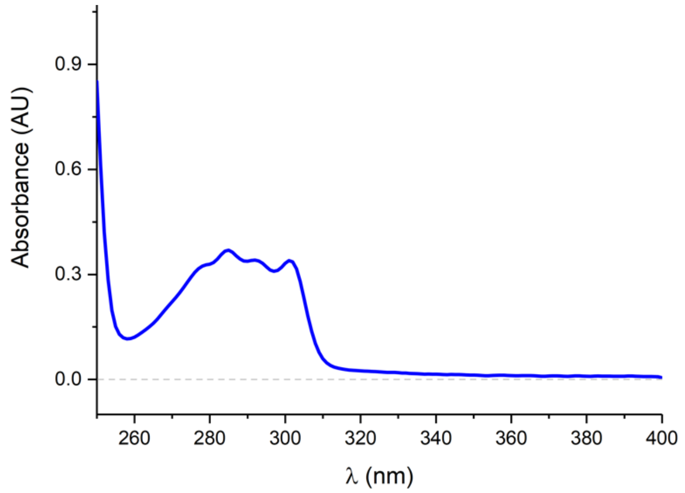

Figure S31. UV-Vis Spectra of (*S*)-**S7a** (163 µM) at 293 K in $CHCl_3$

Macrocycle precursor (*S*)-1a

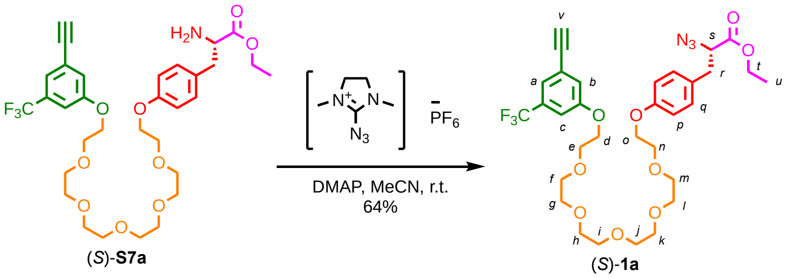

(*S*)-**S7a** (63 mg, 0.097 mmol, 1.0 equiv.), DMAP (35.6 mg, 0.291 mmol, 3.0 equiv.) and ADMP (41.6 mg, 0.154 mmol, 1.5 equiv.) were dissolved in CH_3CN (1 mL). The mixture was stirred at rt for 16h before quenching with 2 mL of water. The aqueous solution was extracted with 5 mL of CH_2Cl_2 and dried over $MgSO_4$. The solvent was removed *in vacuo* and the crude was purified by column chromatography (CH_2Cl_2-EtOAc 100 : 0 → 70 : 30) to obtain (*S*)-**1a** as a colorless oil (42.9 mg, 64% yield).

^{1}H NMR (400 MHz, $CDCl_3$, 298 K) δ 7.35-7.32 (m, 1H, H_a), 7.19-7.16 (m, 1H, H_b), 7.16-7.10 (m, 3H, H_c, H_q), 6.86 (dt, *J* = 8.5, 2.4, 2H, H_p), 4.22 (q, *J* = 7.0, 2H, H_t), 4.18-4.12 (m, 2H, H_d), 4.12-4.08 (m, 2H, H_o), 3.99 (dd, *J* = 8.5, 5.6, 1H, H_s), 3.89-3.80 (m, 4H, H_e, H_p), 3.74-3.61 (m, 16H, H_f, H_g, H_h, H_i, H_j, H_k, H_l, H_m), 3.14-3.06 (m, 2H, H_r, H_v), 2.95 (dd, *J* = 8.5, 14.4, 1H, H_r), 1.27 (7, *J* = 7.0, 3H, H_u)

^{19}F NMR (376 MHz, $CDCl_3$, 298 K) δ: -63.29 (s, 3F, CF_3)

^{13}C NMR (101 MHz, $CDCl_3$, 298 K) δ 170.1, 158.9, 158.1, 132.1 (q, $J_{C\text{-}F}$ = 32.7), 130.3, 128.2, 124.2, 123.5 (q, $J_{C\text{-}F}$ = 272.6), 121.4 (q, $J_{C\text{-}F}$ = 3.8), 121.1, 114.9, 112.8 (q, $J_{C\text{-}F}$ = 3.8), 82.2, 78.7, 71.0, 70.9, 70.7, 70.7 (×3), 70.7 (×2), 69.8, 69.6, 68.1, 67.5, 63.5, 61.9, 36.9, 14.2.

HR-ESI-MS *m/z* = 690.2625 $[M+Na]^+$ calc. 690.2609 for $C_{32}H_{40}F_3N_3NaO_9$.

$[\alpha]_D^{23}$ −16.7 (*c* 0.50, $CHCl_3$)

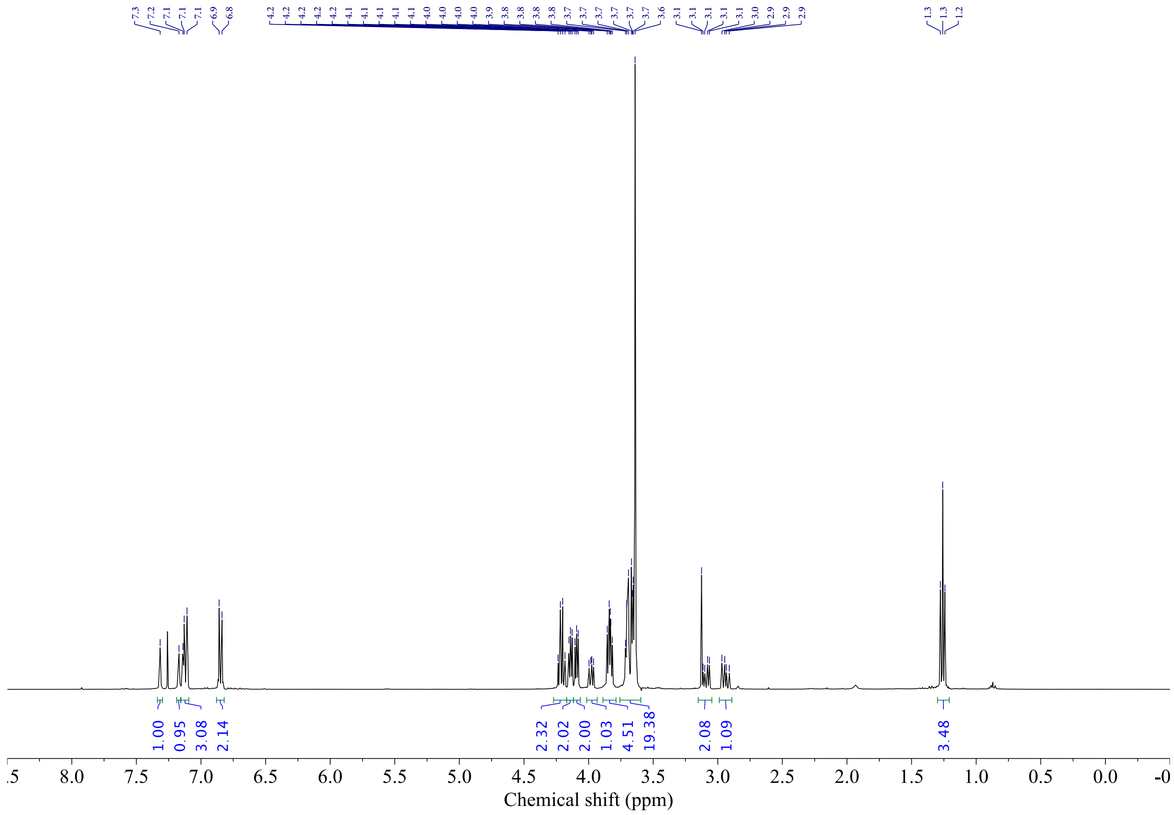

Figure S32. ^{1}H NMR of (*S*)-**1a** ($CDCl_3$, 400 MHz, 298 K)

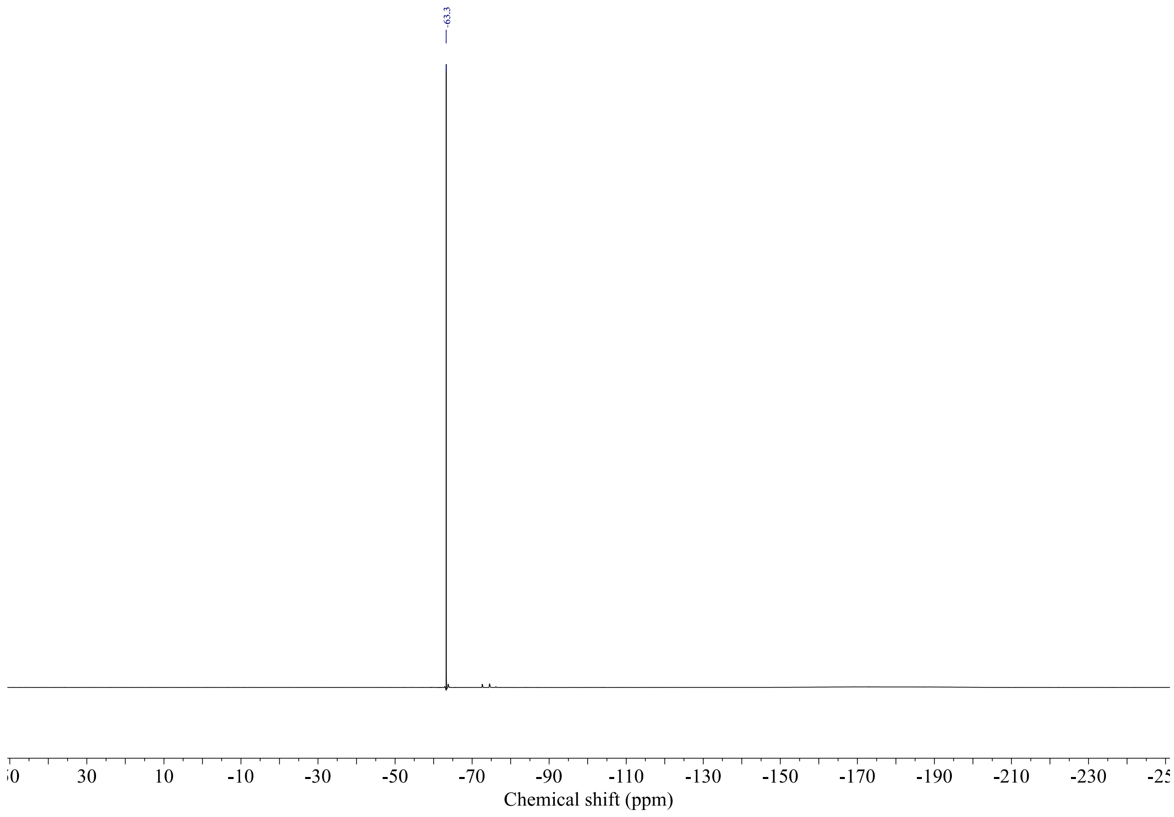

Figure S33. ^{19}F NMR of (*S*)-**1a** ($CDCl_3$, 376 MHz, 298 K)

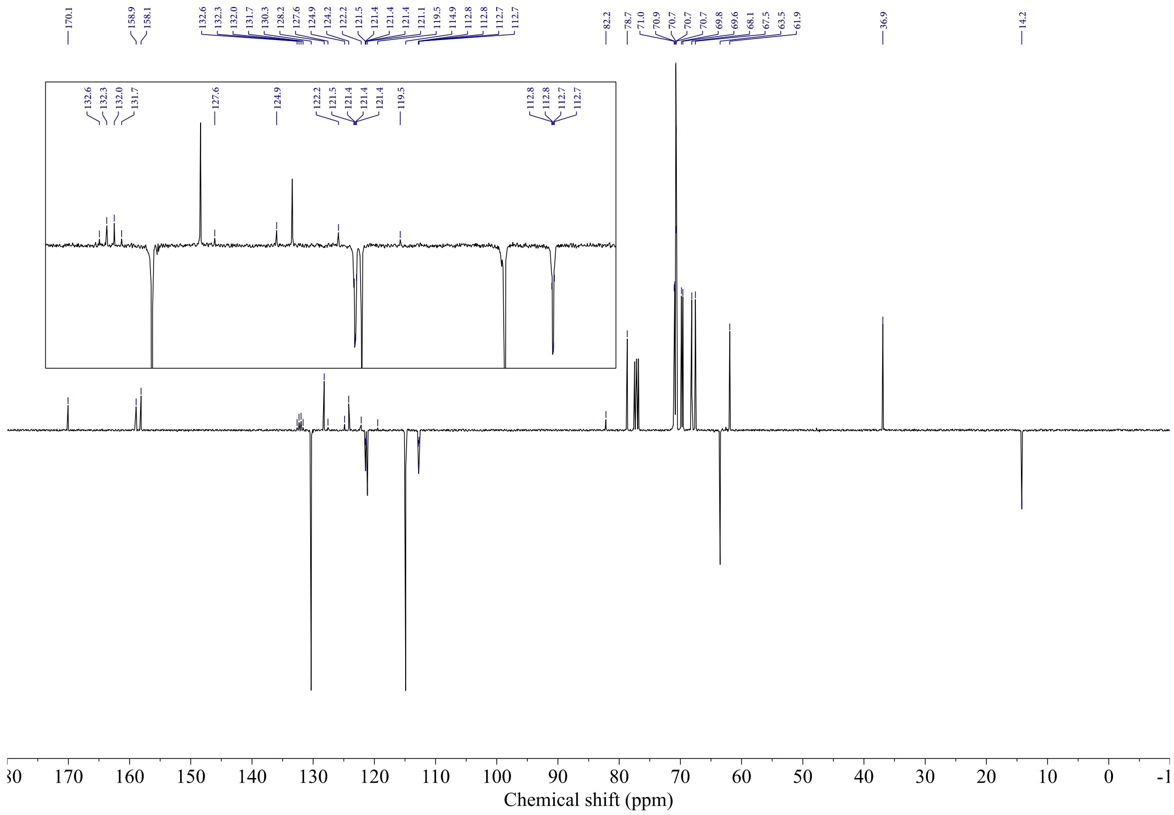

Figure S34. JMOD NMR of (*S*)-**1a** ($CDCl_3$, 101 MHz, 298 K)

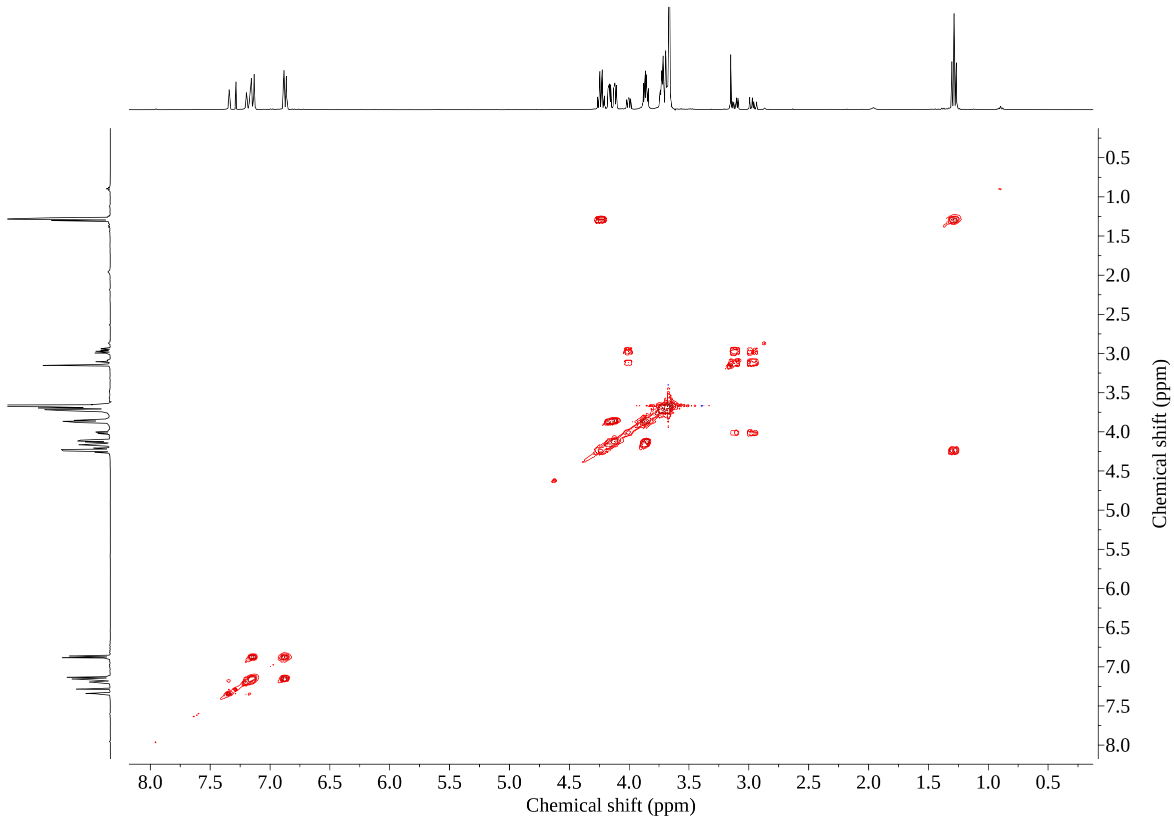

Figure S35. COSY NMR of (*S*)-**1a** ($CDCl_3$, 298 K)

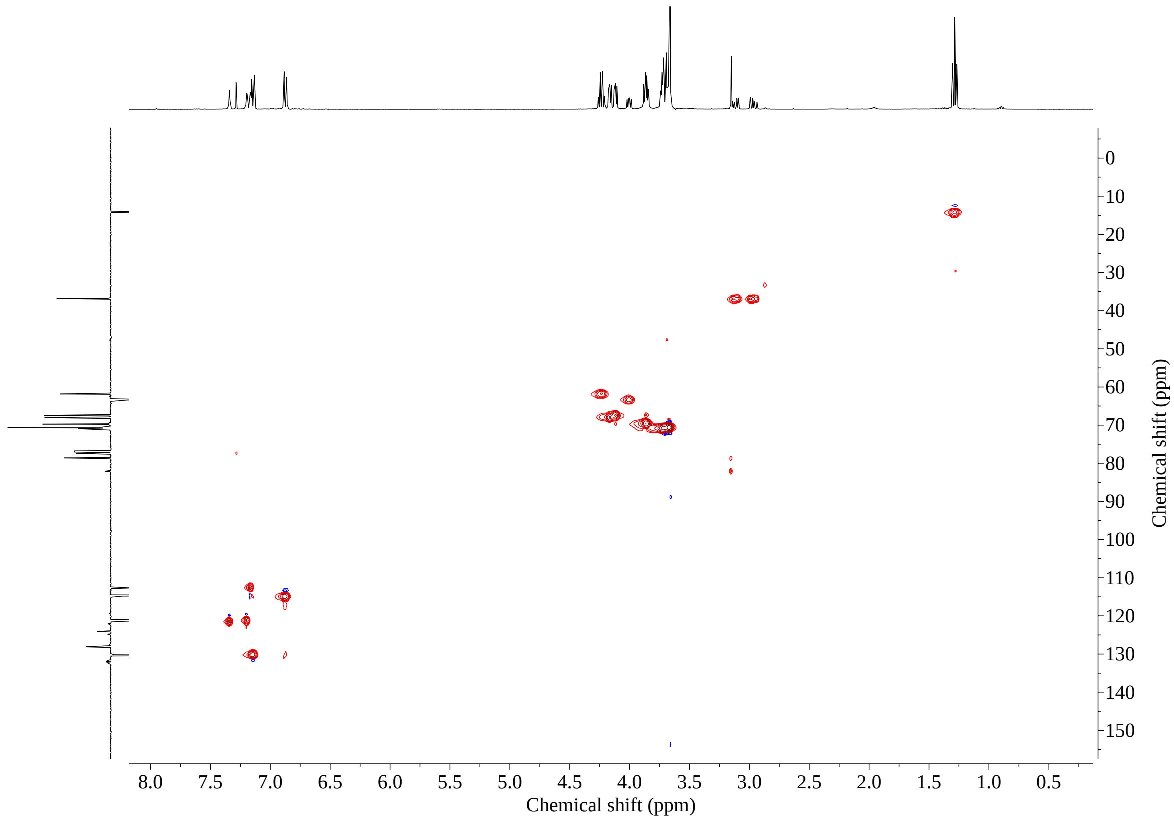

Figure S36. HSQC NMR of (*S*)-**1a** ($CDCl_3$, 298 K)

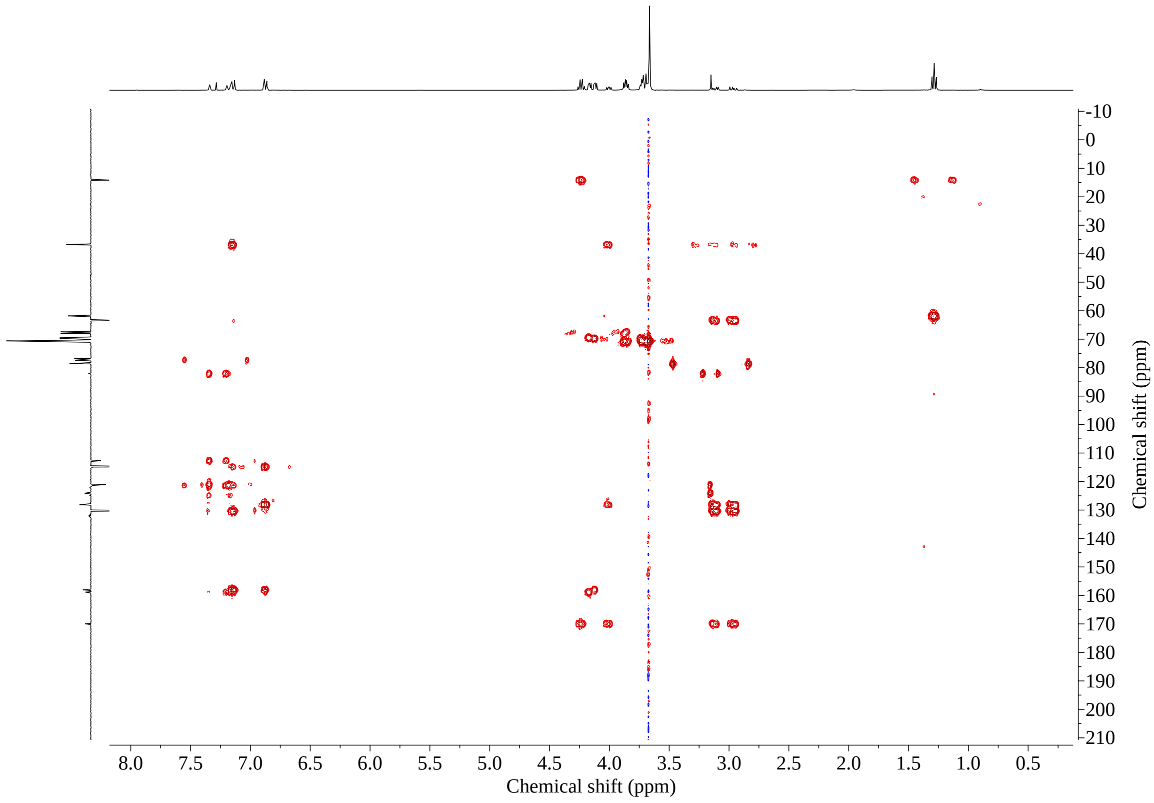

Figure S37. HMBC NMR of (*S*)-**1a** ($CDCl_3$, 298 K)

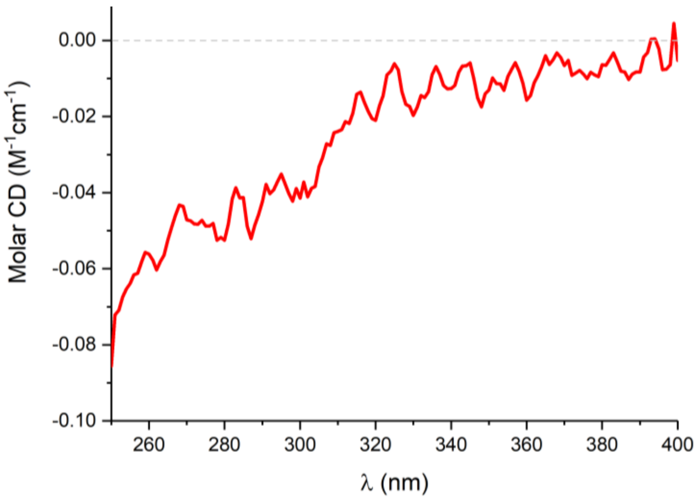

Figure S38. Circular Dichroism Spectra of (*S*)-**1a** (280 µM) at 293 K in $CHCl_3$

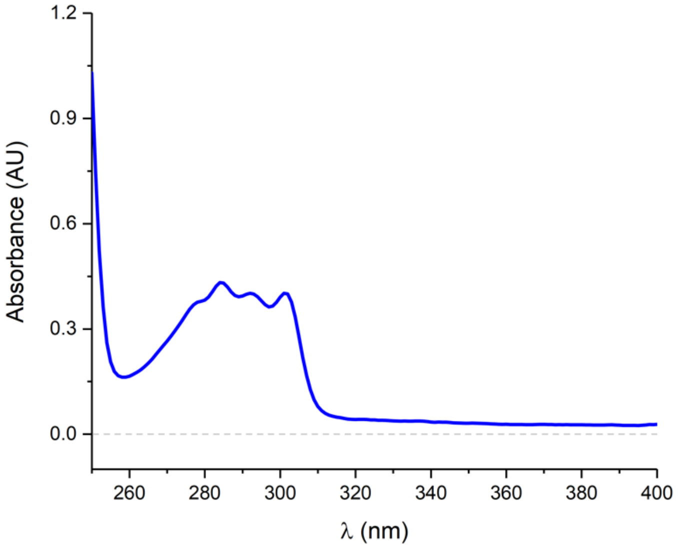

Figure S39. UV-Vis Spectra of (*S*)-**1a** (280 µM) at 293 K in $CHCl_3$

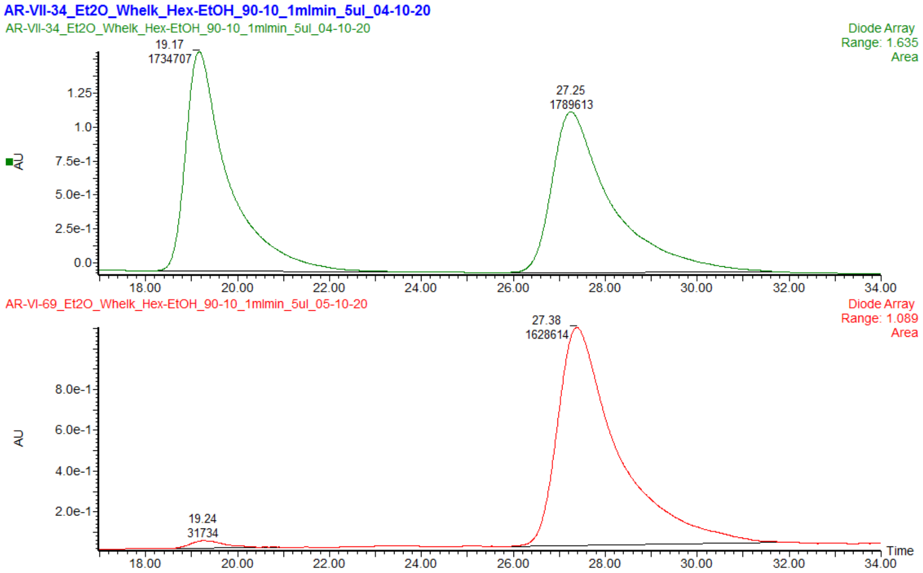

Figure S40. CSP-HPLC of (*S*)-**1a** (loaded in Et_2O). (*S,S*)Whelk, *n*-hexane-ethanol 90 : 10, flowrate 1.0 mLmin^{-1}, retention times (*R*)-**1a** (19.2 min, 31734, 1.9%), (*S*)-**1a** (27.4 min, 1628614, 98.1%).

Boc amine (*S*)-S5b

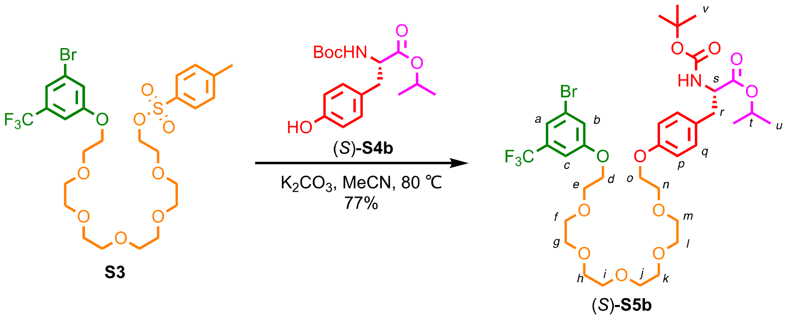

S3 (703 mg, 1.06 mmol, 1.0 equiv.), (*S*)-**S4b** (345 mg, 1.06 mmol, 1.0 equiv.) and K_2CO_3 (586 mg, 4.24 mmol, 4.0 equiv.) were suspended in CH_3CN (10 mL) and the resulting suspension was heated at reflux for 48 h. The reaction mixture was filtered over a Celite® pad, which was washed with CH_2Cl_2 (20 mL). The washings were combined, and the solvent was removed *in vacuo*. The crude was purified by column chromatography (petrol-Et_2O 50 : 50 → 25 : 75) to give (*S*)-**S5b** as a pale yellow oil (662 mg, 77%).

^{1}H NMR (400 MHz, $CDCl_3$, 298 K) δ 7.34-7.31 (m, 1H, H_a), 7.23 (t, J = 2.0, 1H, H_b), 7.10-7.08 (m, 1H, H_c), 7.03 (d, J = 8.2, 2H, H_q), 6.81 (d, J = 8.2, 2H, H_p), 5.04-4.92 (m, 2H, NH, H_t), 4.45 (q, J = 6.4, 1H, H_s), 4.16-4.11 (m, 2H, H_d), 4.10-4.05 (m, 2H, H_o), 3.87-3.79 (m, 4H, H_e, H_n), 3.74-3.60 (m, 16H, H_f, H_g, H_h, H_i, H_j, H_k, H_l, H_m), 3.06-2.88 (m, 2H, H_r), 1.41 (s, 9H, H_v), 1.22-1.18 (m, 6H, H_u)

^{19}F NMR (376 MHz, $CDCl_3$, 298 K) δ: -63.16 (s, 3F, CF_3)

^{13}C NMR (101 MHz, $CDCl_3$, 298 K) δ 171.4, 159.8, 157.9, 155.1, 133.0 (q, $J_{C\text{-}F}$ = 32.9), 130.4, 128.4, 123.1, 123.1 (q, $J_{C\text{-}F}$ = 272.9), 121.3, 120.8 (q, $J_{C\text{-}F}$ = 3.8), 114.6, 110.9 (q, $J_{C\text{-}F}$ = 3.8), 79.7, 71.0, 70.9, 70.7, 70.7 (×3), 70.6 (×2), 69.8, 69.5, 69.1, 68.3, 67.5, 54.7, 37.5, 28.4, 21.8, 21.8.

HR-ESI-MS *m/z* = 827.2933 $[M+NH_4]^+$ calc. 827.2936 for $C_{36}H_{55}BrF_3N_2O_{11}$.

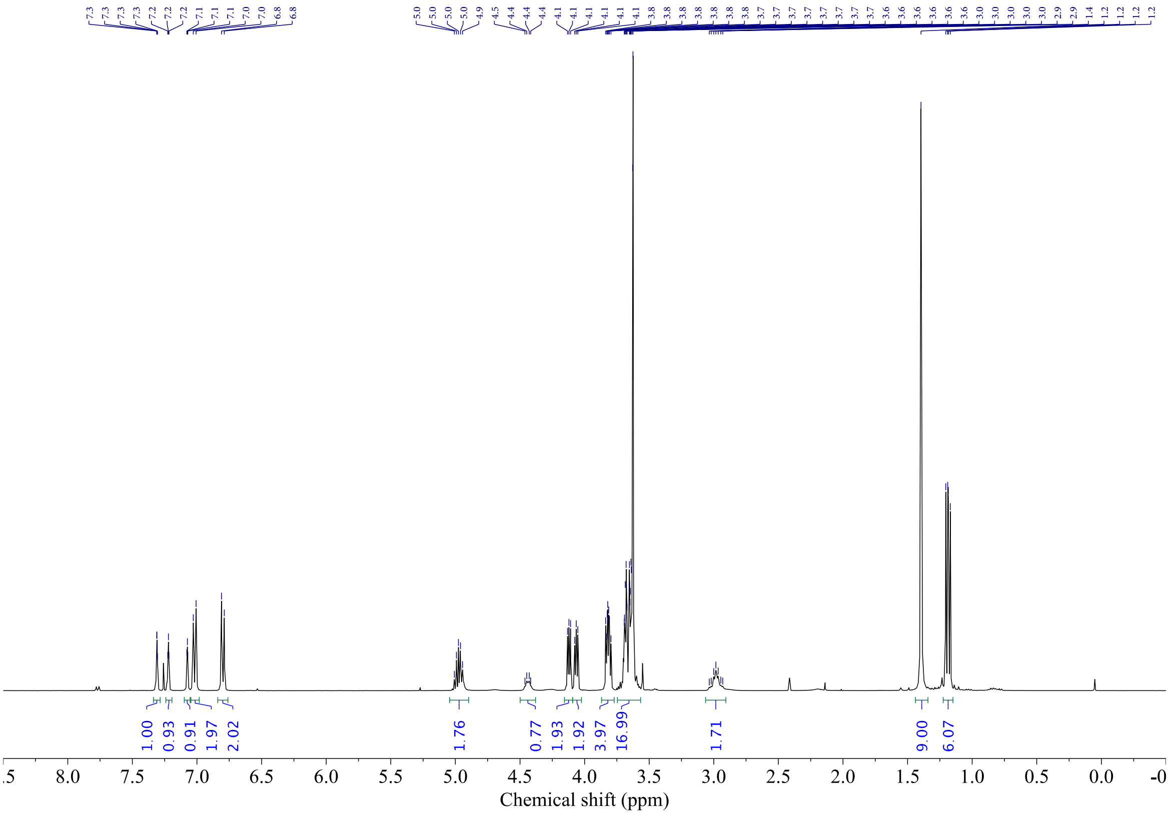

Figure S41. ^{1}H NMR of (*S*)-**S5b** ($CDCl_3$, 400 MHz, 298 K)

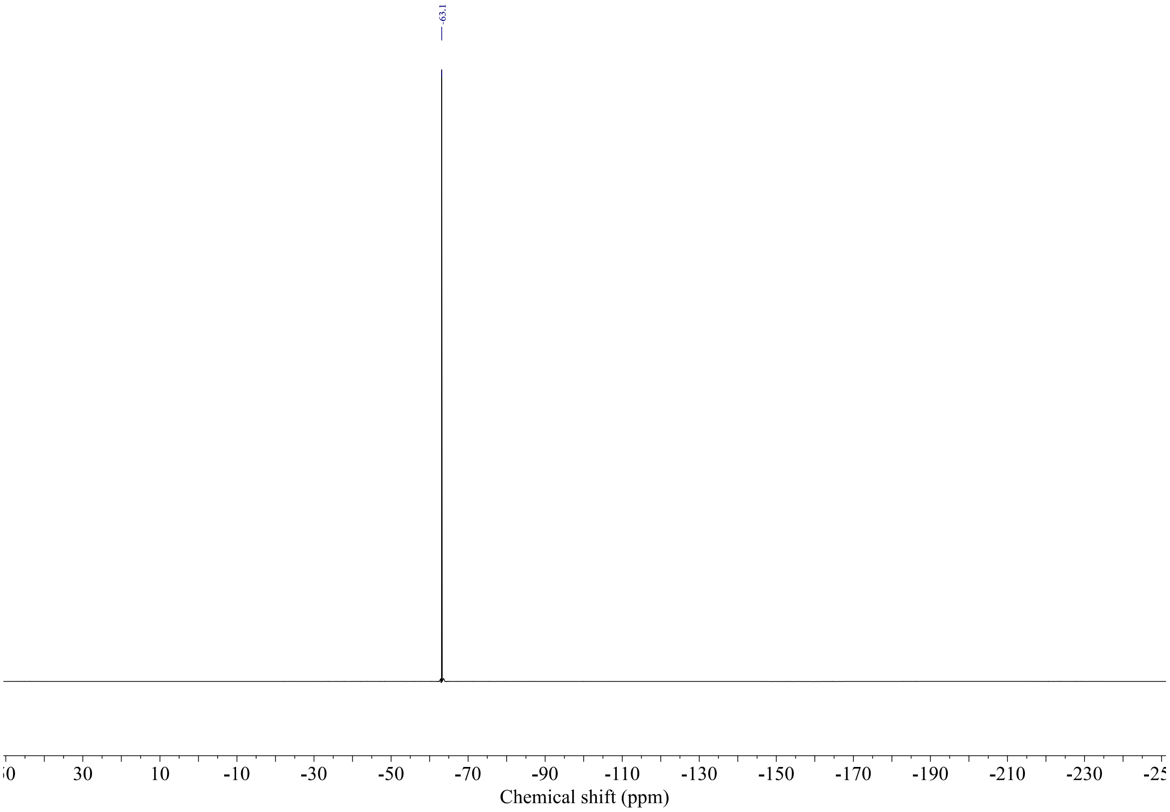

Figure S42. ^{19}F NMR of (*S*)-**S5b** ($CDCl_3$, 376 MHz, 298 K)

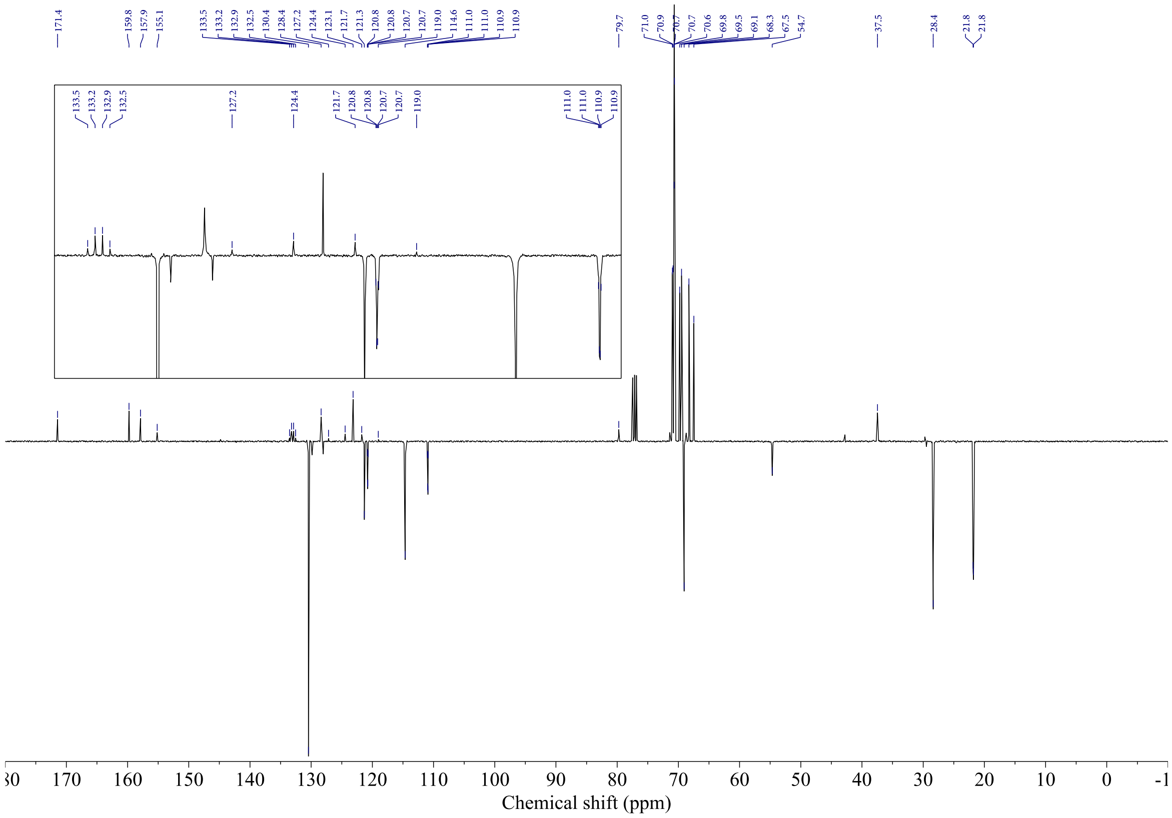

Figure S43. JMOD NMR of (*S*)-**S5b** ($CDCl_3$, 101 MHz, 298 K)

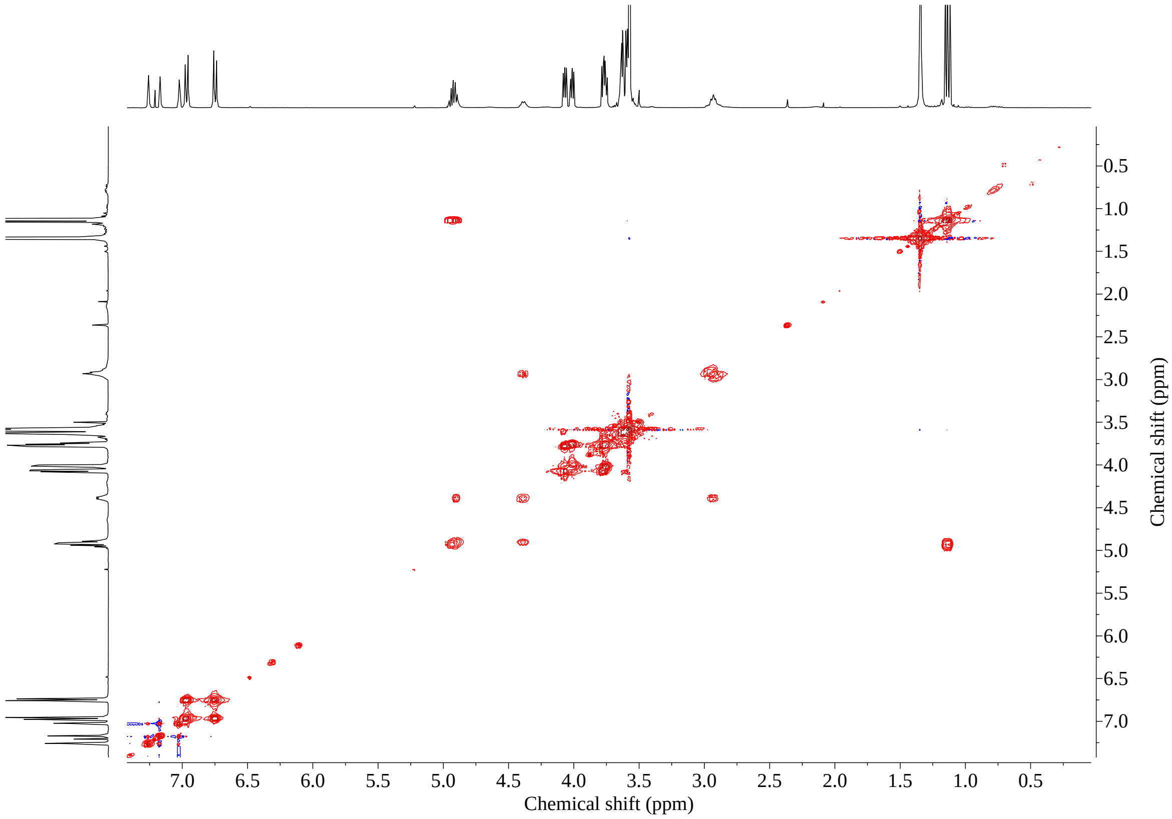

Figure S44. COSY NMR of (*S*)-**S5b** ($CDCl_3$, 298 K)

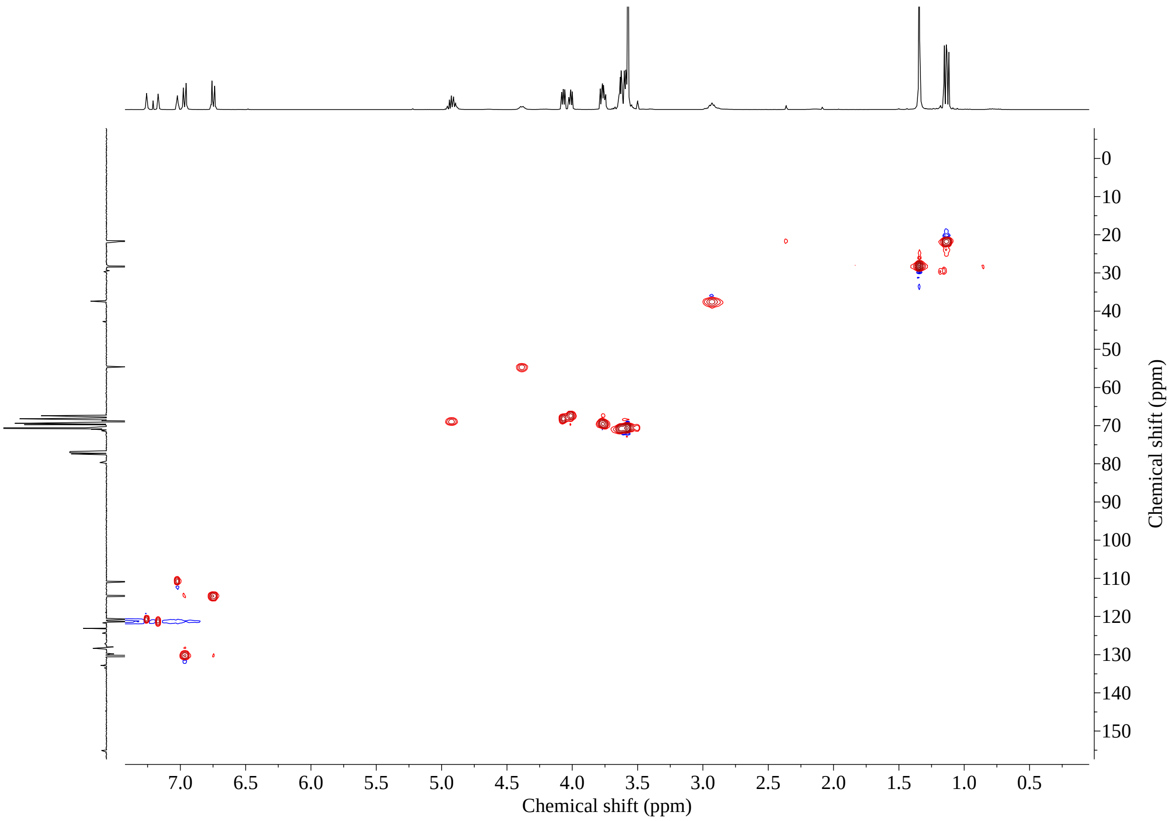

Figure S45. HSQC NMR of (*S*)-**S5b** ($CDCl_3$, 298 K)

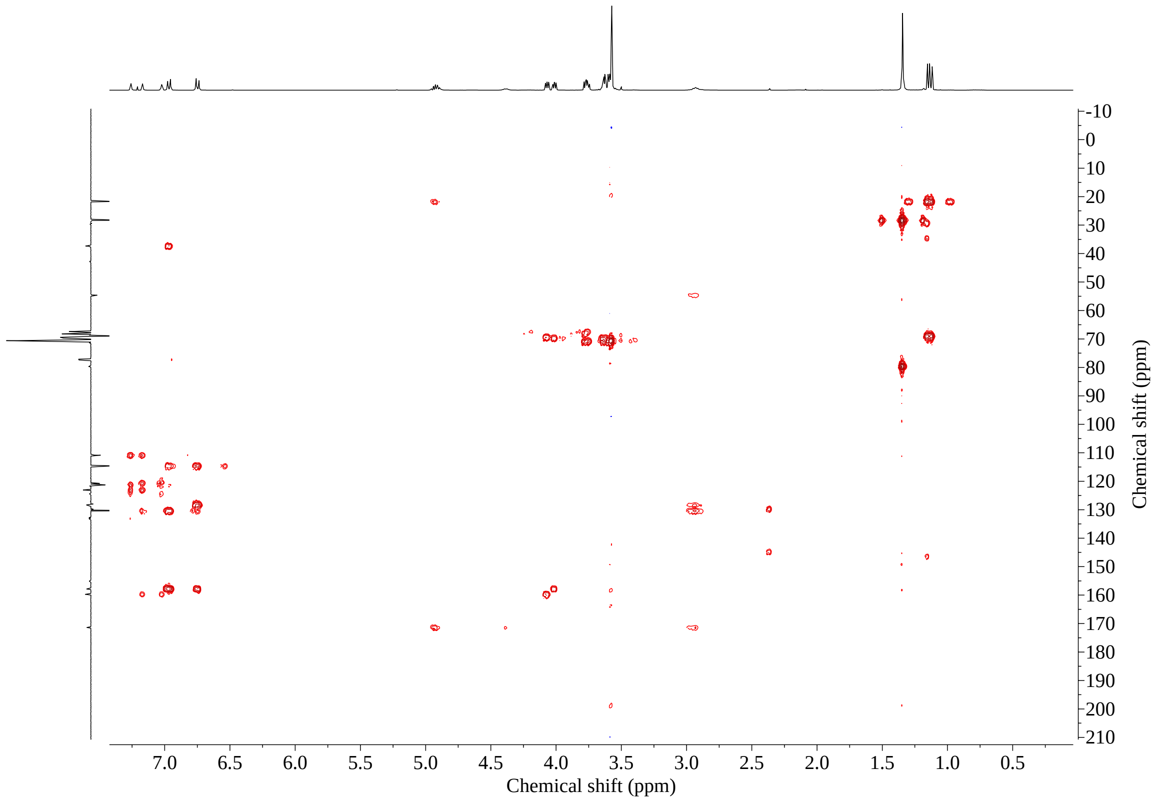

Figure S46. HMBC NMR of (*S*)-**S5b** ($CDCl_3$, 298 K)

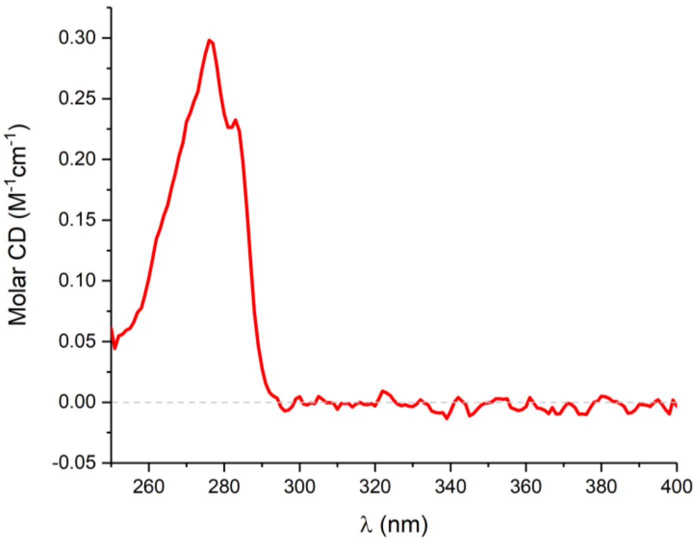

Figure S47. Circular Dichroism Spectra of (*S*)-**S5b** (217 µM) at 293 K in $CHCl_3$

TMS acetylene (*S*)-S6b

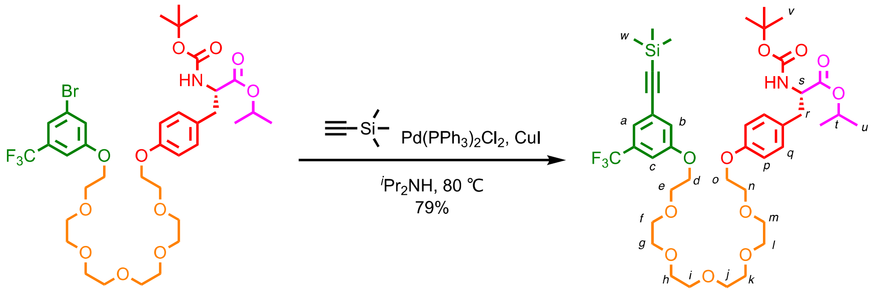

(*S*)-**S5b** (*S*)-**S6b**

(*S*)-**S5b** (662 mg, 0.82 mmol, 1.0 equiv.), $Pd(PPh_3)_2Cl_2$ (12 mg, 0.020 mmol, 0.02 equiv.) and CuI (6 mg, 0.03 mmol, 0.04 equiv.) were suspended in $^iPr_2NH$ (8 mL). The resulting suspension was degassed by bubbling N_2 through the solution over 5 min. Ethynyltrimethylsilane (174 µL, 1.22 mmol, 1.5 equiv.) was added and the mixture was stirred at 80 °C for 96 h. The solvent was removed *in vacuo* and the crude was purified by column chromatography (petrol-Et_2O 50 : 50 → 25 : 75) to give (*S*)-**S6b** as an orange oil (535 mg, 79%).

^{1}H NMR (400 MHz, $CDCl_3$, 298 K) δ 7.31-7.29 (m, 1H [note: signal overlaps with residual $CHCl_3$; integral shown is the expected value], H_a), 7.15-7.12 (m, 1H, H_b), 7.12-7.10 (m, 1H, H_c), 7.04 (d, *J* = 8.7, 2H, H_q), 6.82 (d, *J* = 8.7, 2H, H_p), 5.05-4.89 (m, 2H, NH, H_t), 4.46 (q, *J* = 6.4, 1H, H_s), 4.17-4.12 (m, 2H, H_d), 4.12-4.06 (m, 2H, H_o), 3.88-3.80 (m, 4H, H_e, H_n), 3.74-3.60 (m, 16H, H_f, H_g, H_h, H_i, H_j, H_k, H_l, H_m), 3.09-2.92 (m, 2H, H_r), 1.43 (s, 9H, H_v), 1.24-0.97 (m, 6H, H_u), 0.25 (s, 9H, H_w).

^{19}F NMR (376 MHz, $CDCl_3$, 298 K) δ: -63.27 (s, 3F, CF_3)

^{13}C NMR (101 MHz, $CDCl_3$, 298 K) δ 171.4, 158.7, 157.8, 155.1, 131.9 (q, J_{C-F} = 32.7), 130.4, 128.3, 125.1, 123.5 (q, J_{C-F} = 272.7), 121.1 (q, J_{C-F} = 3.8), 120.5, 114.6, 112.4 (q, J_{C-F} = 3.7), 103.3, 95.9, 79.6, 70.9, 70.8, 70.6, 70.6 (×3), 70.6 (×2), 69.7, 69.5, 69.0, 68.0, 67.4, 54.6, 37.4, 28.3, 21.8, 21.7, -0.2.

HR-ESI-MS *m/z* = 845.4223 $[M+NH_4]^+$ calc. 845.4226 for $C_{41}H_{64}F_3N_2O_{11}Si$.

$[\alpha]_D^{23}$ +7.2 (*c* 0.89, $CHCl_3$)

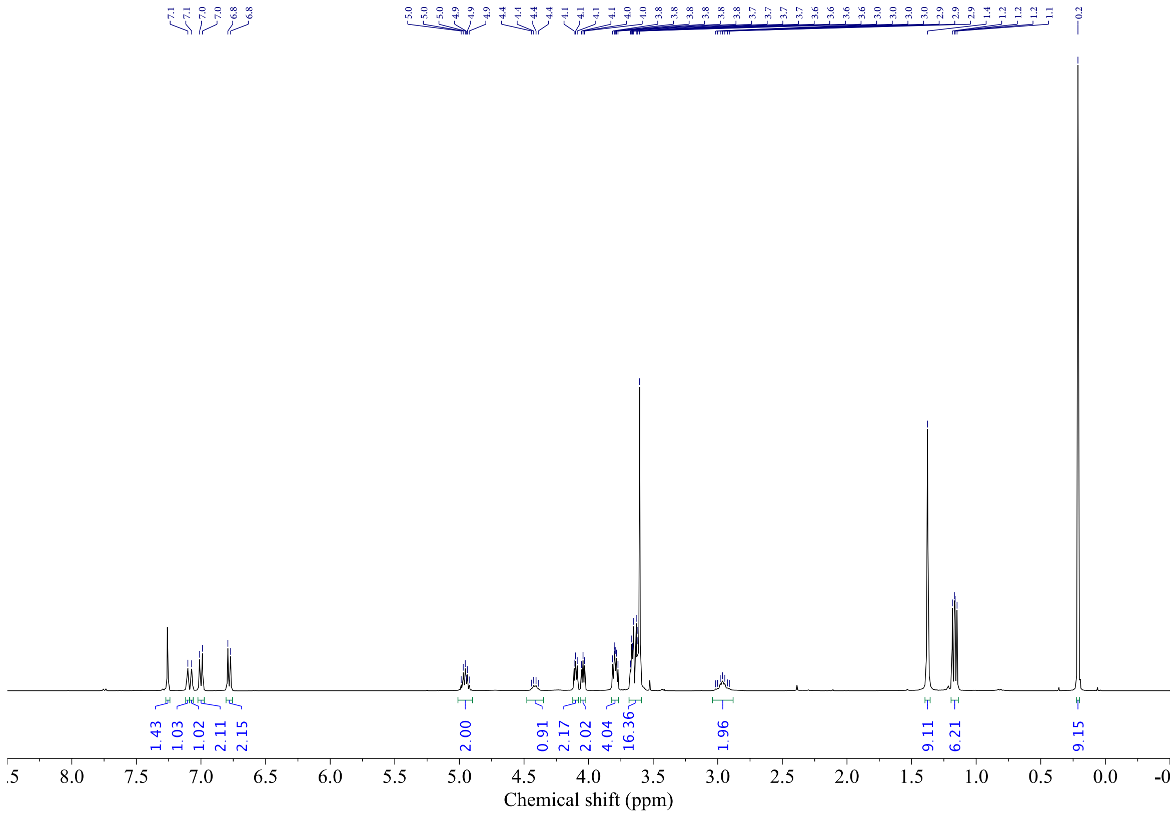

Figure S48. ^{1}H NMR of (*S*)-**S6b** ($CDCl_3$, 400 MHz, 298 K)

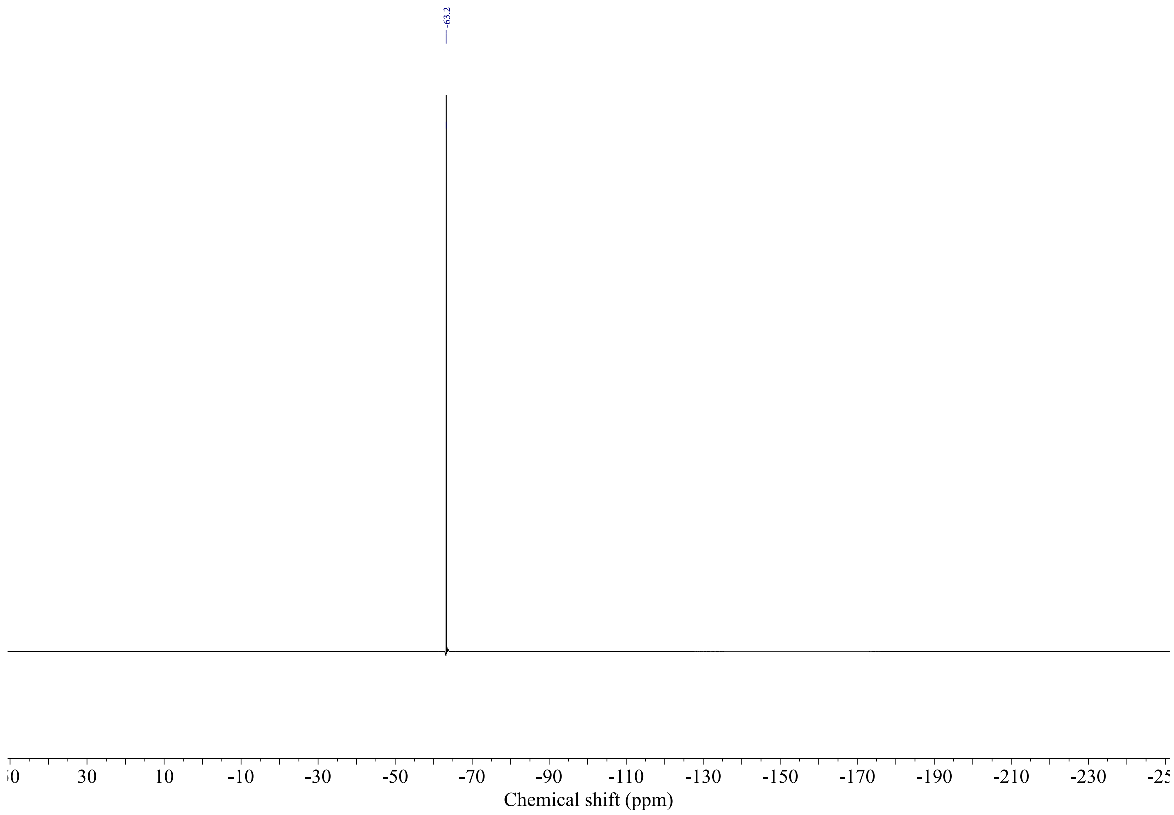

Figure S49. ^{19}F NMR of (*S*)-**S6b** ($CDCl_3$, 376 MHz, 298 K)

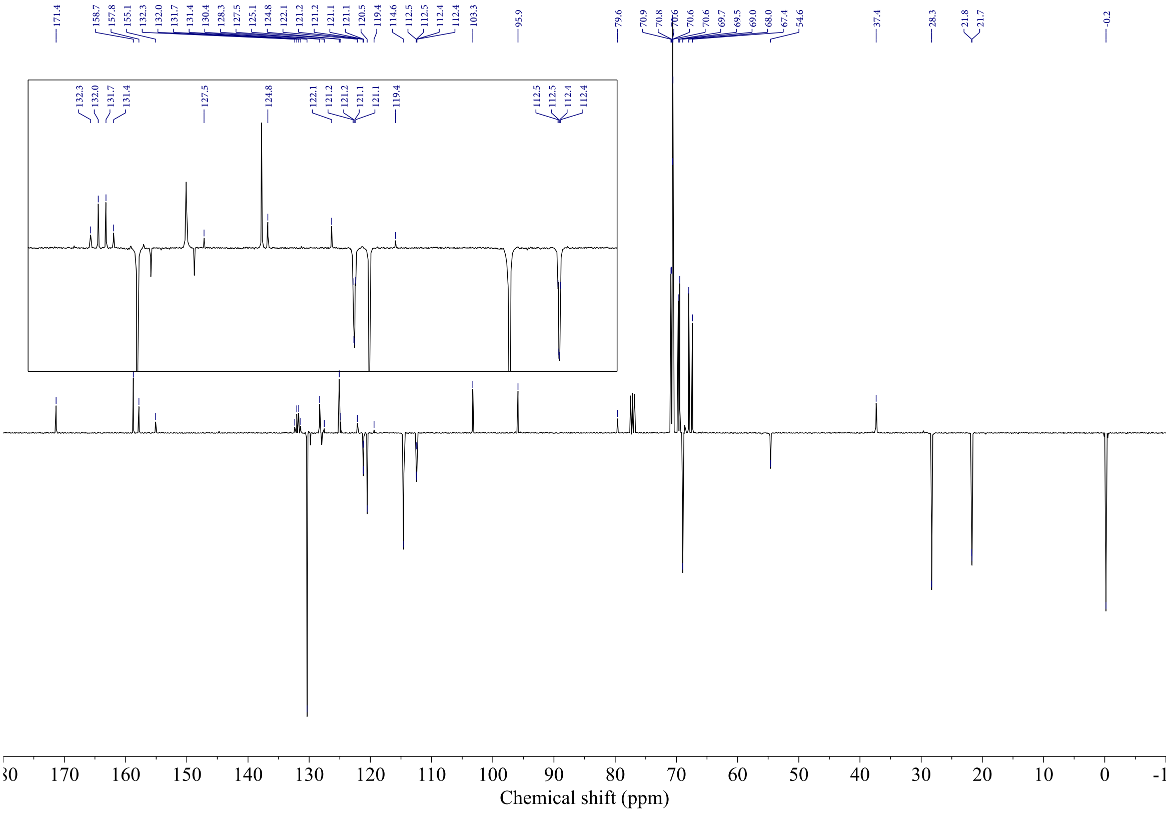

Figure S50. JMOD NMR of (*S*)-**S6b** ($CDCl_3$, 101 MHz, 298 K)

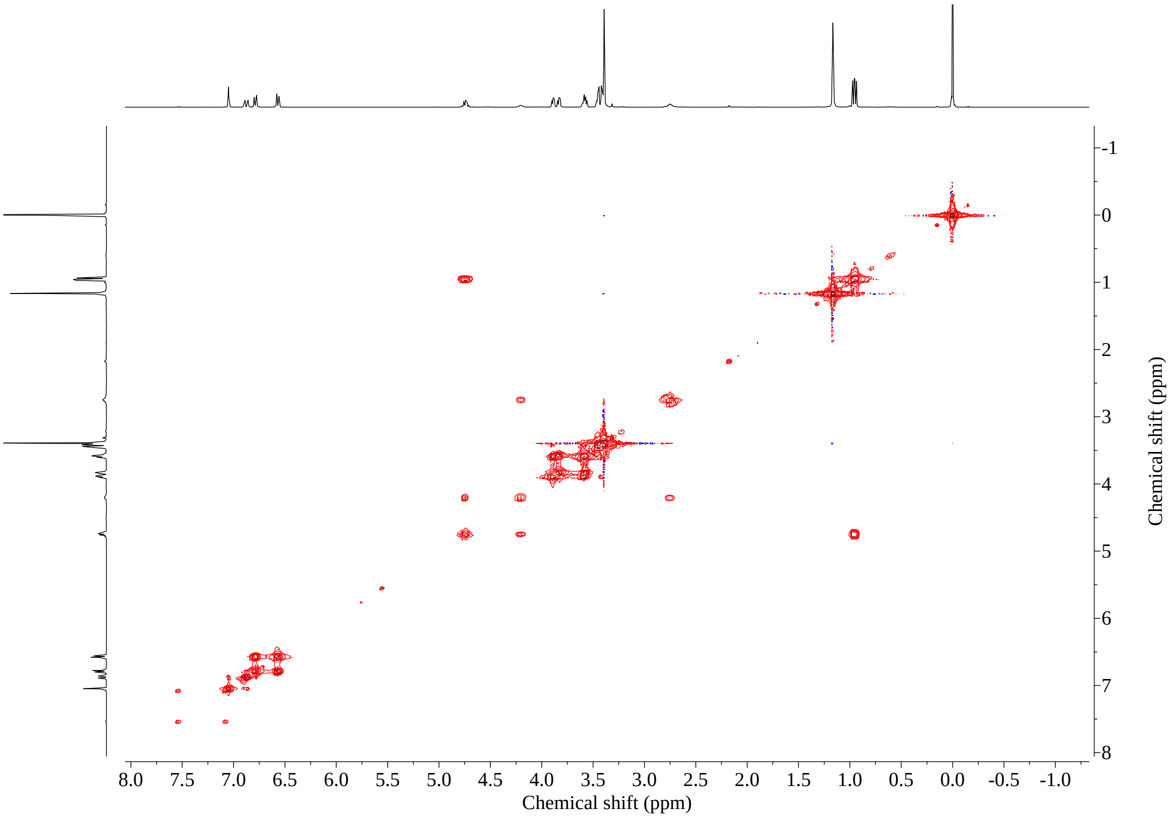

Figure S51. COSY NMR of (*S*)-**S6b** ($CDCl_3$, 298 K)

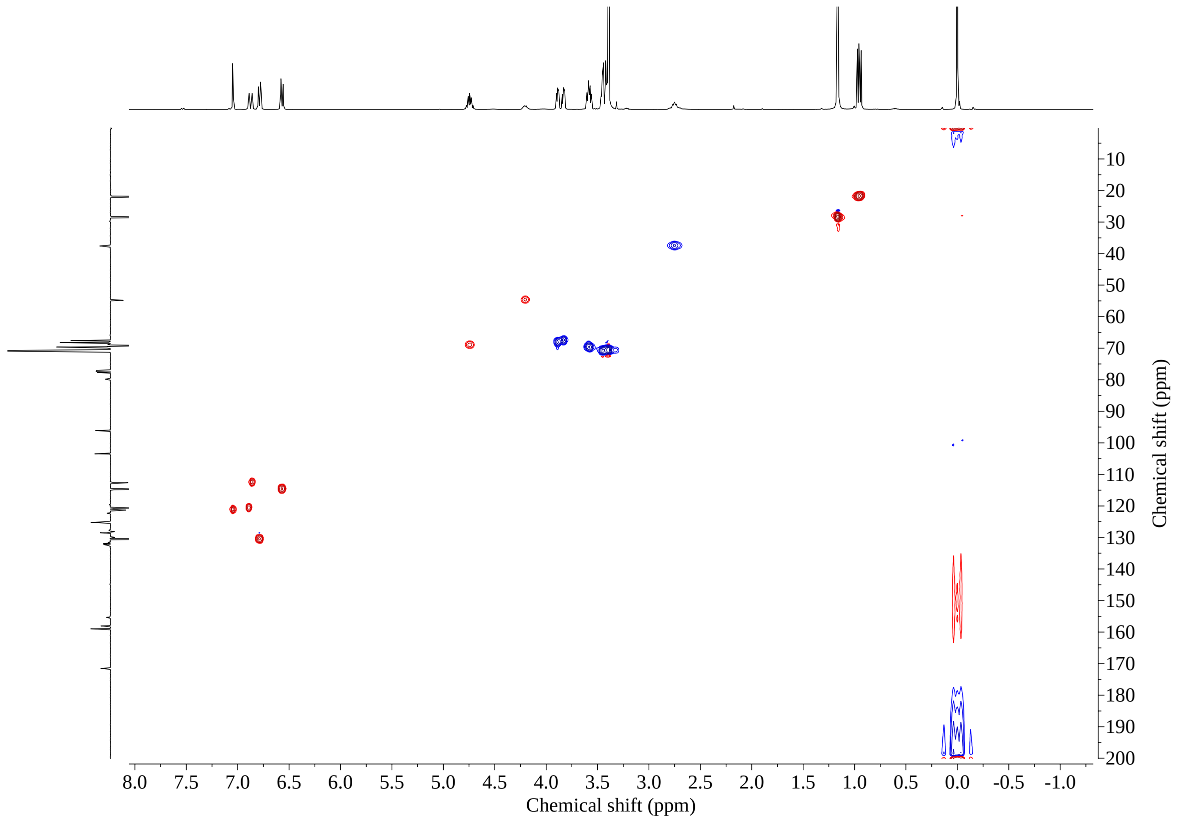

Figure S52. HSQC NMR of (*S*)-**S6b** ($CDCl_3$, 298 K)

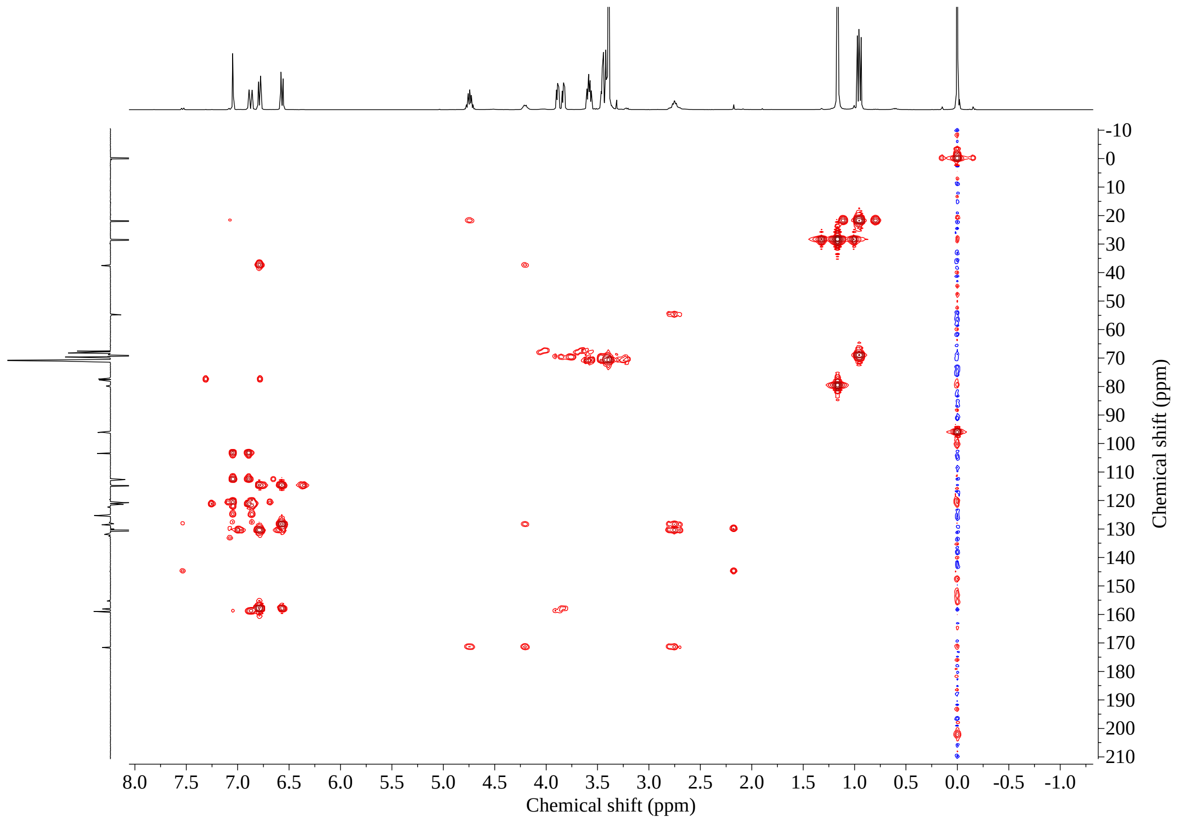

Figure S53. HMBC NMR of (*S*)-**S6b** ($CDCl_3$, 298 K)

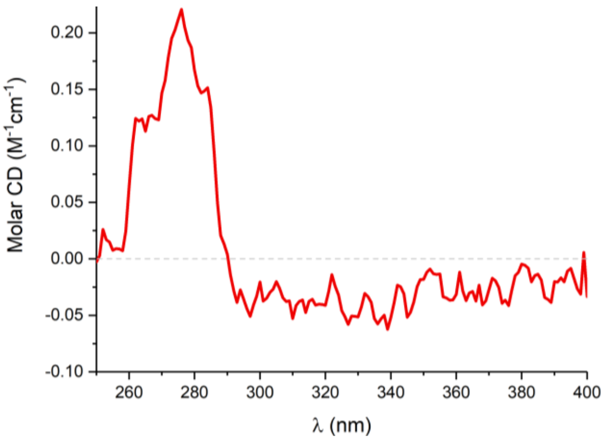

Figure S54. Circular Dichroism Spectra of (*S*)-**S6b** (74.5 µM) at 293 K in $CHCl_3$

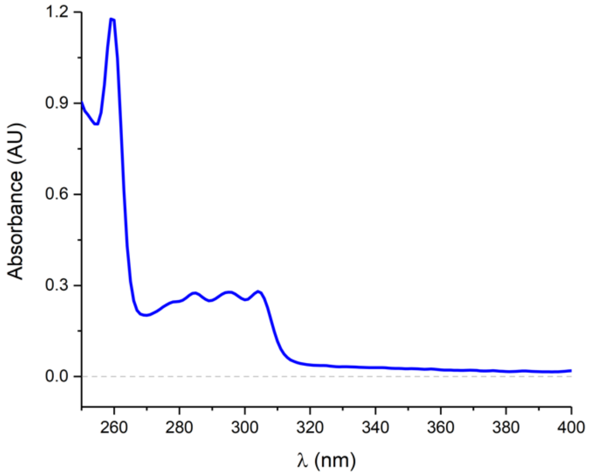

Figure S55. UV-Vis Spectra of (*S*)-**S6b** (74.5 µM) at 293 K in $CHCl_3$

Terminal acetylene (*S*)-S7b

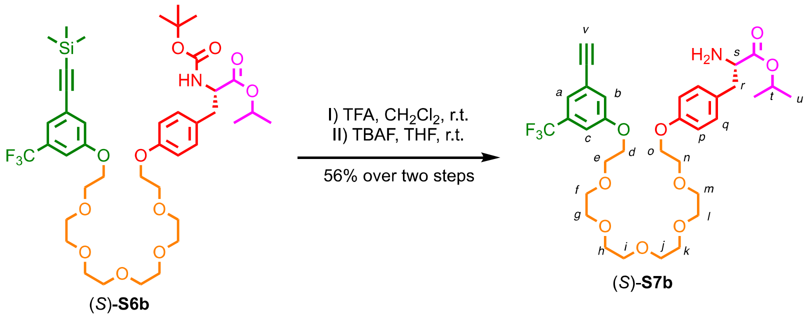

(*S*)-**S6b** (500 mg, 0.60 mmol, 1.0 equiv.) was dissolved in CH_2Cl_2 (5 mL). Trifluoroacetic acid (0.5 mL, 6 mmol, 10.0 equiv.) was added dropwise to the solution and the mixture was stirred at rt for 16 h. The reaction mixture was quenched with sat. $NaHCO_3$ (10 mL), extracted with CH_2Cl_2 (20 mL) and the organic phase dried ($MgSO_4$). The solvent was removed *in vacuo*. The crude was re-dissolved in THF (5 mL), tetrabutylammonium fluoride (1 M in THF, 1.2 mL, 1.2 mmol, 2.0 equiv.) was added and the reaction mixture was stirred at rt for 4 h. The reaction mixture was partitioned between H_2O (20 mL) and EtOAc (30 mL), the phases separated and the organic layer dried ($MgSO_4$). The solvent was removed *in vacuo* and the crude was purified by column chromatography (deactivated SiO_2, CH_2Cl_2-acetone 0 → 20%) to give (*S*)-**S7b** as an orange oil (219 mg, 56% over 2 steps).

^{1}H NMR (400 MHz, $CDCl_3$, 298 K) δ 7.35-7.30 (m, 1H, H_a), 7.19-7.16 (m, 1H, H_b), 7.16-7.13 (m, 1H, H_c), 7.10 (d, *J* = 8.8, 2H, H_q), 6.84 (d, *J* = 8.9, 2H, H_p), 5.01 (sept., *J* = 6.3, 1H, H_t), 4.17-4.03 (m, 4H, H_d, H_o), 3.89-3.79 (m, 4H, H_e, H_n), 3.76-3.58, (m, 17H, H_f, H_g, H_h, H_i, H_j, H_k, H_l, H_m, H_s), 3.12 (s, 1H, H_v) 3.05-2.97 (m, 1H, H_r), 1.86 (br s, 2H, NH), 2.88-2.82 (m, 1H, H_r), 1.25-17(m, 6H, H_u).

^{19}F NMR (376 MHz, $CDCl_3$, 298 K) δ: -63.26 (s, 3F, CF_3)

^{13}C NMR (101 MHz, $CDCl_3$, 298 K) δ 174.5, 158.8, 157.7, 132.0 (q, $J_{C\text{-}F}$ = 32.8), 130.3, 129.4, 124.1, 123.4 (q, $J_{C\text{-}F}$ = 272.7), 121.3 (q, $J_{C\text{-}F}$ = 3.8), 121.0, 114.6, 112.6 (q, $J_{C\text{-}F}$ = 3.7), 82.0, 78.7, 70.9, 70.8, 70.6, 70.6 (×3), 70.6 (×2), 69.7, 69.5, 68.4, 68.0, 67.4, 55.9, 40.1, 21.8, 21.7.

HR-ESI-MS *m/z* = 656.3056 $[M+H]^+$ calc. 656.3041 for $C_{33}H_{45}F_3NO_9$.

$[\alpha]_D^{23}$ −4.5 (*c* 0.79, $CHCl_3$)

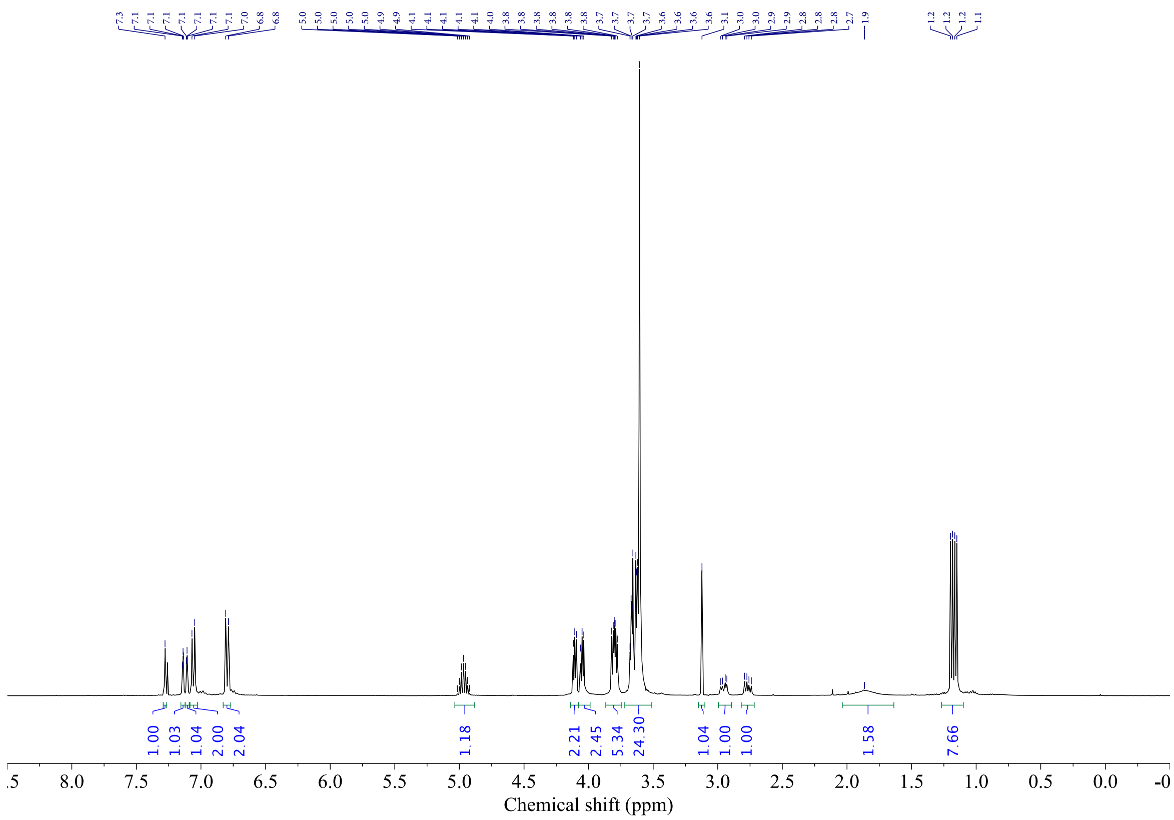

Figure S56. ^{1}H NMR of (*S*)-**S7b** ($CDCl_3$, 400 MHz, 298 K)

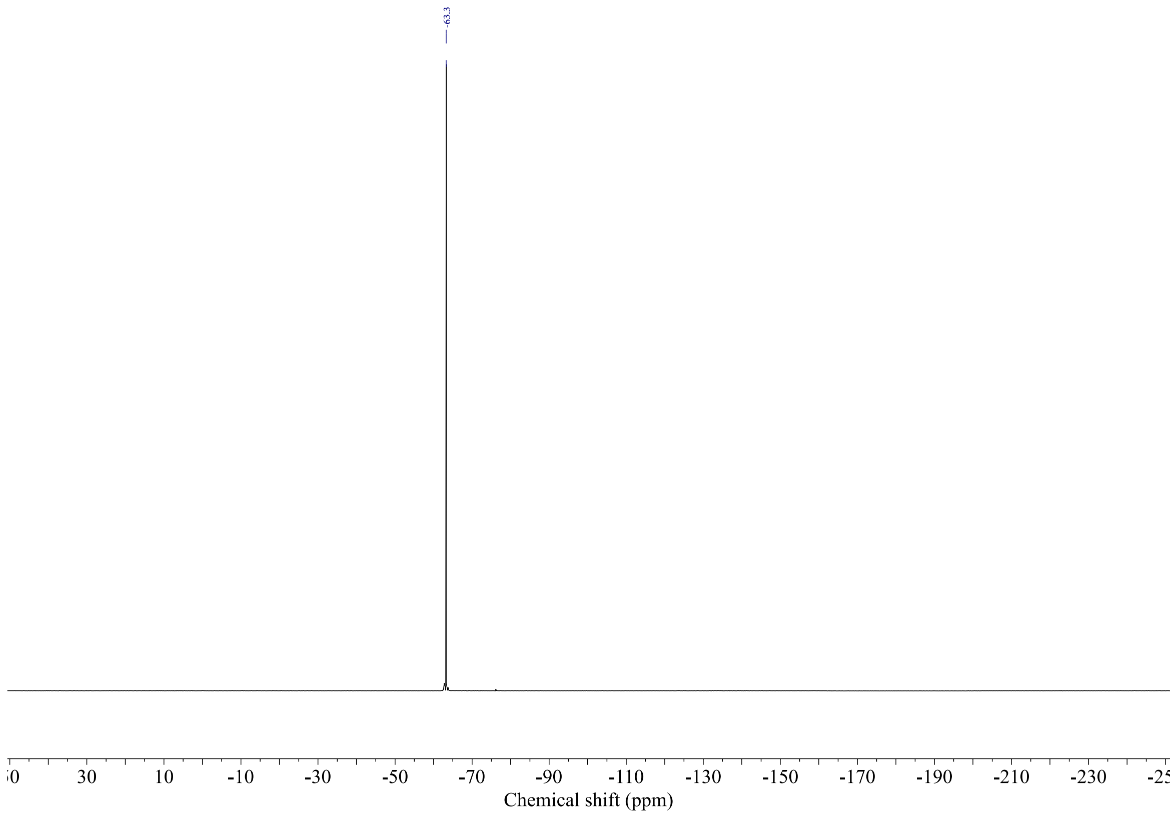

Figure S57. ^{19}F NMR of (*S*)-**S7b** ($CDCl_3$, 376 MHz, 298 K)

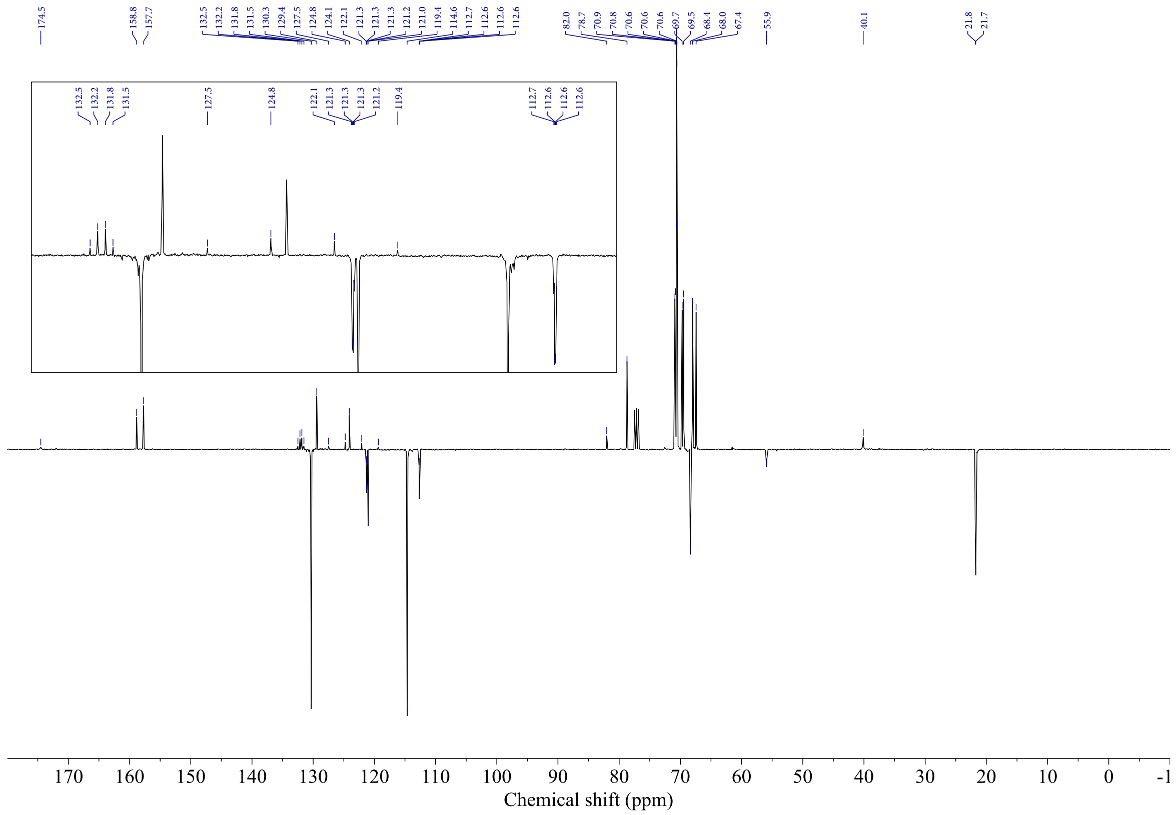

Figure S58. JMOD NMR of (*S*)-**S7b** ($CDCl_3$, 101 MHz, 298 K)

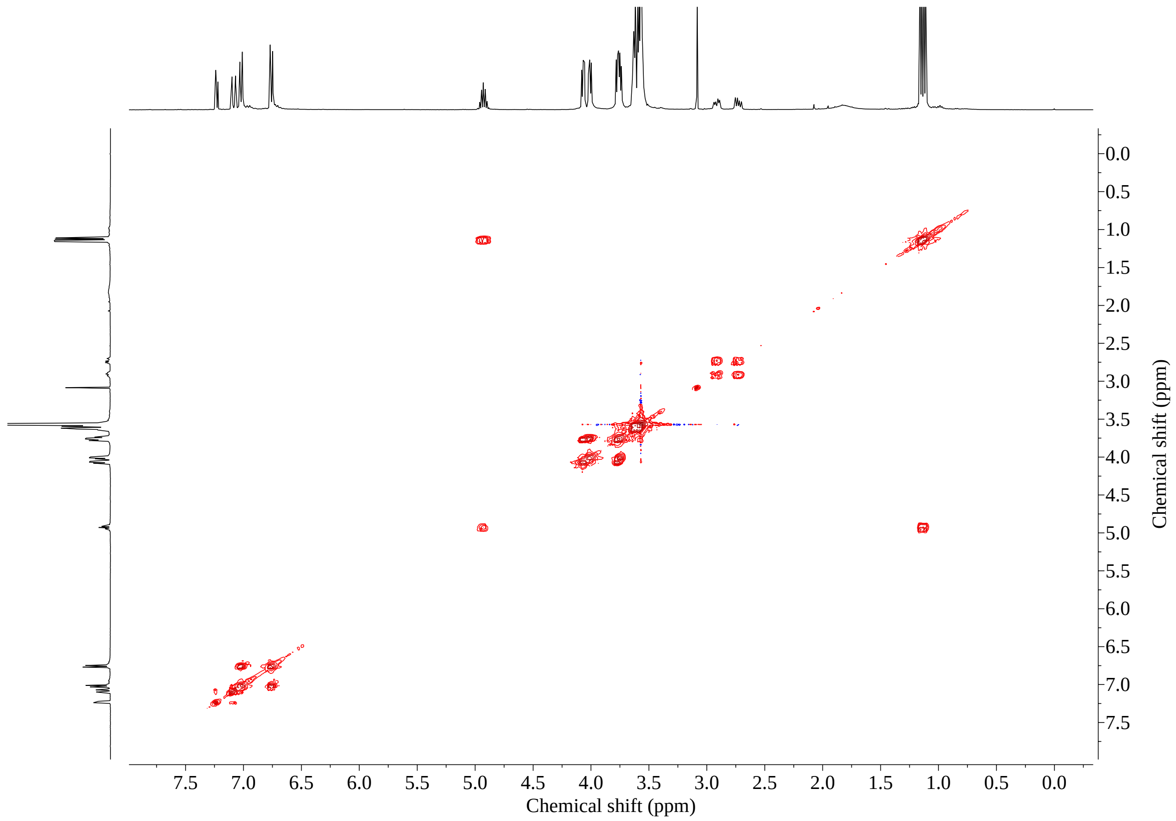

Figure S59. COSY NMR of (*S*)-**S7b** ($CDCl_3$, 298 K))

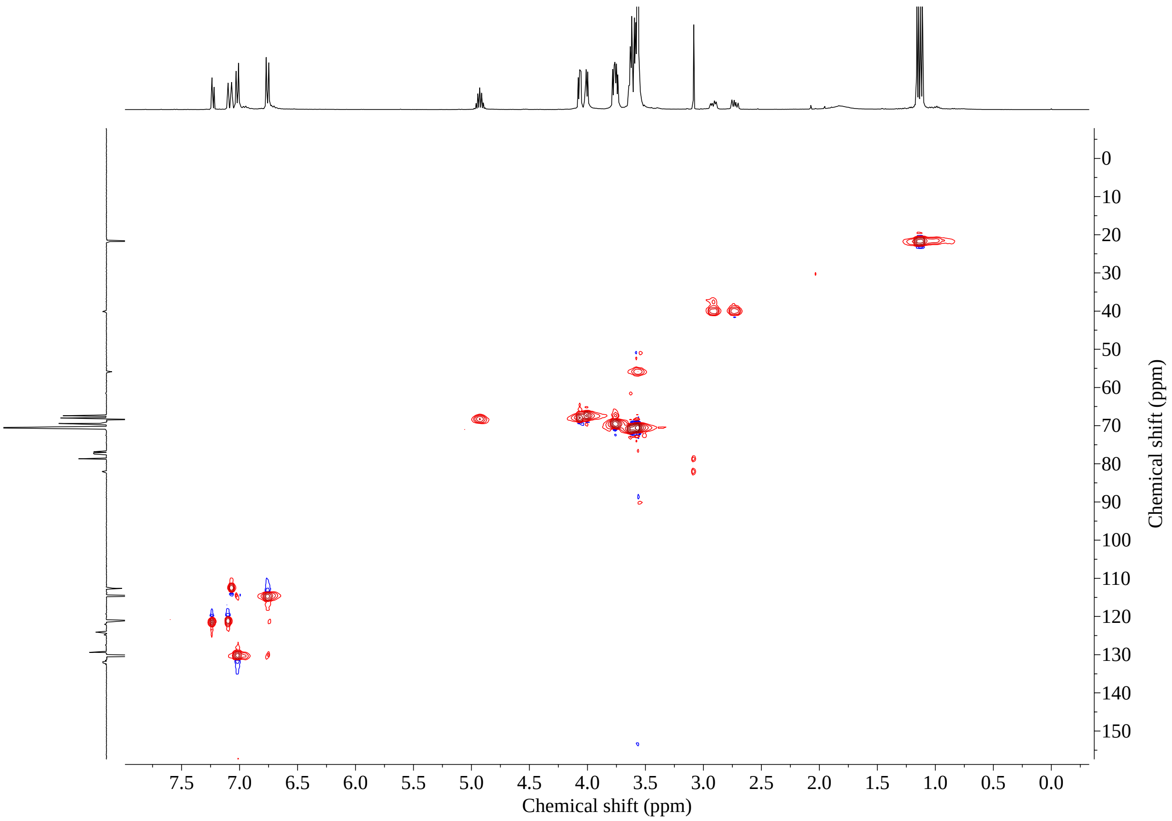

Figure S60. HSQC NMR of (*S*)-**S7b** ($CDCl_3$, 298 K)

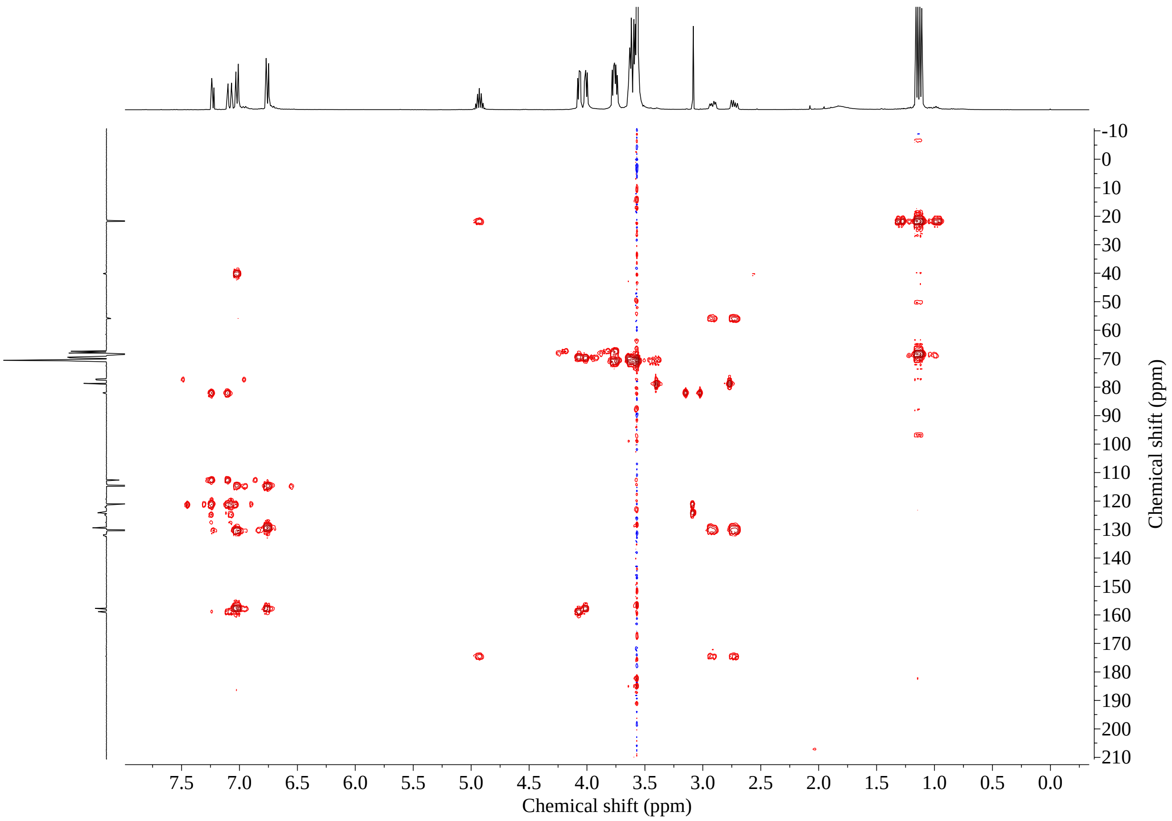

Figure S61. HMBC NMR of (*S*)-**S7b** ($CDCl_3$, 298 K)

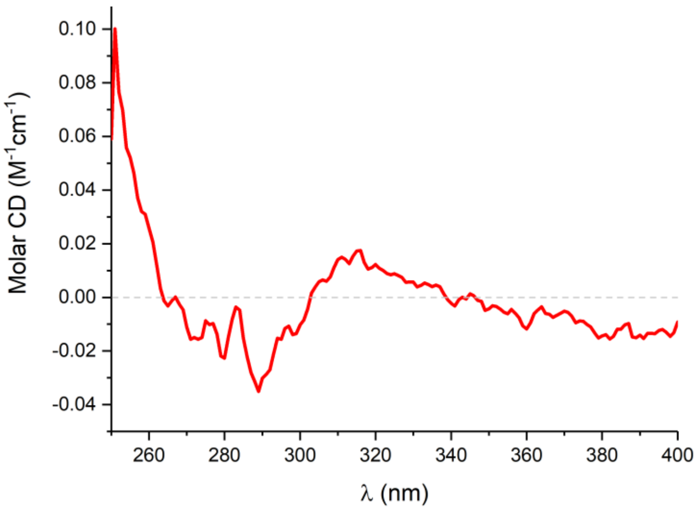

Figure S62. Circular Dichroism Spectra of (*S*)-**S7b** (133 μM) at 293 K in $CHCl_3$

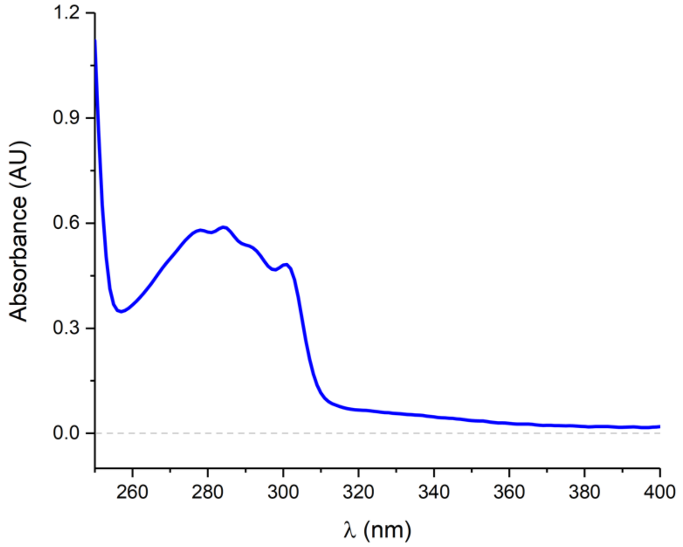

Figure S63. UV-Vis Spectra of (*S*)-**S7b** (133 μM) at 293 K in $CHCl_3$

Macrocycle precursor (*S*)-1b

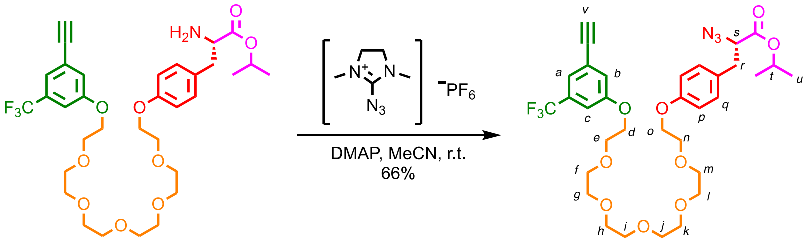

(*S*)-**S7b** (67 mg, 0.10 mmol, 1.0 equiv.), DMAP (37 mg, 0.30 mmol, 3.0 equiv.) and ADMP (44 mg, 0.15 mmol, 1.5 equiv.) were dissolved in CH_3CN (1.0 mL). The mixture was stirred at rt for 16 h before quenching with H_2O (10 mL). The mixture was extracted with CH_2Cl_2 (20 mL) and the organic extract dried ($MgSO_4$). The solvent was removed *in vacuo* and the crude was purified by column chromatography (CH_2Cl_2-Et_2O 100 : 0 → 80 : 20) to obtain (*S*)-**1b** as a colorless oil (45 mg, 66% yield).

^{1}H NMR (400 MHz, $CDCl_3$, 298 K) δ 7.32-7.30 (m, 1H, H_a), 7.18-7.16 (m, 1H, H_b), 7.15-7.08 (m, 3H, H_c, H_q), 6.84 (d, *J* = 8.7, 2H, H_p), 5.05 (sept, *J* = 6.3, 1H, H_t), 4.18-4.11 (m, 2H H_d), 4.11-4.04 (m, 2H, H_o), 3.94 (dd, *J* = 8.4, 5.7, 1H, H_s), 3.87-3.75 (m, 4H, H_e, H_n), 3.76-3.54 (m, 16H, H_f, H_g, H_h, H_i, H_j, H_k, H_l, H_m), 3.13 (s, 1H, H_v), 3.07 (dd, *J* = 14.1, 5.7, 1H, H_r), 2.93 (dd, *J* = 14.1, 8.4, 1H, $H_{r'}$), 1.25 (d, *J* = 6.3, 3H, H_u), 1.21 (d, *J* = 6.2, 3H, $H_{u'}$).

^{19}F NMR (376 MHz, $CDCl_3$, 298 K) δ: -63.27 (s, 3F, CF_3).

^{13}C NMR (101 MHz, $CDCl_3$, 298 K) δ 169.5, 158.9, 158.1, 132.1 (q, J_{C-F} = 32.8), 130.3, 128.2, 124.1, 123.5 (q, J_{C-F} = 272.6), 121.4 (q, J_{C-F} = 3.9), 121.1, 114.8, 112.7 (q, J_{C-F} = 3.8), 82.1, 78.7, 71.0, 70.9, 70.7, 70.7 (×3), 70.6 (×2), 69.8, 69.8, 69.5, 68.1, 67.5, 63.5, 36.8, 21.8, 21.7.

HR-ESI-MS *m/z* = 704.2769 $[M+Na]^+$ calc. 704.2765 for $C_{33}H_{42}F_3N_3NaO_9$.

$[\alpha]_D^{23}$ −10.5 (*c* 0.72, $CHCl_3$)

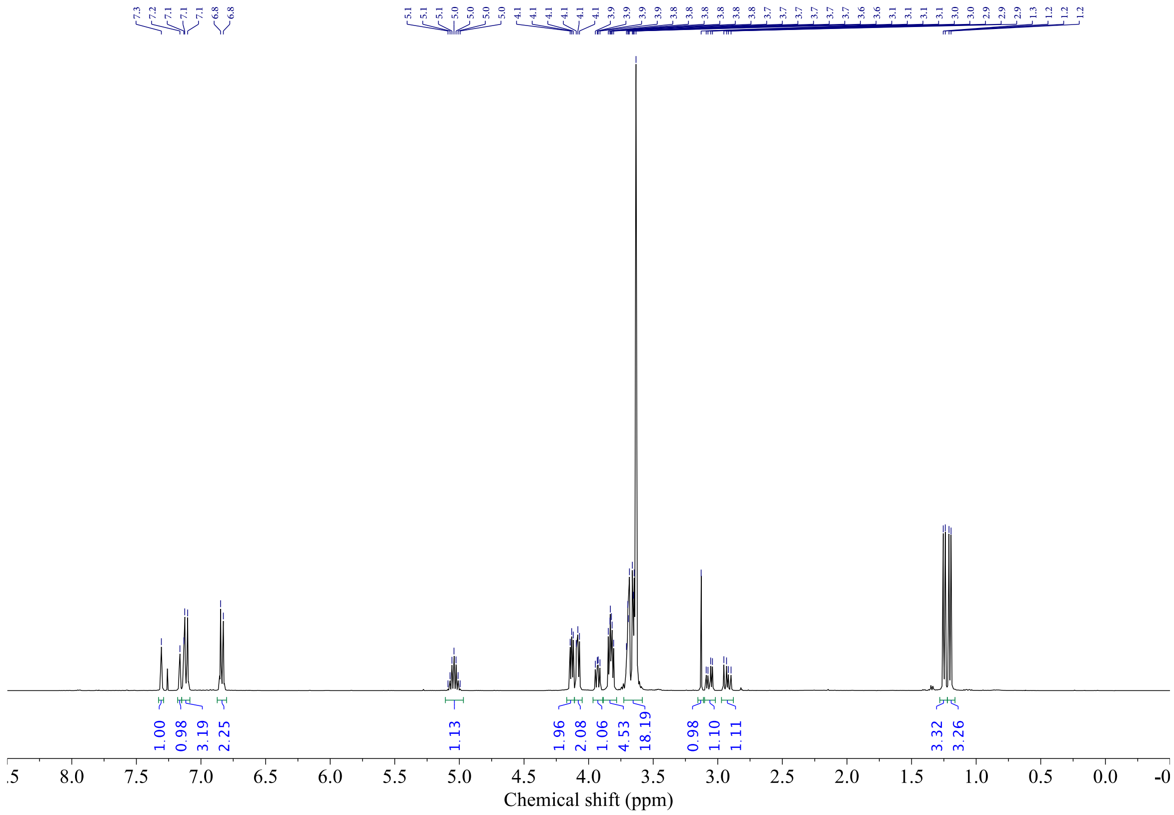

Figure S64. ^{1}H NMR of (*S*)-**1b** ($CDCl_3$, 400 MHz, 298 K)

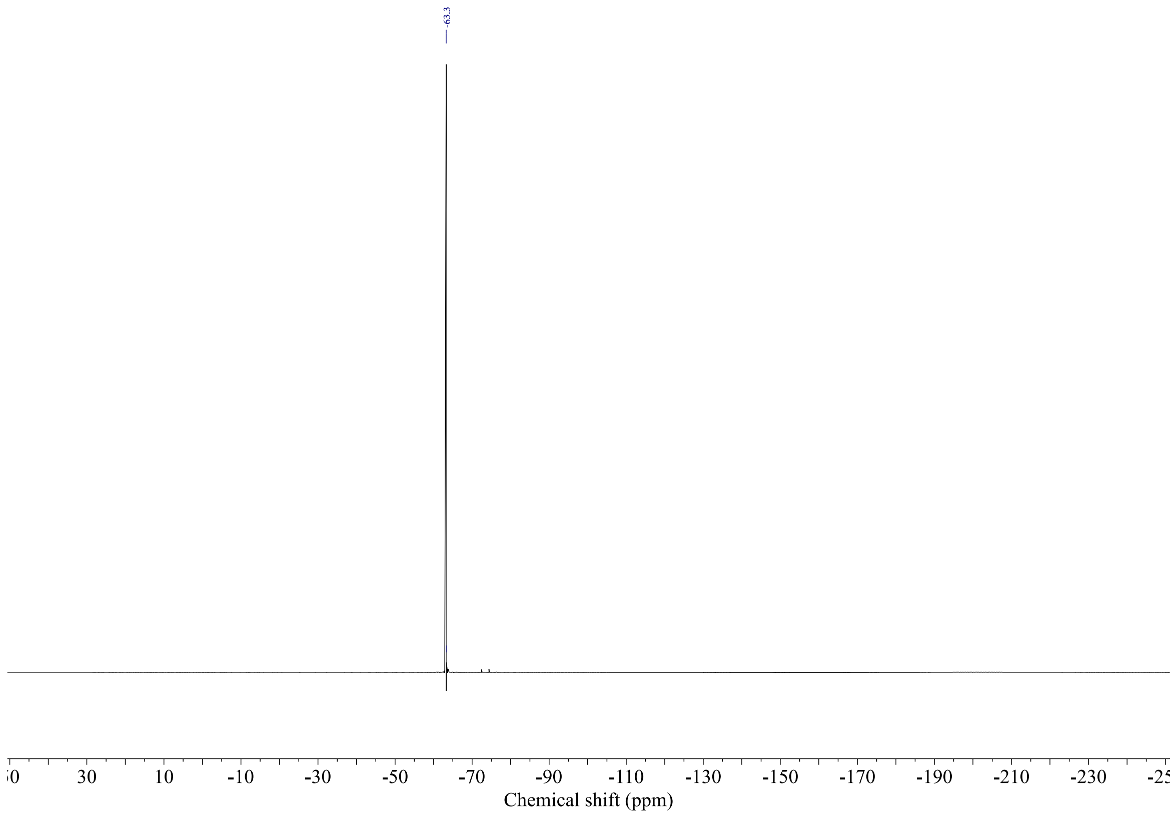

Figure S65. ^{19}F NMR of (*S*)-**1b** ($CDCl_3$, 376 MHz, 298 K)

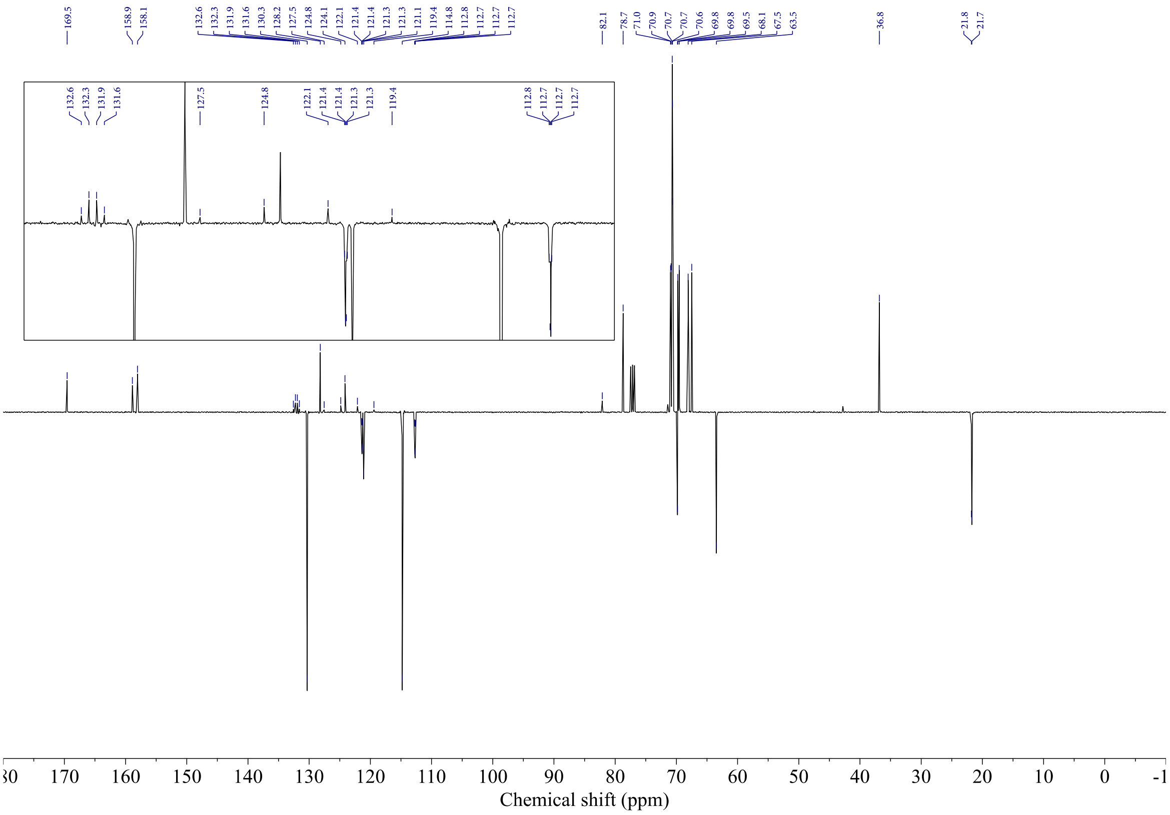

Figure S66. JMOD NMR of (*S*)-**1b** ($CDCl_3$, 101 MHz, 298 K)

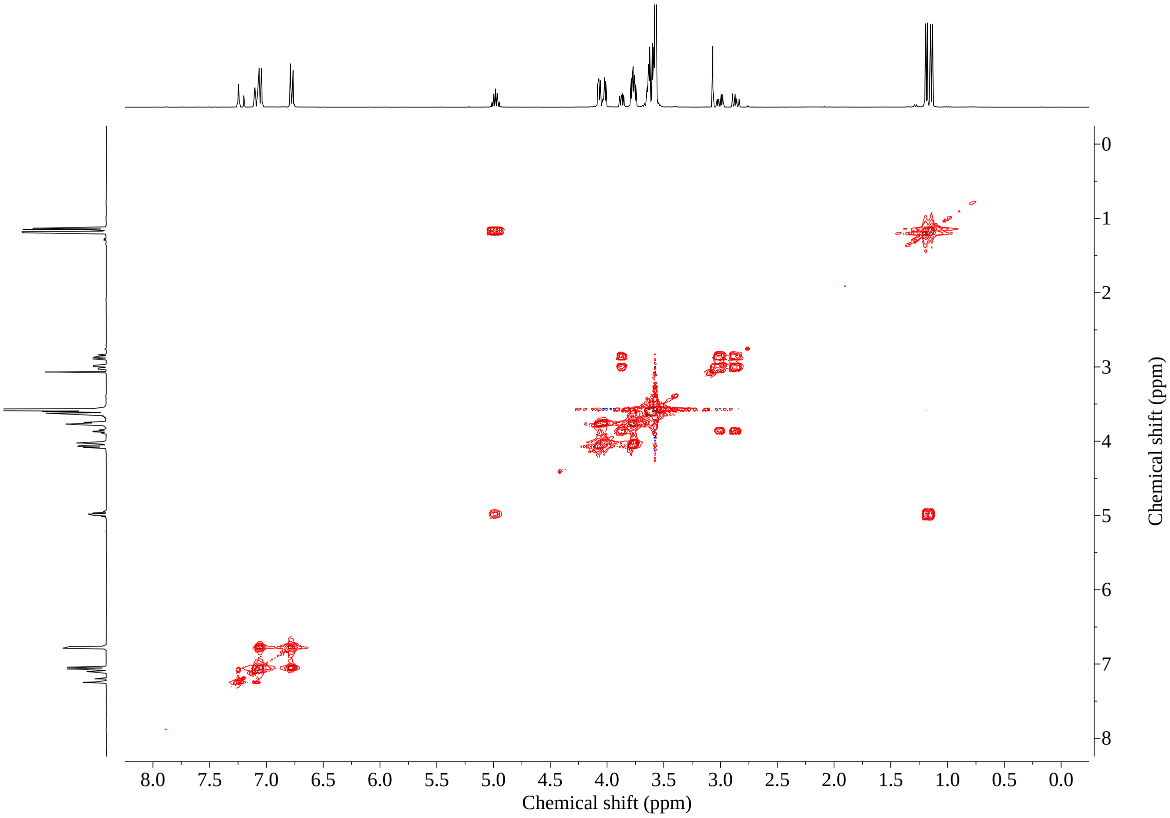

Figure S67. COSY NMR of (*S*)-**1b** ($CDCl_3$, 298 K)

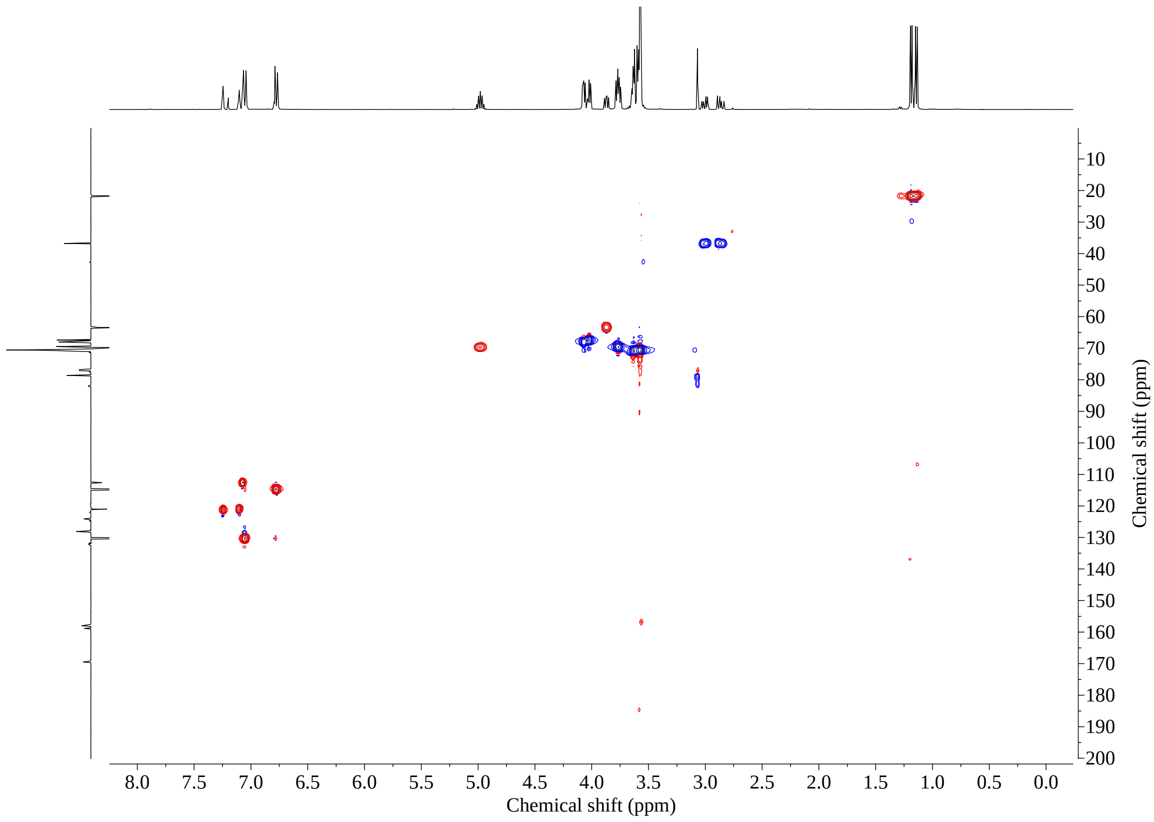

Figure S68. HSQC NMR of (*S*)-**1b** ($CDCl_3$, 298 K)

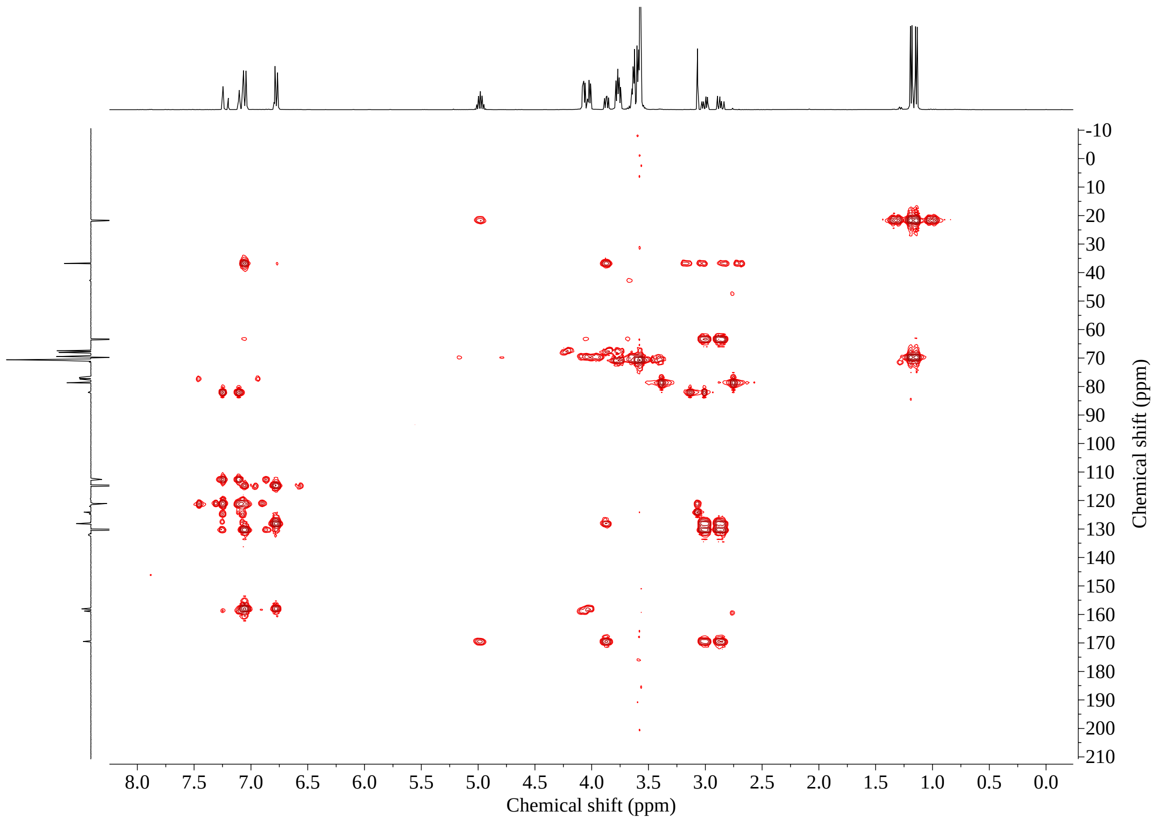

Figure S69. HMBC NMR of (*S*)-**1b** ($CDCl_3$, 298 K)

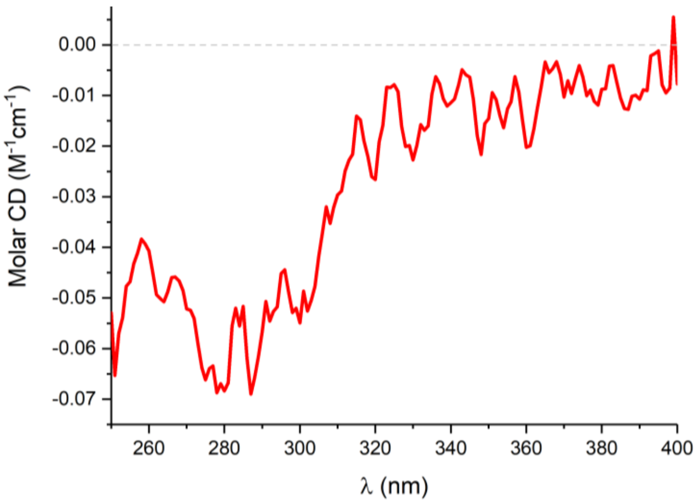

Figure S70. Circular Dichroism Spectra of (*S*)-**1b** (217 μM) at 293 K in $CHCl_3$

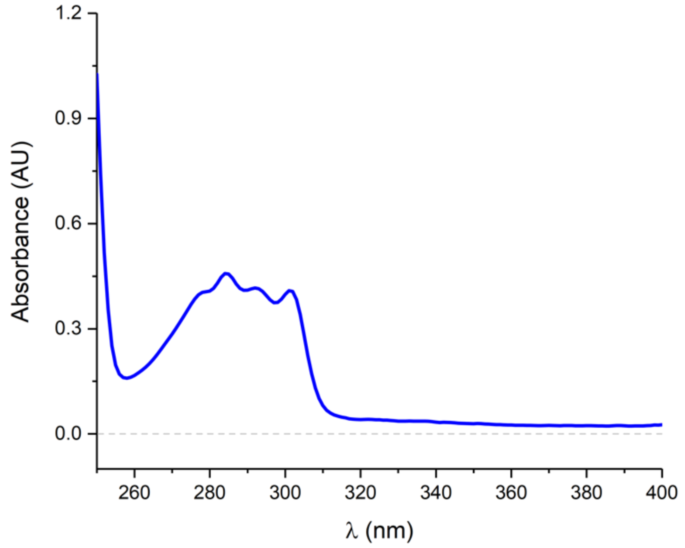

Figure S71. UV-Vis Spectra of (*S*)-**1b** (217 μM) at 293 K in $CHCl_3$

Boc amine (*S*)-S5c

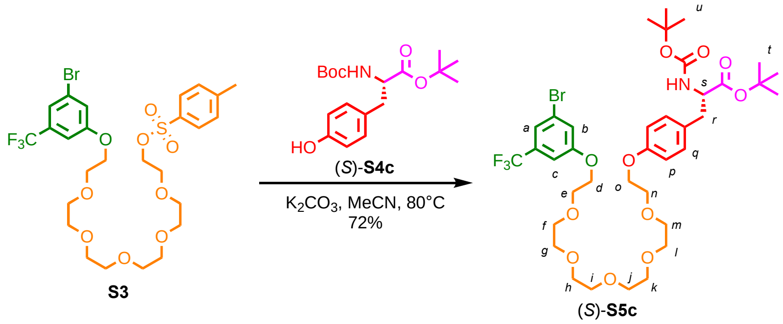

S3 (500 mg, 0.76 mmol, 1.0 equiv.), (*S*)-**S4c** (256 mg, 0.76 mmol, 1.0 equiv.) and K_2CO_3 (420 mg, 3.04 mmol, 4.0 equiv.) were suspended in CH_3CN (8 mL) and the resulting suspension was heated at reflux for 48 h. The reaction mixture was filtered over a Celite® pad, which was washed with CH_2Cl_2 (20 mL). The washings were combined, and the solvent was removed *in vacuo*. The crude was purified by column chromatography (petrol-EtOAc 75 : 25 → 25 : 75) to give (*S*)-**S5c** as a colorless oil (451 mg, 72%).

^{1}H NMR (400 MHz, $CDCl_3$, 298 K) δ 7.35-7.30 (m, 1H, H_a), 7.25-7.23 (m, 1H, H_b), 7.11-7.08 (m, 1H, H_c), 7.06 (d, *J* = 8.2, 2H, H_q), 6.82 (d, *J* = 8.4, 2H, H_p), 4.95 (d, *J* = 8.3, 1H, NH), 4.39 (q, *J* = 8.3, 1H, H_s), 4.17-4.12 (m, 2H, H_d), 4.11-4.05 (m, 2H, H_o), 3.89-3.79 (m, 4H, H_e, H_n), 3.76-3.60 (m, 16H, H_f, H_g, H_h, H_i, H_j, H_k, H_l, H_m), 3.02-2.94 (m, 2H, H_r), 1.42-1.38 (m, 18H, H_u, H_t).

^{19}F NMR (376 MHz, $CDCl_3$, 298 K) δ: -63.16 (s, 3F, CF_3)

^{13}C NMR (101 MHz, $CDCl_3$, 298 K) δ 171.1, 159.8, 157.9, 155.2, 133.1 (q, J_{C-F} = 32.9), 130.6, 128.7, 123.2, 123.2 (app. d, J_{C-F} = 273.0), 120.9 (q, J_{C-F} = 3.9), 114.6, 111.0 (q, J_{C-F} = 3.8), 82.1, 79.7, 71.1, 70.9, 70.8, 70.8 (×3), 70.7 (×2), 69.9, 69.6, 68.3, 67.5, 55.1, 37.7, 28.5, 28.1.

HR-ESI-MS *m/z* = 846.2653 $[M+Na]^+$ calc. 846.2646 for $C_{37}H_{53}BrF_3NNaO_{11}$.

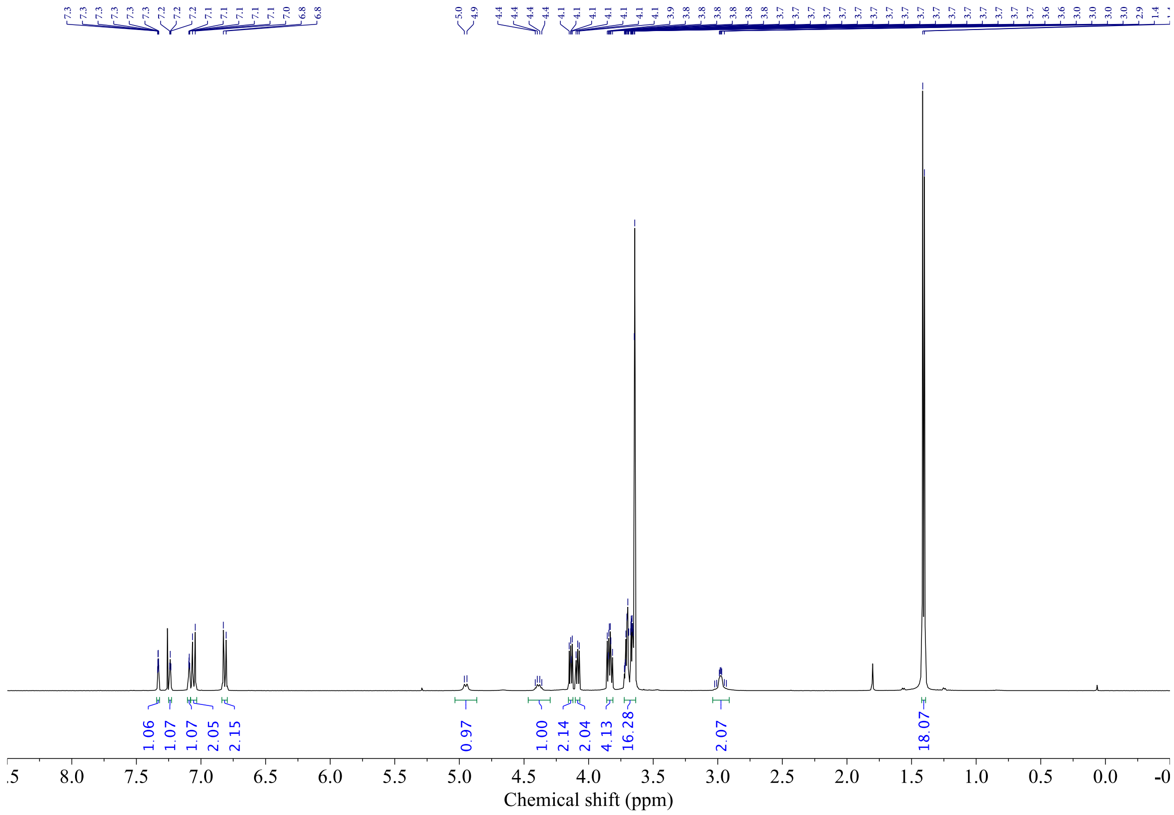

Figure S72. ^{1}H NMR of (*S*)-**S5c** ($CDCl_3$, 400 MHz, 298 K)

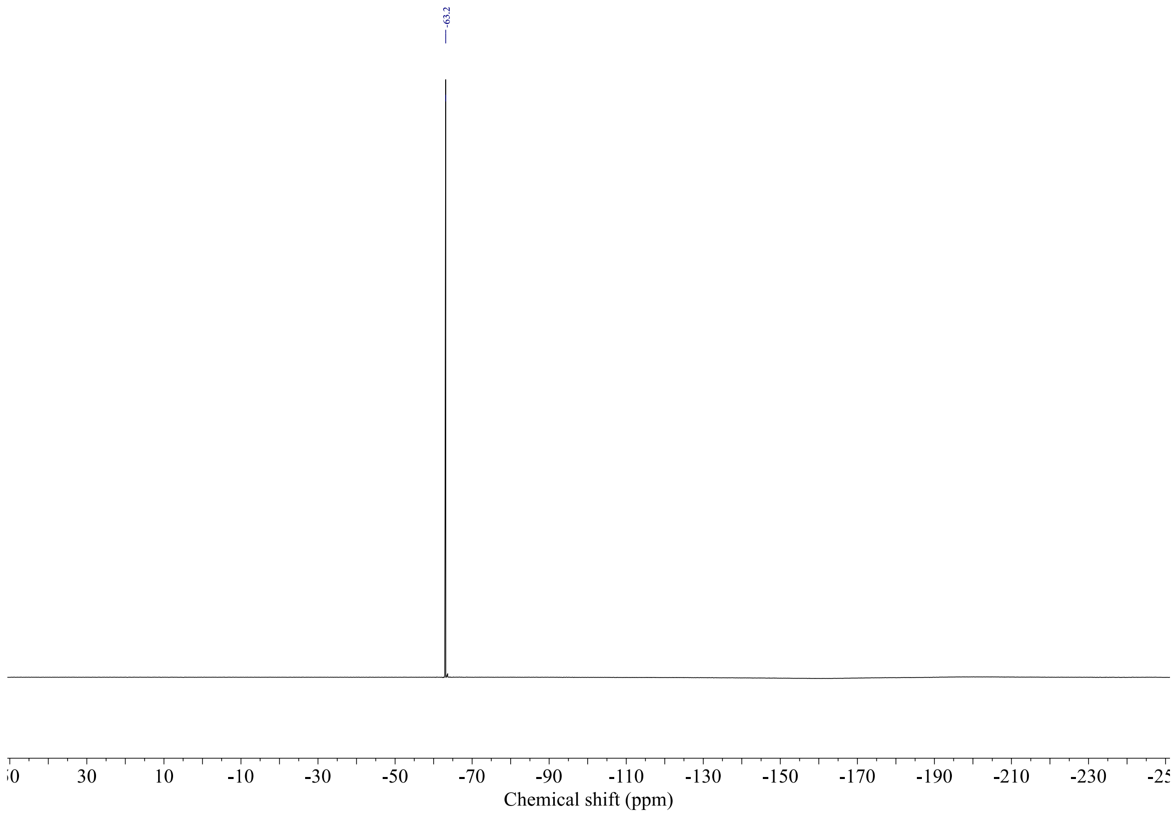

Figure S73. ^{19}F NMR of (*S*)-**S5c** ($CDCl_3$, 376 MHz, 298 K)

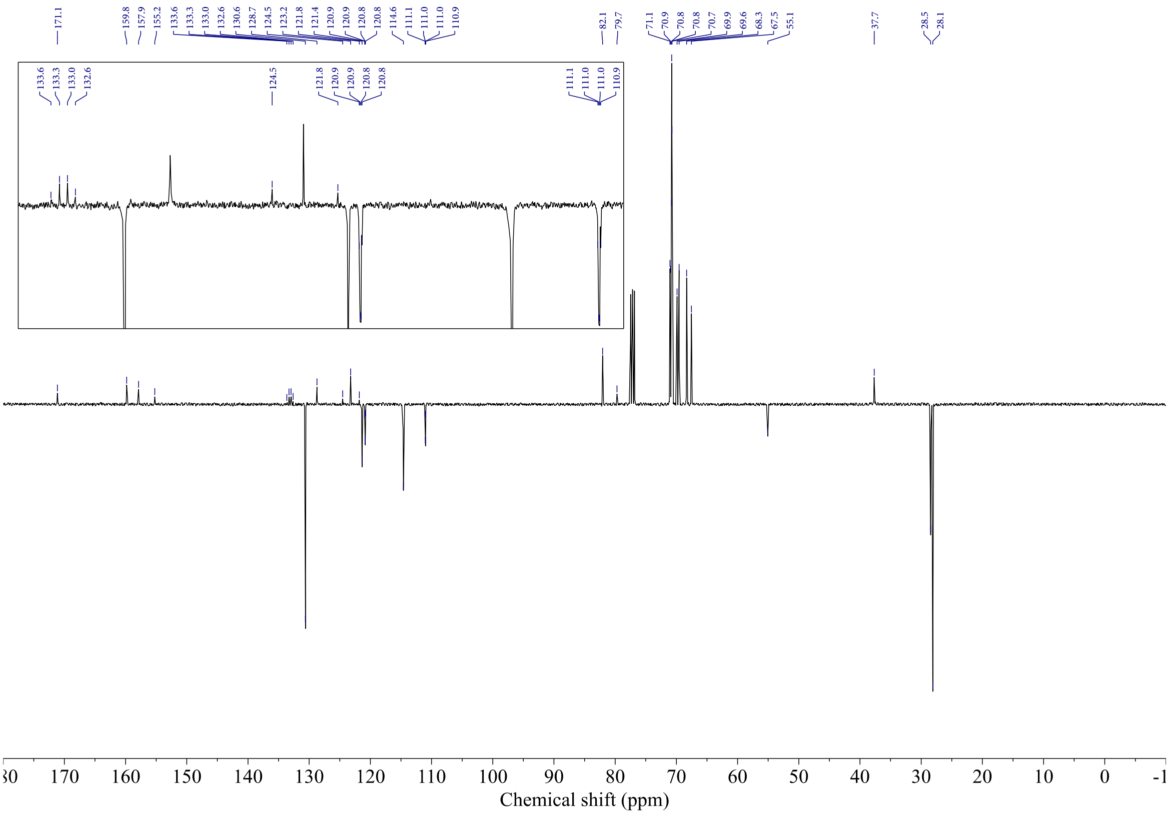

Figure S74. JMOD NMR of (*S*)-**S5c** ($CDCl_3$, 101 MHz, 298 K)

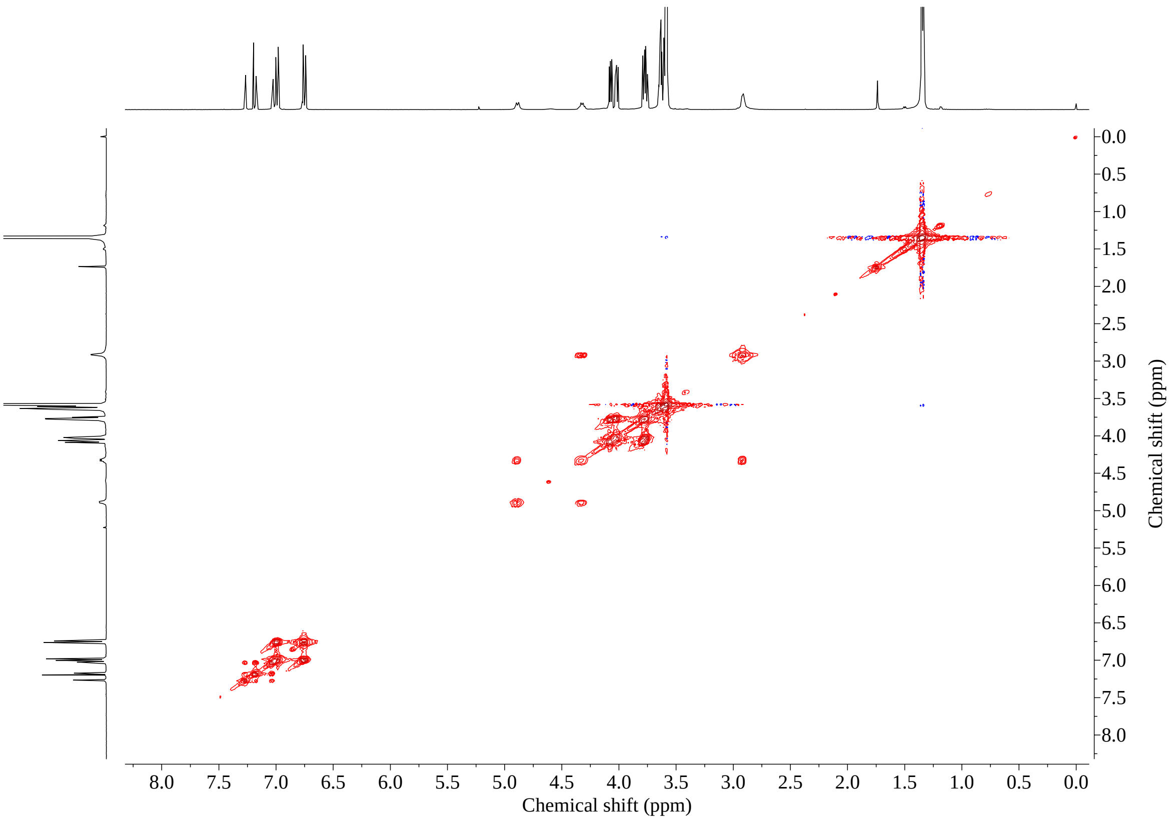

Figure S75. COSY NMR of (*S*)-**S5c** ($CDCl_3$, 298 K)

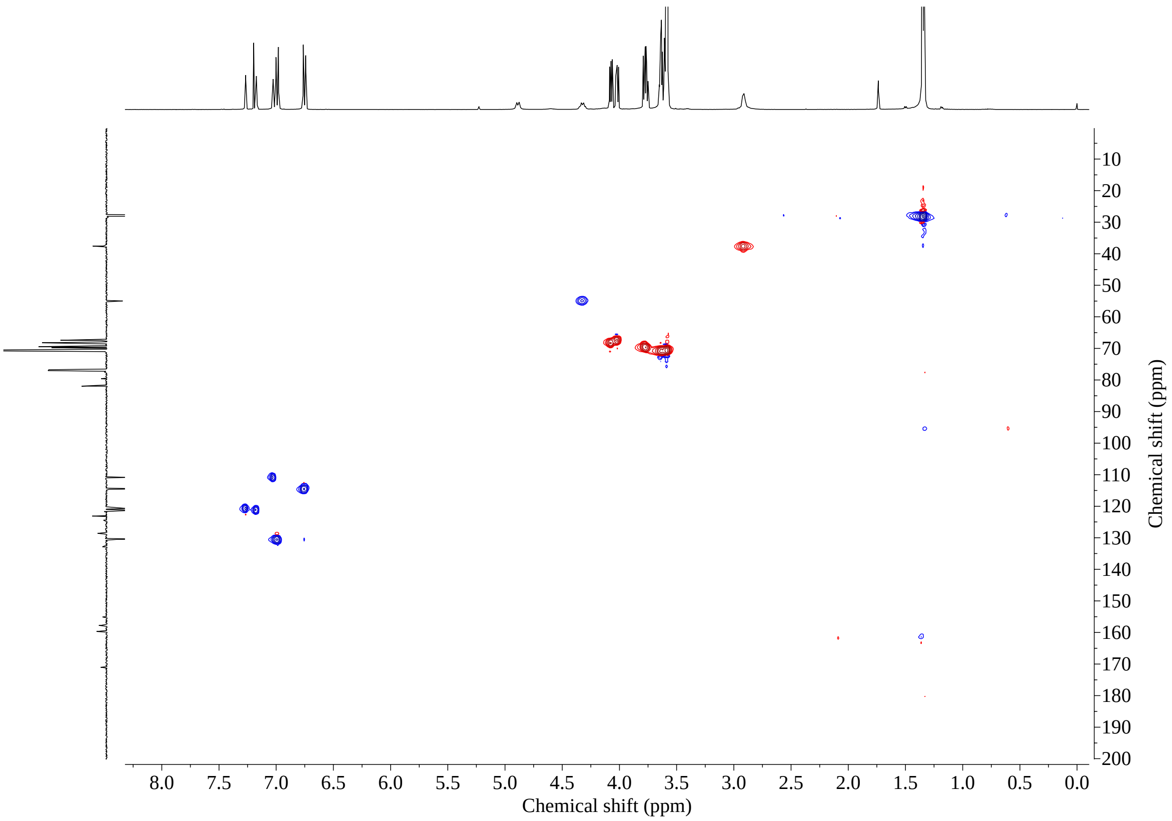

Figure S76. HSQC NMR of (*S*)-**S5c** ($CDCl_3$, 298K)

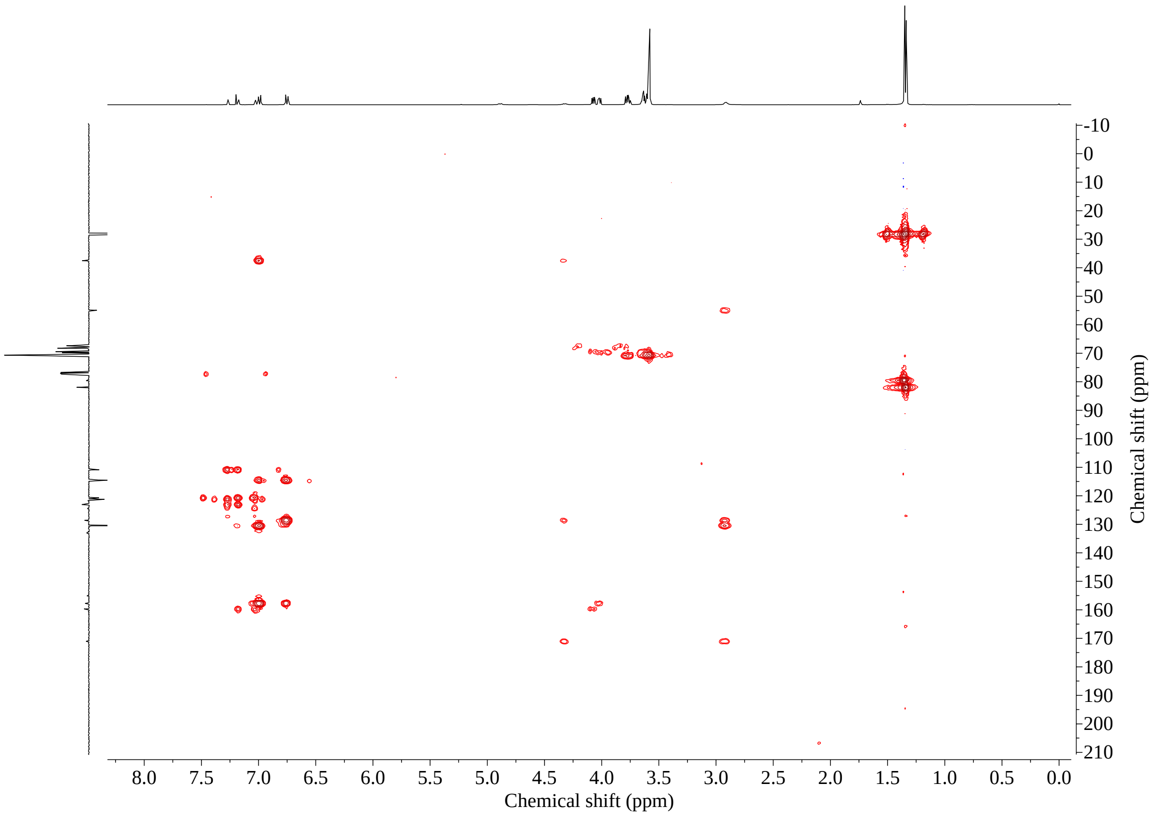

Figure S77. HMBC NMR of (*S*)-**S5c** ($CDCl_3$, 298 K)

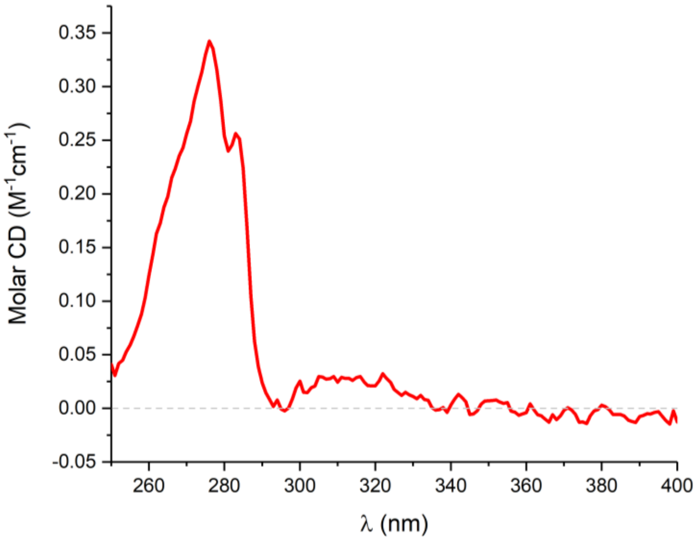

Figure S78. Circular Dichroism Spectra of (*S*)-**S5c** (164 µM) at 293 K in $CHCl_3$

TMS acetylene (*S*)-S6c

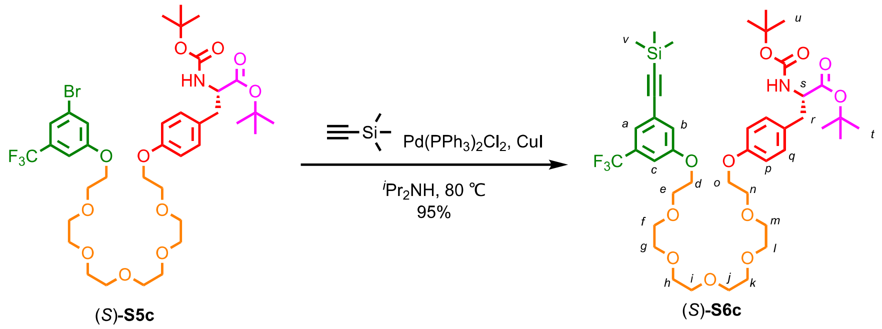

(*S*)-**S5c** (807 mg, 0.98 mmol, 1.0 equiv.), $Pd(PPh_3)_2Cl_2$ (14 mg, 0.020 mmol, 0.02 equiv.) and CuI (8 mg, 0.04 mmol, 0.04 equiv.) were suspended in $^{i}Pr_2NH$ (10 mL). The resulting suspension was degassed by bubbling N_2 through the solution over 5 min. Ethynyltrimethylsilane (210 µL, 1.47 mmol, 1.5 equiv.) was added and the mixture was stirred at 80 °C for 96 h. The solvent was removed *in vacuo* and the crude was purified by column chromatography (petrol-Et_2O 50 : 50 → 0 : 100) to give (*S*)-**S6c** as a yellow oil (787 mg, 95%).

^{1}H NMR (400 MHz, $CDCl_3$, 298 K) δ 7.31-7.29 (m, 1H, H_a), 7.15-7.12 (m, 1H, H_b), 7.12-7.10 (m, 1H, H_c), 7.06 (d, *J* = 8.8, 2H, H_q), 6.82 (d, *J* = 8.5, 2H, H_p), 4.95 (d, *J* = 8.4, 1H, NH), 4.39 (q, *J* = 6.3, 1H, H_s), 4.19-4.06 (m, 4H, H_d, H_o), 3.88-3.79 (m, 4H, H_e, H_n), 3.74-3.58 (m, 16H, H_f, H_g, H_h, H_i, H_j, H_k, H_l, H_m), 3.04-2.89 (m, 2H, H_r), 1.41 (s, 9H, H_t *or* H_u), 1.39 (s, 9H, H_t *or* H_u), 0.25 (s, 9H, H_v).

^{19}F NMR (376 MHz, $CDCl_3$, 298 K) δ: -63.13 (s, 3F, CF_3)

^{13}C NMR (101 MHz, $CDCl_3$, 298 K) δ 171.1, 158.8, 157.9, 155.2, 132.0 (q, $J_{C\text{-}F}$ = 32.8), 130.6, 128.7, 125.2, 124.9 (q, $J_{C\text{-}F}$ = 272.8), 121.3 (q, $J_{C\text{-}F}$ = 3.8), 120.6, 114.6, 112.6 (q, $J_{C\text{-}F}$ = 3.8), 103.4, 96.0, 82.0, 79.7, 71.0, 70.9, 70.7, 70.7 (×3), 70.7 (×2), 69.8, 69.6, 68.1, 67.5, 55.0, 37.6, 28.4, 28.1, -0.1.

HR-ESI-MS *m/z* = 864.3932 $[M+Na]^+$ calc. 864.3936 for $C_{42}H_{62}F_3NNaO_{11}Si$.

$[\alpha]_D^{23}$ +9.8 (*c* 0.88, $CHCl_3$)

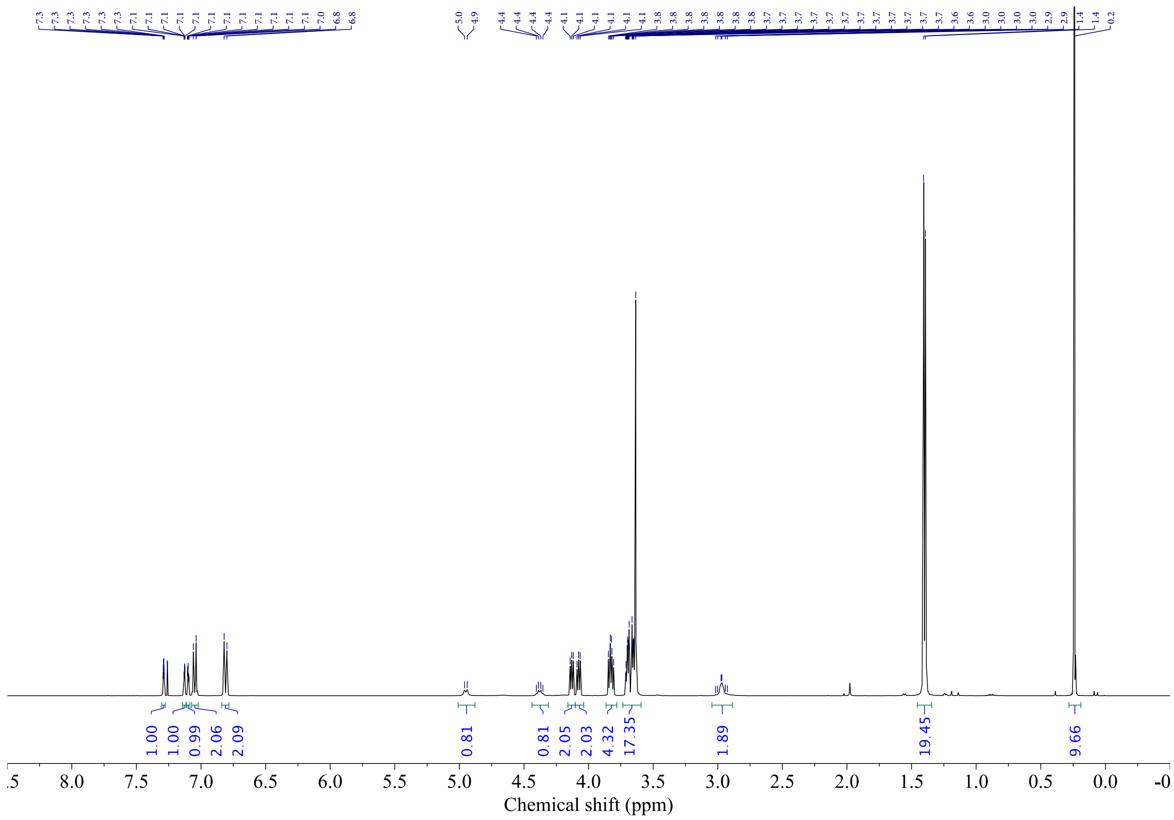

Figure S79. ^{1}H NMR of (*S*)-**S6c** ($CDCl_3$, 400 MHz, 298 K)

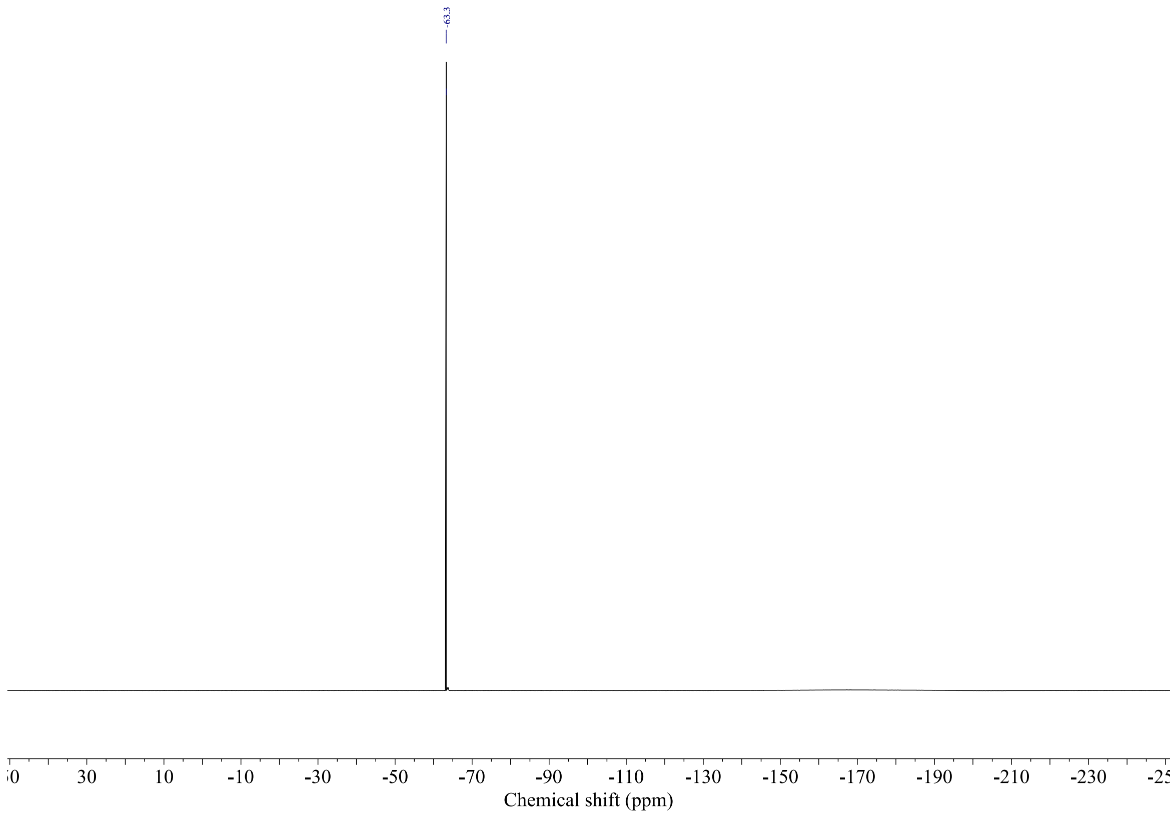

Figure S80. ^{19}F NMR of (*S*)-**S6c** ($CDCl_3$, 376 MHz, 298 K)

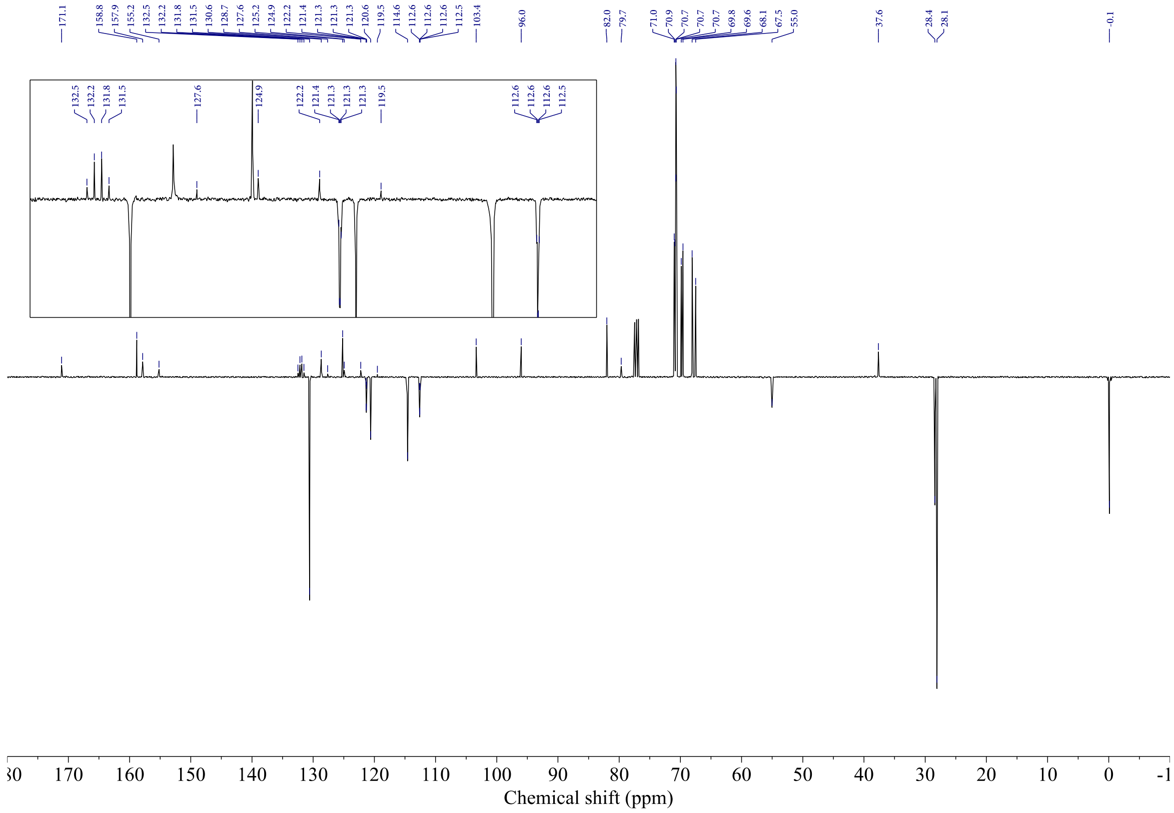

Figure S81. JMOD NMR of (*S*)-**S6c** ($CDCl_3$, 101 MHz, 298 K)

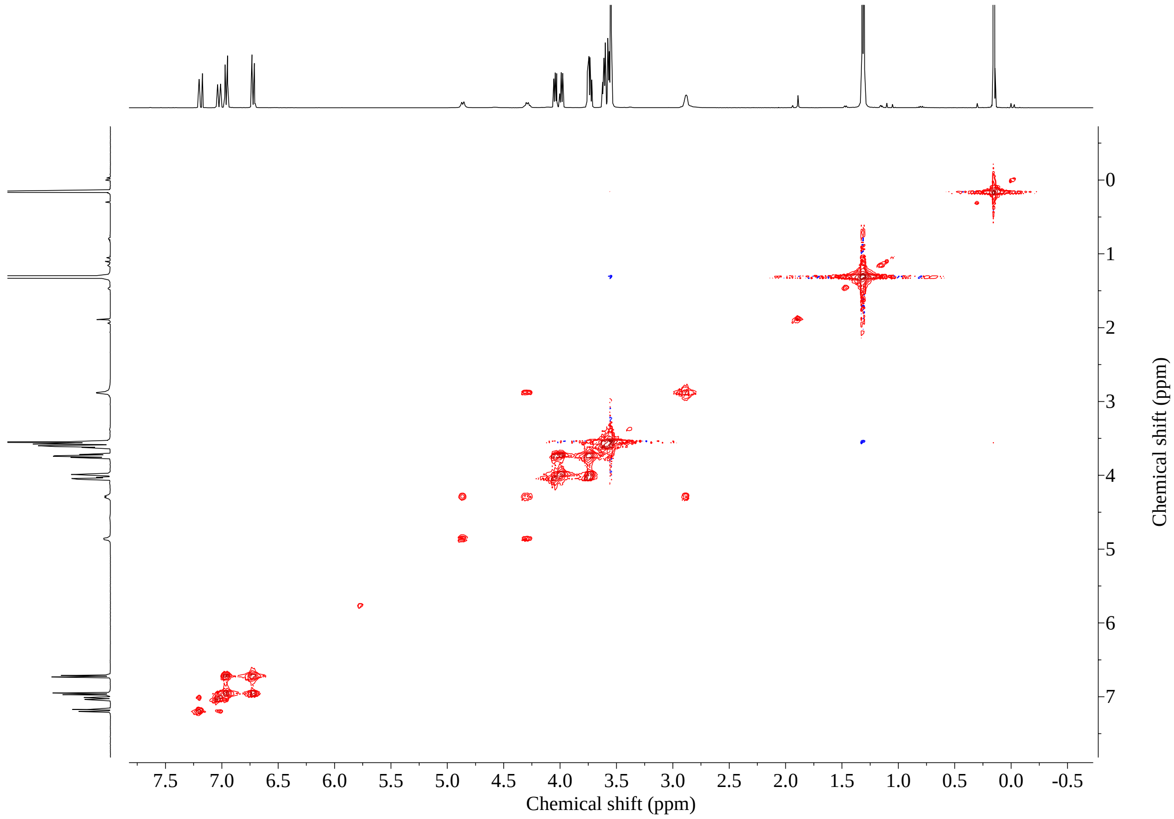

Figure S82. COSY NMR of (*S*)-**S6c** ($CDCl_3$, 298 K)

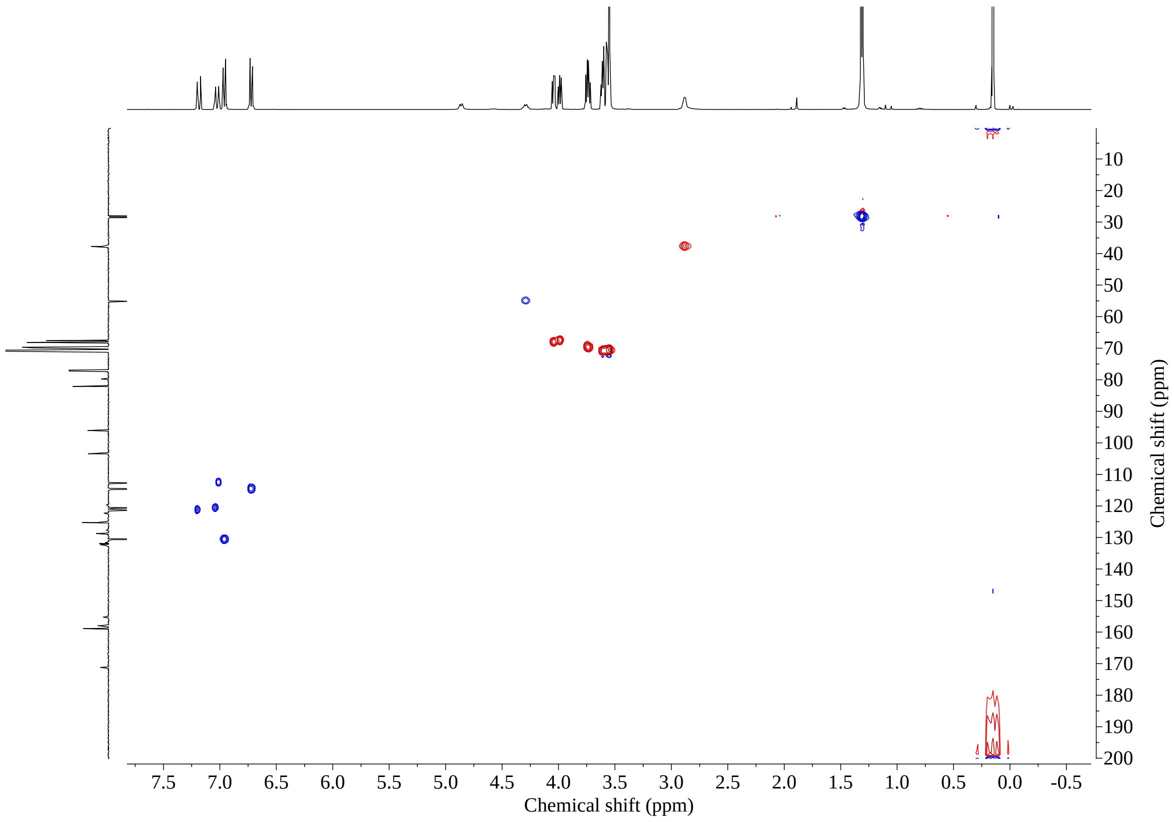

Figure S83. HSQC NMR of (*S*)-**S6c** ($CDCl_3$, 298 K)

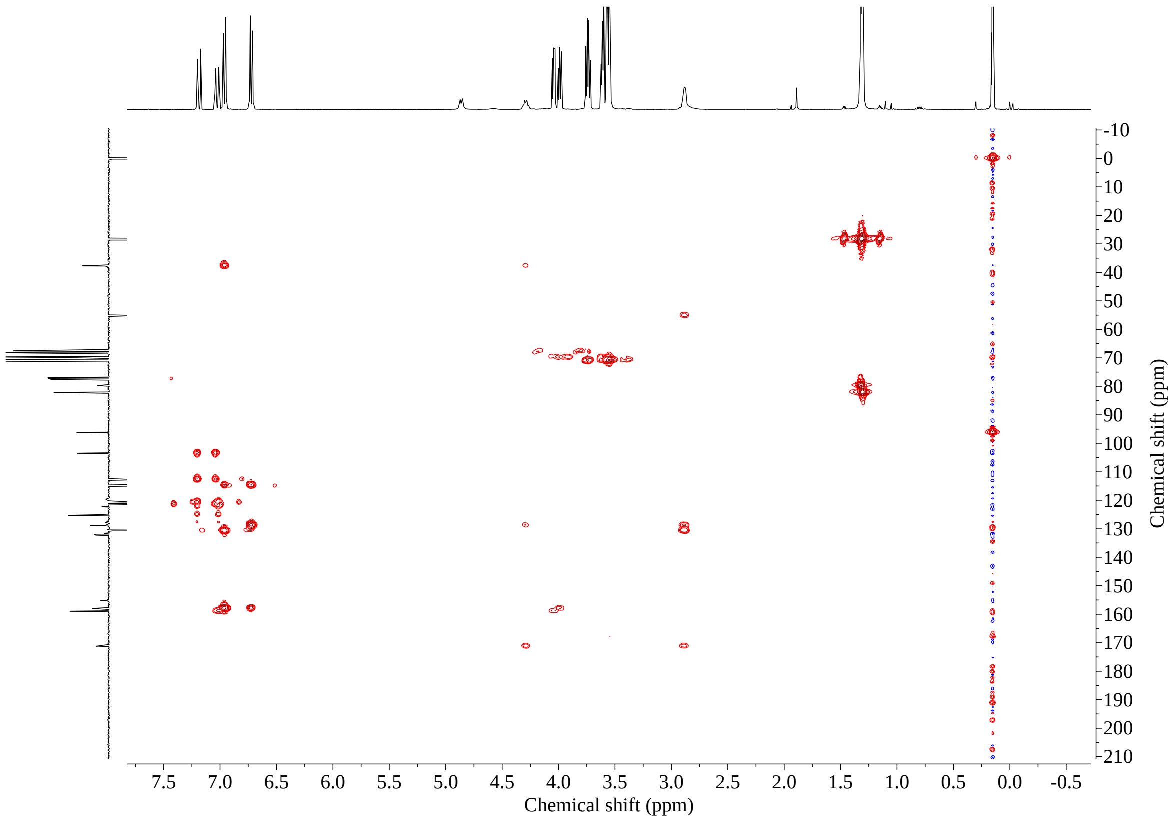

Figure S84. HMBC NMR of (*S*)-**S6c** ($CDCl_3$, 298 K)

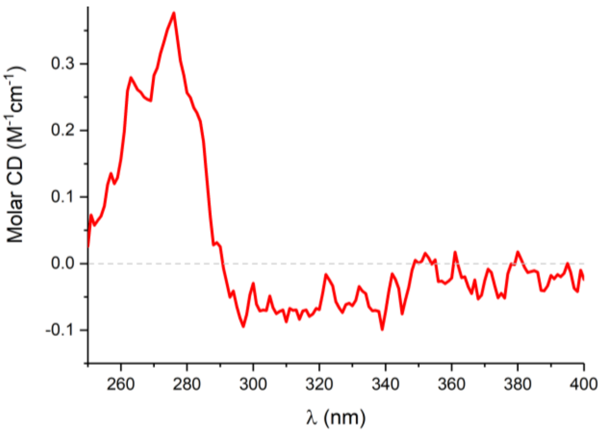

Figure S85. Circular Dichroism Spectra of (*S*)-**S6c** (39.6 µM) at 293 K in $CHCl_3$

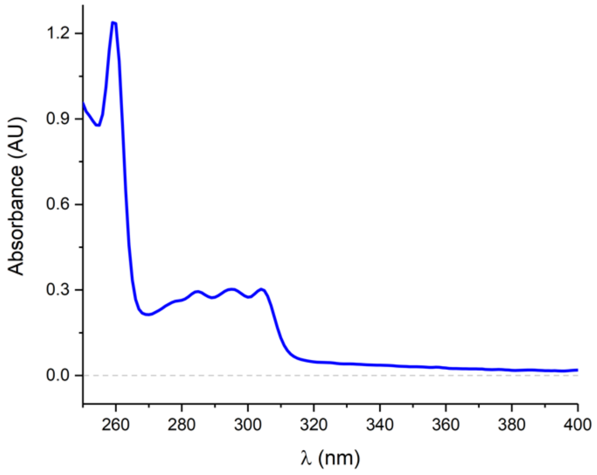

Figure S86. UV-Vis Spectra of (*S*)-**S6c** (39.6 µM) at 293 K in $CHCl_3$

Terminal acetylene (*S*)-S7c

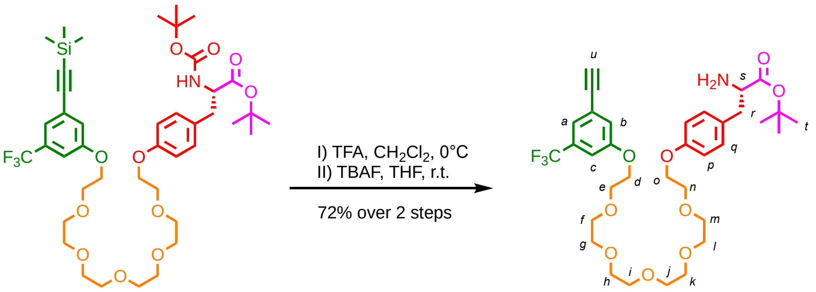

(*S*)-**S6c** (120 mg, 0.14 mmol, 1.0 equiv.) was dissolved in CH_2Cl_2 (1.5 mL). Trifluoroacetic acid (0.10 mL, 1.40 mmol, 10.0 equiv.) was added dropwise to the solution and the mixture was stirred at 0 ºC for 2 h. The reaction mixture was quenched with sat. $NaHCO_3$ (10 mL), extracted with CH_2Cl_2 (20 mL) and the organic phase dried ($MgSO_4$). The solvent was removed *in vacuo*. The crude was re-dissolved in THF (1.5 mL), tetrabutylammonium fluoride (1 M in THF, 0.28 mL, 0.28 mmol, 2.0 equiv.) was added and the reaction mixture was stirred at rt for 4 h. The reaction mixture was partitioned between H_2O (10 mL) and EtOAc (10 mL), the phases separated and the organic layer dried ($MgSO_4$). The solvent was removed *in vacuo* and the crude was purified by column chromatography (deactivated SiO_2, CH_2Cl_2-acetone 0 → 20%) to give (*S*)-**S7c** as a colorless oil (70 mg, 72% over 2 steps).

^{1}H NMR (400 MHz, $CDCl_3$, 298 K) δ 7.35-7.31 (m, 1H, H_a), 7.20-7.16 (m, 1H, H_b), 7.16-7.13 (m, 1H, H_c), 7.11 (d, *J* = 9.0, 2H, H_q), 6.84 (d, *J* = 8.8, 2H, H_p), 4.17-4.12 (m, 2H, H_d or H_o), 4.11-4.06 (m, 2H, H_d or H_o) 3.90-3.80 (m, 4H, H_e, H_n), 3.75-3.62, (m, 16H, H_f, H_g, H_h, H_i, H_j, H_k, H_l, H_m,), 3.61-3.54 (m, 1H, H_s), 3.12 (s, 1H, H_u) 2.96 (dd, *J* = 13.8, 6.3, 1H, H_r), 2.76 (dd, *J* = 13.8, 6.3, 1H, H_r), 1.43 (s, 9H, H_t).

^{19}F NMR (376 MHz, $CDCl_3$, 298 K) δ: -63.16 (s, 3F, CF_3).

^{13}C NMR (101 MHz, $CDCl_3$, 298 K) δ 174.5, 158.9, 157.7, 132.2 (q, $J_{C\text{-}F}$ = 32.8), 130.4, 129.8, 124.2, 123.5 (q, $J_{C\text{-}F}$ = 272.5), 121.4 (q, $J_{C\text{-}F}$ = 3.8), 121.1, 114.7, 112.8 (q, $J_{C\text{-}F}$ = 3.8), 82.2, 81.2, 78.7, 71.0, 70.9, 70.8, 70.7 (×3), 70.7 (×2), 69.9, 69.6, 68.1, 67.5, 56.5, 40.4, 28.1.

HR-ESI-MS *m/z* = 670.3197 $[M+H]^+$ calc. 670.3197 for $C_{34}H_{47}F_3NO_9$.

$[\alpha]_D^{23}$ −1.3 (*c* 0.80, $CHCl_3$)

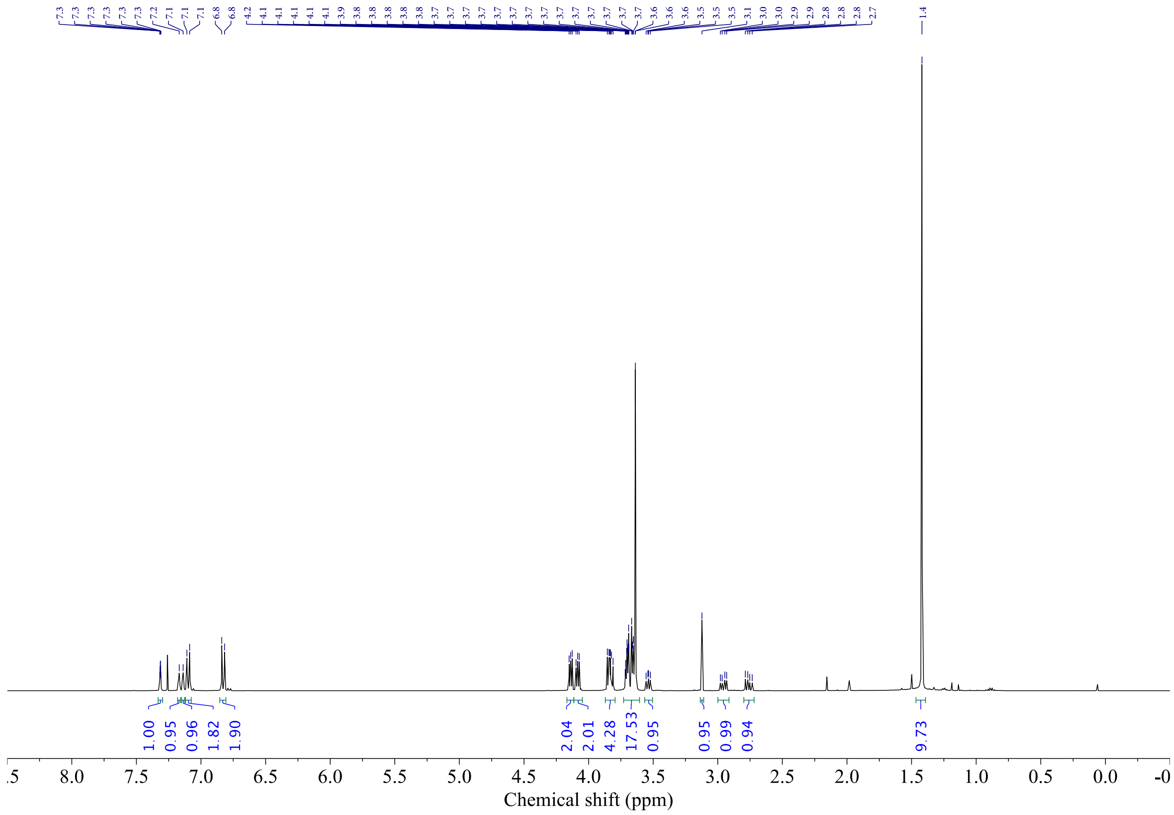

Figure S87. ^{1}H NMR of (*S*)-**S7c** ($CDCl_3$, 400 MHz, 298 K)

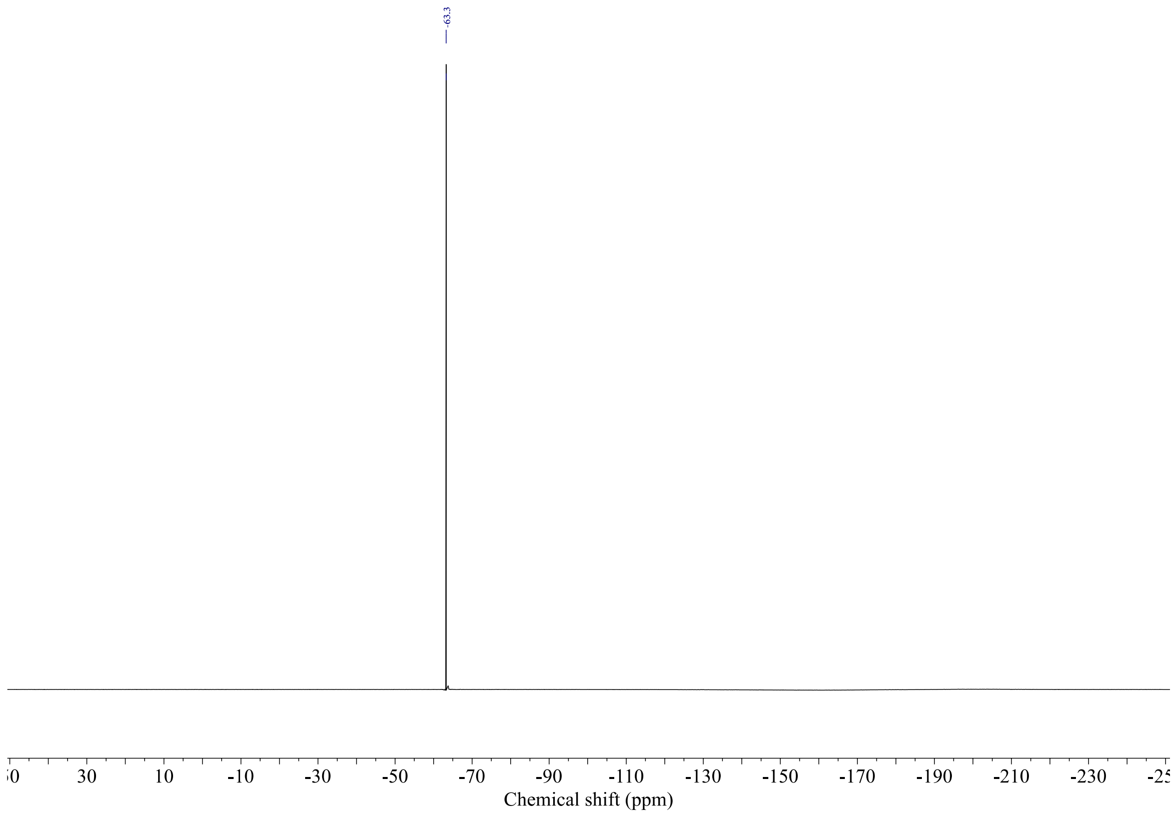

Figure S88. ^{19}F NMR of (*S*)-**S7c** ($CDCl_3$, 376 MHz, 298 K)

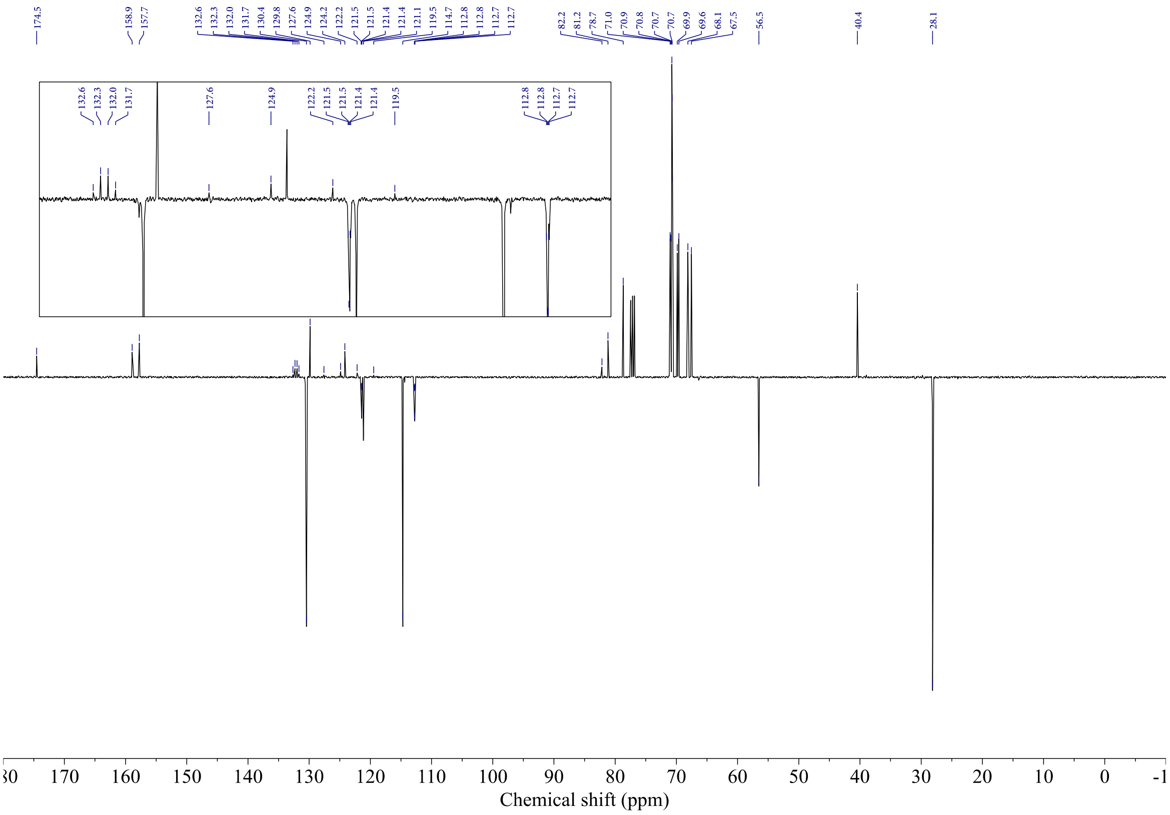

Figure S89. JMOD NMR of (*S*)-**S7c** ($CDCl_3$, 101 MHz, 298 K)

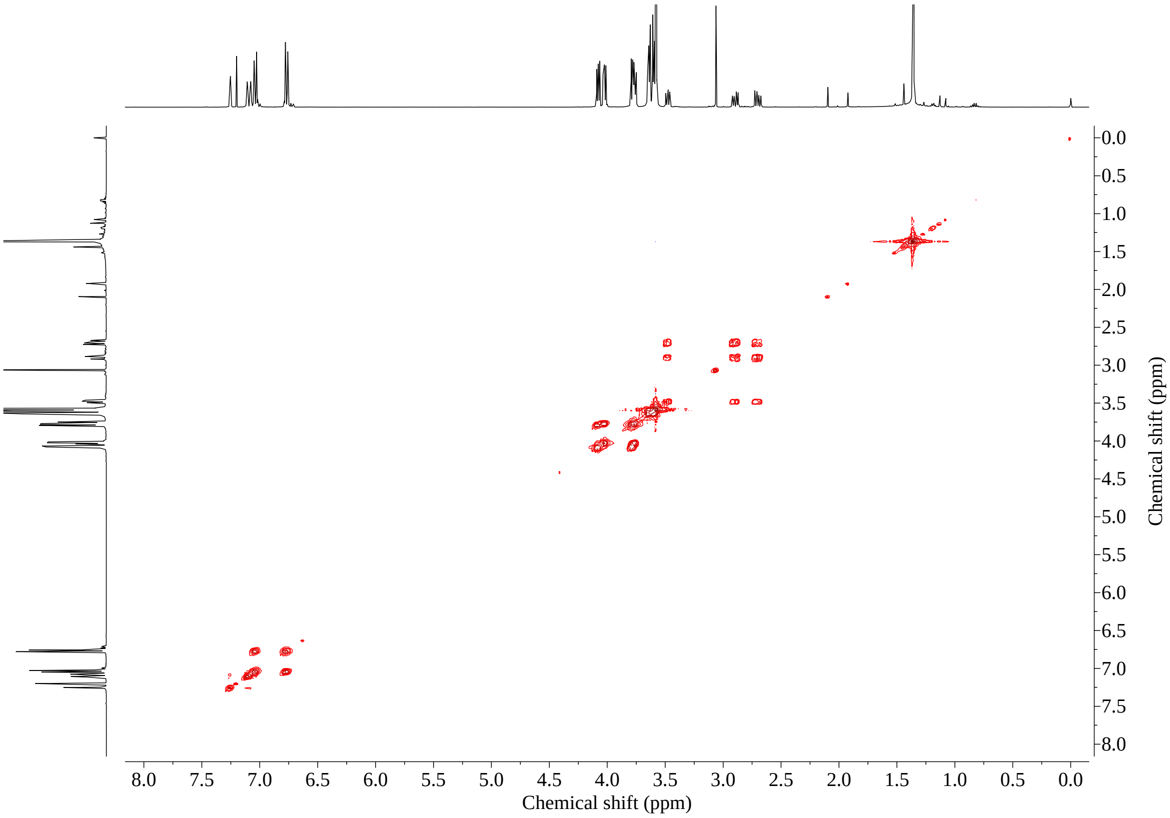

Figure S90. COSY NMR of (*S*)-**S7c** ($CDCl_3$, 298 K)

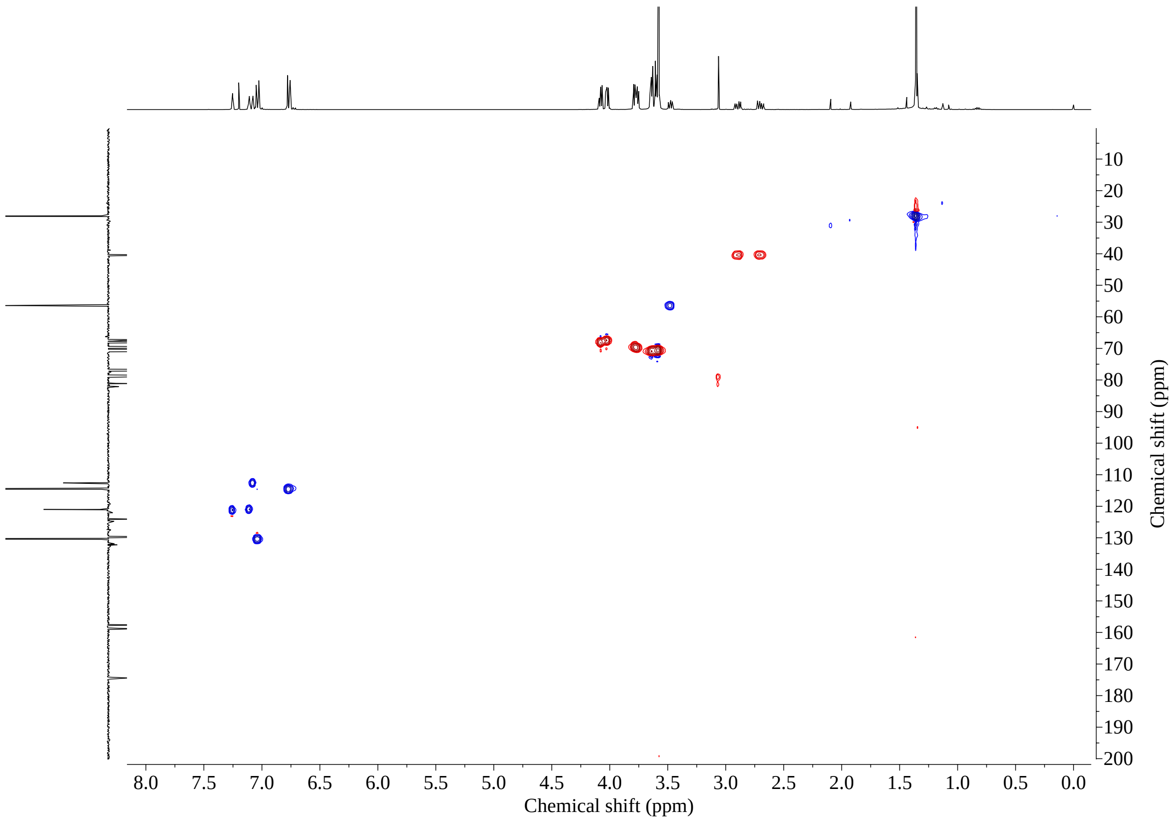

Figure S91. HSQC NMR of (*S*)-**S7c** ($CDCl_3$, 298 K)

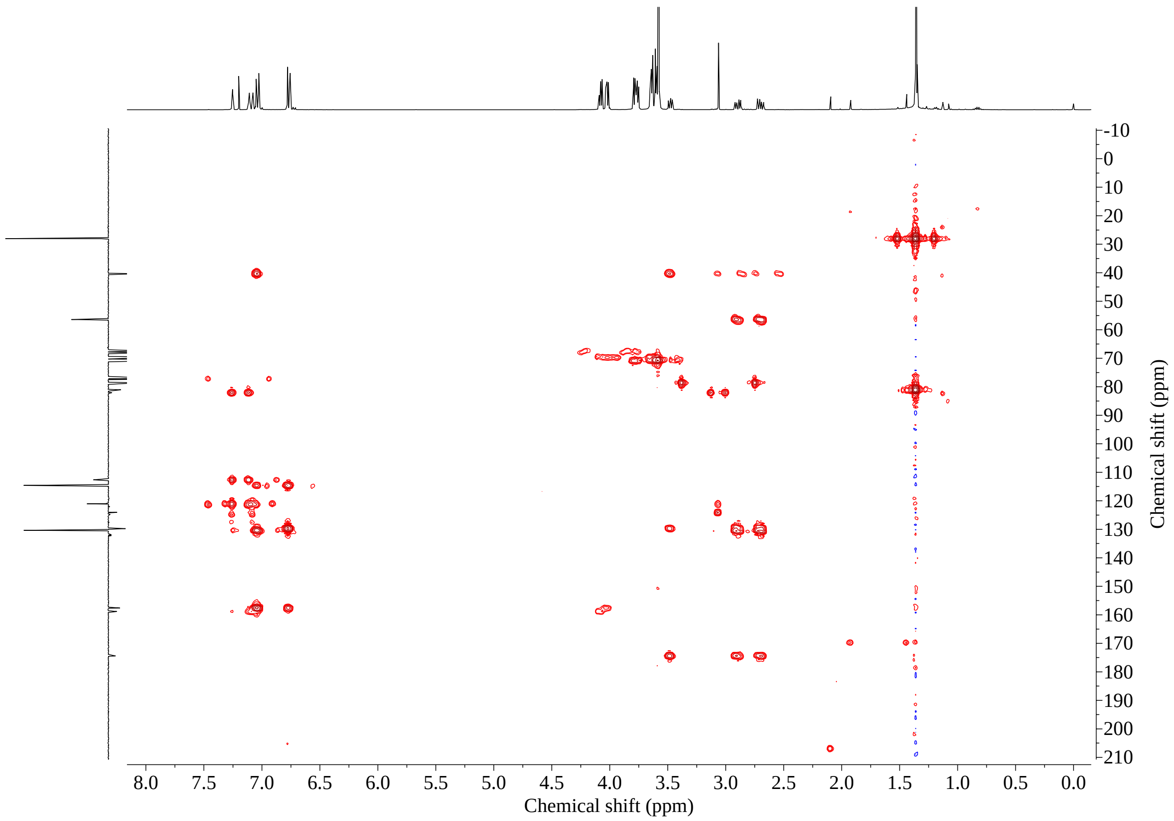

Figure S92. HMBC NMR of (*S*)-**S7c** ($CDCl_3$, 298 K)

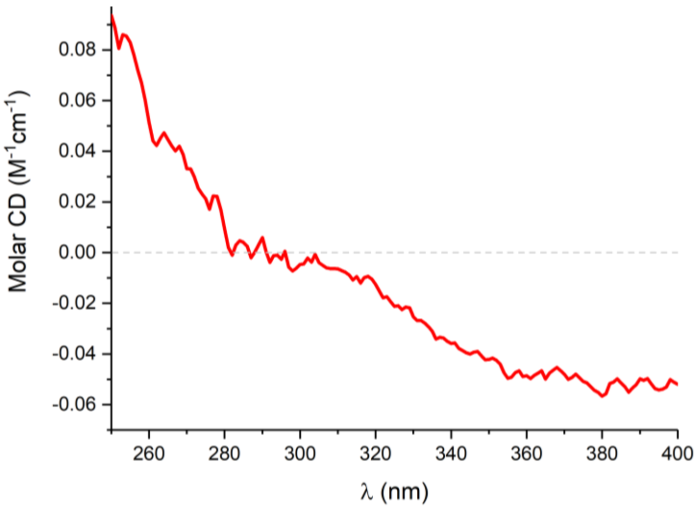

Figure S93. Circular Dichroism Spectra of (*S*)-**S7c** (124 μM) at 293 K in $CHCl_3$

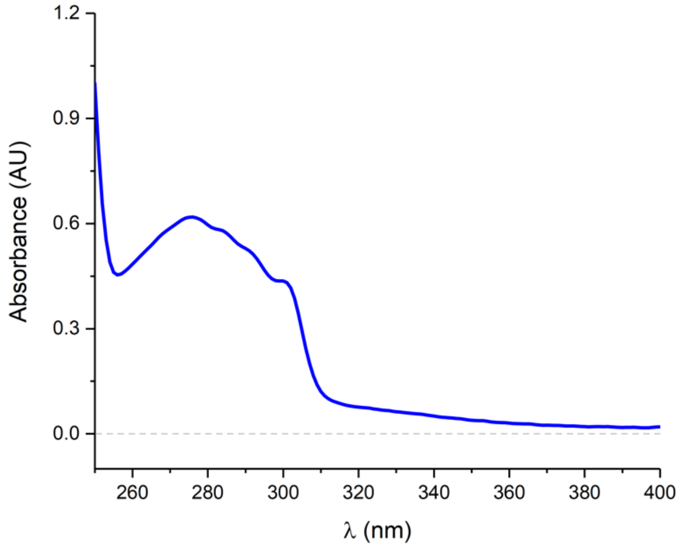

Figure S94. UV-Vis Spectra of (*S*)-**S7c** (124 μM) at 293 K in $CHCl_3$

Macrocycle precursor (*S*)-1c

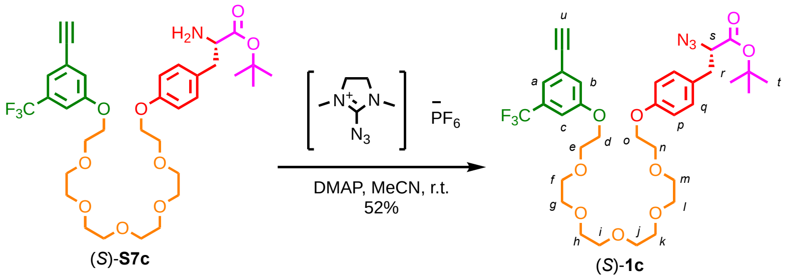

(*S*)-**S7c** (19.8 mg, 0.030 mmol, 1.0 equiv.), DMAP (11.0 mg, 0.090 mmol, 3.0 equiv.) and ADMP (12.9 mg, 0.060 mmol, 2.0 equiv.) were dissolved in CH_3CN (0.5 mL). The mixture was stirred at rt for 16 h before quenching with H_2O (10 mL). The mixture was extracted with CH_2Cl_2 (15 mL) and the organic layer dried ($MgSO_4$). The solvent was removed *in vacuo* and the crude was purified by column chromatography (CH_2Cl_2-EtOAc 100 : 0 → 70 : 30) to obtain (*S*)-**1c** as a colorless oil (11 mg, 52% yield).

^{1}H NMR (400 MHz, $CDCl_3$, 298 K) δ 7.34-7.31 (m, 1H, H_a), 7.20-7.17 (m, 1H, H_b), 7.16-7.10 (m, 3H, H_c, H_q), 6.85 (d, *J* = 8.5, 2H, H_p), 4.17-4.13 (m, 2H, H_d), 4.12-4.07 (m, 2H, H_o), 3.89-3.80 (m, 5H, H_e, H_n, H_s), 3.74-3.62 (m, 16H, H_f, H_g, H_h, H_i, H_j, H_k, H_l, H_m), 3.12 (s, 1H, H_u) 3.06 (dd, *J* = 14.3, 5,8, 1H, H_r), 2.93 (dd, *J* = 13.4, 6.0, 1H, H_r), 1.45 (s, 9H, H_t)

^{19}F NMR (376 MHz, $CDCl_3$, 298 K) δ: -63.14 (s, 3F, CF_3)

^{13}C NMR (101 MHz, $CDCl_3$, 298 K) δ 169.1, 158.9, 158.0, 132.0 (q, $J_{C\text{-}F}$ = 32.8), 130.3, 128.4, 124.1, 123.5 (q, $J_{C\text{-}F}$ = 272.7), 121.3 (q, $J_{C\text{-}F}$ = 3.8), 121.1, 114.7, 112.7 (q, $J_{C\text{-}F}$ = 3.8), 82.9, 82.1, 78.7, 70.9, 70.8, 70.7, 70.6, 70.6 (×3), 70.6 (×2), 69.7, 69.5, 68.1, 67.5, 63.8, 36.8, 28.0.

HR-ESI-MS *m/z* = 718.2924 $[M+Na]^+$ calc. 718.2922 for $C_{34}H_{44}F_3N_3NaO_9$.

$[\alpha]_D^{23}$ −11.8 (*c* 0.74, $CHCl_3$)

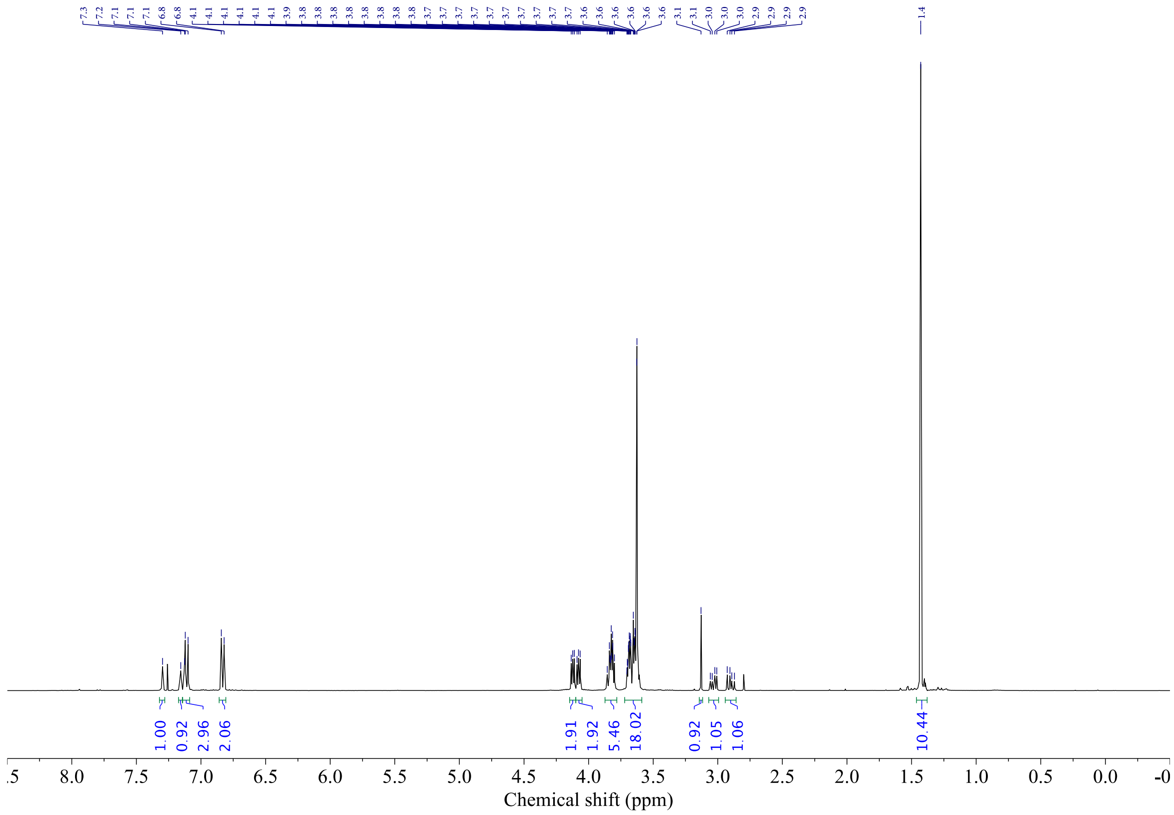

Figure S95. ^{1}H NMR of (*S*)-**1c** ($CDCl_3$, 400 MHz, 298 K)

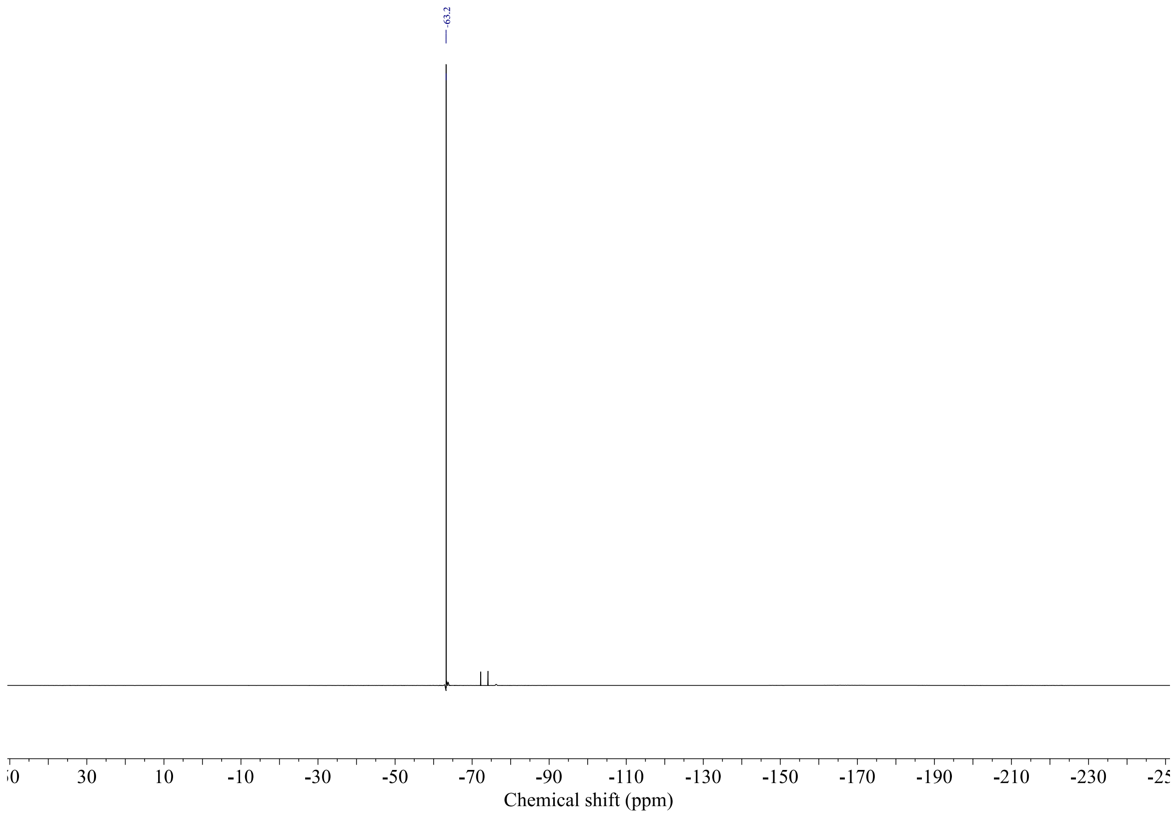

Figure S96. ^{19}F NMR of (*S*)-**1c** ($CDCl_3$, 376 MHz, 298 K)

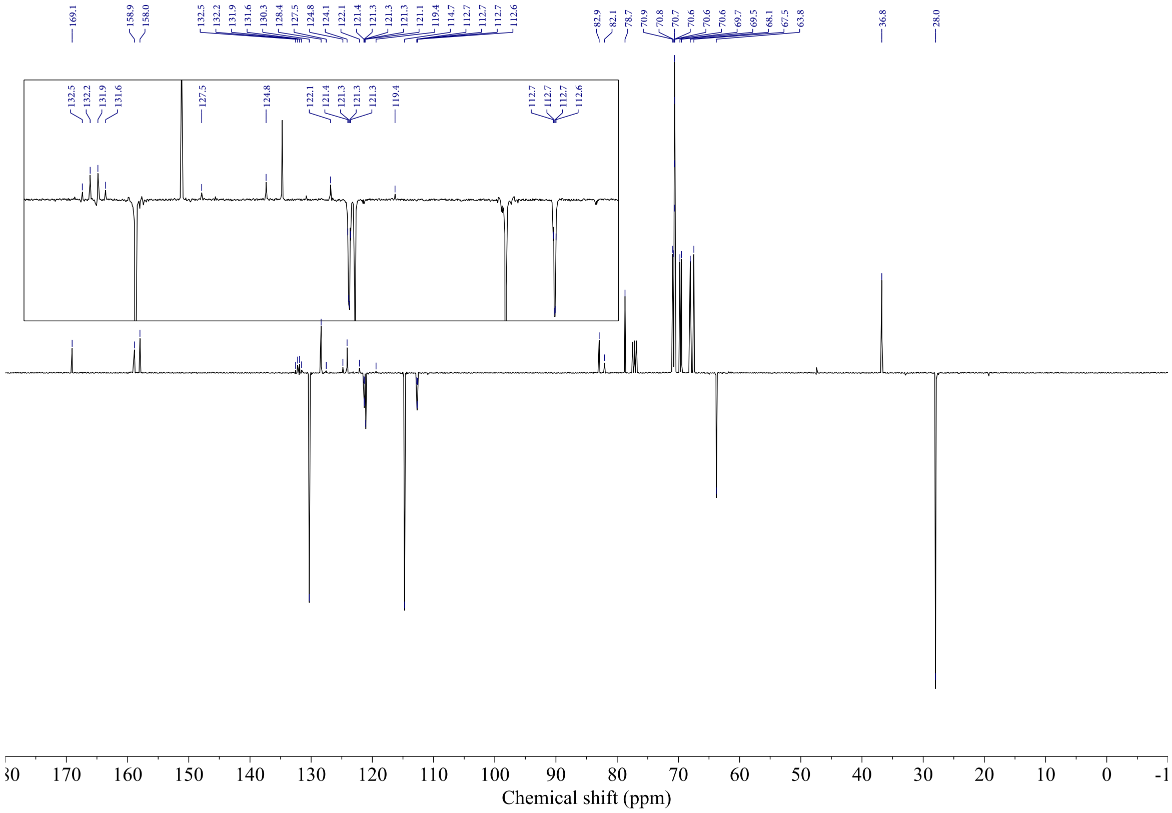

Figure S97. JMOD NMR of (*S*)-**1c** ($CDCl_3$, 101 MHz, 298 K)

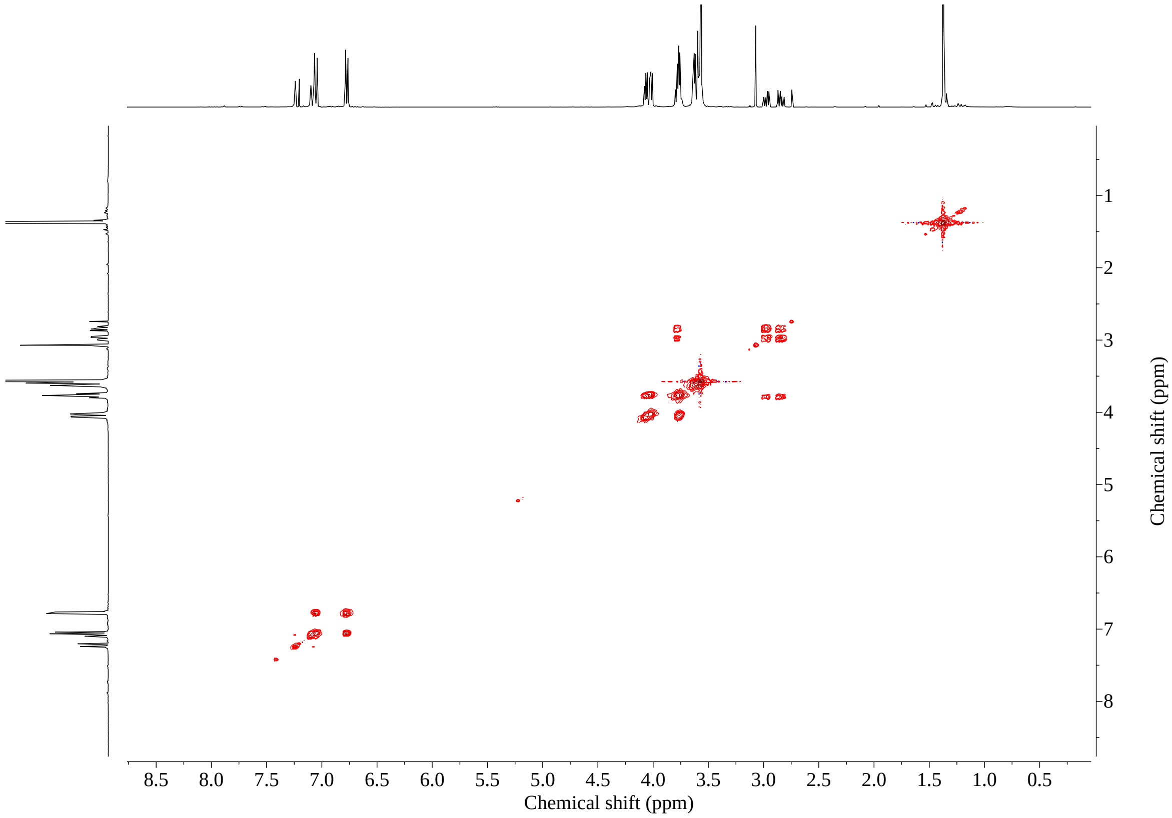

Figure S98. COSY NMR of (*S*)-**1c** ($CDCl_3$, 298 K)

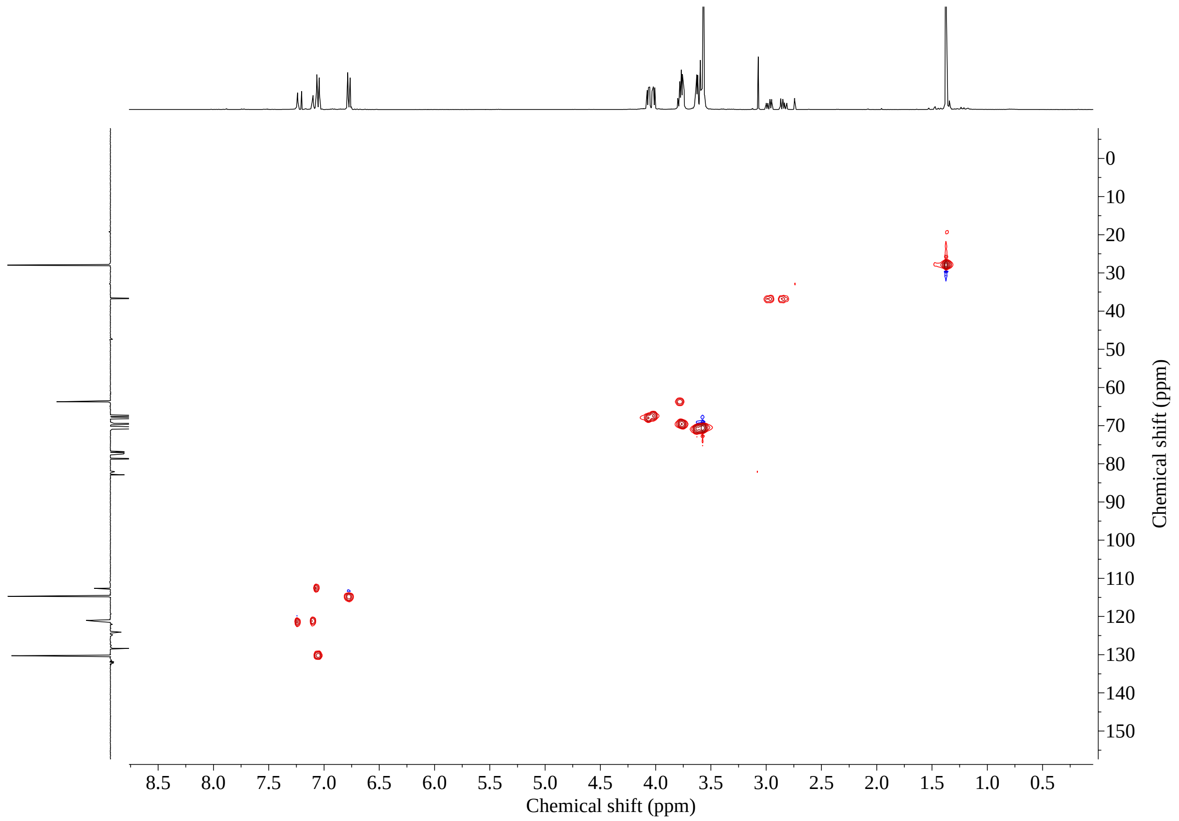

Figure S99. HSQC NMR of (*S*)-**1c** ($CDCl_3$, 298 K)

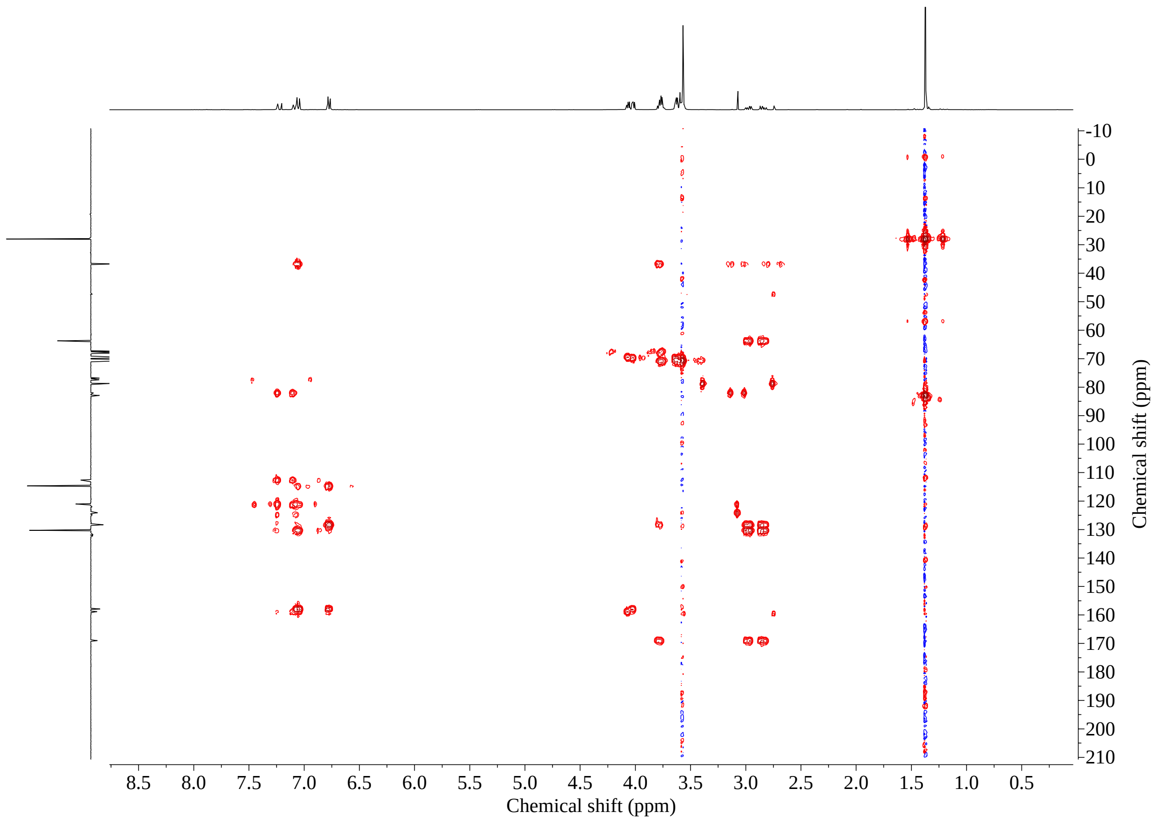

Figure S100. HMBC NMR of (*S*)-**1c** ($CDCl_3$, 298 K)

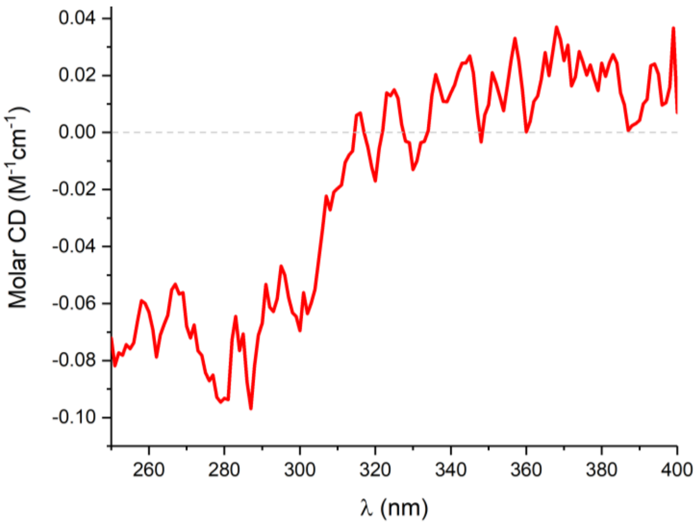

Figure S101. Circular Dichroism Spectra of (*S*)-**1c** (104 μM) at 293 K in $CHCl_3$

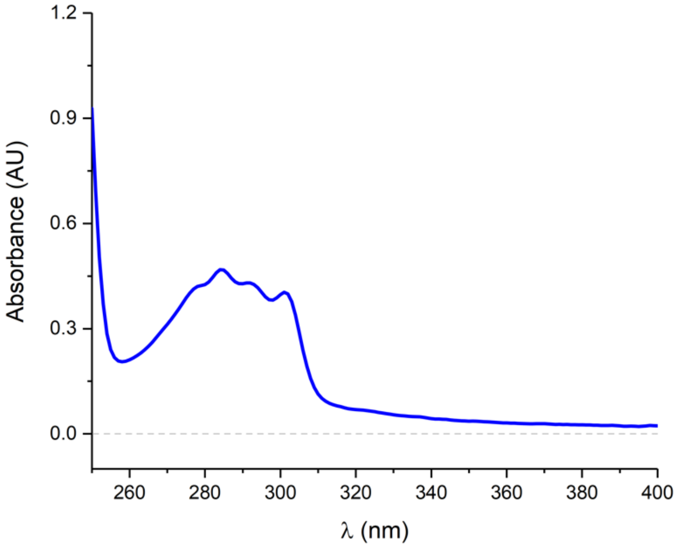

Figure S102. UV-Vis Spectra of (*S*)-**1c** (104 μM) at 293 K in $CHCl_3$

$[Cu(CH_3CN)_2(2)]PF_6$

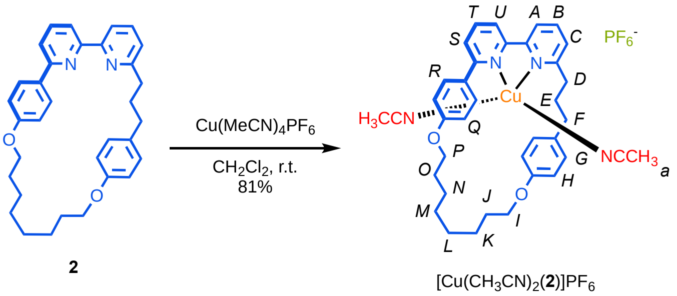

2 (100 mg, 0.20 mmol, 1.0 equiv.) and $[Cu(CH_3CN)_4]PF_6$ (72 mg, 0.19 mmol, 0.96 equiv.) were dissolved in dry CH_2Cl_2 (2 mL). The solution was stirred at rt until a clear, deep-red solution was obtained (~15 min. The solvent was removed *in vacuo* to give $[Cu(CH_3CN)_2($**2**$)]PF_6$ as an ochre foam (121 mg, 81%). No further purification was required.

^{1}H NMR (400 MHz, $CDCl_3$, 298 K) δ: 8.23-7.92 (m, 4H, H_A, H_B, H_T, H_U), 7.80-7.70 (m, 1H, H_S), 7.66-7.51 (m, 3H, H_C, H_R), 7.05 (d, *J* = 7.8, 4H, H_G, H_Q), 6.78 (d, *J* = 8.0, 2H, H_H), 4.09 (t, *J* = 5.5, 2H, H_P), 3.85 (t, *J* = 5.8, 2H, H_I), 2.96 (t, *J* = 7.4, 2H, H_D), 2.67-2.52 (m, 2H, H_F), 2.18-1.68 (m, 12H, H_E, H_O, H_J, H_a), 1.63-1.32 (m, 8H, H_K, H_L, H_M, H_N).

^{19}F NMR (376 MHz, $CDCl_3$, 298 K) δ: -73.26 (d, $J_{F\text{-}P}$ = 712.5, 6F, PF_6)

^{31}P NMR (162 MHz, $CDCl_3$, 298 K) δ: -141.4 (sept, $J_{P\text{-}F}$ = 712.7, 1P, PF_6)

^{13}C NMR (101 MHz, $CDCl_3$, 298 K) δ: 162.6, 160.7, 159.1, 157.5, 152.3, 151.9, 140.6, 140.5, 133.0, 131.7, 130.2, 129.7, 125.9, 125.3, 120.3, 120.2, 116.4 (from HMBC),114.6, 114.5, 67.6, 67.4, 38.7, 33.2, 31.0, 28.9, 28.1, 28.0, 27.7, 25.5, 24.8, 2.0.

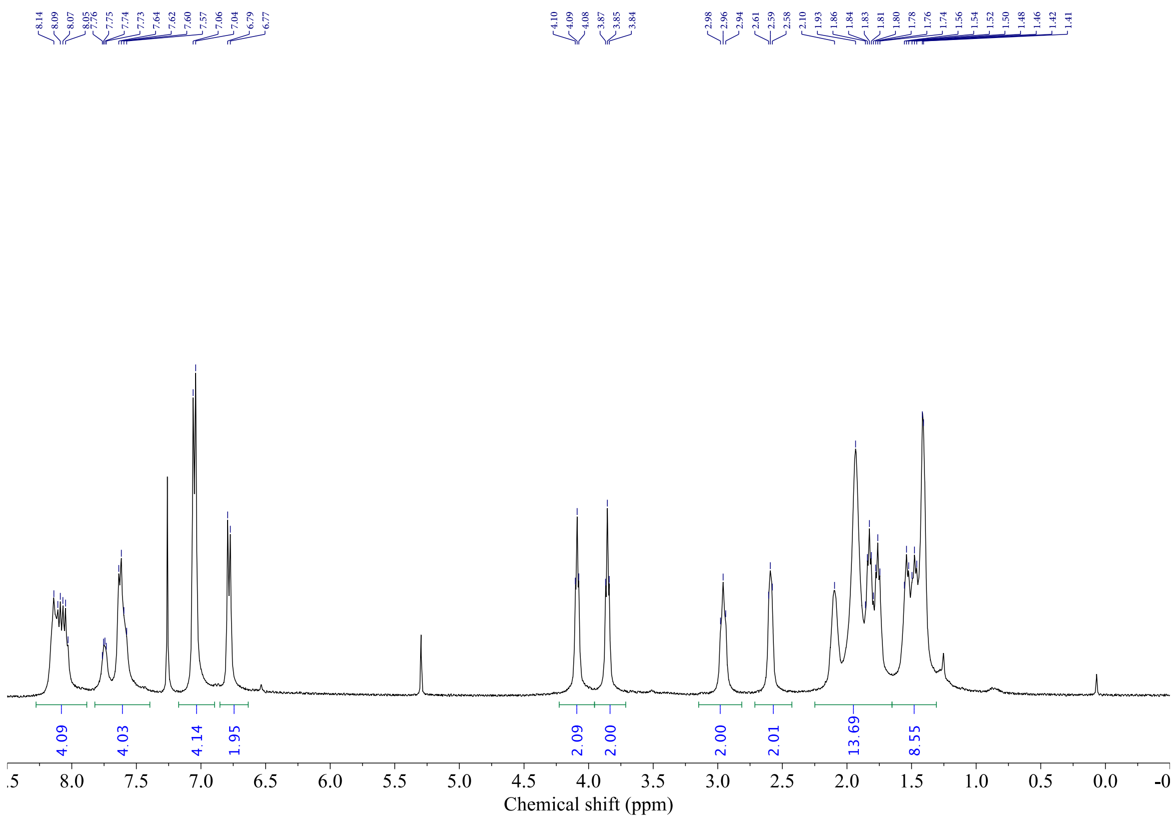

Figure S103. ^{1}H NMR of $[Cu(CH_3CN)_2(\mathbf{2})]PF_6$ ($CDCl_3$, 400 MHz, 298 K)

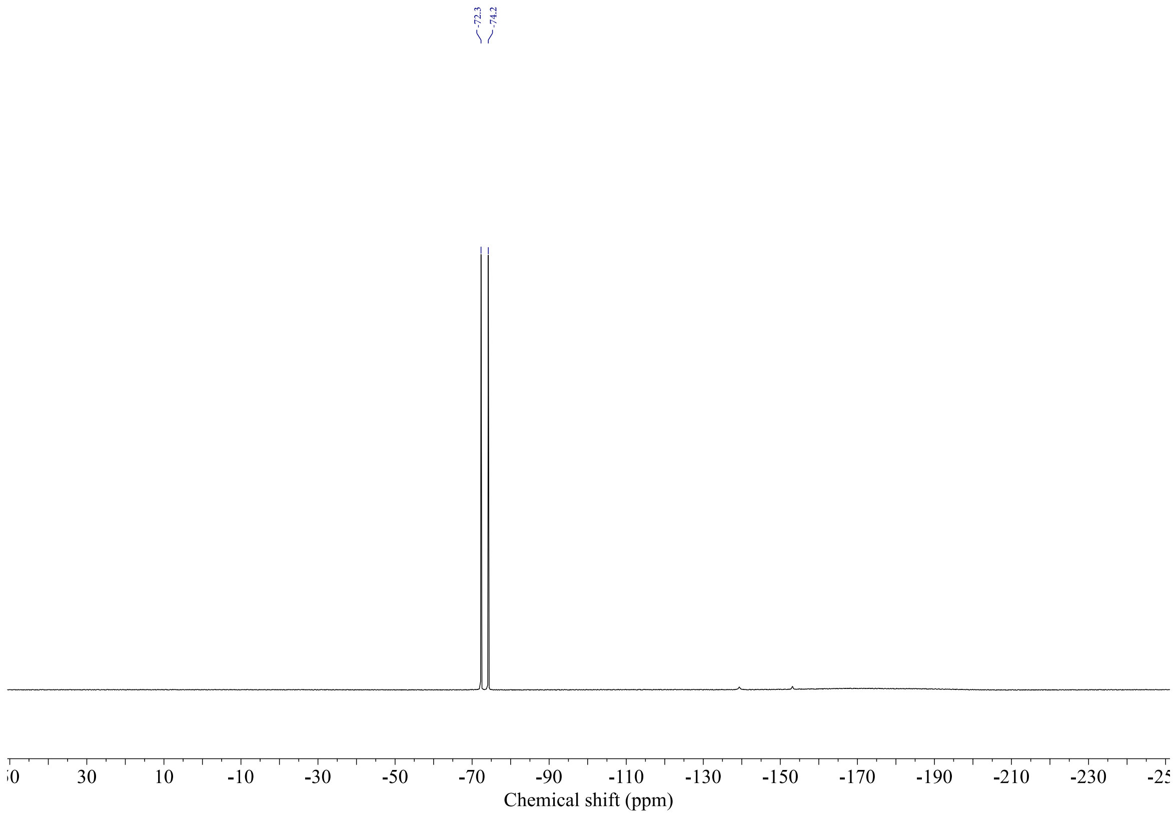

Figure S104. ^{19}F NMR of $[Cu(CH_3CN)_2(\mathbf{2})]PF_6$ ($CDCl_3$, 376 MHz, 298 K)

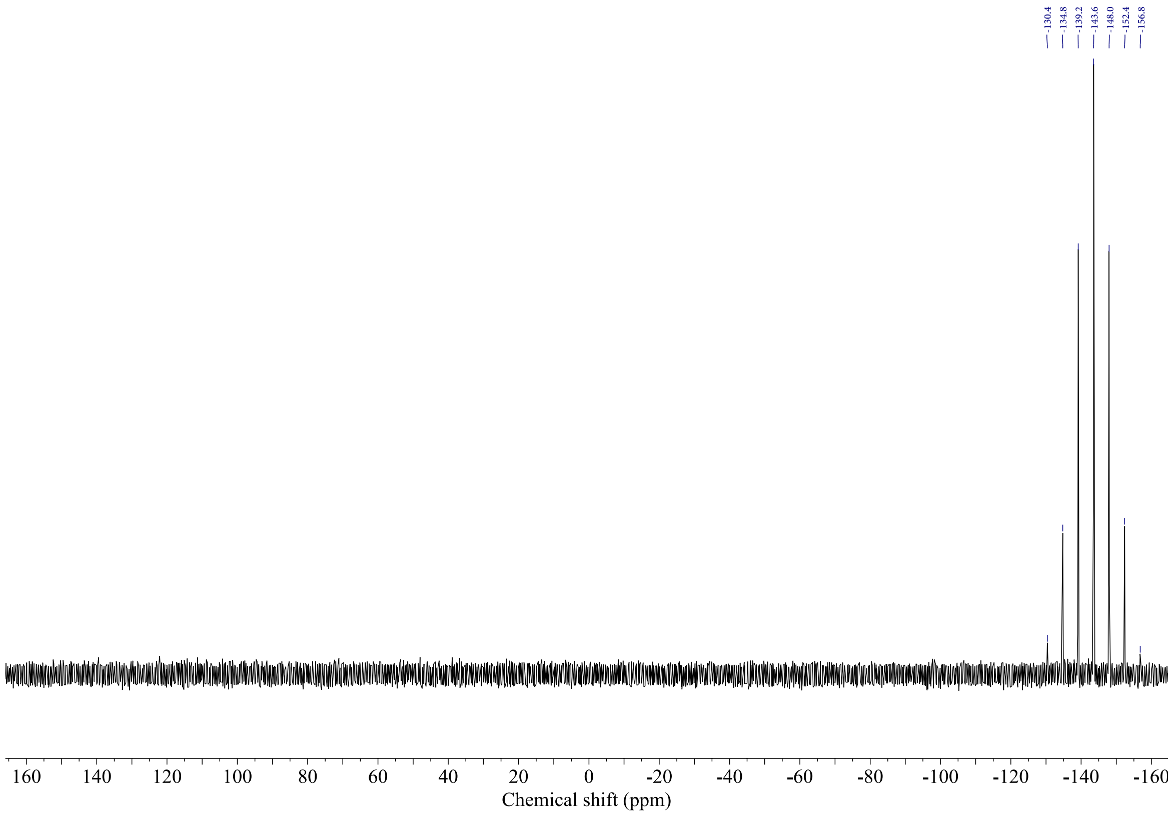

Figure S105. ^{31}P NMR of $[Cu(CH_3CN)_2(\mathbf{2})]PF_6$ ($CDCl_3$, 162 MHz, 298 K)

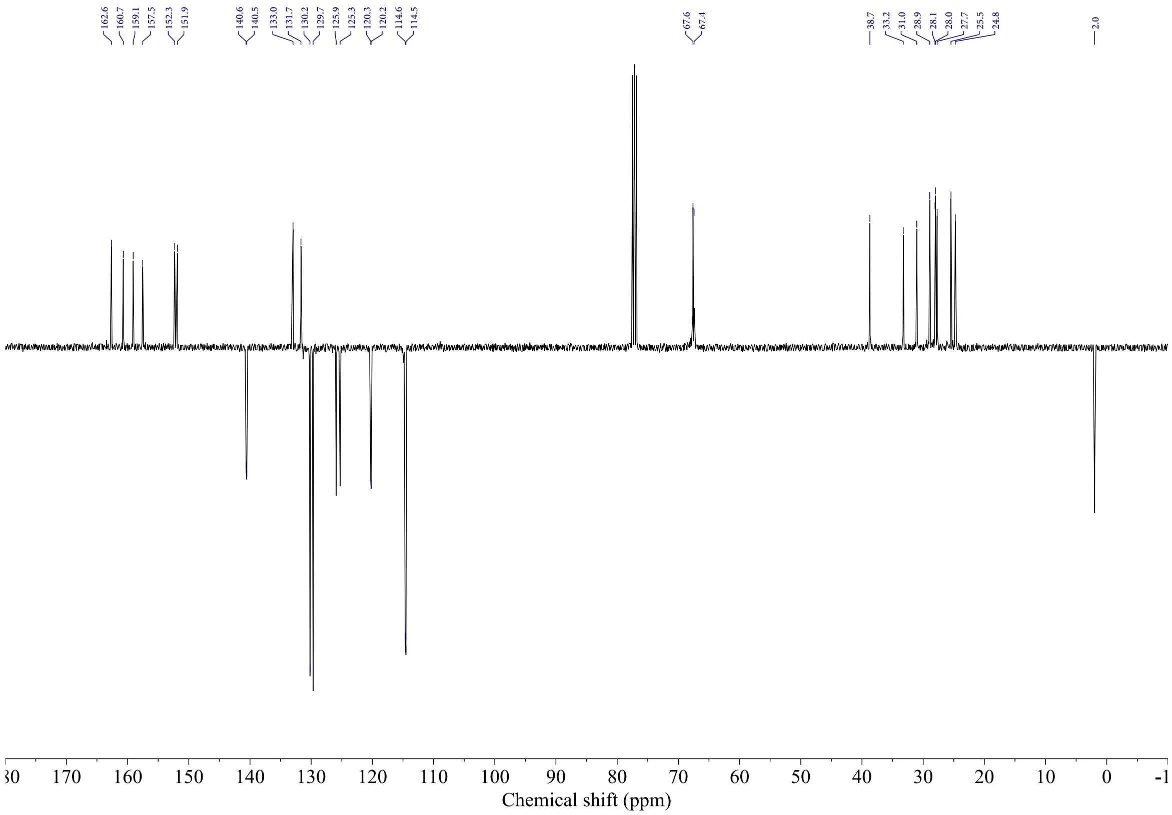

Figure S106. JMOD NMR of $[Cu(CH_3CN)_2(\mathbf{2})]PF_6$ ($CDCl_3$, 101 MHz, 298 K)

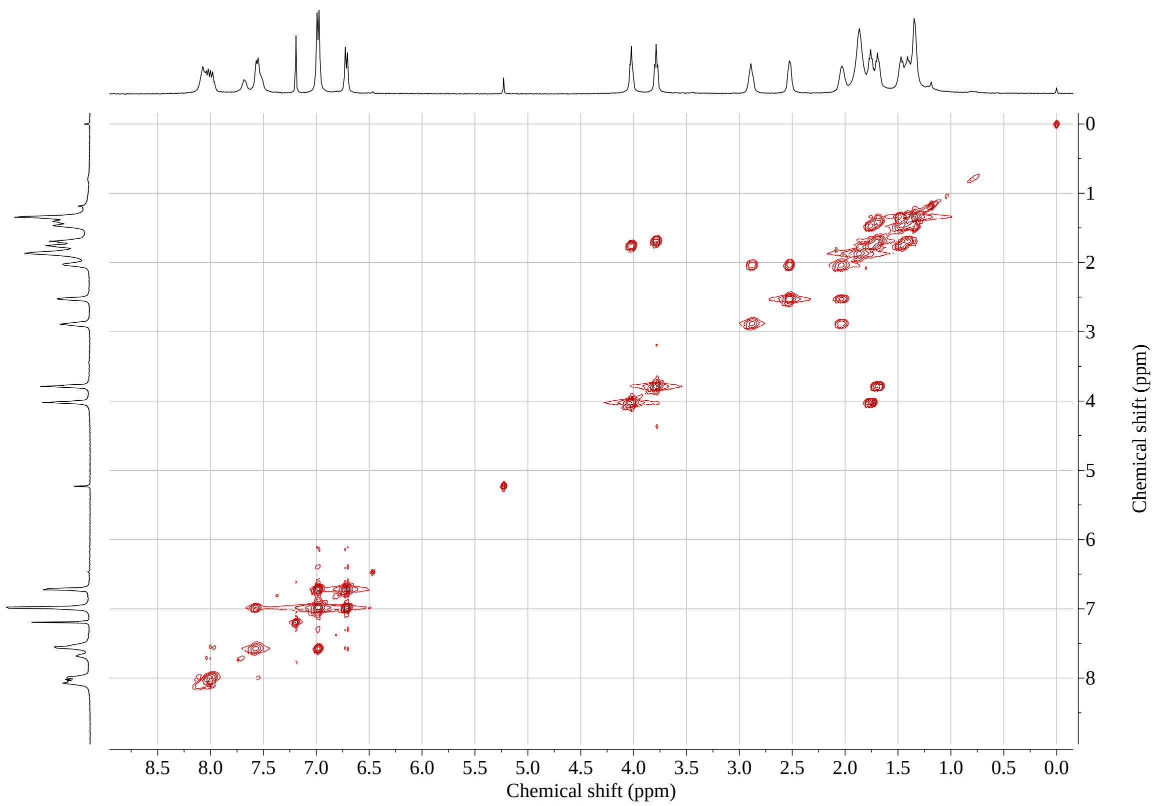

Figure S107. COSY NMR of $[Cu(CH_3CN)_2(\mathbf{2})]PF_6$ ($CDCl_3$, 298 K)

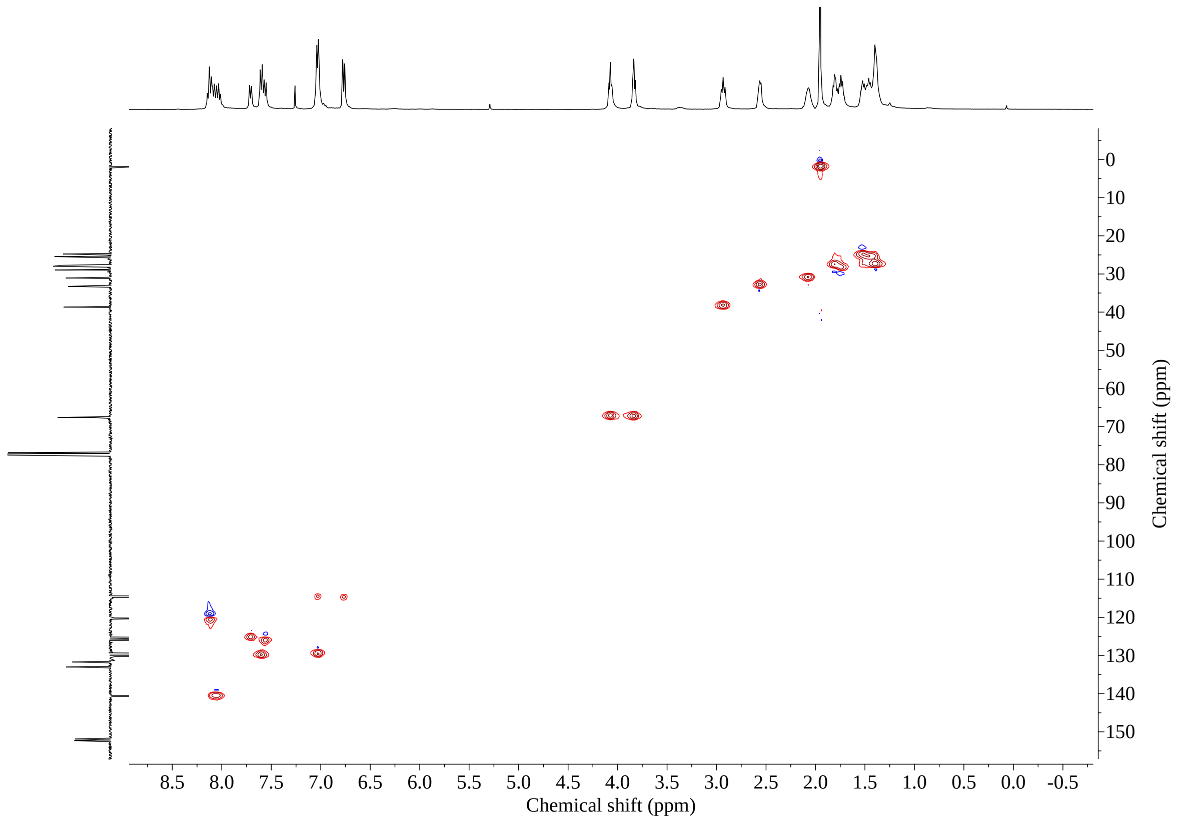

Figure S108. HSQC NMR of $[Cu(CH_3CN)_2(\mathbf{2})]PF_6$ ($CDCl_3$, 298 K)

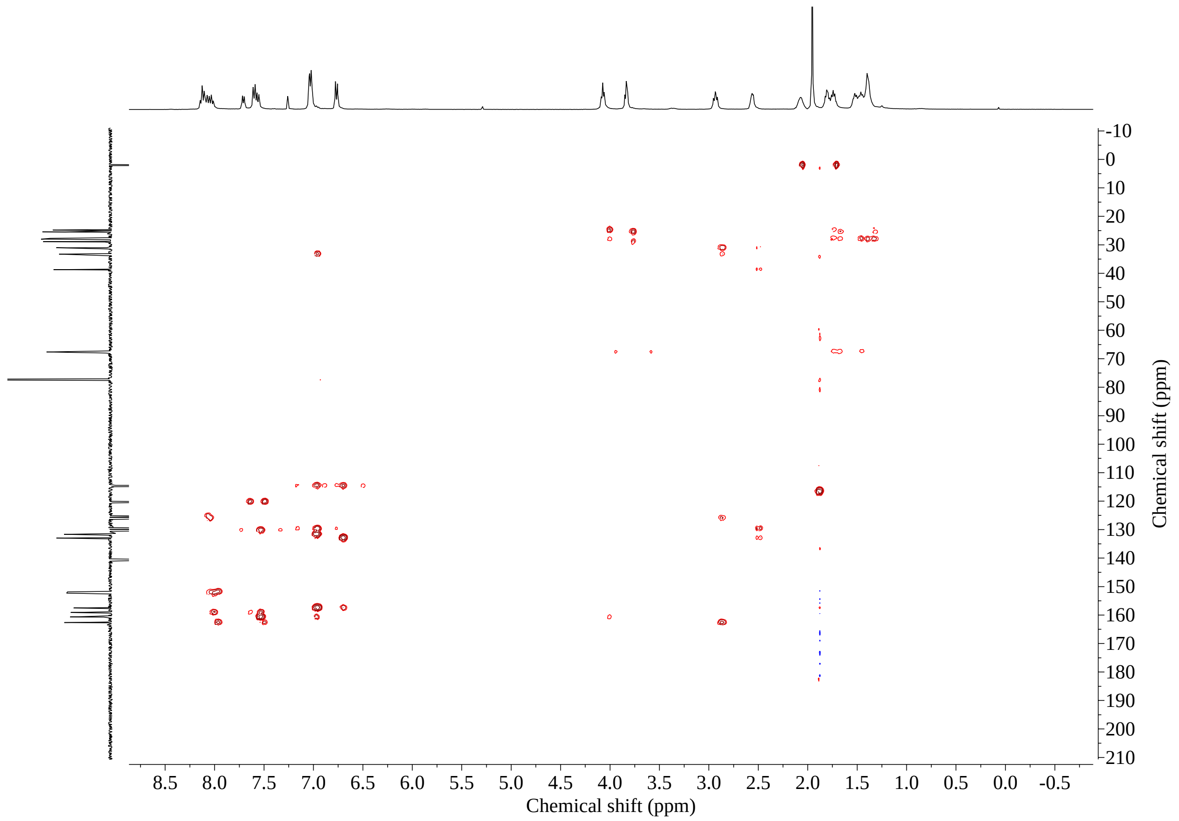

Figure S109. HMBC NMR of [Cu(CH_3CN)$_2$(**2**)]PF_6 ($CDCl_3$, 298 K)

Synthesis of catenanes 3

Catenane (*S*,S_{mt})-3a

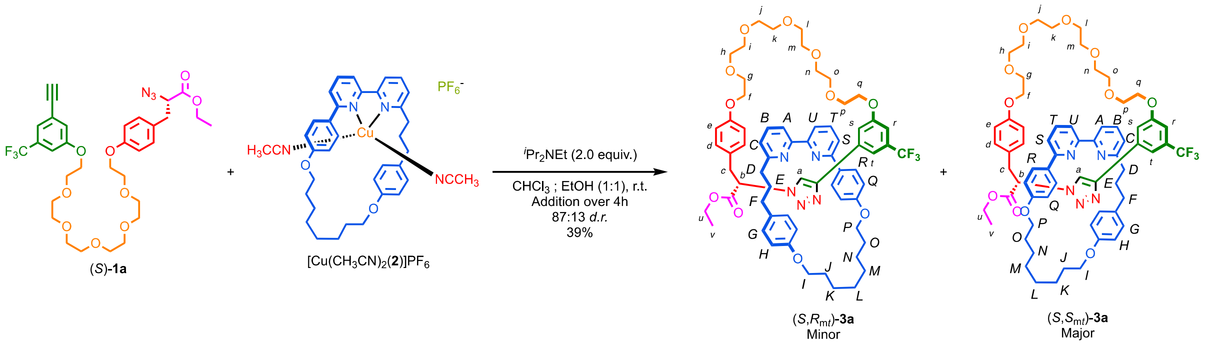

To a solution of [Cu(CH_3CN)$_2$(**2**)]PF_6 (34 mg, 0.046 mmol, 1.0 eq.), ^{i}Pr$_2$NEt (16 µL, 0.092 mmol, 2.0 eq.) in $CHCl_3$-EtOH (1 : 1, 4.6 mL) at rt was added a solution of (*S*)-**1a** (31 mg, 0.046 mmol, 1.0 eq.) in $CHCl_3$-EtOH (1 : 1, 1.8 mL) over 4 h. Once the addition had finished, H_2O (20 mL) was added followed by KCN (30 mg, 0.46 mmol, 10 equiv.) and the mixture stirred at rt for 16 h. The phases were separated, and the aqueous layer was extracted with $CHCl_3$ (10 mL). The combined organic extracts were dried ($MgSO_4$) and the solvent was removed *in vacuo*. Column chromatography (petrol-CH_2Cl_2-CH_3CN 40 : 40 : 20 → 30 : 30 :40 → 15 : 15 : 60) gave catenane **3a** as a yellow oil (21 mg, 39%, 87 : 13 *dr*). The major isomer was assigned as (*S*,S_{mt})-**3a** by analogy with (*S*, S_{mt})-**3b** (see section S7).

^{1}H NMR (500 MHz, $CDCl_3$, 298 K) δ: 9.07 (s, 1H, minor H_a), 8.98 (s, 1H, major H_a), 7.85 (t, *J* = 7.7, 1H, H_B), 7.74 (t, *J* = 7.8, 1H, H_T), 7.68 (dd, *J* = 7.7, 1.0, 1H, H_A), 7.61 (s, 1H, H_s), 7.52 (dd, *J* = 7.8, 0.9, 1H, H_U), 7.42 (s, 1H, H_t), 7.36 (dd, *J* = 7.8, 0.9, 1H, H_S), 7.35 (dd, *J* = 7.8, 0.9, 1H, H_C), 6.76 (d, *J* = 8.6, 2H, H_d), 6.71 (s, 1H, H_r), 6.47 (d, *J* = 8.7, 2H, H_R), 6.42 (d, *J* = 8.7, 2H, H_e), 6.39 (d, *J* = 8.4, 2H, H_G), 6.11 (d, *J* = 8.7, 2H, H_Q), 6.03 (d, *J* = 8.6, 2H, H_H), 5.46 (dd, *J* = 12.4, 3.3, 1H, H_b), 4.30-4.17 (m, 2H, H_u), 4.07-3.15 (m, 29H, H_c, H_f, H_g, H_h, H_i, H_j, H_k, H_l, H_m, H_n, H_o, H_p, H_q, H_I, H_P), 3.02 (dd, *J* = 15.9, 12.4, 1H, $H_{c'}$), 2.79 (td, *J* = 13.3, 4.4, 1H, H_D), 2.61-2.50 (m, 2H, $H_{D'}$, H_F), 2.33 (ddd, *J* = 14.2, 10.7, 4.1, 1H, $H_{F'}$), 1.95- 1.49 (m, 14H, H_E, H_J, H_K, H_L, H_M, H_N, H_O), 1.29 (t, *J* = 7.1, 3H, H_v).

^{19}F NMR (470 MHz, $CDCl_3$, 298 K) δ: -62.48 (s, 3F, major CF_3), 62.54 (s, 3F, minor CF_3).

^{13}C NMR (126 MHz, $CDCl_3$, 298 K) δ: 168.7, 164.0, 159.2, 159.0, 158.2, 157.8, 157.5, 157.5, 156.9, 146.6, 137.5, 137.1, 132.9, 131.8, 131.2, 129.8 (q, J_{C-F} = 32.0), 129.3, 128.9, 128.3, 127.1, 124.3 (app. d [outlying signals too weak to observe], J_{C-F} = 272.7), 123.9, 122.5, 120.1, 119.8, 116.1 (app. d [outlying signals obscured by line width], J_{C-F} = 3.3), 115.5, 115.1, 113.9, 113.6, 109.9 (app. d [outlying signals obscured by line width], J_{C-F} = 3.3), 71.0, 70.9, 70.8, 70.8, 70.7, 70.6, 70.4, 69.8, 69.1, 67.6, 67.5, 67.5, 66.8, 62.5, 62.2, 59.0, 37.5, 35.6, 35.0, 32.5, 29.8, 29.1, 28.9, 28.8, 25.6, 25.6, 24.1, 14.3.

LR-ESI-MS *m/z* = 1160.6 [M+H]$^+$ for $C_{65}H_{76}F_3N_5O_{11}$ (see isotope pattern, Figure S117)

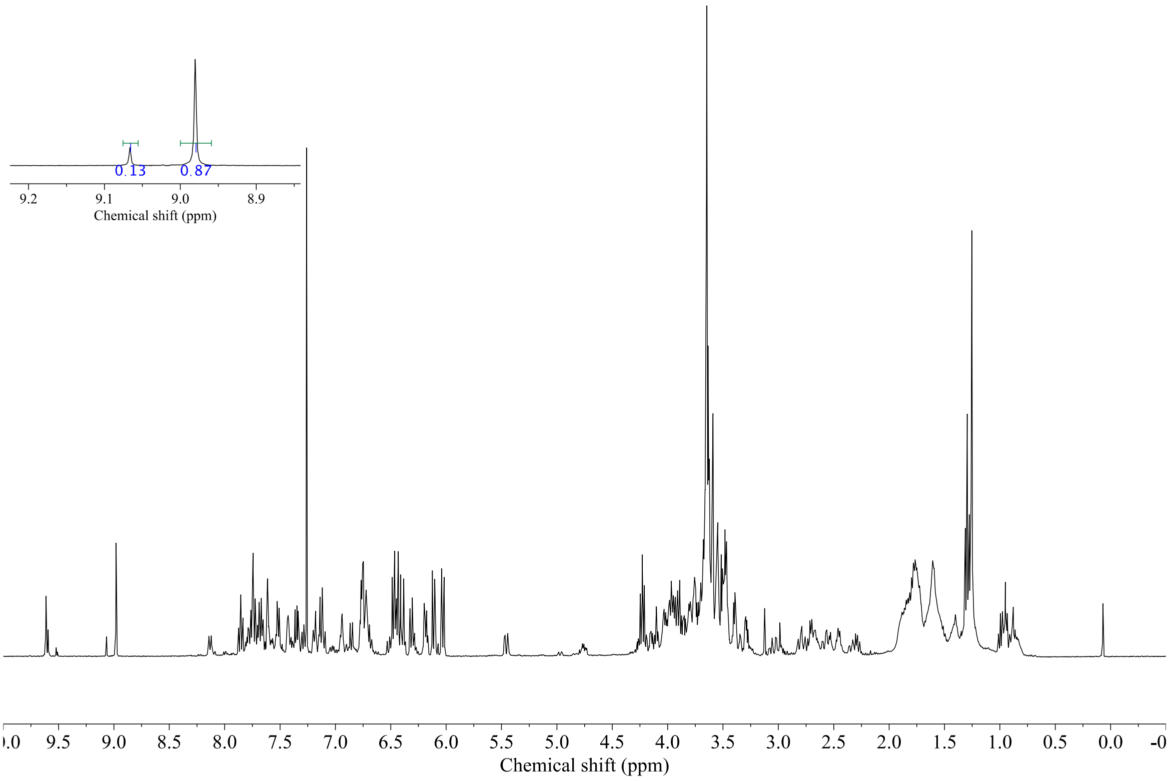

Figure S110. ^{1}H NMR of crude (S,S_{mt})-**3a** ($CDCl_3$, 400 MHz, 298 K)

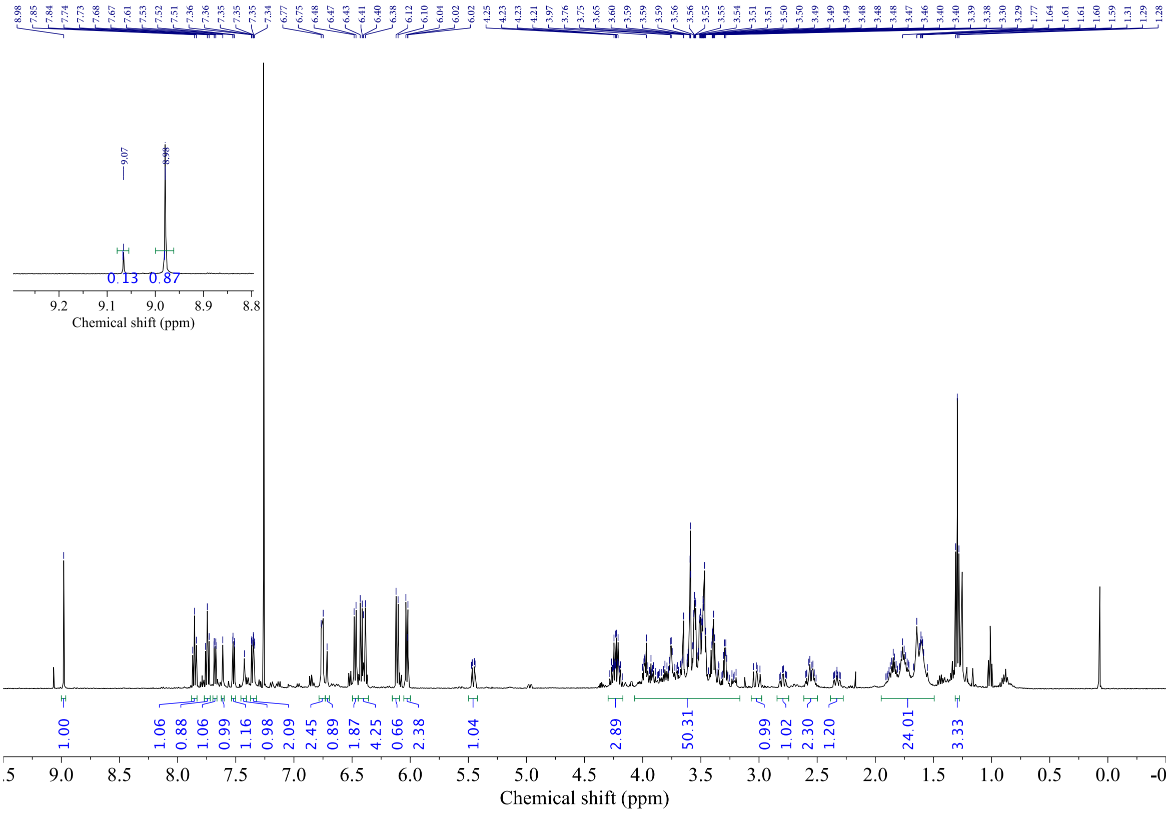

Figure S111. ^{1}H NMR of (S,S_{mt})-**3a** ($CDCl_3$, 500 MHz, 298 K)

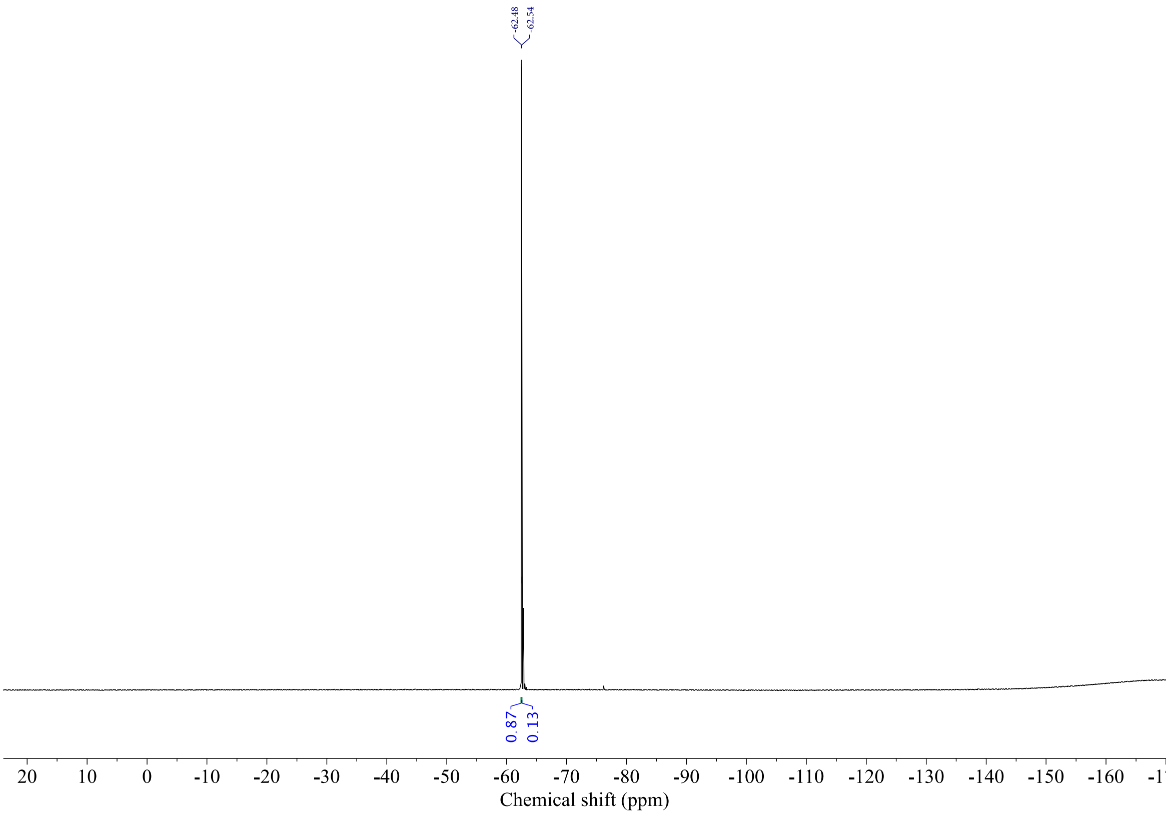

Figure S112. ^{19}F NMR of (S,S_{mt})-**3a** ($CDCl_3$, 470 MHz, 298 K)

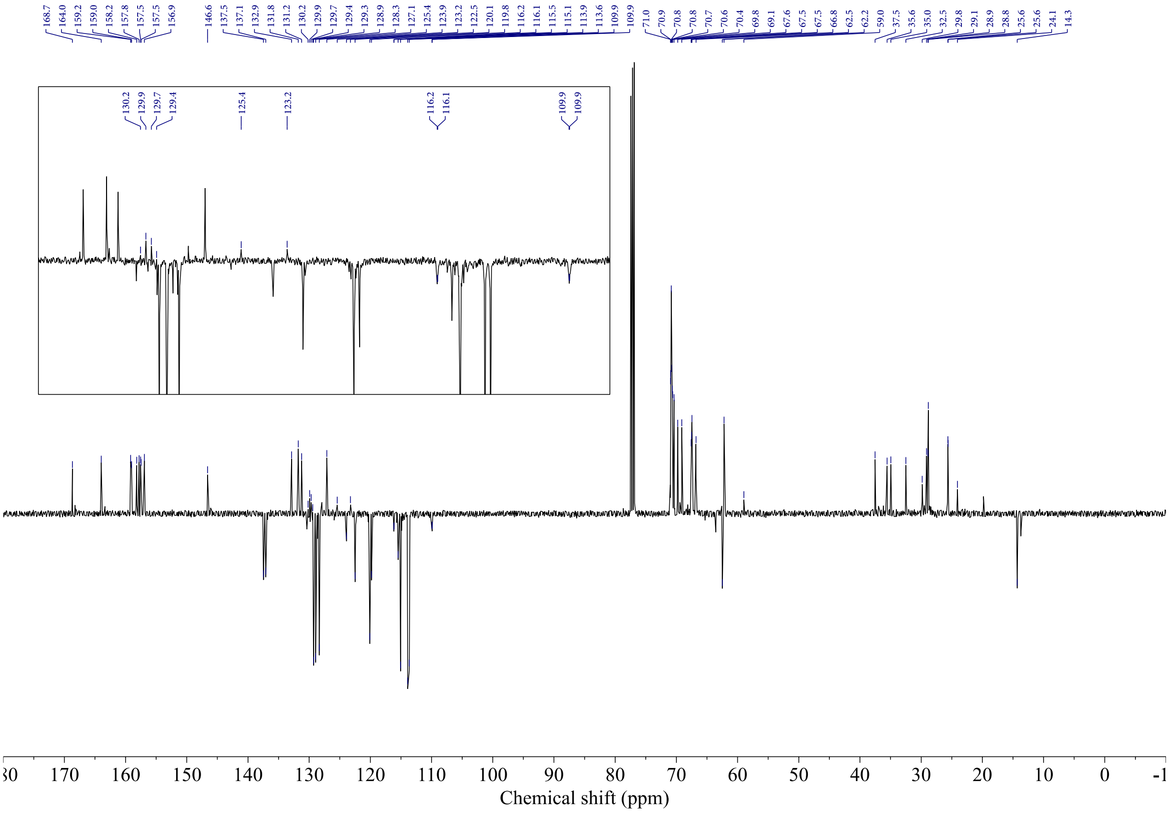

Figure S113. JMOD NMR of (S,S_{mt})-**3a** ($CDCl_3$, 126 MHz, 298 K)

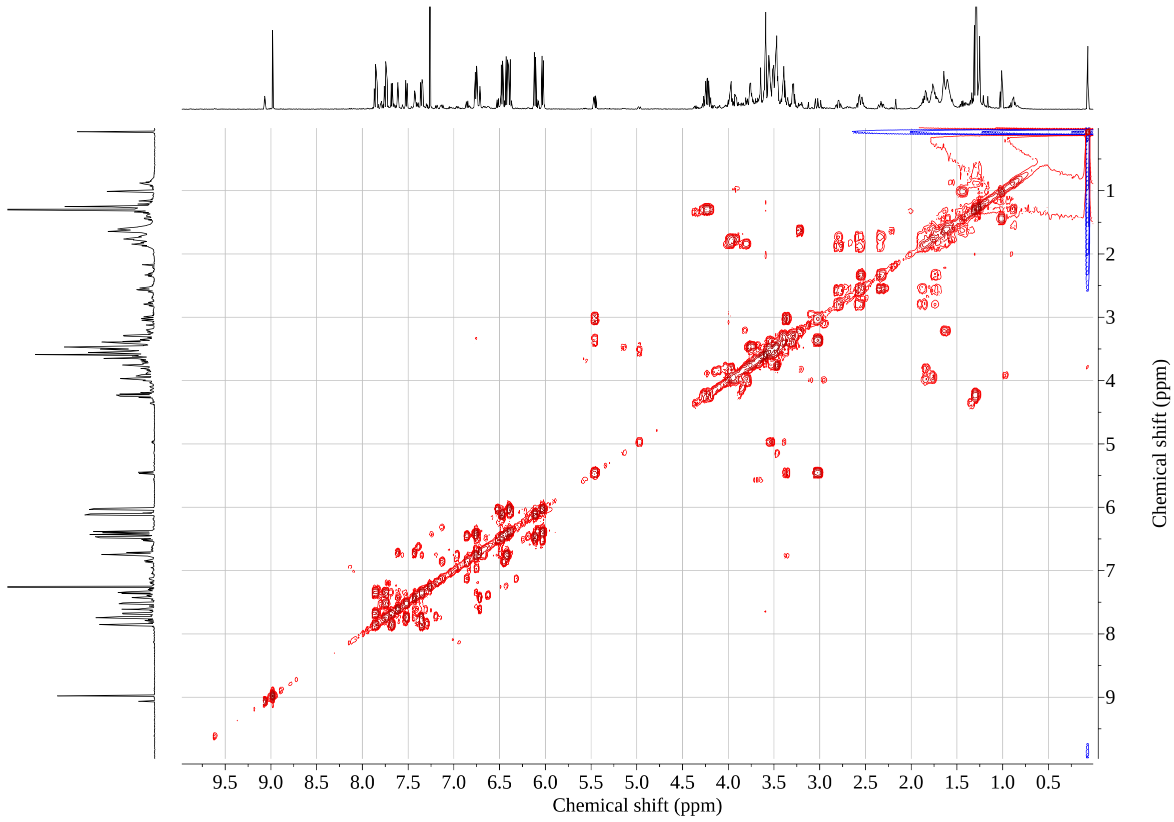

Figure S114. COSY NMR of (S,S_{mt})-**3a** ($CDCl_3$, 298 K)

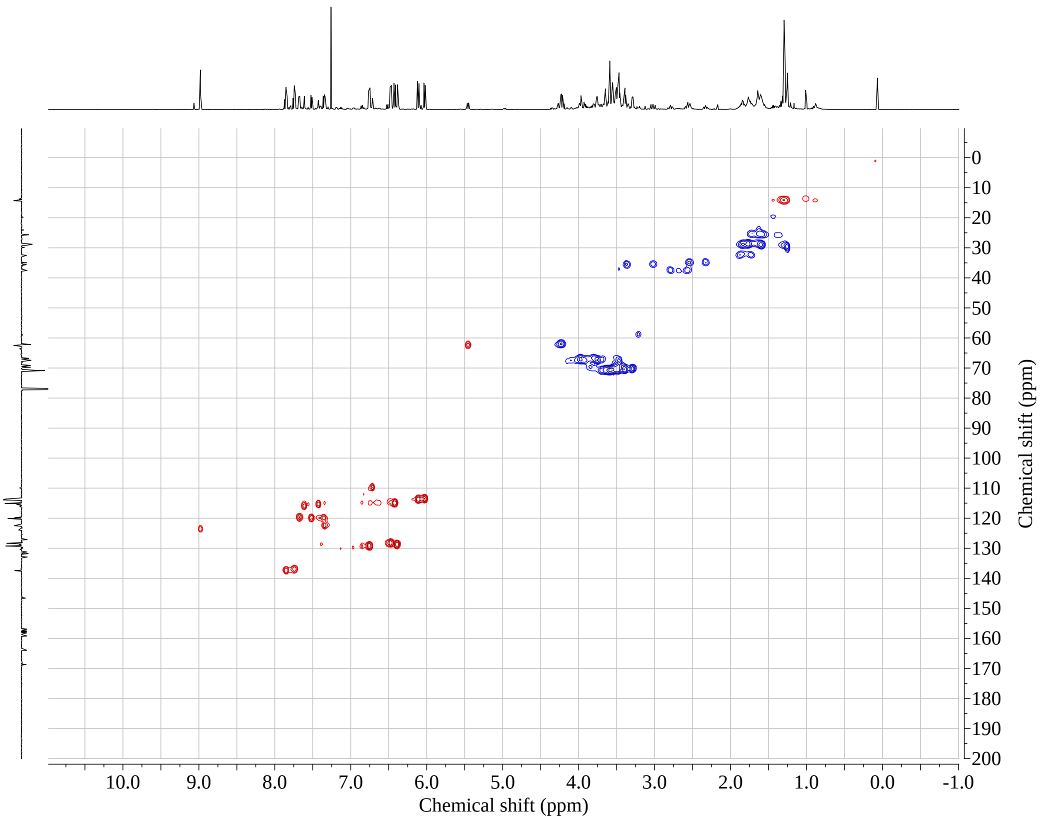

Figure S115. HSQC NMR of (S,S_{mt})-**3a** ($CDCl_3$, 298 K)

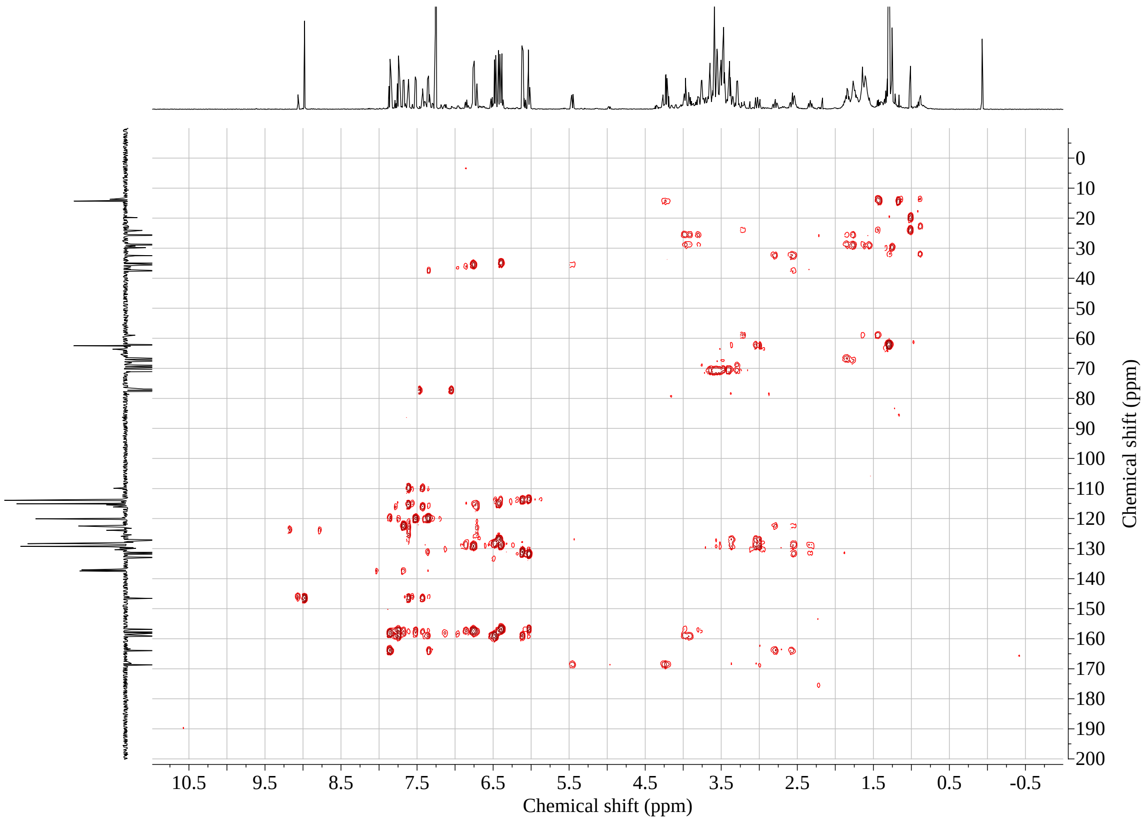

Figure S116. HMBC NMR of (S,S_{mt})-**3a** ($CDCl_3$, 298 K)

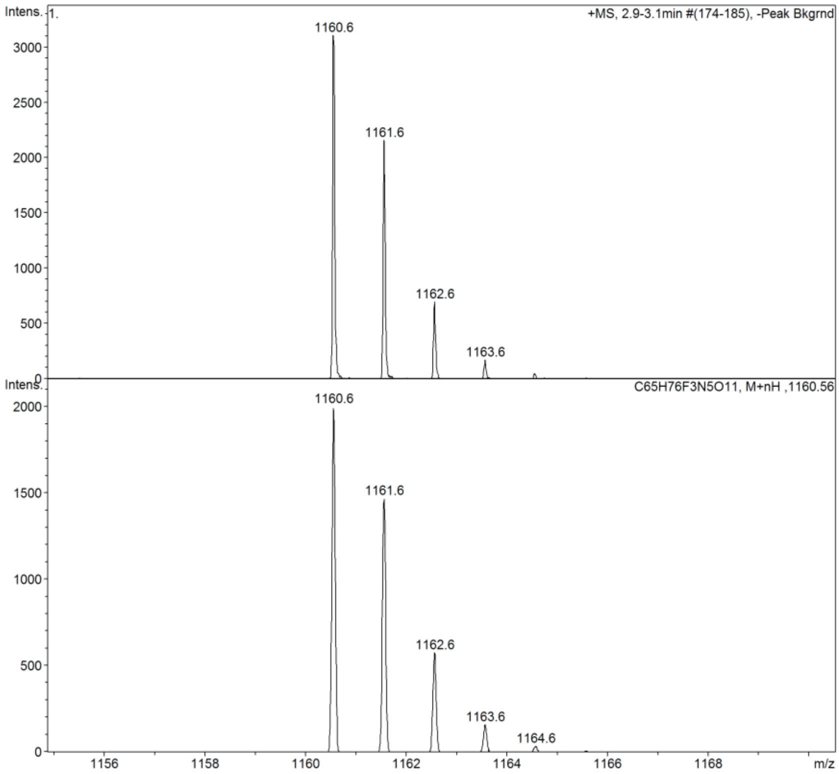

Figure S117. Isotope pattern of (S,S_{mt})-**3a** $C_{65}H_{76}F_3N_5O_{11}$

Catenane (*S*,*S*$_{mt}$)-3b

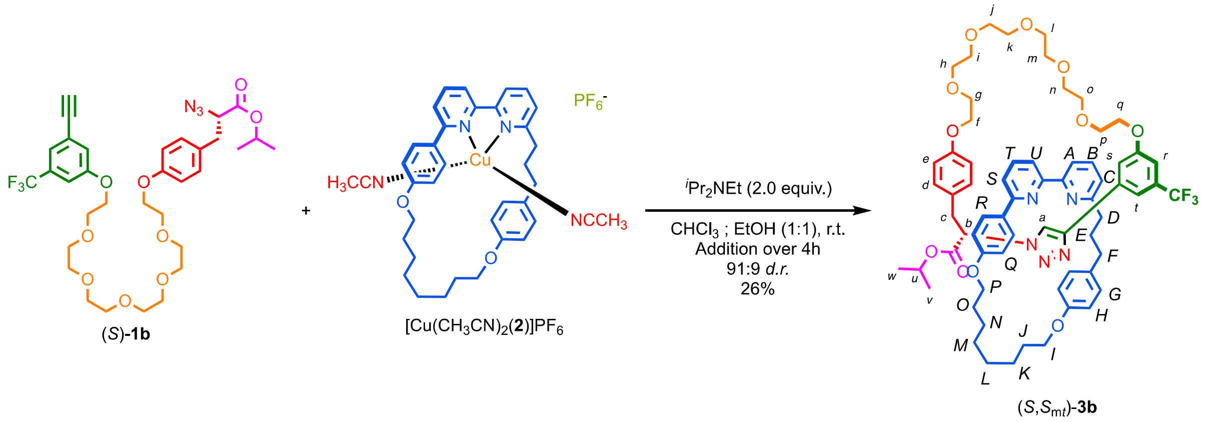

To a solution of $[Cu(CH_3CN)_2(\mathbf{2})]PF_6$ (108 mg, 0.14 mmol, 1.0 eq.), $^{i}Pr_2NEt$ (49µL, 0.28 mmol, 2.0 eq.) in $CHCl_3$-EtOH (1 : 1, 14 mL) at rt was added a solution of (*S*)-**1b** (100 mg, 0.14 mmol, 1.0 eq.) in $CHCl_3$-EtOH (1 : 1, 5.8 mL) over 4 h. Once the addition had finished, H_2O (20 mL) was added followed by KCN (91 mg, 1.4 mmol, 10 equiv.) and the mixture stirred at rt for 16 h. The phases were separated, and the aqueous layer was extracted with $CHCl_3$ (10 mL). The combined organic extracts were dried ($MgSO_4$) and the solvent was removed *in vacuo*. Column chromatography (petrol-acetone 90 : 10 → 60 : 40) gave catenane **3b** as a yellow oil (42 mg, 26%, 91 : 9 *dr*). The major isomer was assigned as (*S*,*S*$_{mt}$)-**3b** based on crystallographic data (see section S7). Non-interlocked macrocycle (*S*)-**S8** (12 mg, 12%) was also isolated as a by-product.

^{1}H NMR (400 MHz, $CDCl_3$, 298 K) δ: 9.01 (s, 1H, minor H_a), 8.94 (s, 1H, major H_a), 7.85 (t, *J* = 7.7, 1H, H_B), 7.74 (t, *J* = 7.8, 1H, H_T), 7.67 (d, *J* = 7.3, 1H, H_A), 7.60 (s, 1H, H_s), 7.51 (d, *J* = 8.2, 1H, H_U), 7.42 (s, 1H, H_t), 7.35 (d, *J* = 7.3, 1H, H_S), 7.34 (d, *J* = 7.5, 1H, H_C), 6.74 (d, *J* = 8.5, 2H, H_d), 6.70 (s, 1H, H_r), 6.48-6.34 (m, 6H, H_e, H_G, H_R), 6.11 (d, *J* = 8.7, 2H, H_Q), 6.02 (d, *J* = 8.6, 2H, H_H), 5.47 (dd, *J* = 12.3, 3.3, 1H, H_b), 5.09 (sept, *J* = 6.2, 1H, H_u), 4.08- 3.14 (m, 29H, H_c, H_f, H_g, H_h, H_i, H_j, H_k, H_l, H_m, H_n, H_o, H_p, H_q, H_I, H_P), 2.96 (dd, *J* = 15.9, 12.4, 1H, $H_{c'}$), 2.86 (td, *J* = 13.4, 4.4, 1H, H_D), 2.65-2.48 (m, 2H, $H_{D'}$, H_F), 2.40-2.26 (m, 1H, $H_{F'}$), 1.98-1.50 (m, 12H, H_J, H_K, H_L, H_M, H_N, H_O), 1.30 (d, *J* = 6.3, 3H, H_v), 1.28 (d, *J* = 6.2, 3H, H_w).

^{19}F NMR (376 MHz, $CDCl_3$, 298 K) δ: -62.46 (s, 3F, major CF_3), 62.52 (s, 3F, minor CF_3).

^{13}C NMR (101 MHz, $CDCl_3$, 298 K) δ 168.2, 164.1, 159.2, 159.0, 158.2, 157.7, 157.5, 157.4, 156.9, 146.6, 137.4, 137.0, 132.9, 131.8, 131.2, 129.7 (app. d [outlying signals too weak to observe], J_{C-F} = 32.0), 129.2, 129.0, 128.4, 127.1, 124.3 (app. d [outlying signals too weak to observe], J_{C-F} = 272.8), 123.8, 122.4, 120.1, 120.1, 119.8, 116.2 (app. d [outlying signals obscured by line width], J_{C-F} = 3.4), 115.5, 115.1, 113.9, 113.6, 109.8 (app. d [outlying signals obscured by line width], J_{C-F} = 2.8), 71.0, 70.9, 70.8, 70.8, 70.7, 70.7, 70.6, 70.3, 70.0, 69.8, 69.1, 67.6, 67.5, 67.4, 66.7, 62.4, 37.6, 35.6, 35.0, 32.6, 29.2, 29.1, 28.9, 28.8, 25.6, 25.6, 21.8, 21.8.

LR-ESI-MS *m/z* = 1174.6 $[M+H]^+$ for $C_{66}H_{78}F_3N_5O_{11}$ (see isotope pattern, Figure S125)

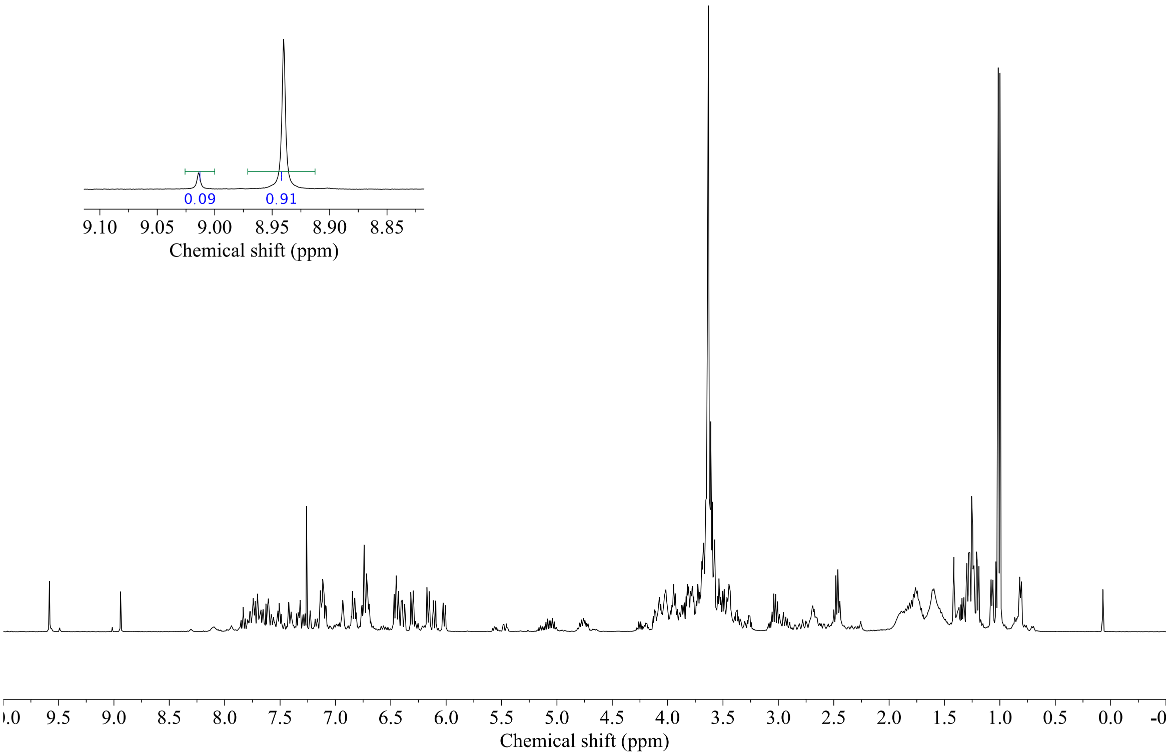

Figure S118. ^{1}H NMR of crude (*S*,*S*$_{mt}$)-**3b** ($CDCl_3$, 400 MHz, 298 K)

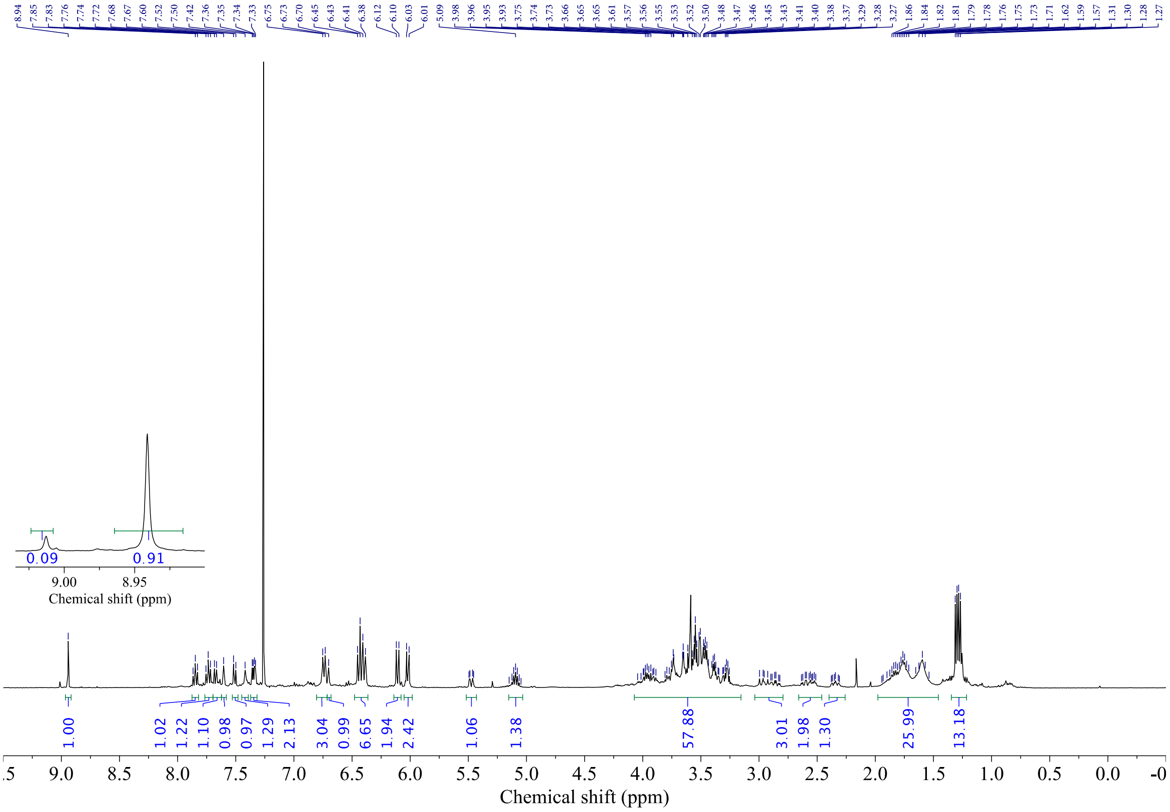

Figure S119. ^{1}H NMR of (*S*,*S*$_{mt}$)-**3b** ($CDCl_3$, 400 MHz, 298 K)

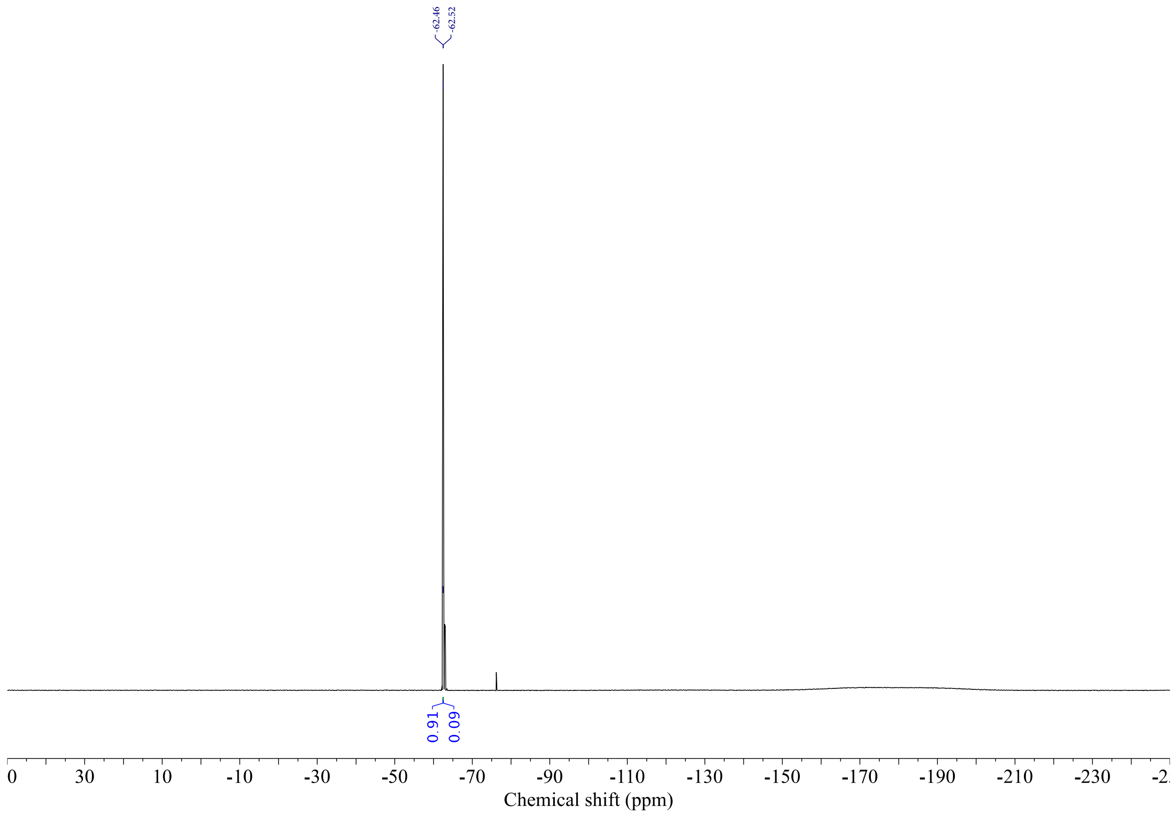

Figure S120. ^{19}F NMR of (*S*,*S*$_{mt}$)-**3b** ($CDCl_3$, 376 MHz, 298 K)

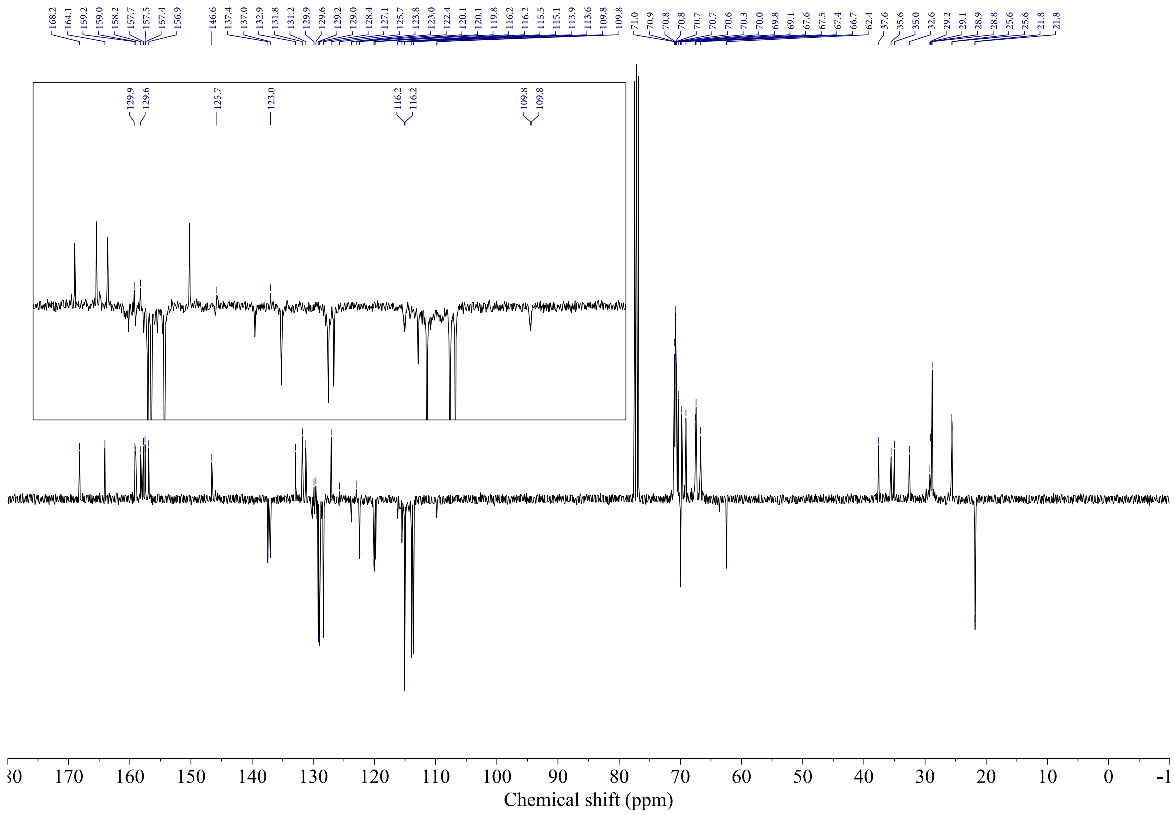

Figure S121. JMOD NMR of (*S*,*S*$_{mt}$)-**3b** ($CDCl_3$, 101 MHz, 298 K)

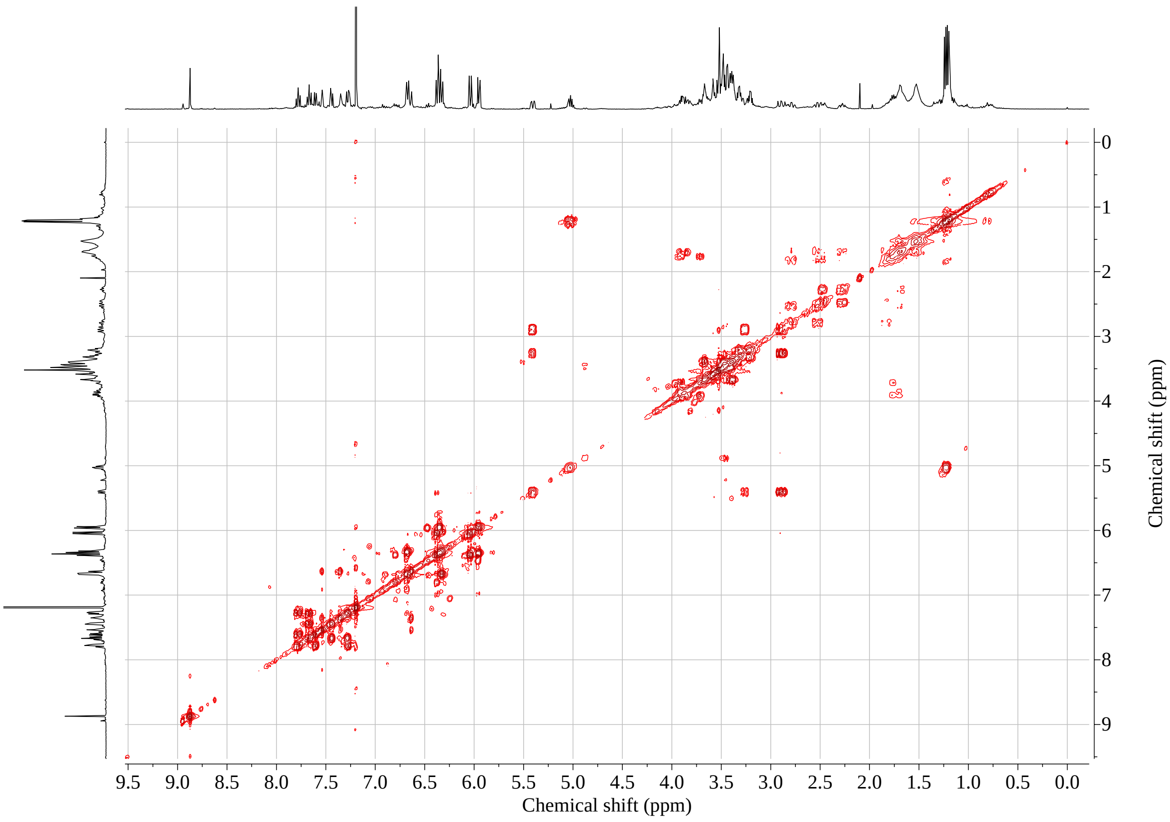

Figure S122. COSY NMR of (*S*,*S*$_{mt}$)-**3b** ($CDCl_3$, 298 K)

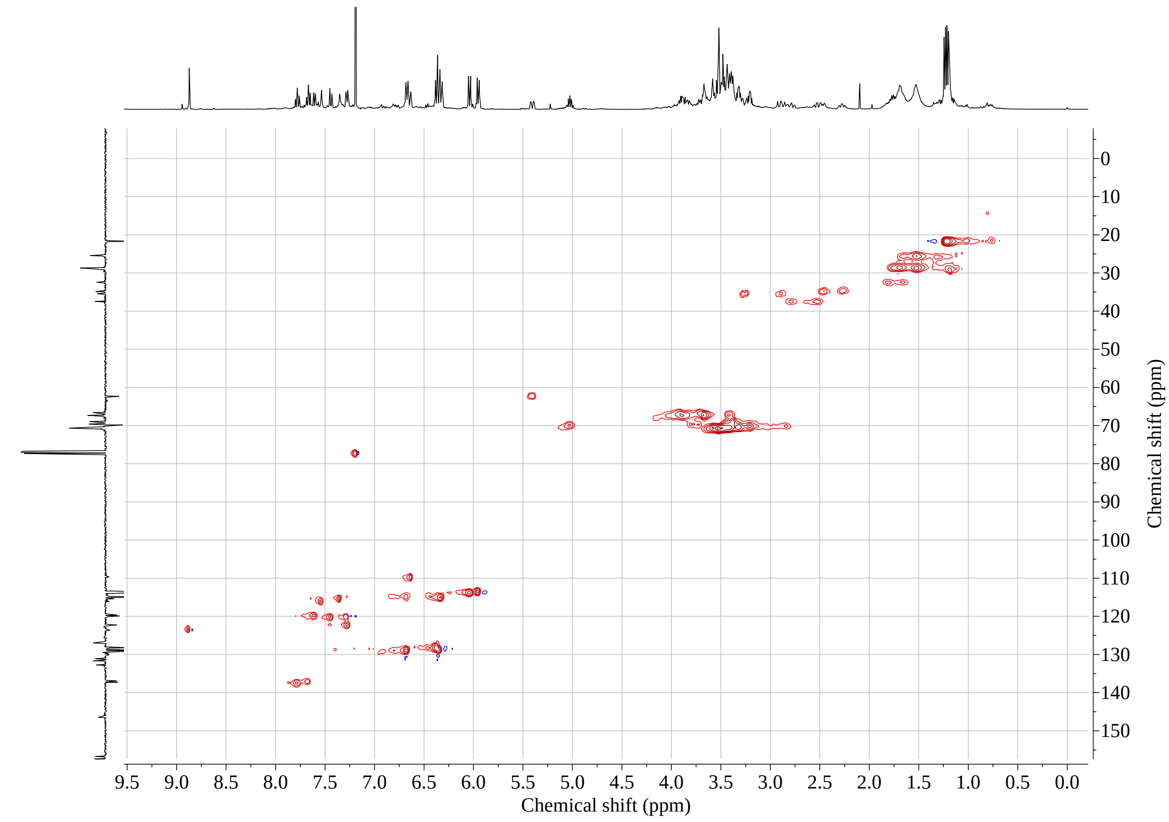

Figure S123. HSQC NMR of (*S*,*S*$_{mt}$)-**3b** ($CDCl_3$, 298 K)

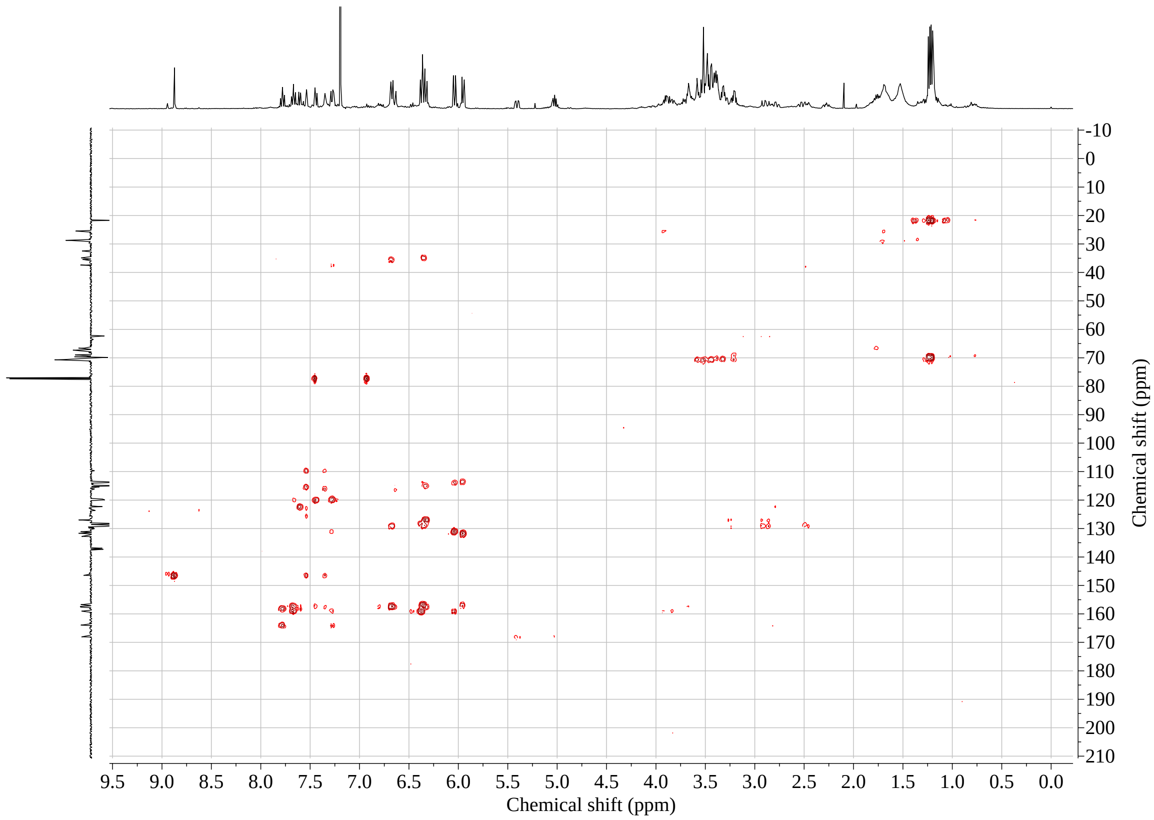

Figure S124. HMBC NMR of (S,S_{mt})-**3b** ($CDCl_3$, 298K)

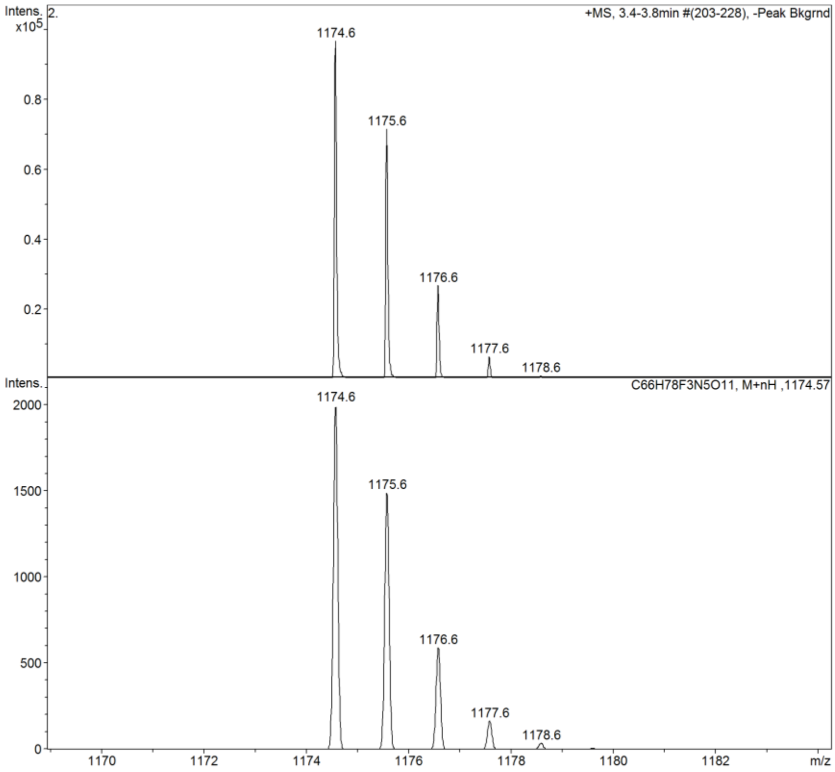

Figure S125. Isotope pattern of (S,S_{mt})-**3b** $C_{66}H_{78}F_3N_5O_{11}$

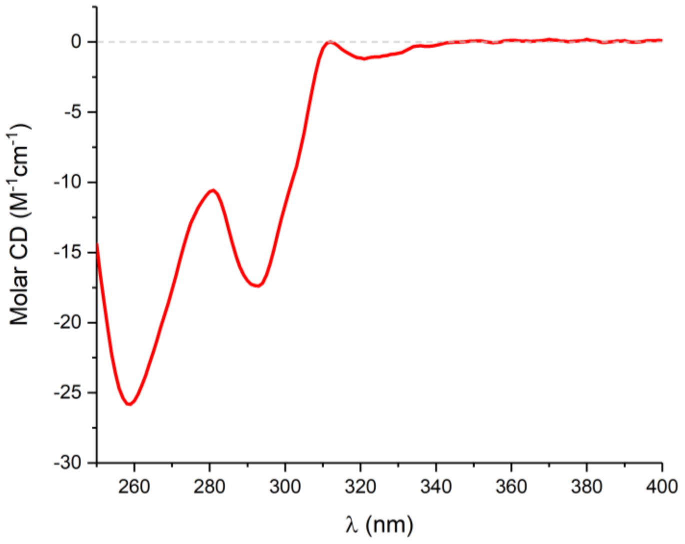

Figure S126. Circular Dichroism Spectra of (*S*,S_{mt})-**3b** (18.3 µM, *d.r.* (*S*,*S*$_{mt}$)-**3b** : (*S*, *R*$_{mt}$)-**3b** 91 : 9) at 293 K in $CHCl_3$

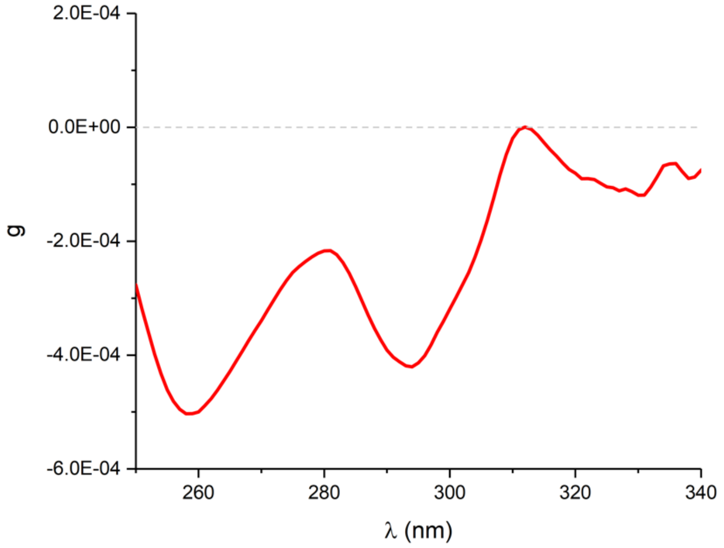

Figure S127. g_{abs} plot of (*S*,S_{mt})-**3b** (18.3 µM, *d.r.* (*S*,*S*$_{mt}$)-**3b** : (*S*, *R*$_{mt}$)-**3b** 91 : 9) at 293 K in $CHCl_3$. g_{abs}(max) = 5.0 × 10^{-4} (258 nm).

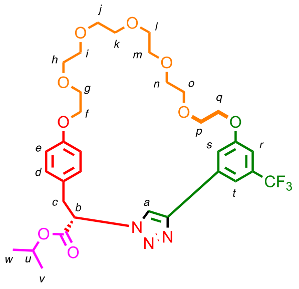

(S)-**S8**

^{1}H NMR (400 MHz, $CDCl_3$, 298 K) δ: 7.72-7.69 (m, 1H, H_t), 7.63 (s, 1H, H_a), 7.41 (t, J = 1.7, 1H, H_s), 7.11-7.08 (m, 1H, H_r), 6.84 (dt, J = 8.7, 2.5, 2H, H_d), 6.74 (dt, J = 8.7, 2.4, 2H, H_e), 5.58 (dd, J = 8.5, 5.4, 1H, H_b), 5.17 (sept., J = 6.5, 1H, H_u), 4.25-4.16 (m, 2H, H_q), 4.09-3.99 (m, 2H, H_f), 3.93-3.84 (m, 2H, H_p), 3.83-3.76 (m, 2H, H_g), 3.73-3.58 (m, 16H, H_h, H_i, H_j, H_k, H_l, H_m, H_n, H_o), 3.48 (dd, J = 14.5, 8.7, 1H, H_c), 3.43 (dd, J = 14.7, 5.1, 1H, $H_{c'}$), 1.35 (d, J = 6.3, 3H, H_v), 1.30 (d, J = 6.4, 3H, H_w).

^{19}F NMR (376 MHz, $CDCl_3$, 298 K) δ: -62.91 (s, 3F, CF_3).

^{13}C NMR (101 MHz, $CDCl_3$, 298 K) δ 167.6, 159.5, 158.4, 146.0, 132.9, 132.5 (q, J_{C-F} = 32.6), 130.2, 126.7, 124.0 (app. d [outlying signals too weak to observe], J_{C-F} = 272.5), 121.2, 115.2, 115.1 (app. s [only left hand signal of quartet observed - others obscured by neighbouring resonances), 115.0, 111.3 (q, J_{C-F} = 3.6), 71.2, 71.1, 70.9, 70.9, 70.9 (×2), 70.8, 70.8, 70.6 (from HSQC), 69.8, 69.7, 68.2, 67.6, 64.5, 38.1, 21.9, 21.8.

HR-ESI-MS m/z = 704.2769 $[M+Na]^+$ calc. 704.2765 for $C_{33}H_{42}F_3N_3NaO_9$.

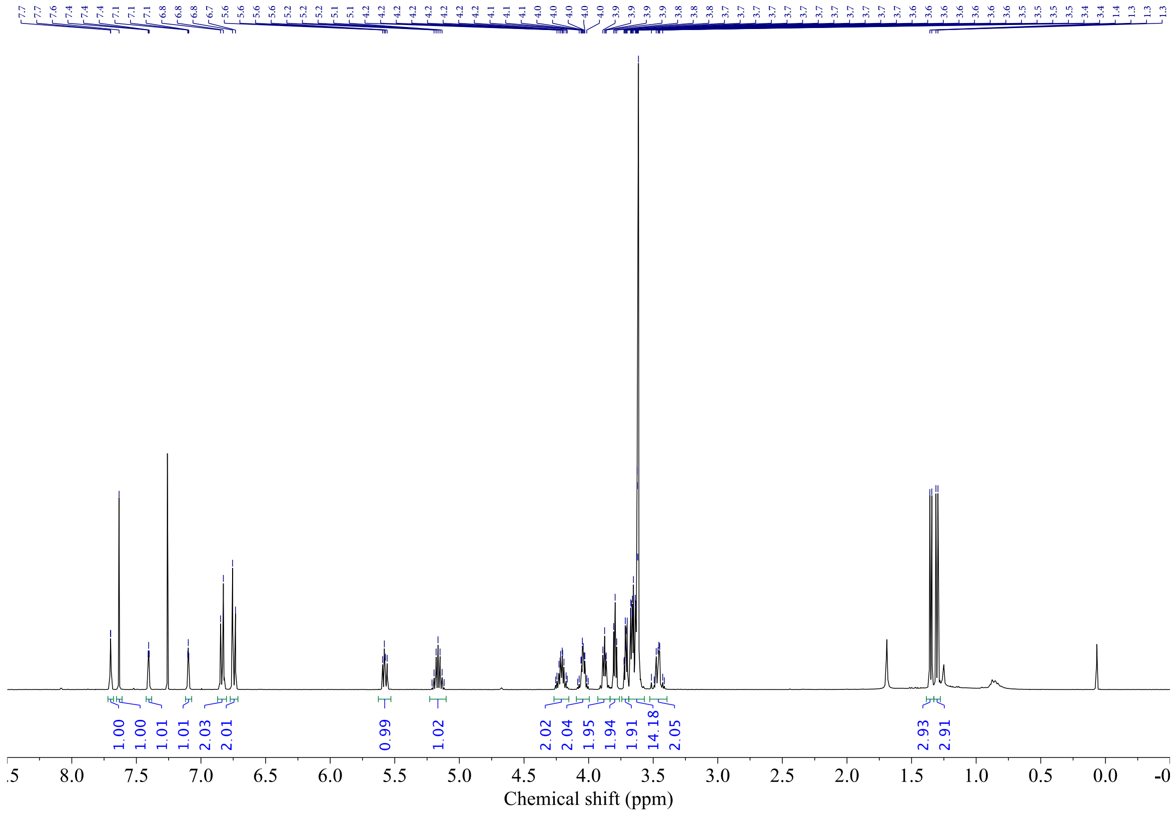

Figure S128. ^{1}H NMR of (S)-**S8** ($CDCl_3$, 400 MHz, 298 K)

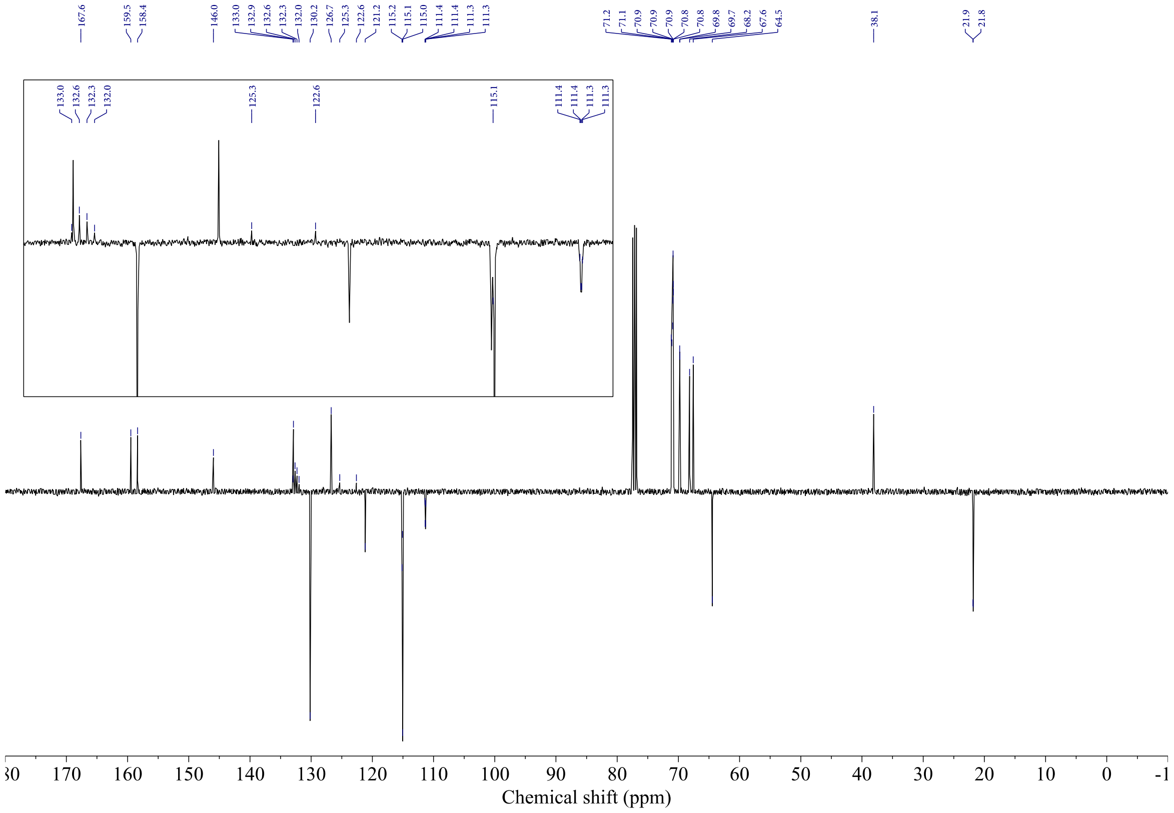

Figure S129. JMOD NMR of (*S*)-**S8** ($CDCl_3$, 101 MHz, 298 K)

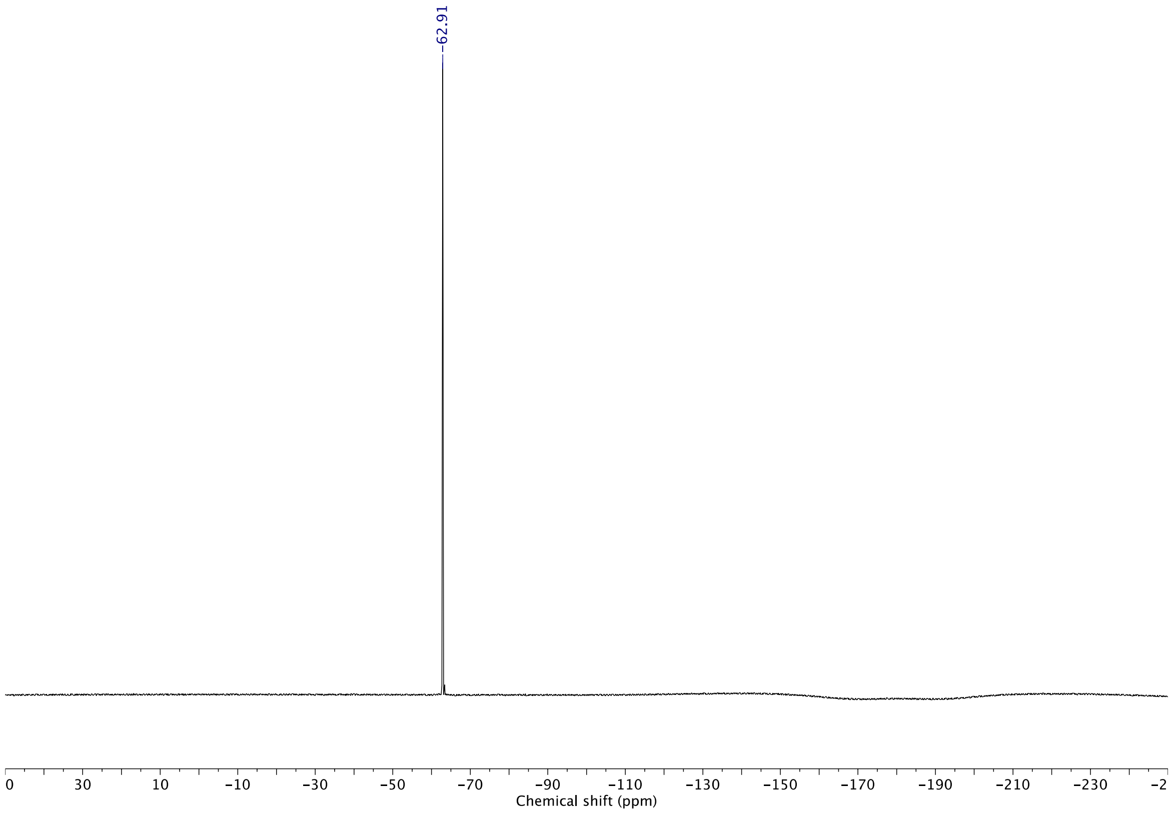

Figure S130. ^{19}F NMR of (*S*)-**S8** ($CDCl_3$, 376 MHz, 298 K)

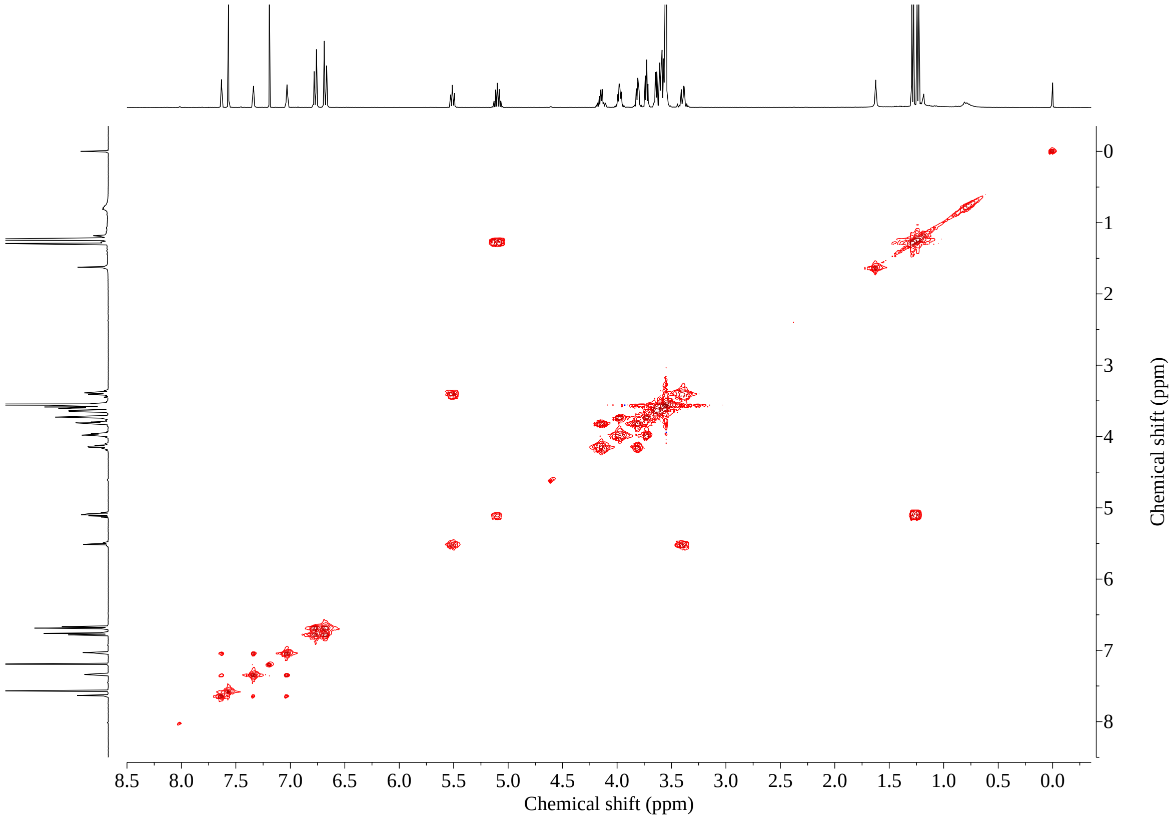

Figure S131. COSY NMR of (*S*)-**S8** ($CDCl_3$, 298 K)

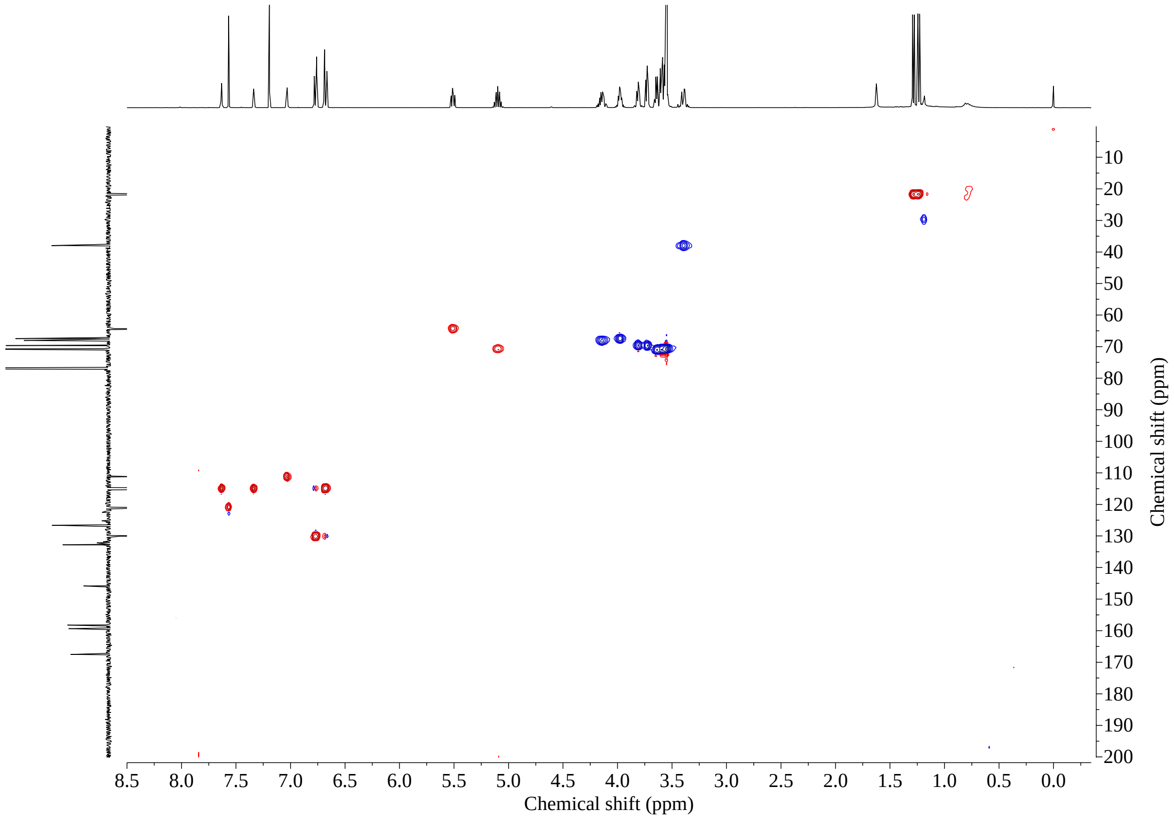

Figure S132. HSQC NMR of (*S*)-**S8** ($CDCl_3$, 298 K)

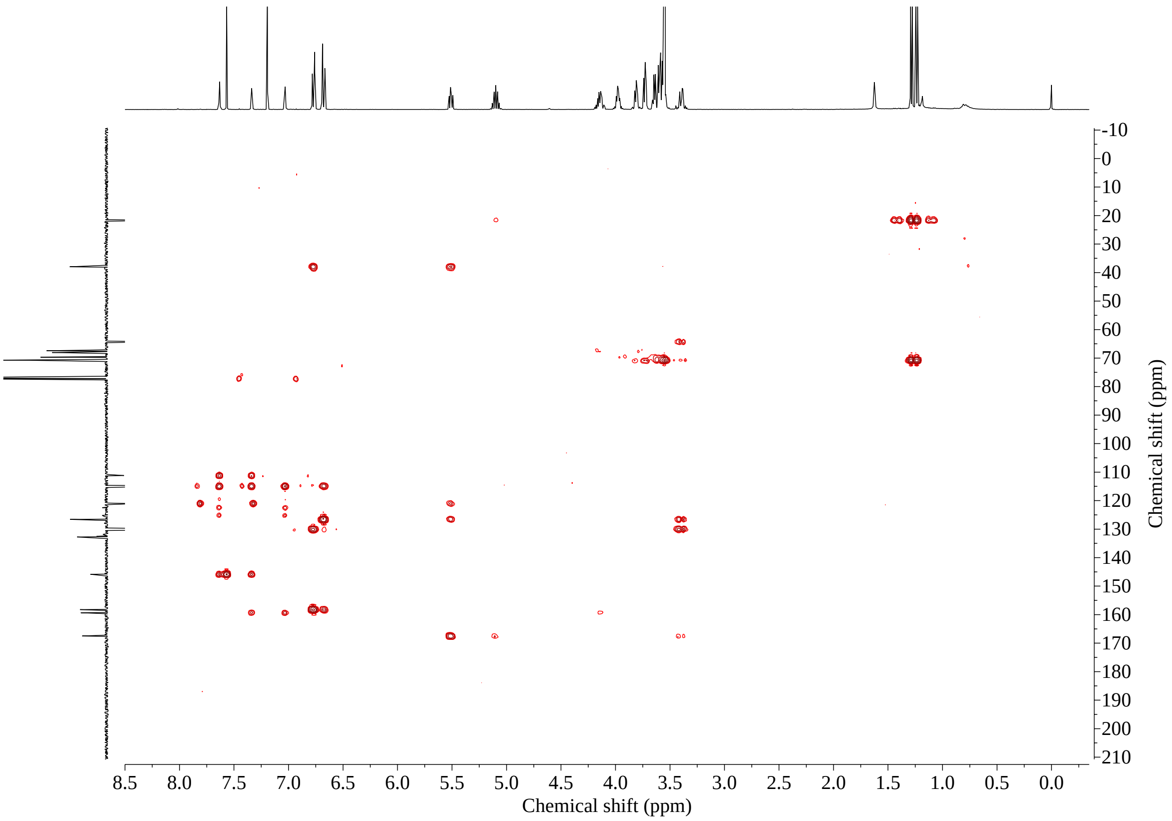

Figure S133. HMBC NMR of (*S*)-**S8** ($CDCl_3$, 298 K)

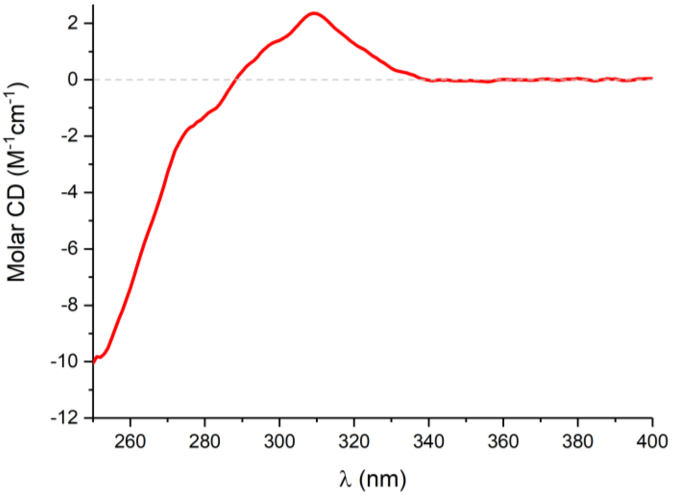

Figure S134. Circular dichroism spectrum of (*S*)-**S8** (45.5 µM) at 293 K in $CHCl_3$

Catenane (*S*,*S*$_{mt}$)-3c

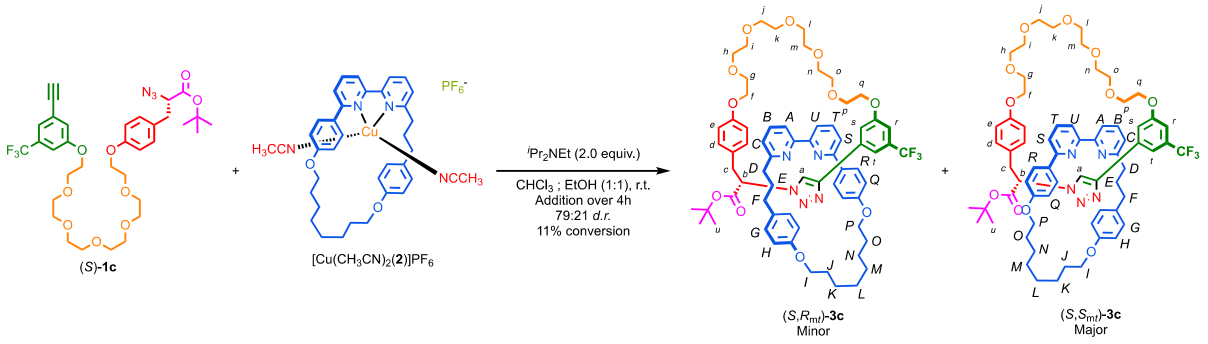

To a solution of [Cu(CH$_3$CN)$_2$(**2**)]PF$_6$ (7.4 mg, 0.01 mmol, 1.0 eq.), ^{i}Pr$_2$NEt (3.5 µL, 0.02 mmol, 2.0 eq.) in CHCl$_3$-EtOH (1 : 1, 1 mL) at rt was added a solution of (*S*)-**1c** (7 mg, 0.01 mmol, 1.0 eq.) in CHCl$_3$-EtOH (1 : 1, 0.4 mL) over 4 h. Once the addition had finished, H_2O (10 mL) was added followed by KCN (7 mg, 0.1 mmol, 10 equiv.) and the mixture stirred at rt for 16 h. The phases were separated, and the aqueous layer was extracted with CHCl$_3$ (10 mL). The combined organic extracts were dried (MgSO$_4$) and the solvent was removed *in vacuo*. ^{1}H NMR analysis of the crude indicated 77% **2** remained unreacted alongside 11% **3c** (84 : 16 *dr*) and 12% by-products **S28c** and **4c**. We were unable to isolate catenane **3c** from the mixture.

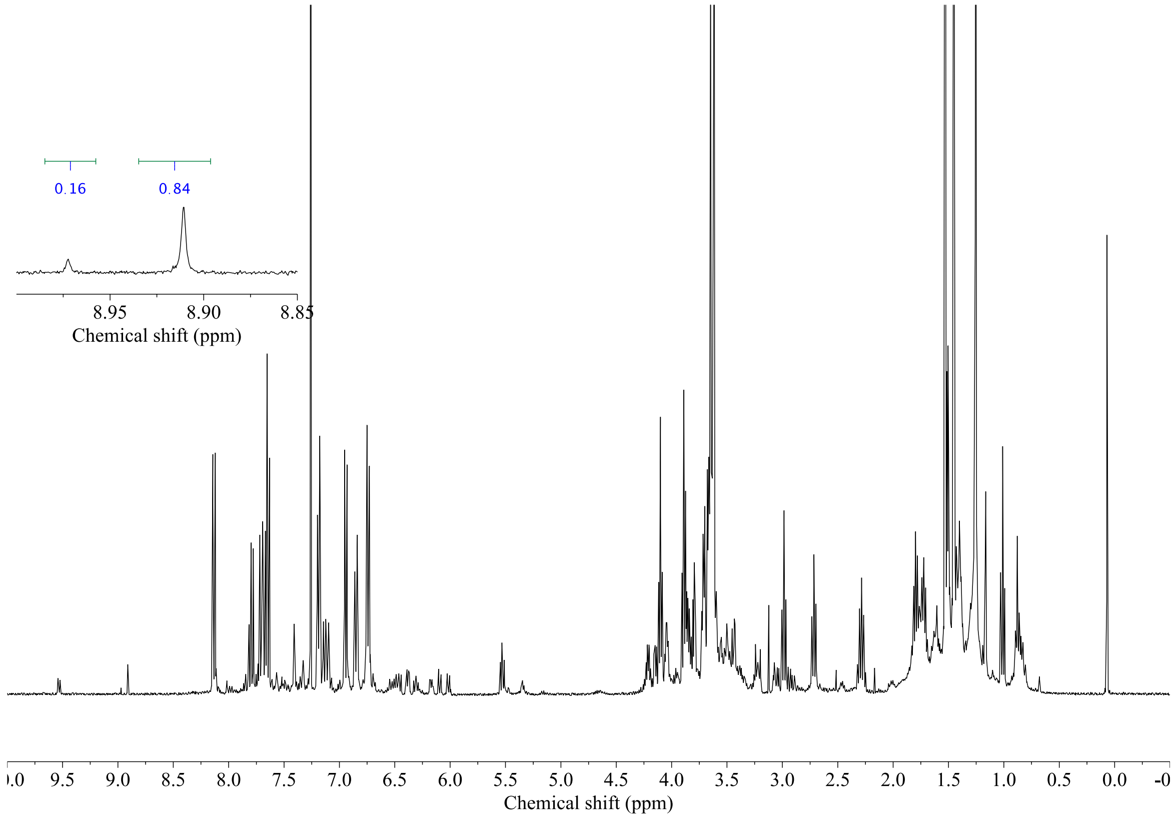

Figure S135. ^{1}H NMR of crude (*S*,S$_{mt}$)-**3c** (CDCl$_3$, 400 MHz, 298 K)

S3. SYNTHESIS OF CATENANE 6 (SCHEME 2, MAIN TEXT)

Synthesis of *rac*-6 precursor S13

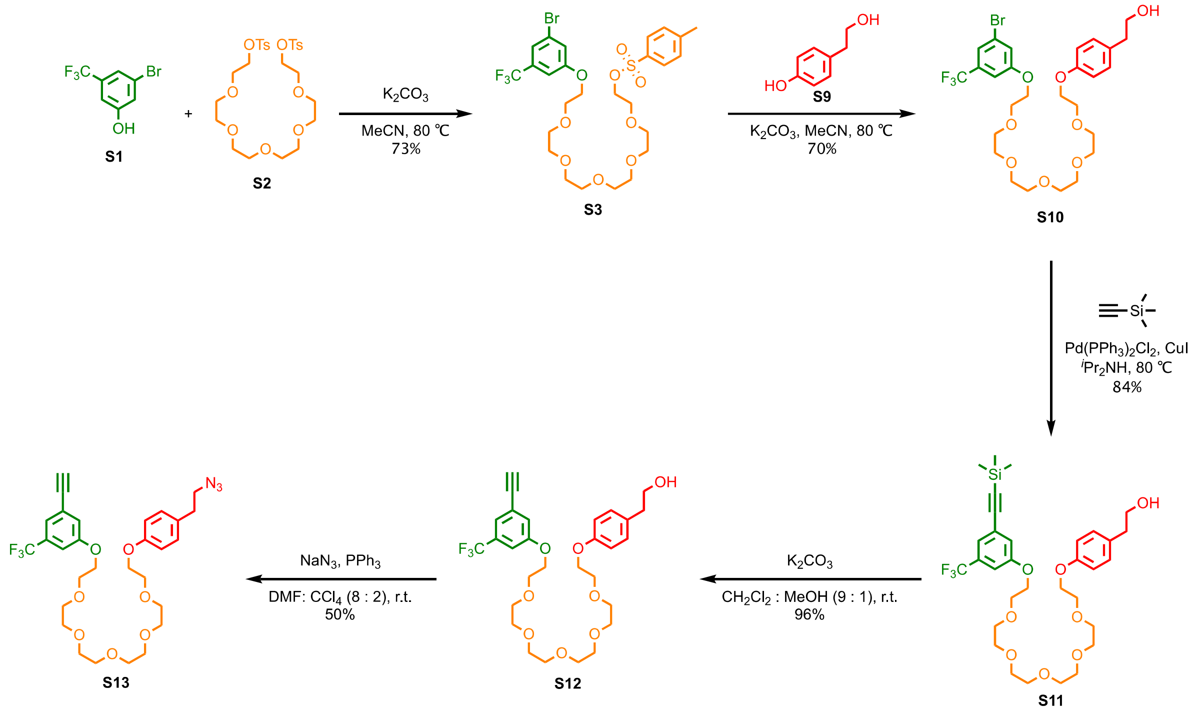

Scheme S2. Synthetic route to macrocycle precursor **S13** for the synthesis of *rac*-**6**

Alcohol S10

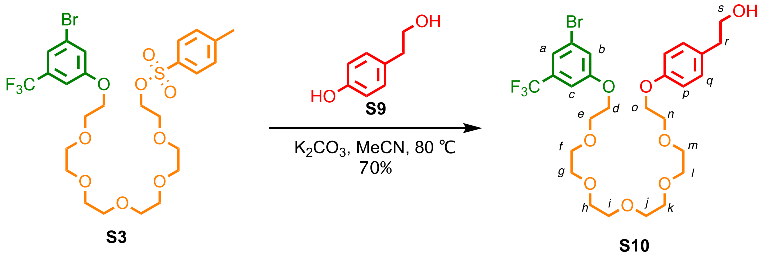

S3 (354 mg, 0.54 mmol, 1.0 equiv.), **S9** (374 mg, 0.54 mmol, 1.0 equiv.) and K_2CO_3 (300 mg, 2.16 mmol, 4.0 equiv.) were suspended in CH_3CN (5 mL) and the resulting suspension was heated at reflux for 48 h. The reaction mixture was filtered over a Celite® pad, which was washed with CH_2Cl_2 (20 mL). The washings were combined, and the solvent was removed *in vacuo*. Column chromatography (petrol-Et_2O-CH_3CN 20 : 80 : 0 → 0 : 100 : 0 → 0 : 90 : 10) gave **S10** as a pale yellow oil (238 mg, 70%).

^{1}H NMR (400 MHz, $CDCl_3$, 298 K) δ 7.34-7.32 (m, 1H, H_a), 7.24 (t, J = 2.1, 1H, H_b), 7.14-7.08 (m, 3H, H_c, H_q), 6.85 (d, J = 8.5, 2H, H_p), 4.16-4.11 (m, 2H, H_d), 4.11-4.07 (m, 2H, H_o), 3.87-3-77 (m, 6H, H_n, H_e, H_s), 3.73-3.61 (m, 16H, H_f, H_g, H_h, H_i, H_j, H_k, H_l, H_m) 2.79 (t, J = 6.5, 2H, H_r), 1.67 (t, J = 5.3, 1H, OH).

^{19}F NMR (376 MHz, $CDCl_3$, 298 K) δ: -63.15 (s, 3F, CF_3).

^{13}C NMR (101 MHz, $CDCl_3$, 298 K) δ 159.8, 157.5, 133.0 (q, $J_{C\text{-}F}$ = 33.0), 130.8, 130.0, 123.2, 123.1 (q, $J_{C\text{-}F}$ = 273.0), 121.3, 120.8 (q, $J_{C\text{-}F}$ = 3.9), 114.8, 110.9 (q, $J_{C\text{-}F}$ = 3.8), 71.0, 70.9, 70.7 (×3), 70.7 (×2), 70.6, 69.8, 69.5, 68.3, 67.5, 63.8, 38.3.

HR-ESI-MS *m/z* = 647.1444 $[M+Na]^+$ calc. 647.1438 for $C_{27}H_{36}BrF_3NaO_8$.

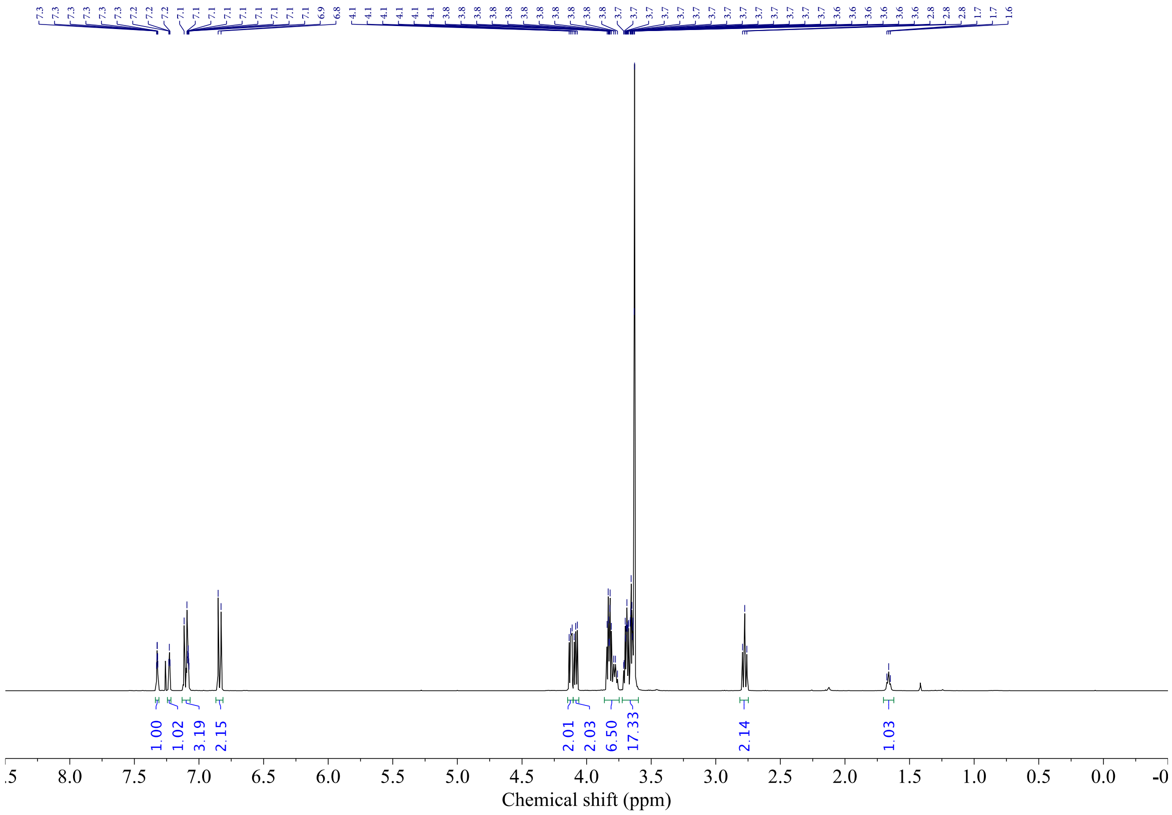

Figure S136. ^{1}H NMR of **S10** ($CDCl_3$, 400 MHz, 298 K)

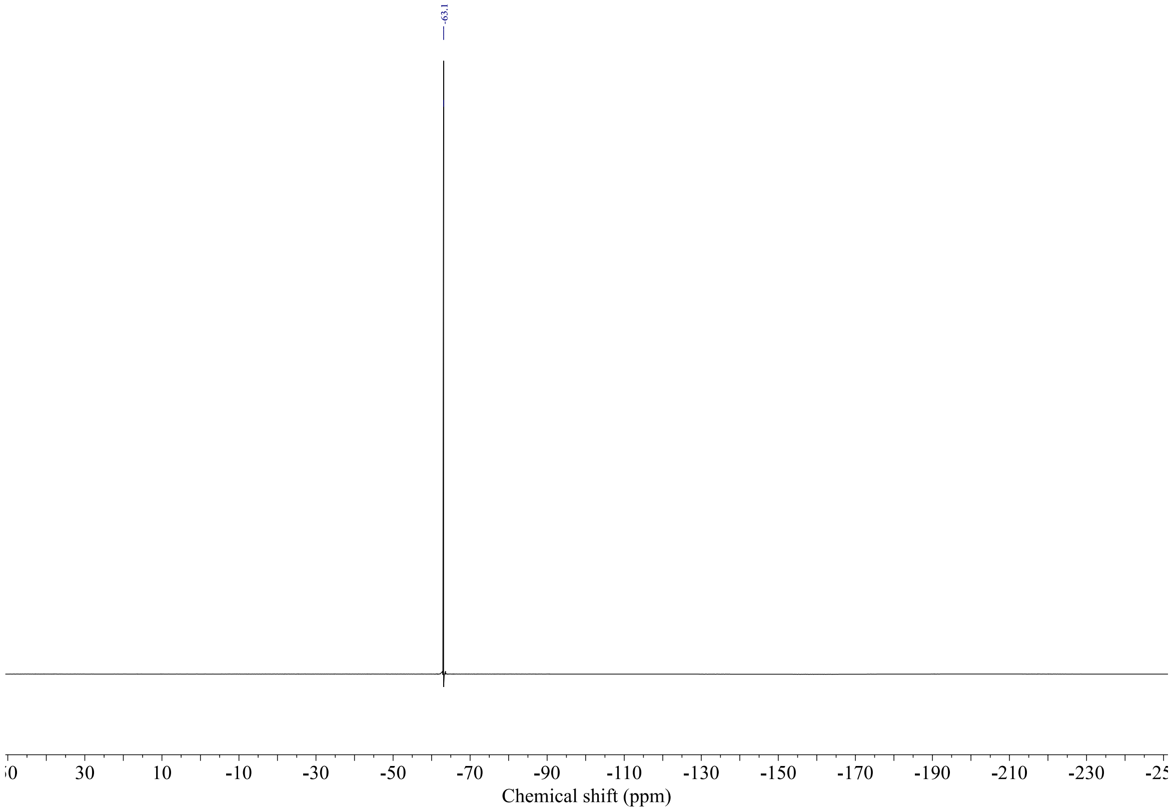

Figure S137. ^{19}F NMR of **S10** ($CDCl_3$, 376 MHz, 298 K)

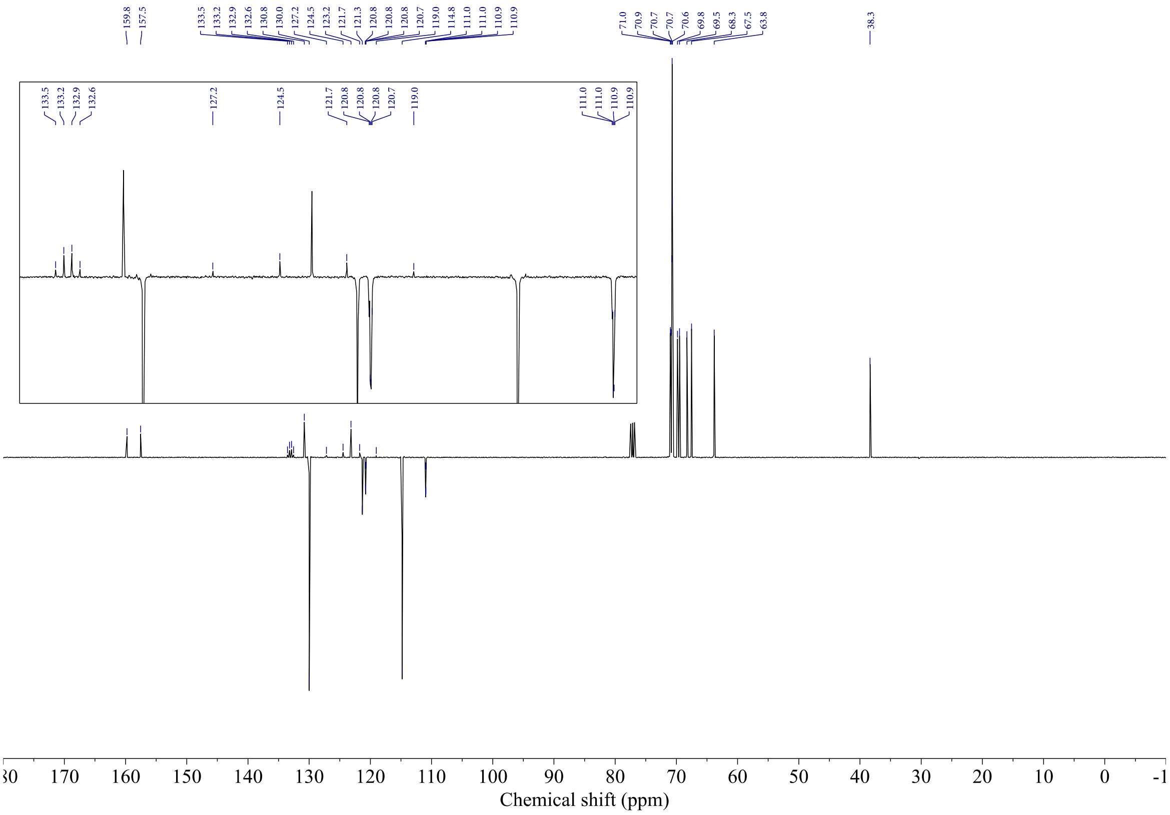

Figure S138. JMOD NMR of **S10** ($CDCl_3$, 101 MHz, 298 K)

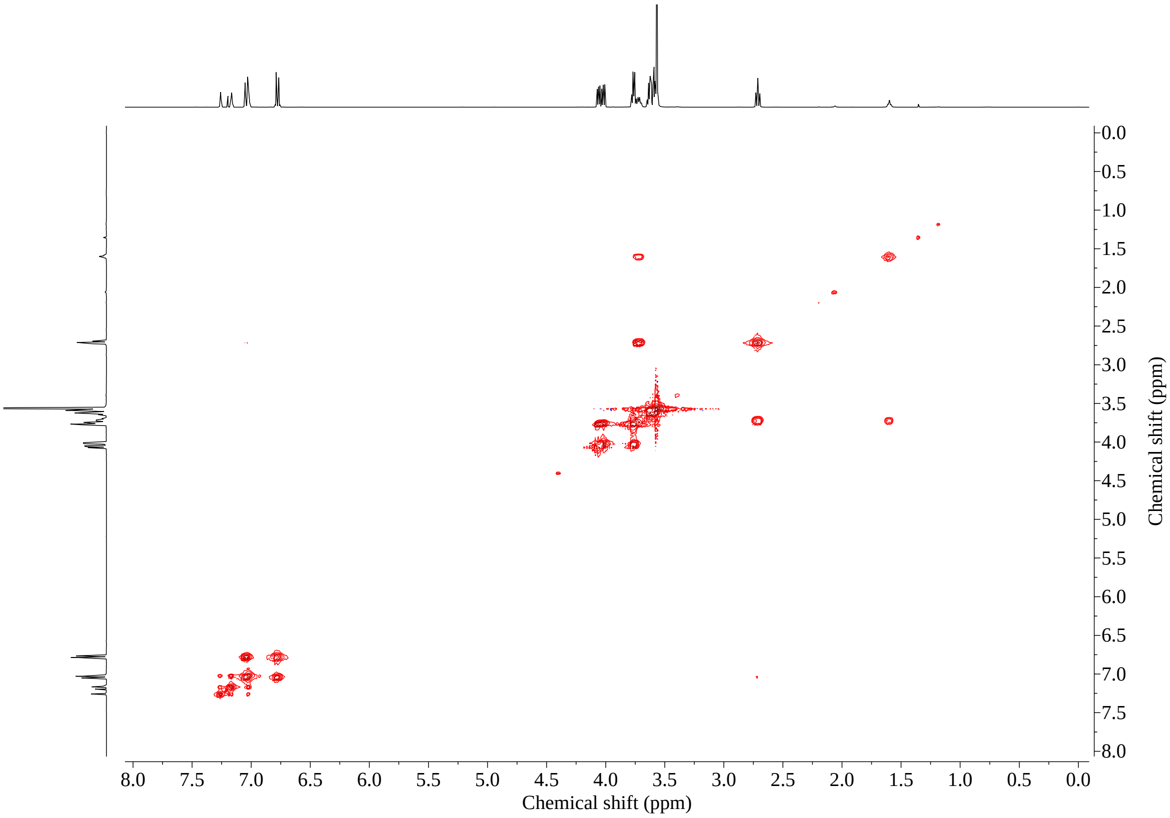

Figure S139. COSY NMR of **S10** ($CDCl_3$, 298 K)

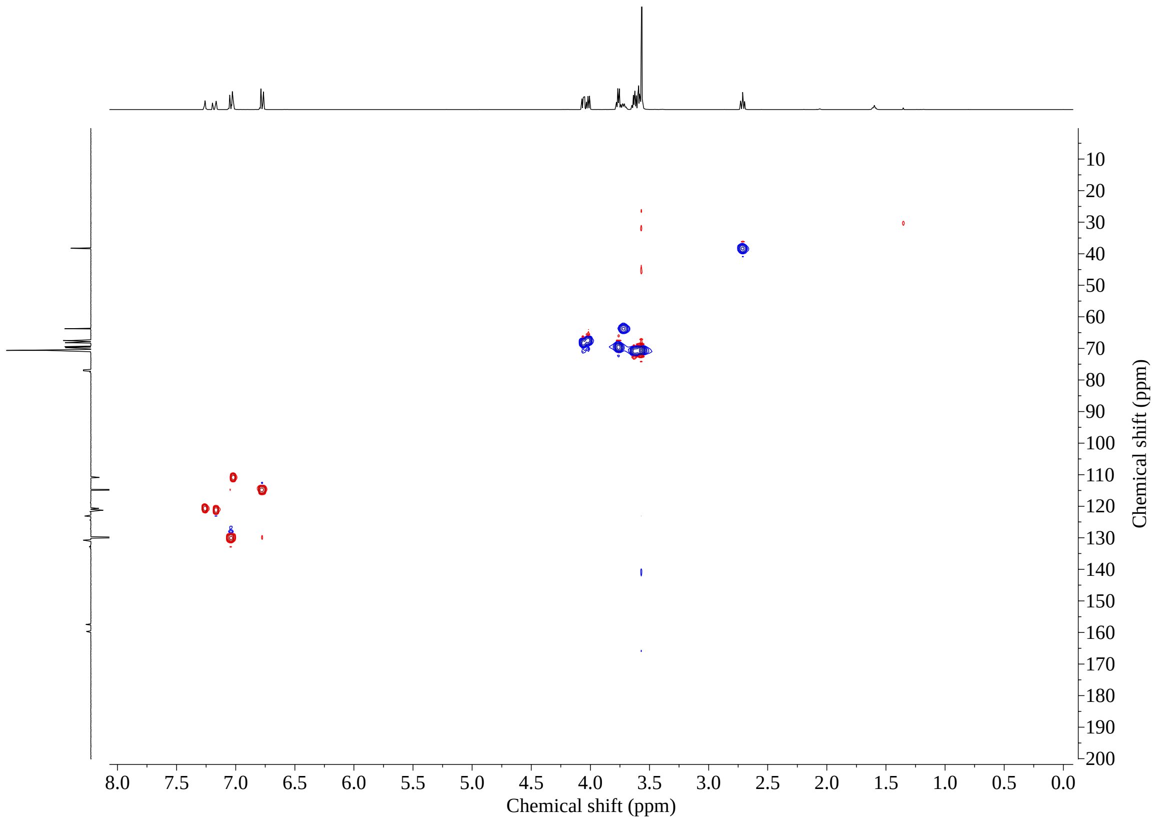

Figure S140. HSQC NMR of **S10** ($CDCl_3$, 298 K)

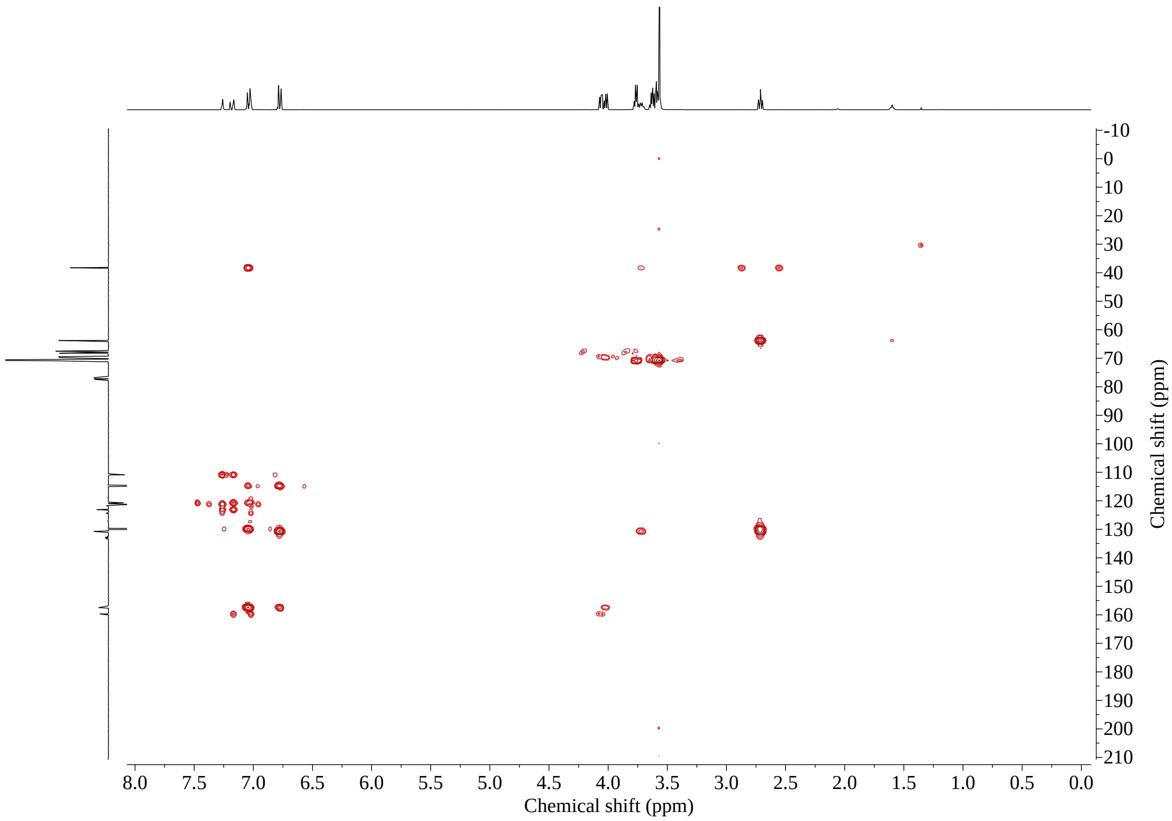

Figure S141. HMBC NMR of **S10** ($CDCl_3$, 298 K)

TMS acetylene S11

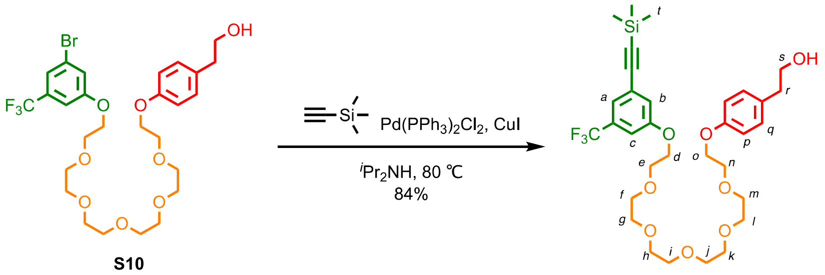

S10 (210 mg, 0.33 mmol, 1.0 equiv.), $Pd(PPh_3)_2Cl_2$ (5 mg, 0.007 mmol, 0.02 equiv.) and CuI (3 mg, 0.01 mmol, 0.04 equiv.) were suspended in $^iPr_2NH$ (3 mL). The resulting suspension was degassed by bubbling N_2 through the solution over 5 min. Ethynyltrimethylsilane (82 µL, 0.50 mmol, 1.5 equiv.) was added and the mixture was stirred at 80 °C for 16 h. The solvent was removed *in vacuo* and the crude was purified by column chromatography (petrol-Et_2O 80 : 20 → 50 : 50 → 30 : 70 → 0 : 100) to give **S11** as a yellow oil (179 mg, 84%).

^{1}H NMR (400 MHz, $CDCl_3$, 298 K) δ 7.32-7.29 (m, 1H, H_a), 7.15-7.08 (m, 4H, H_b, H_c, H_q), 6.88-6.83 (m, 2H, H_p), 4.18-4.06 (m, 4H, H_d, H_o), 3.88-3-77 (m, 6H, H_n, H_e, H_s), 3.75-3.58 (m, 16H, H_f, H_g, H_h, H_i, H_j, H_k, H_l, H_m) 2.79 (t, J = 6.6, 2H, H_r), 1.50 (t, J = 5.8, 1H, OH), 0.25 (s, 9H, H_t).

^{19}F NMR (376 MHz, $CDCl_3$, 298 K) δ: -63.26 (s, 3F, CF_3).

^{13}C NMR (101 MHz, $CDCl_3$, 298 K) δ 158.8, 157.6, 132.0 (q, $J_{C\text{-}F}$ = 32.8), 130.8, 130.0, 125.2, 123.6 (q, $J_{C\text{-}F}$ = 272.7), 121.4 (q, $J_{C\text{-}F}$ = 3.8), 120.6, 114.9, 112.6 (q, $J_{C\text{-}F}$ = 3.8), 103.4, 96.0, 71.0, 70.9, 70.8, 70.7 (×3), 70.7 (×2), 69.9, 69.6, 68.1, 67.6, 63.9, 38.4, -0.1.

HR-ESI-MS *m/z* = 660.3185 $[M+NH_4]^+$ calc. 660.3174 for $C_{32}H_{49}F_3NO_8Si$.

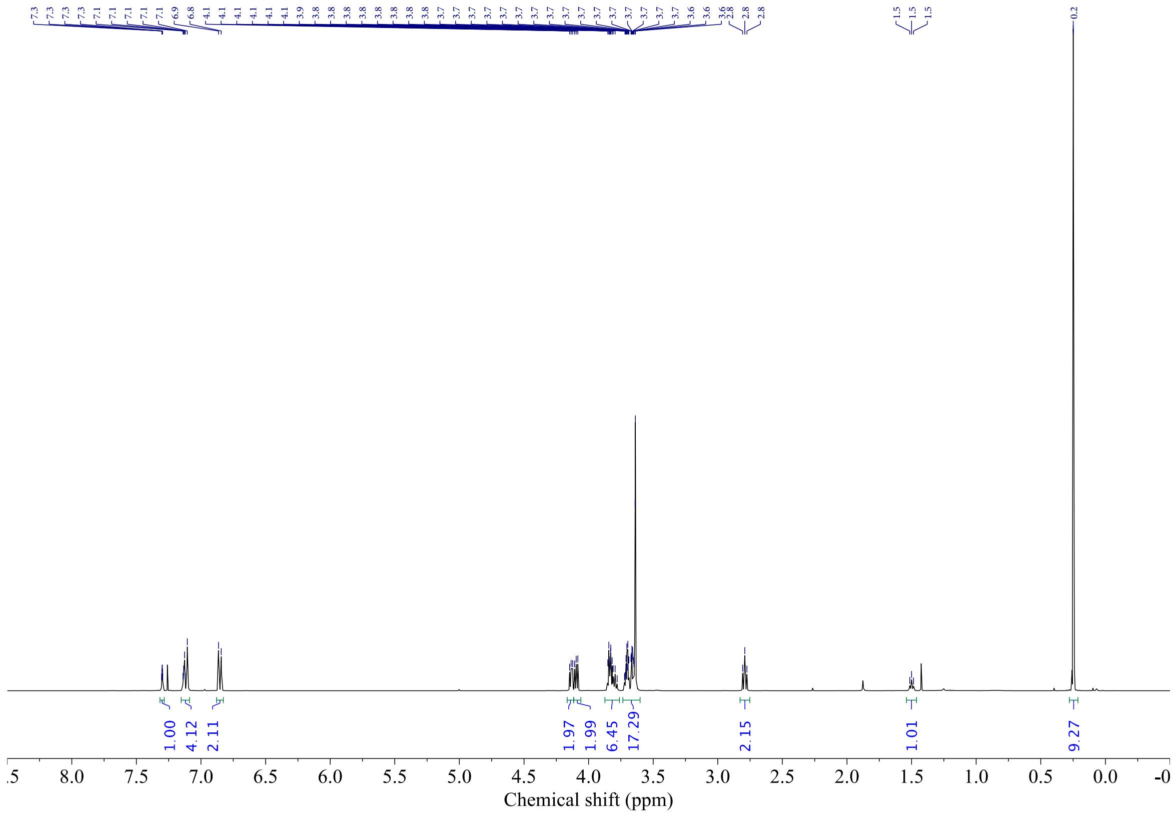

Figure S142. ^{1}H NMR of **S11** ($CDCl_3$, 400 MHz, 298 K)

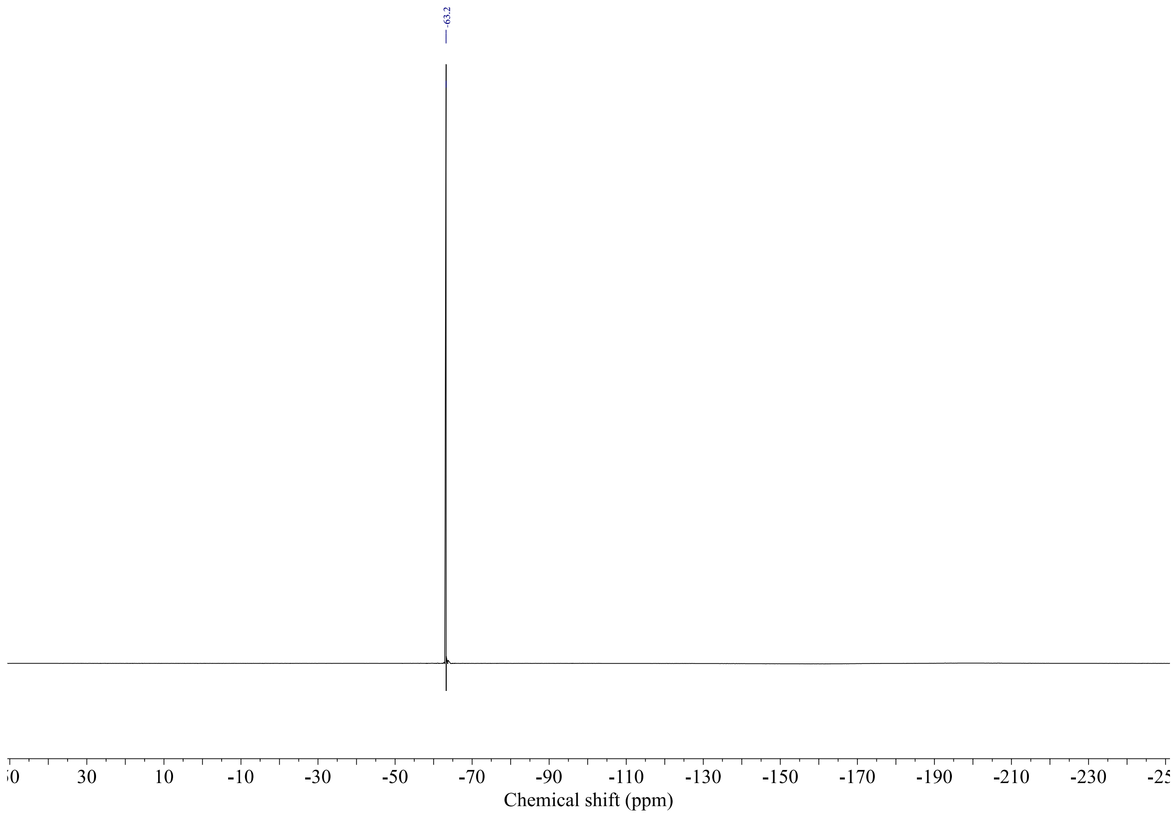

Figure S143. ^{19}F NMR of **S11** ($CDCl_3$, 376 MHz, 298 K)

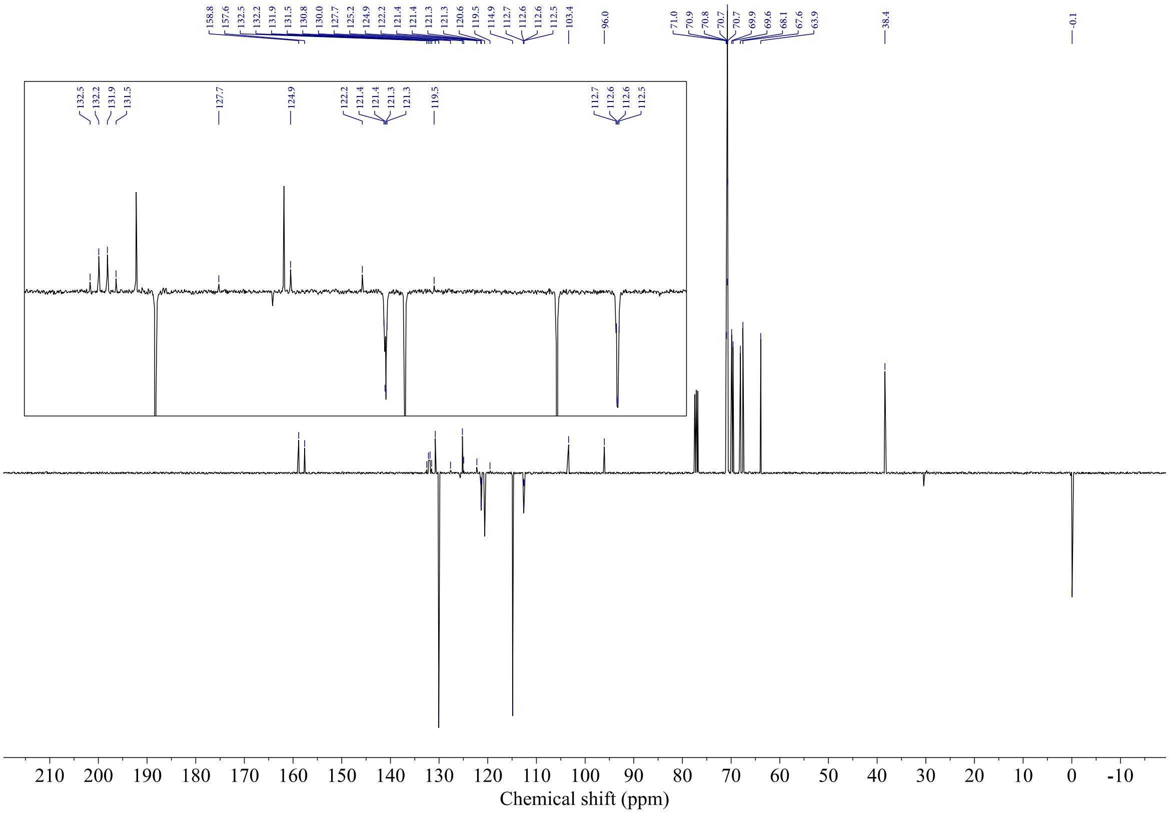

Figure S144. JMOD NMR of **S11** ($CDCl_3$, 101 MHz, 298 K)

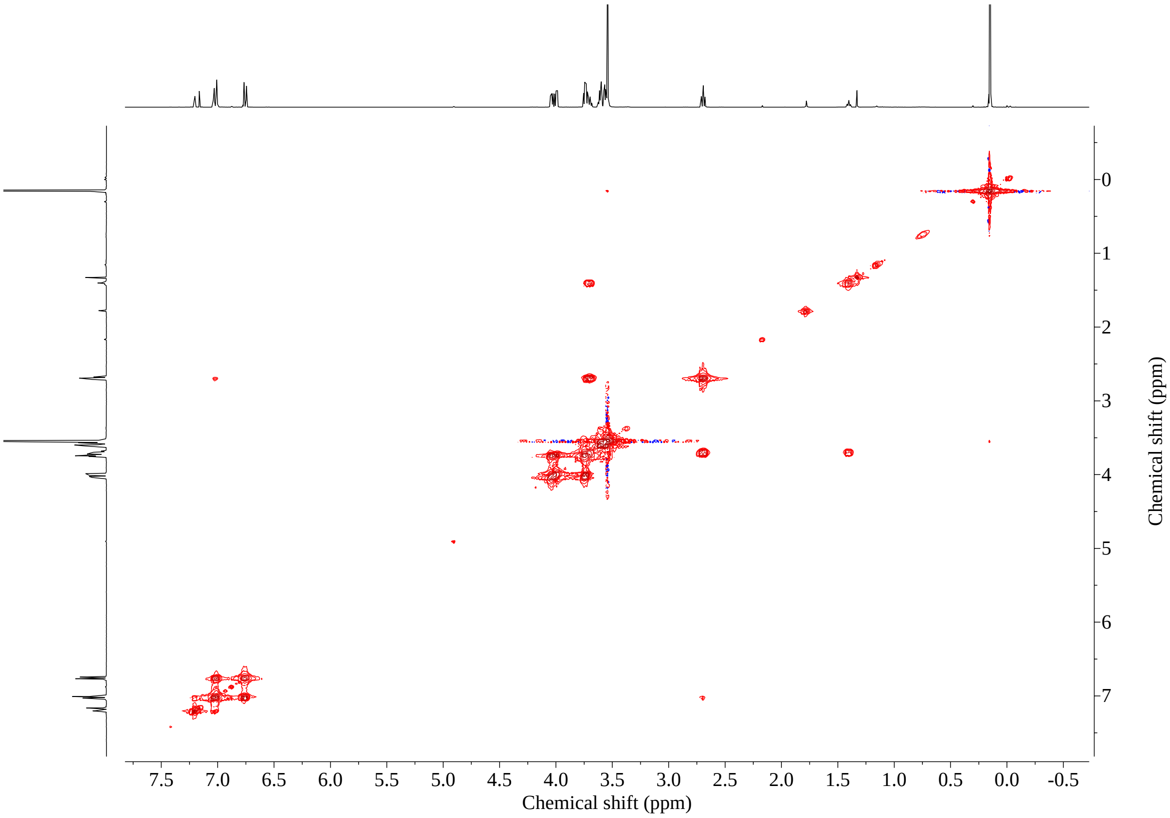

Figure S145. COSY NMR of **S11** ($CDCl_3$, 298 K)

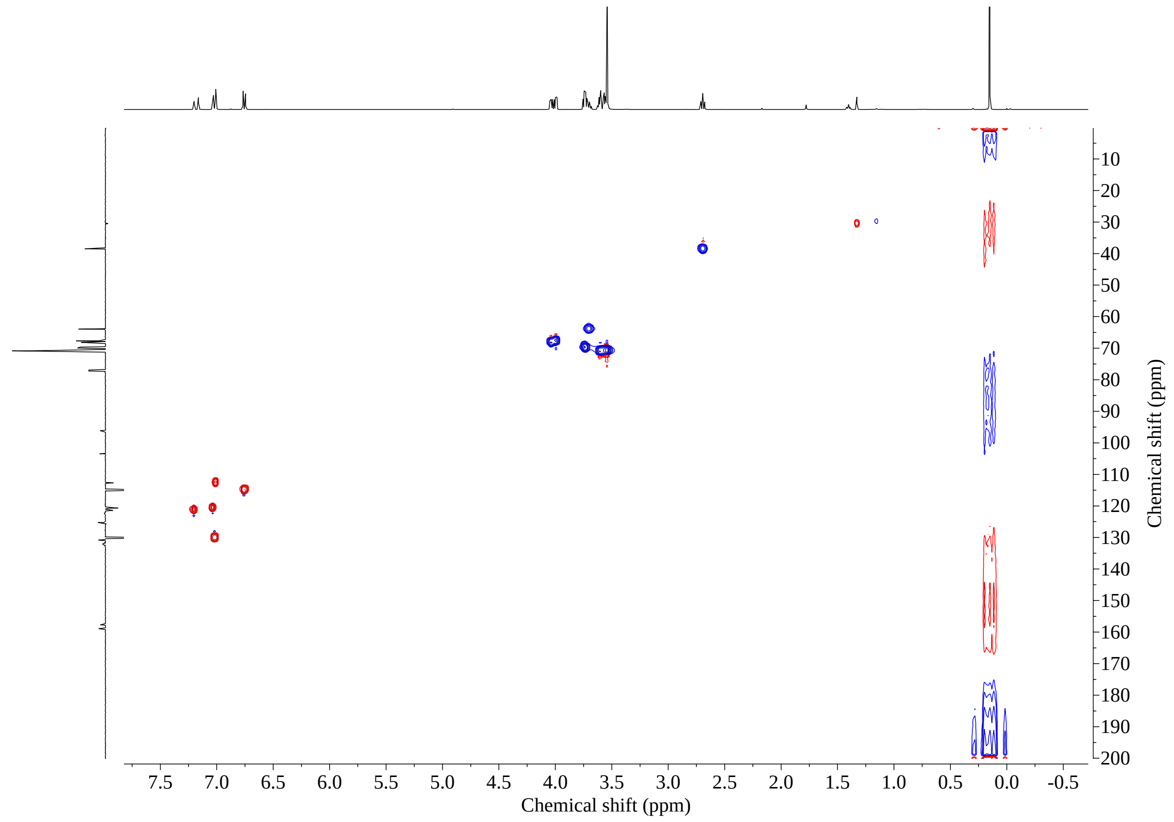

Figure S146. HSQC NMR of **S11** ($CDCl_3$, 298 K)

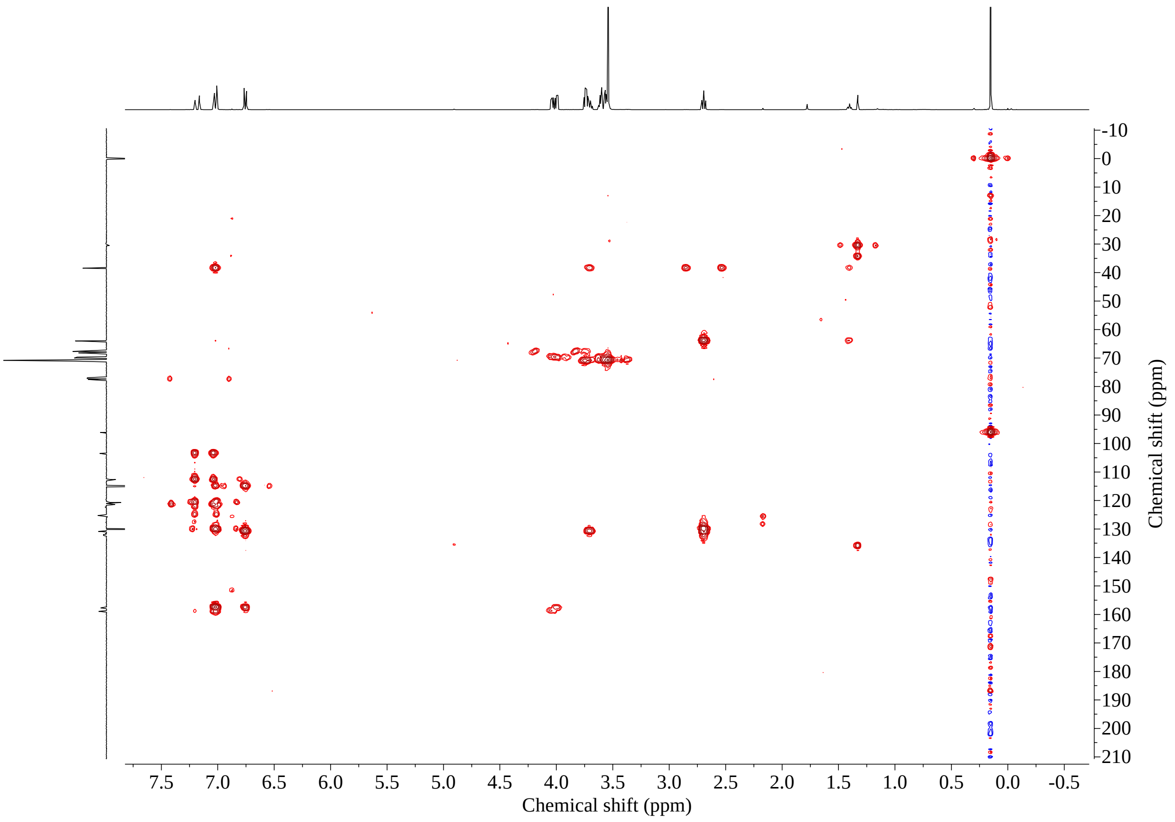

Figure S147. HMBC NMR of **S11** ($CDCl_3$, 298 K)

Terminal acetylene S12

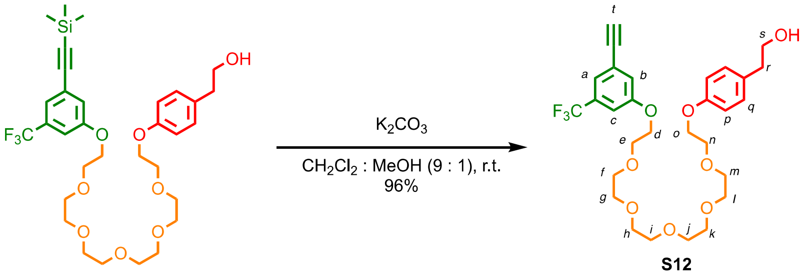

S11 (157 mg, 0.24 mmol, 1.0 equiv.) and K_2CO_3 (70 mg, 0.48 mmol, 2.0 equiv.) were suspended in CH_2Cl_2-MeOH (9 : 1, 2.5 mL) and stirred at rt for 48 h. The reaction mixture was diluted with brine (20 mL), extracted with CH_2Cl_2 (30 mL), the combined organic extracts dried ($MgSO_4$) and the solvent removed *in vacuo* to obtain **S12** as an orange oil (131 mg, 96%), which was used without further purification.

^{1}H NMR (400 MHz, $CDCl_3$, 298 K) δ 7.34-7.31 (m, 1H, H_a), 7.19-7.16 (m, 1H, H_b), 7.15-7.09 (m, 3H, H_c, H_q), 6.86 (d, J = 8.7, 2H, H_p), 4.17-4.07 (m, 4H, H_d, H_o), 3.87-3.78 (m, 6H, H_n, H_e, H_s), 3.73-3.62 (m, 16H, H_f, H_g, H_h, H_i, H_j, H_k, H_l, H_m), 3.06 (s, 1H, H_t), 2.79 (t, J = 6.8, 2H, H_r).

^{19}F NMR (376 MHz, $CDCl_3$, 298 K) δ: -63.13 (s, 3F, CF_3).

^{13}C NMR (101 MHz, $CDCl_3$, 298 K) δ 158.9, 157.6, 132.2 (q, J_{C-F} = 32.8), 130.8, 130.1, 124.2, 123.5 (app. d [outlying signals too weak to observe], J_{C-F} = 272.7), 121.5 (q, J_{C-F} = 3.9), 121.1, 114.9, 112.8 (q, J_{C-F} = 3.8), 82.2, 78.7, 71.0, 70.9, 70.8, 70.7 (×3), 70.7 (×2), 69.9, 69.6, 68.1, 67.6, 63.9, 38.4.

HR-ESI-MS *m/z* = 588.2787 $[M+NH_4]^+$ calc. 588.2779 for $C_{29}H_{41}F_3NO_8$.

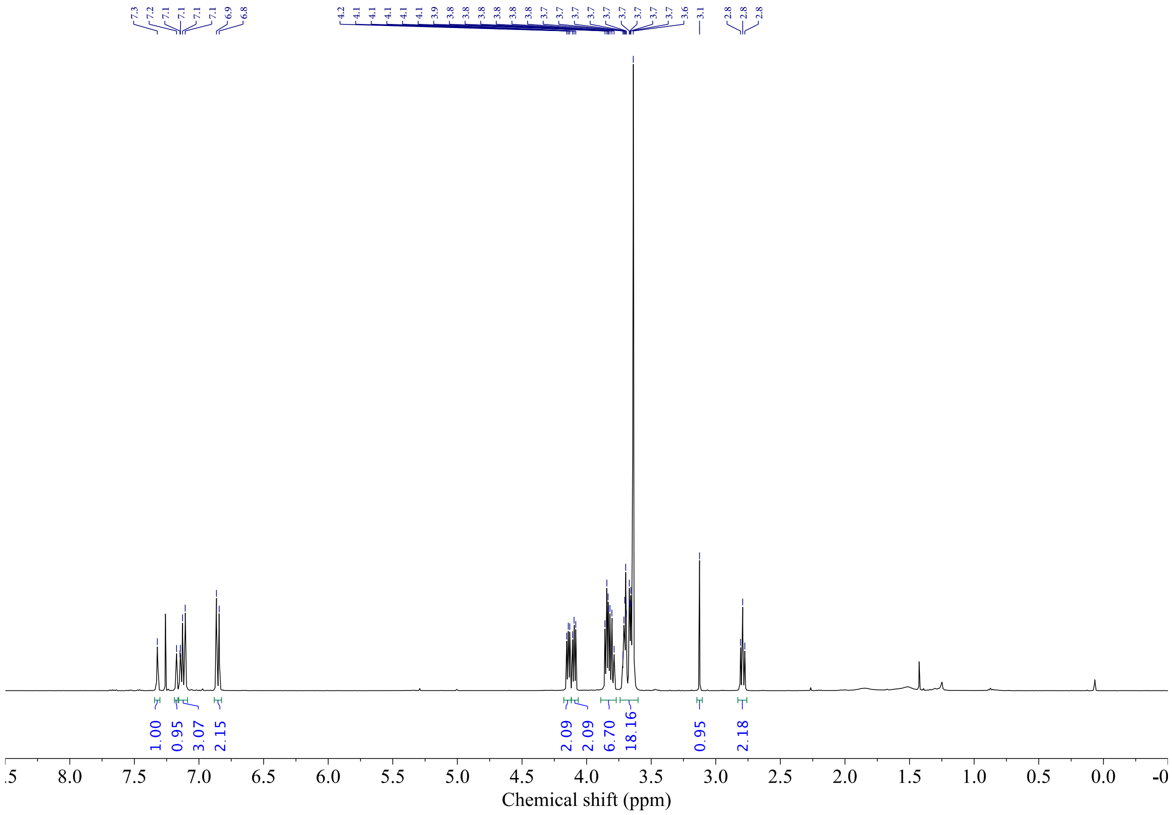

Figure S148. ^{1}H NMR of **S12** ($CDCl_3$, 400 MHz, 298 K)

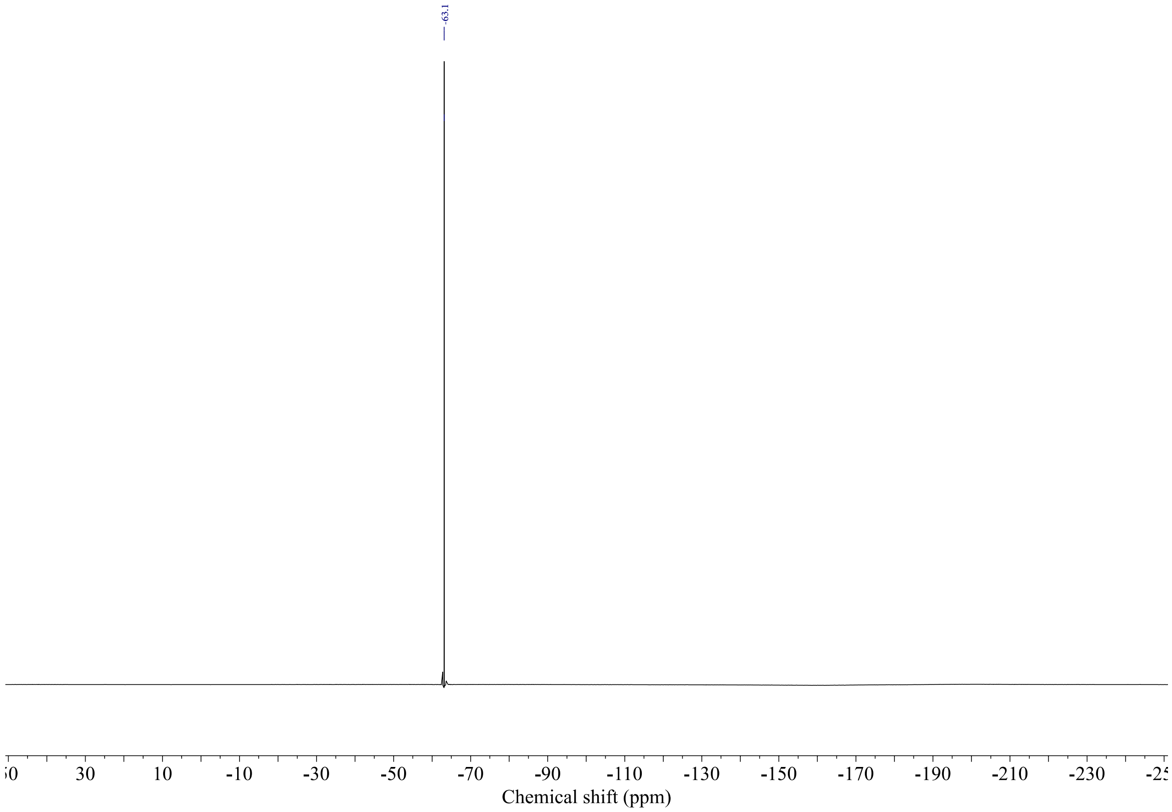

Figure S149. ^{19}F NMR of **S12** ($CDCl_3$, 376 MHz, 298 K)

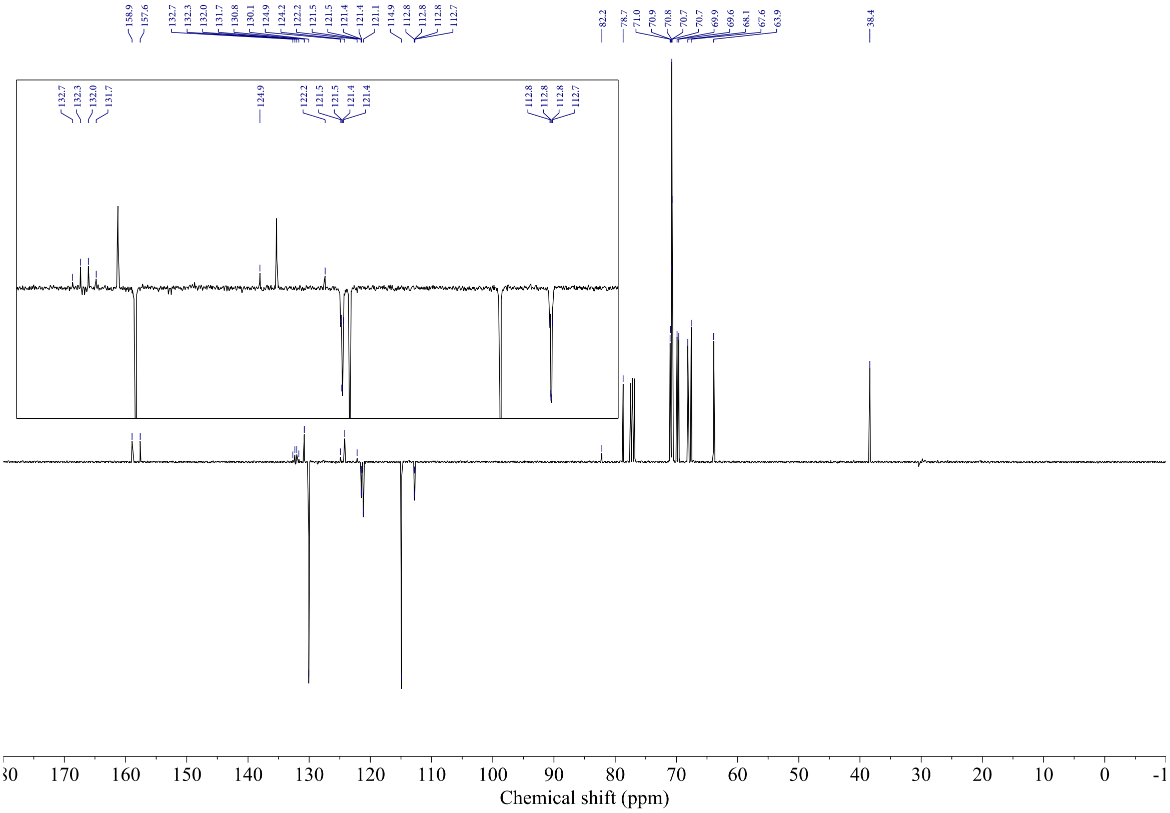

Figure S150. JMOD NMR of **S12** ($CDCl_3$, 101 MHz, 298 K)

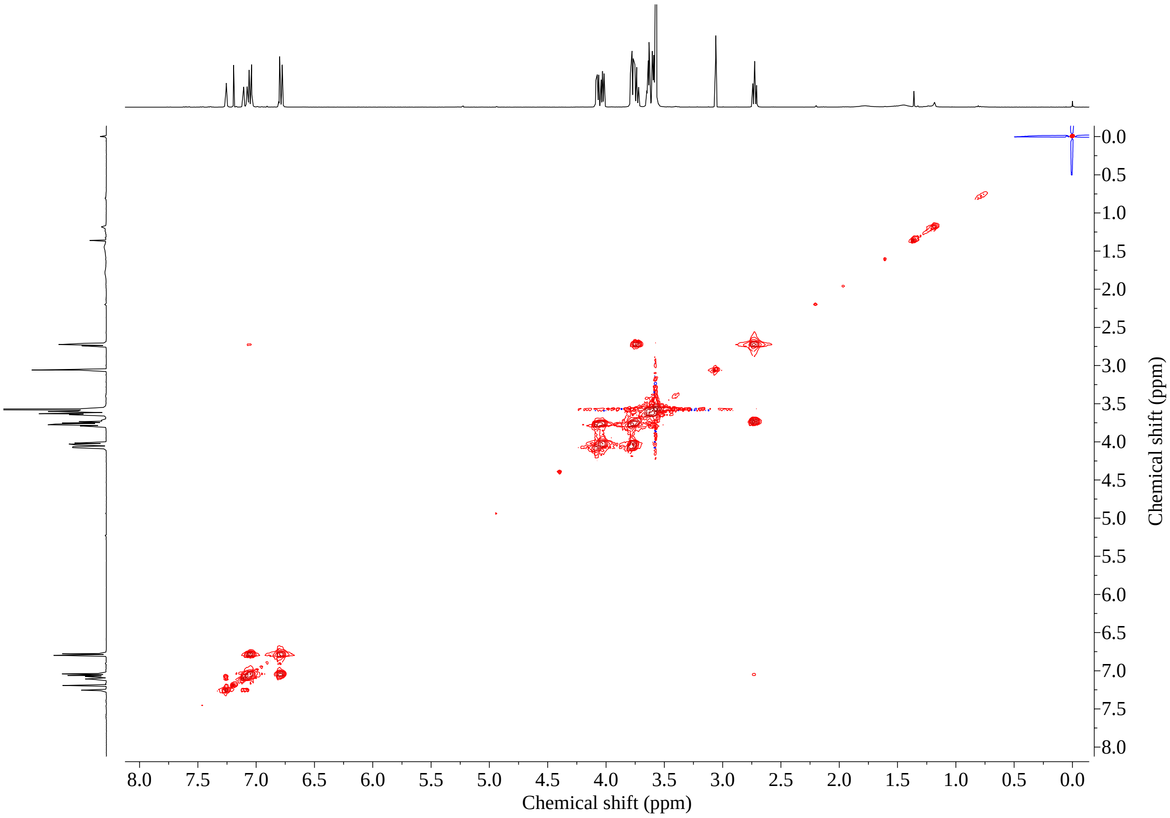

Figure S151. COSY NMR of **S12** ($CDCl_3$, 298 K)

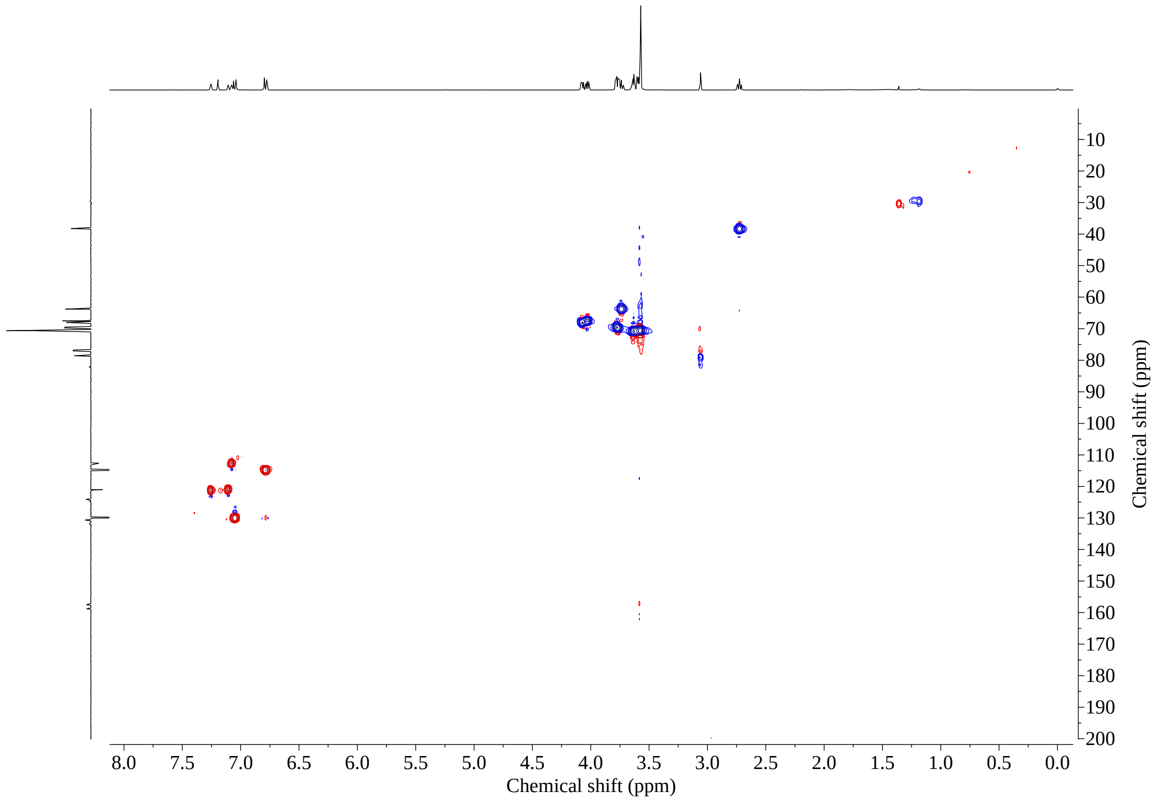

Figure S152. HSQC NMR of **S12** ($CDCl_3$, 298 K)

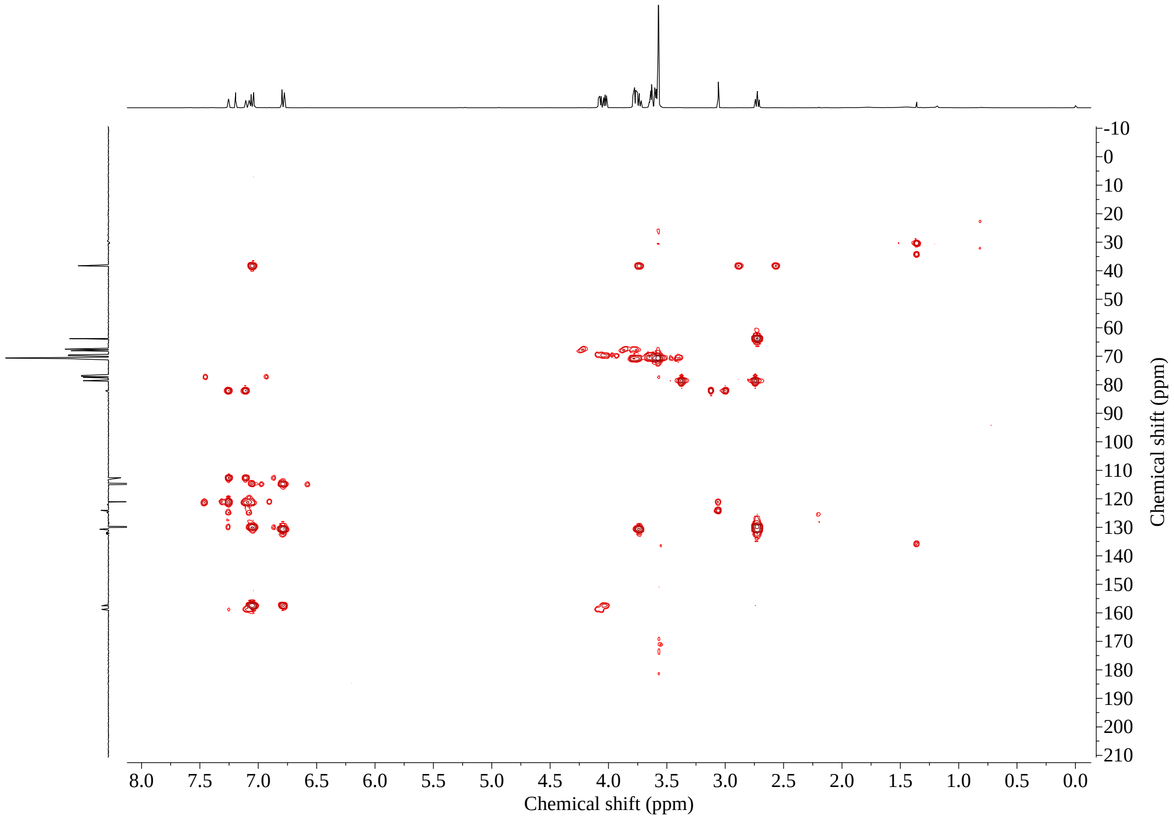

Figure S153. HMBC NMR of **S12** ($CDCl_3$, 298 K)

Macrocycle precursor S13

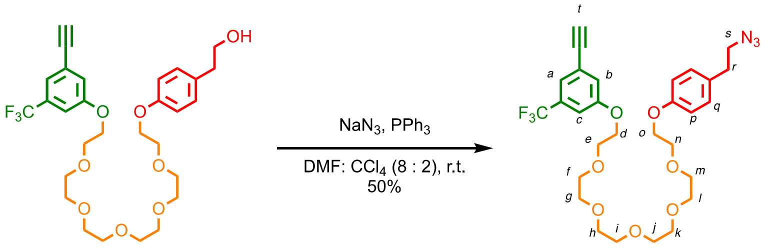

S12 (131 mg, 0.23 mmol, 1.0 equiv.), NaN_3 (18 mg, 0.28 mmol, 1.2 equiv.) and PPh_3 (60 mg, 0.23 mmol, 1.0 equiv.) were dissolved in DMF-CCl_4 (8 : 2, 2.5 mL) and stirred at rt for 16 h. The reaction mixture was quenched with LiCl solution (5% w/w, 10 mL), extracted with Et_2O (30 mL), the combined organic extracts dried ($MgSO_4$) and the solvent removed *in vacuo*. Column chromatography (petrol-Et_2O 50 : 50 → 0 : 100) gave **S13** as a colourless oil (69 mg, 50%).

^{1}H NMR (400 MHz, $CDCl_3$, 298 K) δ 7.34-7.32 (m, 1H, H_a), 7.19-7.16 (m, 1H, H_b), 7.16-7.13 (m, 1H, H_c), 7.11 (d, *J* = 8.5, 2H, H_q) 6.86 (d, *J* = 8.7, 2H, H_p), 4.17-13 (m, 2H, H_d), 4.13-4.08 (m, 2H, H_o), 3.87-3-81 (m, 4H, H_n, H_e), 3.74-3.61 (m, 16H, H_f, H_g, H_h, H_i, H_j, H_k, H_l, H_m), 3.45 (t, *J* = 7.2, 2H, H_s) 3.13 (s, 1H, H_t) 2.82 (t, *J* = 6.8, 2H, H_r).

^{19}F NMR (376 MHz, $CDCl_3$, 298 K) δ: -63.28 (s, 3F, CF_3).

^{13}C NMR (101 MHz, $CDCl_3$, 298 K) δ 158.9, 157.8, 132.2 (q, J_{C-F} = 32.8), 130.3, 129.8, 124.2, 123.5 (q, J_{C-F} = 272.6), 121.4 (q, J_{C-F} = 3.9), 121.1, 114.9, 112.8 (q, J_{C-F} = 3.8), 82.2, 78.7, 71.0, 70.9, 70.7, 70.7 (×3), 70.7 (×2), 69.8, 69.6, 68.1, 67.5, 52.8, 34.6.

HR-ESI-MS *m/z* = 613.2852 $[M+NH_4]^+$ calc. 613.2844 for $C_{29}H_{40}F_3N_4O_7$.

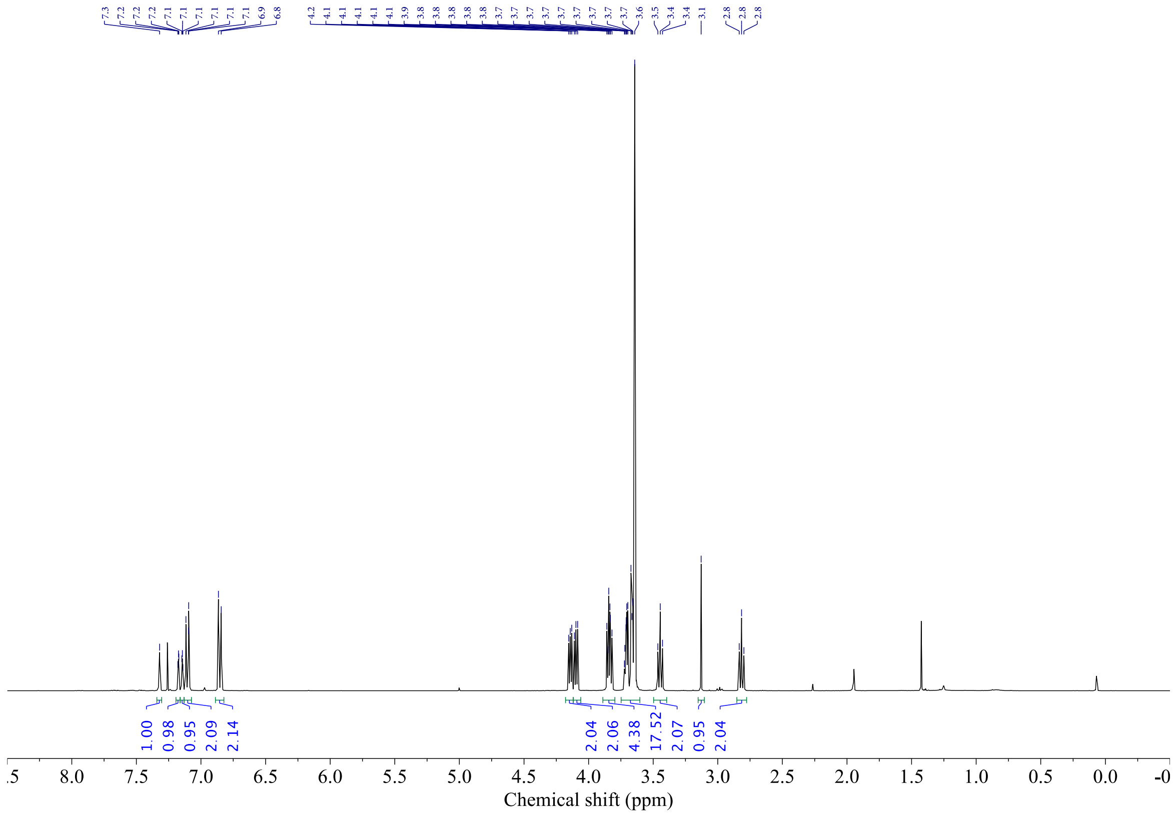

Figure S154. ^{1}H NMR of **S13** ($CDCl_3$, 400 MHz, 298 K)

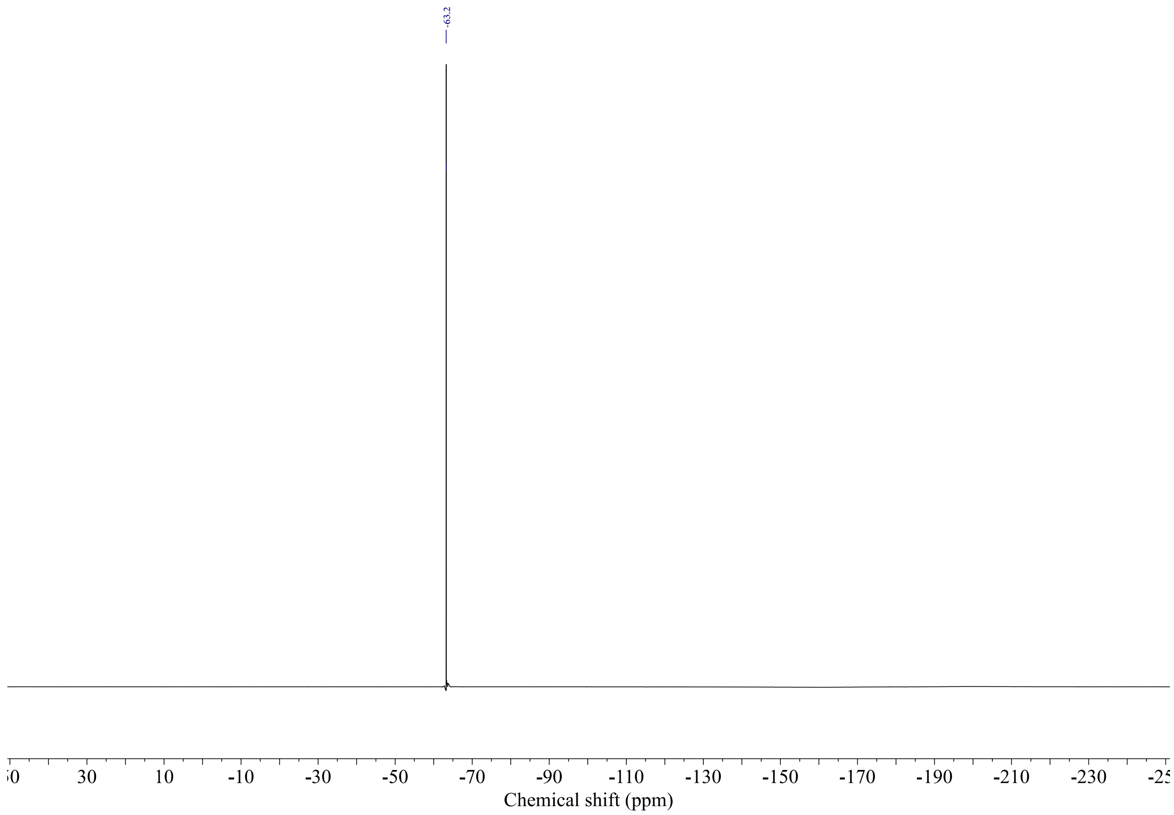

Figure S155. ^{19}F NMR of **S13** ($CDCl_3$, 376 MHz, 298 K)

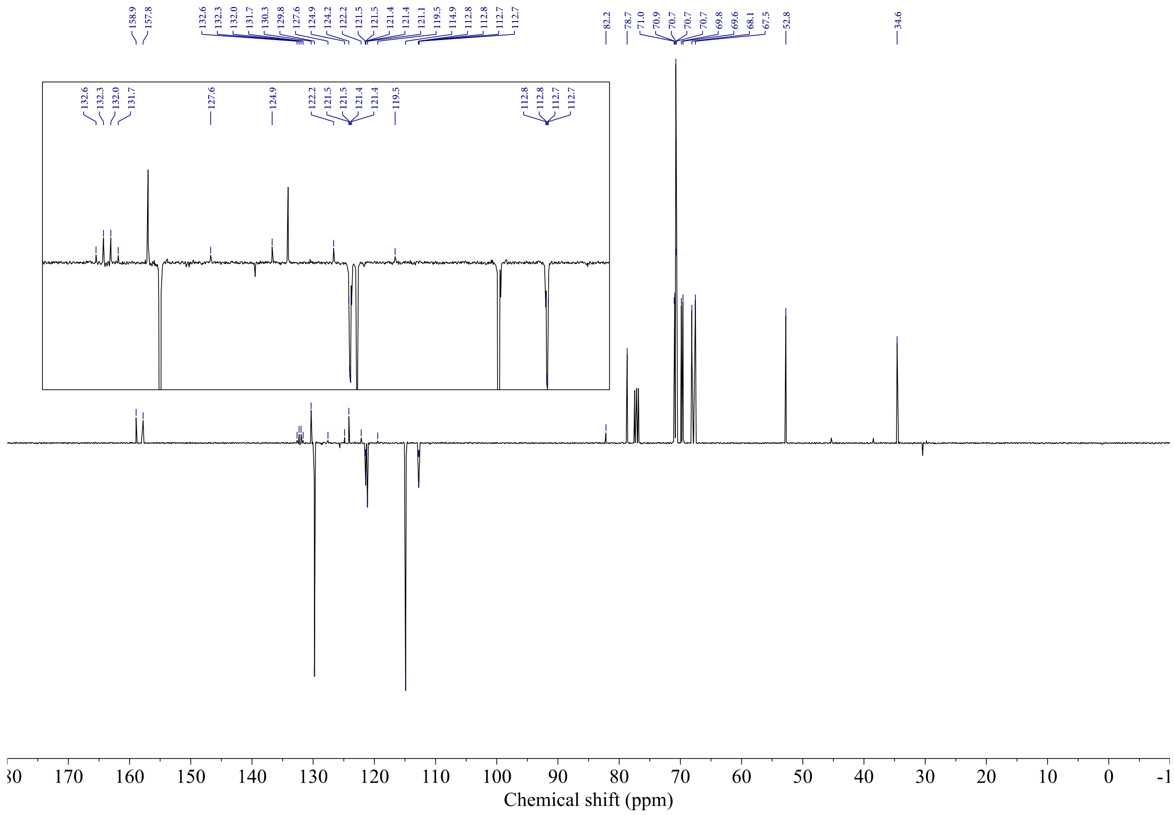

Figure S156. JMOD NMR of **S13** ($CDCl_3$, 101 MHz, 298 K)

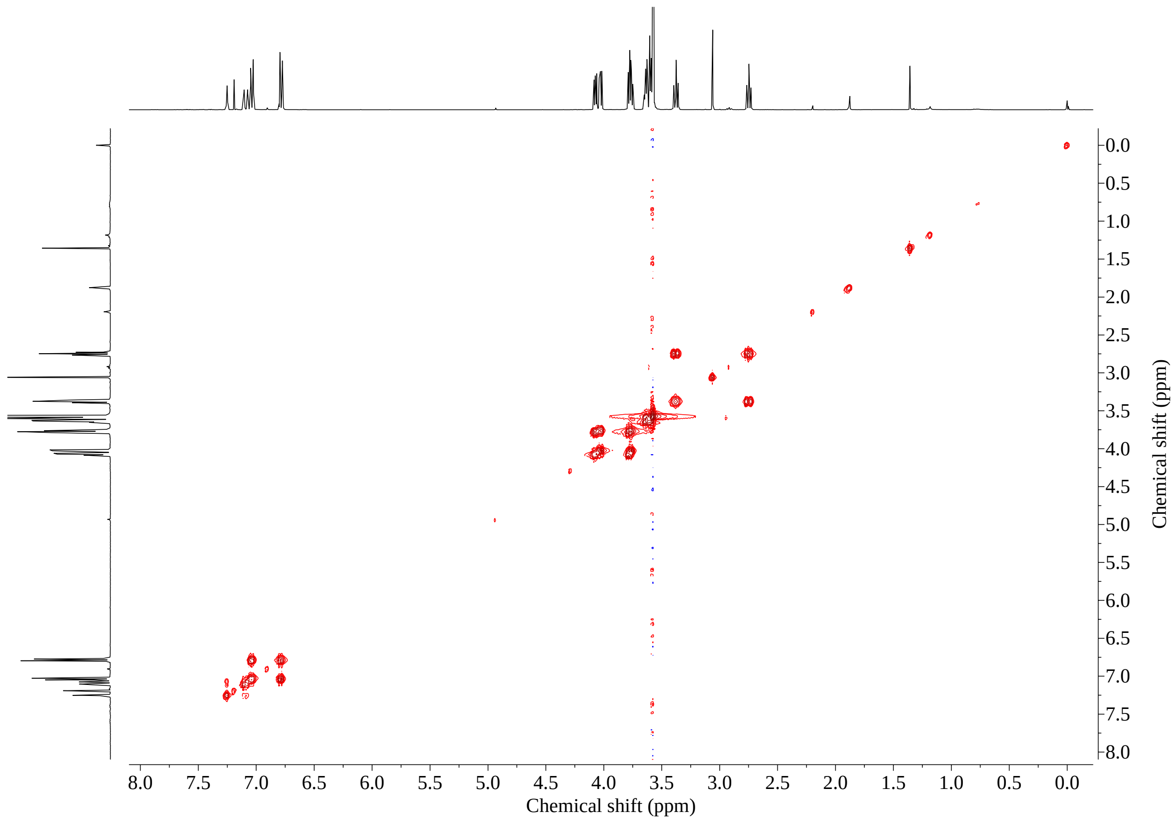

Figure S157. COSY NMR of **S13** ($CDCl_3$, 298 K)

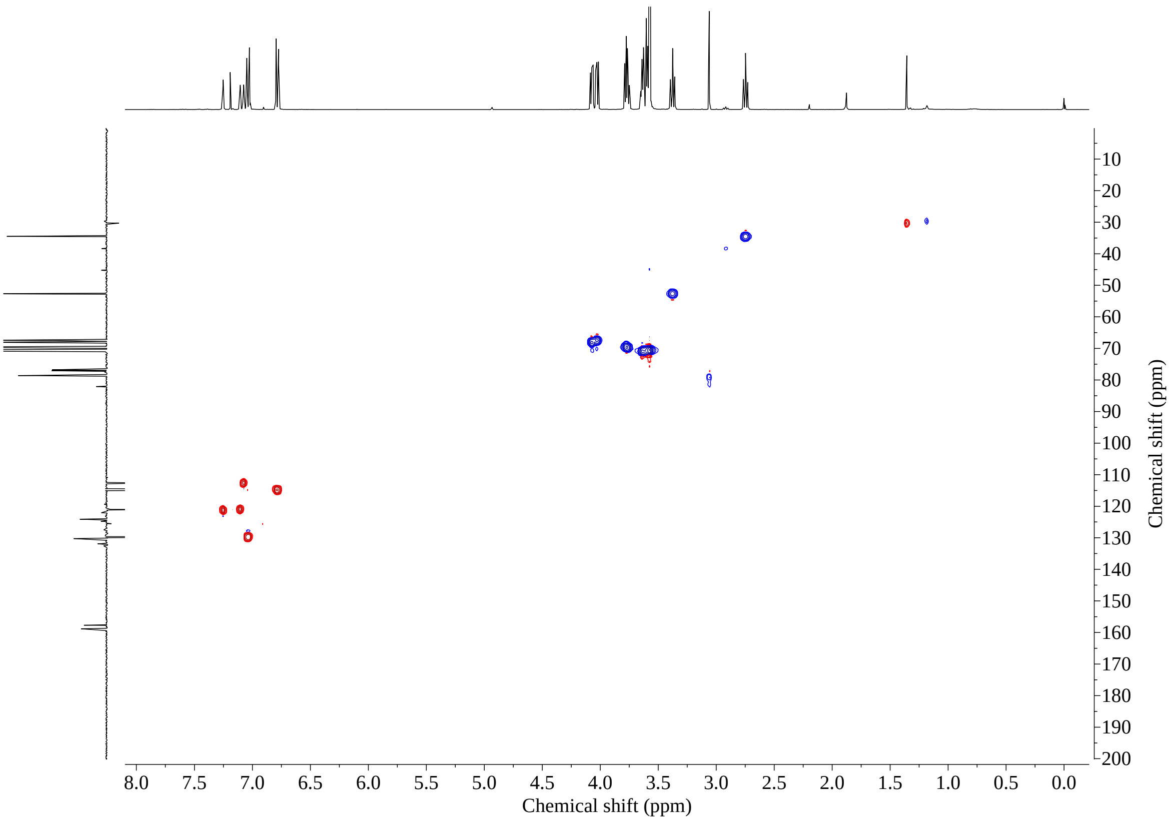

Figure S158. HSQC NMR of **S13** ($CDCl_3$, 298 K)

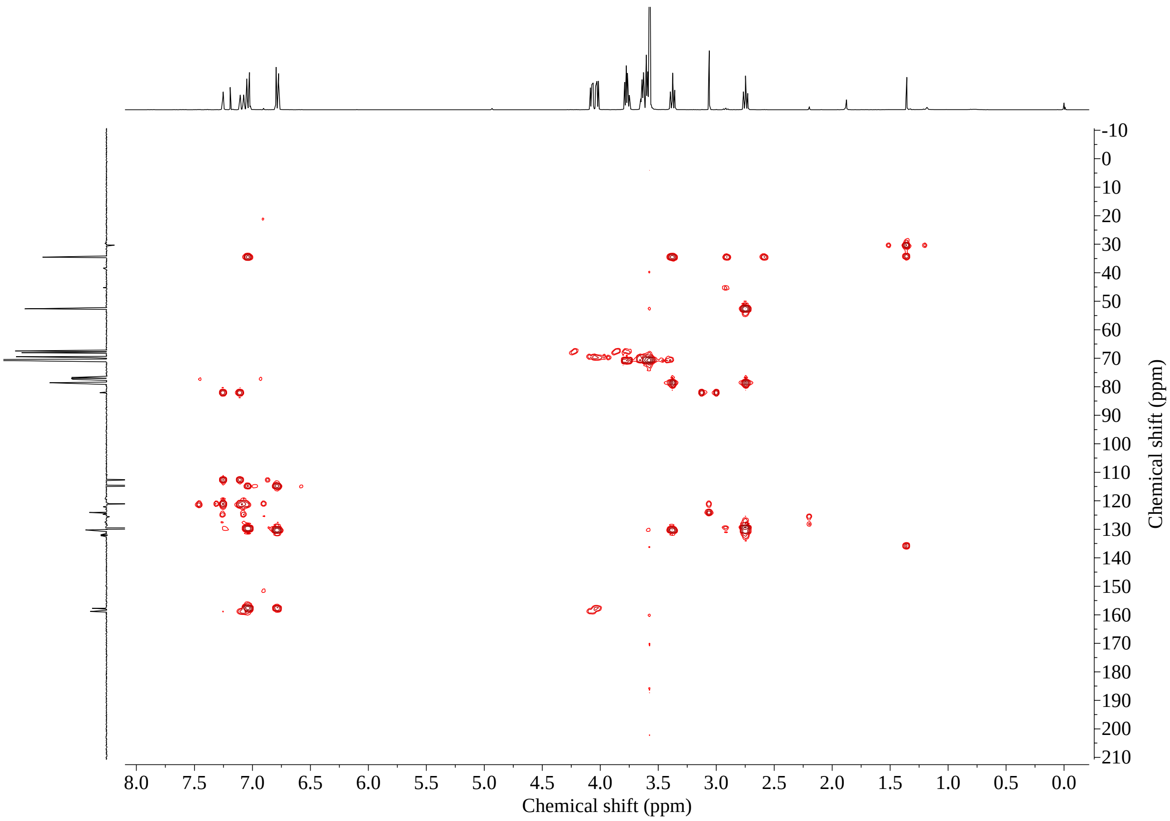

Figure S159. HMBC NMR of **S13** ($CDCl_3$, 298 K)

Synthesis of catenanes *rac*-6 and (S_{mt})-6

Catenane *rac*-6

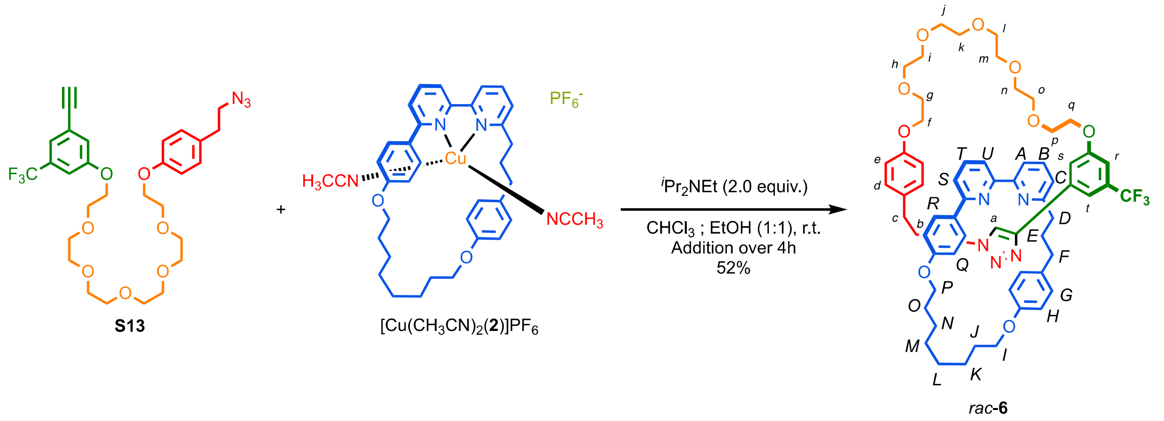

To a solution of [Cu(CH_3CN)$_2$(**2**)]PF_6 (63 mg, 0.085 mmol, 1.0 eq.), ^{i}Pr$_2$NEt (30 µL, 0.17 mmol, 2.0 eq.) in $CHCl_3$-EtOH (1 : 1, 8.5 mL) at rt was added a solution of **S13** (51 mg, 0.085 mmol, 1.0 eq.) in $CHCl_3$-EtOH (1 : 1, 3.4 mL) over 4 h. Once the addition had finished, H_2O (10 mL) was added followed by KCN (55 mg, 0.85 mmol, 10 equiv.) and the mixture stirred at rt for 16 h. The phases were separated, and the aqueous layer was extracted with $CHCl_3$ (10 mL). The combined organic extracts were dried ($MgSO_4$) and the solvent was removed *in vacuo*. Two rounds of column chromatography (petrol-CH_2Cl_2-CH_3CN 45 : 45 : 10 → 40 : 40 : 20 → 25 : 25 : 50; petrol-EtOAc 50 : 50 → 25 : 75) gave catenane *rac*-**6** as a colourless oil (48 mg, 52%).

^{1}H NMR (500 MHz, $CDCl_3$, 298 K) δ: 8.26 (s, 1H, H_a), 7.89-7.83 (m, 1H, H_t), 7.80 (t, J = 7.7, 1H, H_B), 7.79 (t, J = 7.8, 1H, H_T), 7.65 (d, J = 7.7, 1H, H_A), 7.58 (dd, J = 13.5, 0.9, 1H, H_U), 7.57 (dd, J = 13.5, 0.9, 1H, H_S), 7.31-7.27 (m, 1H, H_s), 7.22-7.04 (m, 3H, H_C, H_G), 6.87-6.79 (m, 1H, H_r), 6.57 (d, J = 8.6, 2H, H_d), 6.55 (d, J = 8.6, 2H, H_R), 6.41 (d, J = 8.6, 2H, H_e), 6.40 (d, J = 8.7, 2H, H_H), 6.23 (d, J = 8.6, 2H, H_Q), 4.05-3.90 (m, 5H, H_I, H_P, H_b), 3.86 (ddd, J = 14.0, 9.1, 2.9, 1H, $H_{b'}$), 3.75-3.18 (m, 23H, H_f, H_g, H_h, H_i, H_j, H_k, H_l, H_m, H_n, H_o, H_p, H_q), 3.05 (ddd, J = 9.5, 5.5, 4.2, 1H, $H_{q'}$), 2.88 (ddd, J = 15.2, 8.9, 2.9, 1H, H_c), 2.76 (ddd, J = 15.3, 7.5, 2.8, 1H, $H_{c'}$), 2.53-2.31 (m, 4H, H_D, H_F), 1.90-1.78 (m, 2H, H_O), 1.77-1.64 (m, 4H, H_E, H_J), 1.61-1.40 (m, 8H, H_K, H_L, H_M, H_N).

^{19}F NMR (470 MHz, $CDCl_3$, 298 K) δ: -62.50 (s, 3F, CF_3).

^{13}C NMR (126 MHz, $CDCl_3$, 298 K) δ 163.0, 159.1, 158.9, 158.6, 157.4, 157.3, 157.1, 157.1, 145.9, 137.2, 137.1, 133.2, 132.7, 131.9, 131.1 (q, J_{C-F} = 32.2), 129.3, 129.1 (×2), 129.0, 124.0, 124.3 (q, J_{C-F} = 272.5), 122.2, 120.4, 120.4, 120.1, 114.7, 114.4 (×2), 114.2 (q [right hand 2 signals of quartet obscured by neighbouring resonance; centre of resonance inferred based on coupling constant], J_{C-F} = 4.0), 114.2, 111.9 (q, J_{C-F} = 4.1), 71.1, 71.0, 71.0, 71.0, 70.9, 70.9, 70.7, 70.6, 69.6, 69.4, 67.7, 66.7, 66.6, 66.6, 50.6, 37.4, 35.2, 35.0, 31.8, 29.3, 28.7, 28.7, 28.6, 25.9, 25.7.

LR-ESI-MS *m/z* = 1088.5 $[M+H]^+$ for $C_{62}H_{72}F_3N_5O_9$ (see isotope pattern, Figure S167)

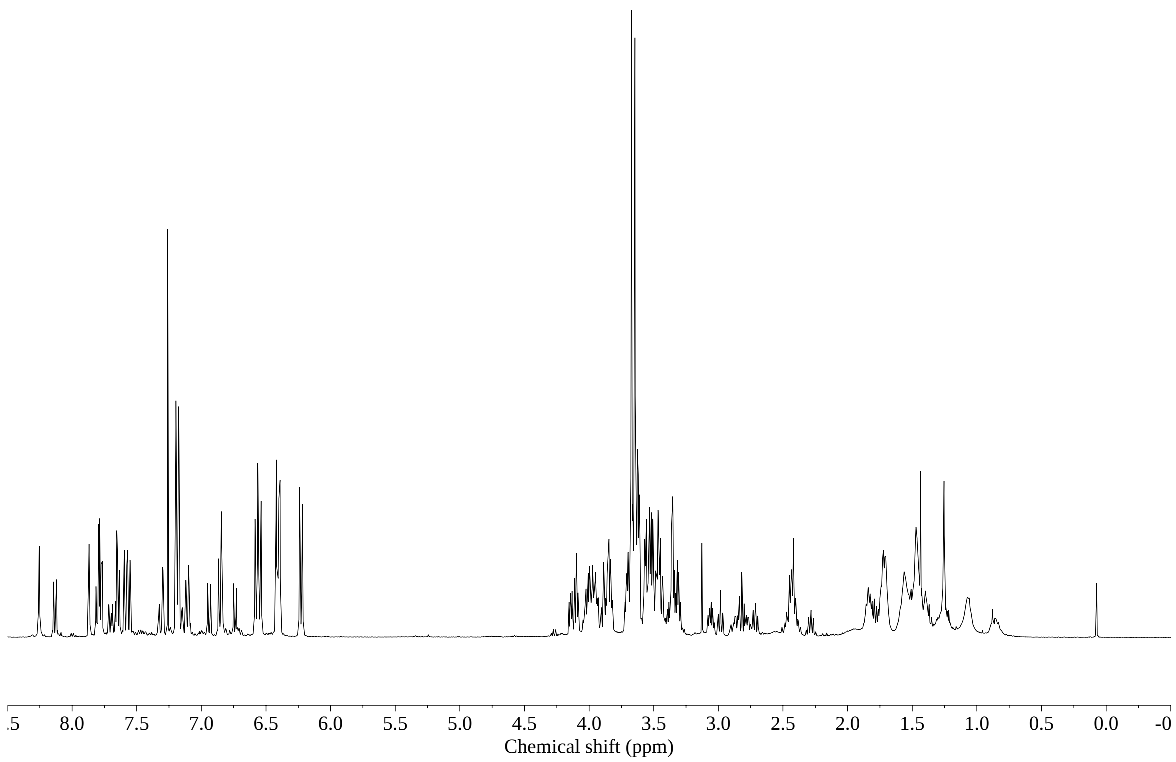

Figure S160. ^{1}H NMR of crude *rac*-**6** ($CDCl_3$, 400 MHz, 298 K)

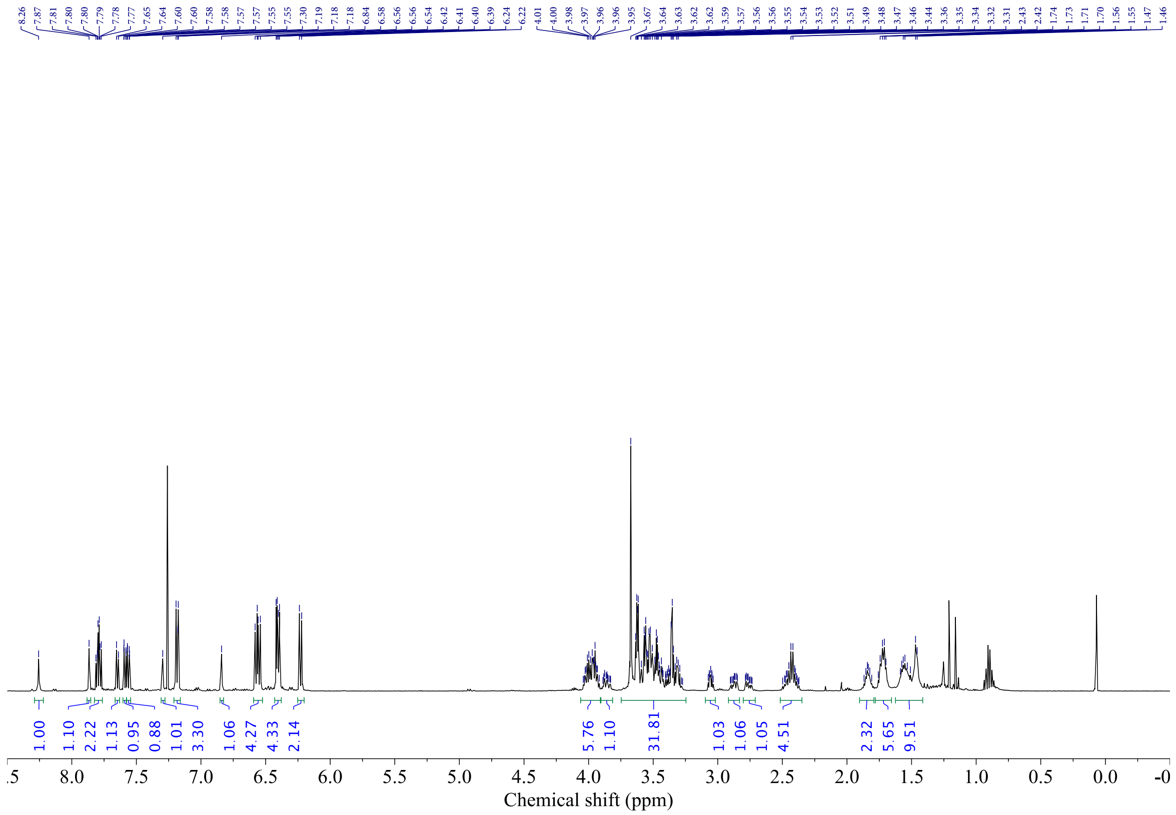

Figure S161. ^{1}H NMR of *rac*-**6** ($CDCl_3$, 500 MHz, 298 K)

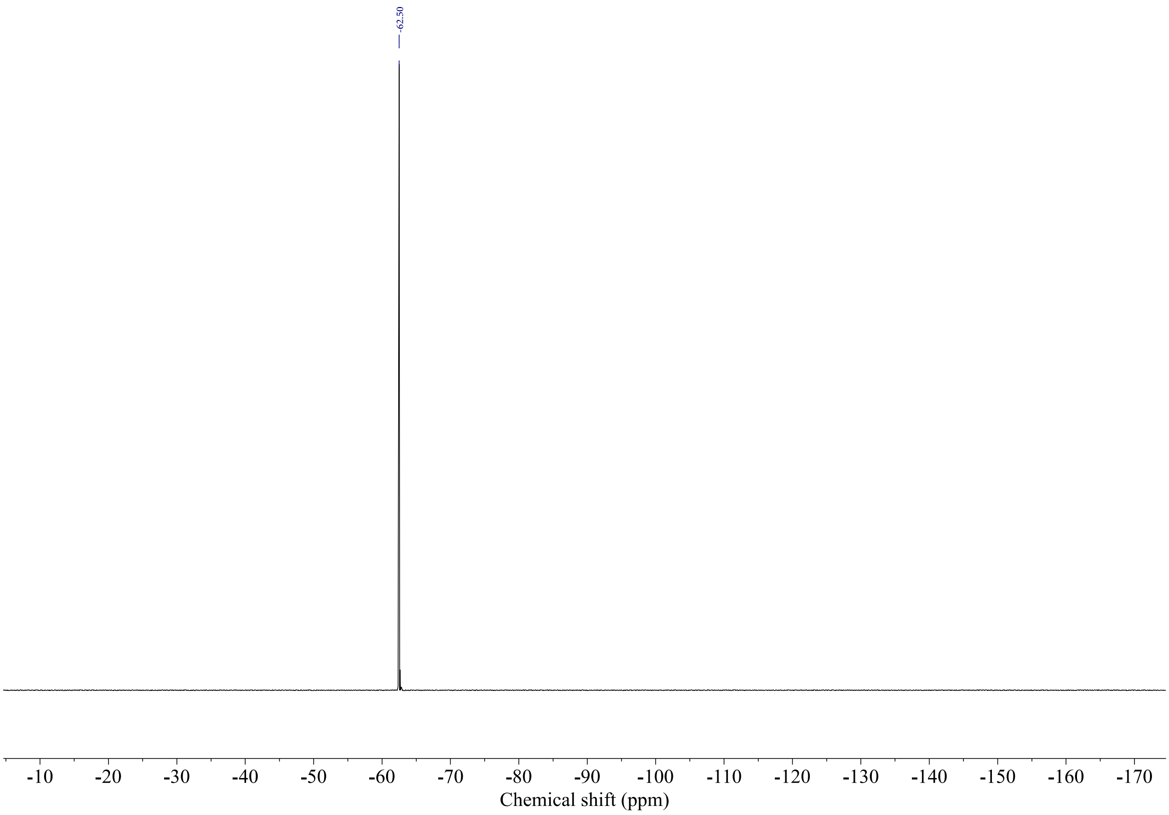

Figure S162. ^{19}F NMR of *rac*-**6** ($CDCl_3$, 470 MHz, 298 K)

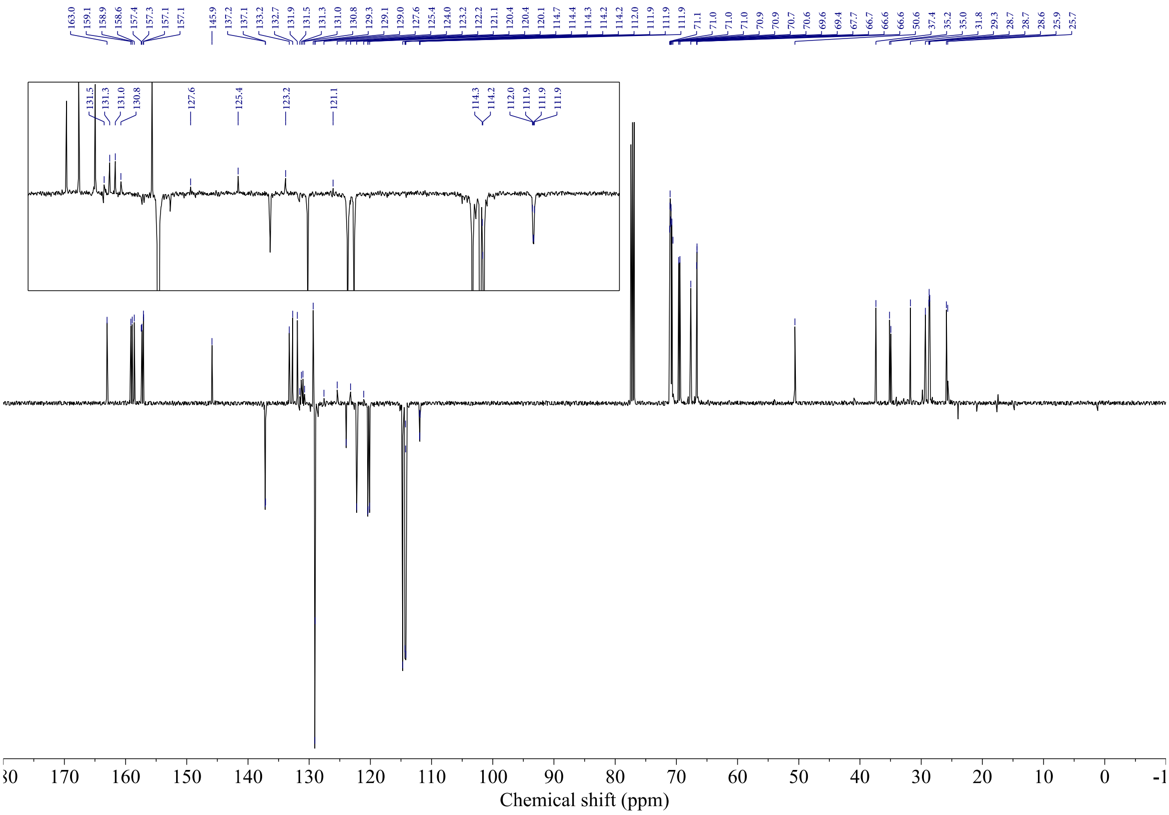

Figure S163. JMOD NMR of *rac*-**6** ($CDCl_3$, 126 MHz, 298 K)

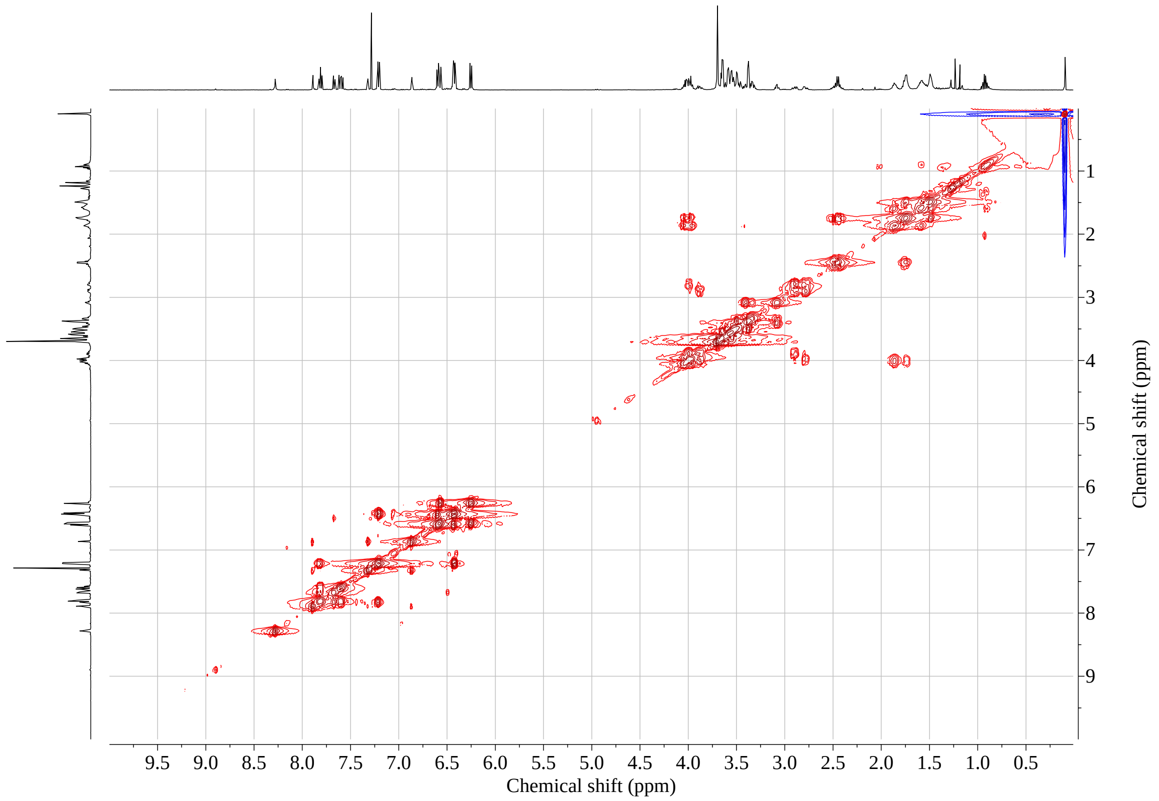

Figure S164. COSY NMR of *rac*-**6** ($CDCl_3$, 298 K)

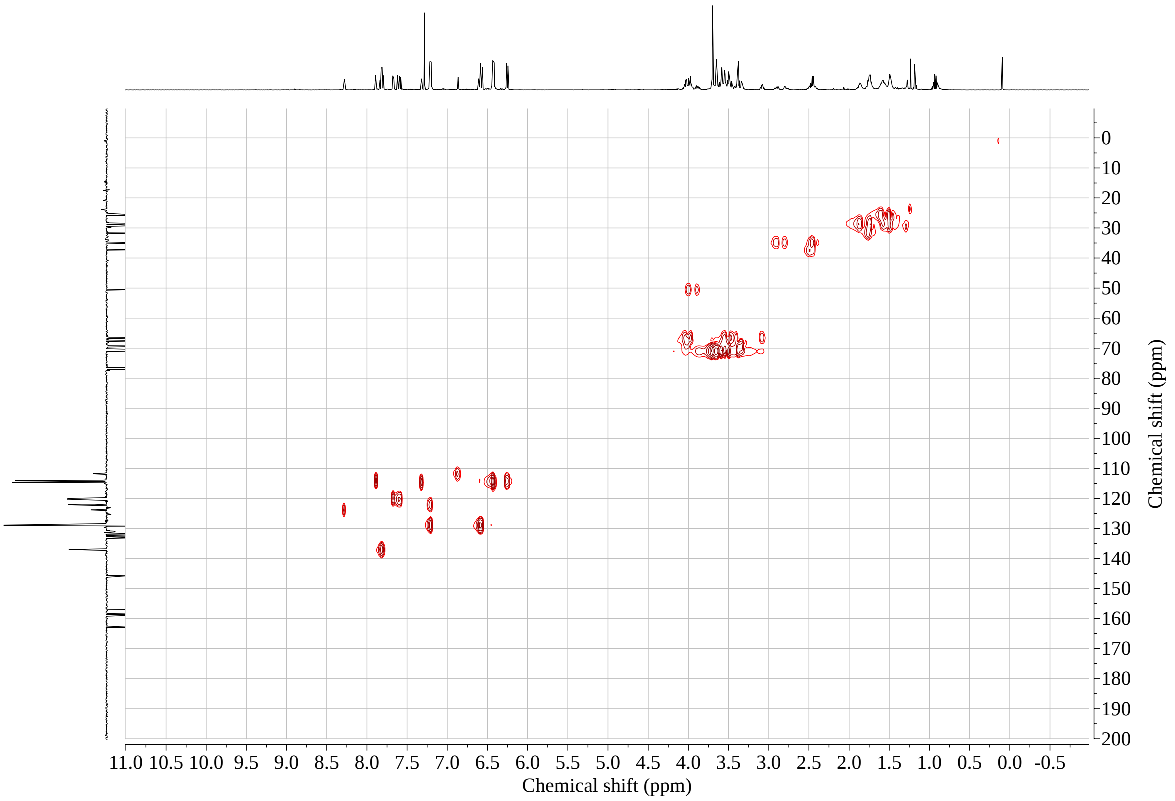

Figure S165. HSQC NMR of *rac*-**6** ($CDCl_3$, 298 K)

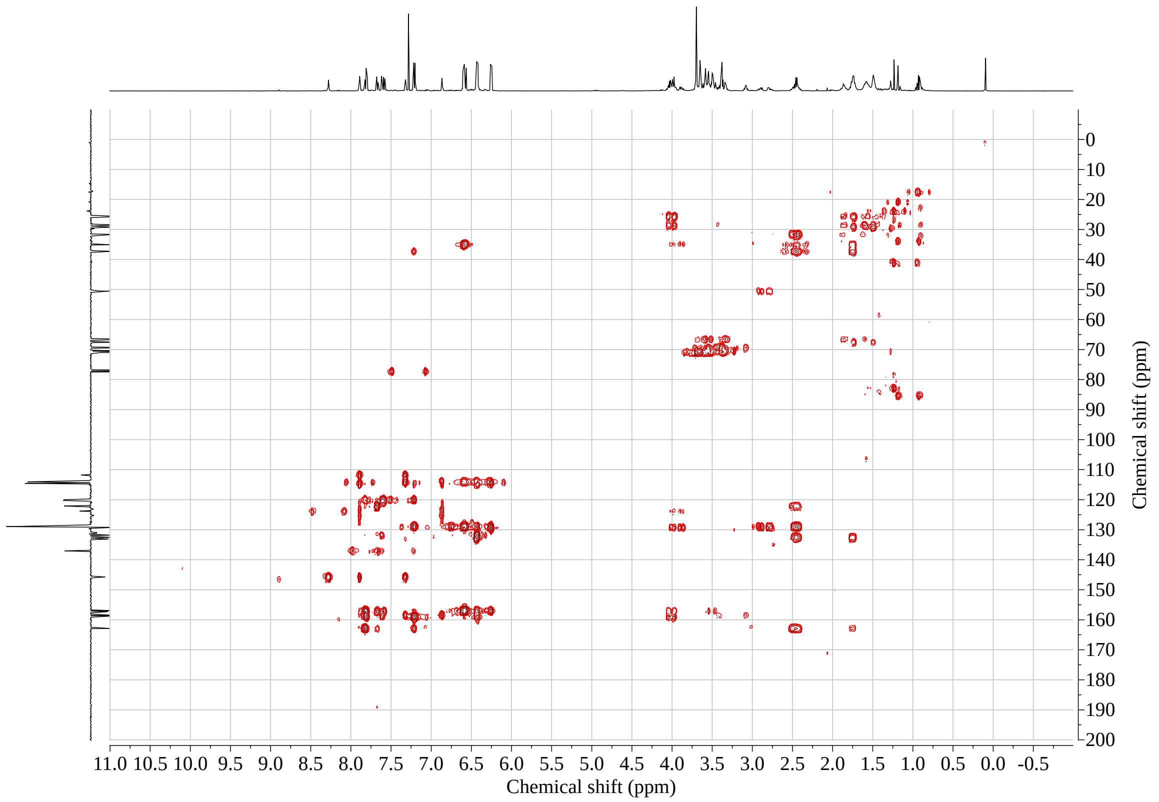

Figure S166. HMBC NMR of *rac*-**6** ($CDCl_3$, 298 K)

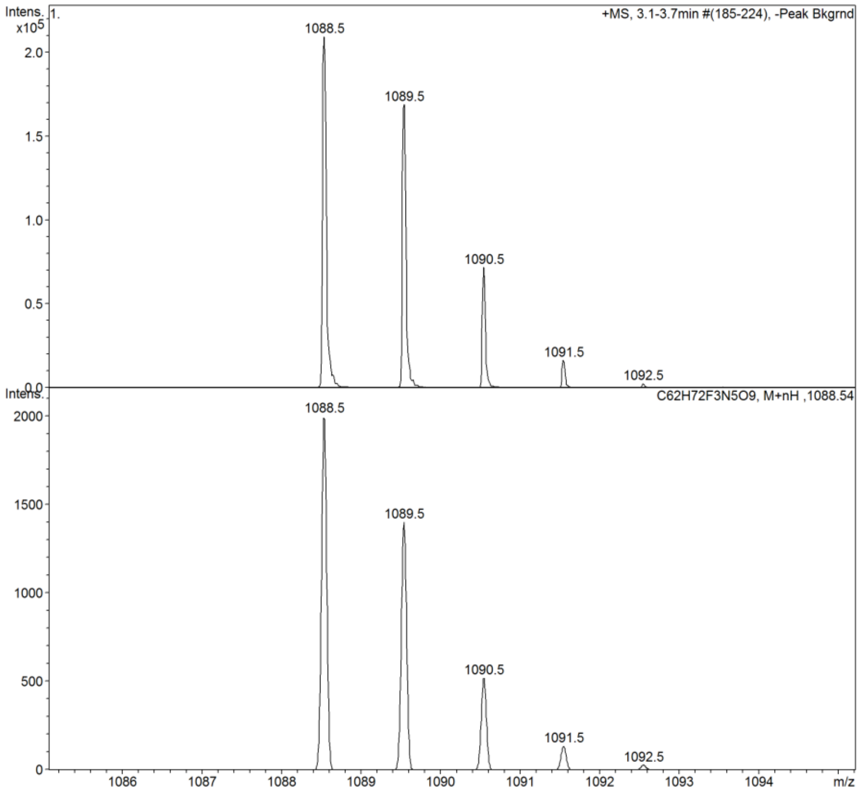

Figure S167. Isotope pattern of *rac*-**6** $C_{62}H_{72}F_3N_5O_9$

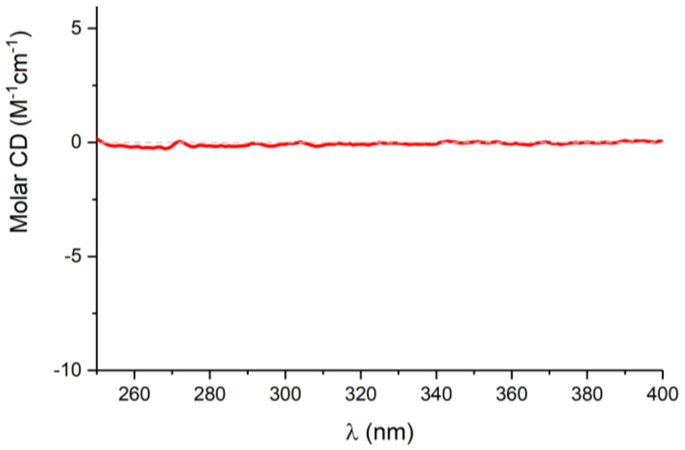

Figure S168. Circular dichroism spectrum of *rac*-**6** (37.5 µM) at 293 K in $CHCl_3$.

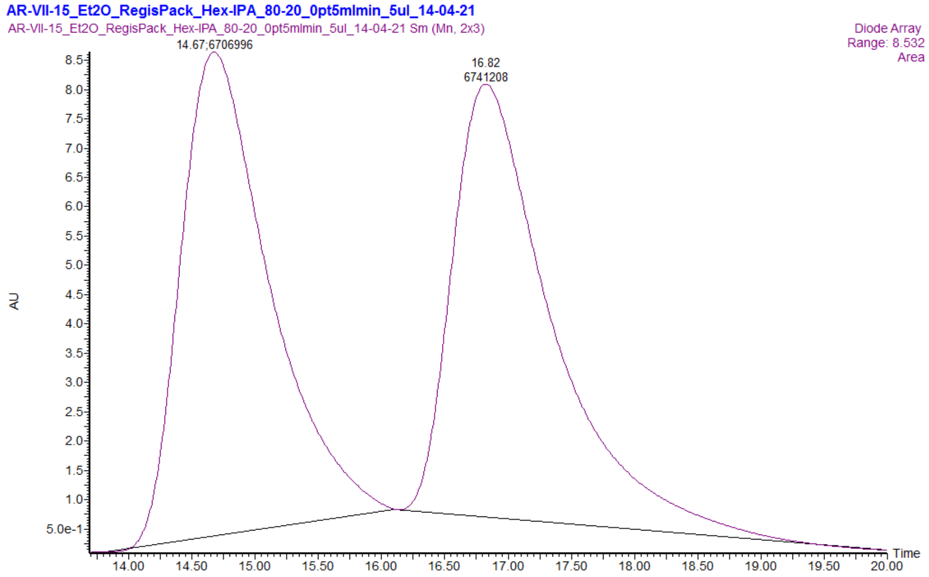

Figure S169. CSP-HPLC of *rac*-**6** (loaded in Et_2O). RegisPack, *n*-hexane-IPA 80 : 20, flowrate 0.5 mLmin^{-1}, retention times *rac*-**6**, (S_{mt})-**6** (14.7 min, 6706996, 49.9%), (R_{mt})-**6** (16.8 min, 6741208, 50.1%).

Catenane (S_{mt})-6

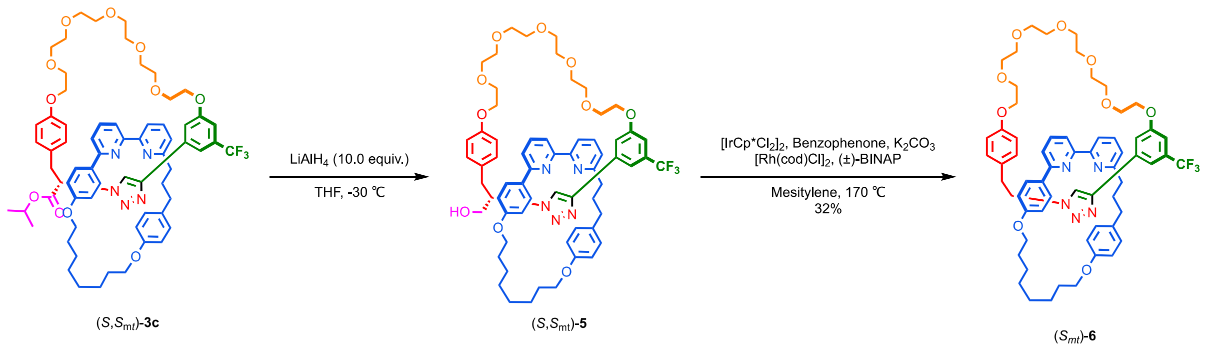

A solution of (S,S_{mt})-**3b** (34 mg, 0.028 mmol, 1.0 equiv.) in degassed THF (0.3 mL) was cooled to -30 ºC in a solid CO_2-acetone bath. $LiAlH_4$ (1 M solution in THF, 0.3 mL, 0.30 mmol, 10.0 equiv.) was added dropwise along the wall of the reaction vessel and reaction mixture stirred at -30 ºC for 1 h. MeOH was added slowly along the wall of the vessel and the resulting mixture allowed to warm to rt. Sat. Rochelle salt solution (5 mL) was added and the resulting mixture was extracted with EtOAc (20 mL). The organic phase was dried ($MgSO_4$) and the solvent removed in vacuo to give a brown oil crude (33 mg) containing catenane (S,S_{mt})-**5** that was used without further purification.

$[IrCp^*Cl_2]_2$ (23 mg, 0.028 mmol, 1.0 equiv.), benzophenone (16 mg, 0.088 mmol, 3.0 equiv.), $[Rh(cod)Cl]_2$ (1.5 mg, 0.0028 mmol, 0.1 equiv.), (±)-BINAP (7 mg, 0.012 mmol, 0.4 equiv.) and K_2CO_3 (16 mg, 0.12 mmol, 4.0 equiv.) and mesitylene (3 mL) were added to the crude. The resulting suspension was degassed by bubbling N_2 for 5 min and then heated with stirring to 170 ºC for 5 h, at which point TLC analysis indicated that the starting material had been. The solution was allowed to cool down to rt and chromatographed directly (petrol-acetone 100:0 → 50:50) to obtain enantioenriched catenane (S_{mt})-**6** as a yellow oil (10 mg, 32% over two steps, 82% *e.e.*). Spectroscopic data were identical to those reported for *rac*-**6** except the circular dichroism spectra and CSP-HPLC.

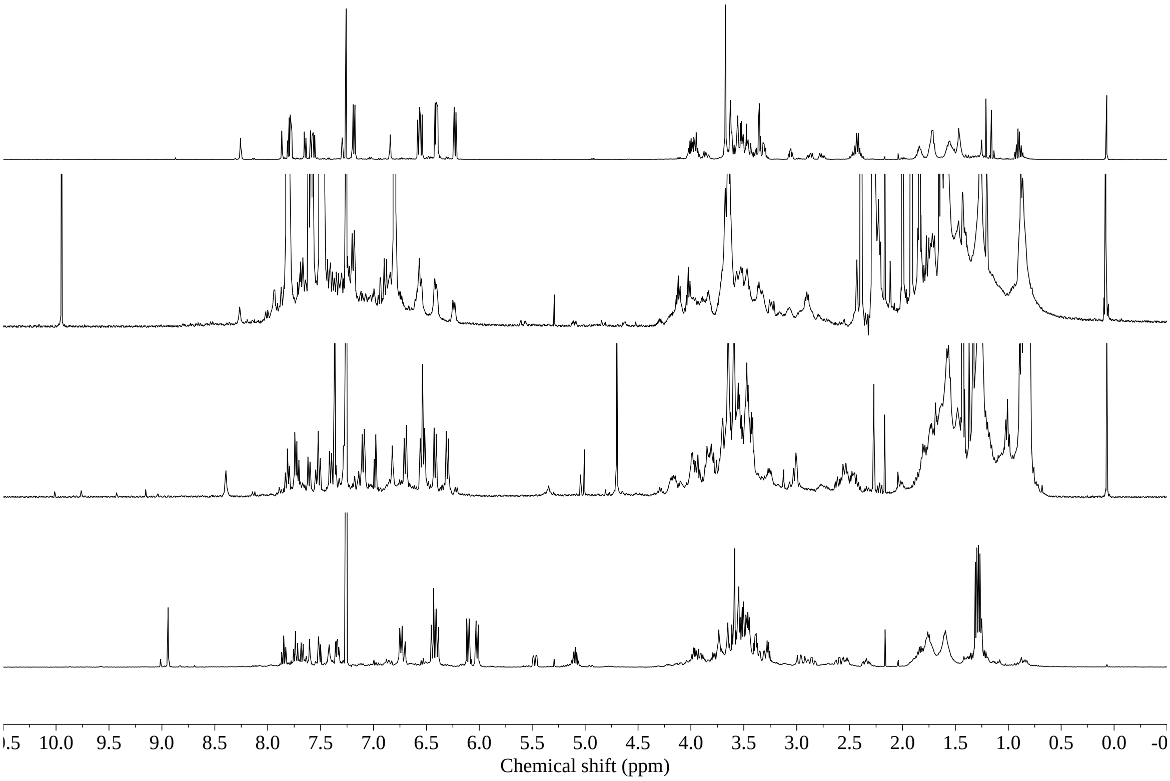

Figure S170. Stacked ^{1}H NMR of (S,S_{mt})-**3c** (bottom), crude (S,S_{mt})-**5** (second from bottom), crude (S_{mt})-**6** (second from top) and *rac*-**6** (top) ($CDCl_3$, 400 MHz, 298 K)

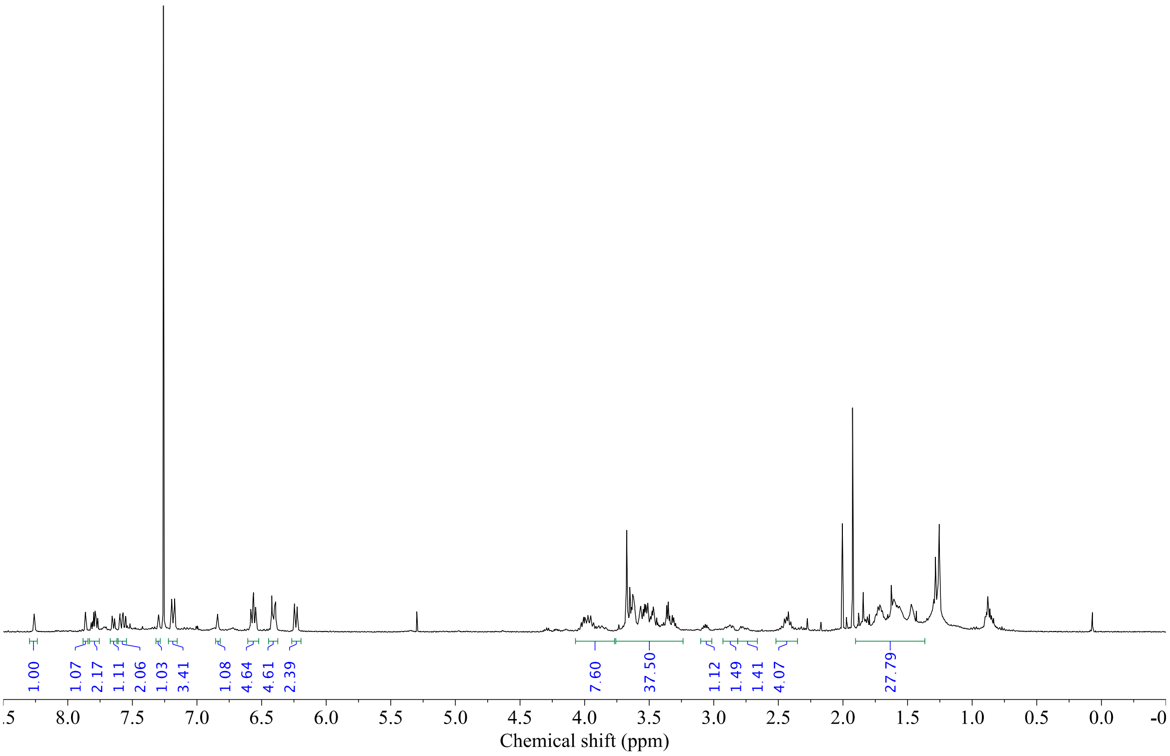

Figure S171. ^{1}H NMR of (S_{mt})-**6** ($CDCl_3$, 400 MHz, 298 K)

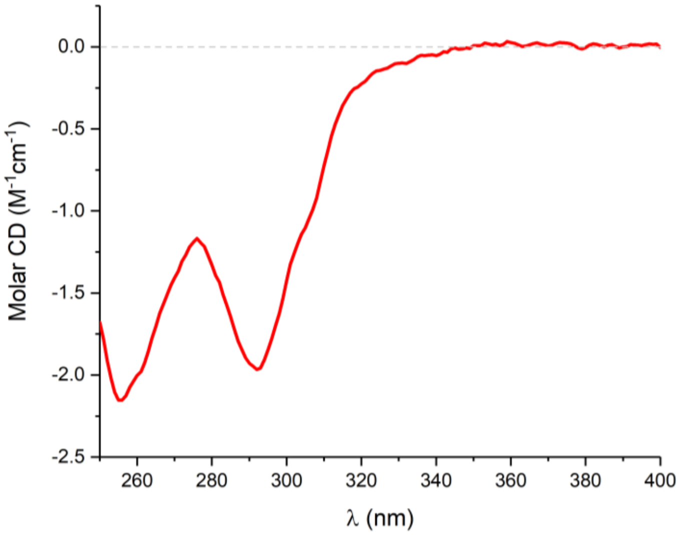

Figure S172. Circular dichroism spectrum of (S_{mt})-**6** (73.5 µM, *e.r.* (S_{mt})-**6** : (R_{mt})-**6** 91 : 9) at 293 K in $CHCl_3$

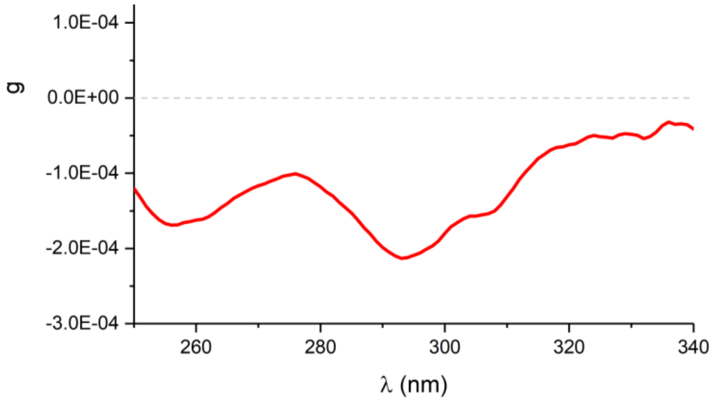

Figure S173. g_{abs} plot of (S_{mt})-**6** (73.5 µM, *e.r.* (S_{mt})-**6** : (R_{mt})-**6** 91 : 9) at 293 K in $CHCl_3$. g_{abs}(max) = 2 × 10^{-4} (293 nm).

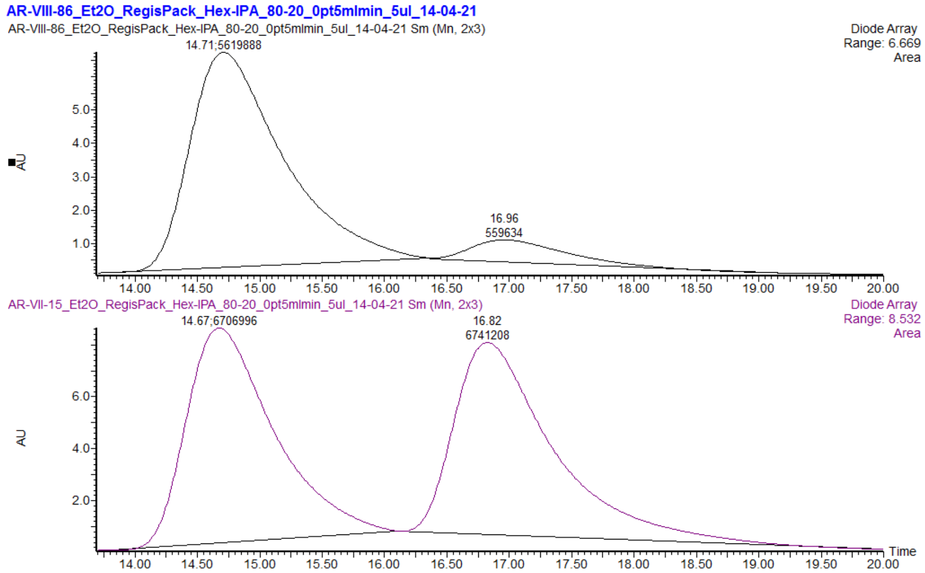

Figure S174. CSP-HPLC of (S_{mt})-**6** (loaded in Et_2O). RegisPack, *n*-hexane-IPA 80 : 20, flowrate 0.5 $mLmin^{-1}$, retention times (S_{mt})-**6**, (S_{mt})-**6** (14.7 min, 5619888, 90.9%), (R_{mt})-**6** (16.9 min, 559634, 9.1%).

S4. SYNTHESIS OF CATENANE 9 (SCHEME 3, MAIN TEXT)

Synthesis of catenane precursors (*S*)-7 and 10

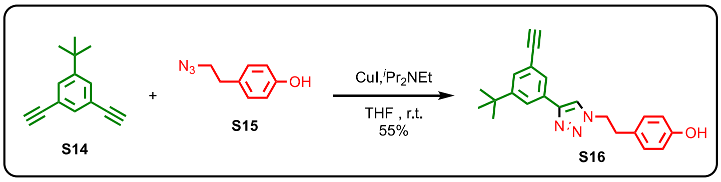

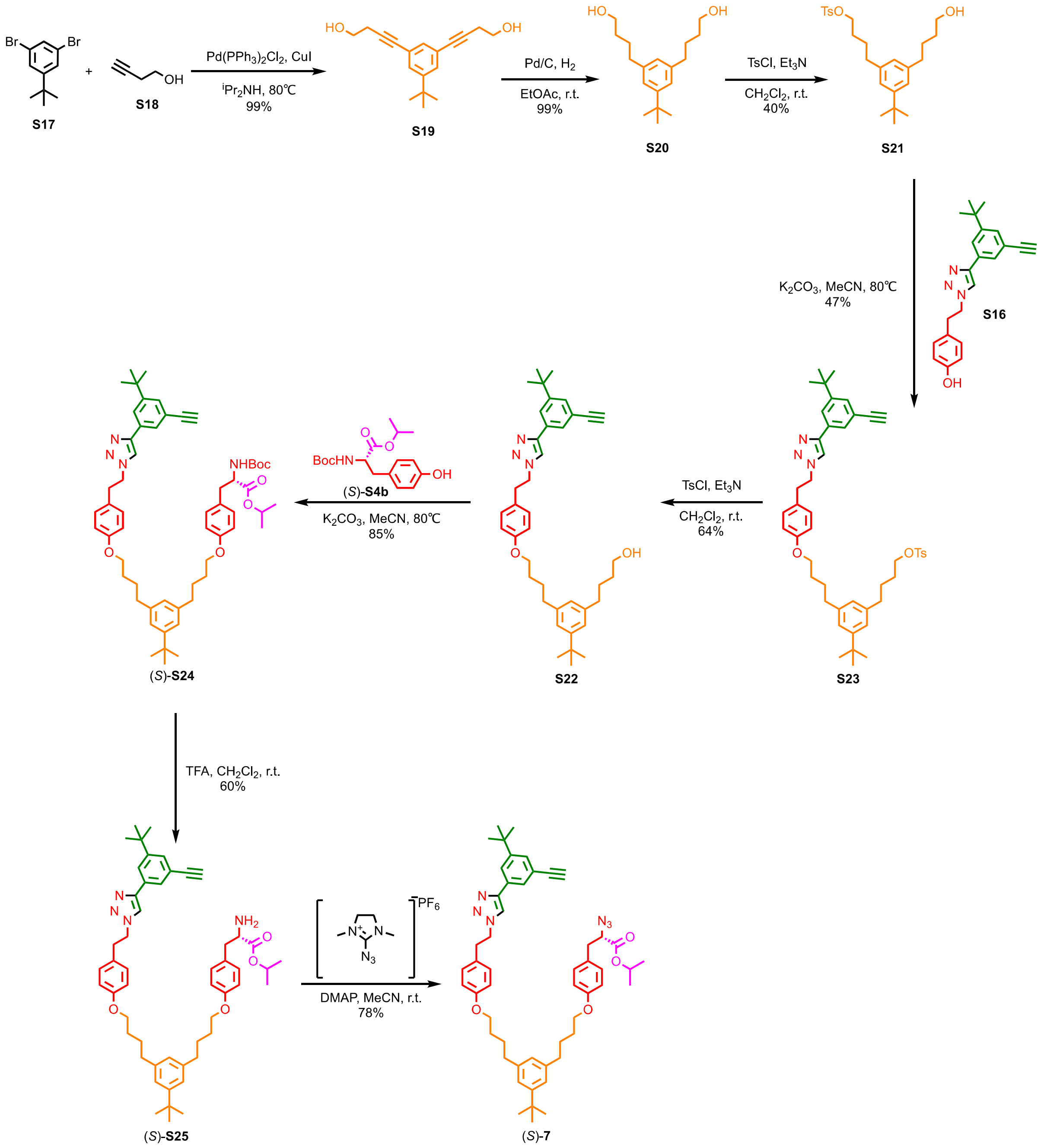

Scheme S3. Synthetic route to macrocycle precursor (*S*)-**7**.

Triazole S16

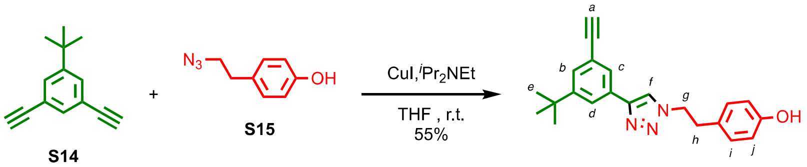

S14 (910 mg, 5.0 mmol, 2.5 equiv.), **S15** (326 mg, 2.0 mmol, 1.0 equiv.) and CuI (15 mg, 0.1 mmol, 0.05 equiv.) were dissolved in degassed THF (20 mL). ^{i}Pr$_2$NEt (350 µL, 2.0 mmol, 1.0 eq.) was added and the reaction mixture was stirred at rt for 16 h. The solvent was removed *in vacuo*. Column chromatography (petrol-EtOAc 70 : 30 → 50 : 50) gave **S16** as a yellow oil (379 mg, 55%).

^{1}H NMR (400 MHz, $CDCl_3$, 298 K) δ: 7.91 (t, *J* = 1.8, 1H, H$_d$), 7.60 (t, *J* = 1.5, 1H, H$_c$), 7.52 (s, 1H, H$_f$), 7.48 (t, *J* = 1.7, 1H, H$_b$), 6.95 (d, *J* = 8.5, 2H, H$_i$), 6.78 (d, *J* = 8.5, 2H, H$_j$), 4.59 (t, *J* = 7.1, 2H, H$_g$), 3.17 (t, *J* = 7.1, 2H, H$_h$), 3.07 (s, 1H, H$_a$), 1.33 (s, 9H, H$_e$).

^{13}C NMR (101 MHz, $CDCl_3$, 298 K) δ: 155.3, 152.4, 147.1, 130.5, 130.0, 129.2, 128.7, 126.7, 123.7, 122.3, 120.4, 115.9, 84.0, 77.0, 52.3, 36.0, 35.0, 31.3.

HR-ESI-MS *m/z* = 346.1917 [M+H]$^+$ calc. 346.1914 for $C_{22}H_{24}N_3O$.

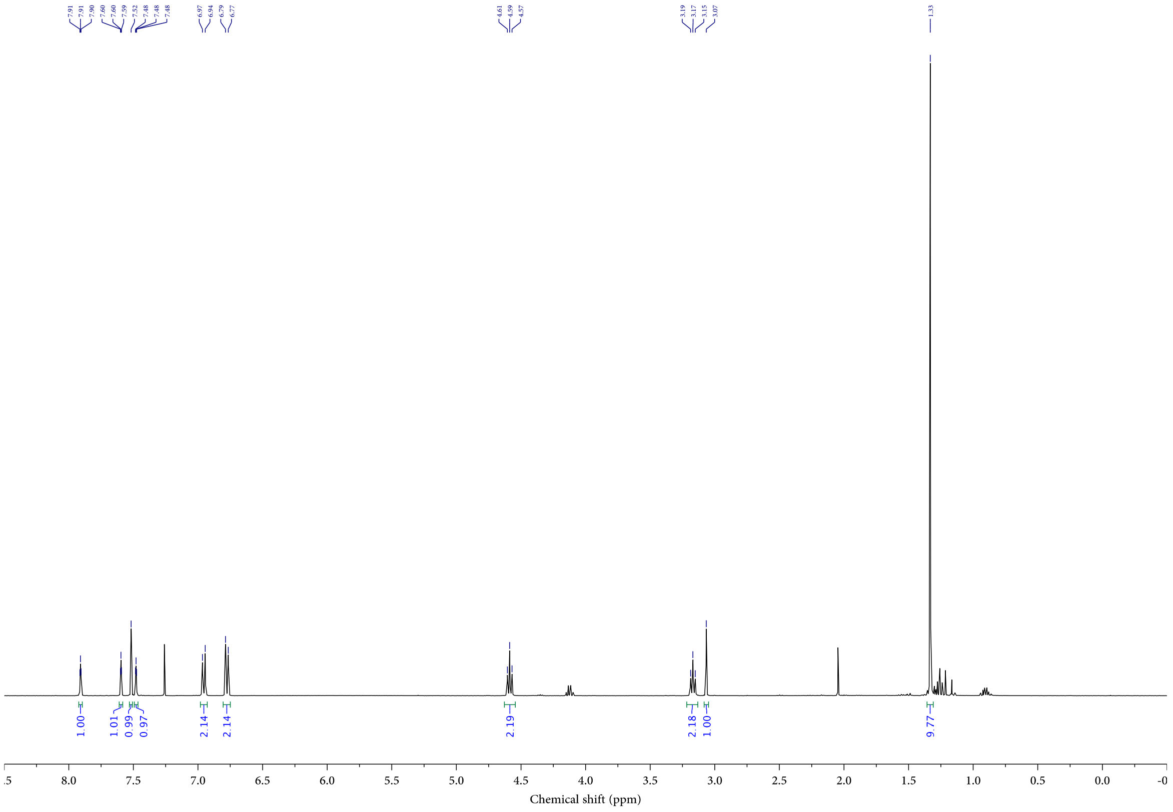

Figure S175. ^{1}H NMR of **S16** ($CDCl_3$, 400 MHz, 298 K)

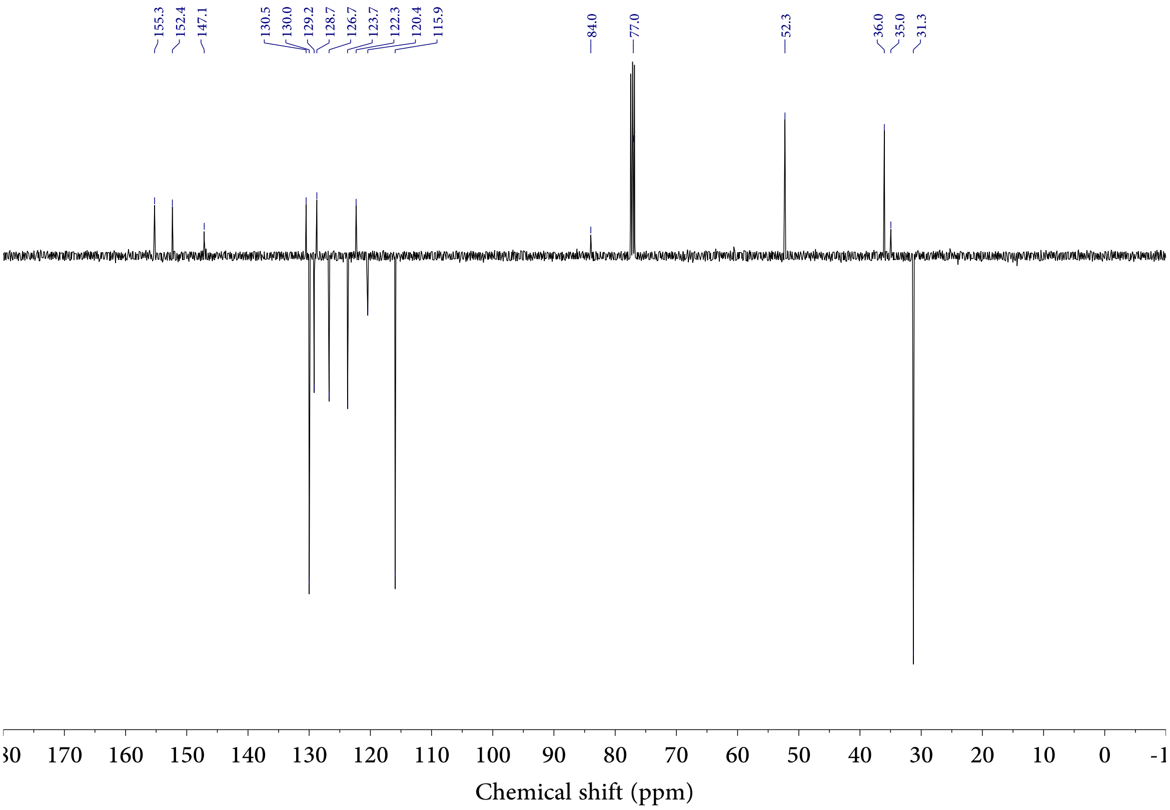

Figure S176. JMOD NMR of **S16** ($CDCl_3$, 101 MHz, 298 K)

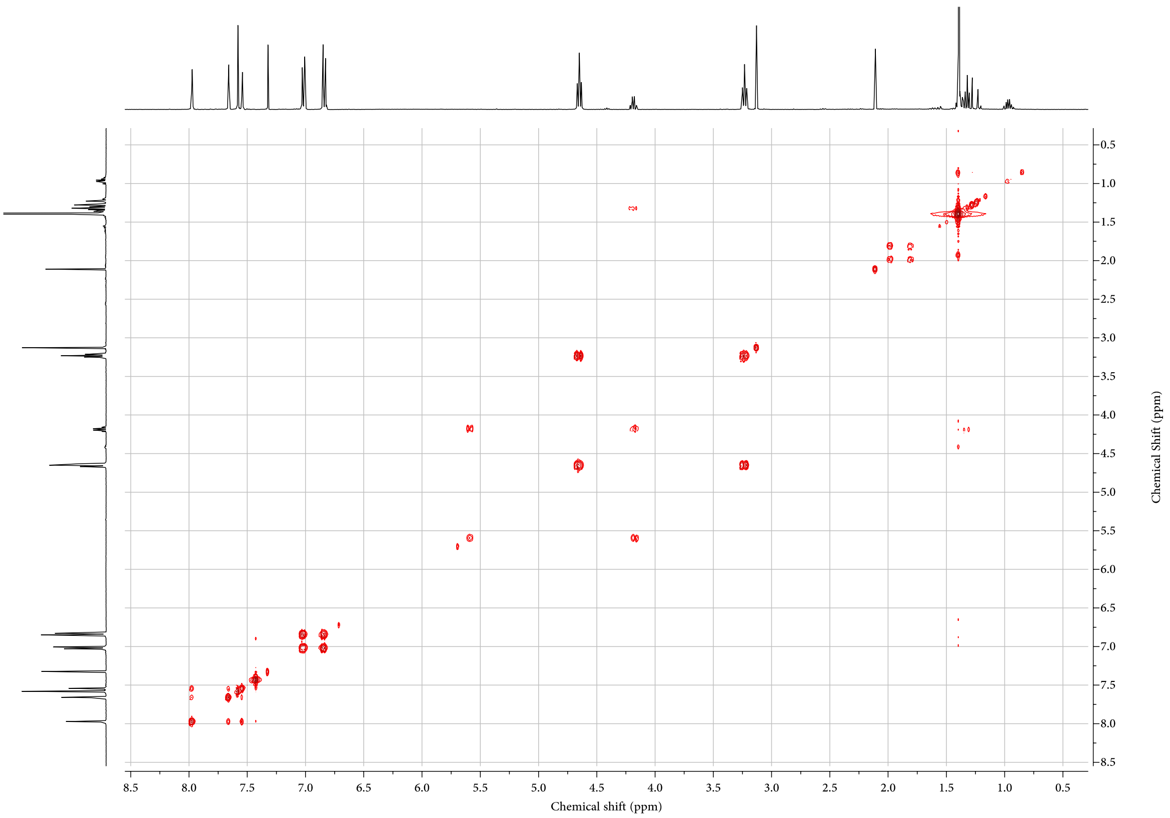

Figure S177. COSY NMR of **S16** ($CDCl_3$, 298 K)

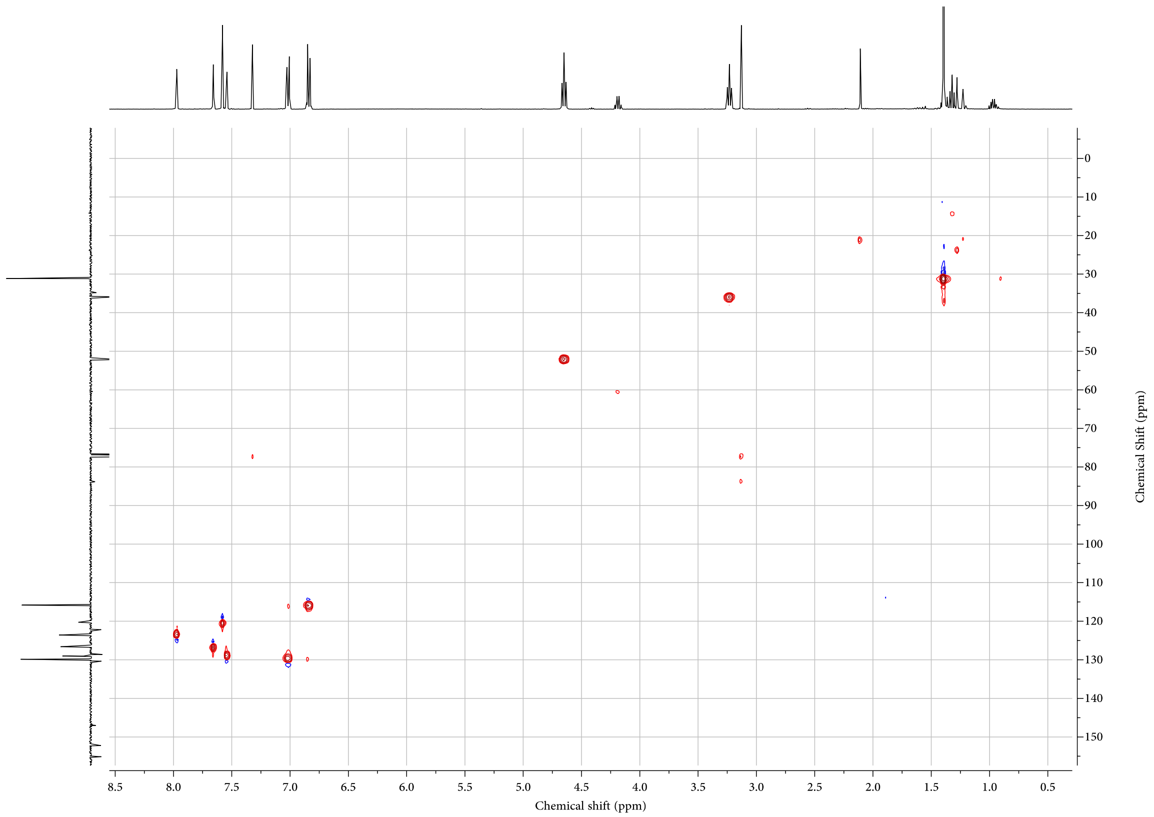

Figure S178. HSQC NMR of **S16** ($CDCl_3$, 298 K)

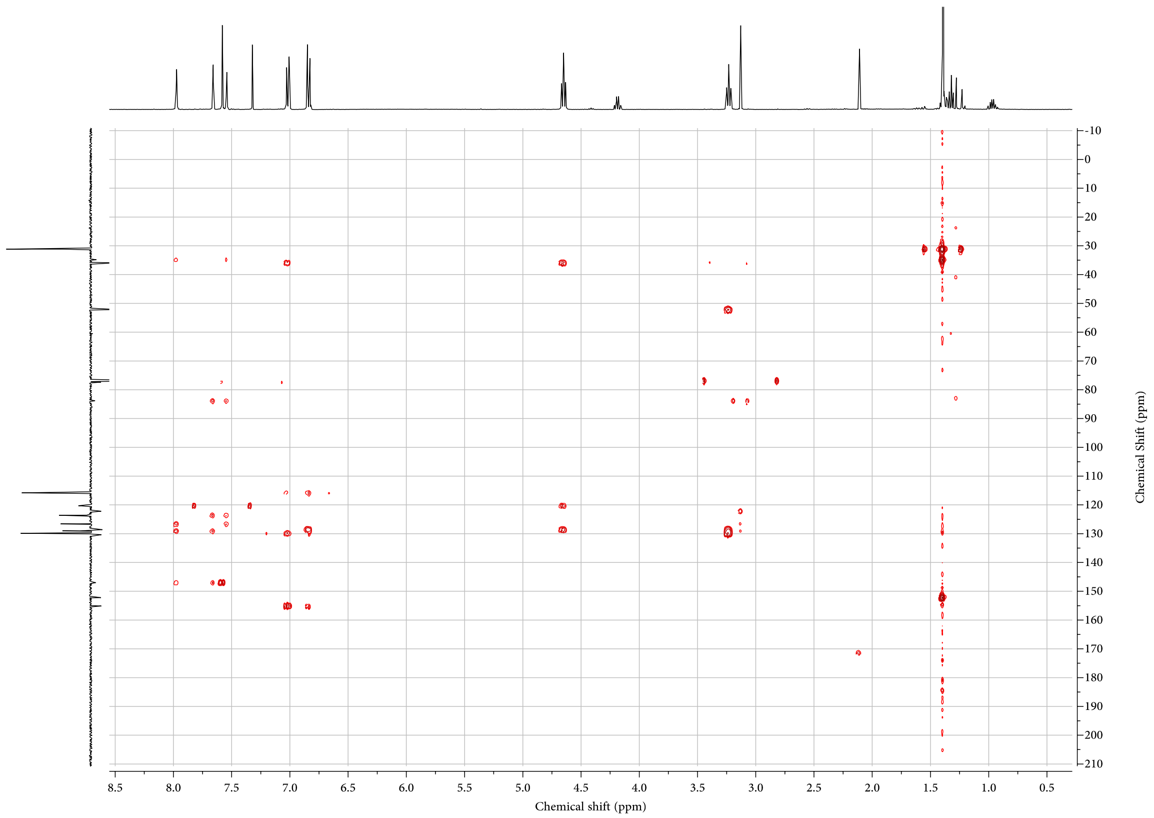

Figure S179. HMBC NMR of **S16** ($CDCl_3$, 298 K)

Diol S19

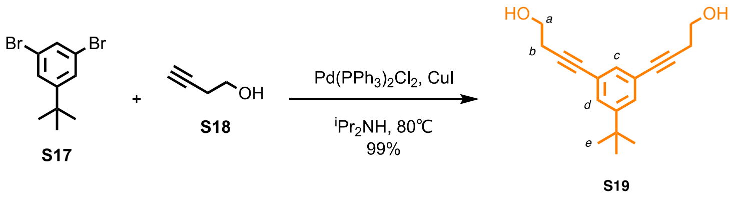

S17 (3.0 g, 10.3 mmol, 1.0 equiv.), $Pd(PPh_3)_2Cl_2$ (145 mg, 0.2 mmol, 0.02 equiv.) and CuI (77 mg, 0.4 mmol, 0.04 equiv.) were suspended in $^iPr_2NH$ (50 mL) and the mixture degassed by bubbling N_2 for 5 min. **S18** (2.2 g, 31 mmol, 3.0 equiv.) was added and the mixture was stirred at 80 °C for 16 h. The solvent was removed *in vacuo*. Column chromatography (petrol-EtOAc 90 : 10 → 60 : 40) gave **S19** as an orange oil (2.75 g, 99%).

^{1}H NMR (400 MHz, $CDCl_3$, 298 K) δ: 7.36 (d, *J* = 1.5, 2H, H_d), 7.30 (t, *J* = 1.5, 1H, H_c), 3.81 (t, *J* = 6.2, 4H, H_a), 2.69 (t, *J* = 6.2, 4H, H_b), 1.29 (s, 9H, H_e).

^{13}C NMR (101 MHz, $CDCl_3$, 298 K) δ: 151.6, 132.1, 128.7, 123.2, 86.4, 82.4, 61.3, 34.8, 31.2, 24.0.

HR-ESI-MS *m/z* = 293.1511 $[M+Na]^+$ calc. 293.1512 for $C_{18}H_{22}NaO_2$.

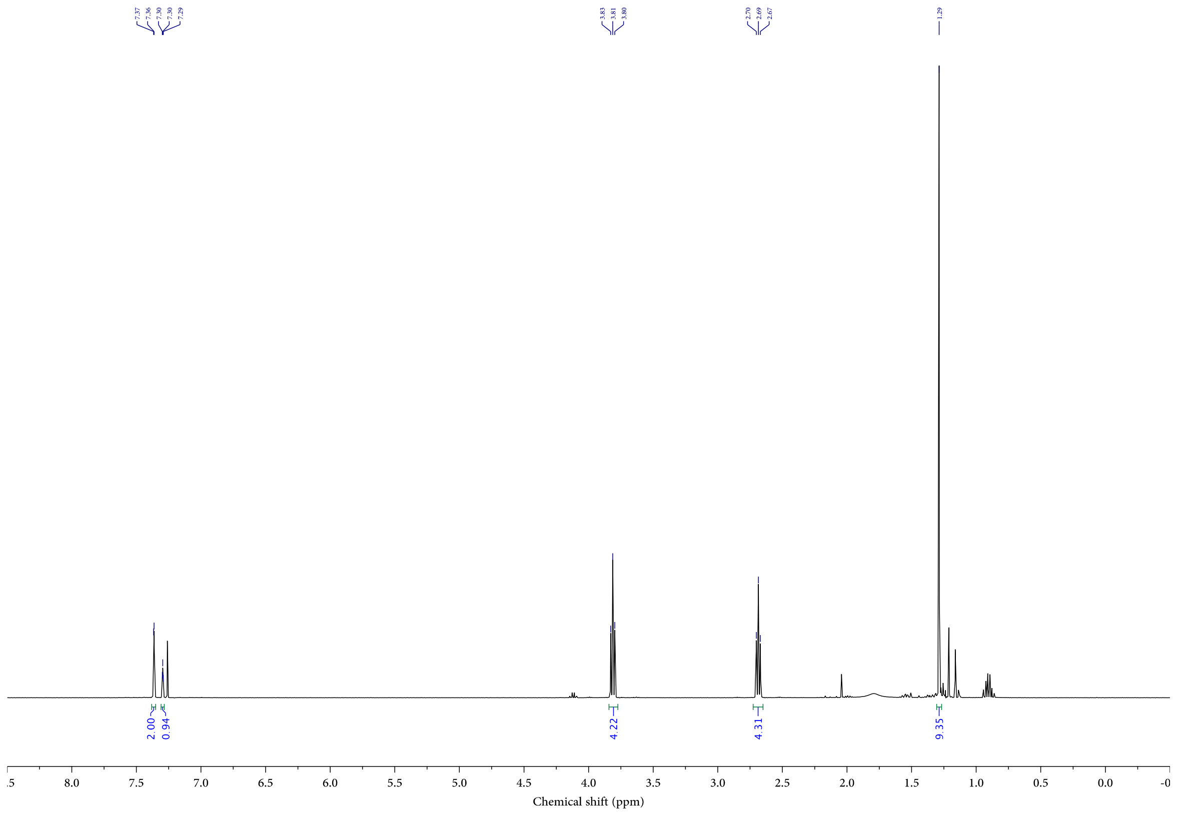

Figure S180. ^{1}H NMR of **S19** ($CDCl_3$, 400 MHz, 298 K)

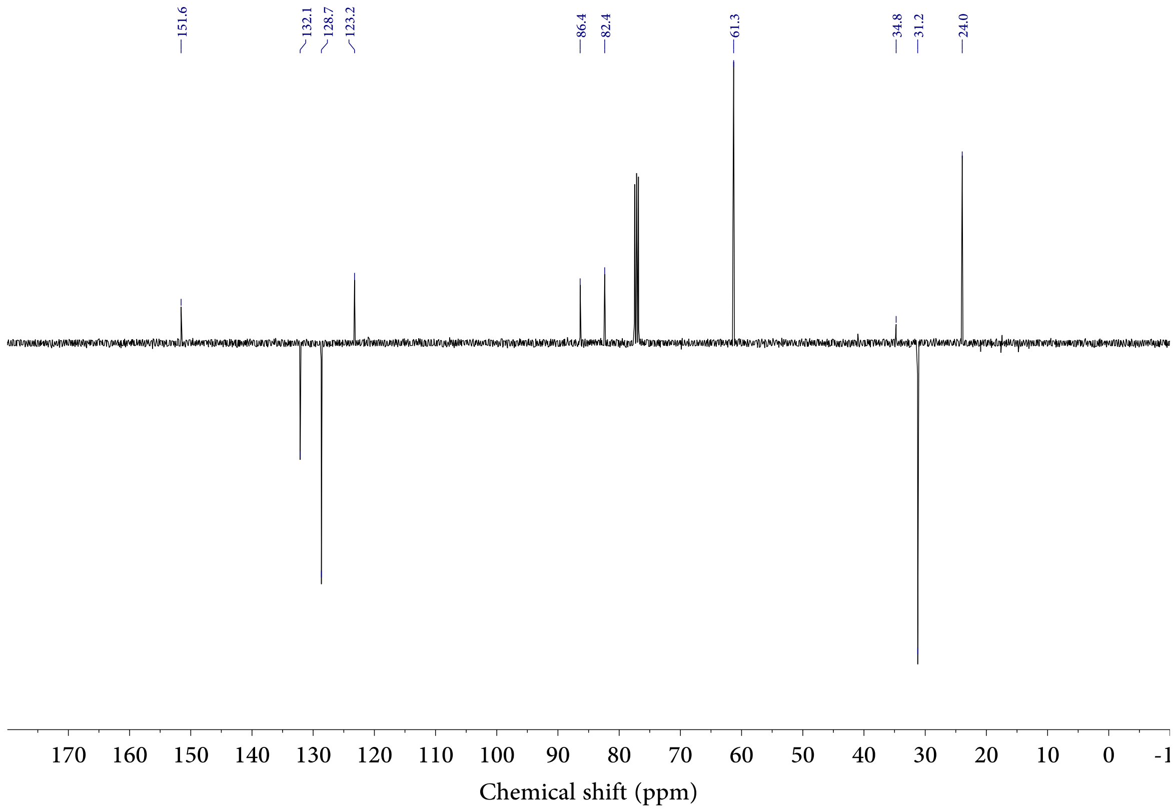

Figure S181. JMOD NMR of **S19** ($CDCl_3$, 101 MHz, 298 K)

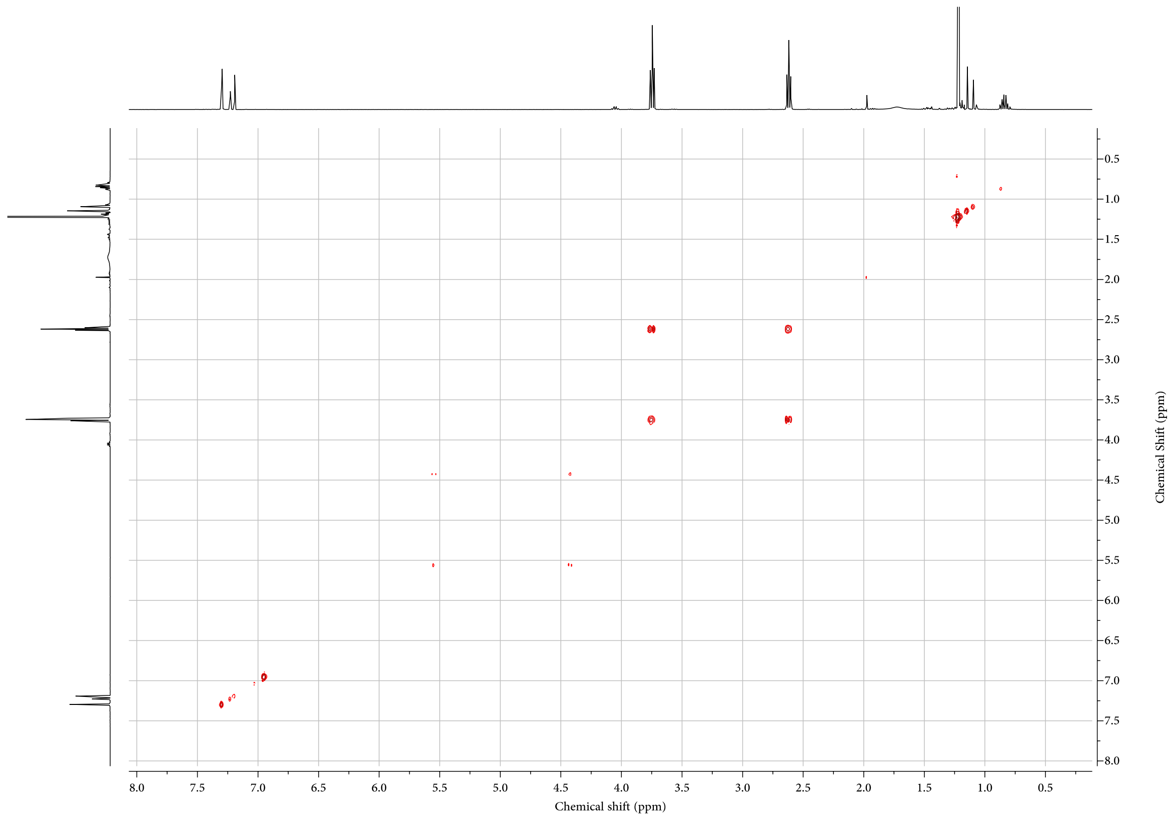

Figure S182. COSY NMR of **S19** ($CDCl_3$, 298 K)

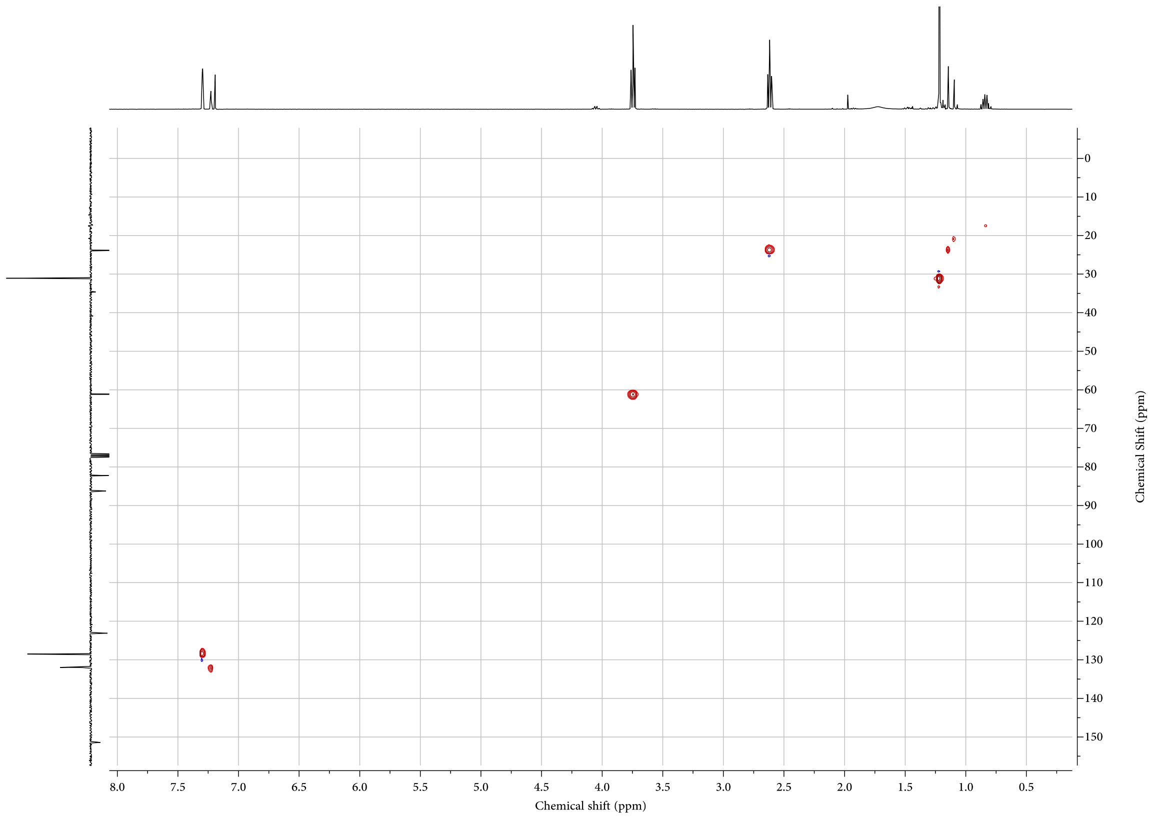

Figure S183. HSQC NMR of **S19** ($CDCl_3$, 298 K)

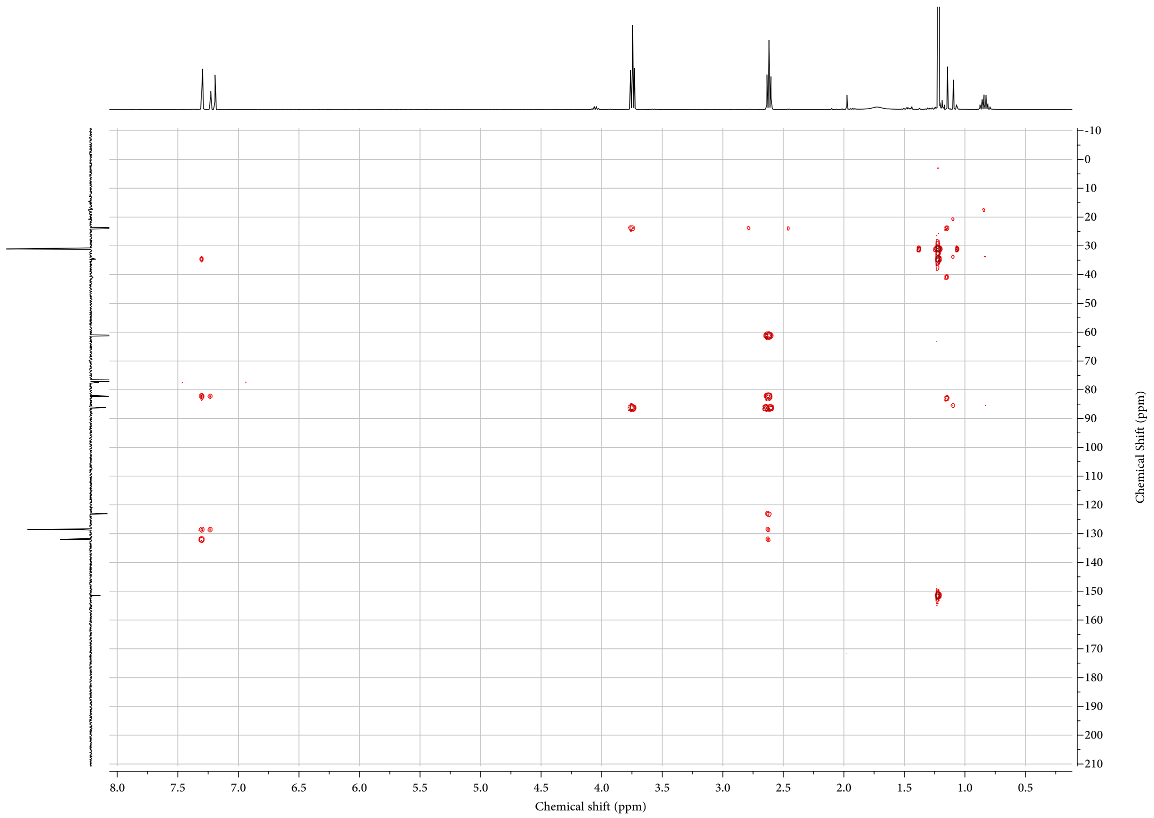

Figure S184. HMBC NMR of **S19** ($CDCl_3$, 298 K)

Diol S20

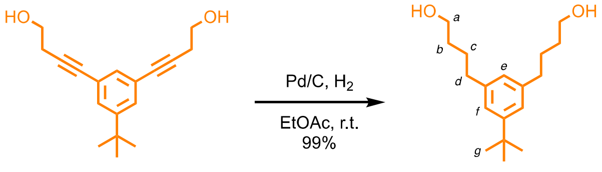

S19 S20

S19 (2.75 g, 10.2 mmol, 1.0 equiv.) and Pd/C (10% w/w, 205 mg) were suspended in EtOAc (100 mL) and the mixture was degassed by bubbling N_2 over 5 min. H_2 was bubbled through the solution for 10 min at rt before an balloon of H_2 was attached to the reaction vessel and the reaction mixture stirred at rt for 16 h. The reaction mixture was filtered over Celite® and washed through with EtOAc (30 mL). The combined washings were reduced *in vacuo* to obtain **S20** as a yellow oil (2.8 g, 99%) that was used without further purification.

^{1}H NMR (400 MHz, $CDCl_3$, 298 K) δ: 7.02 (d, J = 1.6, 2H, H_f), 6.83 (t, J = 1.7, 1H, H_e), 3.66 (t, J = 6.4, 4H, H_a), 2.61 (t, J = 7.5, 4H, H_d), 1.77-1.55 (m, 8H, H_b, H_c), 1.52 (br s, 2H, OH), 1.30 (s, 9H, H_g).

^{13}C NMR (101 MHz, $CDCl_3$, 298 K) δ: 151.3, 142.0, 125.7, 123.1, 63.0, 36.1, 34.7, 32.6, 31.6, 27.8.

HR-ESI-MS m/z = 301.2144 $[M+Na]^+$ calc. 301.2138 for $C_{18}H_{30}NaO_2$.

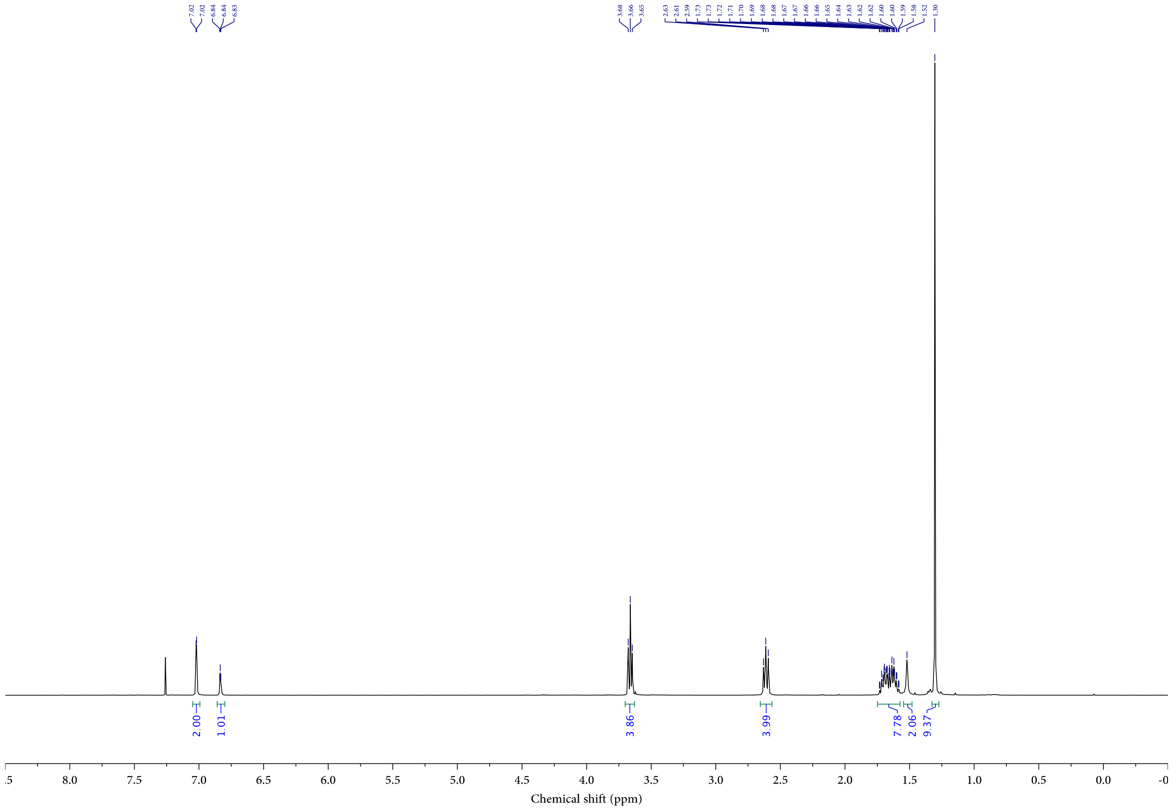

Figure S185. ^{1}H NMR of **S20** ($CDCl_3$, 400 MHz, 298 K)

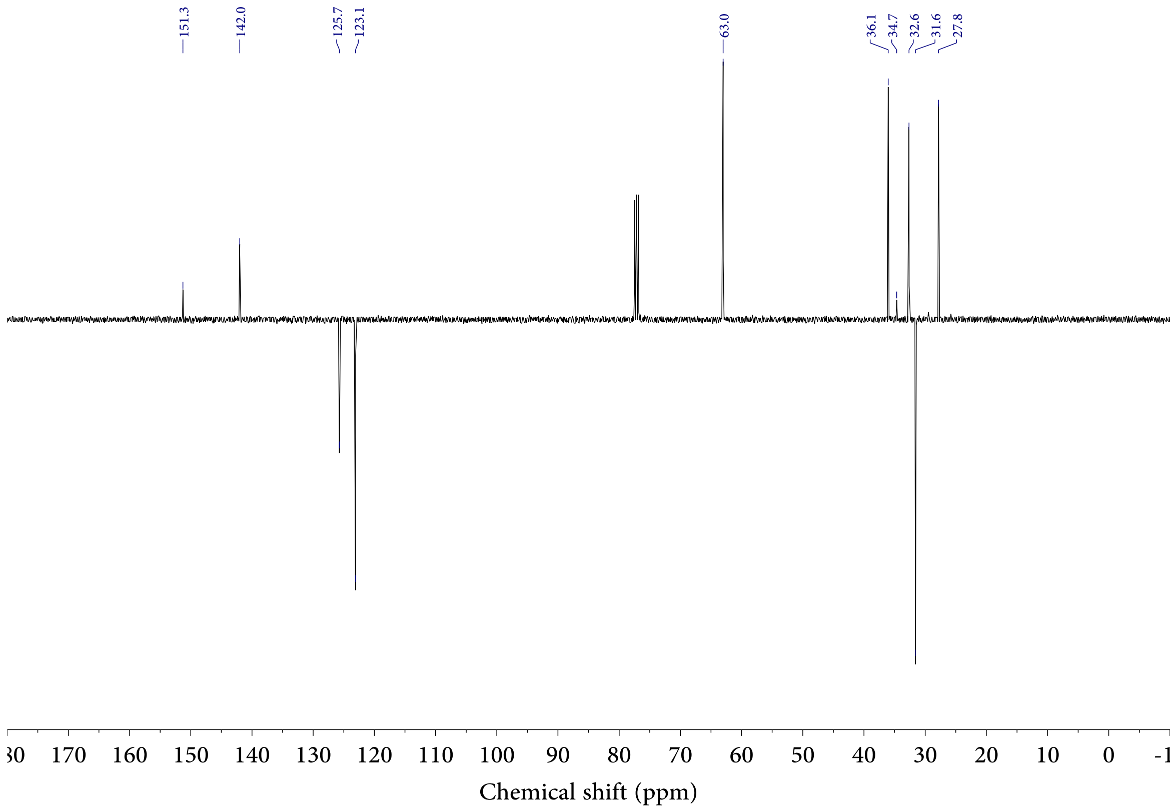

Figure S186. JMOD NMR of **S20** ($CDCl_3$, 101 MHz, 298 K)

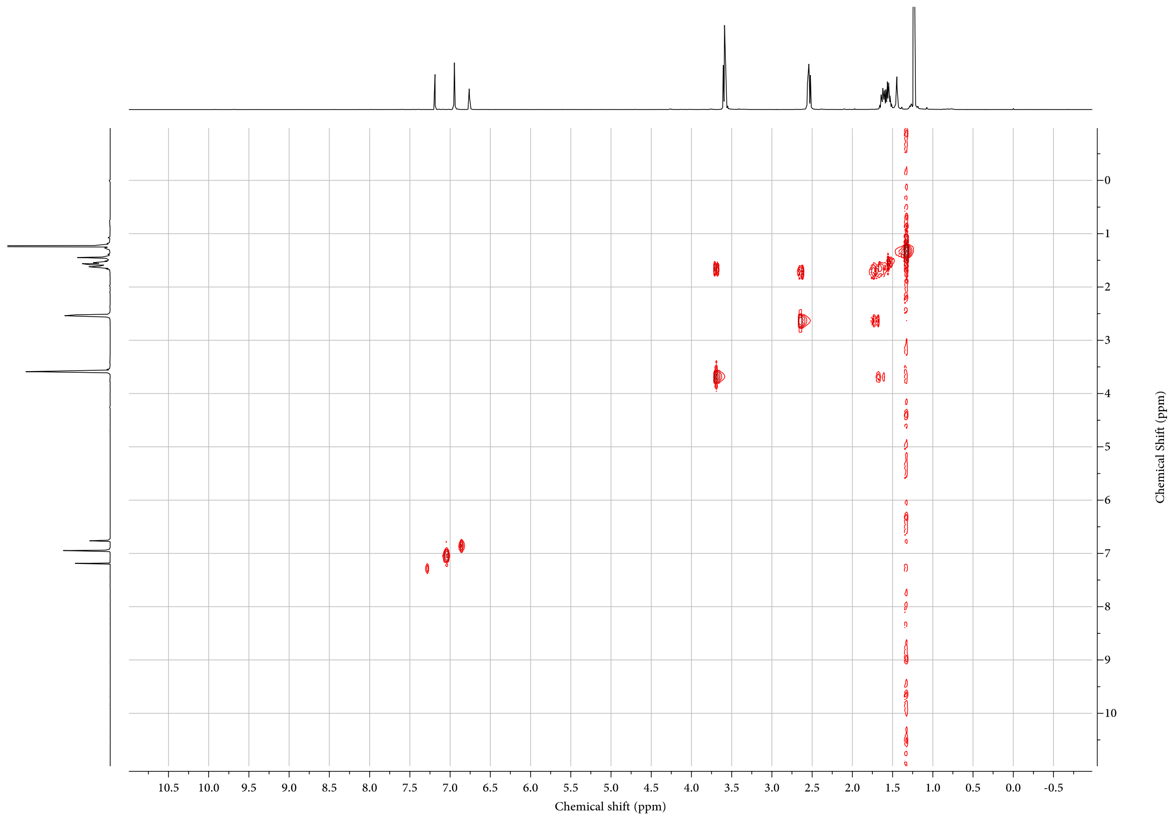

Figure S187. COSY NMR of **S20** ($CDCl_3$, 298 K)

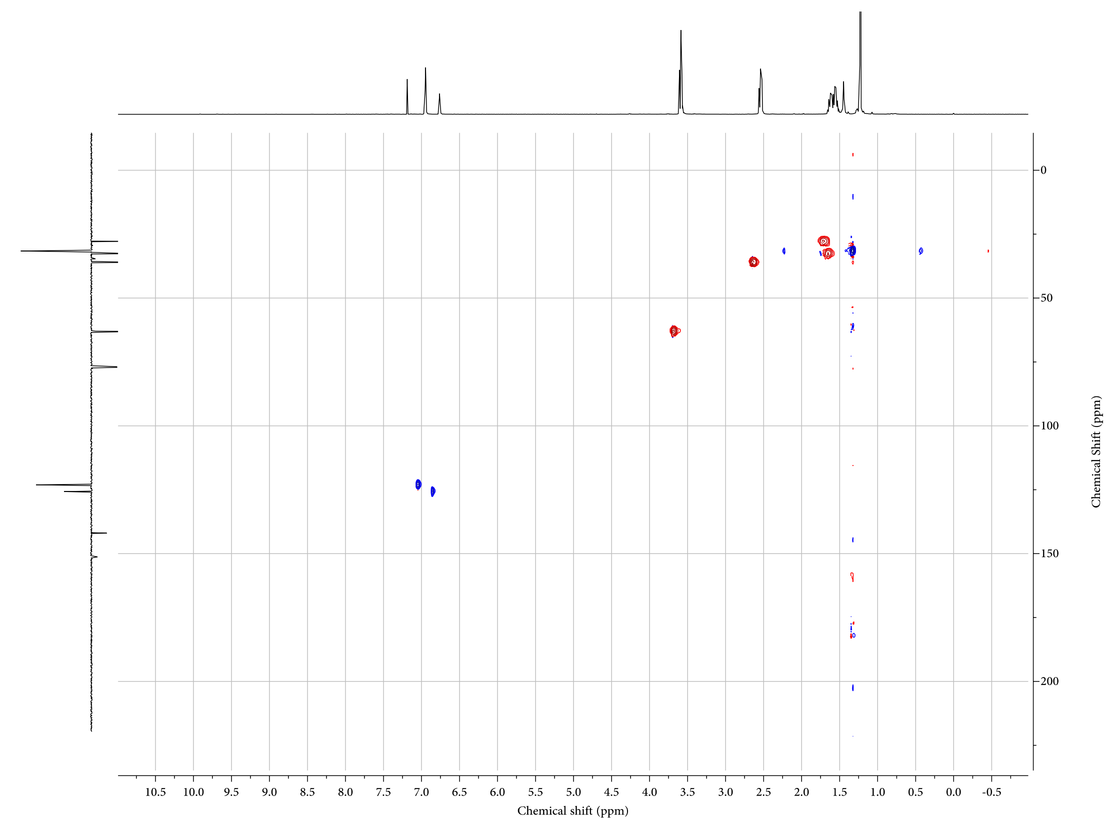

Figure S188. HSQC NMR of **S20** ($CDCl_3$, 298 K)

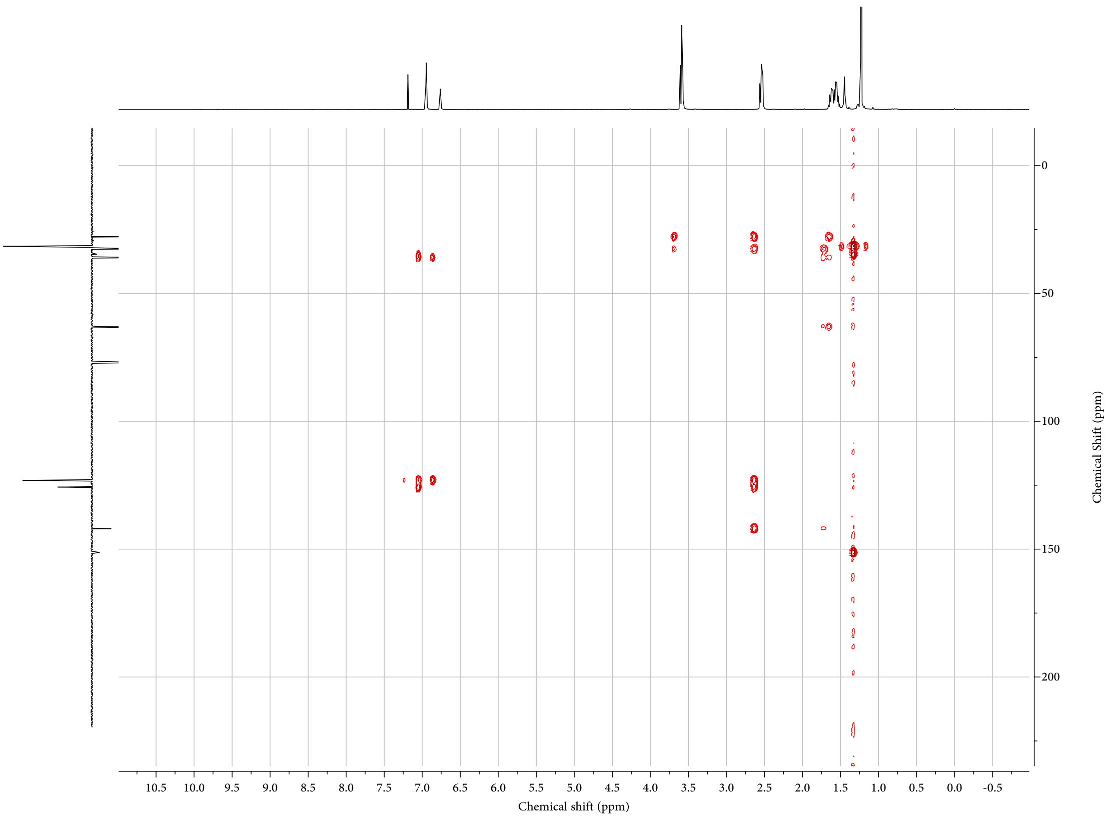

Figure S189. HMBC NMR of **S20** ($CDCl_3$, 298 K)

Tosylate S21

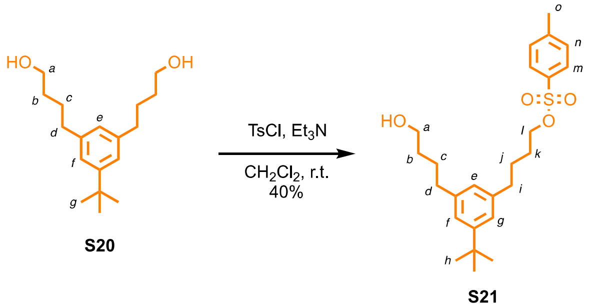

S20 (1.0 g, 3.6 mmol, 1.0 equiv.) and triethylamine (1.5 mL, 11 mmol, 3.0 equiv.) were dissolved in CH_2Cl_2 (36 mL). TsCl (685 mg, 3.6 mmol, 1.0 equiv.) was added and the reaction mixture was stirred at rt for 16 h. The solvent was removed *in vacuo*. Column chromatography (petrol-EtOAc 100:0 → 80:20 → 50:50→ 0:100) gave **S21** as a colorless oil (611 mg, 40% yield).

^{1}H NMR (400 MHz, $CDCl_3$, 298 K) δ: 7.79 (d, J = 8.3, 2H, H_m), 7.34 (d, J = 8.0, 2H, H_n), 7.02 (t, J = 1.8, 1H, H_f), 6.95 (t, J = 1.8, 1H, H_g), 6.77 (t, J = 1.7, 1H, H_e), 4.04 (t, J = 6.0, 2H, H_l), 3.67 (t, J = 6.3, 2H, H_a), 2.60 (t, J = 7.4, 2H, H_d), 2.53 (t, J = 7.3, 2H, H_i), 2.44 (s, 3H, H_o), 1.86 (br s, 1H, OH), 1.74-1.59 (m, 8H, H_b, H_c, H_j, H_k), 1.29 (s, 9H, H_h).

^{13}C NMR (101 MHz, $CDCl_3$, 298 K) δ: 151.3, 144.8, 142.1, 141.3, 133.3, 129.9, 128.0, 125.6, 123.2, 123.0, 70.6, 63.0, 36.0, 35.5, 34.6, 32.6, 31.6, 28.6, 27.8, 27.4, 21.8.

HR-ESI-MS m/z = 433.2409 [M+H]+ calc. 433.2407 for $C_{25}H_{37}O_4S$.

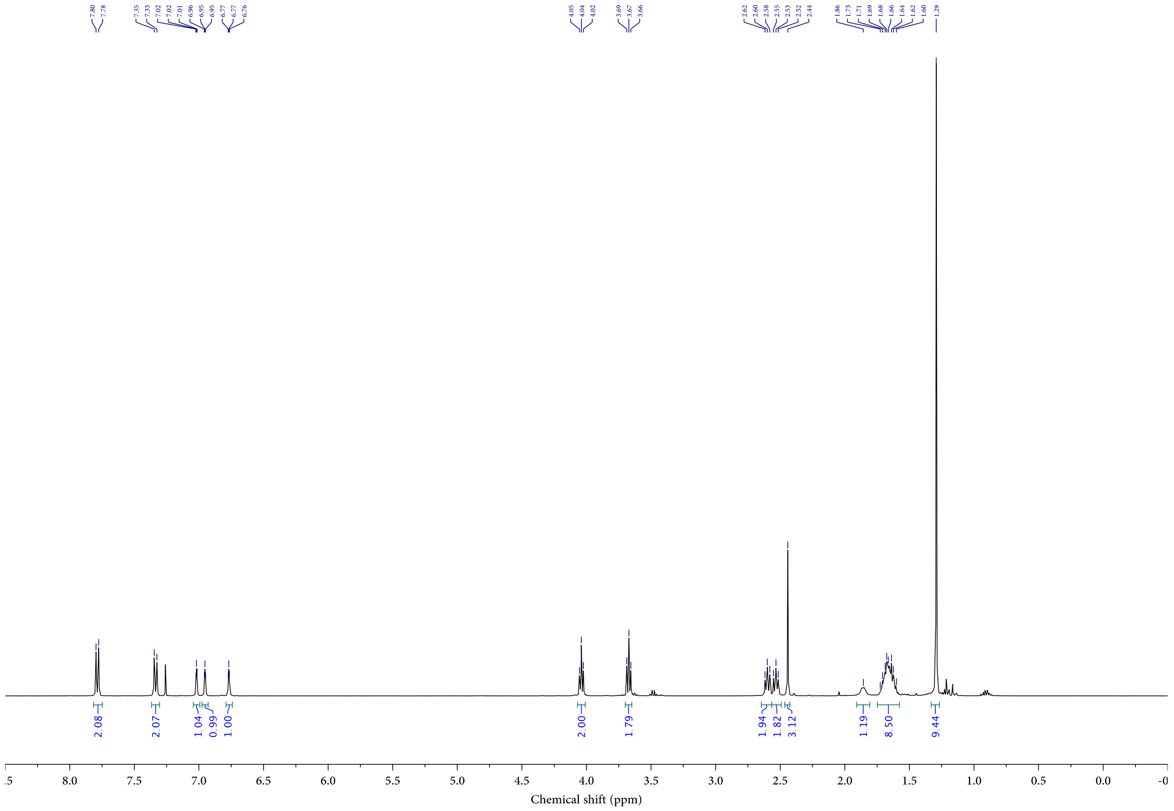

Figure S190. ^{1}H NMR of **S21** ($CDCl_3$, 400 MHz, 298 K)

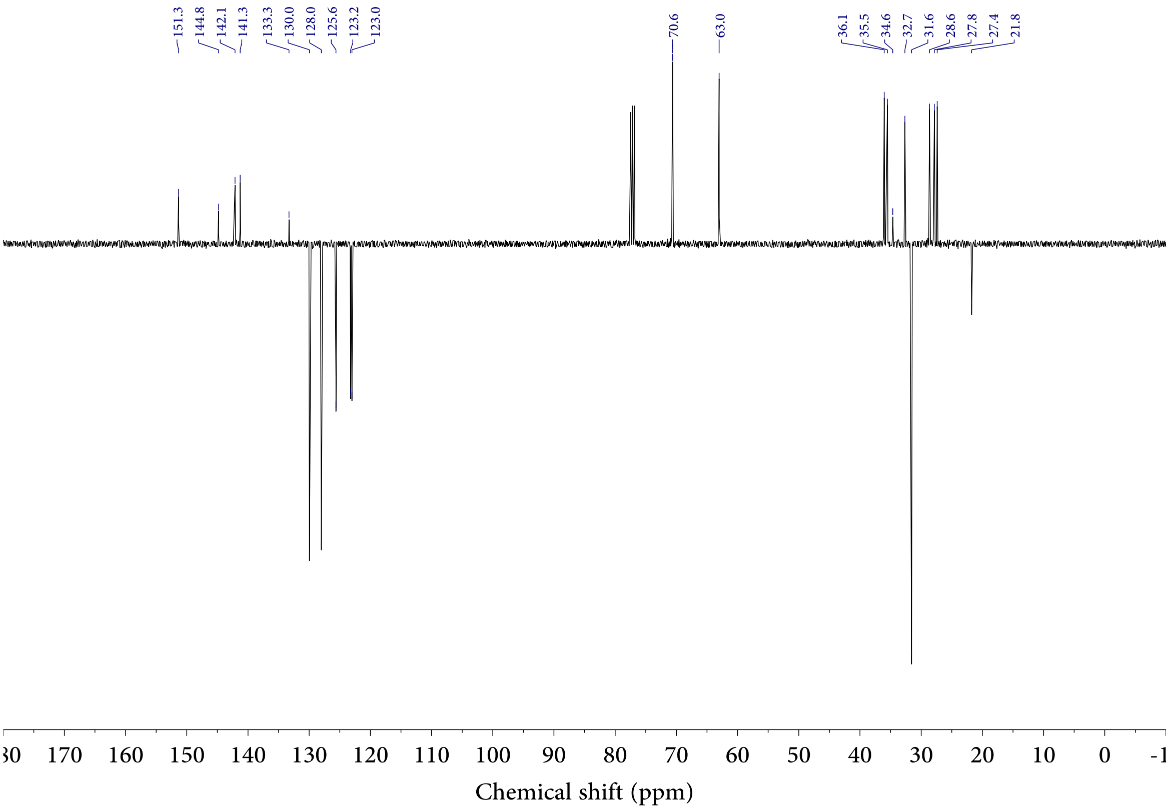

Figure S191. JMOD NMR of **S21** ($CDCl_3$, 101 MHz, 298 K)

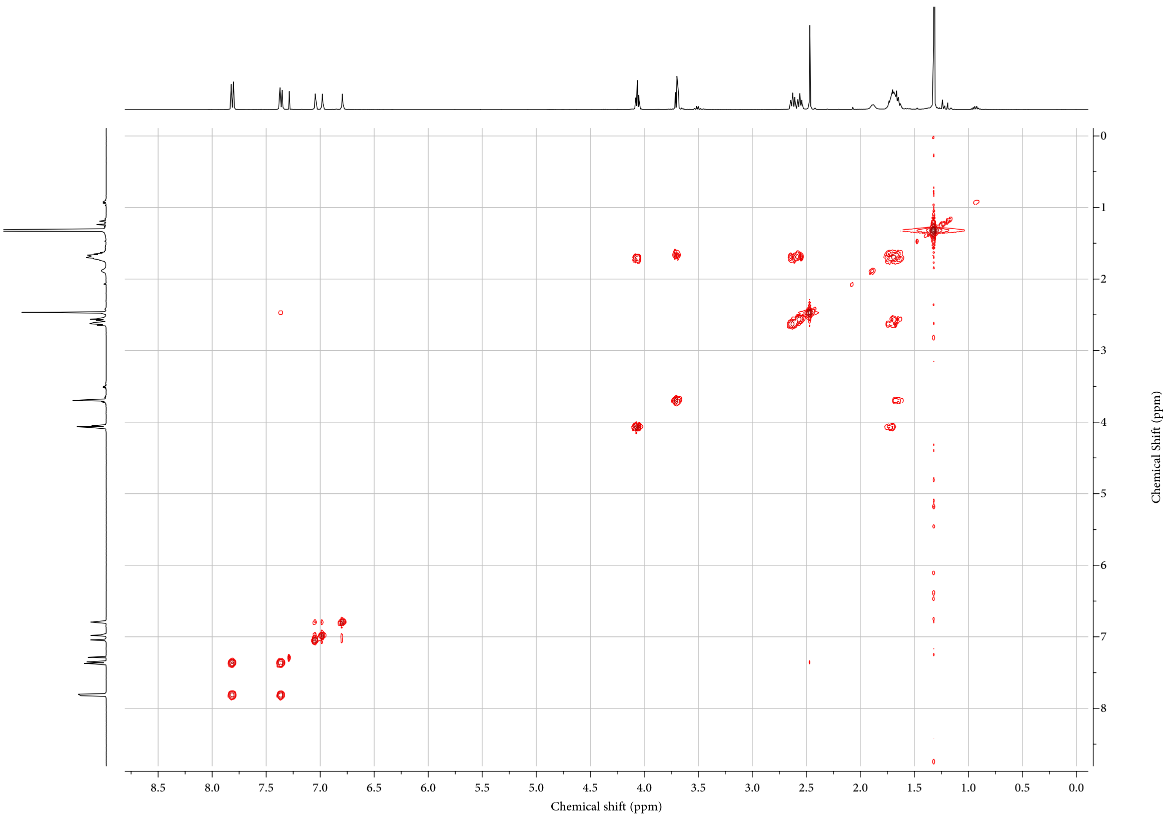

Figure S192. COSY NMR of **S21** ($CDCl_3$, 298 K)

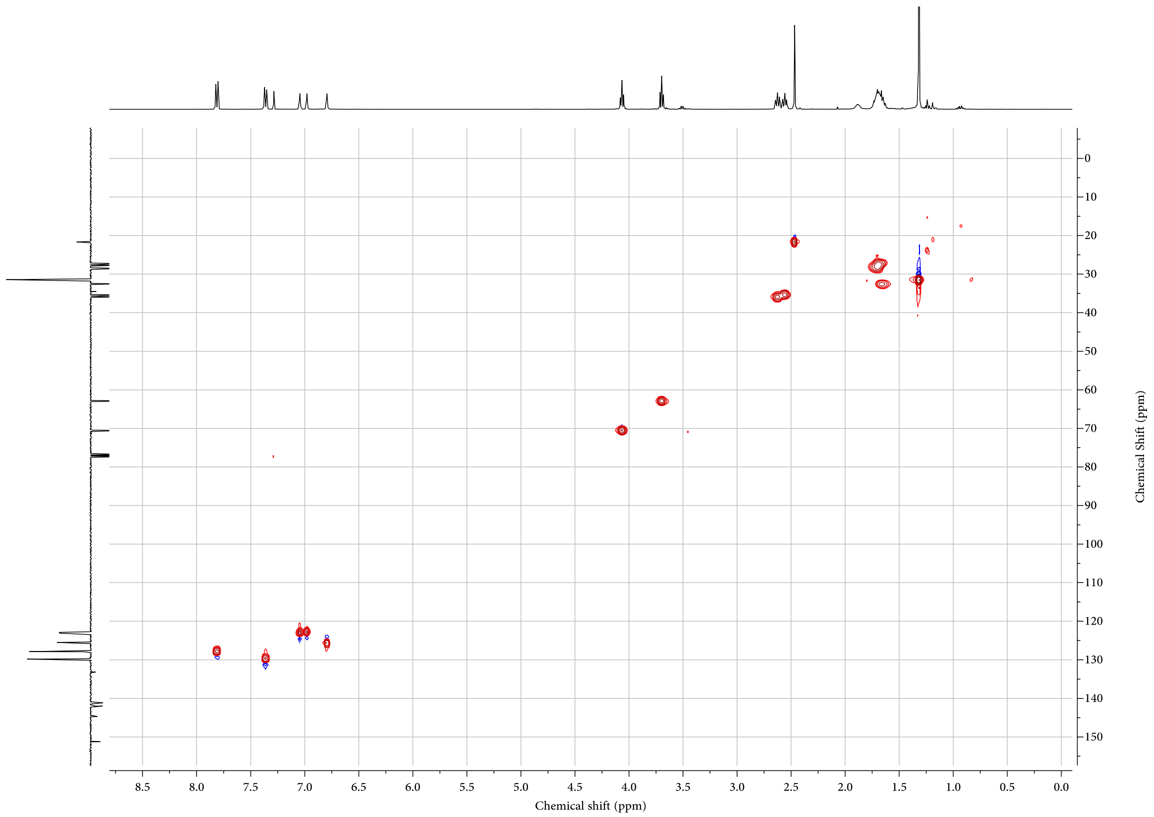

Figure S193. HSQC NMR of **S21** ($CDCl_3$, 298 K)

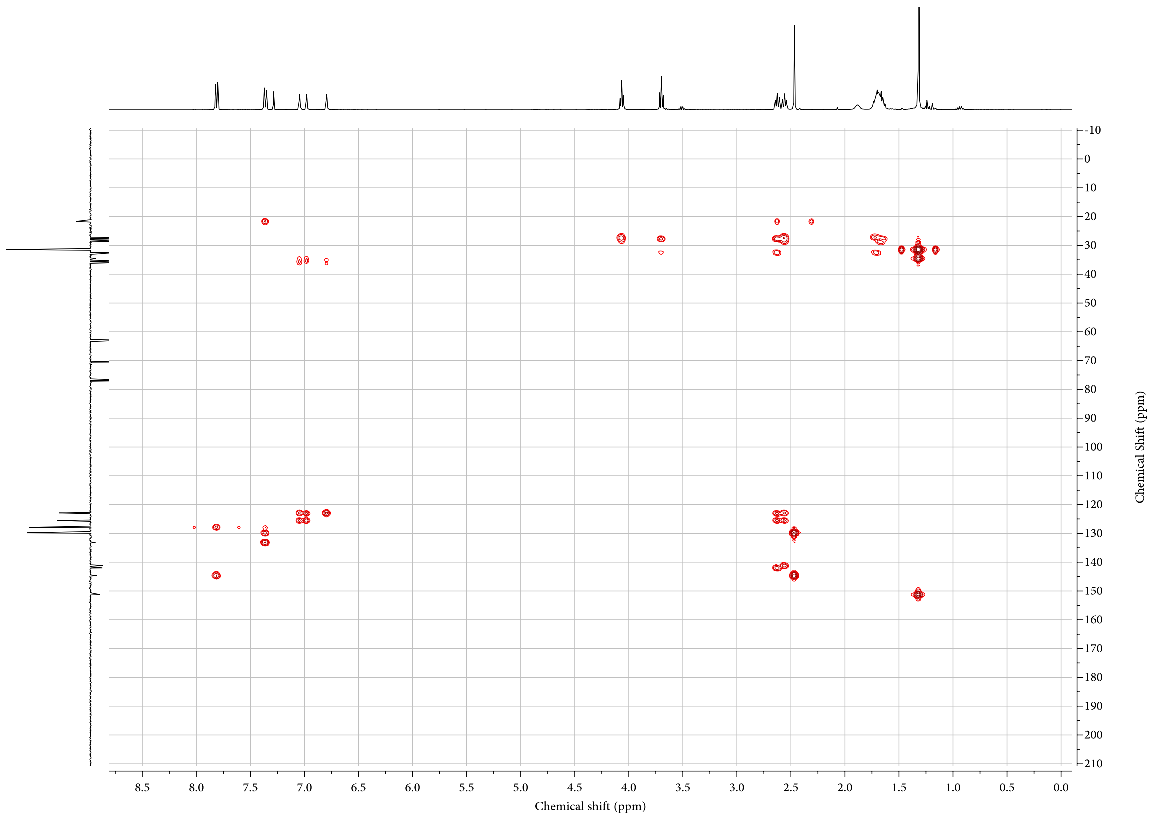

Figure S194. HMBC NMR of **S21** ($CDCl_3$, 298 K)

Aryl ether S22

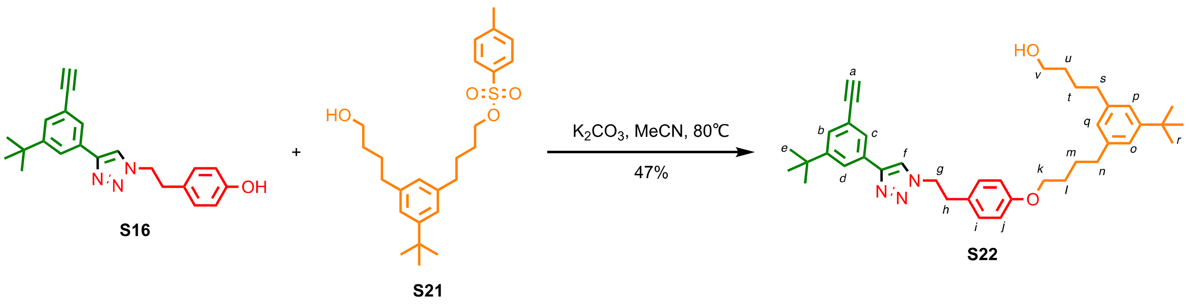

S21 (433 mg, 1.0 mmol, 1.0 equiv.), **S16** (370 mg, 1.08 mmol, 1.0 equiv.) and K_2CO_3 (276 mg, 2.0 mmol, 2.0 equiv.) were suspended in CH_3CN (10 mL) and the mixture heated at reflux for 16 h. The reaction mixture was filtered over a Celite® pad, which was washed with EtOAc (20 mL). The washings were combined, and the solvent was removed *in vacuo*. Column chromatography (petrol-EtOAc 75 : 25 → 70 : 30 → 50 : 50) gave **S22** as a yellow oil (280 mg, 47%).

^{1}H NMR (400 MHz, $CDCl_3$, 298 K) δ: 7.92 (t, *J* = 1.8, 1H, H_d), 7.60 (t, *J* = 1.5, 1H, H_c), 7.53-7.41 (m, 2H, H_b, H_f), 7.09-6.94 (m, 4H, H_i, H_o, H_p), 6.91-6.72 (m, 3H, H_j, H_q), 4.59 (t, *J* = 7.2, 2H, H_g), 3.94 (t, *J* = 6.1, 2H, H_k), 3.67 (t, *J* = 6.3, 2H, H_v), 3.18 (t, *J* = 7.1, 2H, H_h), 3.05 (s, 1H, H_a), 2.73-2.52 (m, 4H, H_n, H_s), 1.89-1.75 (m, 4H, H_l, H_m), 1.73-1.55 (m, 4H, H_t, H_u), 1.35 (s, 9H, H_e), 1.30 (s, 9H, H_r).

^{13}C NMR (101 MHz, $CDCl_3$, 298 K) δ: 158.4, 152.3, 151.3, 147.1, 142.1, 141.9, 130.7, 129.9, 129.1, 128.9, 126.7, 125.7, 123.7, 123.1, 123.1, 122.3, 120.4, 115.0, 84.0, 77.0, 68.0, 63.0, 52.2, 36.1 (×2), 36.0, 35.0, 34.7, 32.7, 31.6, 31.3, 29.2, 28.1, 27.9.

HR-ESI-MS *m/z* = 606.4068 [M+H]$^+$ calc. 606.4054 for $C_{40}H_{52}N_3O_2$.

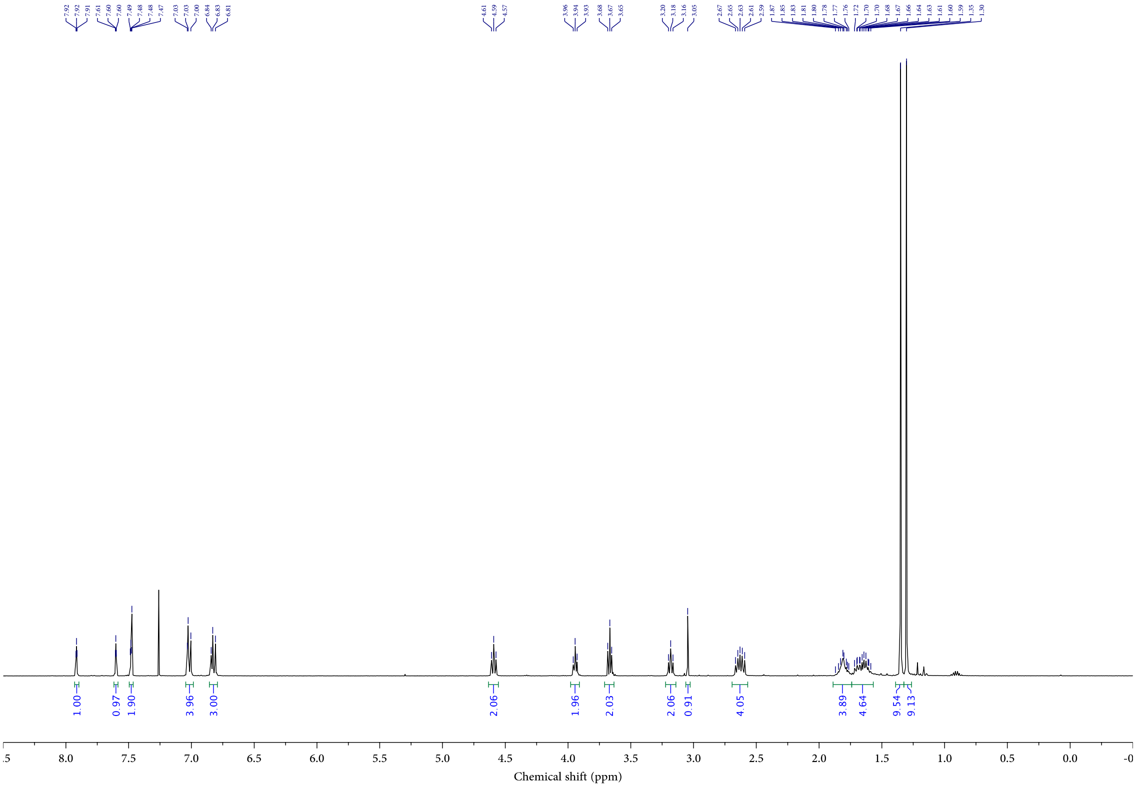

Figure S195. ^{1}H NMR of **S22** ($CDCl_3$, 400 MHz, 298 K)

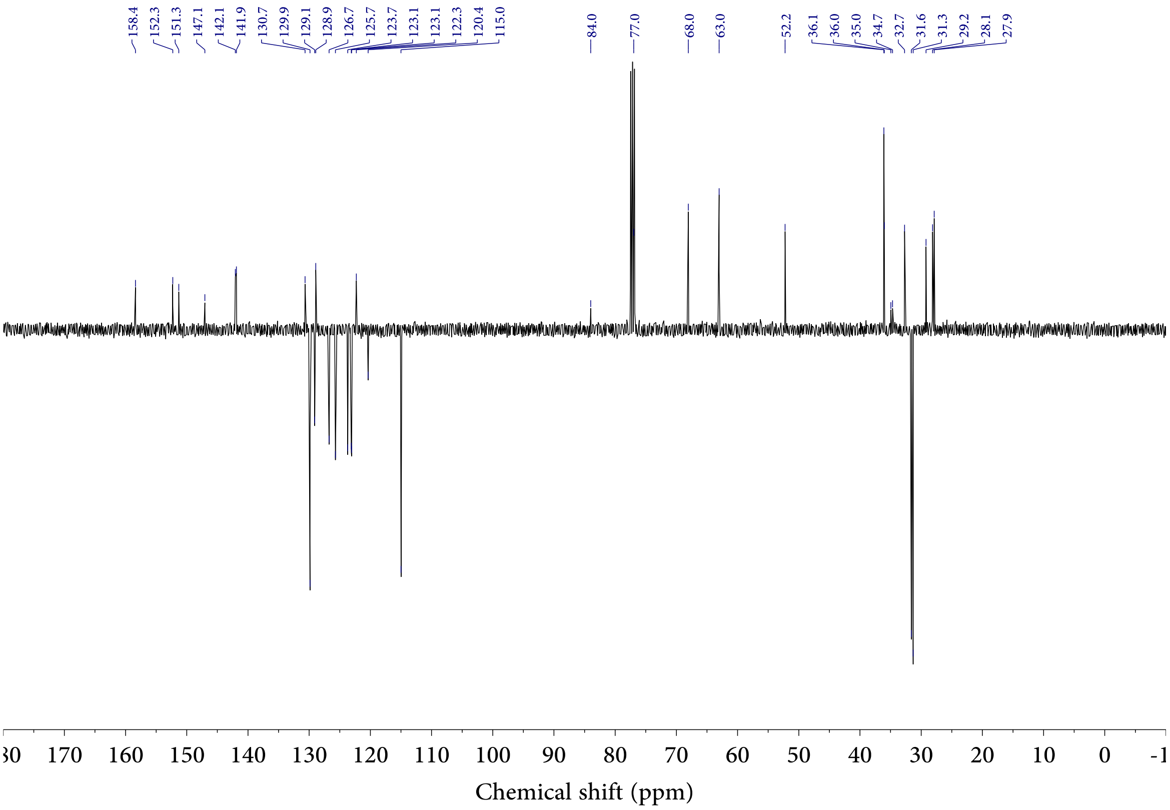

Figure S196. JMOD NMR of **S22** ($CDCl_3$, 101 MHz, 298 K)

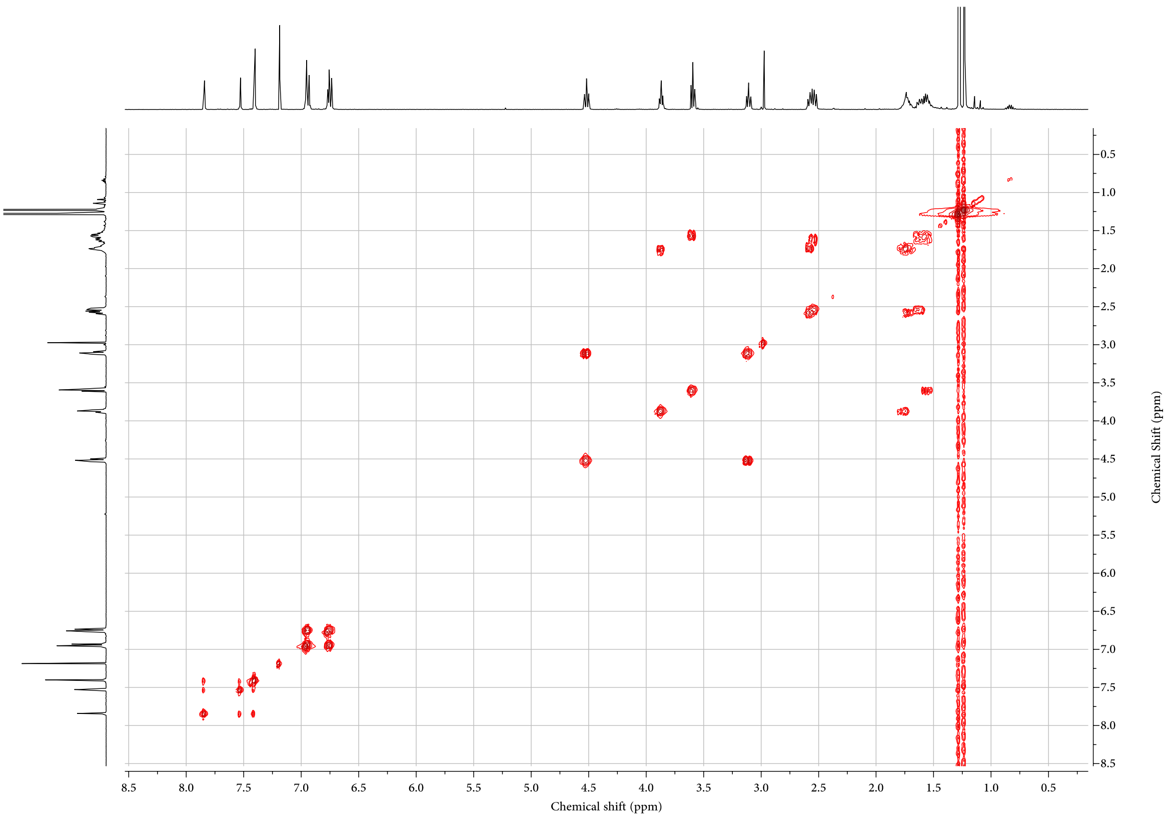

Figure S197. COSY NMR of **S22** ($CDCl_3$, 298 K)

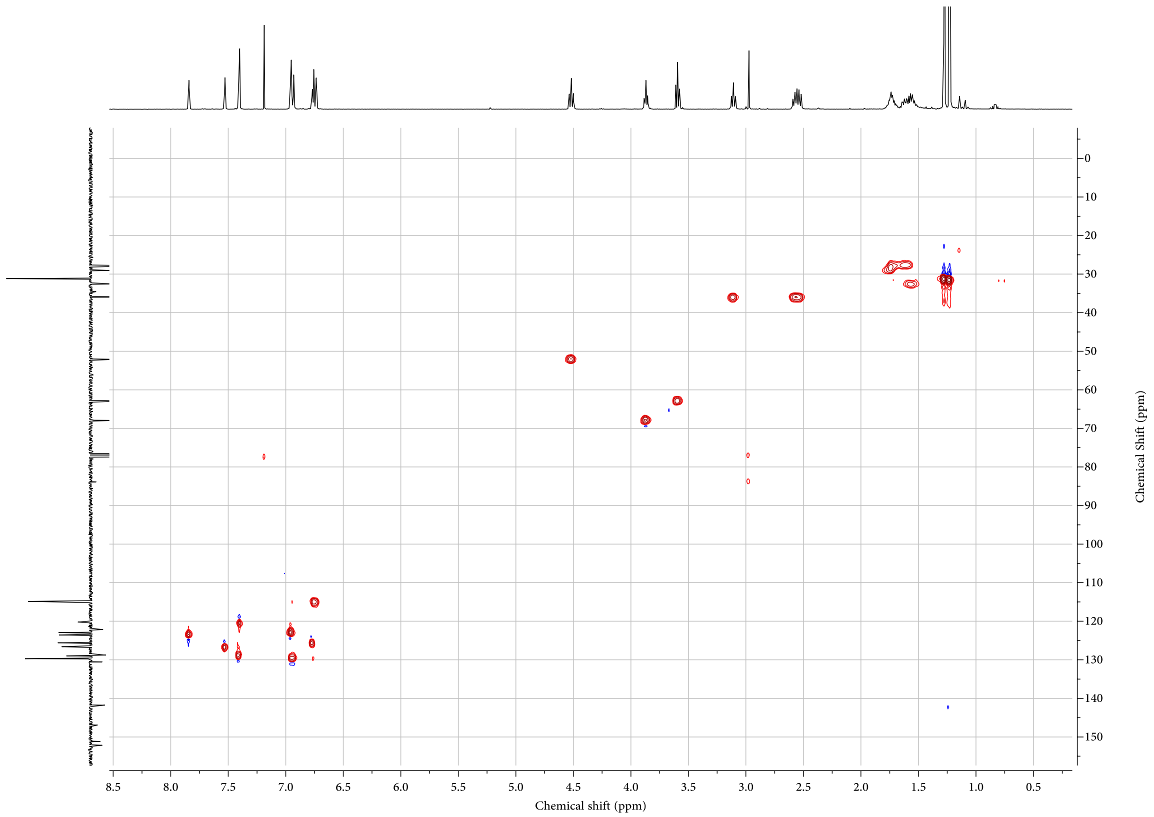

Figure S198. HSQC NMR of **S22** ($CDCl_3$, 298 K)

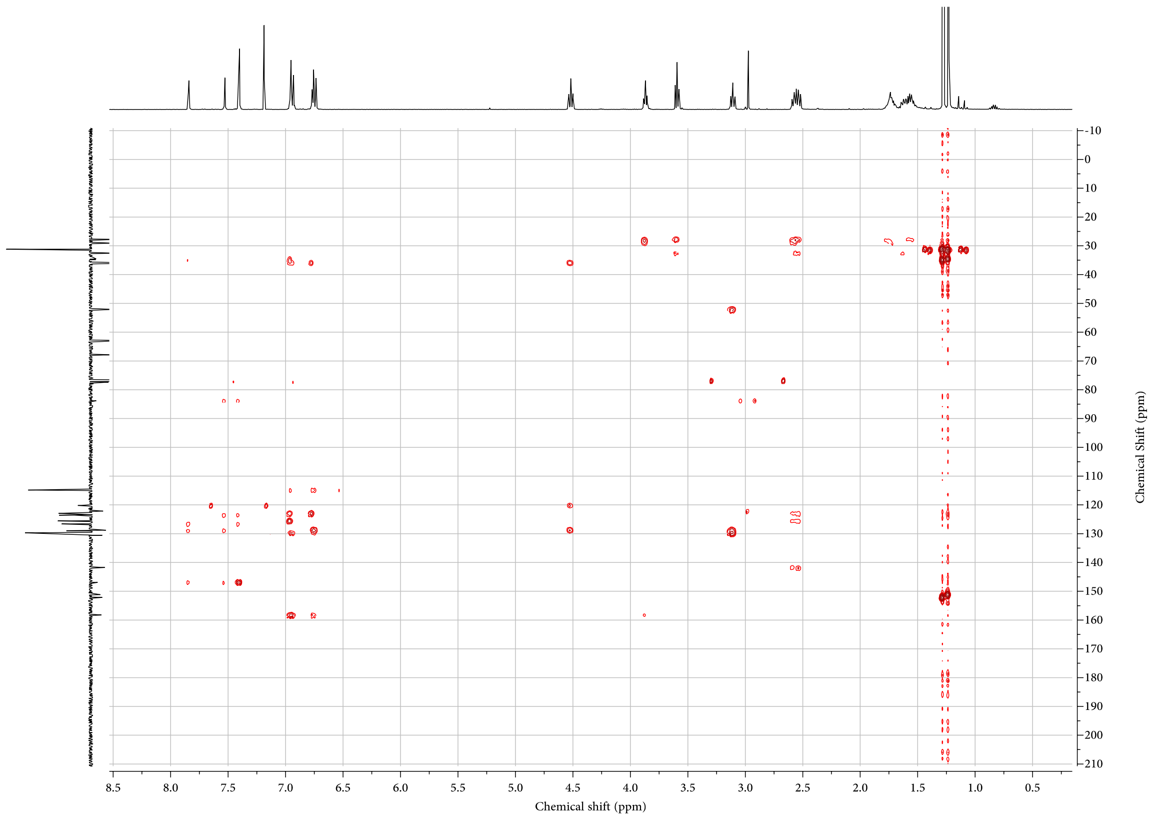

Figure S199. HMBC NMR of **S22** ($CDCl_3$, 298 K)

Tosylate S23

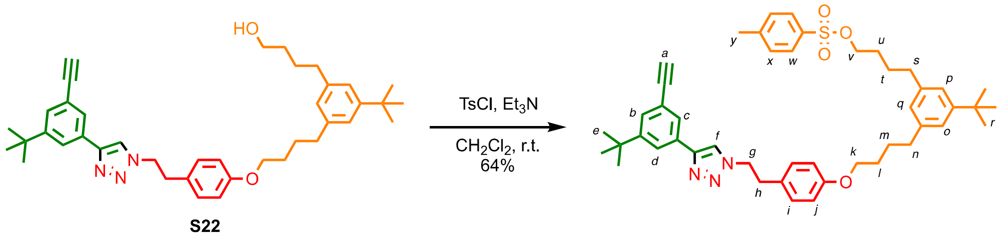

S22 (191 mg, 0.1 mmol, 1.0 equiv.) and triethylamine (140 µL, 1.0 mmol, 3.0 equiv.) were dissolved in CH_2Cl_2 (5 mL). TsCl (190 mg, 1.0 mmol, 3.0 equiv.) was added and the reaction mixture was stirred at rt for 16 h. The solvent was removed *in vacuo*. Column chromatography (petrol-EtOAc 100:0 → 70:30) gave **S23** as a yellow oil (151 mg, 64%).

^{1}H NMR (400 MHz, $CDCl_3$, 298 K) δ: 7.91 (t, *J* = 1.8, 1H, H_d), 7.78 (d, *J* = 8.3, 2H, H_w), 7.60 (t, *J* = 1.5, 1H, H_c), 7.51-7.42 (m, 2H, H_b, H_f), 7.33 (d, *J* = 8.5, 2H, H_x), 7.07-6.99 (m, 3H, H_i, H_o), 6.96 (t, *J* = 1.5, 1H, H_p), 6.82 (d, *J* = 8.7, 2H, H_j), 6.77 (m, 1H, H_q), 4.59 (t, *J* = 7.2, 2H, H_g), 4.04 (t, *J* = 6.1, 2H, H_v), 3.94 (t, *J* = 6.0, 2H, H_k), 3.18 (t, *J* = 7.2, 2H, H_h), 3.05 (s, 1H, H_a), 2.63 (t, *J* = 7.3, 2H, H_n), 2.53 (t, *J* = 7.3, 2H, H_s), 2.43 (s, 3H, H_y), 1.80 (m, 4H, H_l, H_m), 1.75-1.59 (m, 4H, H_t, H_u), 1.35 (s, 9H, H_e), 1.29 (s, 9H, H_r).

^{13}C NMR (101 MHz, $CDCl_3$, 298 K) δ: 158.4, 152.3, 151.4, 147.1, 144.8, 142.0, 141.3, 133.3, 130.8, 130.0, 129.9, 129.1, 129.0, 128.0, 126.7, 125.7, 123.7, 123.2, 123.1, 122.3, 120.3, 115.0, 84.0, 77.0, 70.6, 68.0, 52.2, 36.1, 36.0, 35.5, 35.0, 34.7, 31.6, 31.3, 29.2, 28.7, 28.1, 27.4, 21.8.

HR-ESI-MS *m/z* = 760.4150 $[M+H]^+$ calc. 760.4143 for $C_{47}H_{58}N_3O_4S$.

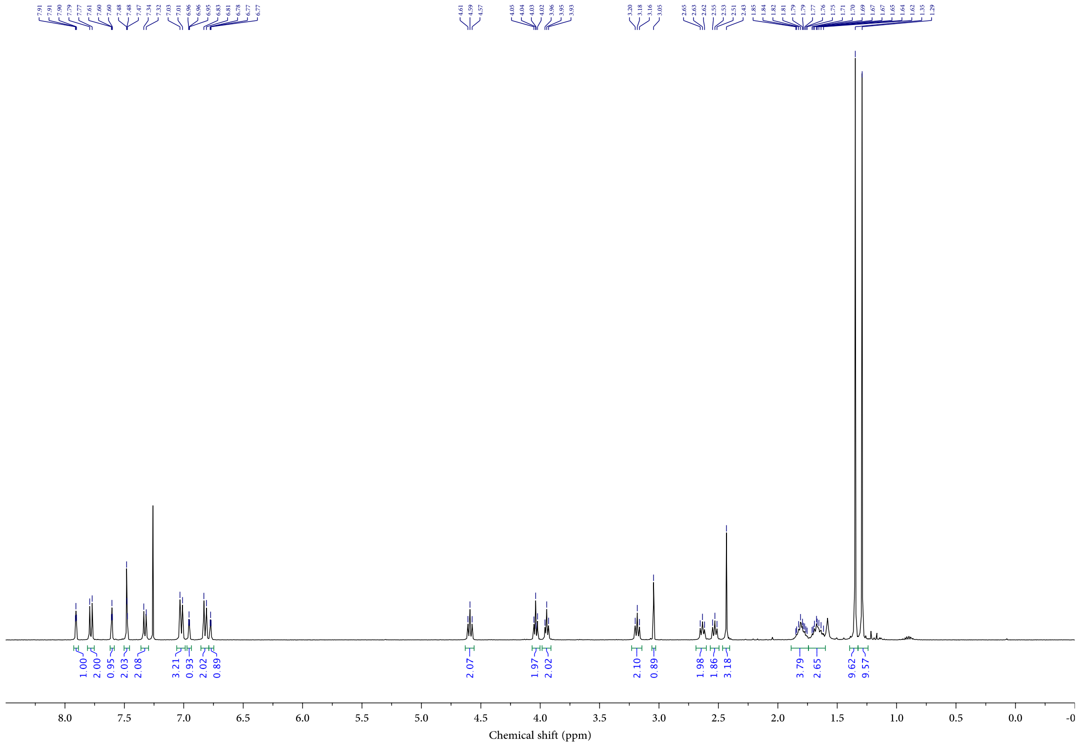

Figure S200. ^{1}H NMR of **S23** ($CDCl_3$, 400 MHz, 298 K)

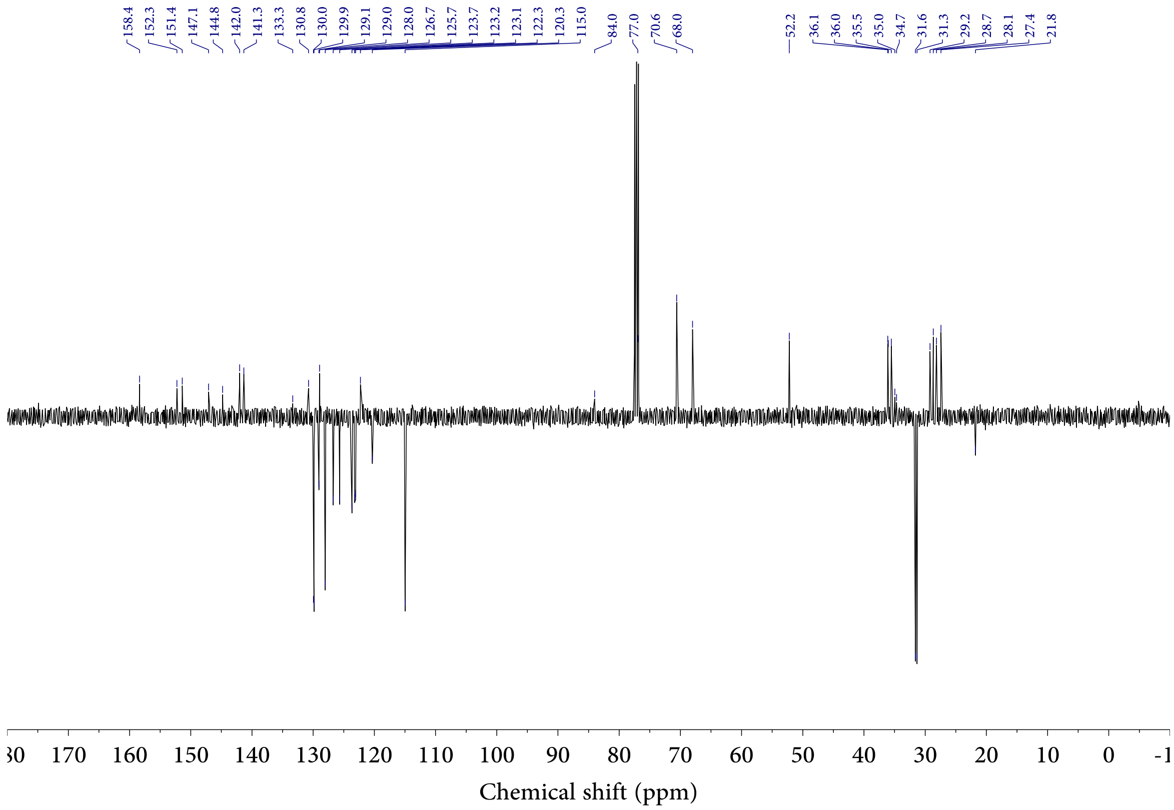

Figure S201. JMOD NMR of **S23** ($CDCl_3$, 101 MHz, 298 K)

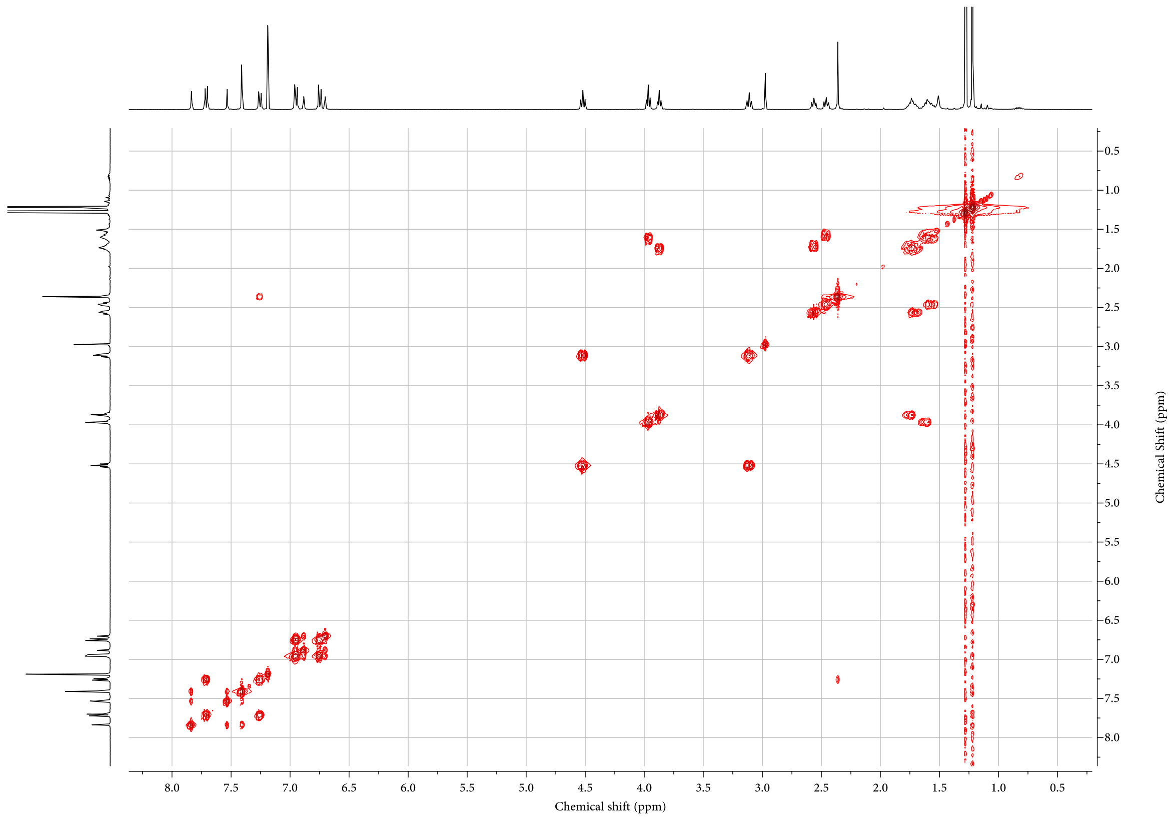

Figure S202. COSY NMR of **S23** ($CDCl_3$, 298 K)

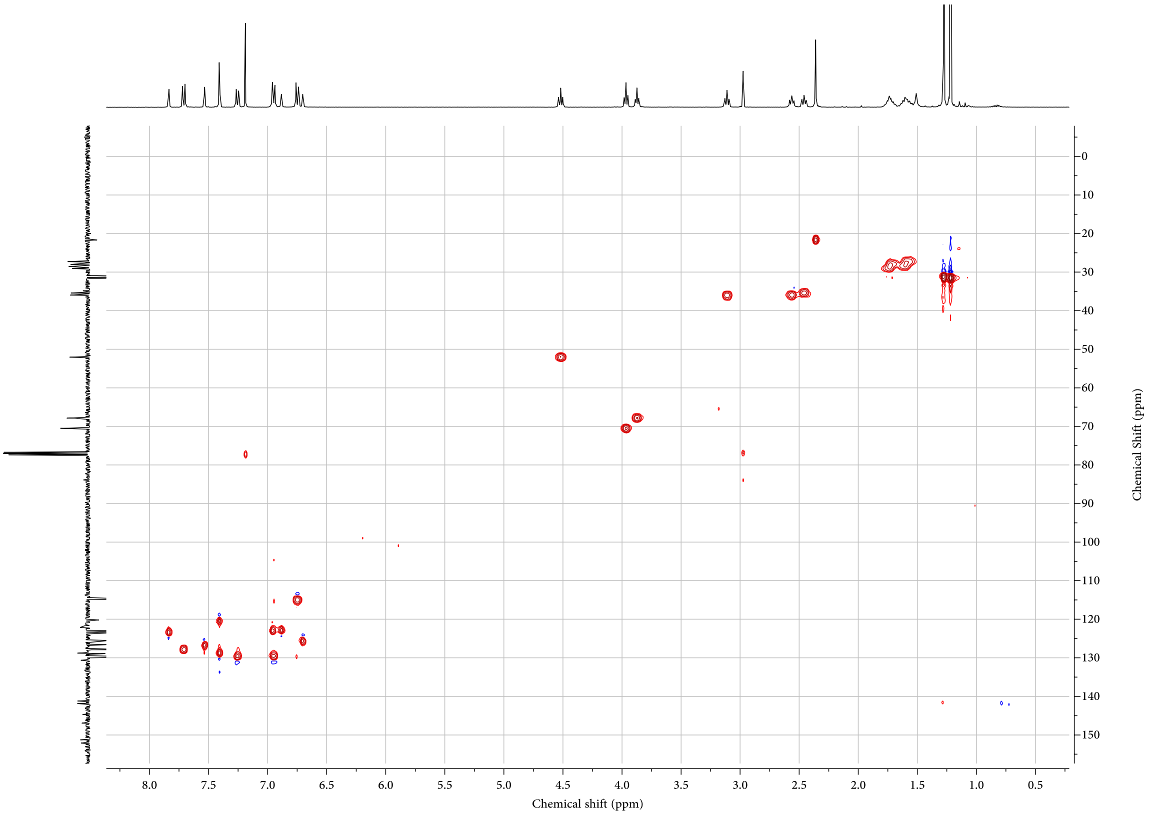

Figure S203. HSQC NMR of **S23** ($CDCl_3$, 298 K)

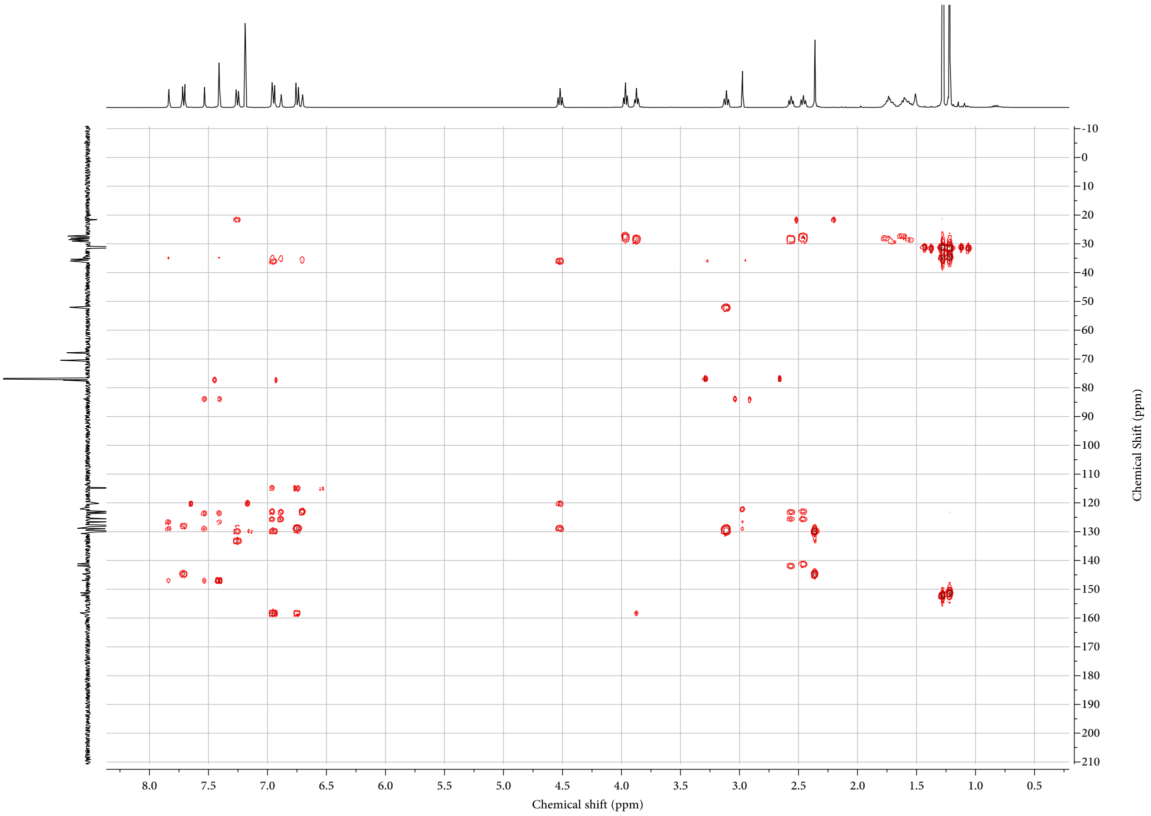

Figure S204. HMBC NMR of **S23** ($CDCl_3$, 298 K)

Boc amine (*S*)-S24

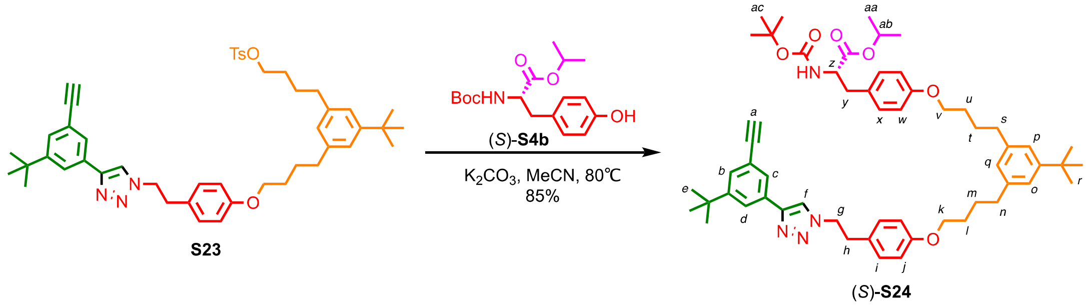

S23 (145 mg, 0.19 mmol, 1.0 equiv.), (*S*)-**S4b** (62 mg, 0.2 mmol, 1.0 equiv.) and K_2CO_3 (105 mg, 0.76 mmol, 4.0 equiv.) were suspended in CH_3CN (2 mL) and heated at reflux for 16 h. The mixture was filtered over a Celite® pad, which was washed with EtOAc (10 mL). The washings were combined, and the solvent removed *in vacuo*. Column chromatography (petrol-EtOAc 100 : 0 → 70 : 30) gave (*S*)-**S24** as a yellow oil (148 mg, 85%).

^{1}H NMR (400 MHz, $CDCl_3$, 298 K) δ: 7.91 (t, *J* = 1.8, 1H, H_d), 7.61 (t, *J* = 1.5, 1H, H_c), 7.53-7.43 (m, 2H, H_b, H_f), 7.10-6.95 (m, 6H, H_i, H_o, H_p, H_x), 6.86 (t, *J* = 1.6, 1H, H_q), 6.84-6.75 (m, 4H, H_j, H_w), 5.07-4.82 (m, 2H, NH, H_{ab}), 4.59 (t, *J* = 7.2, 2H, H_g), 4.52-4.37 (m, 1H, H_z), 3.95 (t, *J* = 5.8, 4H, H_k, H_v), 3.18 (t, *J* = 7.2, 2H, H_h), 3.07-2.83 (m, 3H, H_a, H_y), 2.65 (t, *J* = 7.1, 4H, H_n, H_s), 1.91-1.56 (m, 8H, H_l, H_m, H_t, H_u), 1.42 (s, 9H, H_{ac}), 1.35 (s, 9H, H_e), 1.31 (s, 9H, H_r), 1.22 (d, *J* = 6.2, 3H, H_{aa}), 1.20 (d, *J* = 6.3, 3H, $H_{aa'}$).

^{13}C NMR (101 MHz, $CDCl_3$, 298 K) δ: 171.6, 158.4, 158.3, 155.2, 152.2, 151.3, 147.1, 141.9, 141.9, 130.8, 130.5, 129.8, 129.0, 128.9, 128.1, 126.7, 125.7, 123.6, 123.1, 123.1, 122.3, 120.3, 115.0, 114.6, 84.0, 79.8, 77.0, 69.1, 68.0, 68.0, 54.8, 52.2, 37.6, 36.1, 36.0, 34.9, 34.7, 32.1, 31.6, 31.3, 29.8, 29.5, 29.2, 28.5, 28.2, 21.9, 21.9.

HR-ESI-MS *m/z* = 911.5703 $[M+H]^+$ calc. 911.5681 for $C_{57}H_{75}N_4O_6$.

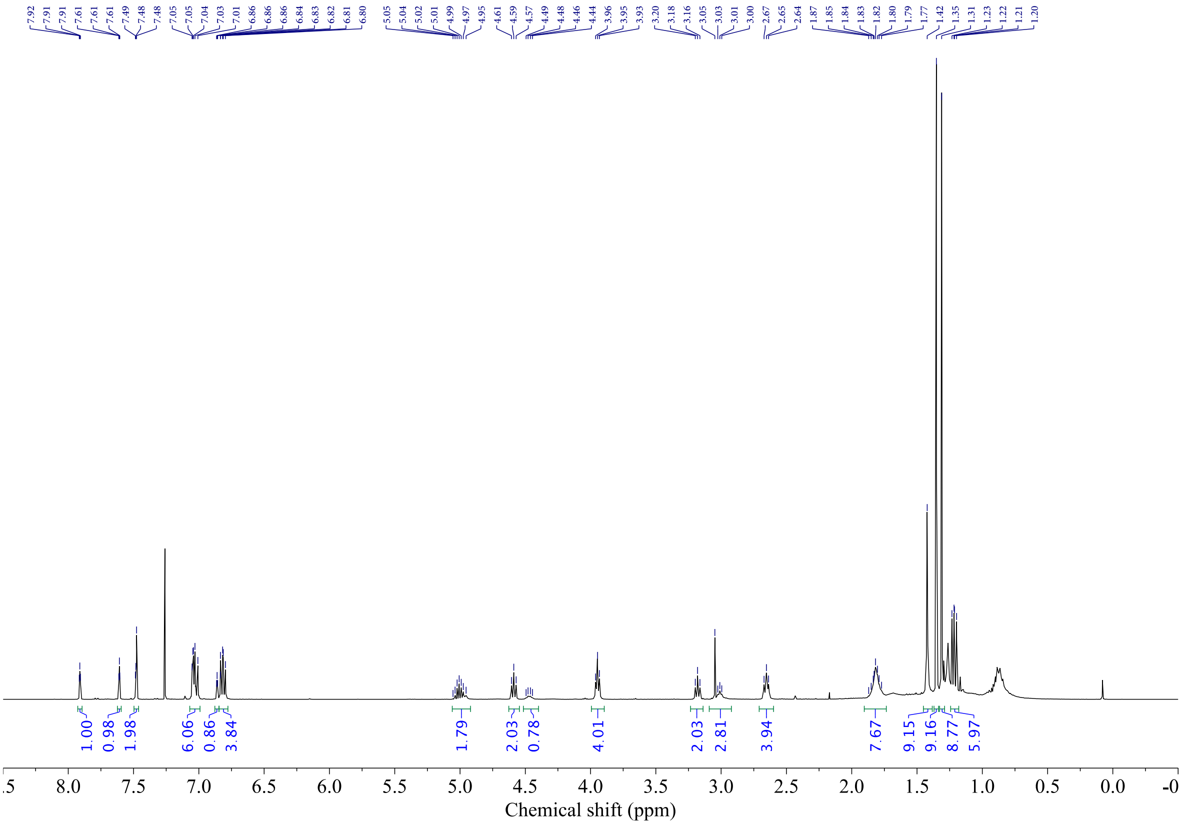

Figure S205. ^{1}H NMR of (*S*)-**S24** ($CDCl_3$, 400 MHz, 298 K)

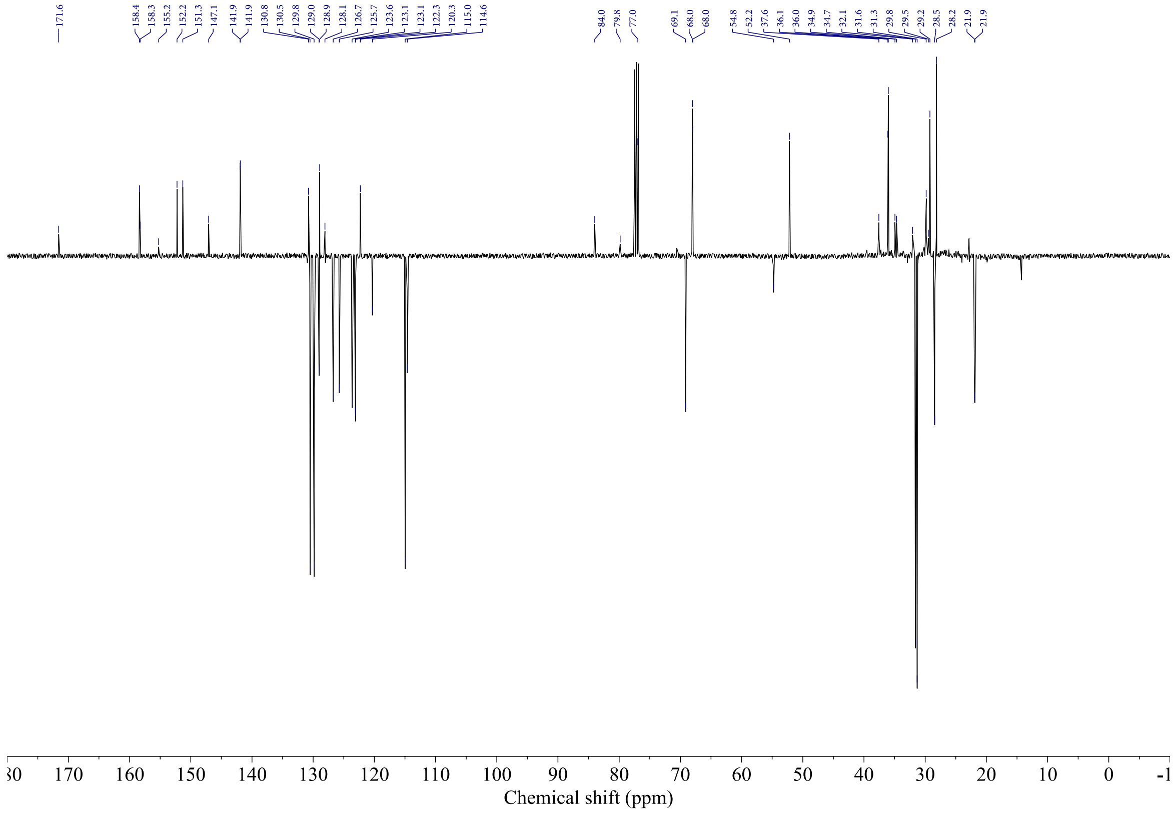

Figure S206. JMOD NMR of (*S*)-**S24** ($CDCl_3$, 101 MHz, 298 K)

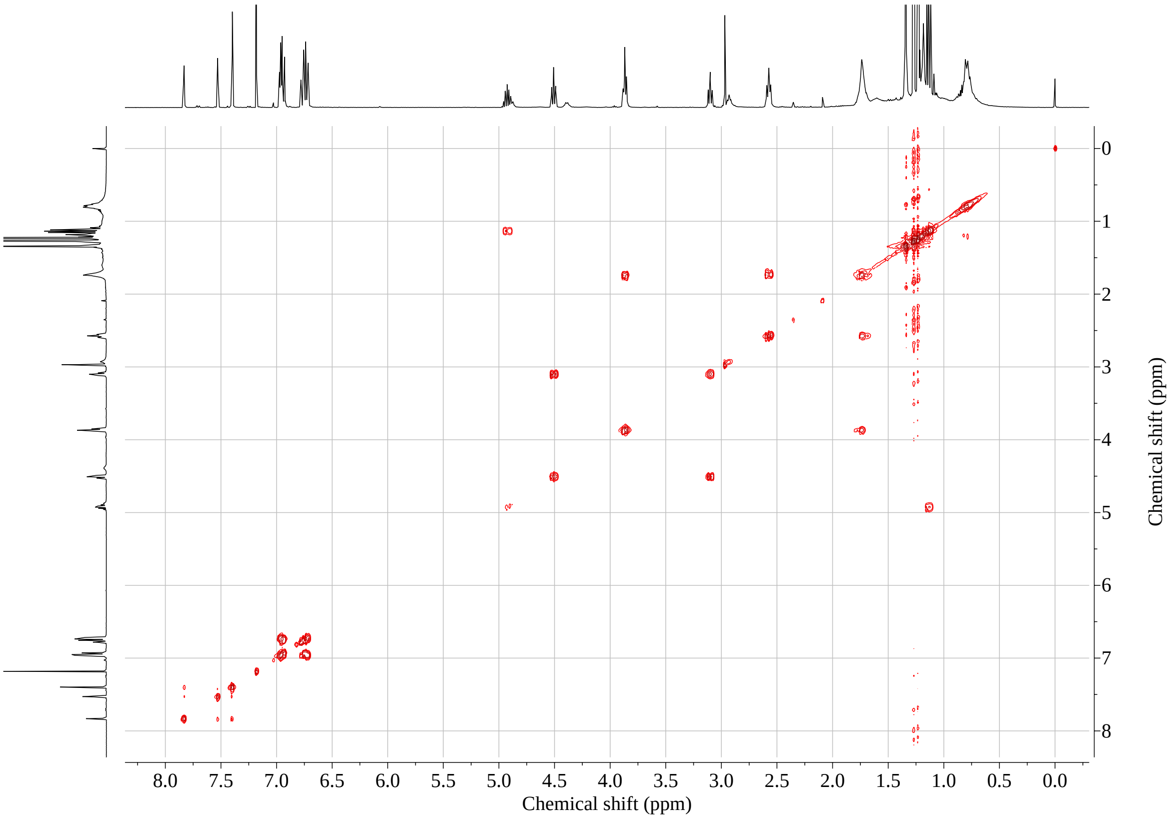

Figure S207. COSY NMR of (*S*)-**S24** ($CDCl_3$, 298 K)

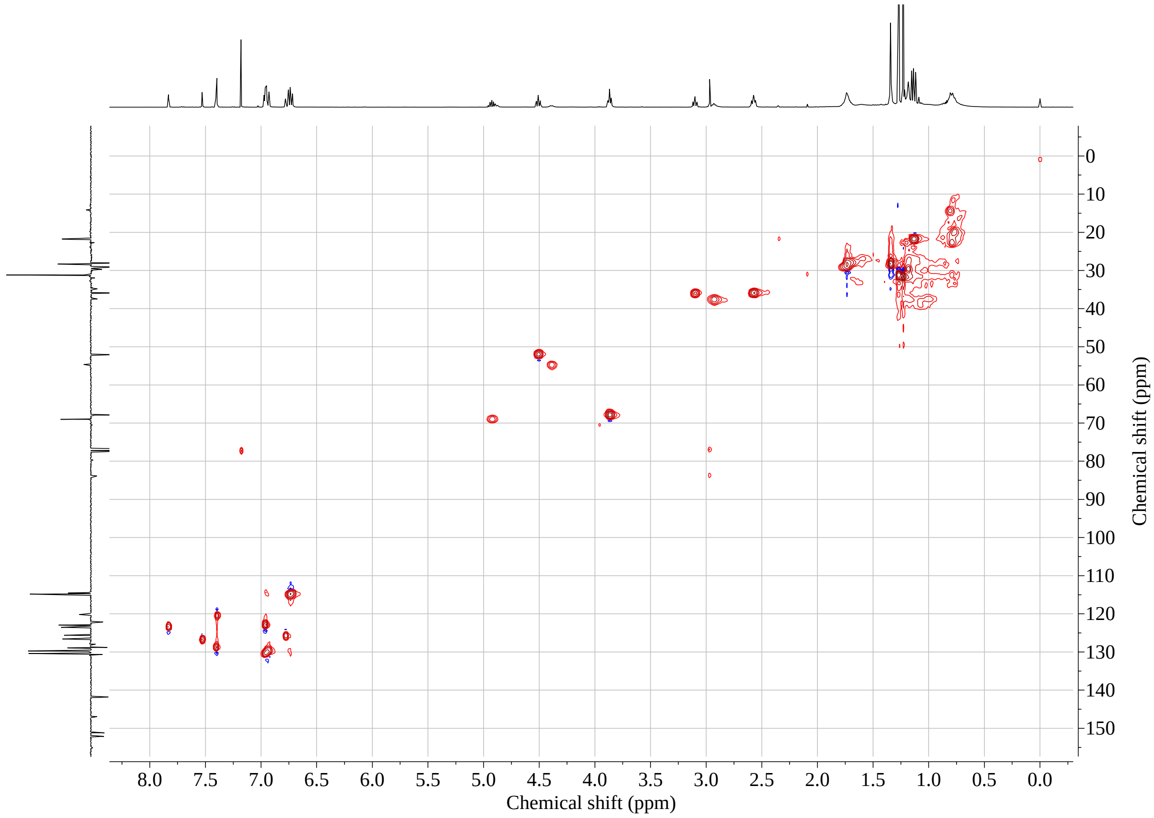

Figure S208. HSQC NMR of (*S*)-**S24** ($CDCl_3$, 298 K)

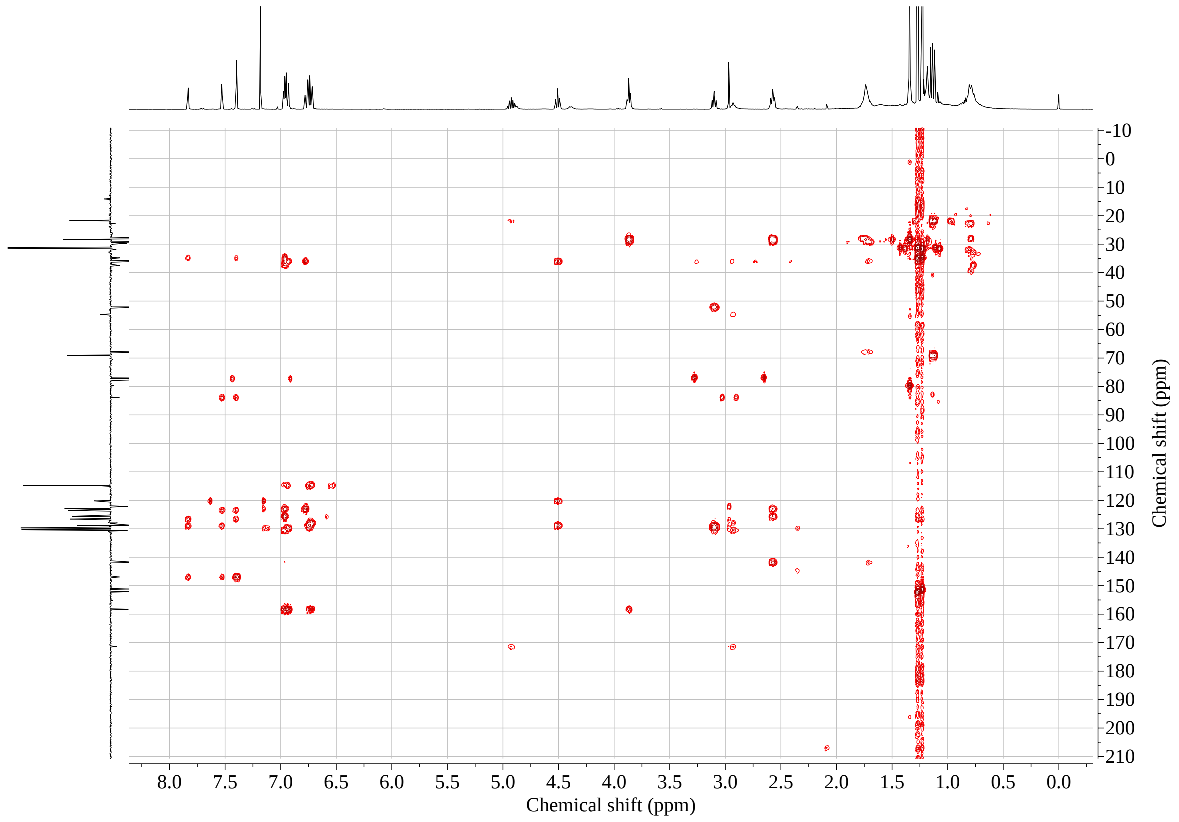

Figure S209. HMBC NMR of (*S*)-**S24** ($CDCl_3$, 298 K)

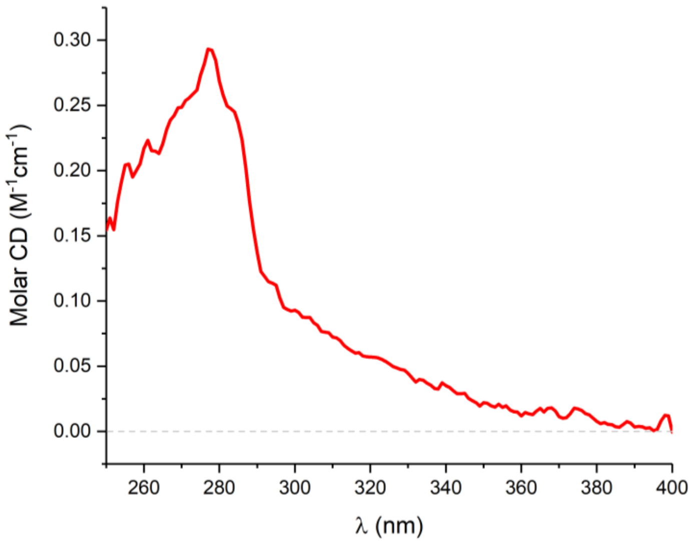

Figure S210. Circular dichroism spectrum of (*S*)-**S24** (79.5 µM) at 293 K in $CHCl_3$

Amine (*S*)-S25

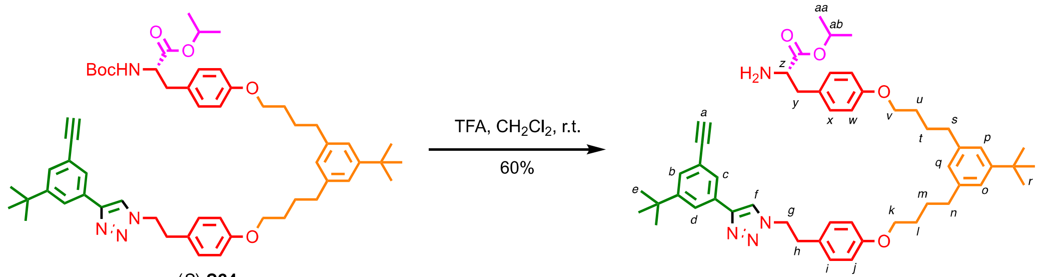

(*S*)-**S24** (*S*)-**S25**

(*S*)-**S24** (148 mg, 0.16 mmol, 1.0 equiv.) was dissolved in CH_2Cl_2 (2 mL). Trifluoroacetic acid (0.12 mL, 1.60 mmol, 10.0 equiv.) was added dropwise and the mixture was stirred at rt for 16 h. Sat. $NaHCO_3$ (10 mL), was added, the mixture was extracted with CH_2Cl_2 (20 mL) and the organic phase dried ($MgSO_4$). The solvent was removed *in vacuo*. Column chromatography (deactivated SiO_2, petrol : EtOAc 100 : 0 → 75 : 25 → 50 : 50 → 25 : 75) gave (*S*)-**S25** as a yellow oil (78 mg, 60%).

^{1}H NMR (400 MHz, $CDCl_3$, 298 K) δ: 7.91 (t, *J* = 1.8, 1H, H_d), 7.61 (t, *J* = 1.6, 1H, H_c), 7.51-7.43 (m, 2H, H_b, H_f), 7.11 (d, *J* = 8.6, 2H, H_x), 7.07-6.96 (m, 4H, H_i, H_o, H_p), 6.89-6.73 (m, 5H, H_j, H_q, H_w), 5.02 (sept, *J* = 6.3, 1H, H_{ab}), 4.59 (t, *J* = 7.2, 2H, H_g), 3.94 (t, *J* = 5.9, 4H, H_k, H_v), 3.70 (t, *J* = 6.6, 1H, H_z), 3.18 (t, *J* = 7.2, 2H, H_h), 3.10-2.97 (m, 2H, H_a, H_y), 2.85 (dd, *J* = 13.8, 7.4, 1H, $H_{y'}$), 2.65 (t, *J* = 7.2, 4H, H_n, H_s), 1.90-1.71 (m, 8H, H_l, H_m, H_t, H_u), 1.35 (s, 9H, H_e), 1.31 (s, 9H, H_r), 1.24 (d, *J* = 6.3, 3H, H_{aa}), 1.21 (d, *J* = 6.3, 3H, $H_{aa'}$).

^{13}C NMR (101 MHz, $CDCl_3$, 298 K) δ: 173.8 (from HMBC), 158.4, 158.3, 152.3, 151.3, 147.1, 142.0, 141.9, 130.8, 130.5, 129.9, 129.0, 128.9, 128.8, 126.7, 125.7, 123.7, 123.1, 123.1, 122.3, 120.3, 115.0, 114.8, 84.0, 77.0, 68.8, 68.0, 68.0, 55.9, 52.2, 39.8, 36.1, 36.0 (×2), 35.0, 34.7, 31.6, 31.3, 29.2 (×2), 28.2 (×2), 21.9, 21.9.

HR-ESI-MS *m/z* = 811.5174 $[M+H]^+$ calc. 811.5157 for $C_{52}H_{67}N_4O_4$.

$[\alpha]_D^{23}$ −0.4 (*c* 0.60, $CHCl_3$)

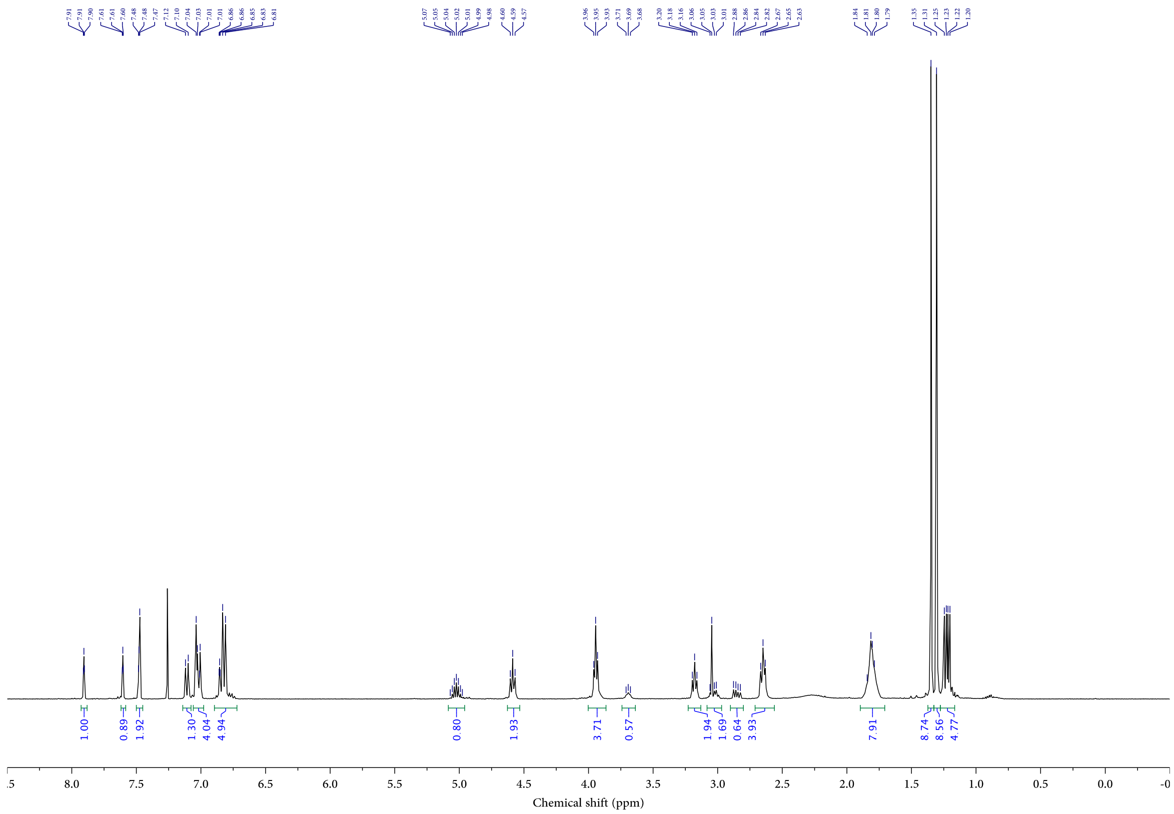

Figure S211. ^{1}H NMR of (*S*)-**S25** ($CDCl_3$, 400 MHz, 298 K)

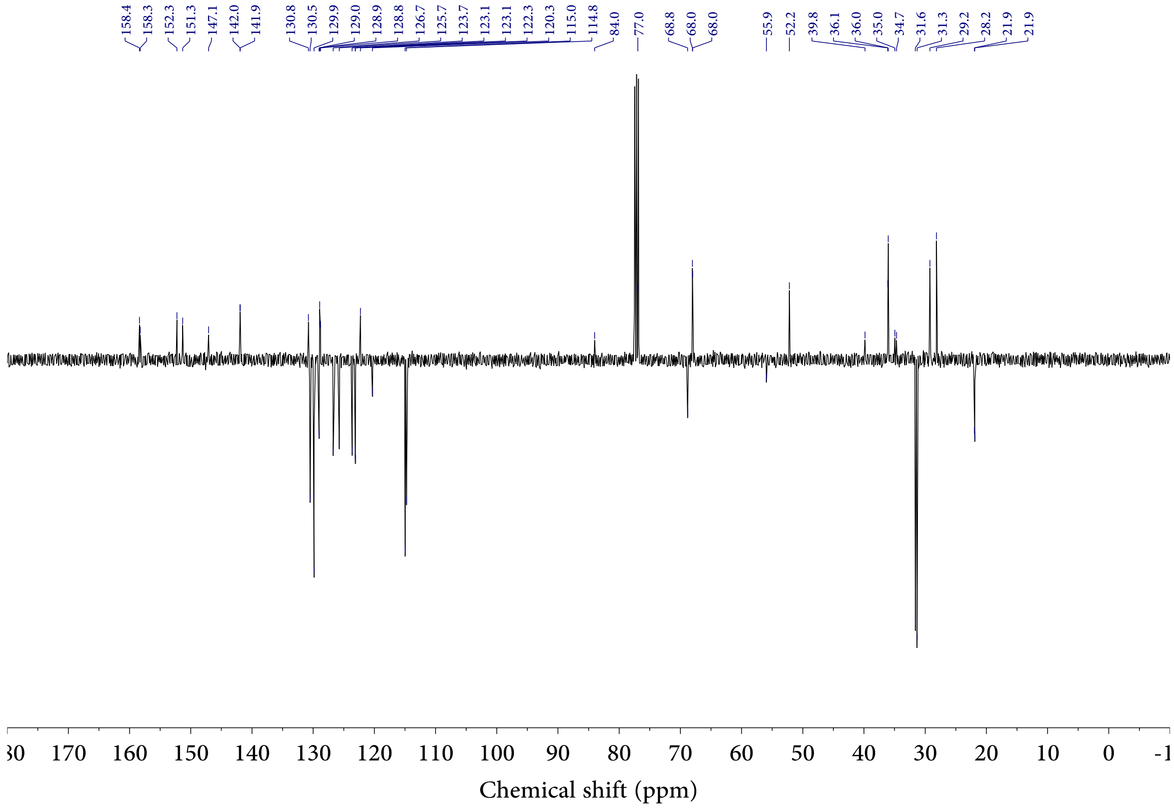

Figure S212. JMOD NMR of (*S*)-**S25** ($CDCl_3$, 101 MHz, 298 K)

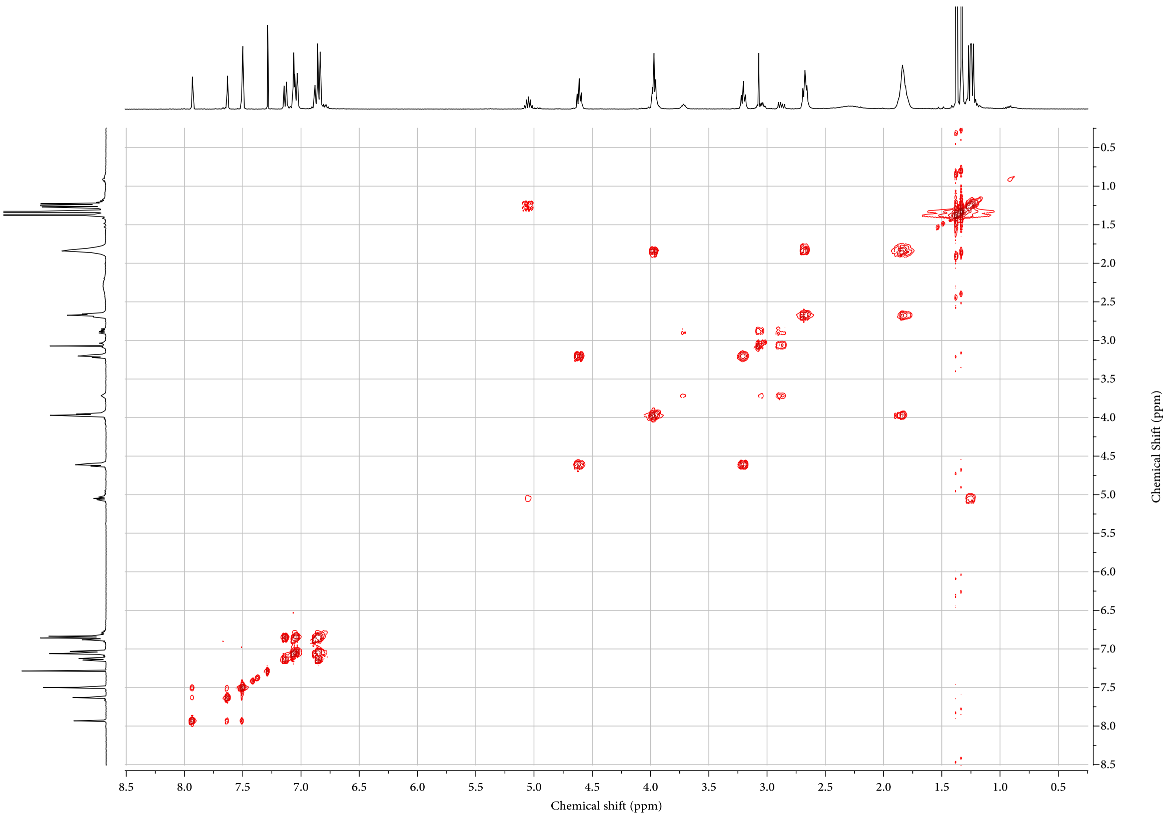

Figure S213. COSY NMR of (*S*)-**S25** ($CDCl_3$, 298 K)

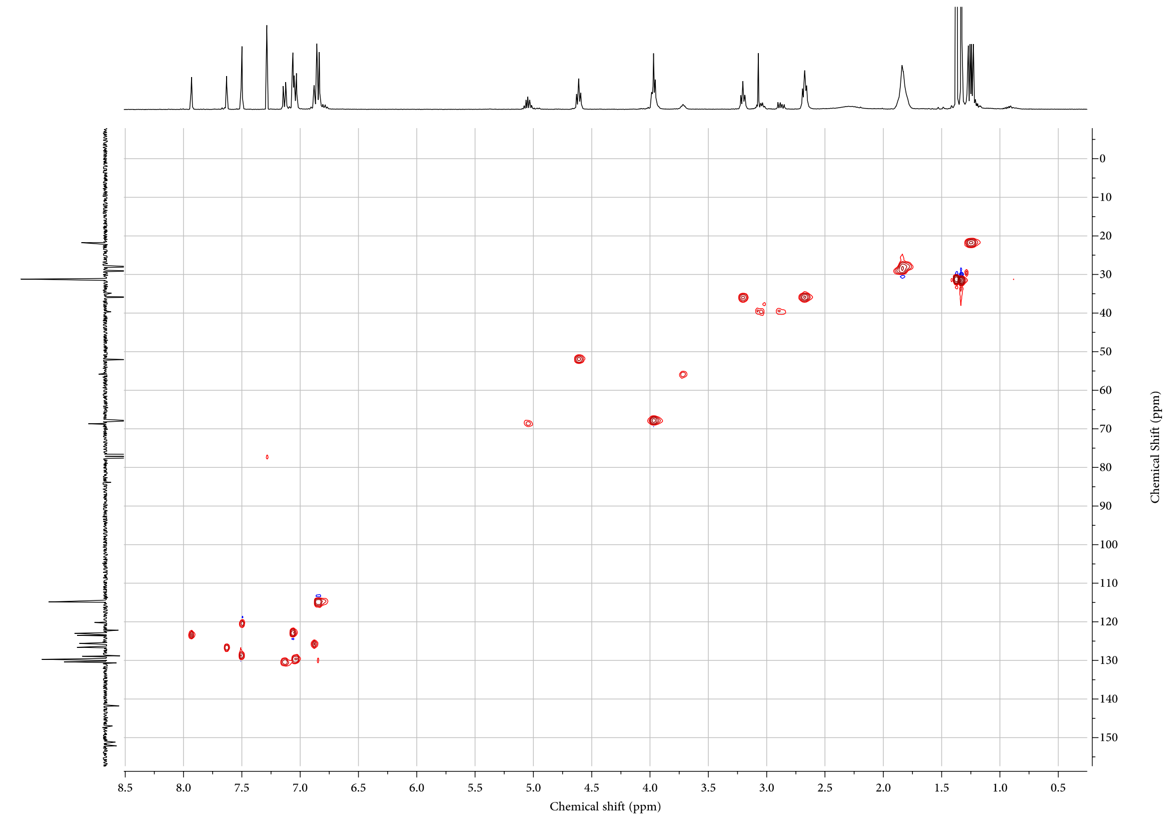

Figure S214. HSQC NMR of (*S*)-**S25** ($CDCl_3$, 298 K)

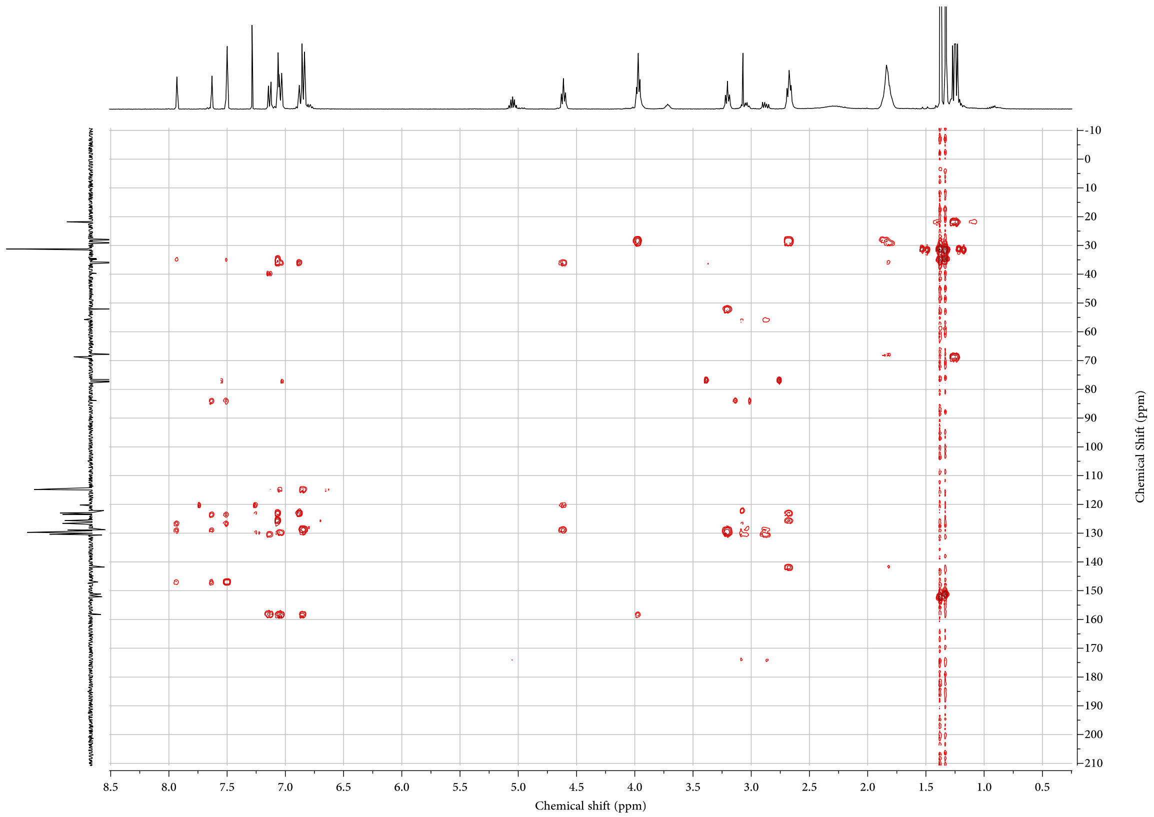

Figure S215. HMBC NMR of (*S*)-**S25** ($CDCl_3$, 298 K)

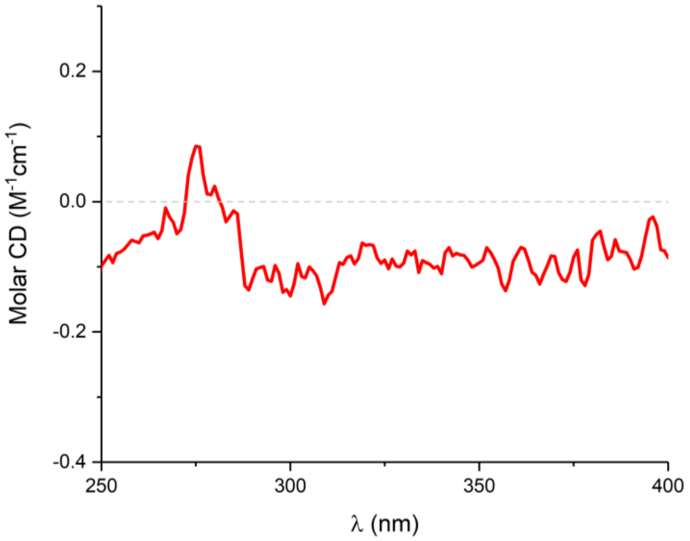

Figure S216. Circular dichroism spectrum of (*S*)-**S25** (41.1 μM) at 293 K in $CHCl_3$

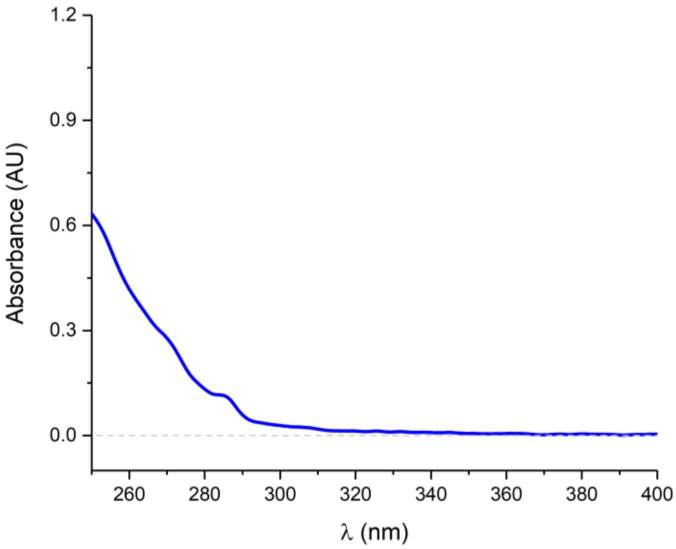

Figure S217. UV-Vis Spectra of (*S*)-**S25** (41.1 µM) at 293 K in $CHCl_3$

Macrocycle precursor (*S*)-7

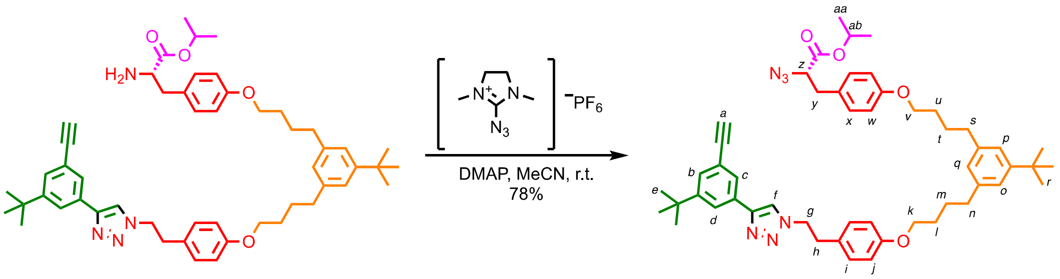

(*S*)-**S25** (51 mg, 0.06 mmol, 1.0 equiv.), DMAP (22 mg, 0.18 mmol, 3.0 equiv.) and ADMP (27 mg, 0.09 mmol, 1.5 equiv.) were dissolved in CH_3CN (1 mL) and the mixture stirred at rt for 16 h. H_2O (5 mL) was added and the mixture extracted CH_2Cl_2 (10 mL). The organic layer was dried ($MgSO_4$) and the the solvent removed *in vacuo*. Column chromatography (petrol-Et_2O 100:0 → 50:50) gave (*S*)-**7** as a colorless oil (39 mg, 78%).

^{1}H NMR (400 MHz, $CDCl_3$, 298 K) δ: 7.92 (t, *J* = 1.8, 1H, H_d), 7.62 (t, *J* = 1.5, 1H, H_c), 7.50-7.46 (m, 2H, H_b, H_f), 7.14 (d, *J* = 8.7, 2H, H_x), 7.07-6.99 (m, 4H, H_i, H_o, H_p), 6.89-6.76 (m, 5H, H_j, H_q, H_w), 5.07 (sept, *J* = 6.3, 1H, H_{ab}), 4.59 (t, *J* = 7.2, 2H, H_g), 3.98-3.92 (m, 5H, H_k, H_v, H_z), 3.18 (t, *J* = 7.2, 2H, H_h), 3.09 (dd, *J* = 14.1, 5.7, 1H, H_y), 3.05 (s, 1H, H_a), 2.95 (dd, *J* = 14.1, 8.5, 1H, $H_{y'}$), 2.66 (t, *J* = 7.2, 4H, H_n, H_s), 1.90-1.68 (m, 8H, H_l, H_m, H_t, H_u), 1.36 (s, 9H, H_e), 1.32 (s, 9H, H_r), 1.27 (d, *J* = 6.3, 3H, H_{aa}), 1.23 (d, *J* = 6.2, 3H, $H_{aa'}$).

^{13}C NMR (101 MHz, $CDCl_3$, 298 K) δ: 169.7, 158.4, 158.4, 152.2, 151.3, 147.1, 141.9, 130.8, 130.4, 129.8, 129.0, 128.9, 127.9, 126.7, 125.7, 123.6, 123.1 (×2), 122.3, 120.3, 115.0, 114.8, 84.0, 77.0, 69.9, 68.0, 68.0, 63.6, 52.2, 36.9, 36.1, 36.0, 36.0 (×2), 34.9, 34.7, 31.6, 31.3, 29.2 (×2), 28.1 (×2), 21.9, 21.8.

HR-ESI-MS *m/z* = 837.5070 $[M+H]^+$ calc. 837.5062 for $C_{52}H_{65}N_6O_4$.

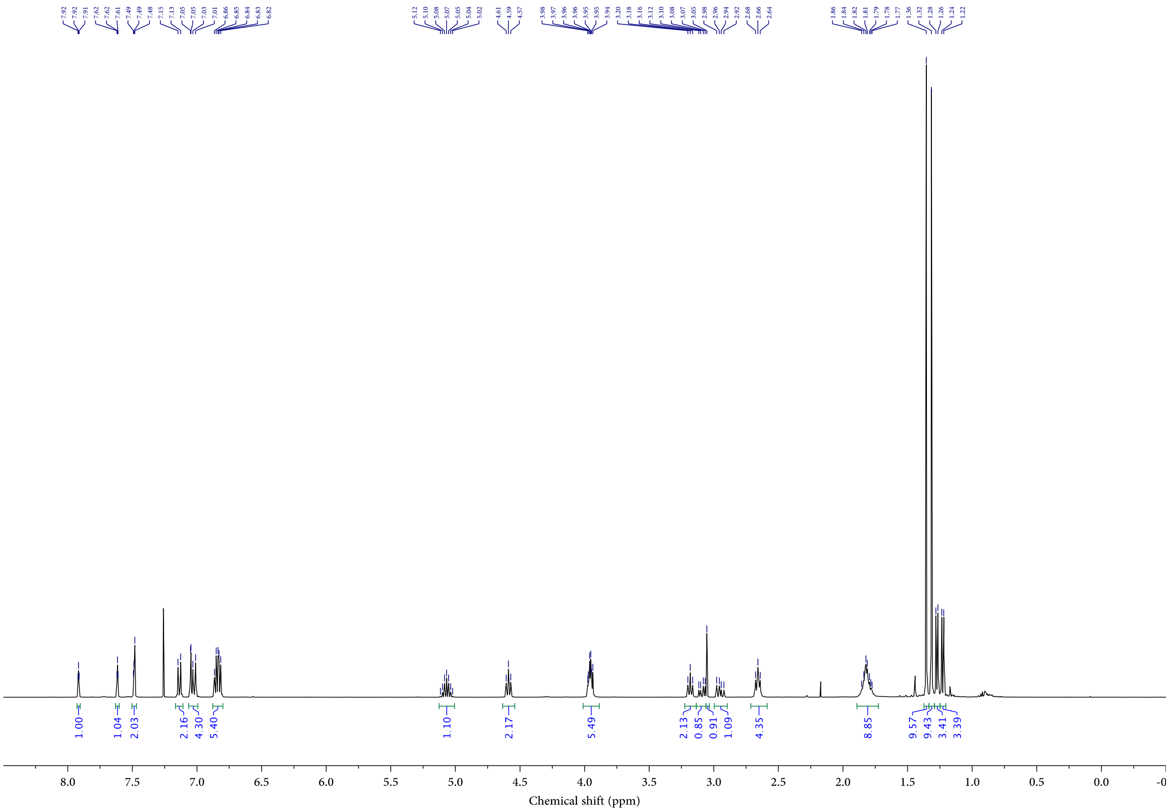

Figure S218. ^{1}H NMR of (*S*)-**7** ($CDCl_3$, 400 MHz, 298 K)

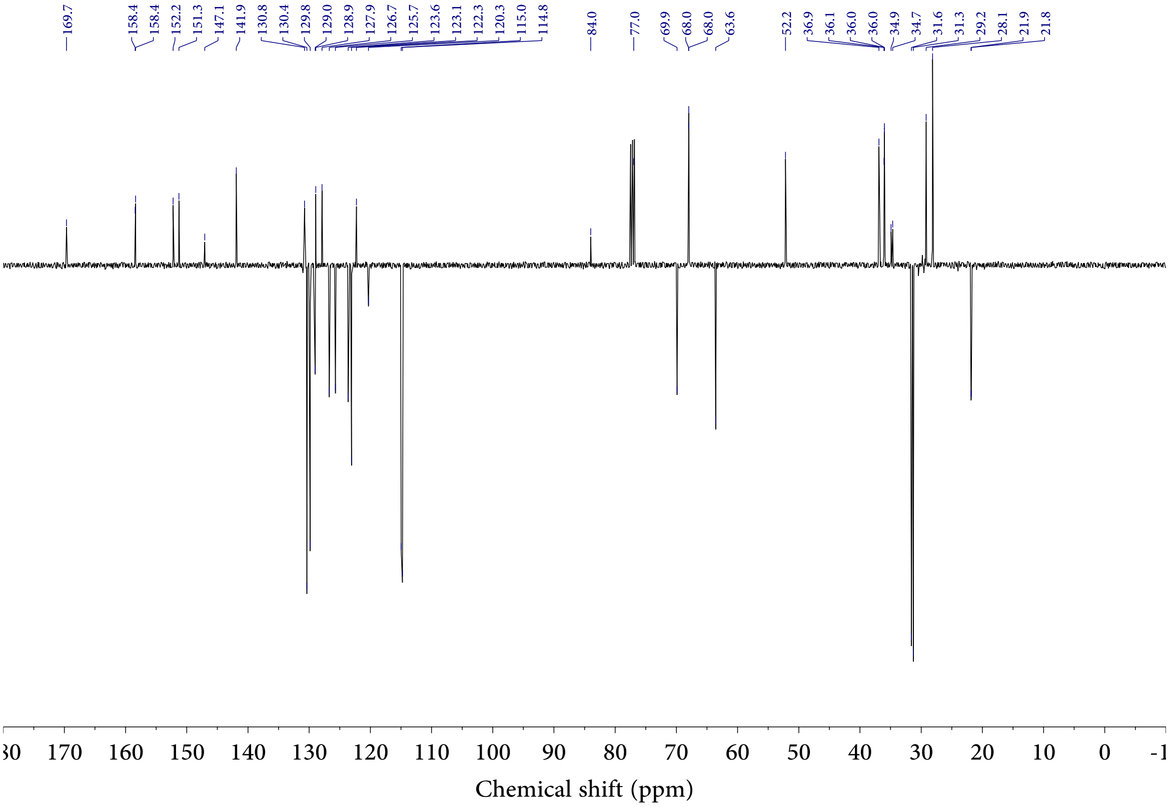

Figure S219. JMOD NMR of (*S*)-**7** ($CDCl_3$, 101 MHz, 298 K)

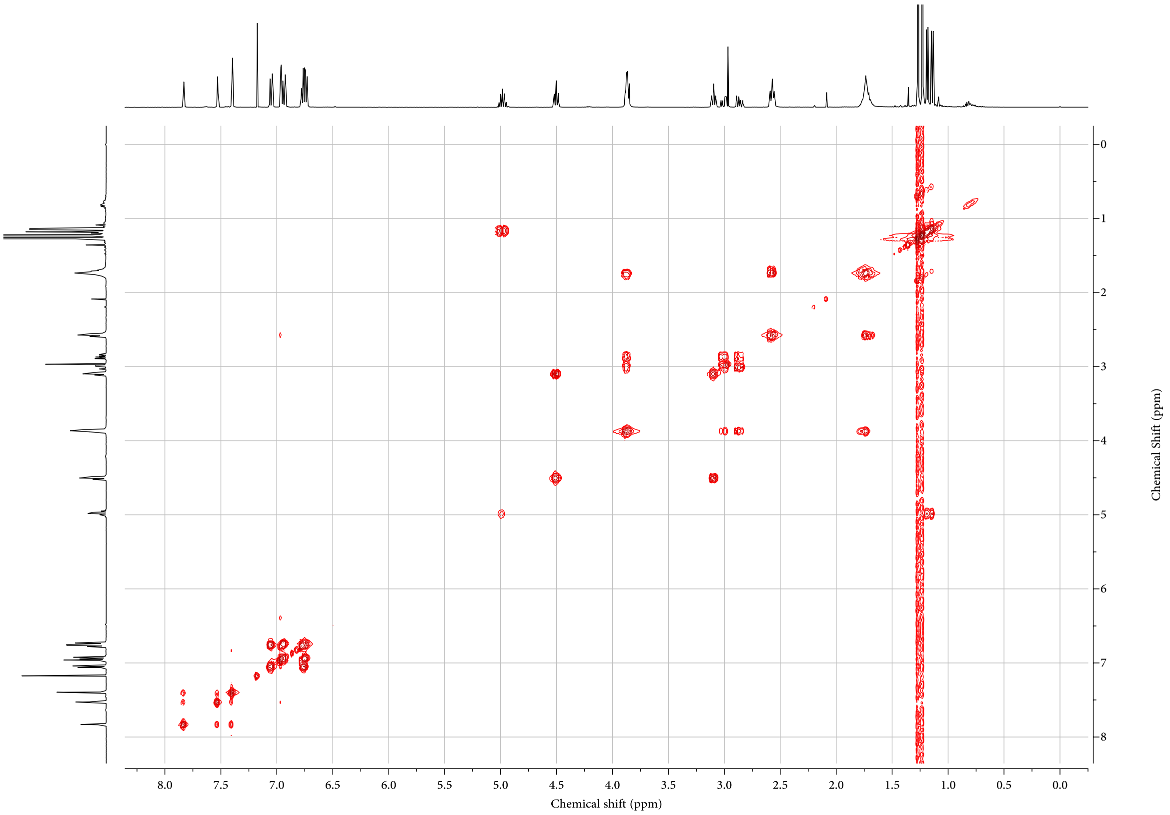

Figure S220. COSY NMR of (*S*)-**7** ($CDCl_3$, 298 K)

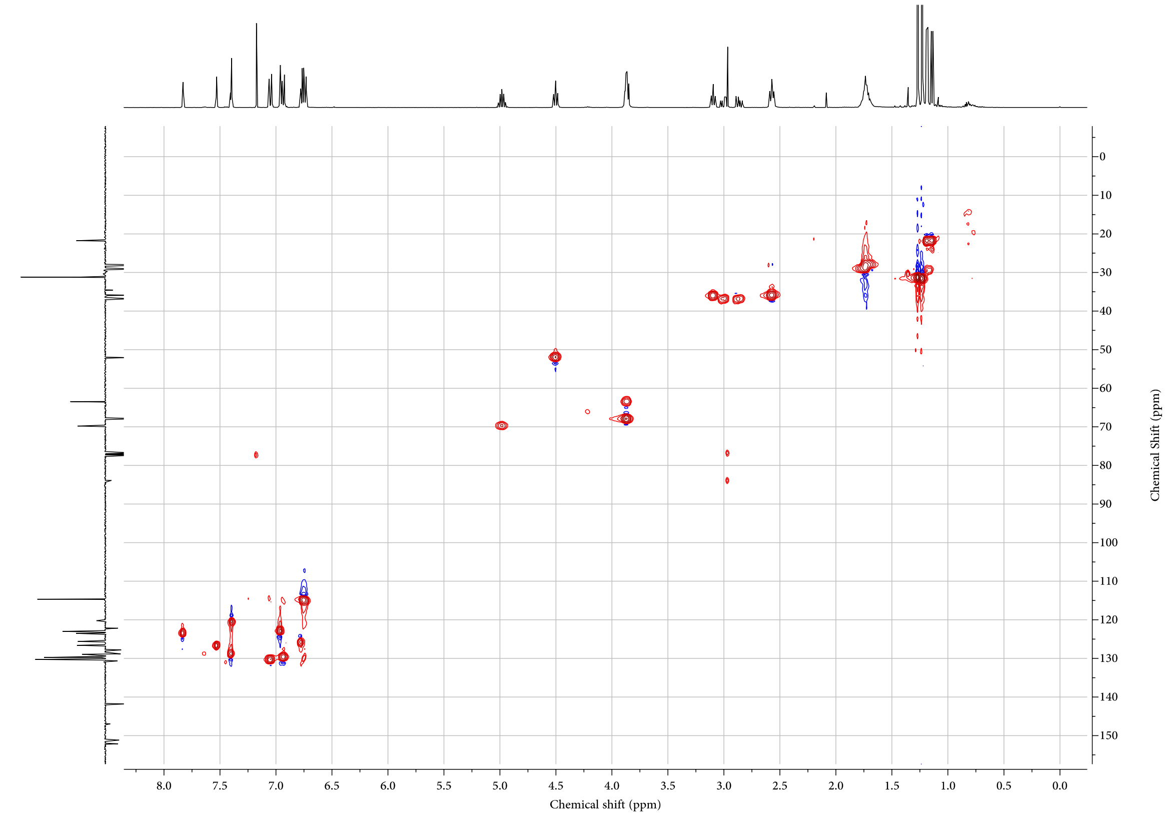

Figure S221. HSQC NMR of (*S*)-**7** ($CDCl_3$, 298 K)

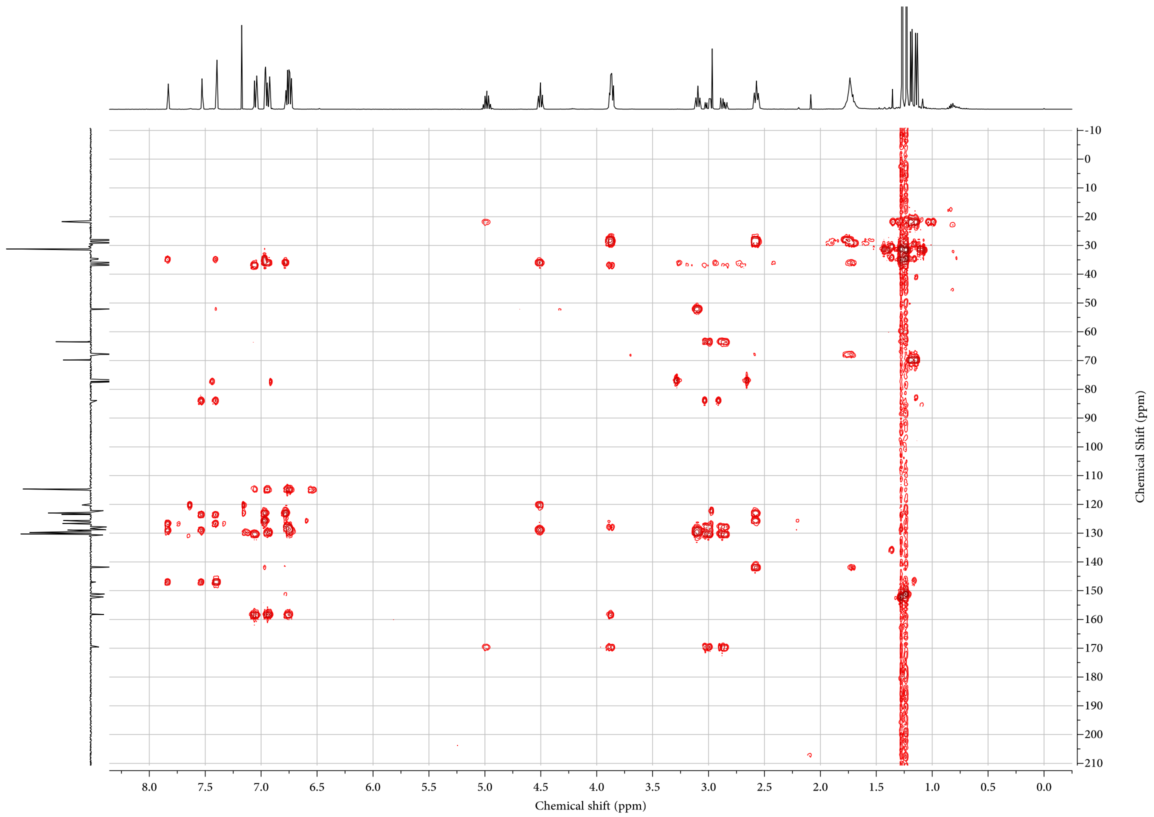

Figure S222. HMBC NMR of (*S*)-**7** ($CDCl_3$, 298 K)

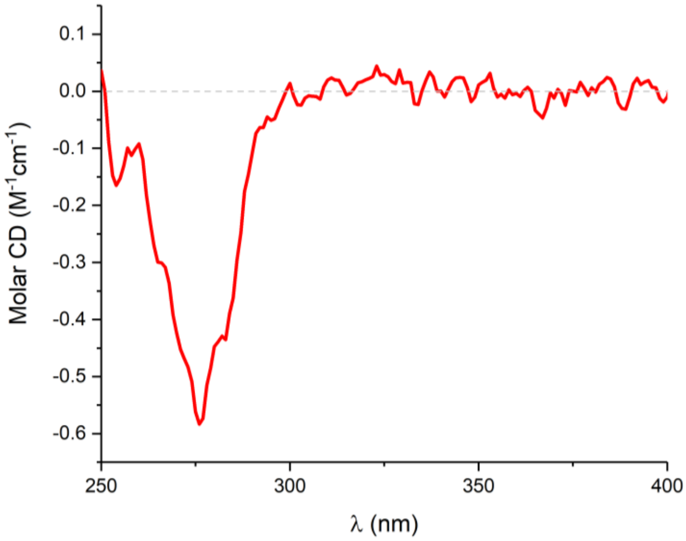

Figure S223. Circular dichroism spectrum of (*S*)-**7** (45.9 µM) at 293 K in $CHCl_3$

Macrocycle precursor 10

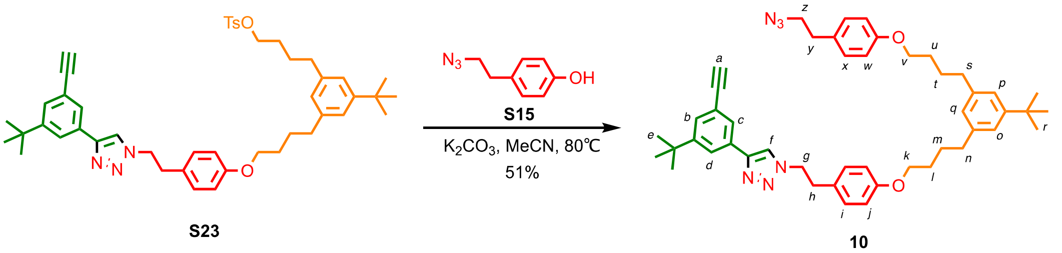

S23 (76 mg, 0.1 mmol, 1.0 equiv.), **S15** (33 mg, 0.2 mmol, 2.0 equiv.) and K_2CO_3 (41 mg, 0.3 mmol, 3.0 equiv.) were suspended in CH_3CN (2 mL) and heated at reflux for 16 h. The reaction mixture was filtered over a Celite® pad, which was washed with EtOAc (10 mL). The washings were combined, and the solvent removed *in vacuo*. Column chromatography (petrol-EtOAc 100 : 0 → 70 : 30) gave **10** as a yellow oil (38 mg, 51%).

^{1}H NMR (400 MHz, $CDCl_3$, 298 K) δ: 7.92 (t, J = 1.8, 1H, H_d), 7.62 (t, J = 1.5, 1H, H_c), 7.51-7.45 (m, 2H, H_b, H_f), 7.12 (d, J = 8.6, 2H, H_x), 7.07-6.98 (m, 4H, H_i, H_o, H_p), 6.89-6.78 (m, 5H, H_j, H_q, H_w), 4.59 (t, J = 7.2, 2H, H_g), 3.96 (app. q, J = 6.0, 4H, H_k, H_v), 3.46 (t, J = 7.2, 2H, H_z), 3.18 (t, J = 7.2, 2H, H_h), 3.06 (s, 1H, H_a), 2.83 (t, J = 7.2, 2H, H_y), 2.66 (t, J = 7.2, 4H, H_n, H_s), 1.92-1.72 (m, 8H, H_l, H_m, H_t, H_u), 1.36 (s, 9H, H_e), 1.32 (s, 9H, H_r).

^{13}C NMR (101 MHz, $CDCl_3$, 298 K) δ: 158.4, 158.1, 152.2, 151.3, 147.1, 141.9, 141.9, 130.7, 130.0, 129.8, 129.8, 129.0, 128.9, 126.7, 125.7, 123.6, 123.1, 123.1, 122.3, 120.3, 114.9, 114.8, 84.0, 77.0, 68.0, 68.0, 52.8, 52.2, 36.1, 36.0, 36.0, 34.9, 34.7, 34.6, 31.6, 31.3, 29.2 (×2), 28.1 (×2).

HR-ESI-MS m/z = 751.4700 $[M+H]^+$ calc. 751.4694 for $C_{48}H_{59}N_6O_2$.

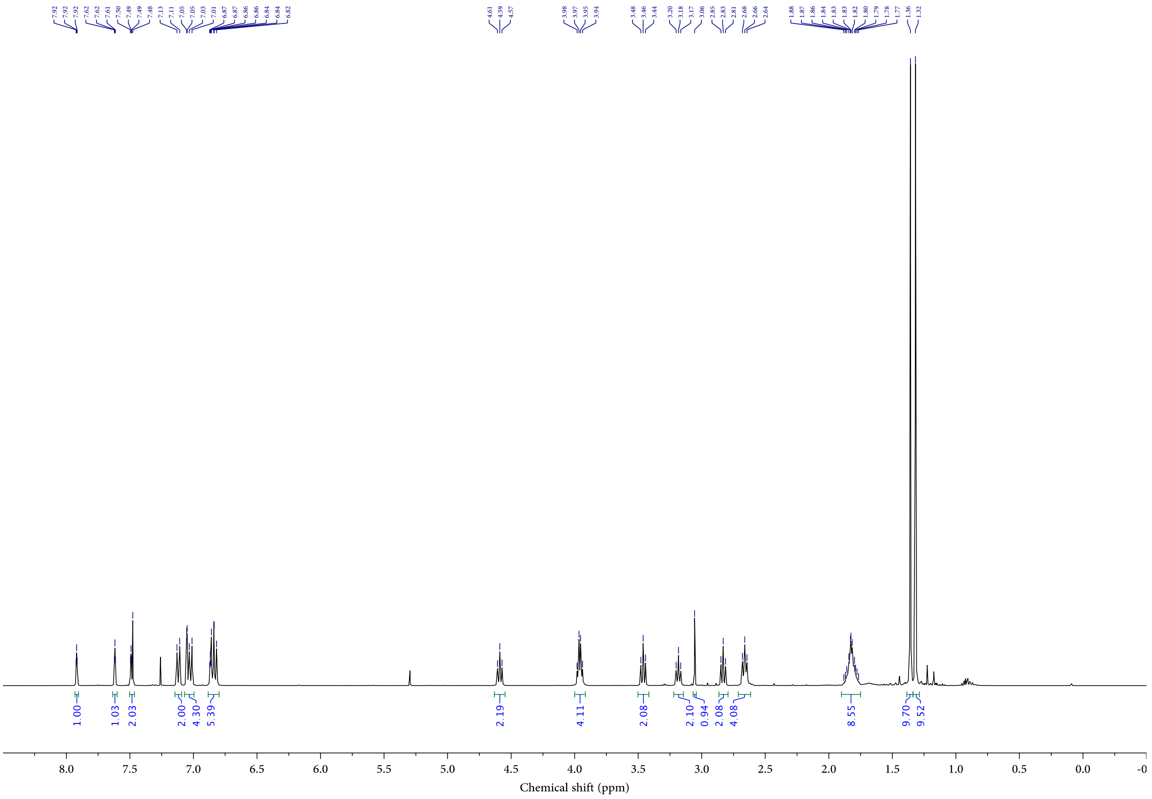

Figure S224. ^{1}H NMR of **10** ($CDCl_3$, 400 MHz, 298 K)

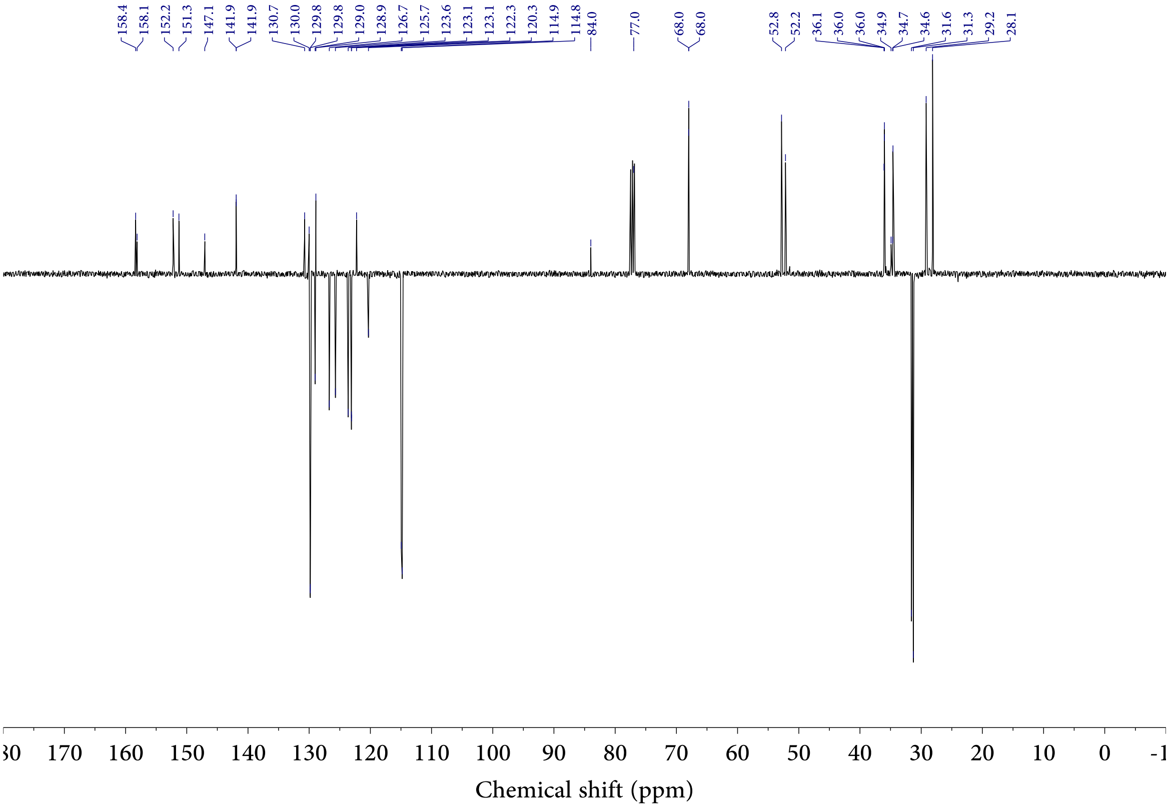

Figure S225. JMOD NMR of **10** ($CDCl_3$, 101 MHz, 298 K)

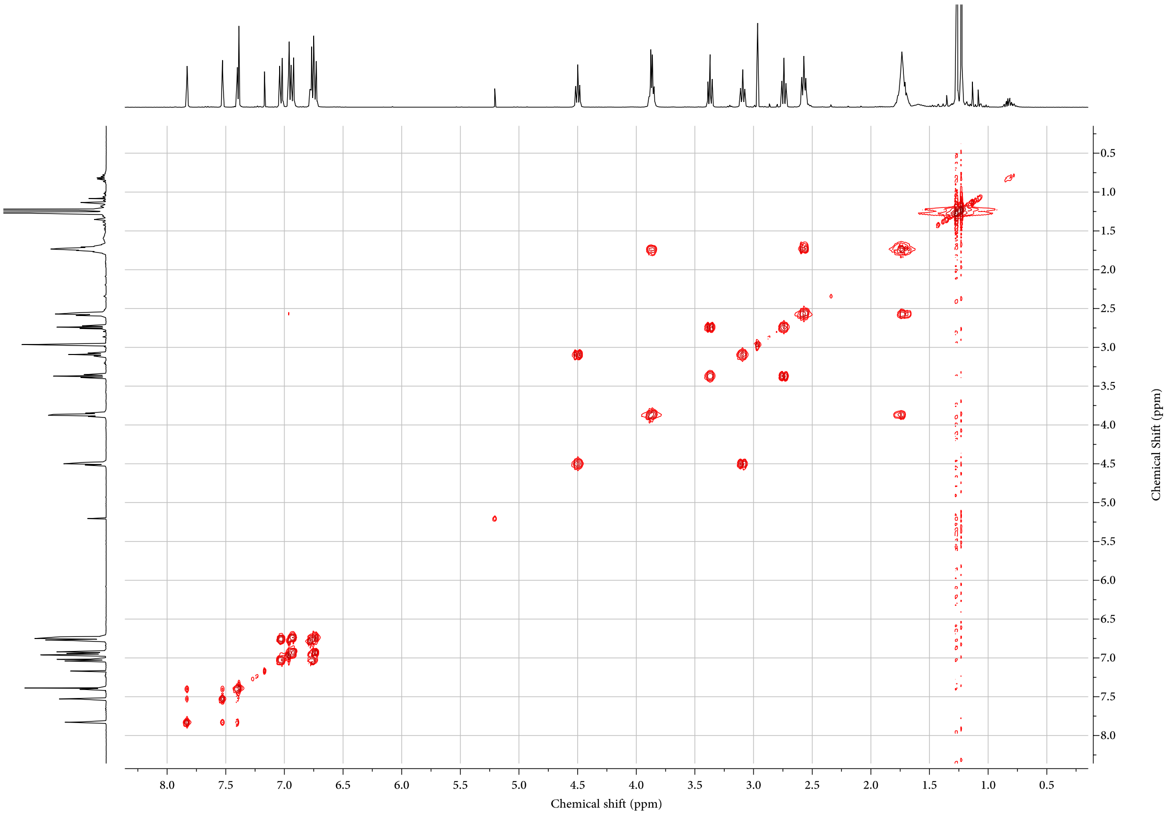

Figure S226. COSY NMR of **10** ($CDCl_3$, 298 K)

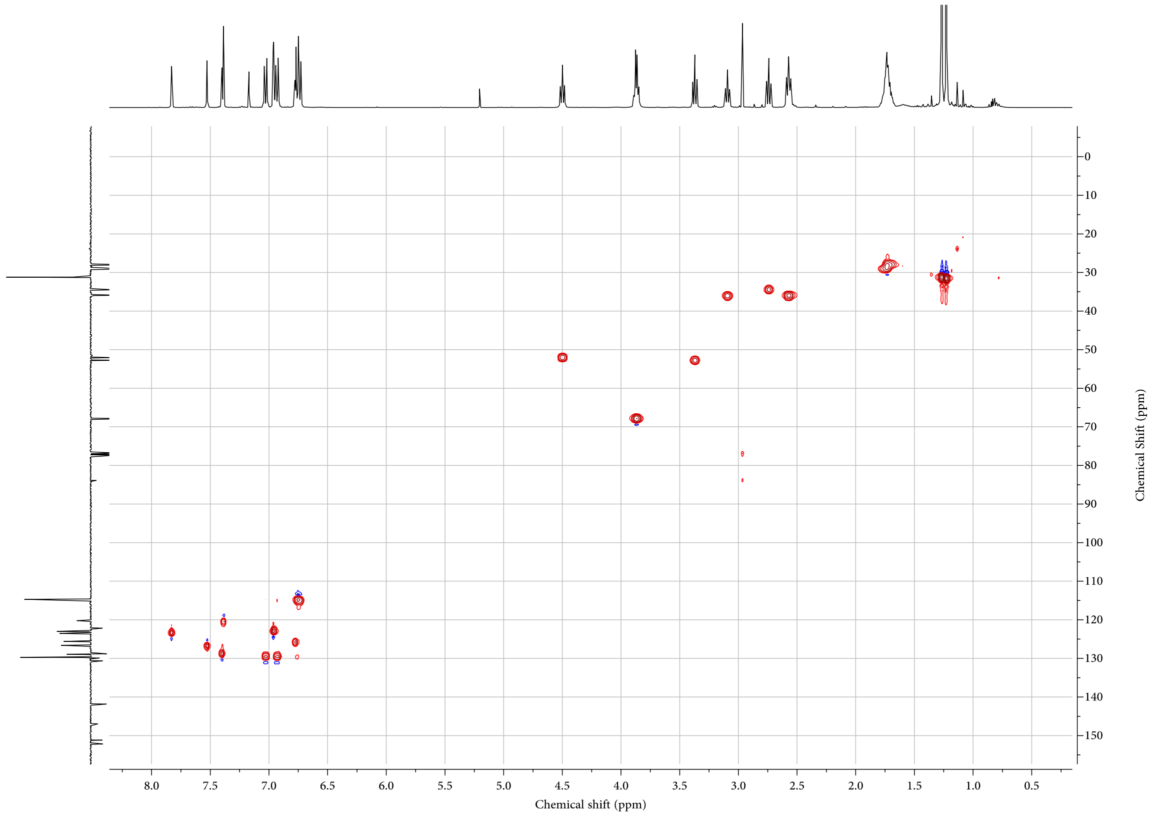

Figure S227. HSQC NMR of **10** ($CDCl_3$, 298 K)

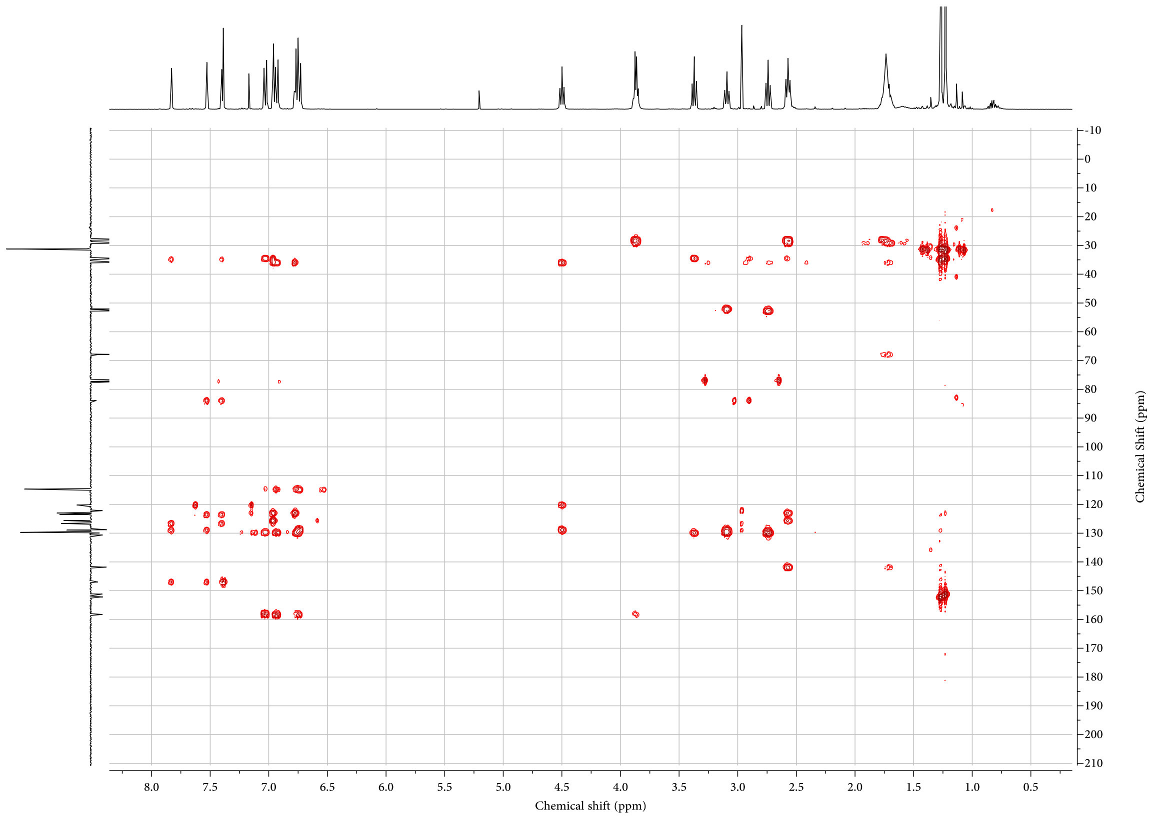

Figure S228. HMBC NMR of **10** ($CDCl_3$, 298 K)

Synthesis of catenane 8

Catenane (*S*,S_{mt})-8

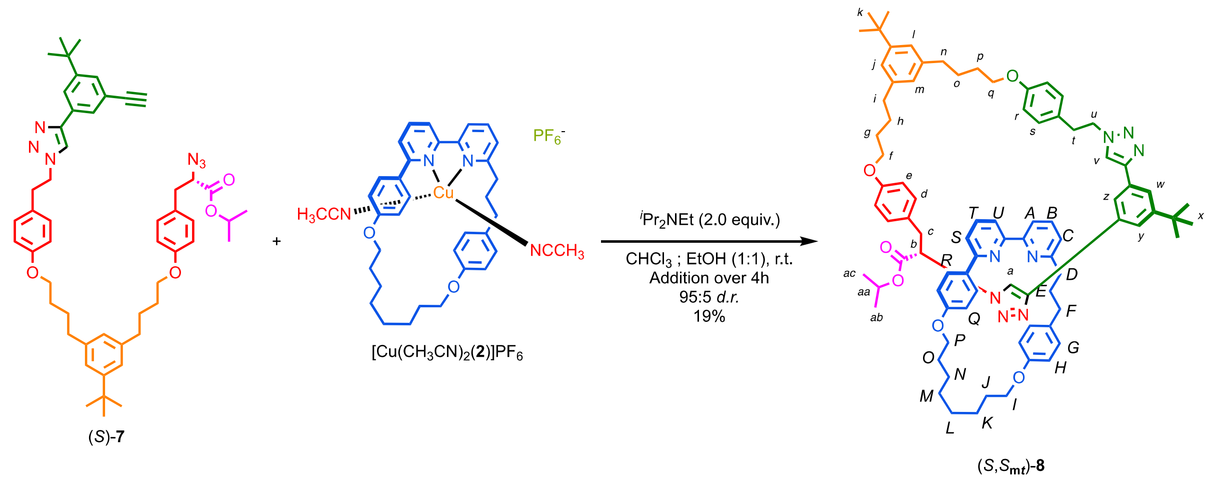

To a solution of [Cu(CH_3CN)$_2$(**2**)]PF_6 (84 mg, 0.11 mmol, 1.0 eq.) and ^{i}Pr$_2$NEt (40 µL, 0.22 mmol, 2.0 eq.) in $CHCl_3$-EtOH (1 : 1, 10 mL) at rt was added a solution of (*S*)-**7** (95 mg, 0.11 mmol, 1.0 eq.) in $CHCl_3$-EtOH (1 : 1, 4.4 mL) over 4 h. Once the addition had finished, H_2O (20 mL) was added followed by KCN (72 mg, 1.1 mmol, 10 equiv.) and the mixture stirred at rt for 16 h. The phases were separated, and the aqueous layer was extracted with $CHCl_3$ (15 mL). The combined organic extracts were dried ($MgSO_4$) and the solvent was removed *in vacuo*. Two rounds of column chromatography (petrol-acetone 100:0 → 90:10→ 80:20→ 70:30; petrol-CH_2Cl_2-CH_3CN 40 : 40 : 20) gave catenane (*S*,S_{mt})-**8** as a colourless oil (28 mg, 19%, 94 : 6 *dr*). The major diastereomer was assigned as (*S*,S_{mt})-**8** by analogy with (*S*, S_{mt})-**3b**. Non-interlocked macrocycle by-product (*S*)-**S26** was also isolated as a yellow oil (19 mg, 19%).

^{1}H NMR (500 MHz, $CDCl_3$, 298 K) δ: 8.97 (s, 1H, major H_a), 8.85 (s, 1H, minor H_a), 7.91 (t, J = 1.8, 1H, H_w), 7.88 (t, *J* = 1.5, 1H, H_z), 7.84 (t, J = 1.7, 1H, H_y), 7.76 (t, *J* = 7.8, 1H, H_B), 7.56 (t, *J* = 7.8, 1H, H_T), 7.53 (d, *J* = 7.5, 1H, H_A), 7.40 (d, *J* = 7.3, 1H, H_U), 7.24 (d, *J* = 7.8, 1H, H_S), 7.20 (d, *J* = 7.6, 1H, H_C), 7.01 (t, *J* = 1.7, 1H, H_j or H_l), 6.99 (t, *J* = 1.7, 1H, H_j or H_l), 6.92 (d, *J* = 8.7, 2H, H_R), 6.90 (s, 1H, H_v), 6.73 (t, *J* = 1.6, 1H, H_m), 6.71 (d, *J* = 8.7, 2H, H_d), 6.63 (d, *J* = 8.5, 2H, H_s), 6.56 (d, *J* = 8.6, 2H, H_e), 6.46 (d, *J* = 8.6, 2H, H_r), 6.21 (d, *J* = 8.5, 2H, H_G), 6.08 (d, *J* = 8.7, 2H, H_Q), 6.02 (d, *J* = 8.5, 2H, H_H), 4.98 (dd, *J* = 9.4, 4.8, 1H, H_b), 4.89 (sept, *J* = 6.6, 1H, H_{aa}), 4.32 (ddd, *J* = 11.6, 7.1, 3.8, 1H, H_u), 4.24-4.06 (m, 1H, $H_{u'}$), 4.04-3.73 (m, 6H, H_I, H_P, H_f or H_q), 3.60-3.47 (m, 2H, H_f or H_q), 3.12-2.90 (m, 4H, H_c, H_t), 2.63-2.41 (m, 6H, H_D, H_i, H_n), 2.36-2.18 (m, 2H, H_F), 1.99-1.84 (m, 2H, H_J), 1.81-1.50 (m, 16H, H_E, H_K, H_N, H_O, H_g, H_h, H_o, H_p), 1.35-1.24 (m, 22H, H_L, H_M, H_k, H_x), 1.13 (d, *J* = 6.3, 3H, H_{ab}), 1.09 (d, *J* = 6.2, 3H H_{ac}).

^{13}C NMR (126 MHz, $CDCl_3$, 298 K) δ 168.1, 164.3, 159.2, 158.9, 157.9, 157.9, 157.8, 157.5, 156.9, 151.2, 150.7, 147.9, 147.3, 142.0, 142.0, 137.0, 136.8, 131.6, 131.4, 130.8, 129.7, 129.5, 129.1, 129.1, 128.5, 128.4, 128.0, 125.7, 125.5, 123.2, 123.1, 123.0, 122.4, 122.3, 121.9, 121.3, 120.8, 119.7, 119.5, 119.5, 114.8, 114.4, 114.2, 113.9, 69.6, 68.0, 67.7 (×2), 67.0, 63.3, 51.7, 37.5, 36.3, 36.2, 35.8, 35.3, 35.0, 35.0, 34.7, 31.6, 31.4, 30.5, 29.8, 29.4, 28.9, 28.8, 28.8, 28.7, 28.2, 25.9, 25.8, 21.7, 21.7.

LR-ESI-MS *m/z* = 1329.8 $[M+H]^+$ for $C_{85}H_{100}N_8O_6$ (see isotope pattern, Figure S235)

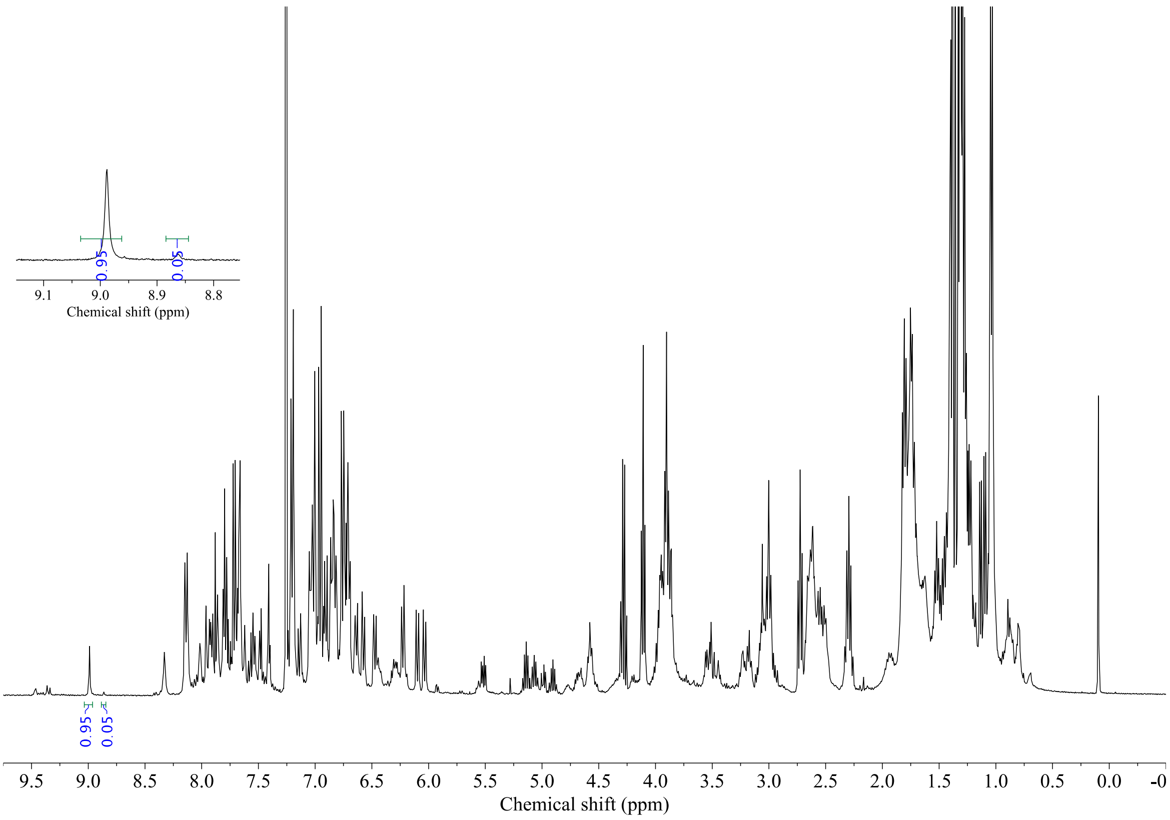

Figure S229. ^{1}H NMR of crude (S,S_{mt})-**8** ($CDCl_3$, 400 MHz, 298 K)

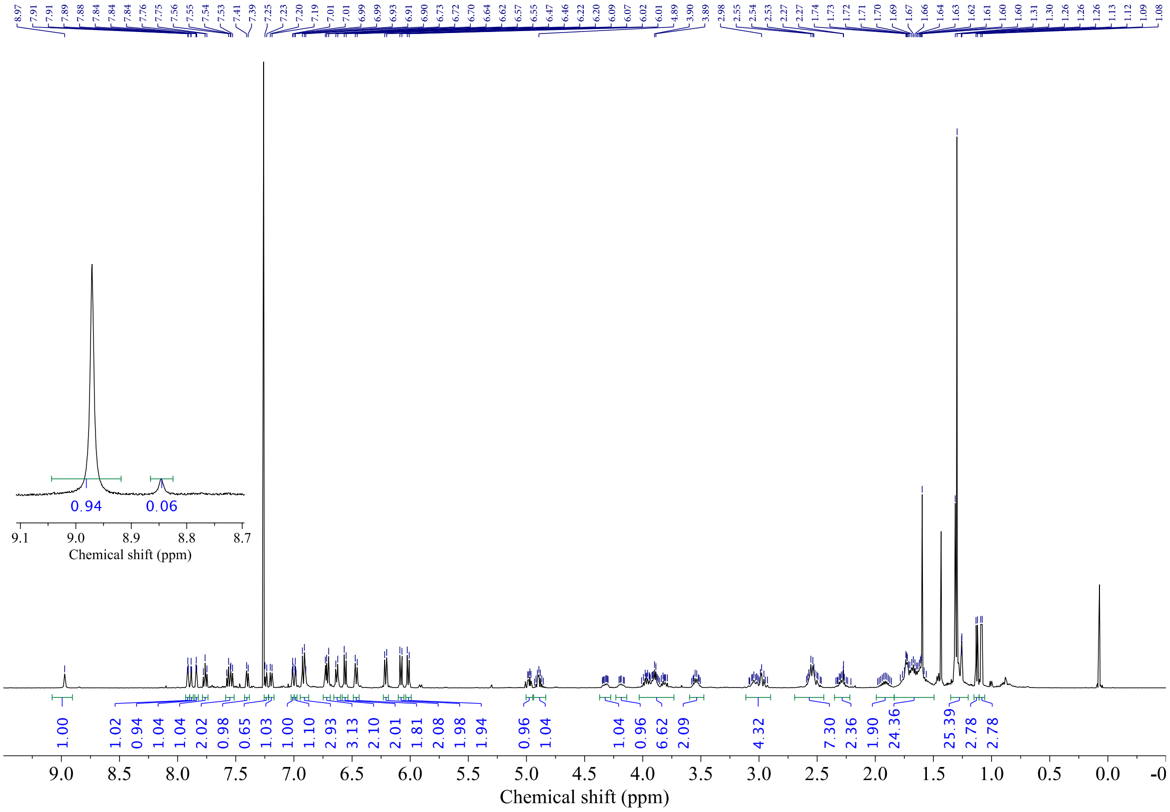

Figure S230. ^{1}H NMR of (S,S_{mt})-**8** ($CDCl_3$, 500 MHz, 298 K)

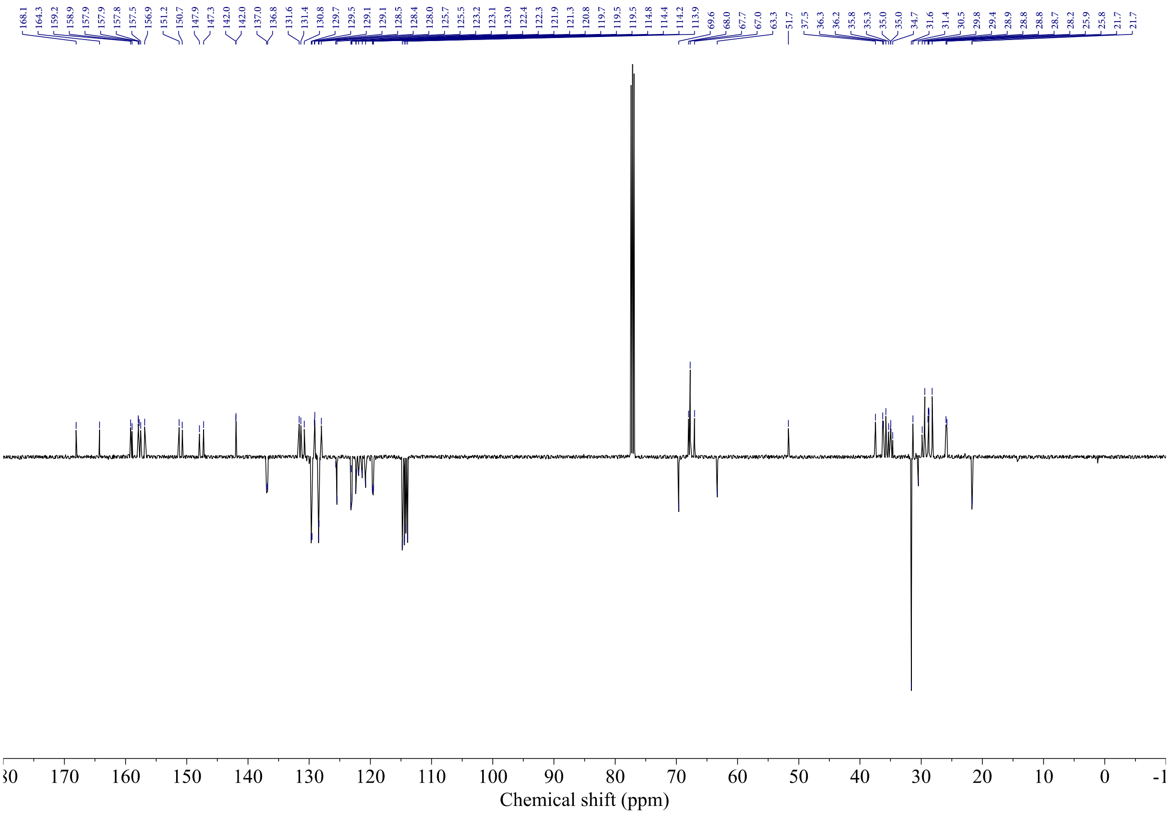

Figure S231. JMOD NMR of (S,S_{mt})-**8** ($CDCl_3$, 126 MHz, 298 K)

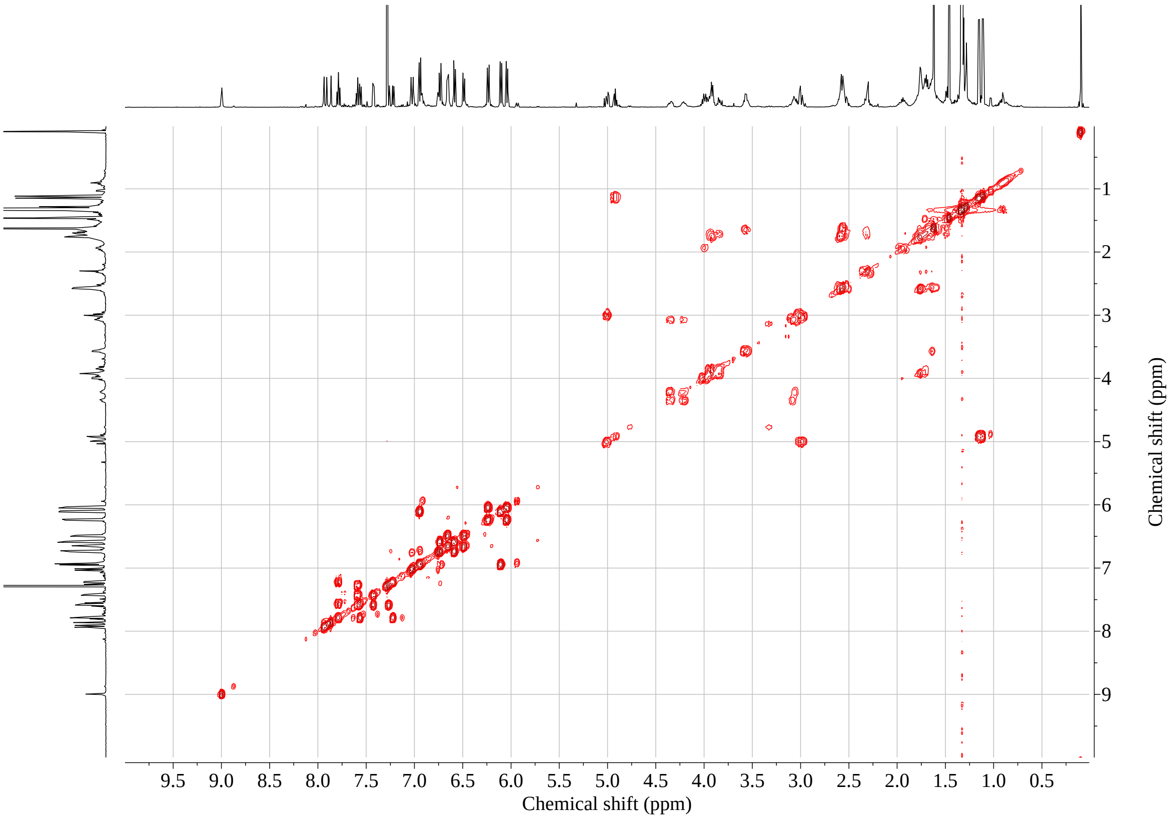

Figure S232. COSY NMR of (S,S_{mt})-**8** ($CDCl_3$, 298 K)

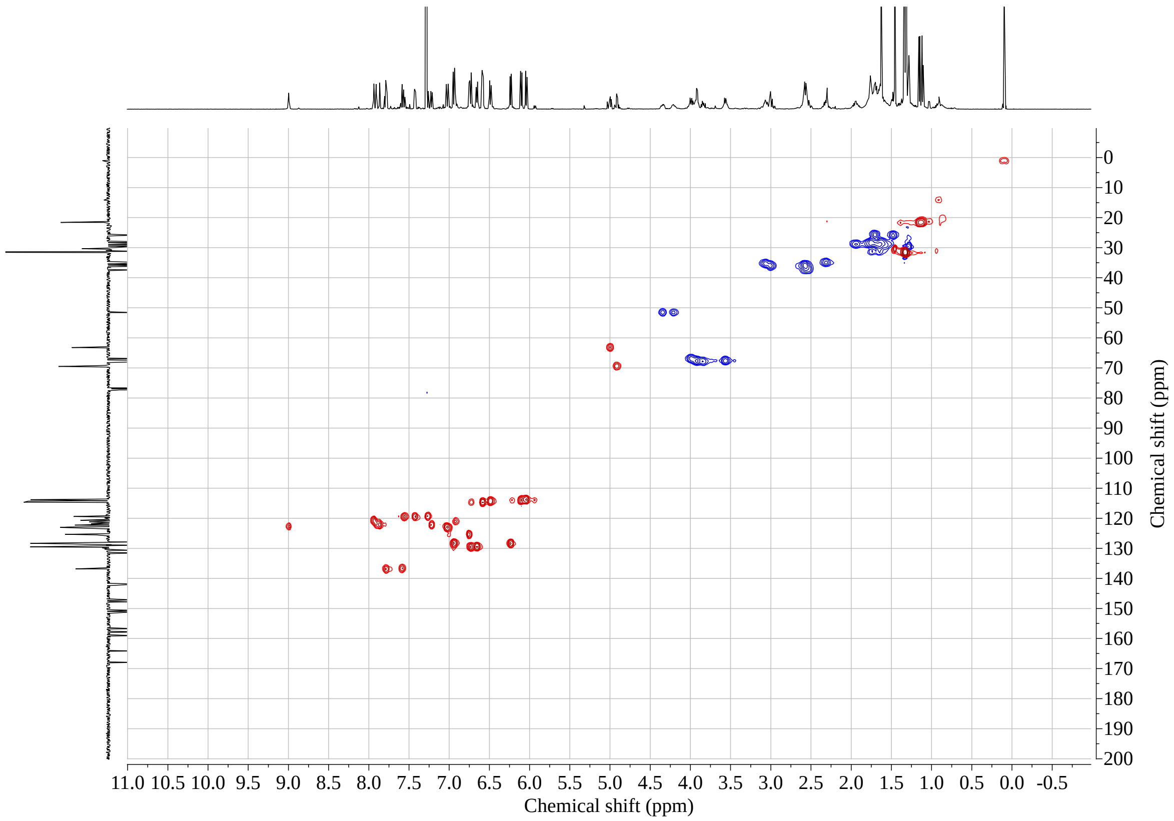

Figure S233. HSQC NMR of (*S*,*S*$_{mt}$)-**8** ($CDCl_3$, 298 K)

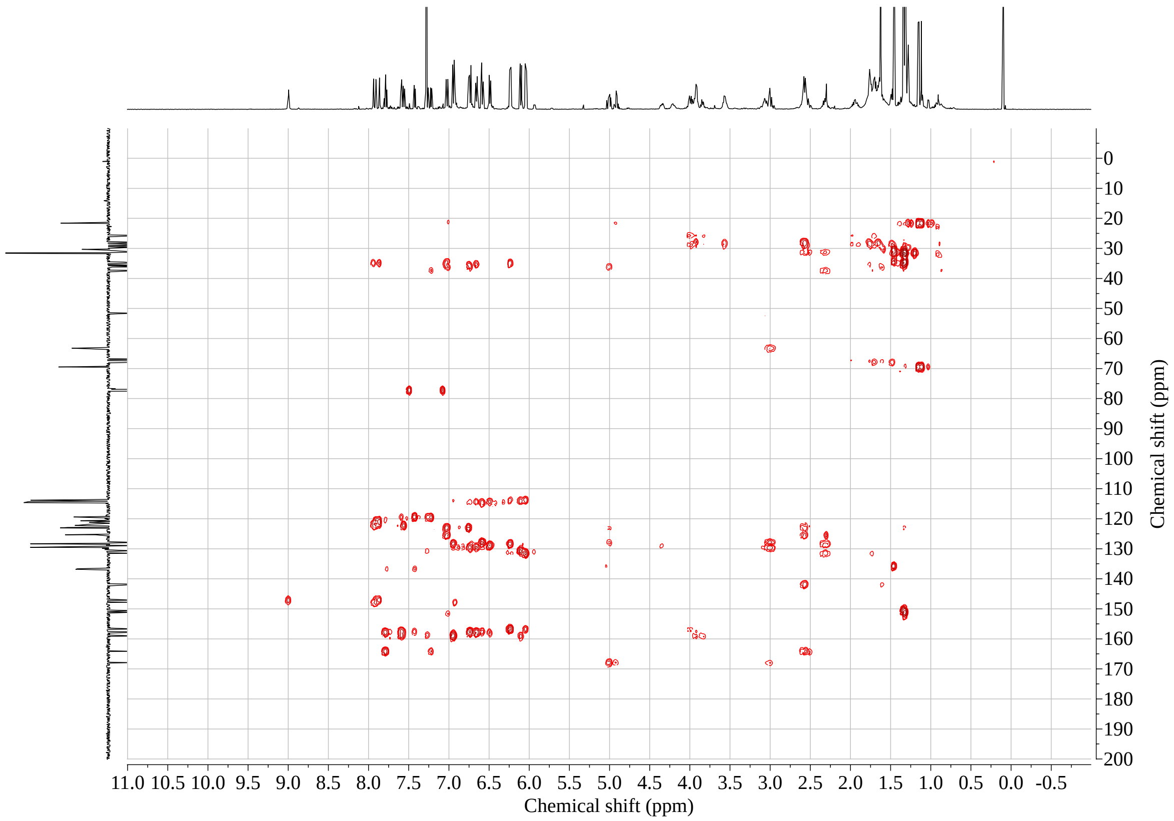

Figure S234. HMBC NMR of (*S*,*S*$_{mt}$)-**8** ($CDCl_3$, 298 K)

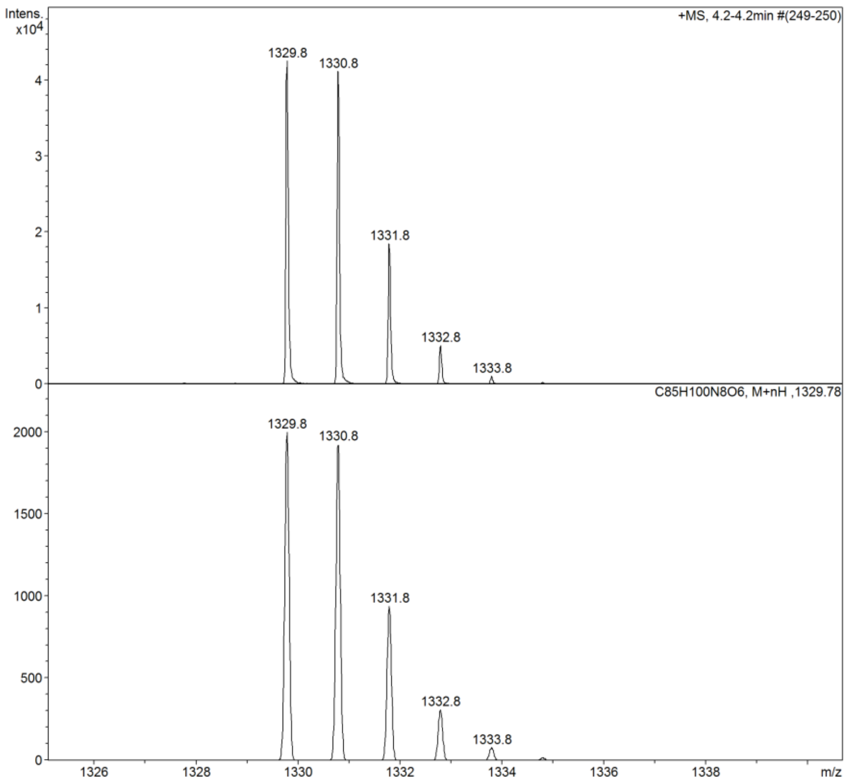

Figure S235. Isotope pattern of (*S*,S$_{mt}$)-**8** $C_{85}H_{100}N_8O_6$

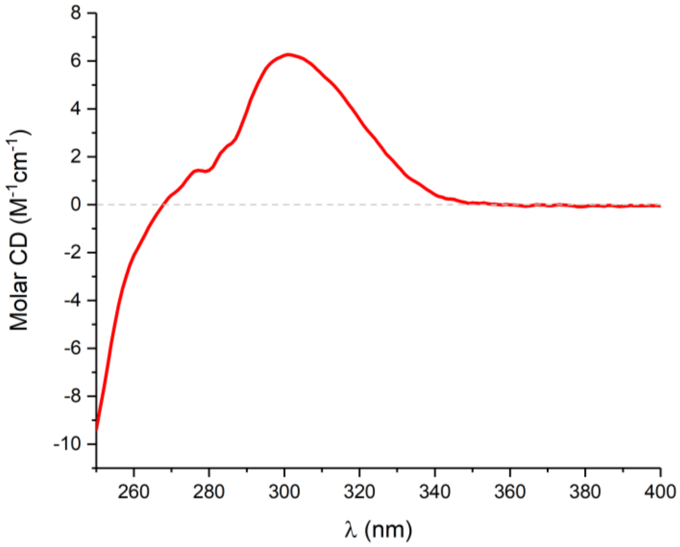

Figure S236. Circular Dichroism Spectra of (*S*,*S*$_{mt}$)-**8** (31.8 µM, *d.r.* (*S*,*S*$_{mt}$)-**8** : (*S*,*R*$_{mt}$)-**8** 94 : 6) at 293 K in $CHCl_3$

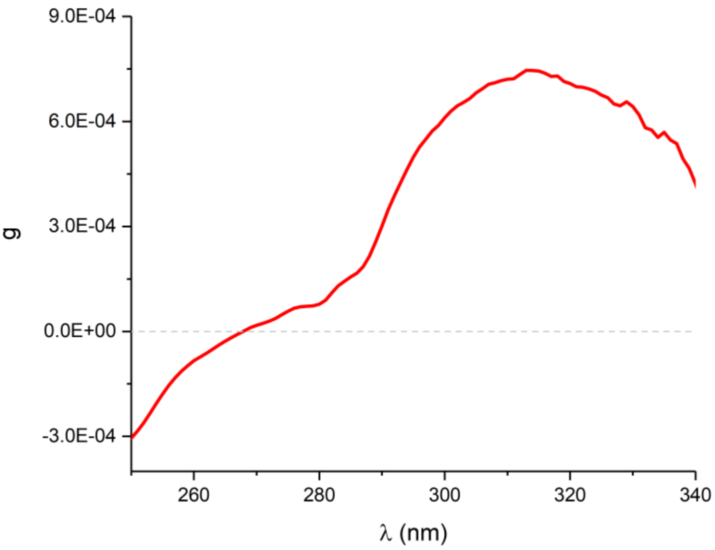

Figure S237. g_{abs} plot of (*S*,*S*$_{mt}$)-**8** (31.8 µM, *d.r.* (*S*,*S*$_{mt}$)-**8** : (*S*,*R*$_{mt}$)-**8** 95 : 5) at 293 K in $CHCl_3$. g_{abs}(max) = 7.5 × 10^{-4} (313 nm).

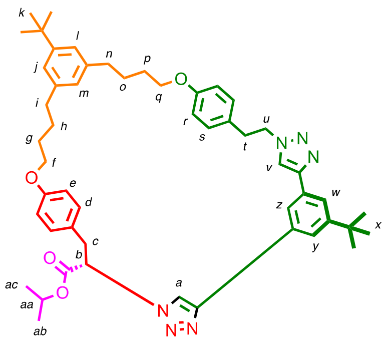

(*S*)-**S26**

^{1}H NMR (400 MHz, $CDCl_3$, 298 K) δ: 7.94 (t, *J* = 1.6, 1H, H_w), 7.86 (s, 1H, H_a), 7.80 (t, *J* = 1.7, 1H, H_z), 7.78 (t, *J* = 1.7, 1H, H_y), 7.40 (s, 1H, H_v), 6.99 (d, *J* = 1.5, 2H, H_j, H_l), 6.89 (d, *J* = 8.7, 2H, H_d or H_s), 6.85 (d, *J* = 8.6, 2H, H_d or H_s), 6.80 (t, *J* = 1.6, 1H, H_m), 6.70 (app. t, *J* = 8.6, 4H, H_e, H_r), 5.50 (dd, *J* = 10.6, 4.4, 1H, H_b), 5.13 (hept, *J* = 6.3, 1H, H_{aa}), 4.69 (ddd, *J* = 13.6, 6.8, 4.2, 1H, H_u), 4.55 (ddd, *J* = 13.6, 7.9, 4.3, 1H, $H_{u'}$), 3.94-3.72 (m, 4H, H_f, H_q), 3.58-3.40 (m, 2H, H_c), 3.33-3.15 (m, 2H, H_t), 2.67-2.44 (m, 4H, H_i, H_n), 1.85-1.67 (m, 8H, H_g, H_h, H_o, H_p), 1.37 (s, 9H, H_x), 1.31 (d, *J* = 6.2, 3H, H_{ab}), 1.29-1.27 (m, 12H, H_k, H_{ac}).

^{13}C NMR (101 MHz, $CDCl_3$, 298 K) δ 168.0, 158.5, 158.2, 152.5, 151.2, 147.6, 147.3, 141.9, 131.1, 130.8, 129.9, 129.7, 128.9, 126.7, 125.4, 123.2, 123.2, 122.7, 122.5, 121.0, 120.4, 120.1, 114.9, 114.8, 70.7, 68.0, 67.9, 64.6, 52.1, 37.7, 36.0, 35.9, 35.4, 35.1, 34.6, 31.6, 31.5, 29.1, 29.0, 28.2, 28.2, 21.8, 21.8.

HR-ESI-MS *m/z* = 837.5080 $[M+H]^+$ calc. 837.5062 for $C_{52}H_{65}N_6O_4$.

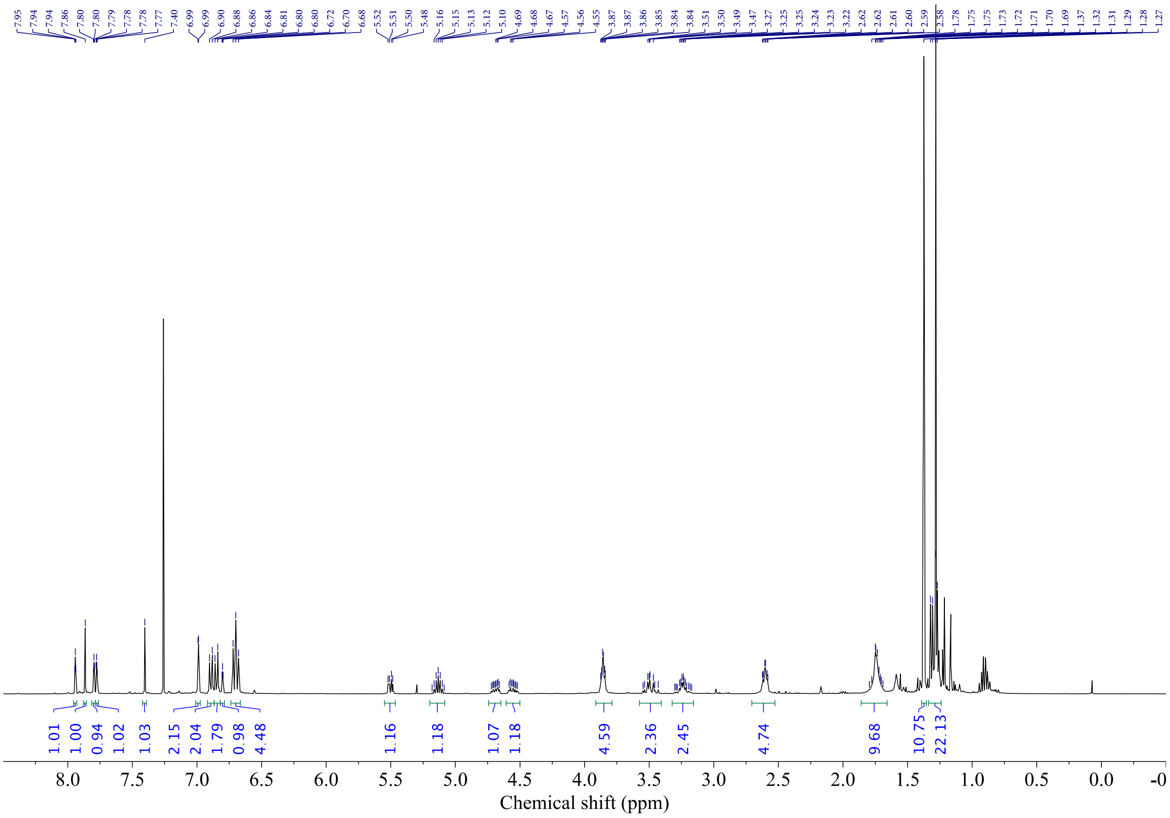

Figure S238. ^{1}H NMR of (*S*)-**S26** ($CDCl_3$, 400 MHz, 298 K)

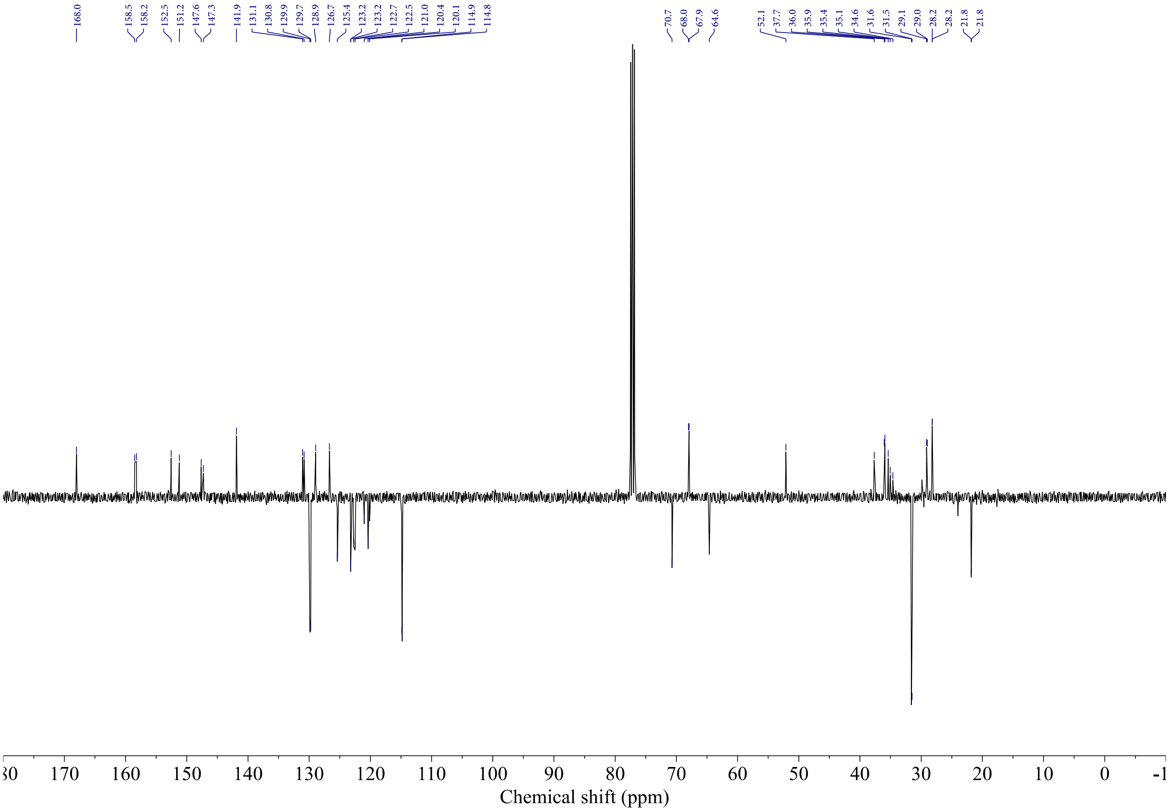

Figure S239. JMOD NMR of (*S*)-**S26** ($CDCl_3$, 126 MHz, 298 K)

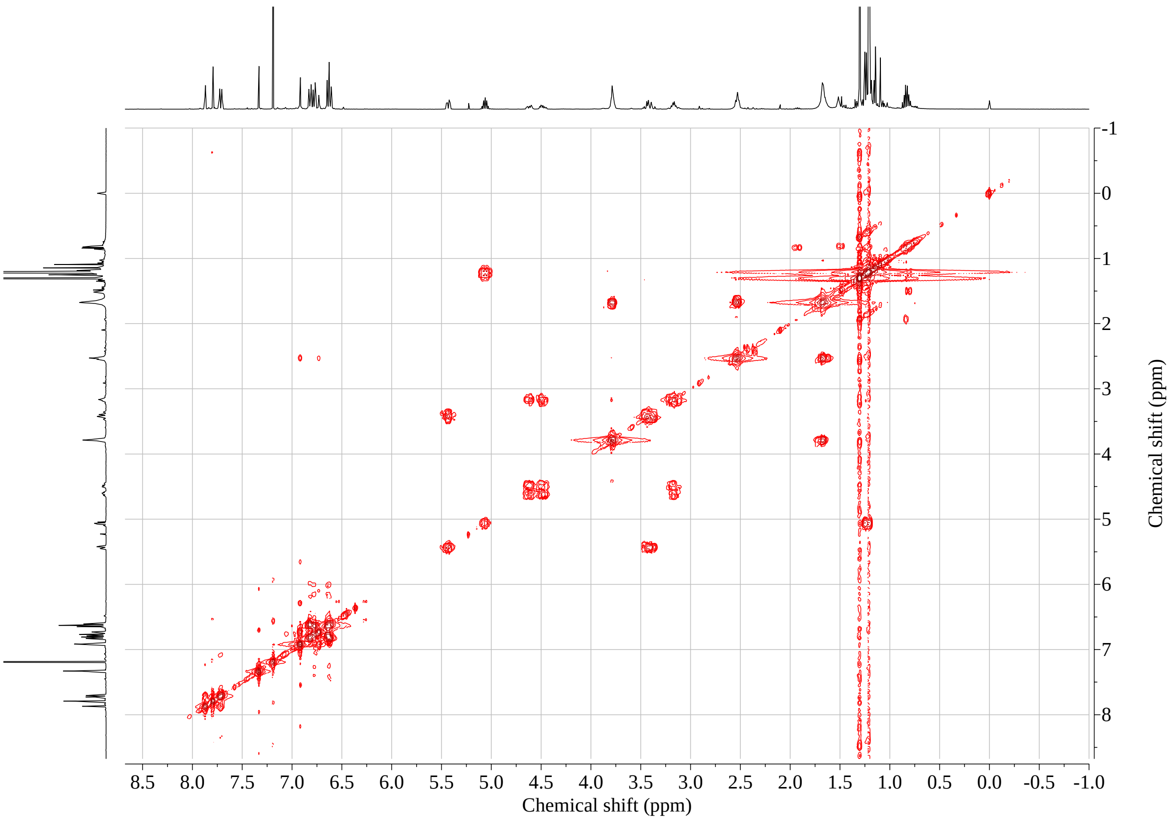

Figure S240. COSY NMR of (*S*)-**S26** ($CDCl_3$, 298 K)

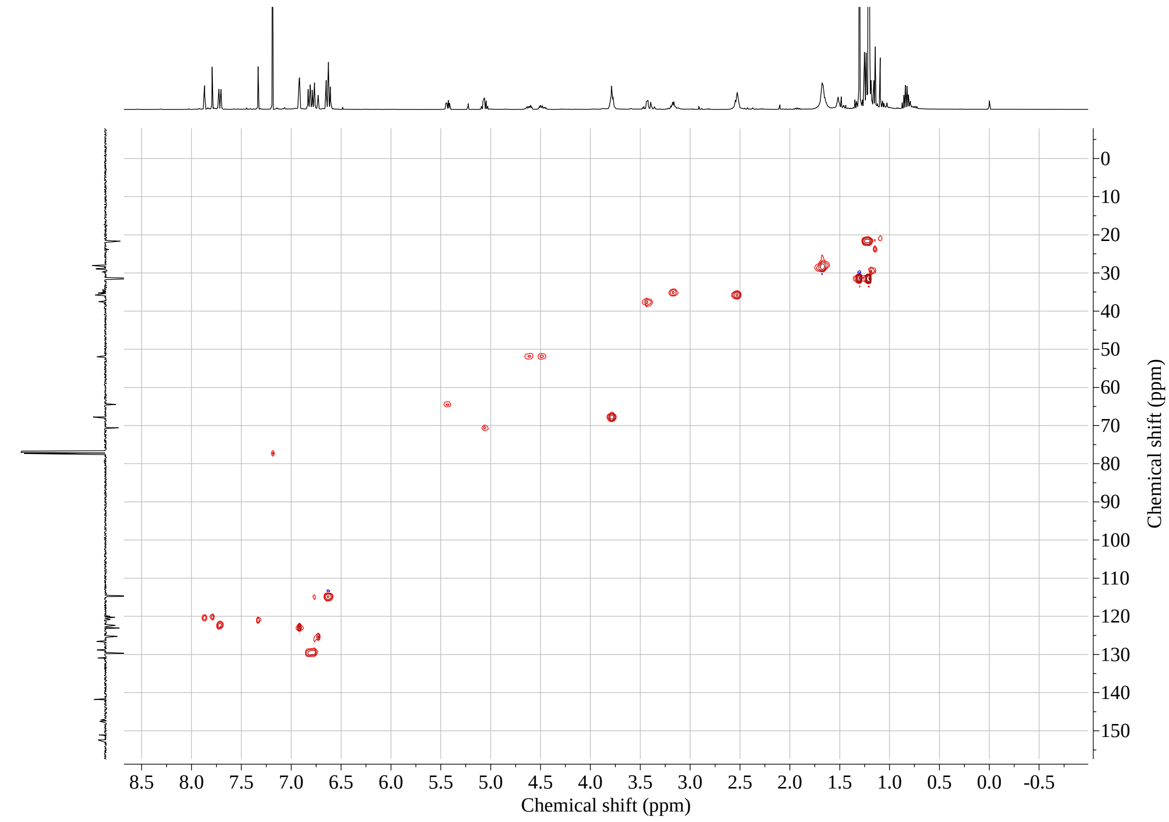

Figure S241. HSQC NMR of (*S*)-**S26** ($CDCl_3$, 298 K)

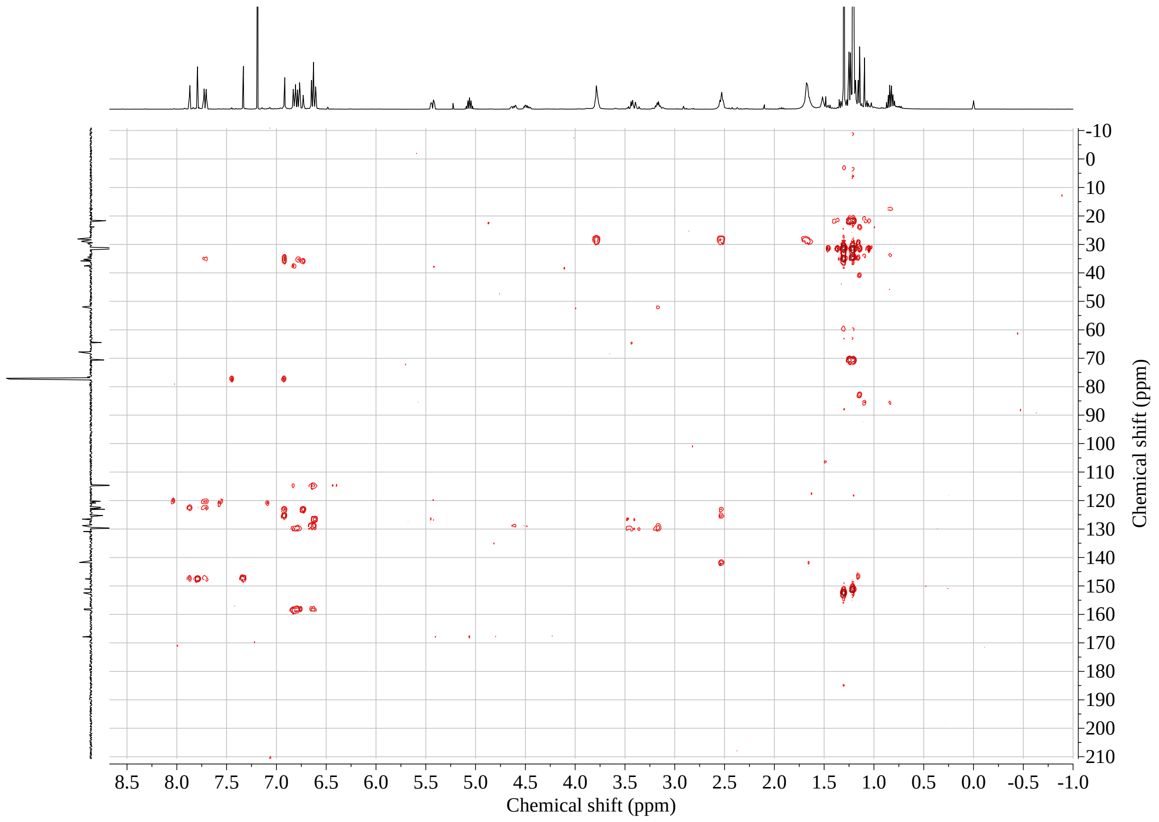

Figure S242. HMBC NMR of (*S*)-**S26** ($CDCl_3$, 298 K)

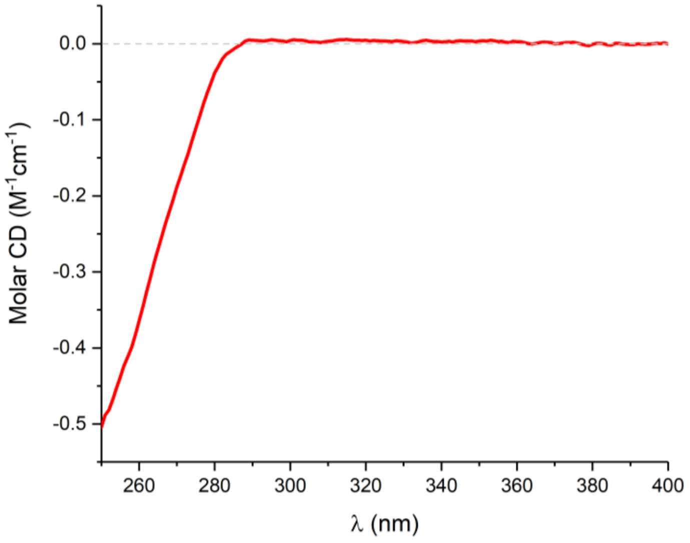

Figure S243. Circular dichroism spectrum of (*S*)-**S26** (637 μM) at 293 K in $CHCl_3$

Synthesis of *rac*-9 and ($S_{co\text{-}mt}$)-9

Catenane *rac*-9

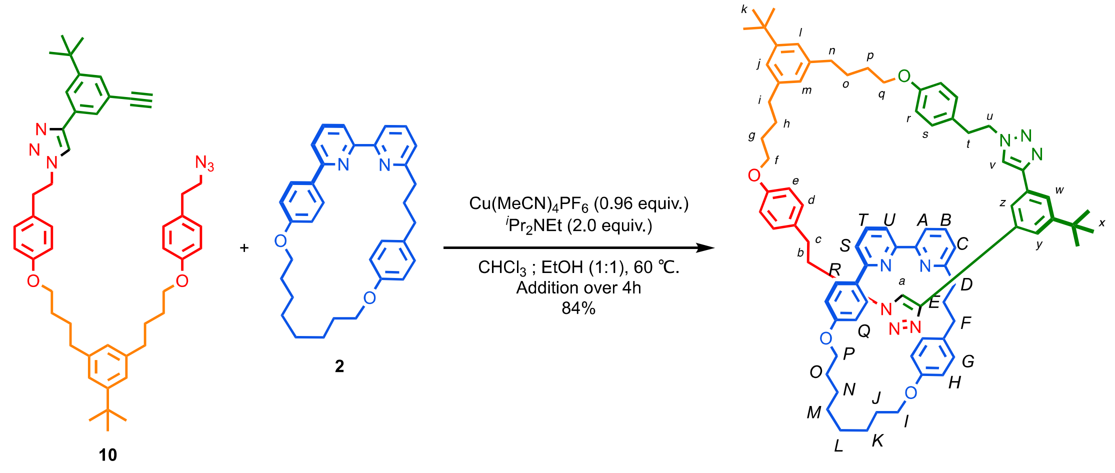

10 + 2 → *rac*-9

To a solution of [Cu(CH$_3$CN)$_4$]PF$_6$ (8.9 mg, 0.024 mmol, 0.96 eq.), **2** (12 mg, 0.025 mmol, 1.0 eq.) and ^{i}Pr$_2$NEt (9 µL, 0.05 mmol, 2.0 eq.) in CHCl$_3$-EtOH (1 : 1, 2.5 mL) at 60 ºC was added a solution of **10** (19 mg, 0.025 mmol, 1.0 eq.) in CHCl$_3$-EtOH (1 : 1, 1 mL) over 4 h. Once the addition had finished, H_2O (10 mL) was added followed by KCN (16 mg, 0.24 mmol, 10 equiv.) and the mixture stirred at rt for 16 h. The phases were separated, and the aqueous layer was extracted with CHCl$_3$ (10 mL). The combined organic extracts were dried (MgSO$_4$) and the solvent was removed *in vacuo*. Two rounds of column chromatography (hexane-EtOAc 90 : 10 → 80 : 20 → 50 : 50; hexane-acetone 80 : 20 → 60 : 40→ 50 : 50) gave catenane *rac*-**9** as a yellow oil (26 mg, 84%).

^{1}H NMR (500 MHz, CDCl$_3$, 298 K) δ: 8.63 (s, 1H, H_a), 7.89 (s, 1H, H_w), 7.76 (s, 1H, H_y), 7.64-7.50 (m, 3H, H_B, H_T, H_z), 7.42 (d, *J* = 7.8, 1H, H_A), 7.38 (d, *J* = 7.7, 1H, H_U), 7.32 (d, *J* = 7.8, 1H, H_S), 7.16 (d, *J* = 8.4, 2H, H_R), 7.04-6.99 (m, 2H, H_C, H_v), 6.97-6.94 (m, 2H, H_j, H_l), 6.86 (d, *J* = 8.5, 2H, H_s), 6.69-6.61 (m, 5H, H_d, H_m, H_r), 6.52 (d, *J* = 8.4, 2H, H_e), 6.34 (app. t, *J* = 8.6, 4H, H_Q, H_G), 6.24 (d, *J* = 8.5, 2H, H_H), 4.55-4.39 (m, 2H, H_u), 4.14 (dt, *J* = 13.6, 6.5, 1H, H_b), 4.01 (q, *J* = 7.6, 1H, H_I), 3.97-3.86 (m, 3H, $H_{I'}$, H_P), 3.80 (dt, *J* = 13.1, 5.9, 1H, $H_{b'}$), 3.70-57 (m, 3H, H_f, H_q), 3.42- 3.34 (m, 1H, $H_{f'}$), 3.28-3.11 (m, 2H, H_t), 2.86-2.77 (m, 2H, H_c), 2.50 (t, *J* = 7.4, 2H, H_n or H_i), 2.46-2.33 (m, 4H, H_D, H_n or H_i), 2.34-2.20 (m, 2H, H_F), 1.94-1.76 (m, 2H, H_J), 1.73-1.38 (m, 20H, H_E,H_K, H_L, H_M, H_N, H_O, H_g, H_h, H_o, H_p), 1.28 (s, 9H, $H_{k\ or}$ H_x), 1.25 (s, 9H, H_k or H_x).

^{13}C NMR (126 MHz, CDCl$_3$, 298 K) δ 163.4, 159.1, 158.9, 158.2, 158.0 (×2), 157.7, 157.0, 151.8, 151.1, 147.7, 146.8, 142.2, 141.8, 137.0, 136.8, 132.3, 131.8, 131.7, 130.1, 129.8, 129.2, 129.1, 129.1, 129.0, 128.7, 125.4, 123.1, 123.0, 122.8, 122.6, 122.1, 121.5, 120.9, 120.6, 120.1, 119.8, 119.7, 114.8, 114.7, 114.6, 114.1, 67.9, 67.7, 67.6, 66.6, 51.9, 50.0, 37.4, 36.1, 35.9, 35.2, 35.0, 34.9, 34.6, 33.8, 31.7, 31.6, 31.4, 29.5, 29.2, 28.9, 28.8, 28.8, 28.5, 28.1, 28.0, 26.0, 25.8.

LR-ESI-MS *m/z* = 1243.7 [M+H]$^+$ for $C_{81}H_{94}N_8O_4$ (see isotope pattern, Figure S252)

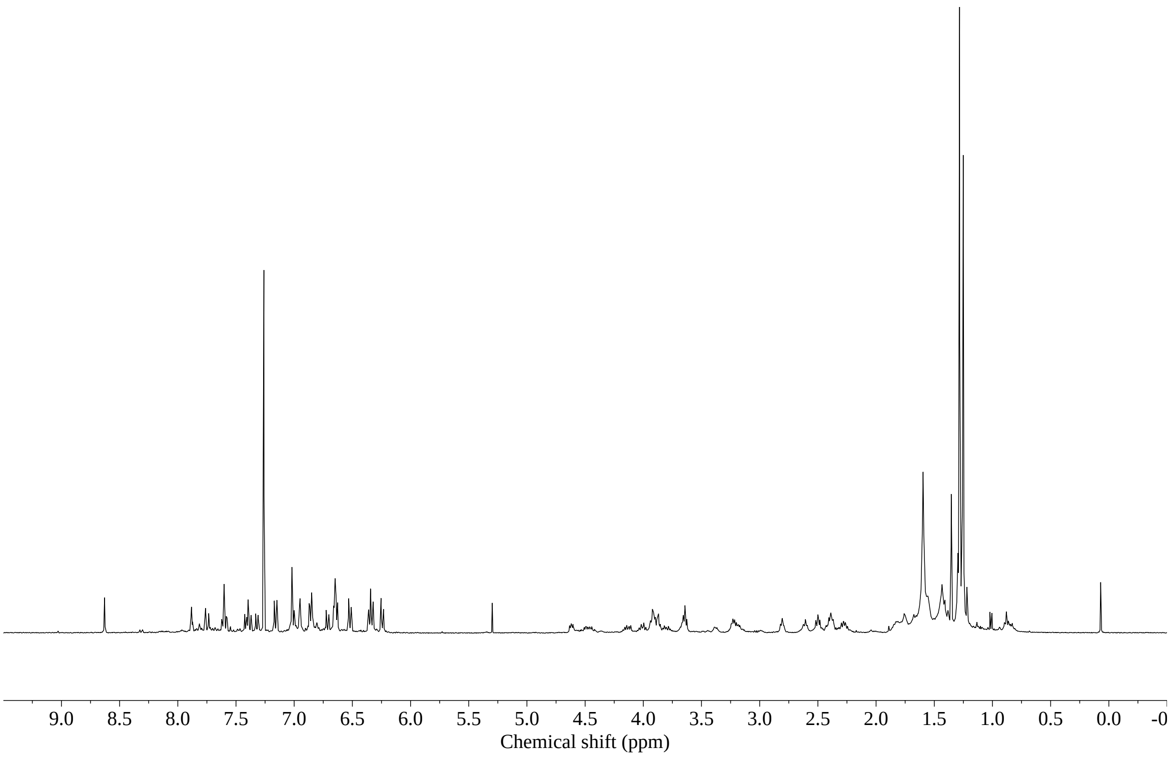

Figure S244. ^{1}H NMR of crude *rac*-**9** ($CDCl_3$, 400 MHz, 298 K)

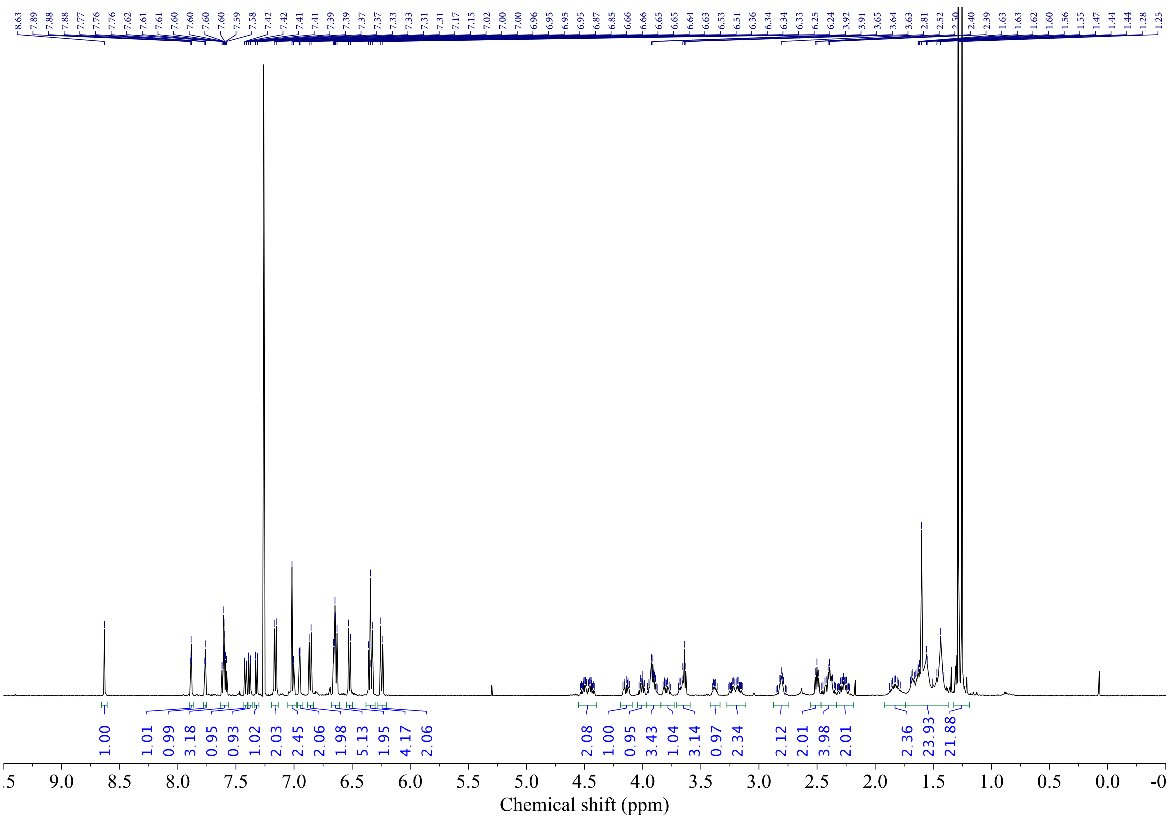

Figure S245. ^{1}H NMR of *rac*-**9** ($CDCl_3$, 500 MHz, 298 K)

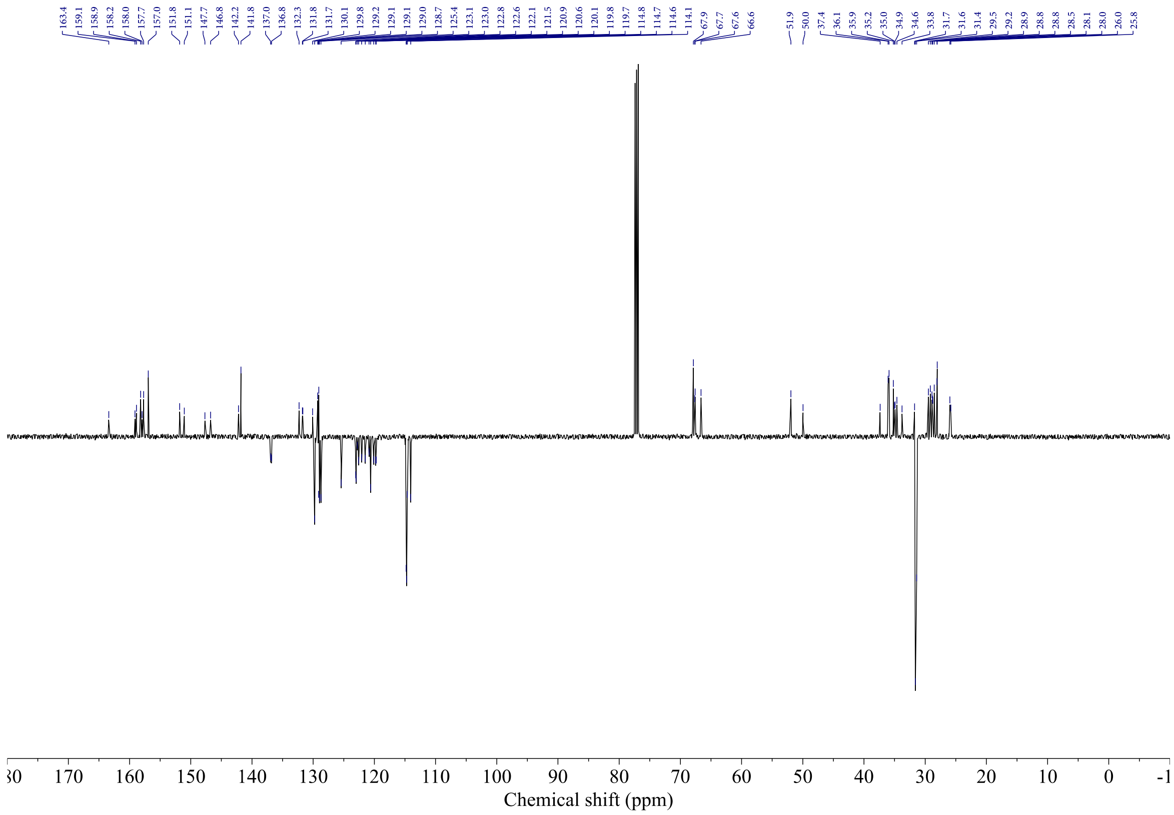

Figure S246. JMOD NMR of *rac*-**9** ($CDCl_3$, 126 MHz, 298 K)

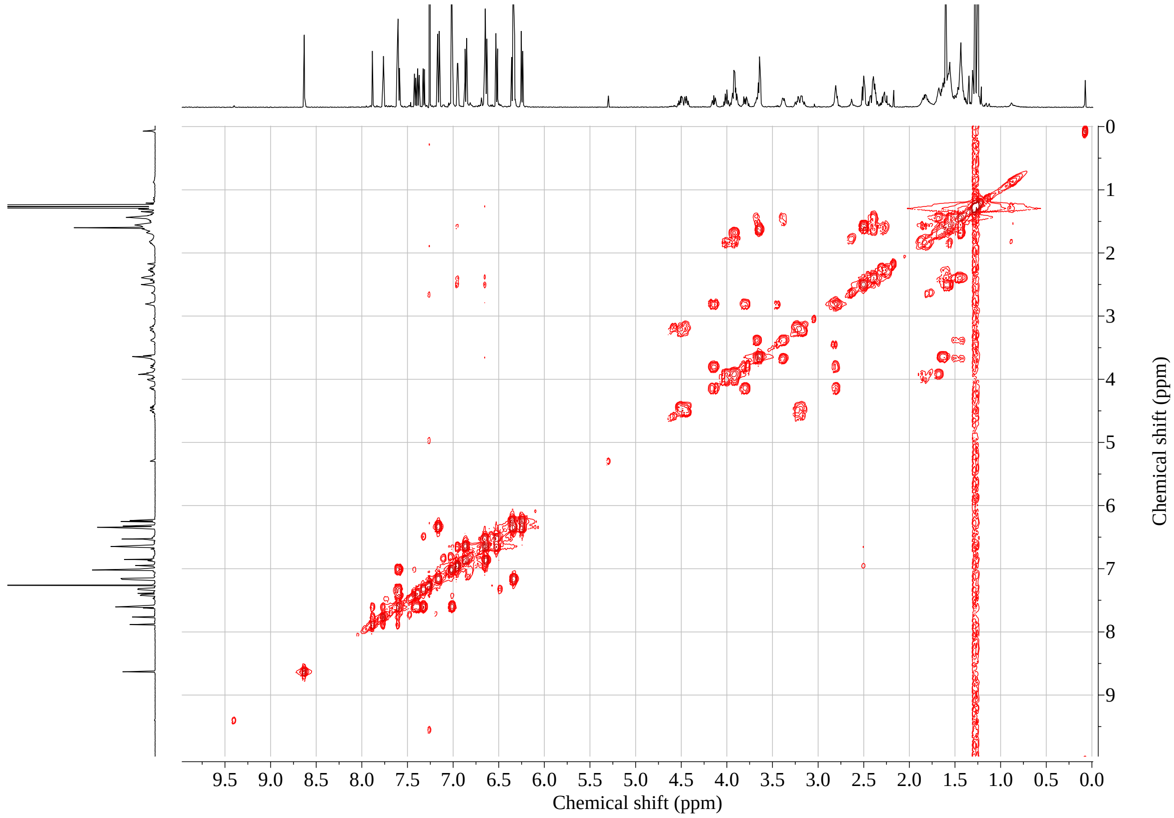

Figure S247. COSY NMR of *rac*-**9** ($CDCl_3$, 298 K)

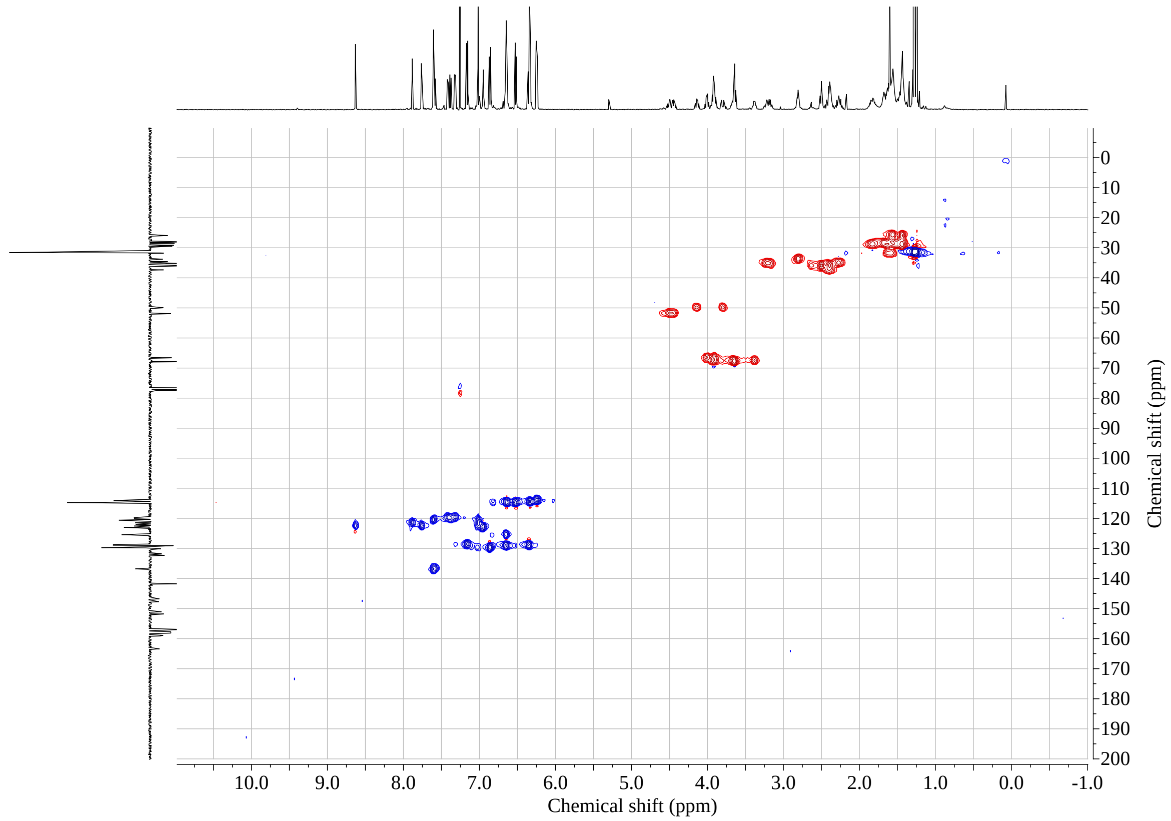

Figure S248. HSQC NMR of *rac*-**9** ($CDCl_3$, 298 K)

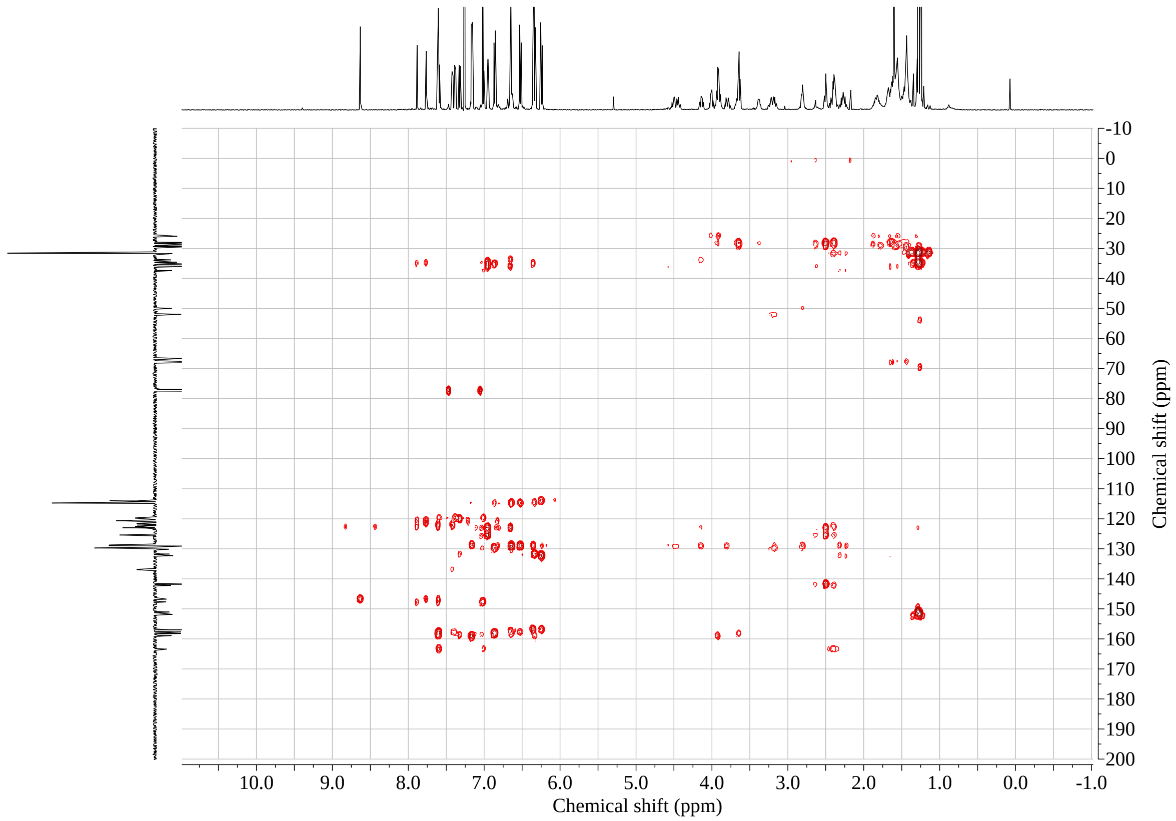

Figure S249. HMBC NMR of *rac*-**9** ($CDCl_3$, 298 K)

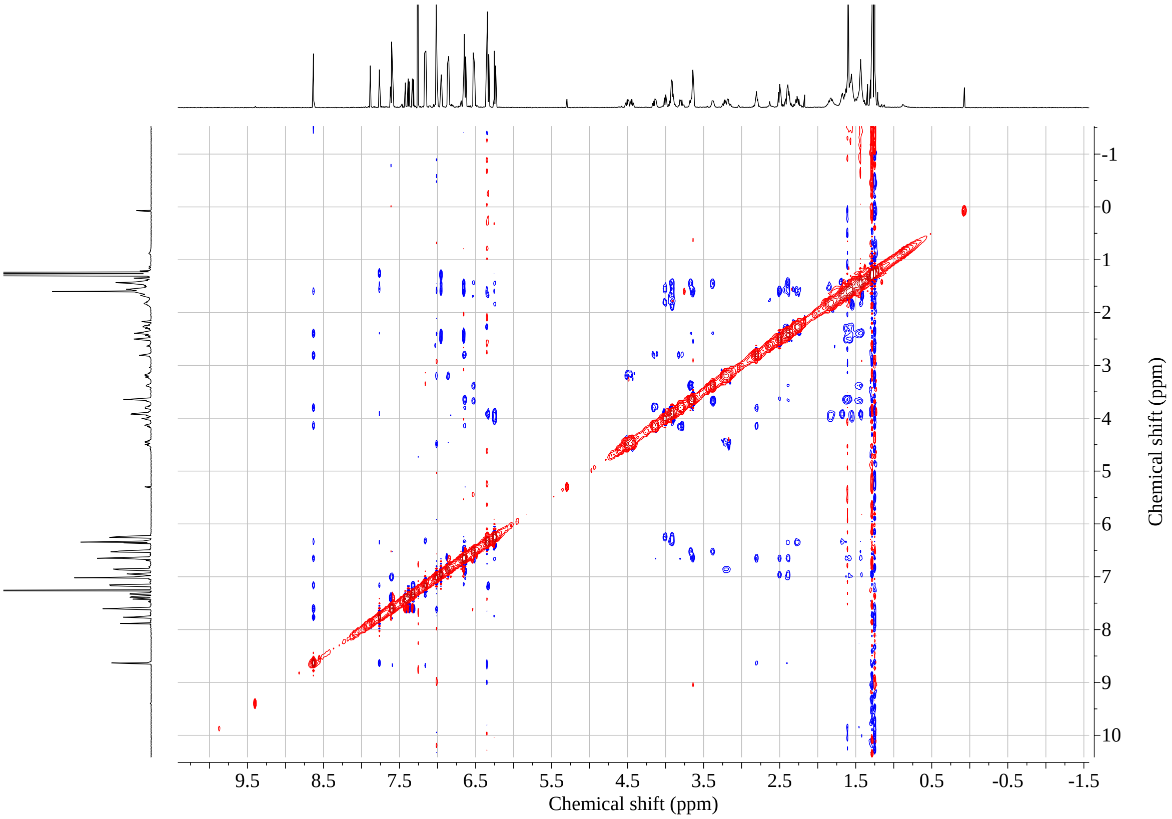

Figure S250. NOESY NMR of *rac*-**9** ($CDCl_3$, 298 K)

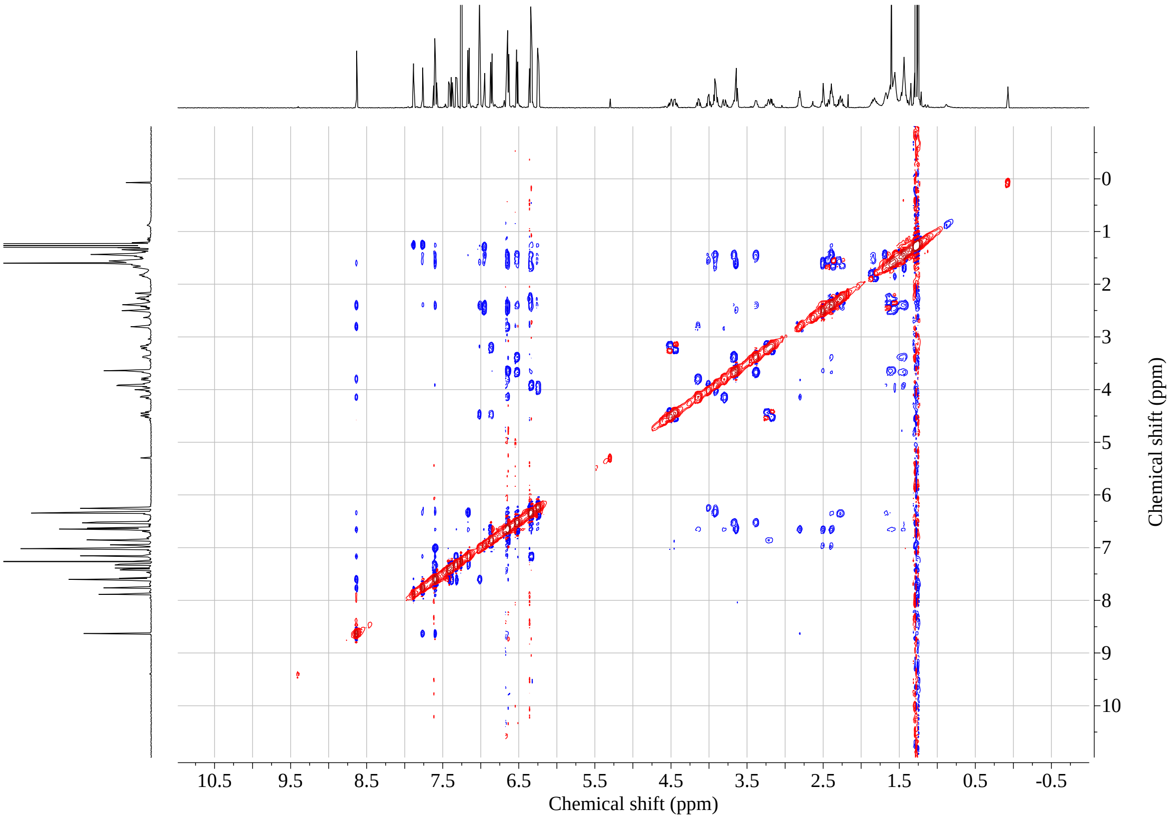

Figure S251. ROESY NMR of *rac*-**9** ($CDCl_3$, 298 K)

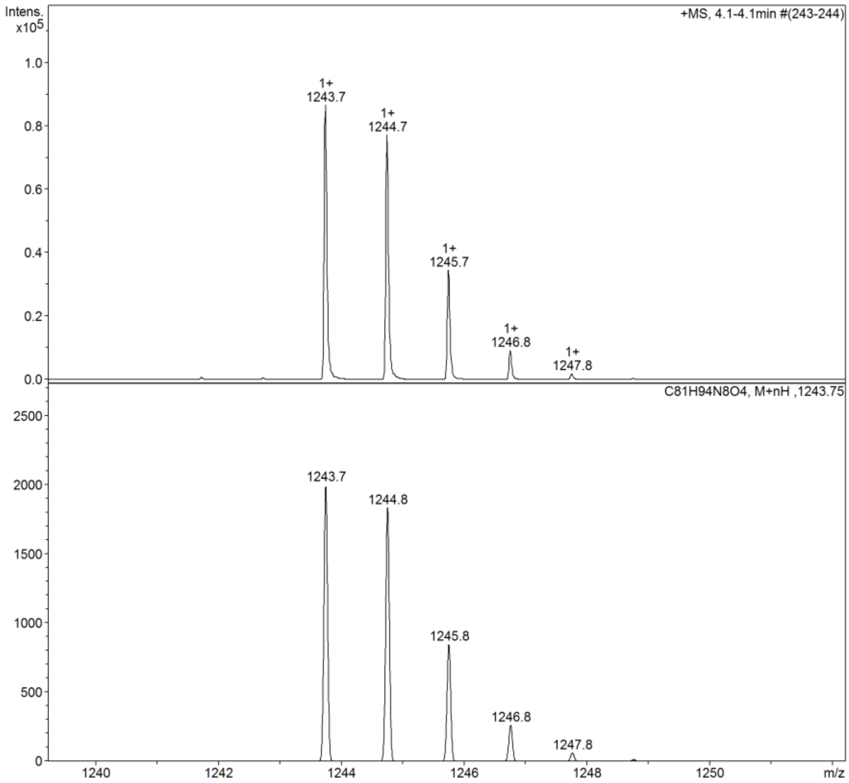

Figure S252. Isotope pattern of *rac*-**9** $C_{81}H_{94}N_8O_4$

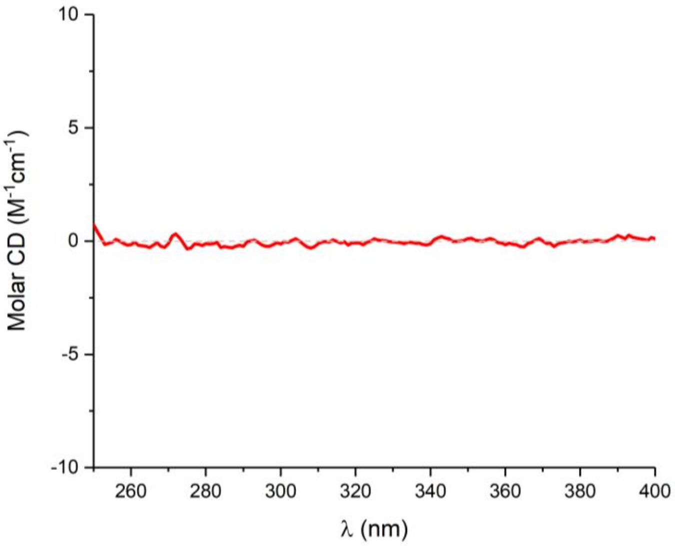

Figure S253. Circular dichroism spectrum of *rac*-**9** (14.9 µM) at 293 K in $CHCl_3$

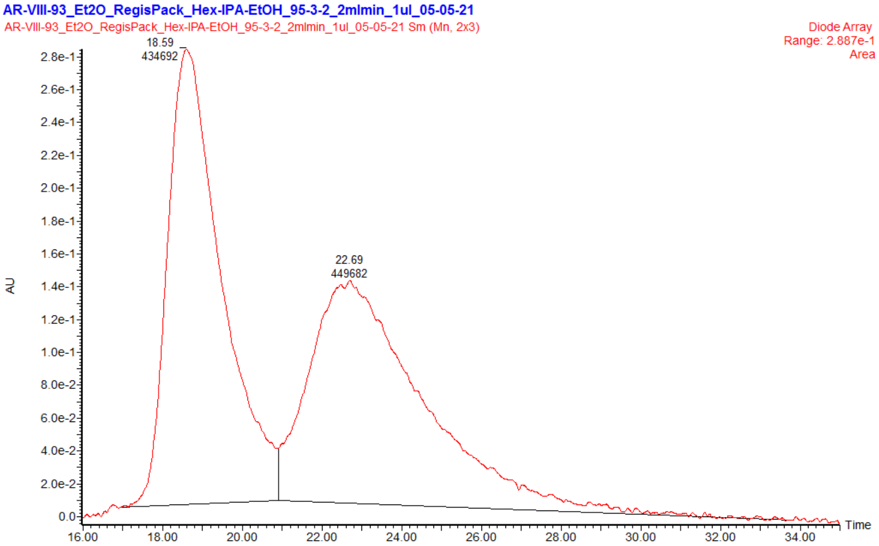

Figure S254. CSP-HPLC of *rac*-**9** (loaded in Et_2O). RegisPack, *n*-hexane-IPA-ethanol 95 : 3 : 2, flowrate 2.0 mLmin^{-1}, retention times *rac*-**9**, (R_{mt})-**9** (18.6 min, 434692, 49.2%), (S_{mt})-**9** (22.7 min, 449682, 50.8%).

Catenane (S_{co-mt})-9

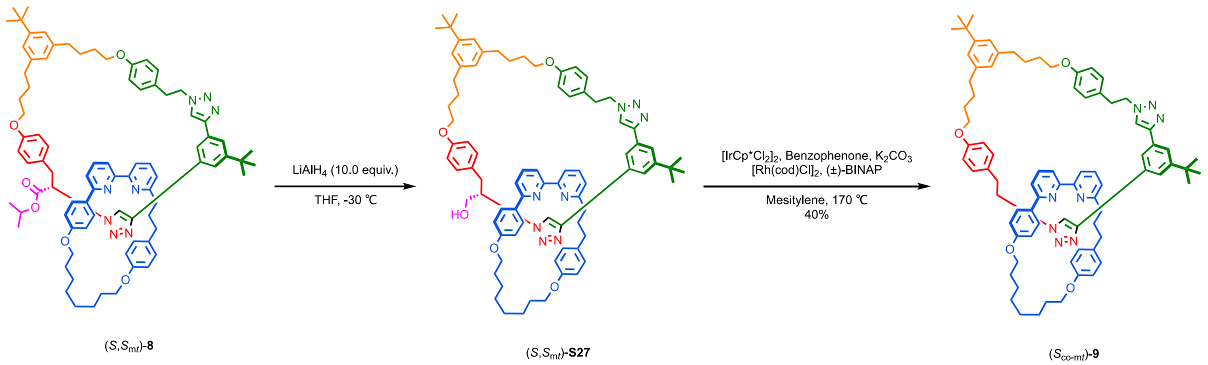

A solution of (S,S_{mt})-**8** (16 mg, 0.012 mmol, 1.0 equiv.) in degassed THF (0.12 mL) was cooled to -30 ºC in a solid CO_2-acetone bath. $LiAlH_4$ (1 M solution in THF, 0.1 mL, 0.1 mmol, 10.0 equiv.) was added dropwise along the wall of the reaction vessel and reaction mixture stirred at -30 ºC for 1 h. MeOH was added slowly along the wall of the vessel and the resulting mixture allowed to warm to rt. Sat. Rochelle salt solution (5 mL) was added and the resulting mixture was extracted with EtOAc (20 mL). The organic phase was dried ($MgSO_4$) and the solvent removed in vacuo to give a yellow oil crude (16 mg) containing catenane (S,S_{mt})-**S27** that was used without further purification.

$[IrCp^*Cl_2]_2$ (10 mg, 0.012 mmol, 1.0 equiv.), benzophenone (7 mg, 0.037 mmol, 3.0 equiv.), $[Rh(cod)Cl]_2$ (0.6 mg, 0.0012 mmol, 0.1 equiv.), (±)-BINAP (3 mg, 0.0050 mmol, 0.4 equiv.) and K_2CO_3 (7 mg, 0.050 mmol, 4.0 equiv.) and mesitylene (1.2 mL) were added to the crude. The resulting suspension was degassed by bubbling N_2 for 5 min and then heated with stirring to 170 ºC for 6 h, at which point TLC analysis indicated that the starting material had been consumed. The solution was allowed to cool to rt and chromatographed directly (hexane-acetone 90:10 → 70:30) to obtain enantioenriched catenane (S_{co-mt})-**9** as a yellow oil (6 mg, 40% yield over two steps, 87% *ee*). Analytical data were identical to those reported for *rac*-**9** with the exception of the circular dichroism spectra and CSP-HPLC. Heating (S_{co-mt})-**9** for 24 h at 170 °C in mesitylene did not cause any loss of stereopurity, highlighting the kinetic stability of the stereogenic unit (Figure S260).

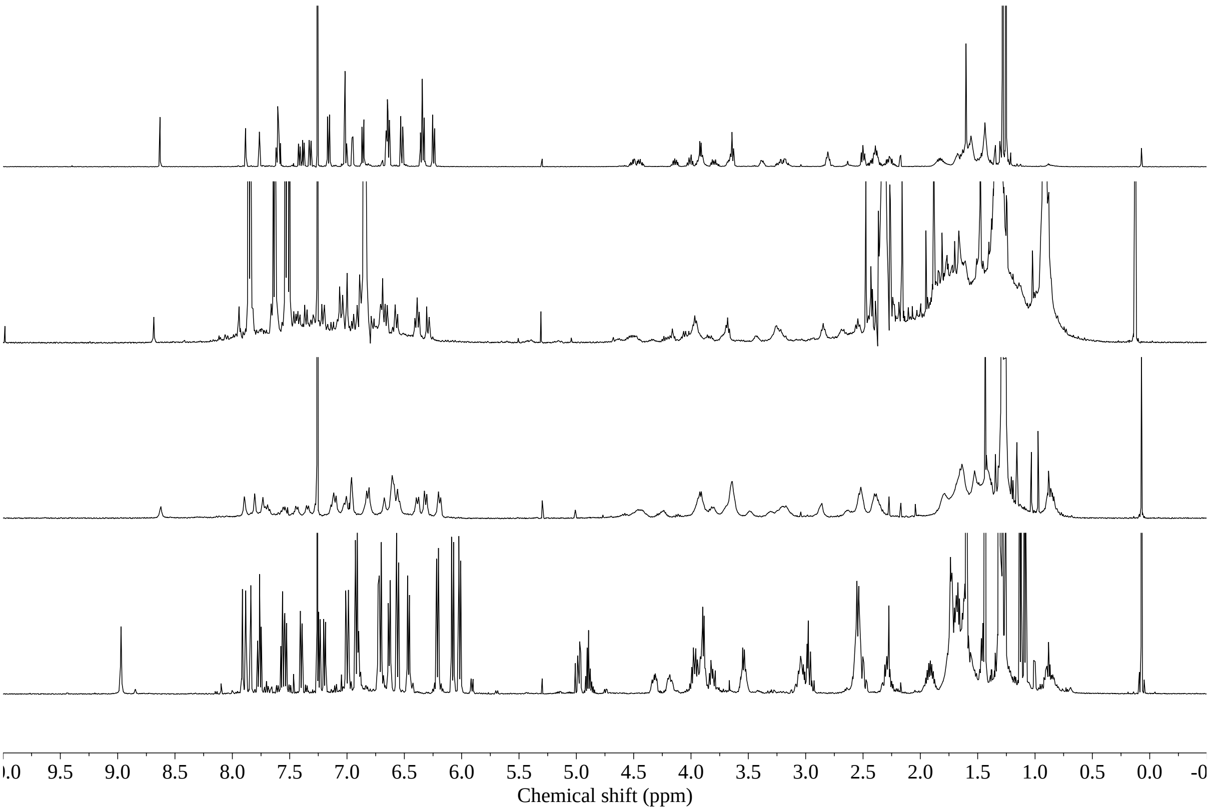

Figure S255. Stacked ^{1}H NMR of (*S*,*S*$_{mt}$)-**8** (bottom), crude (*S*,*S*$_{mt}$)-**S27** (second from bottom), crude (*S*$_{co\text{-}mt}$)-**9** (second from top) and *rac*-**9** (top) ($CDCl_3$, 400 MHz, 298 K)

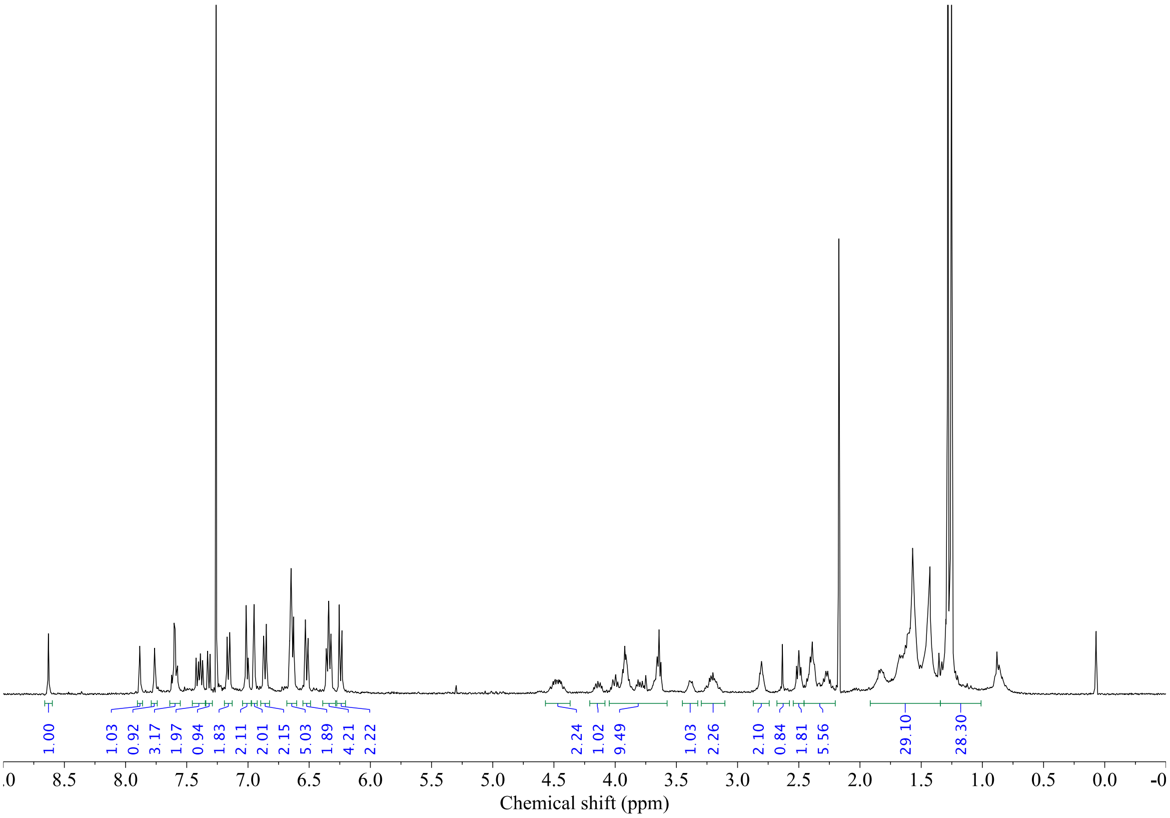

Figure S256. ^{1}H NMR of (*S*$_{co\text{-}mt}$)-**9** ($CDCl_3$, 400 MHz, 298 K)

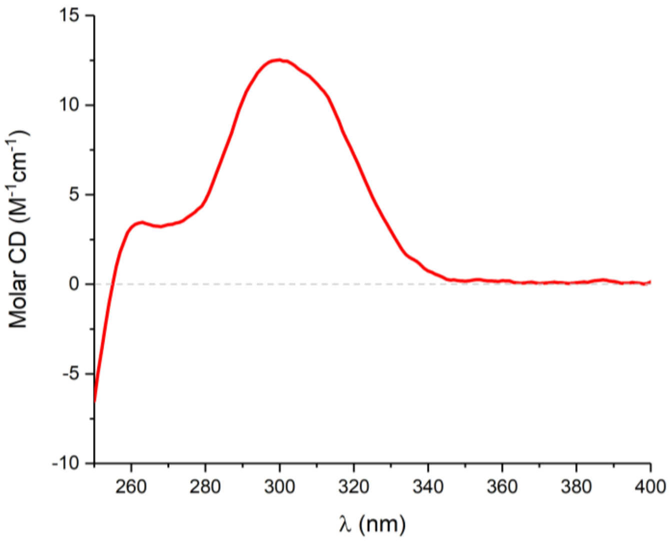

Figure S257. Circular Dichroism Spectra of ($S_{co\text{-}mt}$)-**9** (19.8 μM, *e.r.* ($S_{co\text{-}mt}$)-**9** : ($R_{co\text{-}mt}$)-**9** 93 : 7) at 293 K in $CHCl_3$

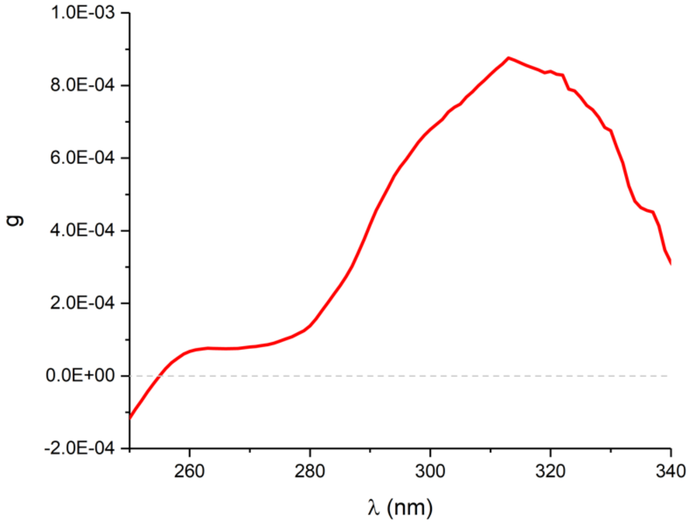

Figure S258. g_{abs} plot of ($S_{co\text{-}mt}$)-**9** (19.8 μM, *e.r.* ($S_{co\text{-}mt}$)-**9** : ($R_{co\text{-}mt}$)-**9** 93 : 7) at 293 K in $CHCl_3$. g_{abs}(max) = 8.8 × 10^{-4} (313 nm).

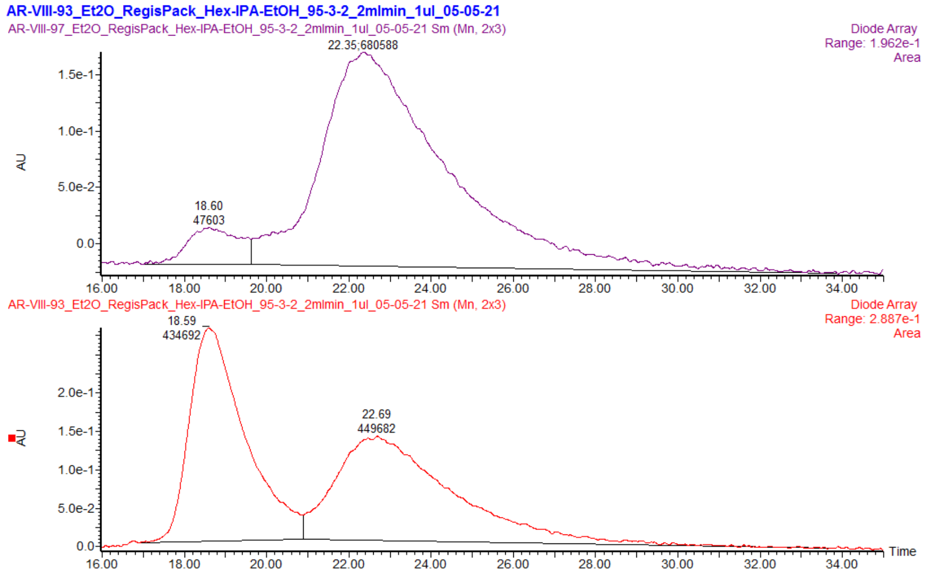

Figure S259. CSP-HPLC of ($S_{co\text{-}mt}$)-**9** (loaded in Et_2O). RegisPack, *n*-hexane-IPA-ethanol 95 : 3 : 2, flowrate 2.0 mLmin^{-1}, retention times ($R_{co\text{-}mt}$)-**9** (18.6 min, 47603, 6.5%), ($S_{co\text{-}mt}$)-**9** (22.3 min, 680588, 93.5%).

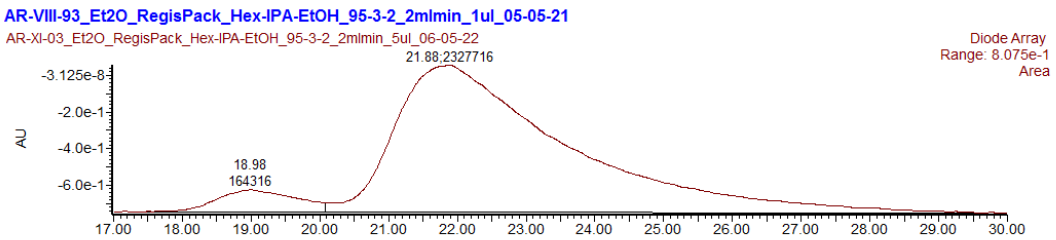

Figure S260. CSP-HPLC of ($S_{co\text{-}mt}$)-**9** (87% *ee*) after heating at 170 ºC for 16h. Loaded in Et_2O, RegisPack, *n*-hexane-IPA-ethanol 95 : 3 : 2, flowrate 2.0 mLmin^{-1}, retention times ($R_{co\text{-}mt}$)-**9** (19.0 min, 164316, 6.6%), ($S_{co\text{-}mt}$)-**9** (21.9 min, 2327716, 93.4%).

S5. Model compounds for auxiliary cleavage study

Alcohol (*S*)-S29

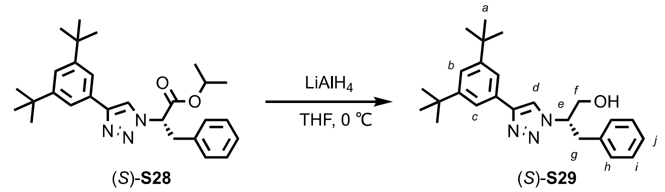

(*S*)-**S28** (*S*)-**S29**

(*S*)-**S28** (40 mg, 0.09 mmol, 1.0 equiv.) was dissolved in dry THF (0.9 mL) and cooled down to 0 °C in an ice bath. $LiAlH_4$ (1M in THF, 0.22 mL, 2.5 equiv.) was added at 0 °C and the reaction mixture was stirred at rt for 1 h, when TLC analysis showed full conversion of the starting material. The reaction mixture was diluted with 1 mL of Et_2O and quenched by adding 0.2 mL of water at 0 °C. Then, 0.2 mL of NaOH 15% (w/v) were added, followed by 3 x 0.2 mL of water. The crude reaction mixture was dried over $MgSO_4$, the solvent was removed *in vacuo* and (*S*)-**S29** was obtained as a white foam (35 mg, 99% yield) and used without further purification.

^{1}H NMR (400 MHz, $CDCl_3$, 298 K) δ 7.62-7.55 (m, 3H, H_c, H_d), 7.42 (t, *J* = 1.8 Hz, 1H, H_b), 7.31-7.20 (m, 3H, H_i, H_j), 7.13-7.05 (m, 2H, H_h), 4.85-4.67 (m, 1H, H_e), 4.11 (d, *J* = 4.2 Hz, 2H, H_f), 3.30 (d, *J* = 7.5 Hz, 2H, H_g), 1.35 (s, 18H, H_a).

^{13}C NMR (101 MHz, $CDCl_3$, 298 K) δ 151.6, 147.5 (from HMBC), 136.5, 129.2, 129.0, 128.9, 127.3, 123.0, 120.8, 120.5, 65.5, 63.9, 38.0, 35.1, 31.6.

HR-ESI-MS *m/z* = 414.2517 $[M+Na]^+$ calc. 414.2516 for $C_{25}H_{33}N_3NaO$.

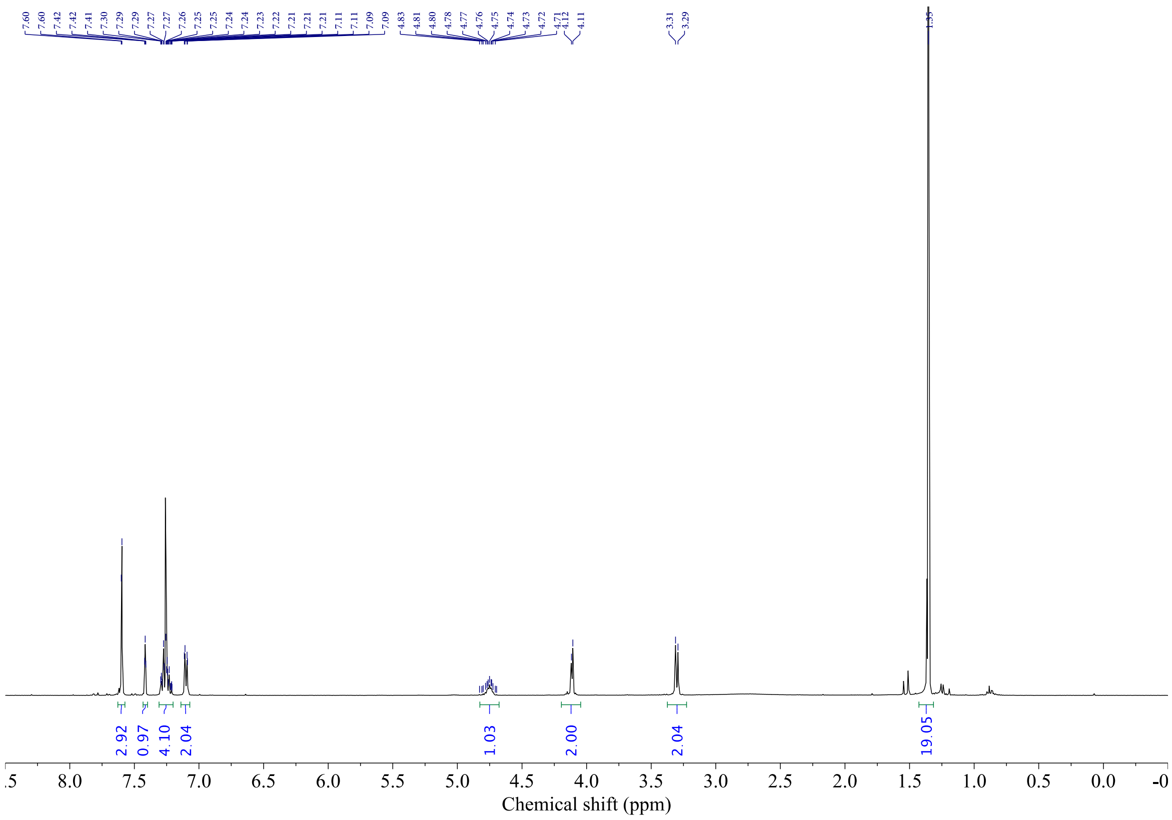

Figure S261. ^{1}H NMR of (*S*)-**S29** ($CDCl_3$, 400 MHz, 298 K).

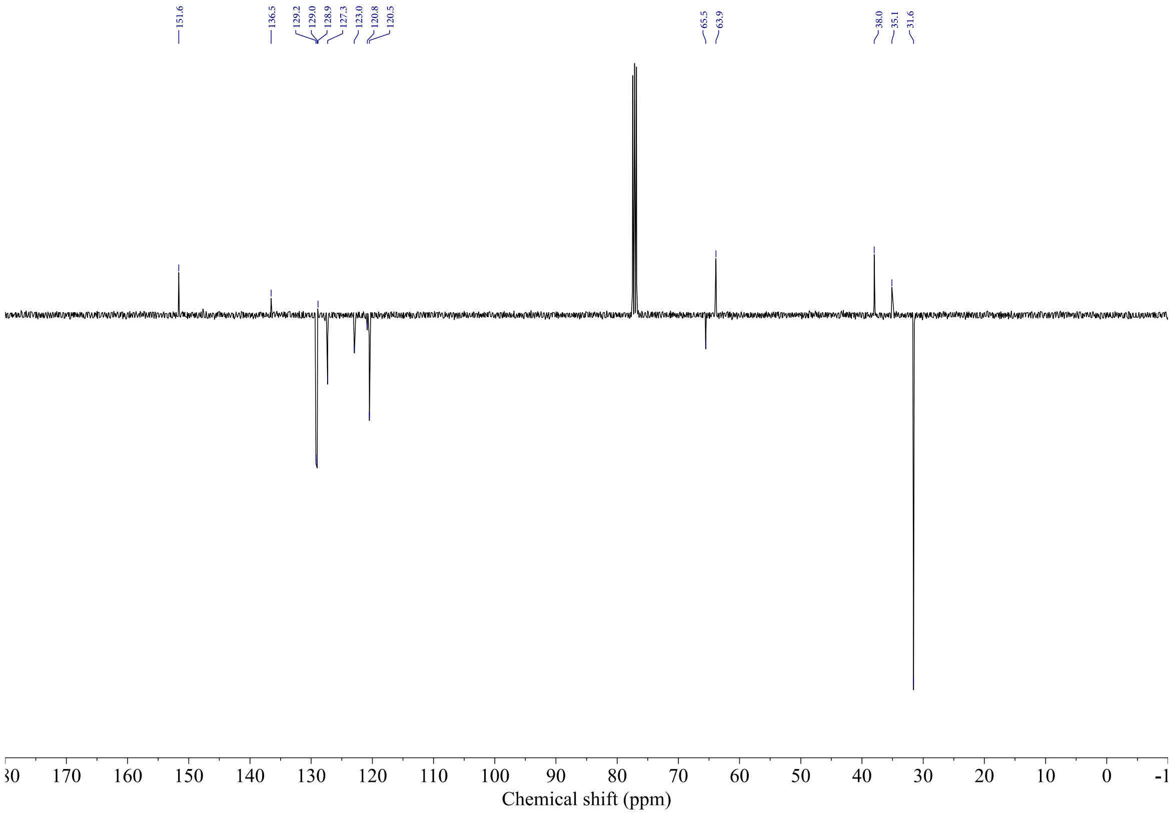

Figure S262. JMOD NMR of (*S*)-**S29** ($CDCl_3$, 400 MHz, 298 K)

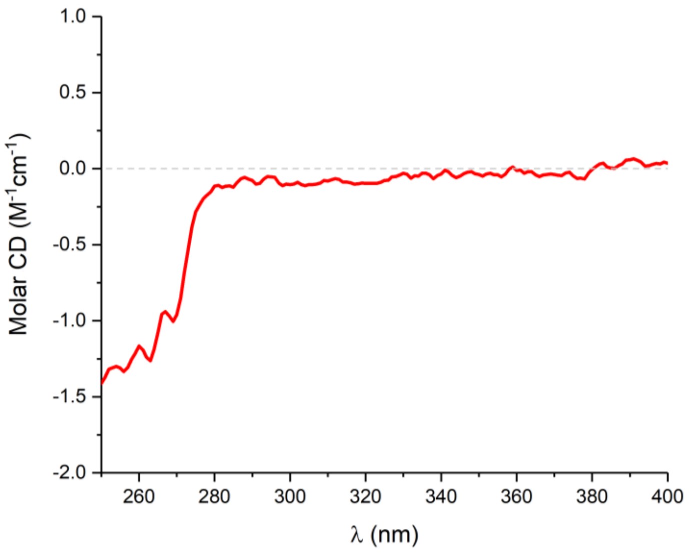

Figure S263. Circular Dichroism Spectra of (*S*)-**S29** (100 µM) at 293 K in $CHCl_3$

Aldehyde (*S*)-S30

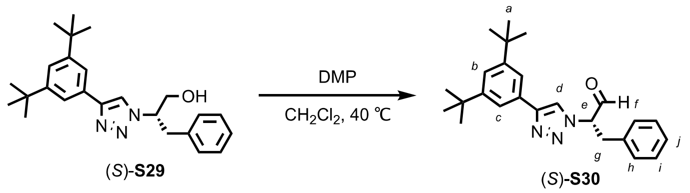

(*S*)-**S29** (34 mg, 0.09 mmol, 1.0 equiv.) and DMP (55 mg, 0.13 mmol, 1.5 equiv.) were dissolved in CH_2Cl_2 (1 mL) and heated at 40 °C over 16 h. The reaction mixture was filtered over Celite® and the crude reaction mixture was purified by column chromatography (petrol-EtOAc 100 : 0 → 50 : 50) gave (*S*)-**S30** as a colourless oil (25 mg, 73%).

^{1}H NMR (400 MHz, $CDCl_3$, 298 K) δ 9.88 (s, 1H, H_f), 7.60 (d, *J* = 1.8 Hz, 2H, H_c), 7.52 (s, 1H, H_d), 7.43 (t, *J* = 1.8 Hz, 1H, H_b), 7.31-7.19 (m, 3H, H_i, H_j), 7.08-7.00 (m, 2H, H_h), 5.34 (dd, *J* = 9.5, 5.2 Hz, 1H, H_e), 3.61 (dd, *J* = 14.3, 5.2 Hz, 1H, H_g), 3.44 (dd, *J* = 14.3, 9.5 Hz, 1H, H_g), 1.36 (s, 18H, H_a).

^{13}C NMR (101 MHz, $CDCl_3$, 298 K) δ 195.0, 151.6, 149.1, 135.0, 129.5, 129.2, 129.1, 127.7, 122.8, 120.4, 120.4, 70.0, 36.8, 35.1, 31.6.

HR-ESI-MS *m/z* = 390.2541 $[M+H]^+$ calc. 390.2540 for $C_{25}H_{32}N_3O$.

$[\alpha]_D^{23}$ –3.8 (*c* 1.40, $CHCl_3$)

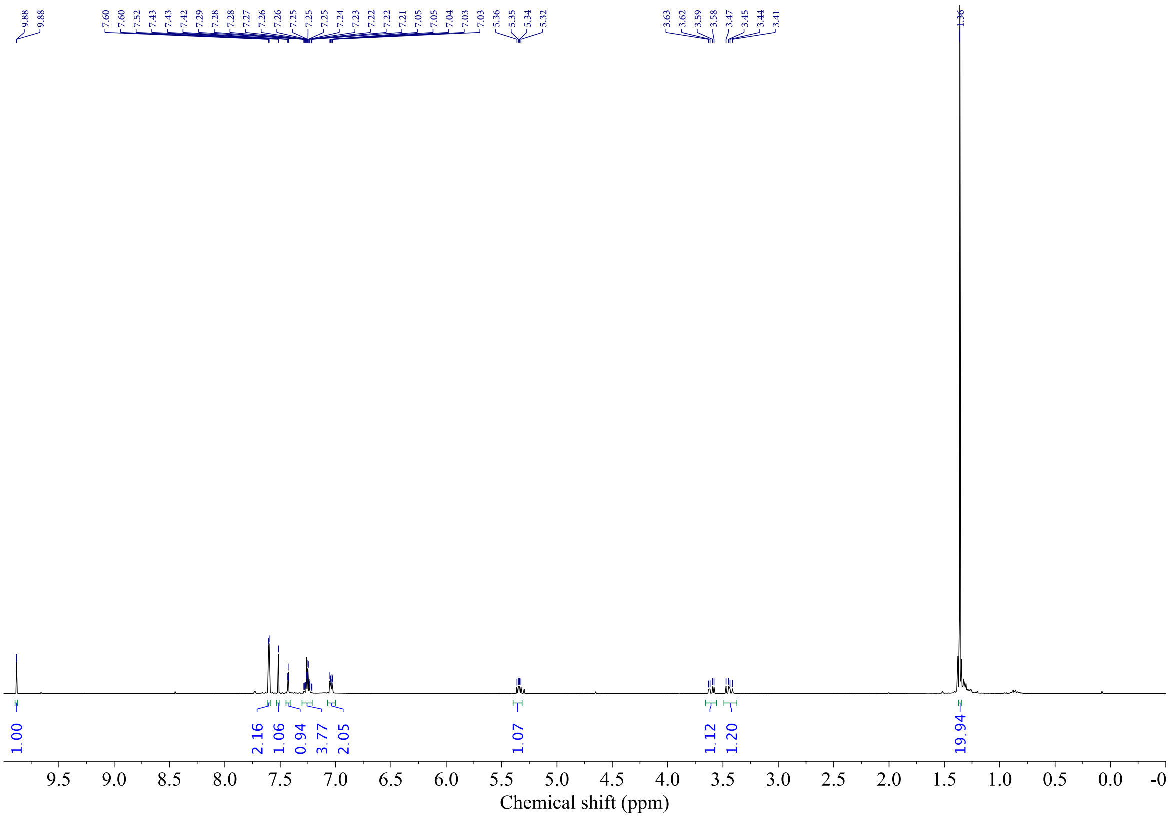

Figure S264. ^{1}H NMR of (*S*)-**S30** ($CDCl_3$, 400 MHz, 298 K)

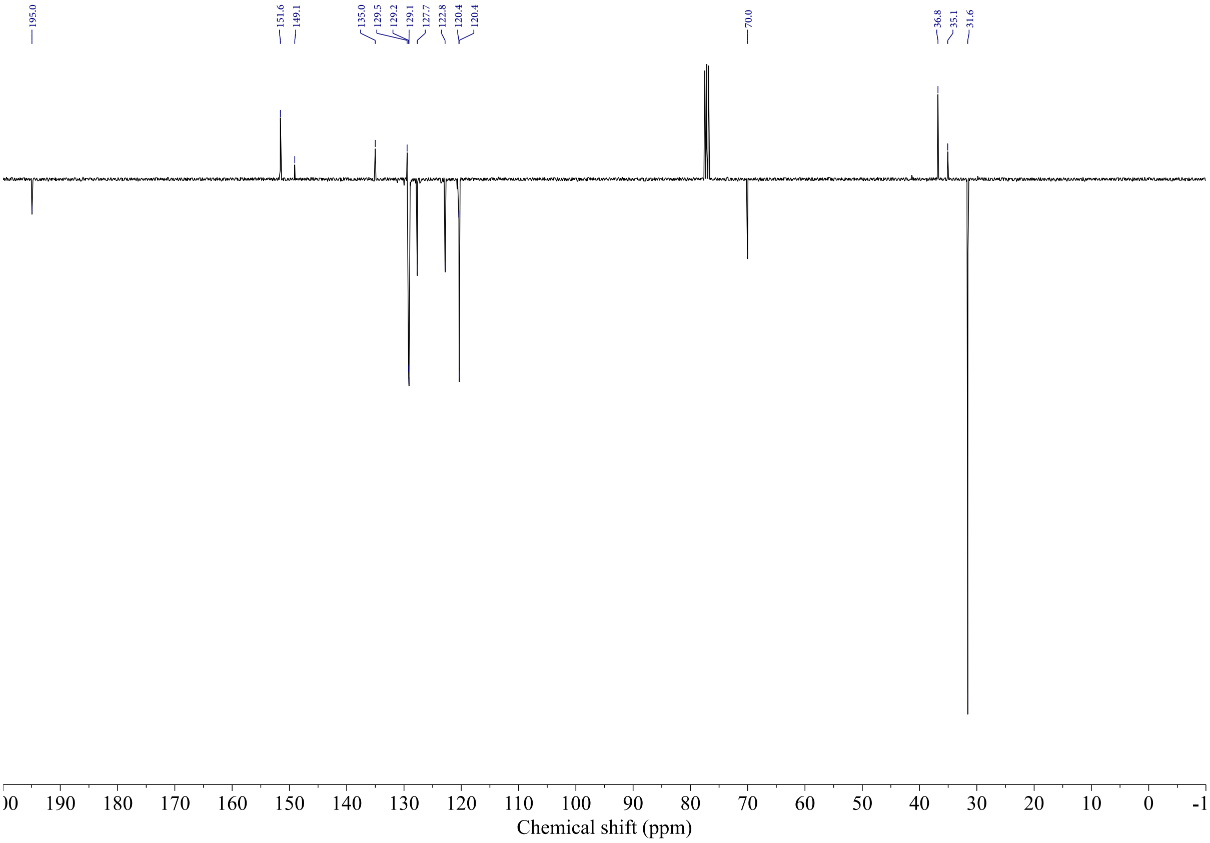

Figure S265. JMOD NMR of (*S*)-**S30** ($CDCl_3$, 400 MHz, 298 K)

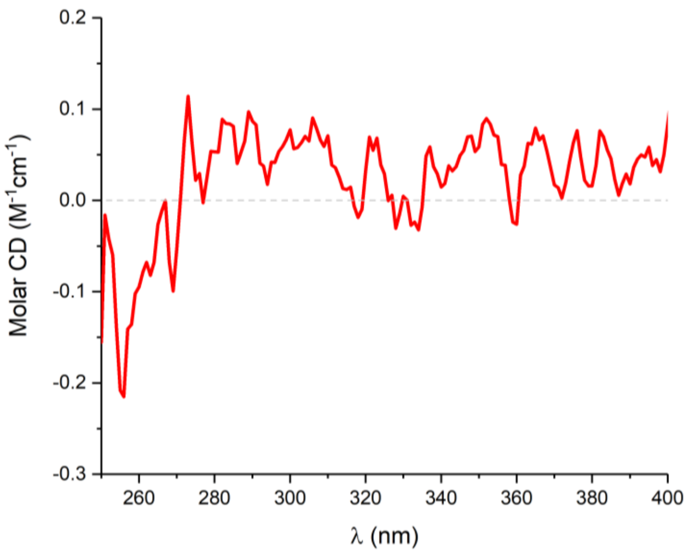

Figure S266. Circular dichroism spectrum of (*S*)-**S30** (64.8 µM) at 293 K in $CHCl_3$

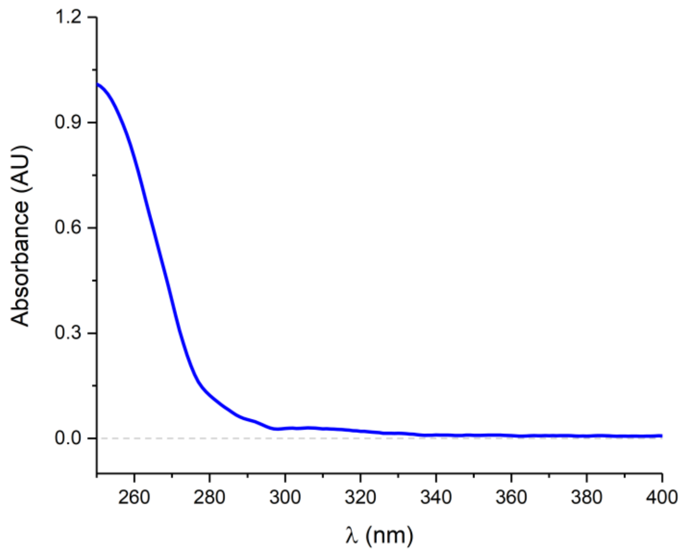

Figure S267. UV-Vis Spectra of (*S*)-**S30** (64.8 µM) at 293 K in $CHCl_3$

Carboxylic acid (*S*)-S31

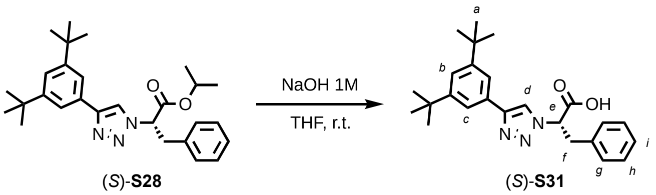

(*S*)-**S28** (47 mg, 0.1 mmol, 1.0 equiv.) was dissolved in THF (0.5 mL) and NaOH (1M, 0.5 mL) was added. The reaction mixture was stirred at rt for 1 h, when TLC analysis showed full conversion of the starting material. The aqueous solution was acidified with citric acid (0.1 M, 5 mL), extracted with 5 mL of CH_2Cl_2 twice and dried over $MgSO_4$. The solvent was removed *in vacuo* and (*S*)-**S31** was obtained as a white foam (40 mg, 93% yield) and used without further purification.

^{1}H NMR (400 MHz, $CDCl_3$, 298 K) δ 7.78 (s, 1H, H_d), 7.58 (d, *J* = 1.8 Hz, 2H, H_c), 7.42 (t, *J* = 1.8 Hz, 1H, H_b), 7.25-7.16 (m, 3H, H_h, H_i), 7.09-6.99 (m, 2H, H_g), 5.71 (dd, *J* = 8.2, 6.1 Hz, 1H, H_e), 3.74-3.46 (m, 2H, H_f), 1.34 (s, 18H, H_a).

^{13}C NMR (101 MHz, $CDCl_3$, 298 K) δ 170.3, 151.7, 148.2, 134.8, 129.2, 129.0, 128.6, 127.8, 123.1, 120.7, 120.6, 64.6, 38.9, 35.1, 31.6.

HR-ESI-MS *m/z* = 406.2496 $[M+H]^+$ calc. 406.2489 for $C_{25}H_{32}N_3O_2$.

$[\alpha]_D^{23}$ −1.2 (*c* 1.00, $CHCl_3$)

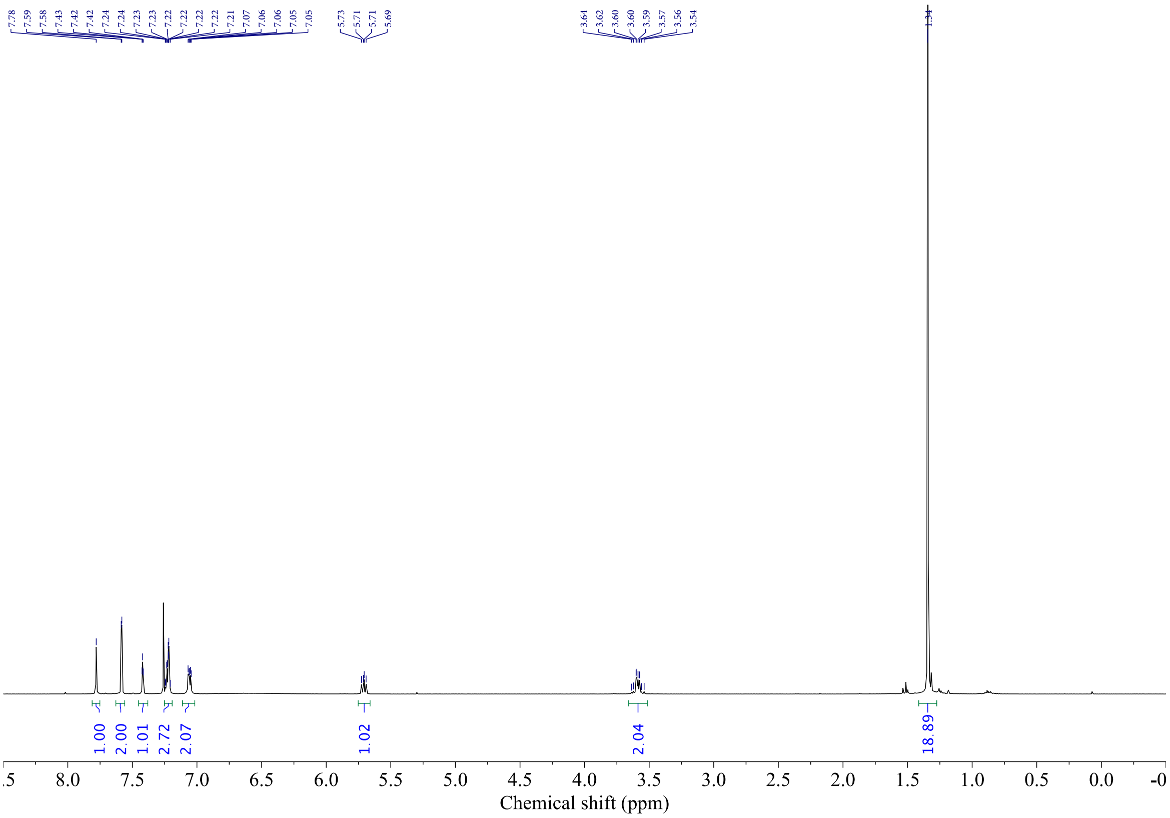

Figure S268. ^{1}H NMR of (*S*)-**S31** ($CDCl_3$, 400 MHz, 298 K)

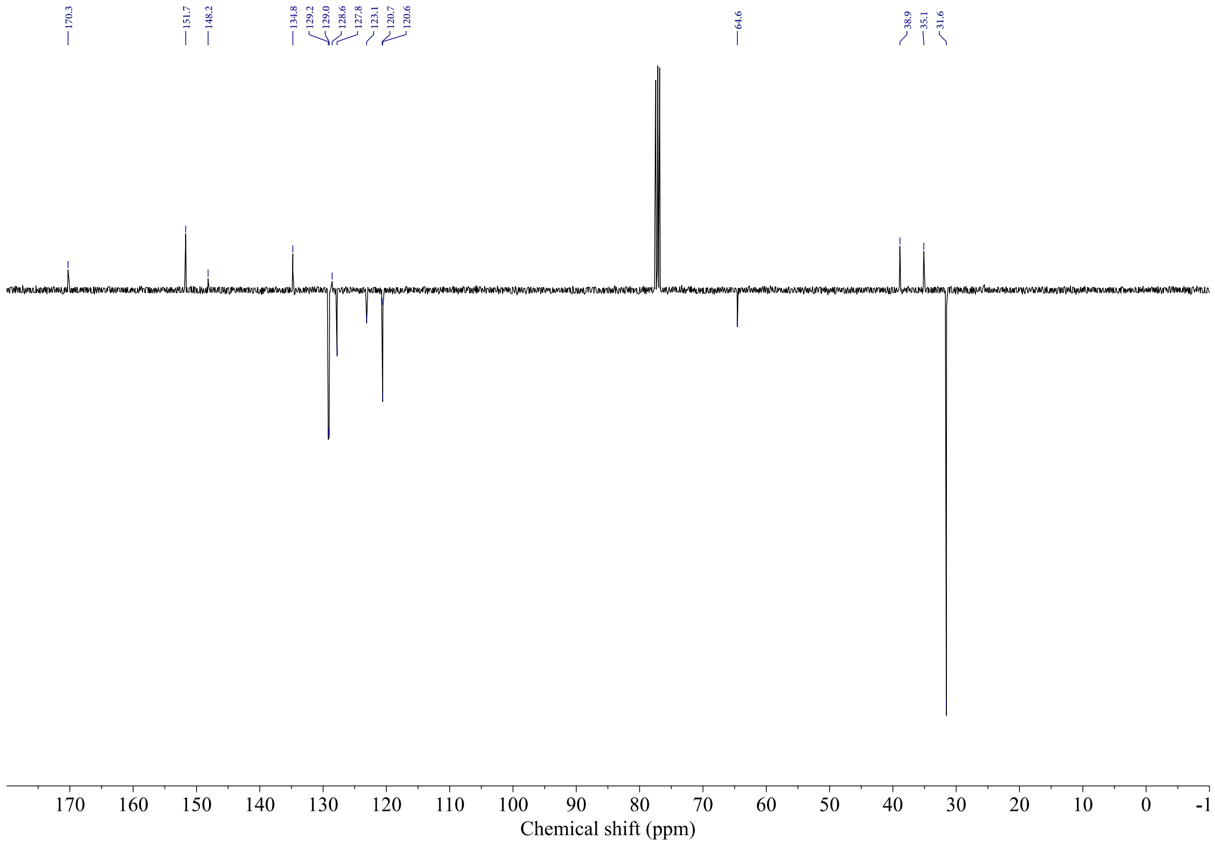

Figure S269. JMOD NMR of (*S*)-**S31** ($CDCl_3$, 400 MHz, 298 K)

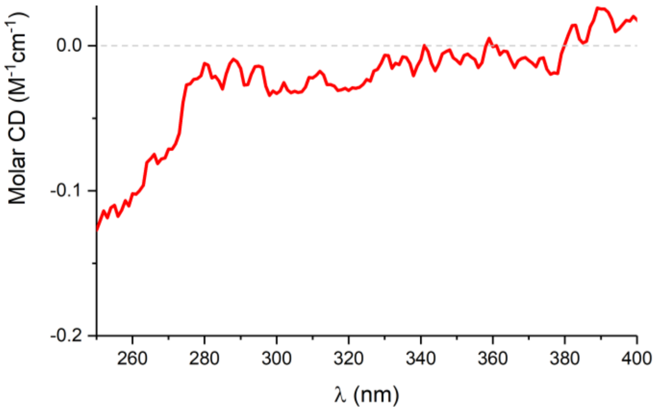

Figure S270. Circular Dichroism Spectra of (*S*)-**S31** (28.8 µM) at 293 K in $CHCl_3$

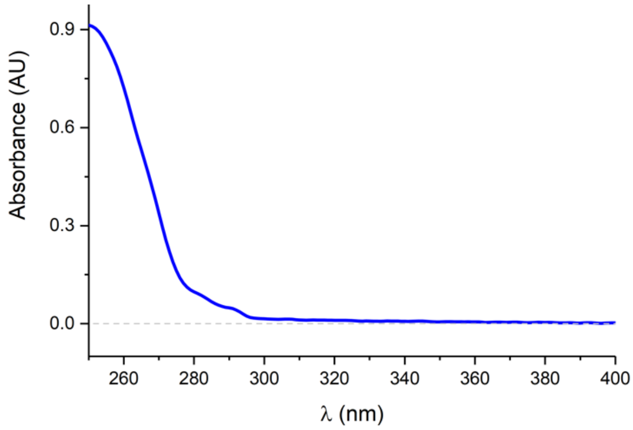

Figure S271. UV-Vis Spectra of (*S*)-**S31** (28.8 µM) at 293 K in $CHCl_3$

Axle S34

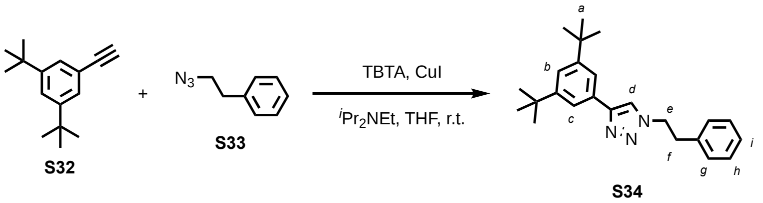

S32 (332 mg, 1.55 mmol, 1.0 equiv.), **S33** (228 mg, 1.55 mmol, 1.0 equiv.), CuI (15 mg, 0.1 mmol, 0.05 equiv.) and TBTA (164 mg, 0.31 mmol, 0.2 equiv.) were dissolved in degassed THF (15 mL). $^{i}Pr_2NEt$ (0.5 mL, 3.0 mmol, 2.0 eq.) was added and the reaction mixture was stirred at rt for 16 h. The solvent was removed *in vacuo* and the residue was purified by column chromatography (petrol-Et_2O 90 : 10 → 50 : 50) gave **S34** as a yellow foam (328 mg, 59%).

^{1}H NMR (400 MHz, $CDCl_3$, 298 K) δ: 7.61 (d, *J* = 1.8 Hz, 2H, H_c), 7.47 (s, 1H, H_d), 7.41 (t, *J* = 1.9 Hz, 1H, H_b), 7.35-7.23 (m, 3H, H_h, H_i), 7.17-7.07 (m, 2H, H_g), 4.64 (t, *J* = 7.2, 2H, H_e), 3.27 (t, *J* = 7.2 Hz, 2H, H_f), 1.36 (s, 18H, H_a).

^{13}C NMR (101 MHz, $CDCl_3$, 298 K) δ: 151.5, 148.3 (from HMBC), 137.2, 129.0, 128.9, 127.3, 122.7, 120.4, 120.1, 52.0, 37.0, 31.6.

HR-ESI-MS *m/z* = 384.2413 $[M+Na]^+$ calc. 384.2410 for $C_{24}H_{31}N_3Na$.

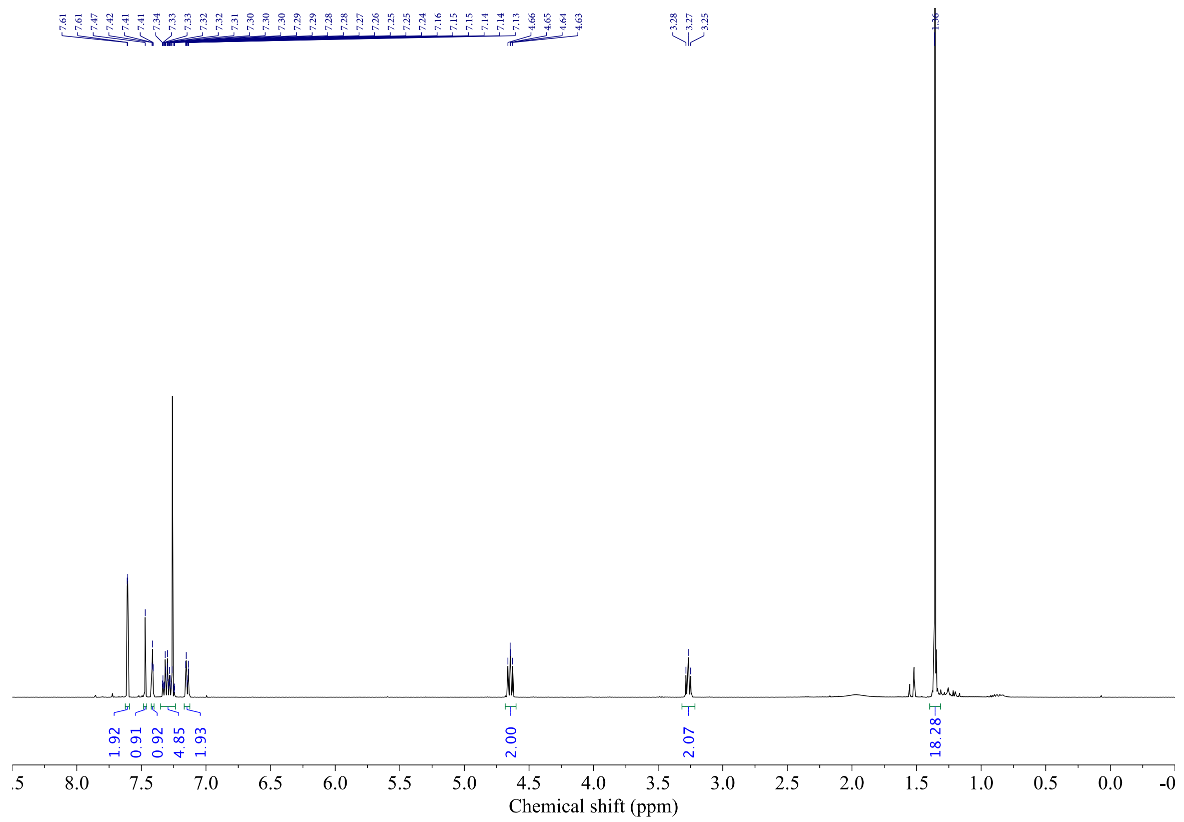

Figure S272. ^{1}H NMR of **S34** ($CDCl_3$, 400 MHz, 298 K)

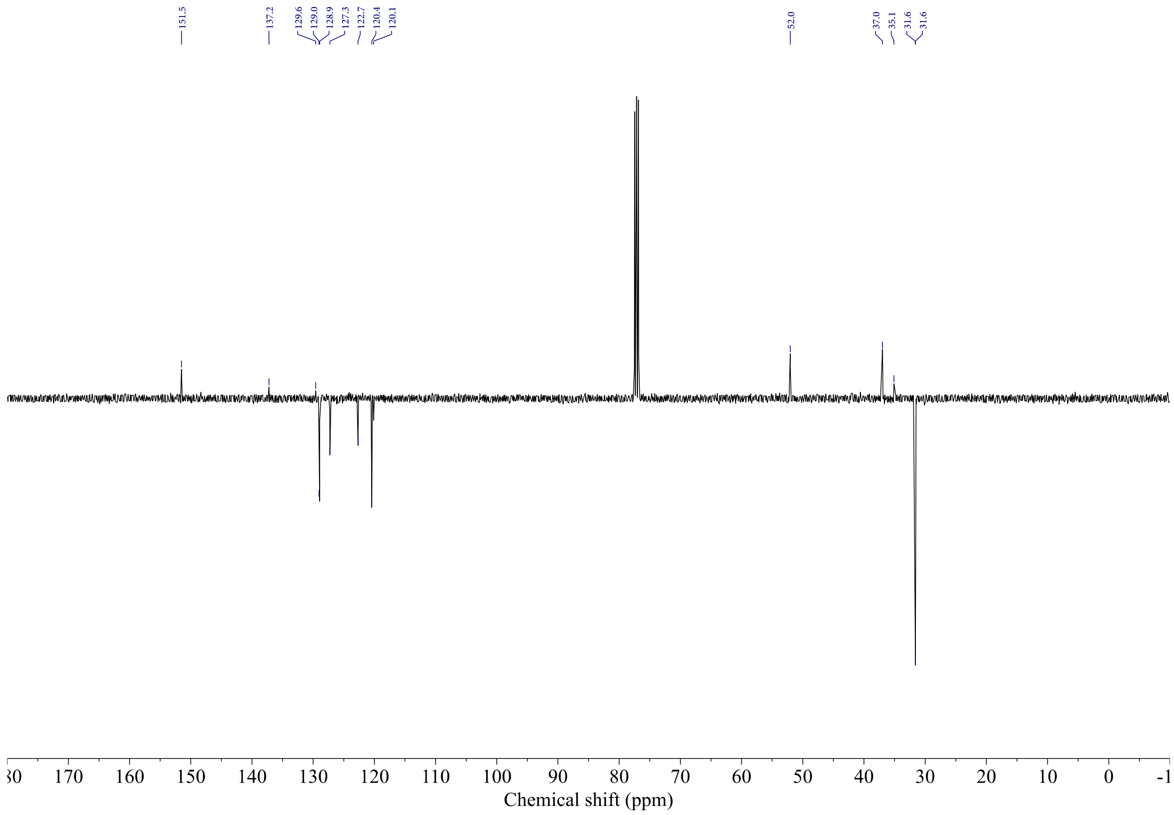

Figure S273. JMOD NMR of **S34** ($CDCl_3$, 400 MHz, 298 K)

S6. Assignment of Stereochemistry for Catenanes 3, 6, 4, S35, 8 and 9

Proposed method for assigning stereochemistry in topologically chiral catenanes

There are no IUPAC guidelines for the assignment of the absolute stereochemistry of topologically chiral catenanes. However, we have previously proposed a general method, which is applied here:

1) Following to the Cahn-Ingold-Prelog (CIP) rules, identify the highest priority atom on one ring and label it as "A"

2) Moving outward from **A** determine the highest priority atom (CIP) that can be used to define an orientation of the ring and label it as "**B**". The orientation of the ring is defined by the vector A → B.

3) Repeat the process on the second ring to identity its orientation.

4) Orient the assembly with the A → B vector of one ring passing through the cavity of the other, directed away from the observer.

5) The direction of the A → B of the second ring defines the stereolabel: clockwise = R_{mt}, counter clockwise = S_{mt}. The "mt" subscript is included to indicate that this stereodescriptor refers to a mechanically topological unit.

Stereochemical assignment of catenanes 3

Using the above rules, catenanes **3** produced in the reaction of macrocycle **2** with pre-macrocycles (*S*)-**1** can be readily assigned. Although there are four possible stereoisomers, because the covalent stereogenic centre is fixed in (*S*)-**1**, catenanes **3** are produced as a pair of mechanical epimers (Figure S274).

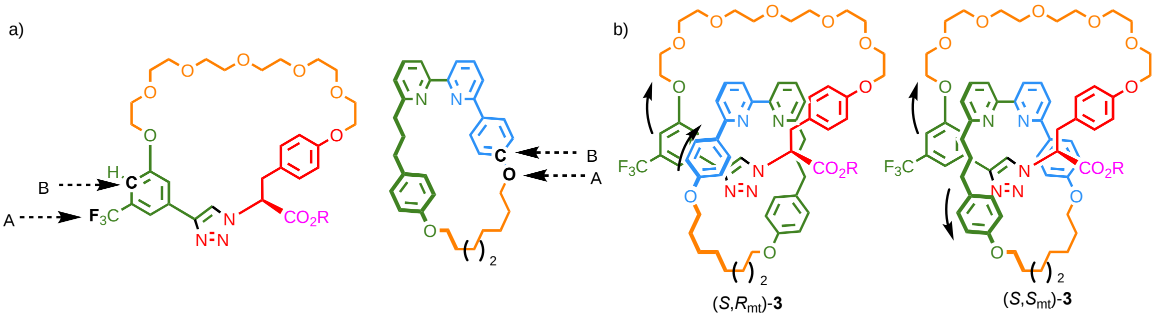

Figure S274. a) Assignment of atom priorities in the interlocked components of catenanes **3**. b) The diastereomeric products formed from the reaction of macrocycle **2** with pre-macrocycles (*S*)-**1** and their stereochemical assignment.

Stereochemical assignment of [2]catenanes S35

[2]Catenanes **S35** are proposed to be by-products from the reaction to form catenanes **3** and arise by initial dimerization of (*S*)-**1** followed by mechanical bond formation. **S35** can form as two isomers, (*S,S,R*$_{mt}$) and (*S,S,S*$_{mt}$). Note that all fluorine atoms are equivalent and so could be labelled as "A" - the same absolute configuration is always obtained.

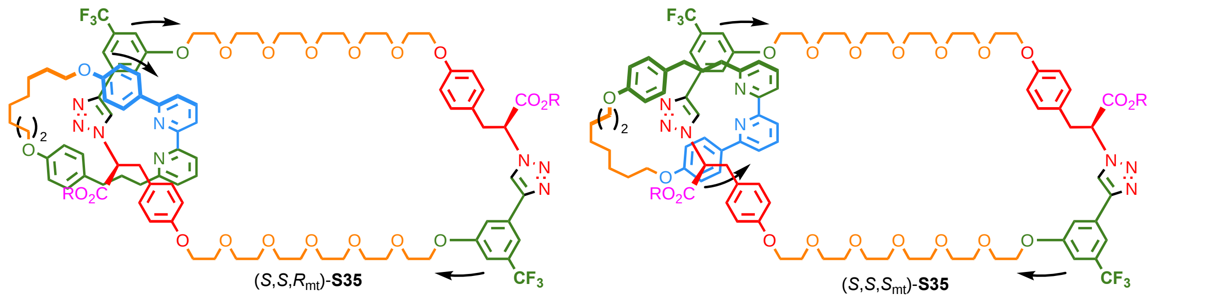

Figure S275. Assignment of stereolabels for dimeric [2]catenane **S35** formed as by-products in the reaction of macrocycle **2** with macrocycle precursor (*S*)-**1**.

Stereochemical assignment of [2]catenanes 4

[3]Catenanes **4** are proposed to be by-products from the reaction to form catenanes **3** and arise by AT-CuAAC dimerization of (*S*)-**1** followed by a second mechanical bond formation. The assignment of [3]catenanes **4** appears more complicated than [2]catenanes **3** and **S35**. However, if each pair of directly interlocked rings are taken in turn, the same process can be applied to arrive at two mechanical stereolabels. Thus, catenanes **4** forms as 3 diastereoisomers: (*S,S,R*$_{mt}$,*R*$_{mt}$), (*S,S,R*$_{mt}$,*S*$_{mt}$) and (*S,S,S*$_{mt}$,*S*$_{mt}$).

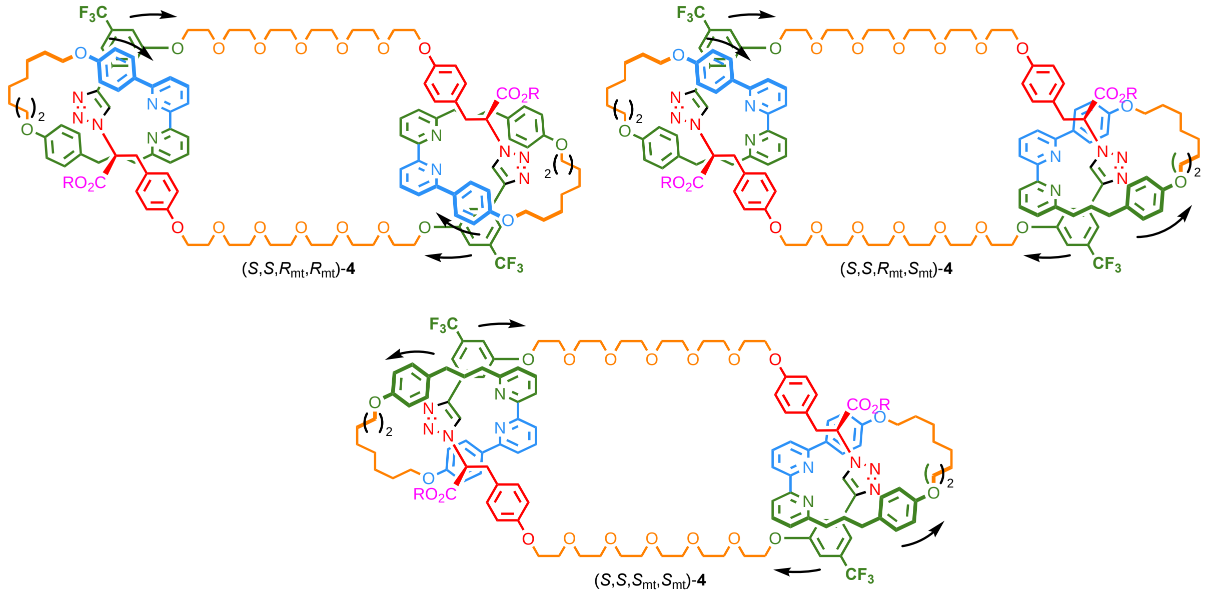

Figure S276. Assignment of stereolabels for [3]catenanes **4**.

Stereochemical assignment of catenane 6

Ablation of the covalent stereogenic unit of catenanes **3** gives rise to catenane **6** in which only the mechanical topological stereogenic unit remains. The relative orientations of the rings do not change in this process and so an unequal mixture of catenanes **3b** gives rise to enantioenriched **6**.

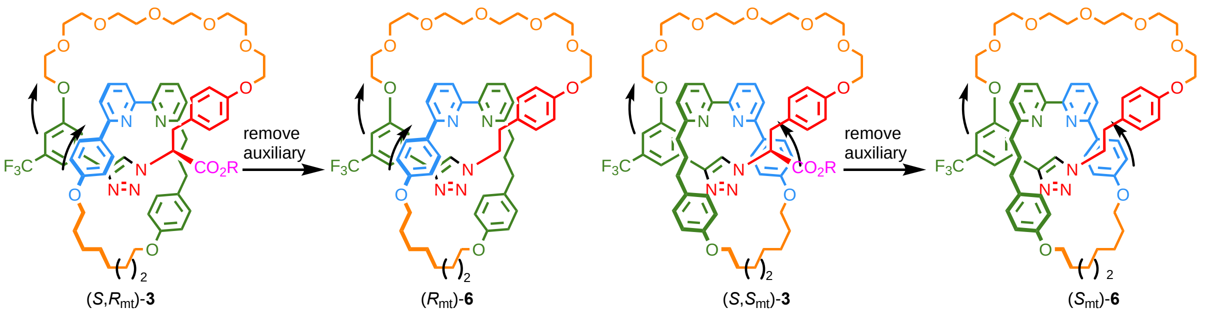

Figure S277. Assignment of stereolabels for catenane **6**.

Stereochemical assignment of catenane 8

The stereochemistry of catenane **8** is assigned as per catenanes **3** with the atom priorities shown (Figure S278a). The major isomer obtained in the case of **8** is assumed to have the same relative orientation of the triazole and macrocycle as **3a** given the similarity of the reacting functional groups and the absolute selectivity obtained. It should be noted that, having identified atom **A**, in this case we must continue exploring as far as the indicated N before it is possible to unambiguously determine the orientation of the macrocycle.

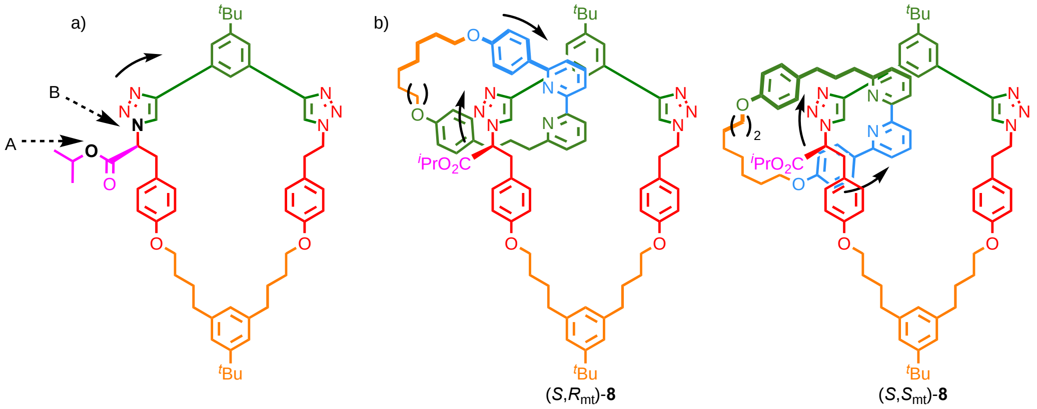

Figure S278. a) Assignment of the orientation of the triazole containing ring of catenane **8**. b) Assignment of stereolabels for the diastereomers of catenane **8** formed from macrocycle precursor (*S*)-**7**.

Stereochemical assignment of catenane 9

The orientation of the triazole containing ring of catenane **9** cannot be determined by applying the previous rules - the covalent structure of the ring is bilaterally symmetric and so the two oxygen atoms are indistinguishable and would give rise to opposite orientations. To determine the orientation of the ring, the encircling bipyridine macrocycle is considered as a "ghost substituent" on the atoms in the region of the triazole macrocycle it encircles. This allows atoms A and B to be assigned and the stereolabel is then

assigned following the rules presented above. The "co-mt" subscript is included to indicate that this stereodescriptor refers to a co-conformationally mechanically “topological” unit.

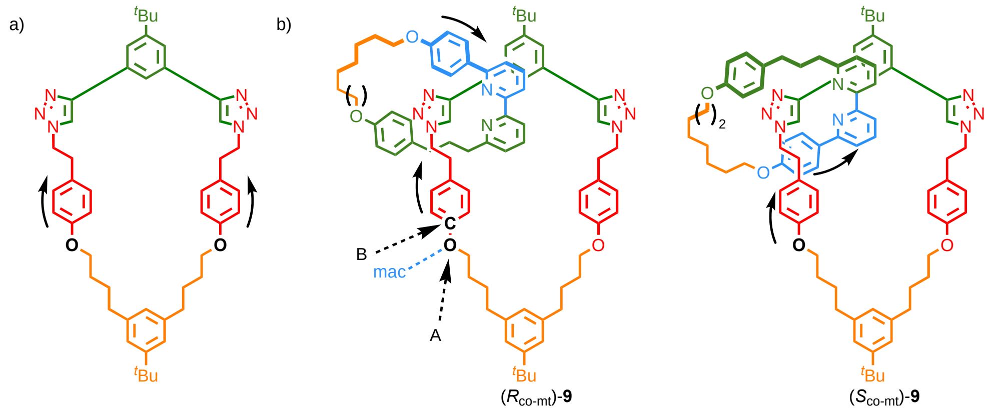

Figure S279. a) Structure of the non-interlocked triazole macrocycle of **9** indicating the oxygen atoms are of equivalent priority. b) Structure and stereochemical assignment of the enantiomers of **9**, which requires the bipyridine ring to be considered as a ghost substituent of the O atom it encircles.

S7. Crystallographic Data

Single Crystal X-ray Diffraction Data for Catenane *rac*-(*S*,*S*$_{mt}$)-3b

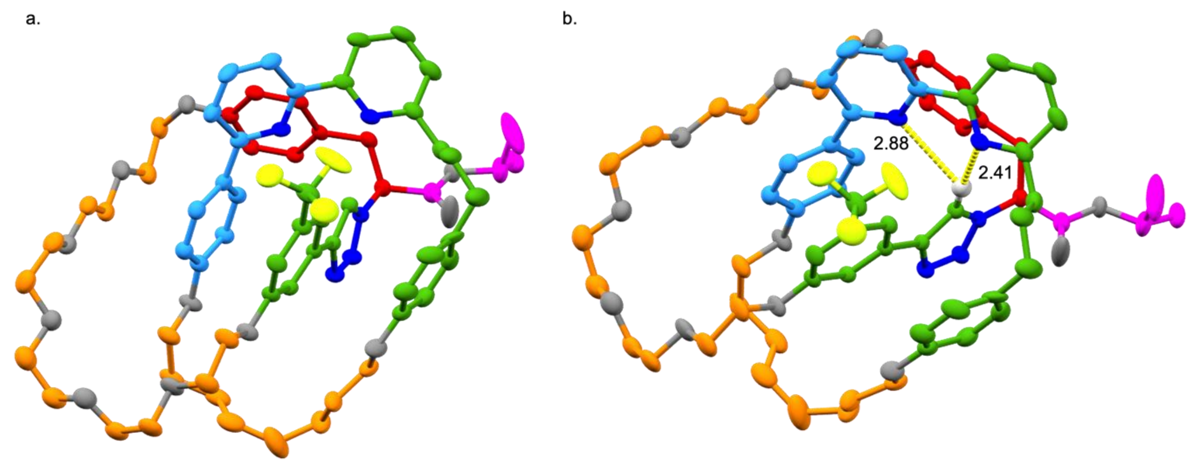

Figure S280. Ellipsoid plot of the asymmetric unit for *rac*-(*S*,*S*$_{mt}$)-**3b** with hydrogens omitted for clarity (a) and key intercomponent interactions highlighted (b). Thermal ellipsoids at 50% probability, disorder omitted for clarity.

Single crystals of *rac*-(*S*,*S*$_{mt}$)-**3b** were grown by slow vapour diffusion of *n*-pentane into a concentrated dichloromethane solution of *rac*-(*S*,*S*$_{mt}$)-**3b**, yielding colourless block crystals. Data was collected at 100 K on a Rigaku 007 HF diffractometer equipped with a HyPix 6000HE hybrid pixel array detector. Cell determination, data reduction, cell refinement, and absorption correction were processed by CrysalisPro[9]. The structure was solved within Olex2[10] by ShelXT[11] with refinement by ShelXL[12]. The asymmetric cell contained one molecule of $C_{66}H_{78}F_3N_5O_{11}$ and disordered solvent. The unit cell contained both enantiomers related by an inversion centre. The disordered solvent was believed to contain primarily two CH_2Cl_2 molecules. Modelling of the disordered solvent molecules was attempted, however, a satisfactory solution was unable to be found, and thus the program SQUEEZE[13] implemented within PLATON[14] was used to account for the electron density within this region of the unit cell. SQUEEZE identified solvent accessible voids of 594 Å^3 and 166 electrons per unit cell were recovered.

Table S1. Single crystal Diffraction and refinement statistics for *rac*-(*S*,*S*$_{mt}$)-**3b**

CCDC Number	2125552	ρ_{calc} g/cm^3	1.122
Empirical Formula	$C_{66}H_{78}F_3N_5O_{11}$	μ/mm^{-1}	0.08
Formula Weight	1174.33	F(000)	1248
Temperature/K	100	Crystal size/mm^3	0.19 × 0.14 × 0.08
Crystal System	Triclinic	Radiation	Mo *K*α (λ=0.71073)
Space Group	*P*-1	2Θ range/°	4.2 – 62.6
a/Å	15.6580(4)	Index range	$-21 \le h \le 21$, $-21 \le k \le 21$, $-22 \le l \le 22$
b/Å	15.6891(4)	Reflections Collected	75604
c/Å	16.5291(5)	Independent Reflections	17882
α/°	111.692(3)	Data/parameters/restraints	17882/858/94
β/°	102.885(2)	Goodness-of-fit on F^2	1.024
γ/°	102.286(2)	Final R-factor [$I \ge 2\sigma(I)$]	0.0724
Volume/Å^3	3477.15(18)	Final R indexes [all data]	R_1 = 0.1039, wR_2 = 0.2069
Z	2	Largest diff. peak/hole e/Å^3	0.82/-0.36

Single Crystal X-ray Diffraction Data for Catenane *rac*-**6**

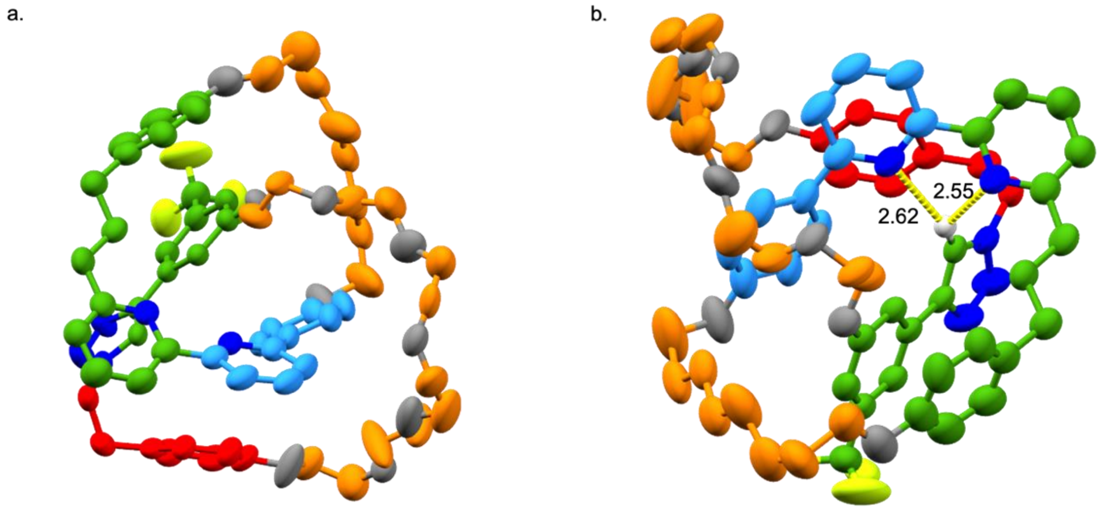

Figure S281. Ellipsoid plot of the asymmetric unit for *rac*-**6** with hydrogens omitted for clarity (a) and key intercomponent interactions highlighted (b). Thermal ellipsoids at 50% probability, disorder omitted for clarity.

Single crystals of *rac*-**6** were grown from a diethyl ether solution by slow evaporation, yielding colourless block crystals. Data was collected at 100 K on a Rigaku 007 HF diffractometer equipped with a HyPix 6000HE hybrid pixel array detector. Cell determination, data reduction, cell refinement, and absorption correction were processed by CrysalisPro[9]. The structure was solved within Olex2[10] by ShelXT[11] with refinement by ShelXL[12]. The asymmetric cell contained one molecule of $C_{62}H_{72}F_3N_5O_9$. The unit cell contained both enantiomers related by an inversion centre. This structure is characterised by extensive disorder. There was some indication that the data could be twinned by merohedry as well, and as such, we did an extensive twin search. We were unable to find any twin law using PLATON's TWINROTMAT[14] or Olex2's Extended Twin Search. Twin laws could be found using CrysAlisPro, however, the components were not stable and showed no separation along any dimension. Moreover, attempting to solve the structure using the twinned data reduction significantly diminished the solve. Consequently, while we cannot definitively rule out twinning, we believe that it is not the primary cause of the high wR2 and goodness of fit statistics; it seems that the extensive disorder of the structure is difficult to capture with standard modelling methods.

Table S2. Single crystal Diffraction and refinement statistics for *rac*-**6**

CCDC Number	2129422	μ/mm^{-1}	0.77
Empirical Formula	$C_{62}H_{72}F_3N_5O_9$	F(000)	2312
Formula Weight	1088.24	Crystal size/mm^3	0.26 × 0.17 × 0.10
Temperature/K	100	Radiation	Cu $K\alpha$ (λ=1.54178)
Crystal System	Monoclinic	2Θ range/°	6.4 – 140.6
Space Group	$P2_1/n$	Index range	$-11 \le h \le 11$, $-45 \le k \le 49$, $-17 \le l \le 17$
a/Å	9.4937(1)	Reflections Collected	51452
b/Å	40.8410(3)	Independent Reflections	10518
c/Å	14.5524(1)	Data/parameters/restraints	10518/879/787
β/°	98.961(1)	Goodness-of-fit on F^2	2.189
Volume/Å^3	5573.56(8)	Final R-factor [I ≥ 2σ(I)]	0.1175
Z	4	Final R indexes [all data]	R_1 = 0.1242, wR_2 = 0.4392
ρ_{calc} g/cm^3	1.297	Largest diff. peak/hole e/Å^3	1.14/-0.81

Single Crystal X-ray Diffraction Data for Catenane *rac-9*

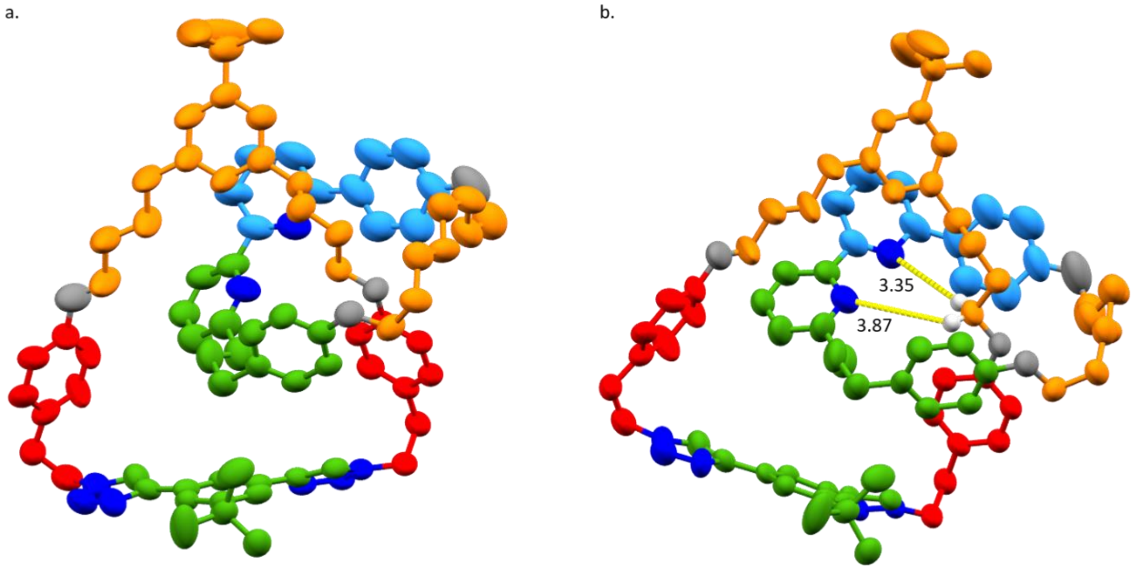

Figure S282. Ellipsoid plot of the asymmetric unit for *rac*-**9** with hydrogens omitted for clarity (a) and key intercomponent interactions highlighted (b). Thermal ellipsoids at 50% probability, disorder omitted for clarity.

Single crystals of *rac*-**9** were grown from a diethyl ether solution by slow evaporation, yielding thin colourless needle crystals. Data was collected at 100 K on a Rigaku 007 HF diffractometer equipped with a HyPix 6000HE hybrid pixel array detector. Cell determination, data reduction, cell refinement, and absorption correction were processed by CrysalisPro[9]. The structure was solved within Olex2[10] by ShelXT[11] with refinement by ShelXL[12]. The asymmetric unit contained one molecule of $C_{81}H_{94}N_8O_4$. The unit cell contained both enantiomers related by an inversion centre. This structure proved to be a challenging sample for SCXRD. Thin needle crystals are challenging for SXCRD and that was especially the case for this compound. Even using prolonged exposure times with a Cu source, the high angle data was consistently weaker than desired. All attempts have been made to collect better diffraction data, but so far these efforts have been unsuccessful. Nevertheless, the atom connectivity presented in the model is unambiguous and consistent with all other experimental data.

Table S3. Single crystal Diffraction and refinement statistics for *rac*-**9**

CCDC Number	2129424	μ/mm^{-1}	0.56
Empirical Formula	$C_{81}H_{94}N_8O_4$	F(000)	2672
Formula Weight	1243.64	Crystal size/mm^3	0.10 × 0.09 × 0.03
Temperature/K	100	Radiation	Cu *K*α (λ=1.54178)
Crystal System	Monoclinic	2Θ range/°	5.8 – 89.8
Space Group	*P*2$_1$/*n*	Index range	$-20 \le h \le 20$, $-10 \le k \le 10$, $-28 \le l \le 28$
a/Å	22.4213(13)	Reflections Collected	39952
b/Å	10.9157(4)	Independent Reflections	6076
c/Å	30.1770(13)	Data/parameters/restraints	6076/868/124
β/°	105.322(5)	Goodness-of-fit on F^2	1.018
Volume/Å^3	7123.1(6)	Final R-factor [$I \ge 2\sigma(I)$]	0.0781
Z	4	Final R indexes [all data]	R_1 = 0.1369, wR_2 = 0.2374
ρ_{calc} g/cm^3	1.160	Largest diff. peak/hole e/Å^3	0.47/-0.31

S8. Optimisation of conditions for the synthesis of catenanes 3

We assessed the outcome of the reaction between macrocycle **2** and pre-macrocycle **1a** (Scheme S4) by ^{1}H NMR analysis of the crude reaction product (Figure S283). Each spectrum contained three sets of signals that were integrated separately: i) a doublet at 8.20 ppm that corresponds to H_R (2H) of macrocycle **2**; ii) two singlets at 9.07 and 8.98 ppm that correspond to triazole proton H_a (1H) of the diastereomers of catenane **3a**; iii) a group of signals 9.48 – 9.65 ppm that we tentatively attribute (see Section S10) to the triazole protons, H_a of [2]catenane **S35a** and [3]catenane **4a**, referred to collectively as "oligomers". As we were interested in the fate of macrocycle **2**, and each signal between 9.48 and 9.65 ppm corresponds to one triazole encircled by one macrocycle, we took the sum of the integrals of all peaks in this region and treated them as 1H. Working on the assumption that macrocycle **2** was converted either to catenane **3a**, oligomers or remained unreacted, the distribution of macrocycle between these different species was then expressed as a percentage and the conversion of macrocycle **2** to catenane **3** was then used as a metric for the reaction optimisation (Table S4). The best conditions (entry 6) were then applied to the synthesis of catenanes **3b** and **3c** (entries 11 and 12).

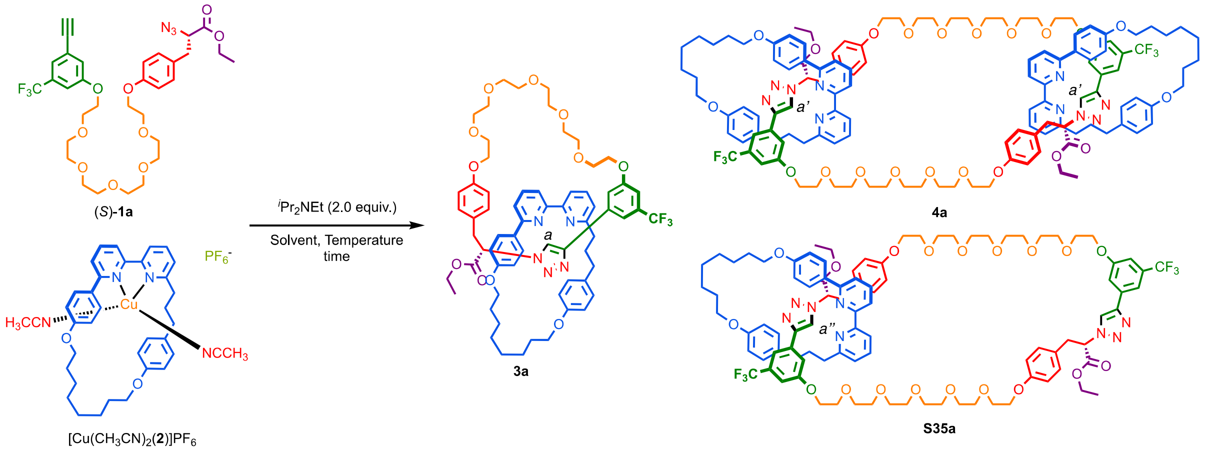

Scheme S4. Formation of by-products **4a** and **S35a** during AT-CuAAC synthesis of **3a**.

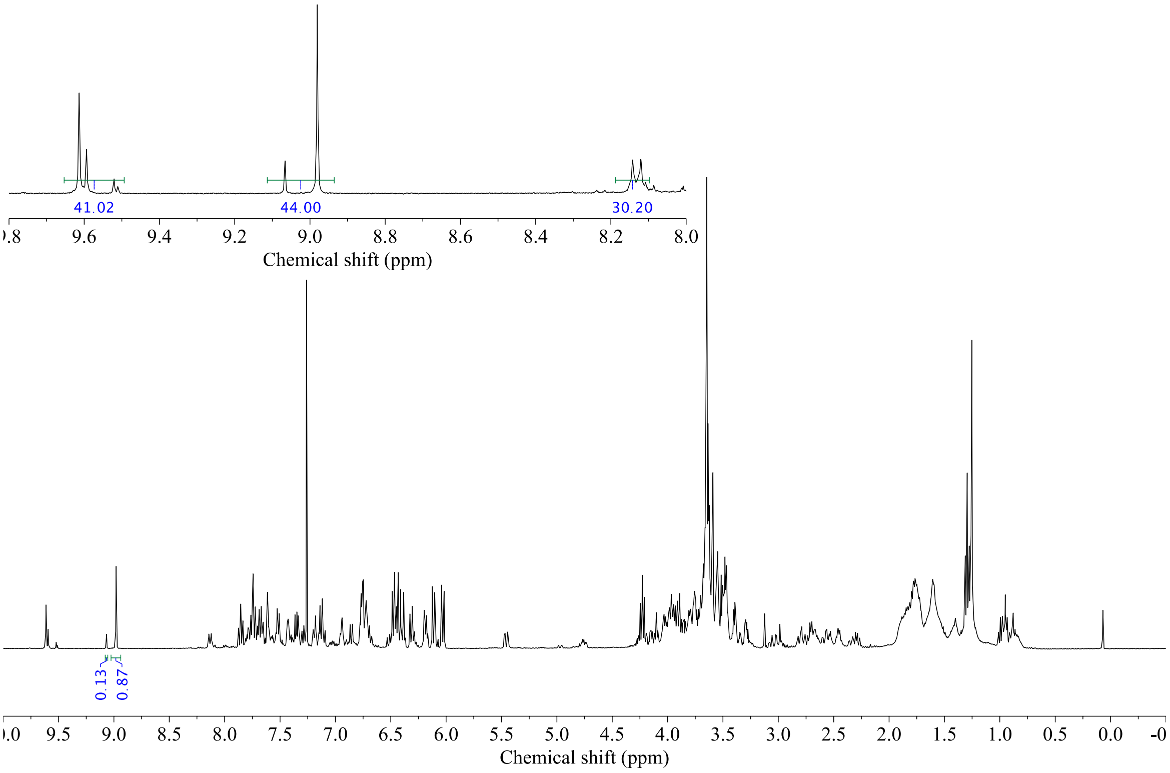

Figure S283. ^{1}H NMR of crude **3a** AT-CuAAC reaction under conditions as entry 6, Table S4 ($CDCl_3$, 400 MHz, 298 K). Signals between ~9.5 and 9.65 ppm are tentatively attributed to catenanes **4a** and **S35a**. Integration of these signals relative to those of **3a** (9.07 and 8.98 ppm) and **2** (2H doublet at 8.20 ppm), allowed the ratio of these species to be determined (Table S4).

Table S4. Optimisation of AT-CuAAC reaction conditions for macrocycle precursors **1a-c**.

Entry	precursor	Solvent	T / °C	Addition time / h	**2**	**3**	**oligomers**	*d.r.*
1	**1a**	$CHCl_3$	60	16	69%	27%	3%	87:13
2	**1a**	$CHCl_3$:EtOH (1:1)	60	4	34%	44%	22%	85:15
3	**1a**	$CHCl_3$:EtOH (1:1)	60	8	47%	37%	16%	81:19
4	**1a**	$CHCl_3$:EtOH (1:1)	60	16	45%	42%	13%	82:18
5	**1a**	$CHCl_3$:EtOH (1:1)	40	4	25%	41%	34%	88:12
6	**1a**	$CHCl_3$:EtOH (1:1)	25	4	15%	44%	41%	87:13
7	**1a**	$CHCl_3$:EtOH (1:1)	0	4	25%	27%	48%	86:14
8	**1a**	THF	25	4	27%	34%	38%	73:27
9	**1a**	CH_3CN	25	4	100%	-	-	-
10	**1a**	$CHCl_3$:EtOH (9:1)	25	4	40%	31%	29%	90:10
11	**1b**	$CHCl_3$:EtOH (1:1)	25	4	14%	30%	56%	91:9
12	**1c**	$CHCl_3$:EtOH (1:1)	25	4	77%	11%	12%	84:16

S9. Optimisation of Methods to Remove the Chiral Auxiliary

Radical Decarboxylation Reactions

Decarboxylation of the tyrosine moiety was proposed as a method for erasing the covalent stereocentre to obtain enantioenriched topologically chiral catenanes in which the mechanical bond provides the sole stereogenic unit. Test reactions were carried out with non-interlocked model compound (*S*)-**S31**. However, when treated with silver benzoate in presence of an oxidant,[15–17] (*S*)-**S31** gave only traces of the desired decarboxylated product **S34** (Scheme S5). Instead, the major products were **S37** and **S38**. Product **S37** is proposed to arise via homolytic cleavage of the proposed intermediate carbon-centered radical **S36**. Side-product **S38** results from the dimerisation of **S36**. Based on these initial results it seemed clear that radical pathways would not selectively yield the desired product in good yield and so we decided to focus on other approaches.

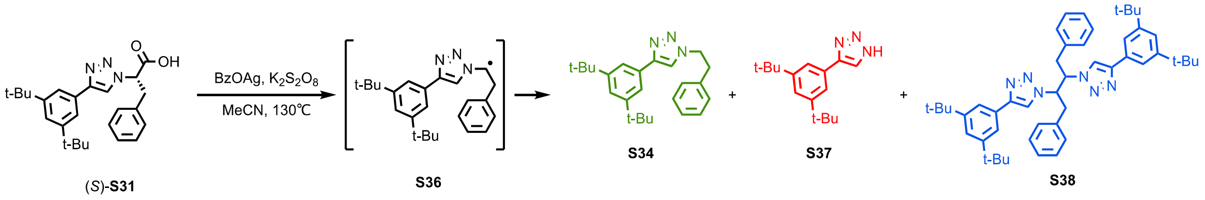

Scheme S5. Ag^{I}-mediated decarboxylation of (*S*)-**S31**.

Rh^{I}-Mediated Decarbonylation Reactions

The metal-catalysed decarbonylation of aldehydes is not a commonly employed synthetic methodology,[18] perhaps because it is usually addressed as an undesired side reaction in the context of hydroformylation[19] and hydroacylation[20] reactions. However this has not prevented its separate study as a useful methodology[21] and use in synthesis.[22–24] Model system (*S*)-**S30** was initially used to optimise the Rh^{I}-mediated decarbonylation reaction. Treating (*S*)-**S30** with a stoichiometric amount of highly-active catalyst $[Rh(COD)Cl]_2$ at 50 °C resulted in decomposition of the starting material (Table S5, entry 1).[23] Addition of bidentate phosphine ligands successfully afforded **S34** (entries 2, 5 and 6). Conversion increased over time, but catalyst turn over seemed to drop at extended periods of time, probably due to catalyst degradation (entries 3 and 4). Thus, increasing catalyst loading product **S34** could finally be obtained in 94% conversion as measured by ^{1}H NMR (entry 8). With this condition in hand, we moved on to try them on catenane **S39**.

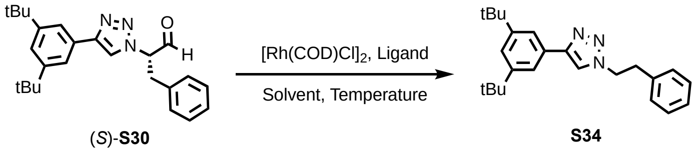

Scheme S6. Rh^{I}-decarbonylation of (*S*)-**S30**.

Table S5. Optimisation of reaction conditions on Scheme S6.

Entry	Rh^{I} eq.	Ligand (eq.)	Solvent	temperature (^{o}C)	time (h)	Conversion
1	1.00	-	PhMe	50	16	decomp.
2	0.05	(±)-BINAP (0.1)	DME	80	16	15%
3	0.05	(±)-BINAP (0.1)	DME	80	40	47%
4	0.05	(±)-BINAP (0.1)	DME	80	64	64%
5	0.05	dppp (0.1)	DME	80	16	7%
6	0.05	dppf (0.1)	DME	80	16	20%
7	0.20	(±)-BINAP (0.4)	DME	80	16	51%
8	0.20	(±)-BINAP (0.4)	DME	80	40	94%

Reduction of catenane **3b** with $LiAlH_4$ gave alcohol **5**. Oxidation of **5** with the Dess-Martin periodinane resulted in significant decomposition (72% of non-interlocked **2** relative to **S39** observed by ^{1}H NMR analysis of the crude product). This was improved by using a Swern oxidation protocol but non-interlocked **2** (40% relative to **S39**) was still observed. Serendipitously, during the synthesis of **5**, we observed that slow addition of $LiAlH_4$ (1.2 equiv.) to **3b** along the wall of the vial at -78 ^{o}C gave **S39** as the major product (~77% based on integration of ^{1}H NMR relative to other products) alongside catenane **5** (14%), macrocycle **2** (9%). Treating the crude reaction product under our optimised decarbonylation conditions successfully afforded catenane **6**, albeit in modest (17%) yield due to extensive decomposition to give non-interlocked **2** (Scheme S7). Although very convenient, the reduction step to give **S39** was poorly reproducible; different ratios of alcohol **5** and macrocycle **2** were obtained run-to-run alongside the desired product.

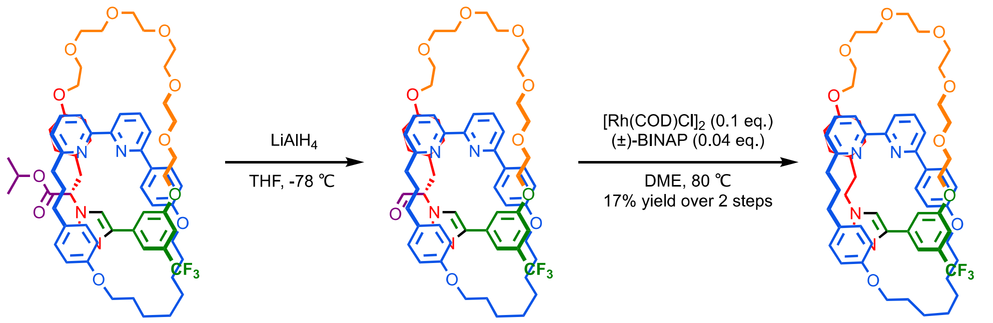

3b **S39** (S_{mt})-**6**

Scheme S7. Direct synthesis of catenane **S39** from **3b** and subsequent decarbonylation to yield catenane (S_{mt})-**6**.

In light of the results above, we examined a tandem Oppenauer-type oxidation–Rh(I)-decarbonylation procedure.[21] Pleasingly, these conditions allowed us to directly convert catenane **3b**, via catenane **5**, to catenane **6** in reproducibly in higher isolated yield over 2 steps (Scheme S8).

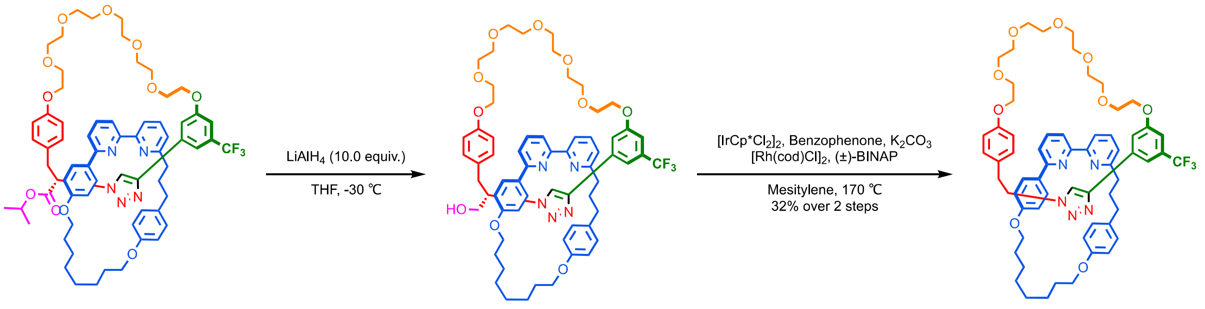

(S,S_{mt})-**3b** (S,S_{mt})-**5** (S_{mt})-**6**

Scheme S8. Reduction of **3b** and tandem Oppeanuer-type oxidation–Rh(I)-decarbonylation to yield catenane **6**.

S10. IDENTIFICATION OF BY-PRODUCTS S35 AND 4

The high ppm signals (9.48 – 9.65) observed in the ^{1}H NMR of the crude product of **2** and **1a** (Figure S110) are tentatively assigned as the triazole C-H resonances of [3]catenane **4a** and [2]catenane **S35a**, both of which arise by initial dimerization of **1a** followed by macrocyclisation, by analogy with previously reported structures combined with stereochemical analysis and supported by MS data.

The C-H resonance of a triazole unit encircled by a bipyridine macrocycle in a rotaxane or catenane is typically observed at high ppm (e.g. catenane **3a**) due to an H-bonding interaction between the bipyridine N and the triazole CH, suggesting that the signals between 9.48 and 9.65 ppm belong to interlocked structures. LC-MS analysis of the crude reaction product revealed species with *m/z* values consistent with [3]catenane **4a** and [2]catenane **S35a**. The five resonances observed by ^{1}H NMR between 9.48 and 9.65 ppm is consistent with this assignment due to the stereochemical complexity of these structures; both contain two fixed covalent stereogenic units and one (**S35a**) or two (**4a**) mechanical stereogenic units resulting in 2 and 3 diastereomers respectively (Figure S284).

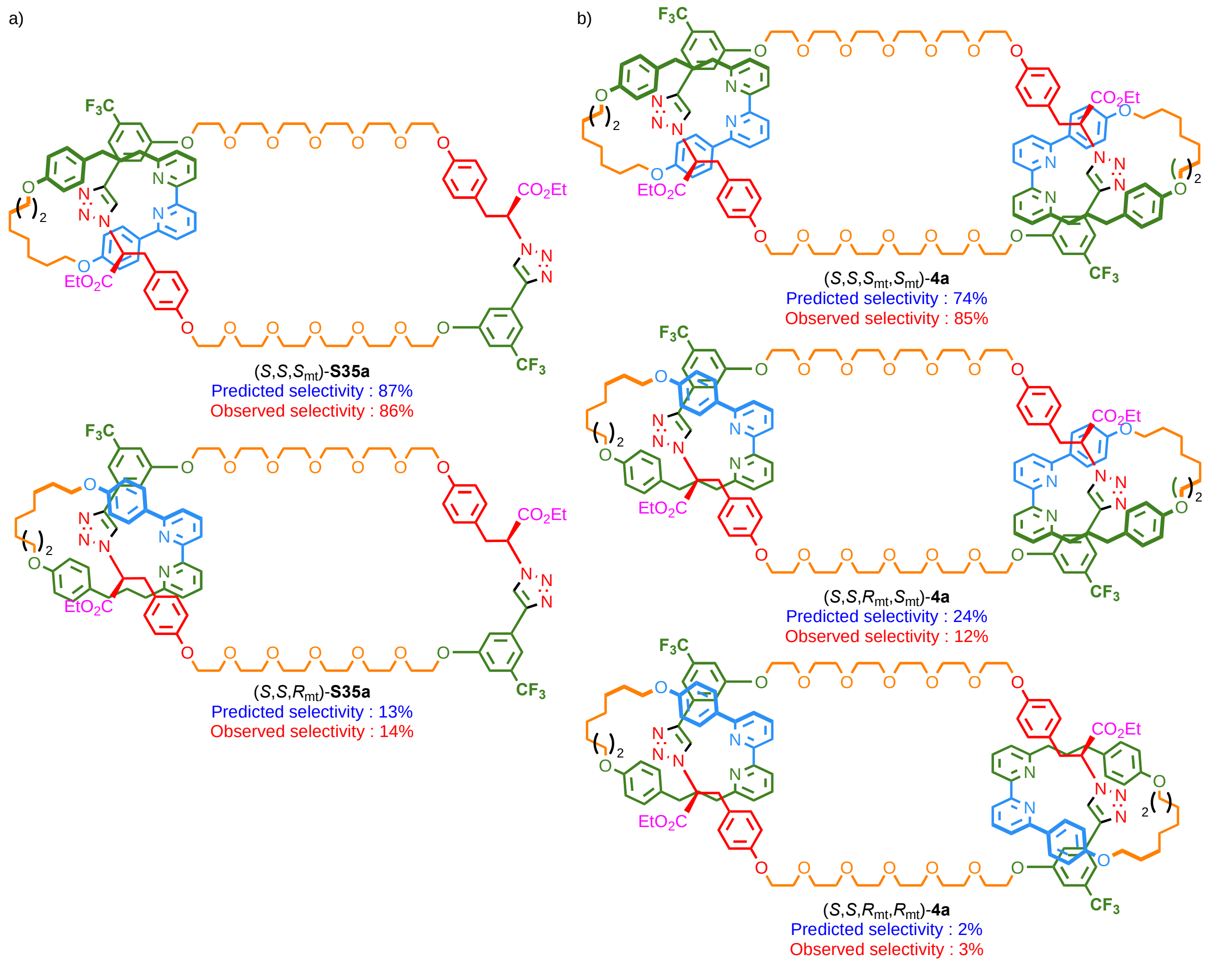

Figure S284. Available diastereomeric products of (a) [2]catenane **S35a** and (b) [3]catenane **4a** and their predicted relative abundance based on each mechanical bond being formed with comparable selectivity to **3a** (87 : 13 (S,S_{mt})-**3a** : (S,R_{mt})-**3a**).

If we assume, as a first approximation, that each mechanical bond is formed with the same selectivity as in **3a** (87 : 13), [2]catenane **S35a** is predicted to be formed as an 87 : 13 mixture of (S,S,S_{mt})-**S35a** and (S,S,R_{mt})-**S35a**, whereas [3]catenane **4b** is expected to be formed as a 74 : 24 : 2 mixture of (S,S,S_{mt},S_{mt})-**4a**, (S,S,R_{mt},S_{mt})-**4a** and (S,S,S_{mt},S_{mt})-**4a**. Line fitting of the region between 9.48 and 9.65 ppm allowed us to isolate five peaks that could be integrated (Figure S285). Pleasingly, the ratio between the peaks at 9.59 and 9.51 ppm was found to be 86 : 14 which is consistent with the ratio expected for **S35a** (Figure S285). The remaining peaks at 9.61, 9.60 and 9.52 ppm were found to have a relative integration of 86 : 2 : 12 (Figure S285). The agreement between the ratio predicted for [3]catenane **4a** using our simple model (stereoselectivity of the two bond forming steps is identical) and the values measured is reasonable given that the bond forming steps are unlikely to be fully independent in such a crowded structure.

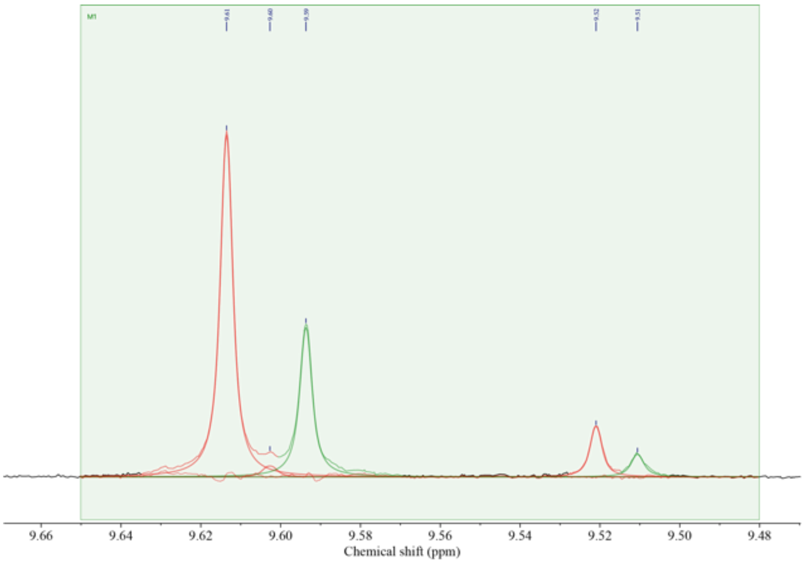

δ / ppm	Area	ratio
9.61	5946	85%
9.60	207	3%
9.59	2621	86%
9.52	850	12%
9.51	420	14%

Figure S285. Partial ^{1}H NMR of the crude reaction product of **1a** and **2** ($CDCl_3$, 400 MHz, 298 K) showing line-fit region containing signals proposed to be due to **S35a** and **4a** highlighted in green. Peaks corresponding to **4a** stereoisomers highlighted in red and peaks corresponding to **S35a** stereoisomers highlighted in green.

Attempts to isolate pure sample of **4a** and **S35a** met with mixed results. A fraction containing these species was obtained from the purification of **3a** after flushing the chromatography column with 10% MeOH in CH_2Cl_2 (Figure S286, middle spectrum). This material was then subjected to preparative TLC (10 × hexane-acetone-Et_3N 75 : 24 : 1, then hexane-acetone-Et_3N 70 : 29 : 1; bands then removed and extracted with 10% MeOH in CH_2Cl_2) which provided a sample containing predominantly **4a** (Figure S286, bottom spectrum) based on HPLC-HR-MS analysis (Figure S287). However, additional components were observed by HPLC and we were unable to isolate **S35a** and **4a** in analytically pure form. Given that the potential for co-conformational isomerism complicates matters (see section S11), no further purification was attempted.

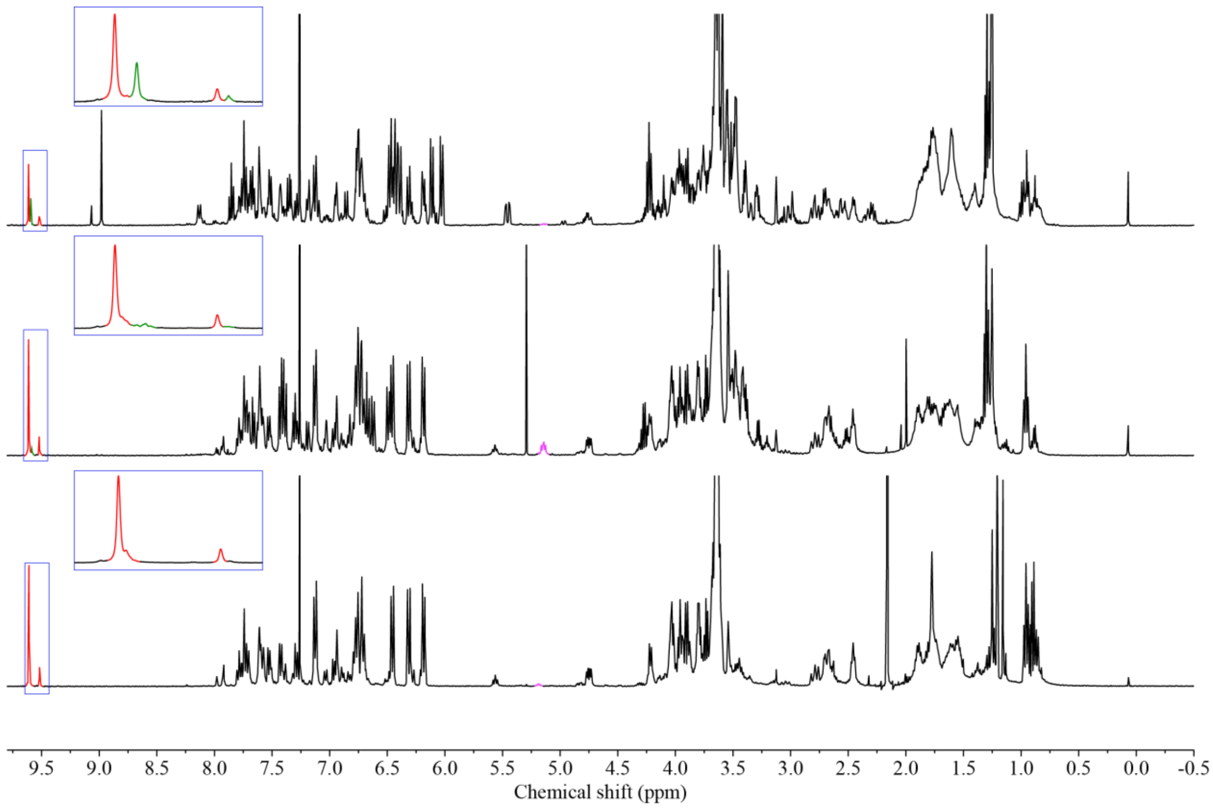

Figure S286. ^{1}H NMR of crude catenane **3a** (Top, $CDCl_3$, 400 MHz, 298 K), late column fractions (10% MeOH in CH_2Cl_2) containing a mixture of **S35a** and **4a** (Middle, $CDCl_3$, 400 MHz, 298 K) and a fraction containing predominantly [3]catenane **4a** (Bottom, $CDCl_3$, 400 MHz, 298 K). Signals tentatively assigned to triazole protons $H_{a'}$ (**4a**) and $H_{a''}$ (**S35a**) are highlighted in red and green respectively. Evidence of co-conformational isomerisation were observed – the signal highlighted in pink is consistent with H_b of molecules in which the macrocycle has escaped the triazole compartment, based on analysis of **3a** (section S11).

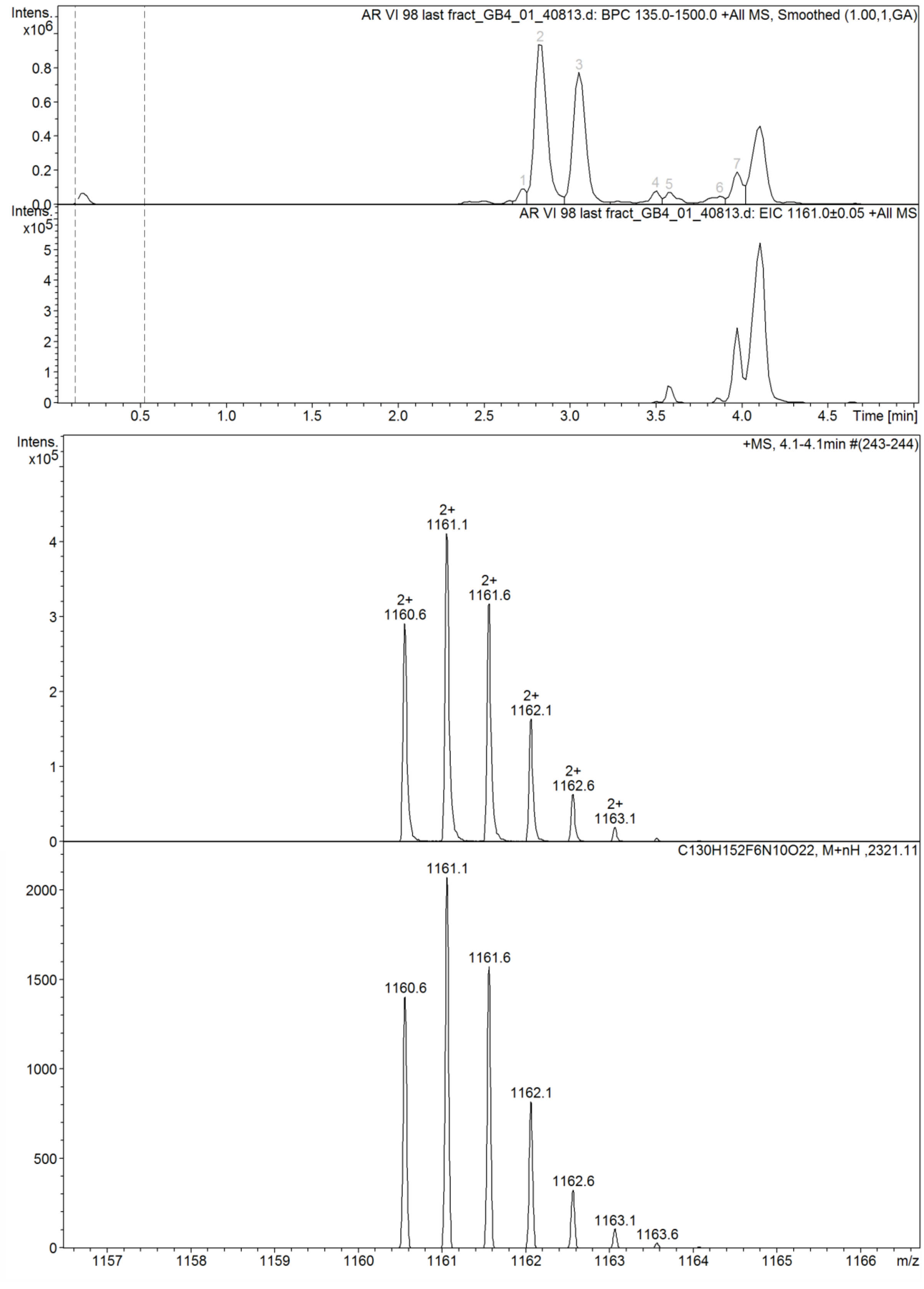

Figure S287. HPLC-HR-MS of a fraction containing **4a** (top). Isotope pattern of **4a** $C_{130}H_{152}F_6N_{10}O_{22}$ (bottom).

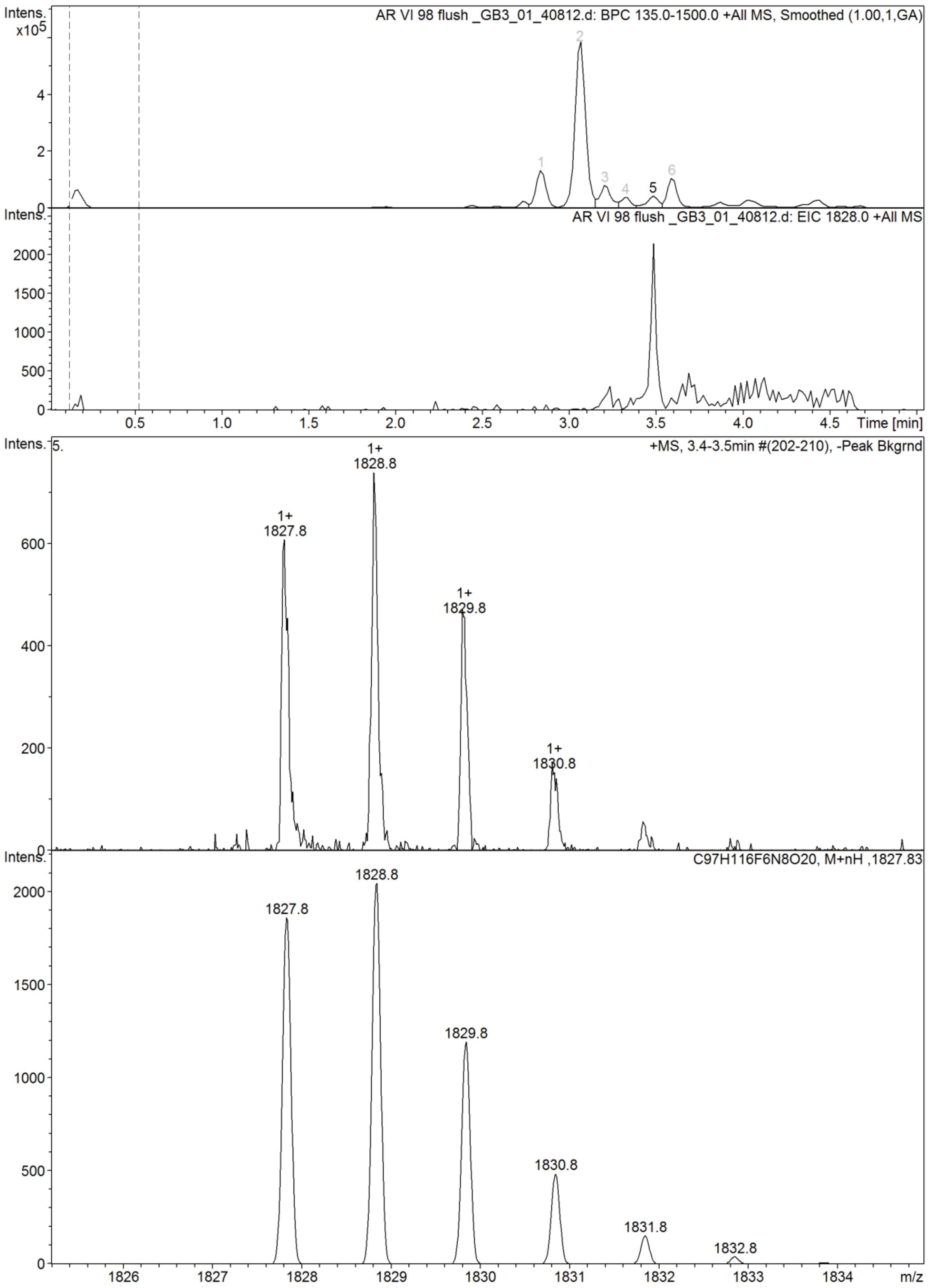

Figure S288. HPLC-HR-MS of the flushed fraction containing **S35a** (top). Isotope pattern of **S35a** $C_{97}H_{116}F_6N_8O_{20}$ (bottom).

S11. CO-CONFORMATIONAL EQUILIBRIUM OF CATENANES 3A AND 3B

^{1}H NMR analysis of purified samples of [2]catenanes **3a** and **3b** revealed that resonances associated with the ethylene glycol unit (4.07 - 3.15 ppm) appeared to have a higher integration than expected relative to other signals (Figure S111 and Figure S119). We initially considered that this may be due to small molecule contaminants (e.g. non-interlocked triazole containing macrocycle (*S*)-**S8**) but DOSY NMR (e.g **3b**, Figure S289) and LC-MS analysis suggested that no species of different mass or diffusion constant were present and no significant signals of (*S*)-**S8** could be identified. In addition, during attempts to purify [3]catenane **4a** (Section S10), a fraction was isolated containing a small amount of **3a** and a second species that contained similar signals but which lacked the high ppm singlet associated with the triazole unit encircled by a bipyridine macrocycle (Figure S292). LC-MS analysis suggested that the major species present had the same m/z as **3a** (Figure S291).

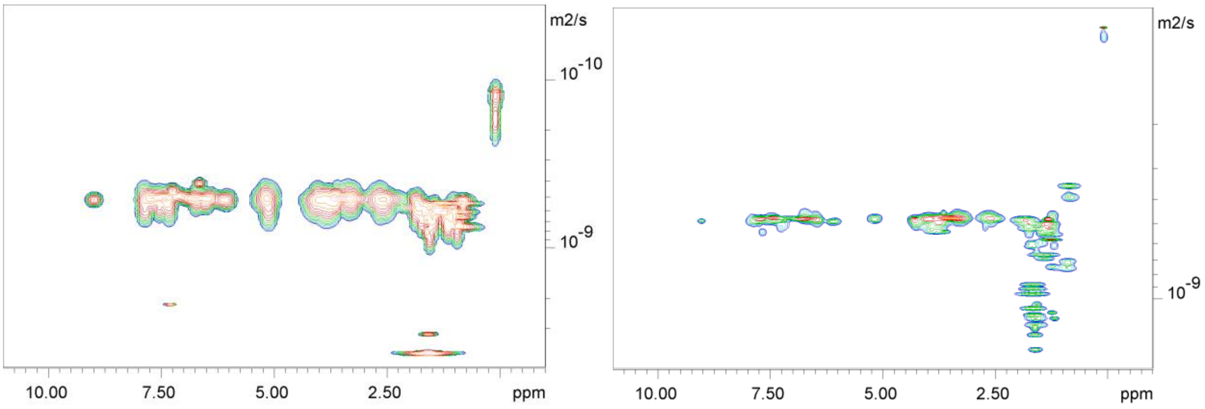

Figure S289. DOSY NMR ($CDCl_3$, 500 MHz, 298 K) of catenane **3b** (left) and **3a**$_{co\text{-}conf}$ (right)

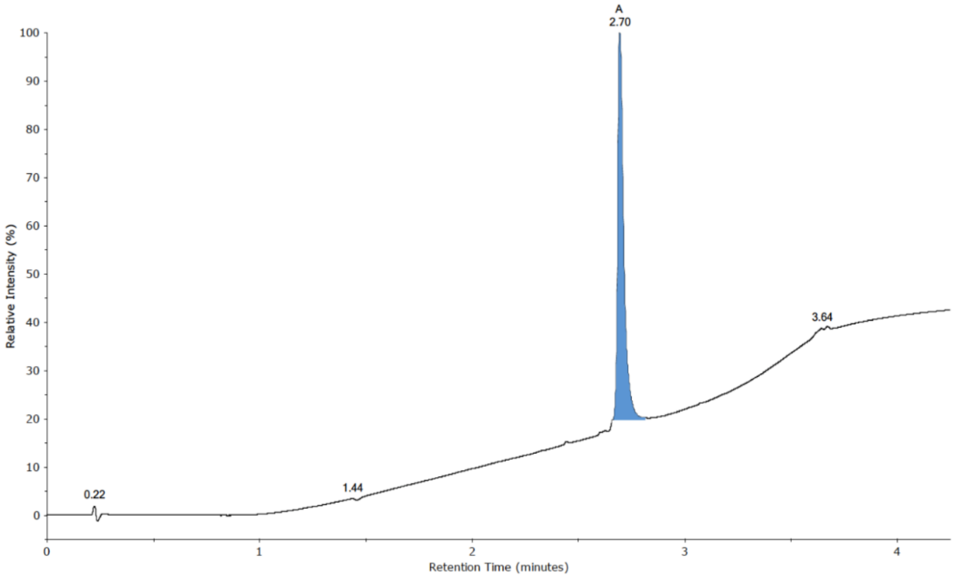

Figure S290. HPLC trace of **3a**$_{co\text{-}conf}$.

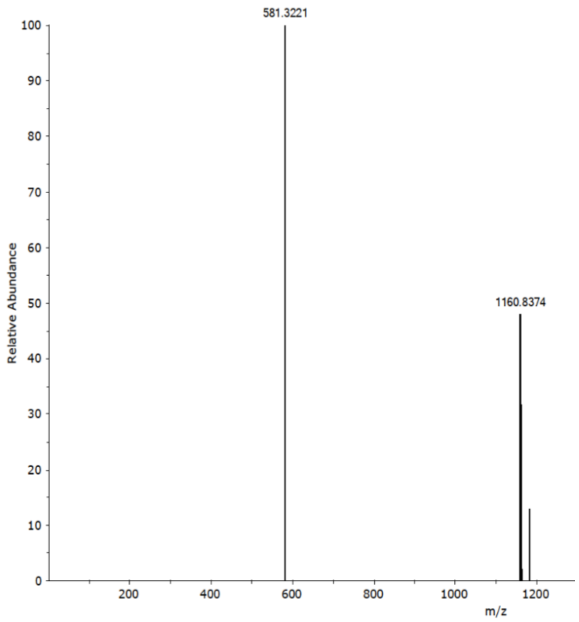

Figure S291. ESI-MS (positive mode) of **3a**$_{co\text{-}conf}$.

Simple physical models (CPK) suggested that the aryl-CF_3 unit and tyrosine ethyl ester units are not large enough to prevent co-conformational exchange in **3a** and thus the bipyridine macrocycle is not permanently trapped around the triazole unit after mechanical bond formation. Working on the assumption that the additional species was a co-conformational isomer of **3a**, **3a**$_{co\text{-}conf}$, detailed analysis of the ^{1}H NMR spectrum of the impure sample obtained suggested that triazole proton $H_{a'}$ was shifted 1.3 ppm upfield relative to its position in **3a**, consistent with a new structure, **3a**$_{co\text{-}conf}$, in which the bipyridine macrocycle no longer occupied the triazole compartment.

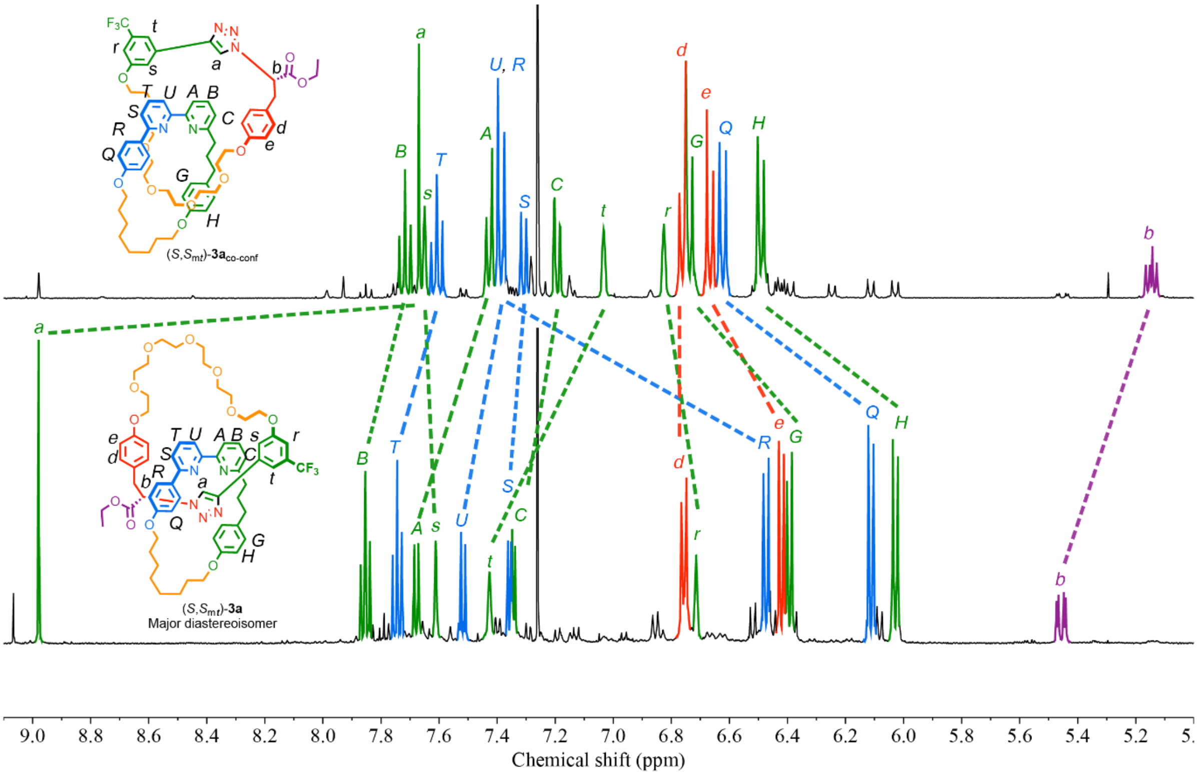

Figure S292. ^{1}H NMR of **3a** (Bottom, $CDCl_3$, 500 MHz, 298 K) and analytical sample of **3a**$_{co\text{-}conf}$ (top, $CDCl_3$, 400 MHz, 298 K).

In keeping with the proposal that **3a** can undergo co-conformational isomerism in which the macrocycle moves from the triazole-containing compartment and the ethylene glycol chain, the semi-purified sample **3a**$_{co\text{-}conf}$ was found to evolve to contain a higher proportion of **3a** over 10 months under solvent free conditions at rt (Figure S293), demonstrating that the isomerisation process is reversible.

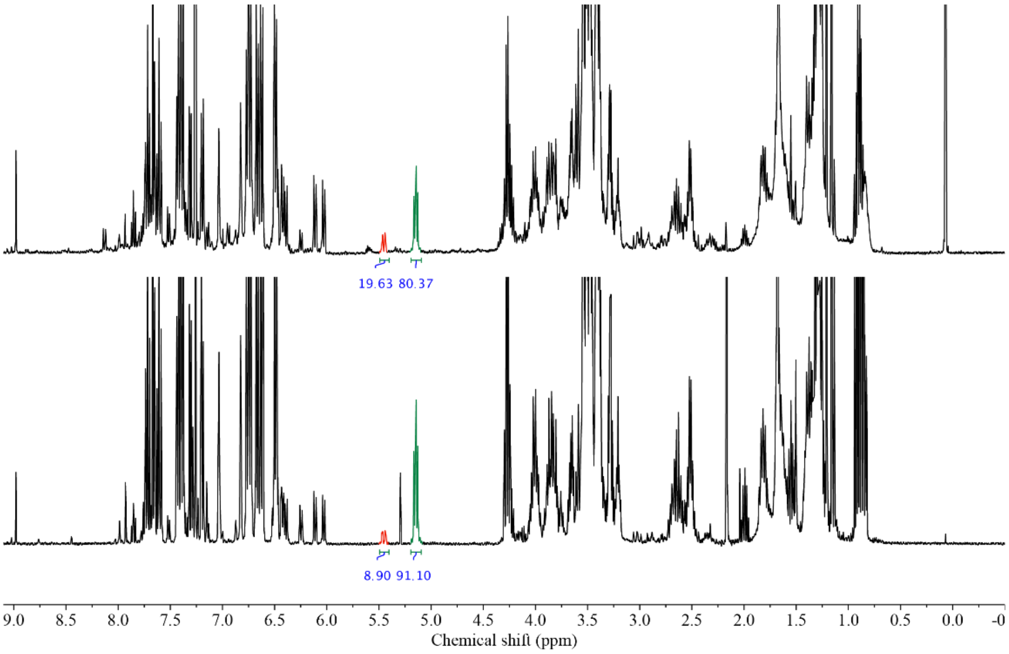

Figure S293. ^{1}H NMR ($CDCl_3$, 400 MHz, 298 K) of the sample containing **3a**$_{co\text{-}conf}$ after purification (bottom) and after 10 months storage under solvent-free conditions at rt (top). Ratios based on H_b of the major diastereoisomer for each co–conformation, **3a** in red and **3a**$_{co\text{-}conf}$ in green.

To further investigate the dynamic nature of **3a**, we studied its behaviour in different solvents on the grounds that co–conformational equilibria are biased by solvation. The sample reached equilibrium after 16 hours at 100 ºC in d_6-DMSO at which point a 91 : 9 ratio of **3a**$_{co\text{-}conf}$ to **3a** was observed (Figure S294).

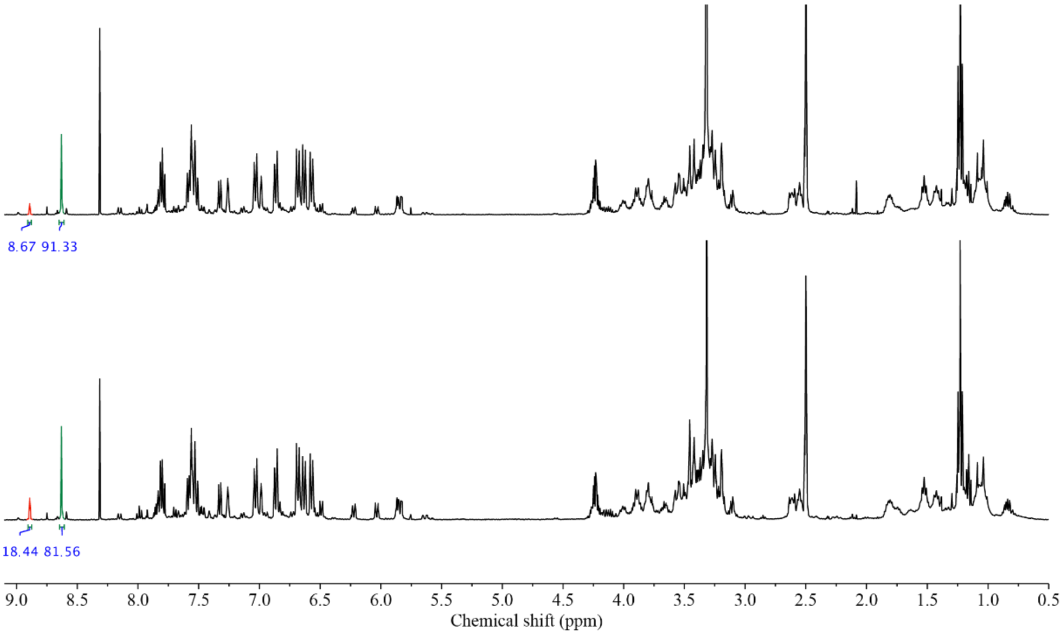

Figure S294. ^{1}H NMR (d_6-DMSO, 400 MHz, 298 K) of **3a**$_{co\text{-}conf}$ before (bottom) and after annealing at 100 ºC (top). Ratios based on H_a of the major diastereoisomer for each co–conformation, **3a** in red and **3a**$_{co\text{-}conf}$ in green.

Dissolving the same sample in $CDCl_3$ and heating at 60 ºC for 6 days resulted in a 73 : 27 ratio of **3a**$_{co\text{-}conf}$ to **3a** (Figure S295), further demonstrating that the isomerisation process is reversible.

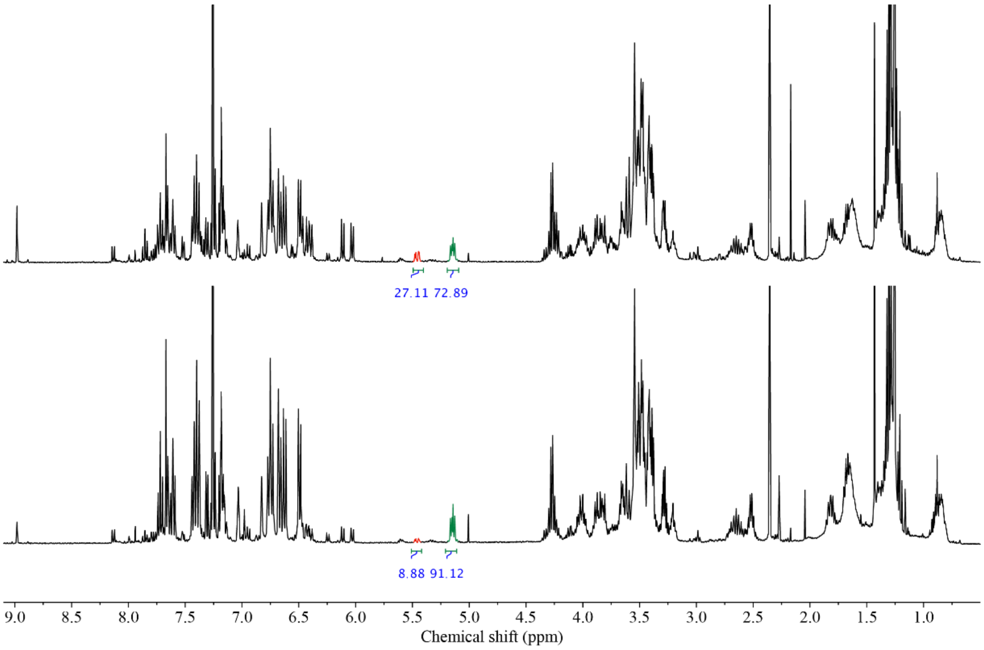

Figure S295. ^1H NMR ($CDCl_3$, 400 MHz, 298 K) stack of **3a**$_{co\text{-}conf}$ before (bottom) and after annealing at 60 ºC (top). Ratios based on H_b of the major diastereoisomer for each co–conformation, **3a** in red and **3a**$_{co\text{-}conf}$ in green.

Having obtained data to support the dynamic behaviour of **3a**, we turned our attention to a purified sample of **3b**. In this case, although previous results suggest that tyrosine isopropyl ester motif is too large for the macrocycle to move past,[2] the aryl-CF_3 unit is still small enough to allow the macrocycle to escape the triazole compartment. As in the case of **3a**, catenane **3b** evolved to a new species that is tentatively assigned as **3b**$_{co\text{-}conf}$ during storage under solvent–free conditions; ^1H NMR spectra of the same sample taken 5 months apart showed significant evolution a species tentatively assigned as **3b**$_{co\text{-}conf}$ (Figure S296).

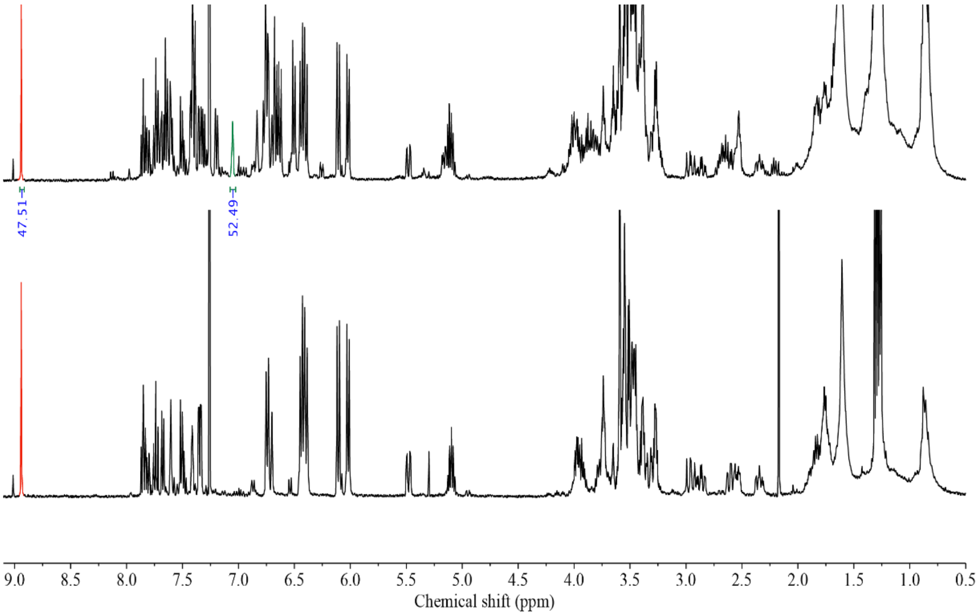

Figure S296. ^1H NMR ($CDCl_3$, 400 MHz, 298 K) stack of **3b** after purification (bottom) and storage (5 months, rt) (top). Ratios based on H_a of the major diastereoisomer for **3b** in red, and H_t of the major diastereoisomer for **3b**$_{co\text{-}conf}$ in green.

Heating a sample of **3b** in d_6-DMSO (100 ºC, 48 h) produced a 94 : 6 mixture of $\mathbf{3b}_{\text{co-conf}}$: **3b** (Figure S297).

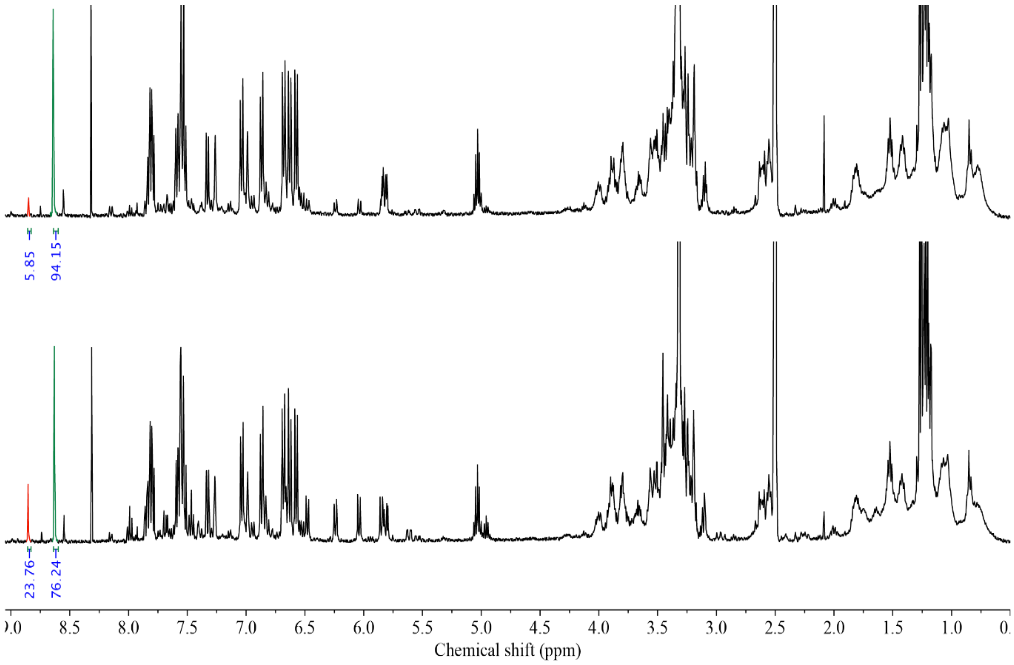

Figure S297. ^{1}H NMR (d_6-DMSO, 400 MHz, 298 K) of a mixture of **3b** and $\mathbf{3b}_{\text{co-conf}}$ before (bottom) and after annealing at 100 ºC (top). Ratios based on H_a of the major diastereoisomer for each co–conformation, **3b** in red and $\mathbf{3b}_{\text{co-conf}}$ in green.

Dissolving the same sample in $CDCl_3$ and annealing (60 ºC, 6 days) produced an 80 : 20 mixture of $\mathbf{3b}_{\text{co-conf}}$ and **3b** (Figure S298), demonstrating that the process is reversible.

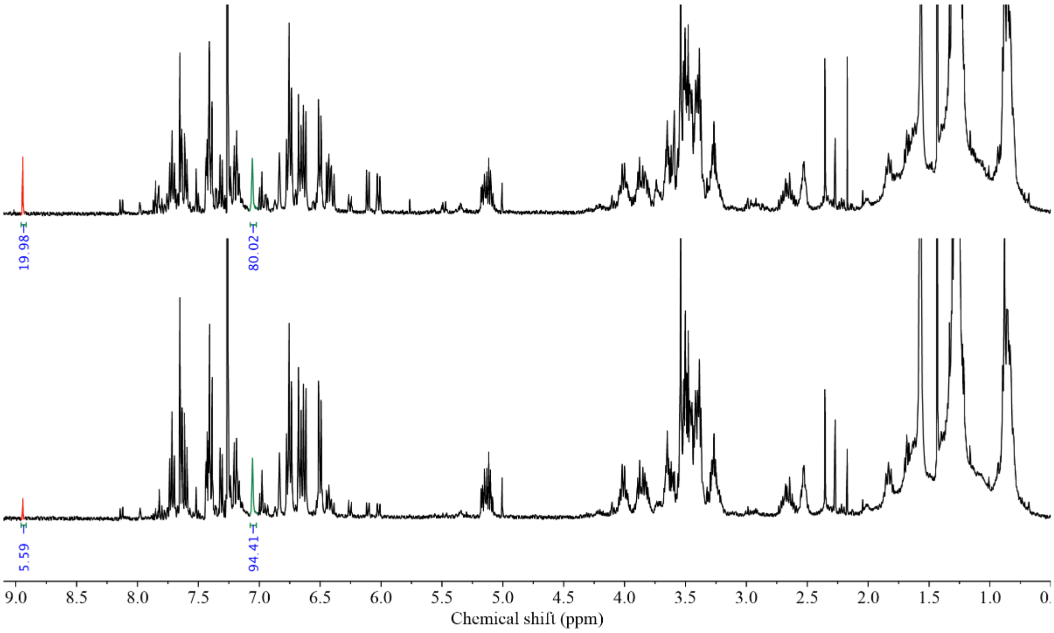

Figure S298. ^{1}H NMR ($CDCl_3$, 400 MHz, 298 K) stack of a mixture of **3b** and $\mathbf{3b}_{\text{co-conf}}$ before (bottom) and after annealing at 60 ºC (top). Ratios based on H_a of the major diastereoisomer for **3b** in red, and H_t of the major diastereoisomer for $\mathbf{3c}_{\text{co-conf}}$ in green.

Based on these preliminary studies, the samples of catenanes **3a** and **3b** isolated after the AT-CuAAC reaction contain a meta-stable isomer of these products. When stored at rt under solvent free conditions for prolonged periods or when heated in solvent, these isomerise to a new species with the same mass and

diffusion constant that we propose to be a second co-conformation, $\mathbf{3}_{\text{co-conf}}$, in which the macrocycle encircles the ethylene glycol unit. These other species, along with the corresponding minor diastereoisomers, are thought to account for the observed over-integration of signals between 4.07 and 3.15 ppm in the isolated samples of catenanes **3a** and **3b** (Figure S111) and (Figure S119).

S12. References

(1) Pigorsch, A.; Köckerling, M. The Crystallization of Extended Niobium-Cluster Framework Compounds: A Novel Approach Using Ionic Liquids. *Cryst. Growth Des.* **2016**, *16* (8), 4240–4246. https://doi.org/10.1021/acs.cgd.6b00225.

(2) de Juan, A.; Lozano, D.; Heard, A. W.; Jinks, M. A.; Suarez, J. M.; Tizzard, G. J.; Goldup, S. M. A Chiral Interlocking Auxiliary Strategy for the Synthesis of Mechanically Planar Chiral Rotaxanes. *Nat. Chem.* **2021**. https://doi.org/10.1038/s41557-021-00825-9.

(3) Sekiguchi, H.; Muranaka, K.; Osada, A.; Ichikawa, S.; Matsuda, A. Efficient Synthesis of Hsp90 Inhibitor Dimers as Potential Antitumor Agents. *Bioorganic Med. Chem.* **2010**, *18* (15), 5732–5737. https://doi.org/10.1016/j.bmc.2010.05.075.

(4) Chen, P.; Cheng, P. T. W.; Alam, M.; Beyer, B. D.; Bisacchi, G. S.; Dejneka, T.; Evans, A. J.; Greytok, J. A.; Hermsmeier, M. A.; Humphreys, W. G.; Jacobs, G. A.; Kocy, O.; Lin, P. F.; Lis, K. A.; Marella, M. A.; Ryono, D. E.; Sheaffer, A. K.; Spergel, S. H.; Sun, C. Q.; Tino, J. A.; Vite, G.; Colonno, R. J.; Zahler, R.; Barrish, J. C. Aminodiol HIV Protease Inhibitors. Synthesis and Structure - Activity Relationships of P1/P1′ Compounds: Correlation between Lipophilicity and Cytotoxicity. *J. Med. Chem.* **1996**, *39* (10), 1991–2007. https://doi.org/10.1021/jm950717a.

(5) Tse, Y. C.; Docker, A.; Zhang, Z.; Beer, P. D. Lithium Halide Ion-Pair Recognition with Halogen Bonding and Chalcogen Bonding Heteroditopic Macrocycles. *Chem. Commun.* **2021**, *57* (40), 4950–4953. https://doi.org/10.1039/d1cc01287h.

(6) Qing, Z.; Takacs, J. M. Click-Connected Ligand Scaffolds: Macrocyclic Chelates for Asymmetric Hydrogenation. *Org. Lett.* **2008**, *10* (4), 545–548. https://doi.org/10.1021/ol702890s.

(7) Jinks, M. A.; de Juan, A.; Denis, M.; Fletcher, C. J.; Galli, M.; Jamieson, E. M. G.; Modicom, F.; Zhang, Z.; Goldup, S. M. Stereoselective Synthesis of Mechanically Planar Chiral Rotaxanes. *Angew. Chem. Int. Ed. Engl.* **2018**, *57* (45), 14806–14810. https://doi.org/10.1002/anie.201808990.

(8) Soltani Rad, M. N.; Behrouz, S.; Khalafi-Nezhad, A. A Simple One-Pot Procedure for the Direct Conversion of Alcohols into Azides Using TsIm. *Tetrahedron Lett.* **2007**, *48* (19), 3445–3449. https://doi.org/10.1016/j.tetlet.2007.03.049.

(9) Rigaku Oxford Diffraction. CrysAlisPro Software System. Rigaku Oxford Diffraction 2021.

(10) Dolomanov, O. V.; Bourhis, L. J.; Gildea, R. J.; Howard, J. A. K.; Puschmann, H. OLEX2: A Complete Structure Solution, Refinement and Analysis Program. *J. Appl. Crystallogr.* **2009**, *42* (2), 339–341. https://doi.org/10.1107/S0021889808042726.

(11) Sheldrick, G. M. SHELXT - Integrated Space-Group and Crystal-Structure Determination. *Acta Crystallogr.*

Sect. A Found. Crystallogr. **2015**, *71* (1), 3–8. https://doi.org/10.1107/S2053273314026370.

(12) Sheldrick, G. M. Crystal Structure Refinement with SHELXL. *Acta Crystallogr. Sect. C Struct. Chem.* **2015**, *71* (Md), 3–8. https://doi.org/10.1107/S2053229614024218.

(13) Spek, A. L. PLATON SQUEEZE: A Tool for the Calculation of the Disordered Solvent Contribution to the Calculated Structure Factors. *Acta Crystallogr. Sect. C Struct. Chem.* **2015**, *71*, 9–18. https://doi.org/10.1107/S2053229614024929.

(14) Spek, A. L. PLATON, A Multipurpose Crystallographic Tool. Utrecht University: Utrecht, The Netherlands 2005.

(15) Johnson, R. G.; Ingham, R. K. The Degradation of Carboxylic Acid Salts by Means of Halogen: The Hunsdiecker Reaction. *Chem. Rev.* **1956**, *56* (2), 219–269. https://doi.org/10.1021/cr50008a002.

(16) Wang, Z.; Zhu, L.; Yin, F.; Su, Z.; Li, Z.; Li, C. Silver-Catalyzed Decarboxylative Chlorination of Aliphatic Carboxylic Acids. *J. Am. Chem. Soc.* **2012**, *134* (9), 4258–4263. https://doi.org/10.1021/ja210361z.

(17) Font, M.; Quibell, J. M.; Perry, G. J. P.; Larrosa, I. The Use of Carboxylic Acids as Traceless Directing Groups for Regioselective C-H Bond Functionalisation. *Chem. Commun.* **2017**, *53* (41), 5584–5597. https://doi.org/10.1039/c7cc01755c.

(18) Walborsky, H. M.; Allen, L. E. The Stereochemistry of Tris(Triphenylphosphine) Rhodium Chloride Decarbonylation of Aldehydes. *J. Am. Chem. Soc.* **1971**, *93* (21), 5465–5468. https://doi.org/10.1021/ja00750a026.

(19) Van Leeuwen, P. W. N. M.; Kamer, P. C. J.; Reek, J. N. H.; Dierkes, P. Ligand Bite Angle Effects in Metal-Catalyzed C-C Bond Formation. *Chem. Rev.* **2000**, *100* (8), 2741–2769. https://doi.org/10.1021/cr9902704.

(20) Willis, M. C. Transition Metal Catalyzed Alkene and Alkyne Hydroacylation. *Chem. Rev.* **2010**, *110* (2), 725–748. https://doi.org/10.1021/cr900096x.

(21) Kreis, M.; Palmelund, A.; Bunch, L.; Madsen, R. A General and Convenient Method for the Rhodium-Catalyzed Decarbonylation of Aldehydes. *Adv. Synth. Catal.* **2006**, *348* (15), 2148–2154. https://doi.org/10.1002/adsc.200600228.

(22) Hu, P.; Snyder, S. A. Enantiospecific Total Synthesis of the Highly Strained (-)-Presilphiperfolan-8-Ol via a Pd-Catalyzed Tandem Cyclization. *J. Am. Chem. Soc.* **2017**, *139* (14), 5007–5010. https://doi.org/10.1021/jacs.7b01454.

(23) Miura, T.; Nakamuro, T.; Nagata, Y.; Moriyama, D.; Stewart, S. G.; Murakami, M. Asymmetric Synthesis and Stereochemical Assignment of 12C/13C Isotopomers. *J. Am. Chem. Soc.* **2019**, *141* (34), 13341–13345. https://doi.org/10.1021/jacs.9b07181.

(24) Zeng, C. M.; Han, M.; Covey, D. F. Neurosteroid Analogues. 7. A Synthetic Route for the Conversion of 5β- Methyl-3-Ketosteroids into 7(S)-Methyl-Substituted Analogues of Neuroactive Benz[e]Indenes. *J. Org. Chem.* **2000**, *65* (7), 2264–2266. https://doi.org/10.1021/jo991953m.
